# Transcriptomic analysis of flower induction for long-day pitaya by supplementary lighting in short-day winter season

**DOI:** 10.1186/s12864-020-6726-6

**Published:** 2020-04-29

**Authors:** Rui Xiong, Chengli Liu, Min Xu, Shuang-shuang Wei, Jia-quan Huang, Hua Tang

**Affiliations:** Hainan Key Laboratory for Sustainable Utilization of Tropical Bioresources, College of Tropical Crops, Hainan University, No. 58 Renmin Avenue, Haikou, 570228 Hainan P. R. China

**Keywords:** Pitaya, Supplementary lighting-induced flowering, de novo RNA-Seq analysis, Differentially expressed genes, qRT-PCR

## Abstract

**Background:**

Pitayas are currently attracting considerable interest as a tropical fruit with numerous health benefits. However, as a long-day plant, pitaya plants cannot flower in the winter season from November to April in Hainan, China. To harvest pitayas with high economic value in the winter season, it is necessary to provide supplementary lighting at night to induce flowering. To further explore the molecular regulating mechanisms of flower induction in pitaya plants exposed to supplementary lighting, we used de novo RNA sequencing-based transcriptomic analysis for four stages of pitaya plants subjected to light induction.

**Results:**

We assembled 68,113 unigenes in total, comprising 29,782 unigenes with functional annotations in the NR database, 20,716 annotations in SwissProt, 18,088 annotations in KOG, and 11,059 annotations in KEGG. Comparisons between different samples revealed different numbers of significantly differentially expressed genes (DEGs). A number of DEGs involved in energy metabolism-related processes and plant hormone signaling were detected. Moreover, we identified many *CONSTANS-LIKE*, *FLOWERING LOCUS T*, and other DEGs involved in the direct regulation of flowering including *CDF* and *TCP*, which function as typical transcription factor genes in the flowering process. At the transcriptomic level, we verified 13 DEGs with different functions in the time-course response to light-induced flowering by quantitative reverse-transcription PCR analysis.

**Conclusions:**

The identified DEGs may include some key genes controlling the pitaya floral-induction network, the flower induction and development is very complicated, and it involves photoperiod perception and different phytohormone signaling. These findings will increase our understanding to the molecular mechanism of floral regulation of long-day pitaya plants in short-day winter season induced by supplementary lighting.

## Background

The pitaya plant (*Hylocereus polyrhizus* Britton & Rose) belongs to the Cactaceae family, genus *Hylocereus*, and is a long-day and diploid tropical plant. At present, the academic community generally believes that it originated in southern Mexico. Nowadays, it is geographically widespread, occurring in Israel, China, and Southeast Asia [1]. The earliest report on pitayas was published in the *General and Natural History of the Indies* in 1535, and clearly records that this fruit may be edible [2]. The stem of the pitaya plant, with its waxy surface, can store a large amount of water. Its leaves have metamorphosed into thorns as a result of long-term environmental stresses. Furthermore, crassulacean acid metabolism (CAM) carbon assimilation allows the pitaya plant to endure extreme environments [3]. The fruits are prized for not only their fine appearance and striking colors but also the potentially beneficial effects of *H. polyrhizus* fruit betacyanins on high-fat-associated human diseases [4]. A number of studies have been undertaken to ascertain the nutritional value of pitayas, revealing that they are a rich source of organic acids, sugars, polyphenols, vitamin C [5], and antioxidants [6].

When the plant has passed from the juvenile to the adult phase and from the vegetative to the reproductive stage, it can be induced to flower. The pitaya flower is spectacular, as it elongates at dusk and blooms at night, and then withers after pollination in the daytime. The pitaya plant is a long-day plant. In the northern hemisphere, pitaya flowers bloom from May to the end of October [7]. However, pitaya plants cannot flower in the winter season from November to April in Hainan, China, owing to the short-day conditions. To harvest pitayas with high economic value in the winter season, supplementary lighting must be provided at night to induce flowering. To produce pitayas in the short-day winter season, it is necessary to supply sufficient light time to promote flowering [8]. Supplementary lighting is a useful and proven technology for farmers to produce enough pitayas to meet consumer demand.

Flowering is regulated by an integrated network of biochemical and genetic pathways, such as the photoperiod pathway, vernalization pathway, gibberellic acid (GA) pathway, and autonomous pathway. Various input signals activate the signal transduction pathways that control flowering time. The photoperiod pathway refers to the response to day length and light quality and has been demonstrated to play a crucial role in controlling flowering in *Arabidopsis* [9]. In the presence of light, *CONSTANS* (*CO*) has been proven to be a key regulator of flower promotion through the activation of *FLOWERING LOCUS T* (*FT*) and *SUPPRESSOR OF OVEREXPRESSION OF CONSTANS 1* (*SOC1*) under long-day conditions [10]. Subsequently, FT associates with FLOWERING LOCUS D (FD), and the FT–FD complex promotes expression of the downstream floral meristem identity genes, such as *APETALA1* (*AP1*) and *LEAFY* (*LFY*), to induce flowering [11]. In *Arabidopsis*, plant hormone signaling (via GA, jasmonic acid (JA), and auxins, etc.) is required for floral induction. Furthermore, GA directly promotes *SOC1* and *LFY* expression and increases the level of *SOC1* mRNA, which in turn activates the downstream genes *LFY* and *AP1* to induce flowering [12]. One study found that tomato plants with *jai1*–*1* mutants exhibit delayed flower opening, indicating that JA acts as a positive regulator of flowering [13]. In addition, indole-3-acetic acid (IAA) is likely to be involved in the mechanism that controls the growth of the male gametophyte to the egg cell in the ovule, demonstrating that auxins are a major controlling signal that synchronize flower development in *Arabidopsis* [14].

The phenomenon of flowering is an important developmental process for higher plants. Flowering plays important roles in the plant growth cycle, especially in the transition from vegetative growth to reproductive development. To date, there have been few studies related to the molecular and genetic mechanisms of flowering in pitaya plants. It is an interesting thing to investigate the kind of supplement light which can able to promote pitaya flowering. In this study, we conducted a light supplementation experiment to induce flower bud differentiation in the short-day winter season. Four different stages of flower development in pitaya plants were sampled for high-throughput transcriptome sequencing and comparative analysis to screen out differentially expressed genes (DEGs) related to flower induction. It is beneficial to explore the genes related to flower induction and explore the molecular mechanism of flowering in pitaya plants, a unique long-day CAM plant.

## Results

### Flowering induction of pitaya by supplementary light treatment in the winter season

**Fig. 1:**
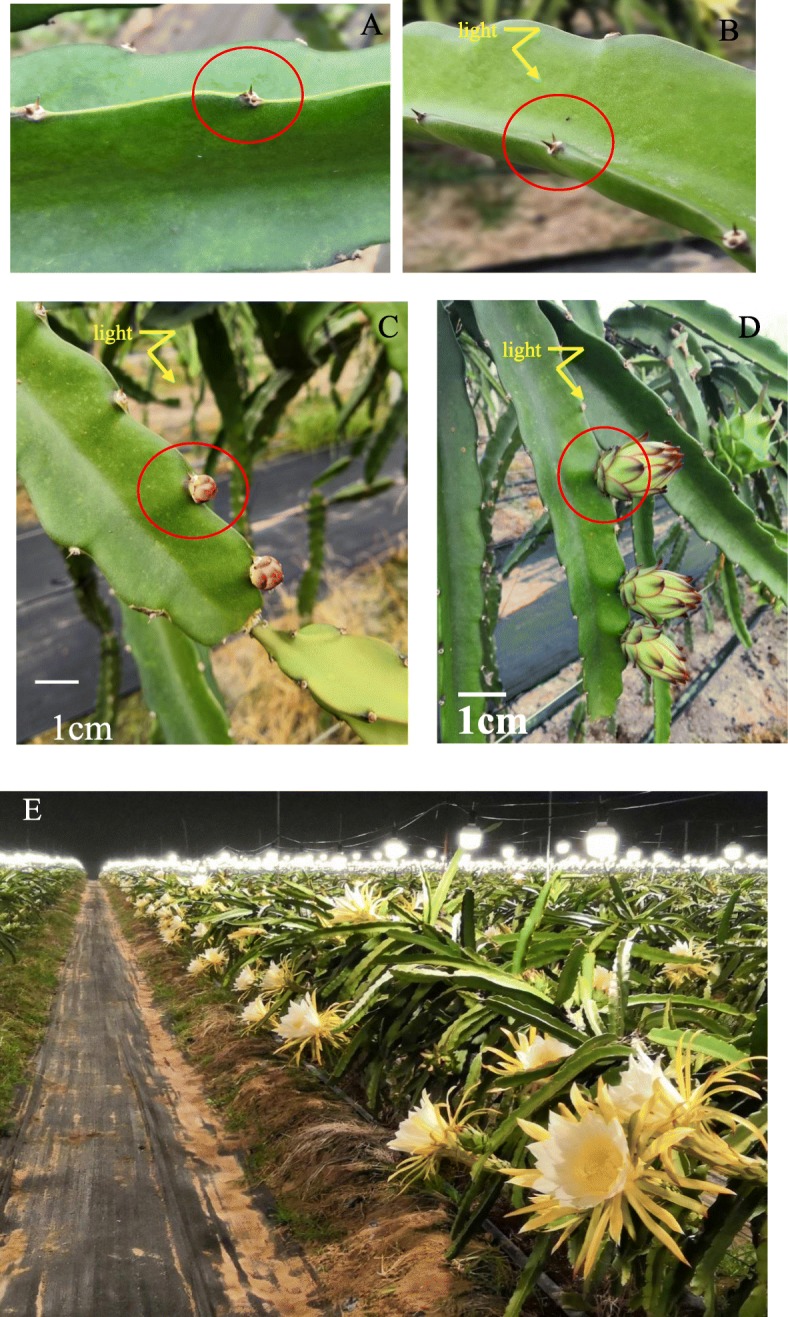
Supplementary light experiment. **a** NL represents the control group (no light, no flowering); **b** L0 represents the no-flowering stage in the light-treated group; **c** L1 represents the flower bud stage under light treatment; **d** L2 represents one-week post-bud stage under light treatment; **e** flowering of pitaya plants induced by light treatment in the winter season. The red cycle indicated the sampling position on plant

Our flowering induction experiment results demonstrate that sufficient supplementary light can induce flowering in pitaya plants if the minimum air temperature is ≥15 °C. In this experiment, NL represented the control group (no light, no flowering) (Fig. 1a), L0 represented no flowering in the light-treated group (Fig. 1b), L1 represented the flower bud stage in the light-treated group (Fig. 1c), and L2 represented 1 week after the bud stage in the light-treated group (Fig. 1d). Approximately 20–25 days after the buds appeared, the pitaya flowers were in full bloom at night (Fig. 1e), signifying the success of this flower induction system with respect to pitaya production in the winter season.

### Sequencing, de novo assembly, and annotation

To elucidate the underlying molecular events involved in light-induced flowering, we sent pitaya plant samples from the NL, L0, L1, and L2 stages to the Gene Denovo Biotechnology Co. (Guangzhou, China) for de novo assembly of RNA-Seq data. More than 39 Gb of raw data reads were retrieved. The numbers of high-quality clean reads in different samples were showed as Supplementary Fig. S1. These findings indicate that the proportion of high-quality clean reads after filtration of each sample was relatively high, signifying that the sequencing quality was good. The de novo assembly resulted in a total of 60,580,077 bases, but only 68,113 unigenes (not less than 200 nt) had an N50 of 1730 nt (Table 1).

**Table 1:** Results of assembly

Gene number	GC percentage	N50	Max length	Min length	Average length	Total assembled bases
68,113	41.96	1730	36,165	201	889	60,580,077

**Fig. 2:**
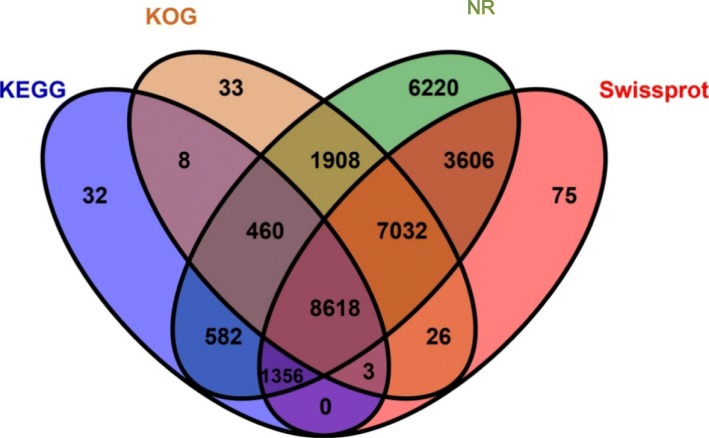
Venn diagram of the four database annotations

The average length of our assembly was 889 nt, of which the maximum length of a unigene was 36,165 nt, the minimum length was 201 nt, and the GC (guanine-cytosine) percentage was 41.96%. Supplementary S2 presents the size distribution of the unigenes. To search for homologous sequences, annotation analysis was performed on the unigenes using four public databases: KOG, KEGG, SwissProt, and NR. In total, 29,959 unigenes (after removing duplicates) were found in at least one of these databases (Table 2). There were 29,782 unigenes with functional annotations in the NR database, 20,716 annotations in SwissProt, 18,088 annotations in KOG, and 11,059 annotations in KEGG. We also observed that 8618 unigenes were annotated by all four major databases (Fig. 2). Moreover, we found only 6220, 75, 33, and 32 unique annotations in the NR, SwissProt, KOG, and KEGG databases, respectively.

**Table 2:** Results of annotations in four databases

Total unigenes	NR	SwissProt	KOG	KEGG	Genes with annotation	Genes without annotation
68,113	29,782	20,716	18,088	11,059	29,959	38,164

### Global analysis of differential expression profiling

The abundance of each gene was determined by counting reads per kilobase per million reads (RPKM) to infer the expression level. Therefore, this method could be used to directly compare the differences in gene expression among the samples. The correlation between the gene expression levels among the samples was a key criterion in determining whether the experiments were reliable and whether the samples chosen were suitable. If one sample had a high degree of similarity to another, the correlation between them would be very close to 1. We calculated the correlation values between samples based on the RPKM results. According to the standard recommended by the Encyclopedia of DNA Elements (ENCODE) project [15], the square of the correlation value should be ≥0.92 (under ideal experimental conditions and with suitable samples).

**Fig. 3:**
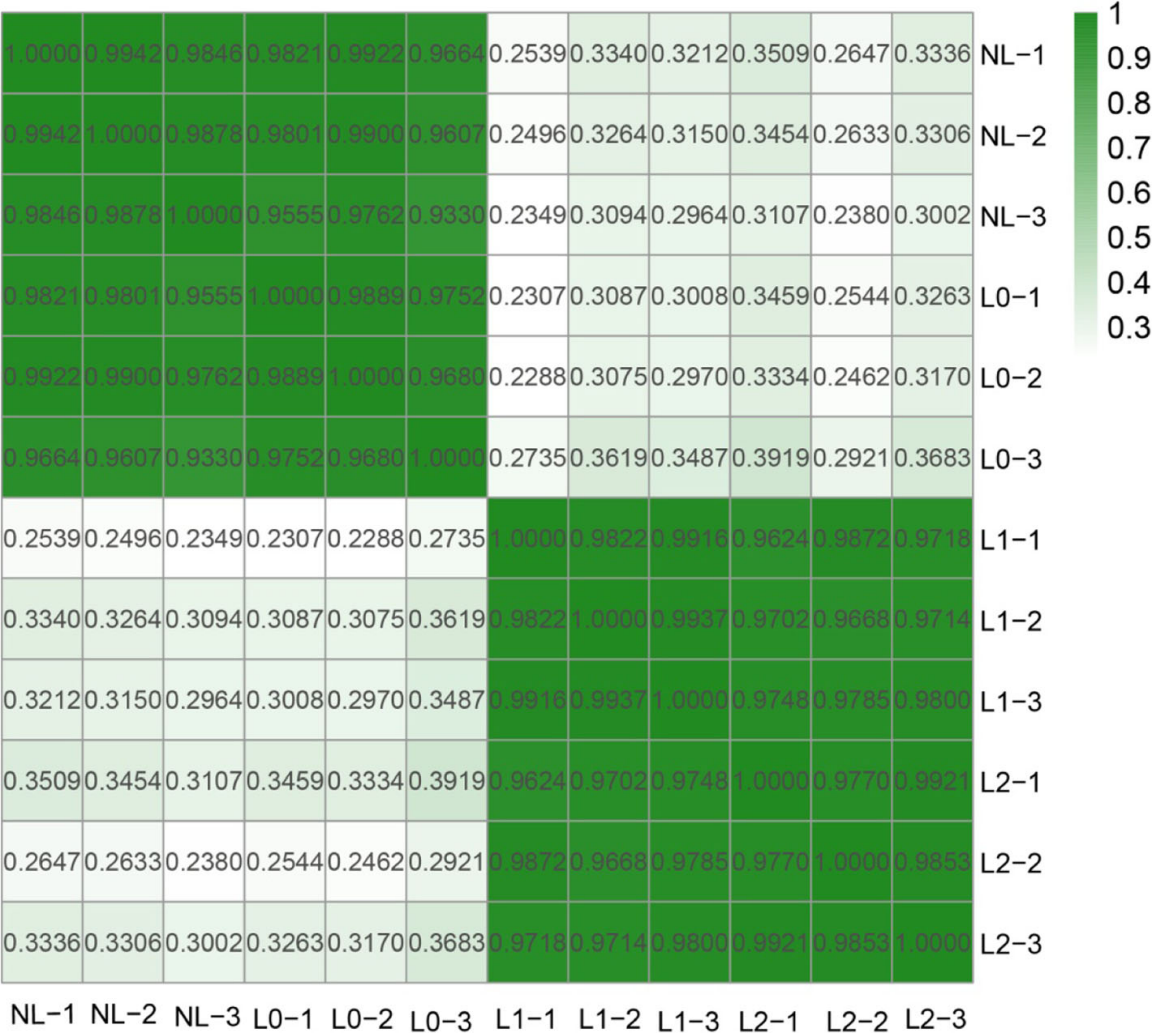
Relationship analysis of the samples. NL: control group (no light, no flowering); L0: stage of no flowering under light treatment; L1: flower bud stage under light treatment; L2: one-week post-bud stage under light treatment

The heatmap of correlations for these samples is presented in Fig. 3. From the figure, we can see that the correlation between the three repetitions for the NL stage was above 0.98, and that between the three repetitions for the L0 stage was above 0.96. Similarly, the correlations between the three repetitions for the L1 and L2 stages were above 0.99 and 0.97, respectively, signifying that the levels of expression between all biological replicates were highly correlated. Conversely, we found a low level of correlation between the different samples, indicating that the differences between the four samples were significant.

**Fig. 4:**
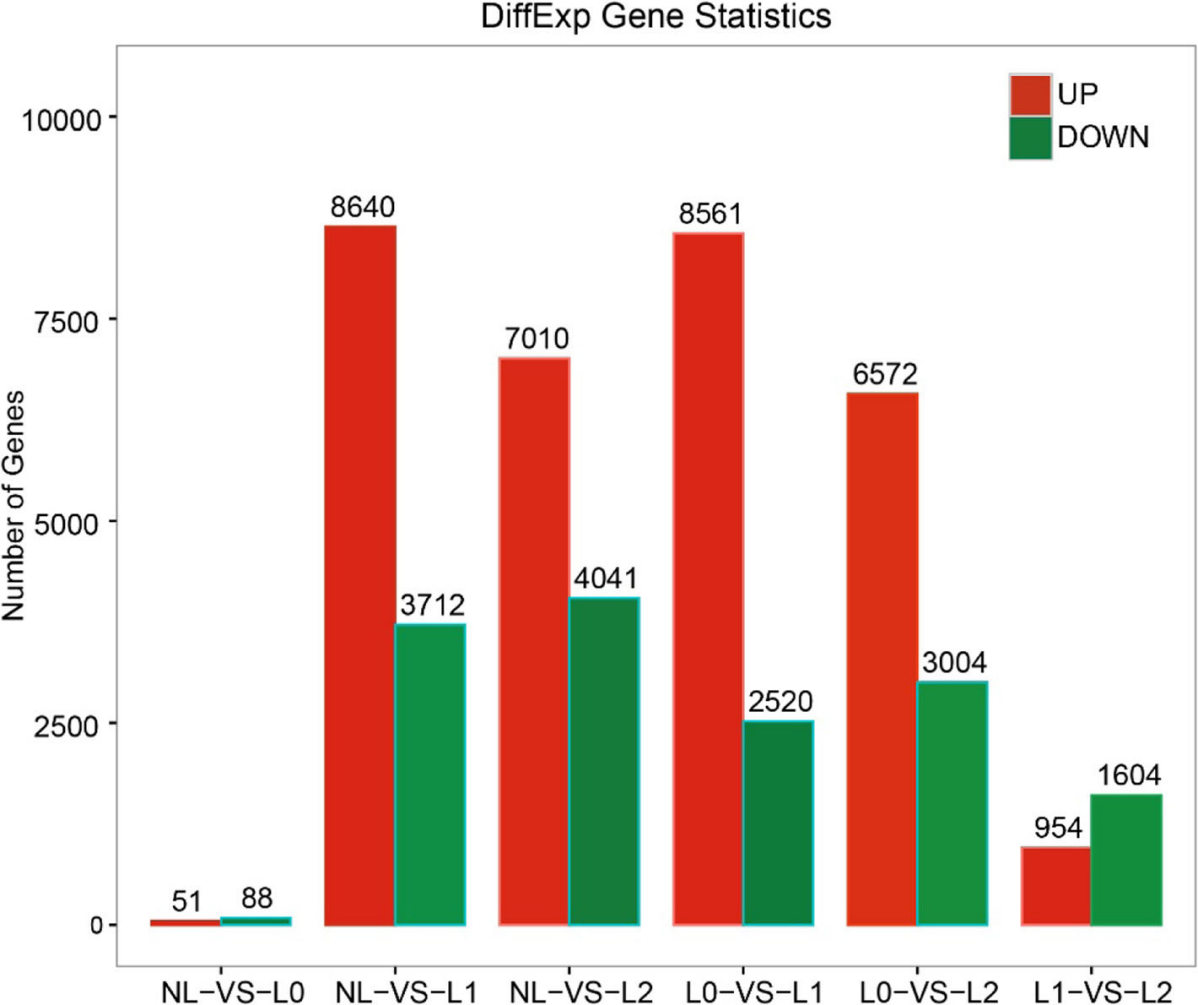
Gene statistics for DEGs in different samples. NL: control group (no light, no flowering); L0: stage of no flowering under light treatment; L1: flower bud stage under light treatment; L2: one-week post-bud stage under light treatment

In the present study, comparisons between different samples resulted in different numbers of DEGs (Fig. 4). When NL and L0 were compared, we found only 51 up-regulated genes and 88 down-regulated genes, totaling 139 DEGs. A total of 12,352 DEGs were obtained when we compared NL and L1, of which 8640 genes were up-regulated and 3712 genes were down-regulated. Thus, compared with the NL sample, the number of DEGs in the L1 sample was approximately nine times that in the L0 sample, of which the number of up-regulated genes in the L1 sample was 169 times that in the L0 sample and the number of down-regulated genes in the L1 sample was 41 times that in the L0 sample. Similar results were found in other comparisons of the groups.

Gene ontology (GO) functional enrichment analysis was applied in our subsequent experiment to annotate the expression patterns of the DEGs to the selected GO terms: molecular function, cellular component, and biological process. As a single gene often has multiple different functions, the same gene could appear under different terms, and each histogram could be statistically independent of any other.

In our study, 50 GO terms relating to molecular function, 30 GO terms relating to cellular component, and 158 GO terms relating to biological process were enriched in the L0 stage, compared with the NL stage, after light treatment (Supplementary file S3, S4, S5). Under the same light treatment, DEGs associated with the L1 stage were enriched by 399, 162, and 1042 GO terms relating to molecular function, cellular component, and biological process, respectively (Supplementary file S6, S7, S8). Moreover, findings regarding the GO analysis of other groups were similar to those described above. Most notably, we found that the main enriched terms of all groups were the same, regardless of ontologies. In particular, “cellular component” was mainly related to the cell part, membrane, and organelle; “molecular function” was mainly related to binding, catalytic activity, and transport activity; and “biological process” was mainly related to cellular, metabolic, and single-organism transport, suggesting that DEGs primarily enriched in those terms may be associated with the induction of pitaya blossoms.

To further investigate the gene expression profiles, we performed KEGG pathway analysis to determine some of the DEGs involved in important biochemical, metabolic, and signal transduction pathways during the light-induced flowering of pitaya plants. For example, two auxin response factors (unigene0028447 and unigene0002811) were up-regulated, two genes related to JA (unigene0030164 and unigene0033693) were up-regulated, and five genes related to starch and sucrose metabolism (unigene0052631, unigene0036015, unigene0040385, unigene0041472, and unigene0035017) were also up-regulated.

### Energy-related genes in the process of flowering induction

During our investigation of the light-induced flowering of pitaya plants, we focused on the DEGs in the NL, L0, and L1 stages. Before a plant flowers, essential organic nutrients accumulate to accommodate the large consumption of nutrients during flowering. Soluble sugars and soluble proteins are important nutrients that are closely associated with plant flowering. Large increases in ATP concentration initiated by changes in environmental conditions, especially different forms of light treatment, have been observed [16].

Our analysis revealed that two genes, unigene0046195 (beta-glucosidase BoGH3B-like) and unigene0014135 (ATP synthase), both associated with the Krebs cycle of glucose metabolism, were up-regulated in the L0 stage compared with the NL stage. Moreover, the expression levels of eight genes (unigene0006689, unigene0031088, unigene0044754, unigene0009228, unigene0045801, unigene0047216, unigene0050048, and unigene0053090) involved in sucrose synthesis were all increased in the L1 stage, three of which were up-regulated in the L1 stage compared with the L0 stage. These findings related to energy metabolism demonstrate that large amounts of sucrose are needed as an energy supply during the flowering process.

### Plant hormone and signal transduction related genes

Three types of phytohormones—auxins, JA, and brassinosteroids (BRs)—have been individually connected to floral timing, although they have not been extensively studied. These hormones can be transported as signal molecules in plants, producing signal transduction cascades that direct a series of metabolic activities.

**Fig. 5:**
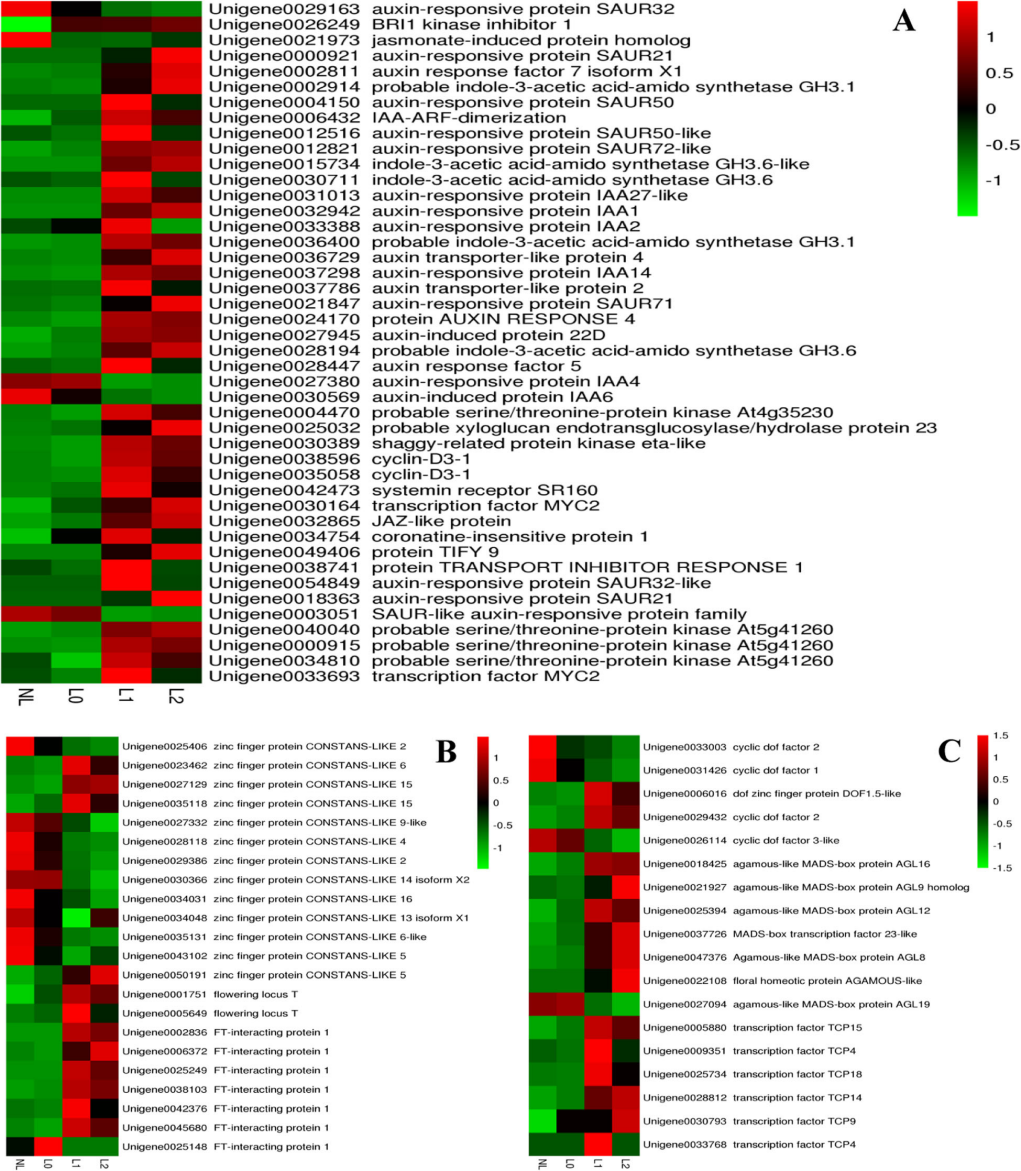
Heatmap of the DEGs associated with various plant hormones(**a**), *CO* and *FT*(**b**), main TFs involved pitaya flowering(**c**).. NL: control group (no light, no flowering); L0: stage of no flowering under light treatment; L1: flower bud stage under light treatment; L2: one-week post-bud stage under light treatment

Genetic evidence has revealed that the polar transport of auxins in *Arabidopsis* controls flower formation and differentiation [17]. Genes regulating floral organ development and gynoecium vascularization have been discovered, indicating the probable involvement of auxins in flower development. In the present study (Fig. 5a and Supplementary file S9), we found an auxin-related gene (unigene0029163) that was significantly down-regulated in L0 compared with NL. Twenty-three genes were significantly expressed in L1, of which two genes (unigene0027380 and unigene0030569) were down-regulated and 21 genes were up-regulated.

A previous study reported that the flowering of the BR biosynthesis-deficient *det2* mutant of *Arabidopsis thaliana* is delayed by at least ten days compared with the wild type; the level of endogenous BR was 10% lower than that of the wild type [18]. Compared with NL, only one up-regulated gene was detected in L0 (unigene0026249), whereas six up-regulated DEGs (unigene0004470, unigene0025032, unigene0030389, unigene0038596, unigene0035058, and unigene0042473) were observed in L1. Furthermore, compared with L0, eight up-regulated genes involved in the BR metabolism pathway were observed in L1.

*In general,* JA is known to activate transcription factors (TFs) that trigger a large-scale response to various abiotic and biotic stresses, and it also plays an important role in regulating flower opening. Jasmonate-ZIM domain (JAZ) proteins have been shown to regulate the levels of JA to counteract flower abscission in *Nicotiana attenuata* plants [19]. In the present study, we found one gene that was down-regulated (unigene0021973) in L0 and four genes that were up-regulated (unigene0030164, unigene0032865, unigene0034754, and unigene0049406) in L1. In addition, only one up-regulated gene (unigene0033693) was detected in L1 compared with L0.

GA hormone signaling is very important to floral transition. In the present study, we have found three DELLA protein GAI genes (Unigene0005738, Unigene0031663, Unigene0046182) were significantly up-regulated during the flower induction and development stages from NL vs L1, L0 vs L1 (Supplementary file S9).

### *CO* and *FT* or other directly regulating DEGs

In plants, seasonal changes in day length are sensed by the leaves, which initiate long-distance signaling that induces flowering at the shoot apex. In *Arabidopsis*, *FLOWERING LOCUS T* (*FT*), now known as florigen, is crucial for the appropriate timing of flowering [20], and *CONSTANS* (*CO*) plays a key role in the activation of *FT* expression [21]. Thus, *FT* acts partially downstream of *CONSTANS* (*CO*), which promotes flowering in response to long days. In the present study (Fig. 5b and Supplementary file S10), relative to NL, in the light treatment group L0, one gene—the *CONSTANS*-related gene (unigene0025406)—was found to be significantly down-regulated, and the *FT* gene was not significantly expressed. Compared with NL, 13 *CONSTANS-LIKE* genes were significantly differentially expressed in L1, among which three genes were up-regulated (unigene002346462, unigene0027129, and unigene0035118) and ten genes were down-regulated (unigene0025406, unigene0027332, unigene0028118, unigene0029386, unigene0030366, unigene0034031, unigene0034048, unigene0035131, unigene0043102, and unigene0050191). Furthermore, two up-regulated *FT* genes (unigene0001751 and unigene0005649) were significantly differentially expressed. One *FT* gene (unigene0025148) was down-regulated, and six *FT* genes (unigene0002836, unigene0006372, unigene0025249, unigene0038103, unigene0042376, and unigene0045680) were up-regulated in L1 compared with L0.

In addition to these well-known *CO* and *FT* genes, other specific genes are closely related to the regulation of plant flowering processes. For example, the protein encoded by *Hd3a*, a rice ortholog of *FT*, moves from the leaf to the shoot apical meristem and induces flowering in rice, suggesting that the Hd3a protein may be the rice florigen [22]. In our analysis, an *HD3a*-like gene (unigene0019970), the expression of which was decreased under light treatment, was detected in L0. The *HD3a*-like gene may be up-regulated during the rice flowering period but down-regulated during the pitaya flowering period.

The *LEAFY* gene is an important element in the transition from the vegetative to the reproductive phase, as *LEAFY* is both necessary and sufficient to initiate the growth of individual flowers. On long days, *Arabidopsis* plants flower soon after germination, in parallel with rapid up-regulation of LEAFY [23]. In the present study, we found one *LEAFY* homolog gene (unigene0024655) to be significantly up-regulated in L1. We also detected two genes encoding the flowering time control protein in L1, where the expression of *FPA* (unigene0045587) was down-regulated and that of *FCA* (unigene0049096) was up-regulated. One flowering-promoting factor 1-like gene (unigene0023129) was up-regulated in L1.

Light is one of the clock entrainment signals that can induce a plant’s circadian rhythm and act as a bridge in the signaling network between environmental stimulation and the internal processes of the plant. According to the KEGG pathway analysis, some DEGs were associated with the circadian rhythm in pitaya plants. One gene encoding pseudo response regulator APRR5-like protein (unigene0045107) was down-regulated in L0, but not in NL. Two down-regulated circadian clock associated 1-like genes (unigene0036436 and unigene0049229), two down-regulated genes encoding EARLY FLOWERING 4-like protein (unigene0022870 and unigene0039817), and an up-regulated gene encoding EARLY FLOWERING 1-like protein (unigene0043786) were expressed in L1 compared with NL.

### Transcription factors control the course of flowering

Cycling DoF factor (CDF) proteins belong to the larger family of plant-specific DoF (DNA binding with one finger) TFs, which presumably include a single C2-C2 zinc finger and a highly conserved DNA-binding domain [24]. Several *Arabidopsis* DoF transcription factors—CDF1, 2, 3, and 5—are implicated in circadian rhythms and photoperiodism, and their expression at elevated levels is sufficient to repress flowering [25]. In this study (Fig. 5c and Supplementary file S11), we found two CDF genes (unigene0033003 and unigene0031426) that were down-regulated in L0 compared with NL. Similarly, three CDF genes (unigene0031426, unigene0026114, and unigene0033003) were down-regulated and two genes (unigene0029432 and unigene0006016) were up-regulated in L1.

During the development and evolution of flowering plants, the MADS domain transcription factor plays an important role, because it determines the establishment of flower morphology and flower development. The MADS-box gene contains a conserved MADS-box motif and is generally classified as type I or type II with a conserved K-, I-, and C-domain subfamily. In general, the type II subfamily can be divided into ABCDE models during the development of the flower [26]. Compared with NL, no significantly differentially expressed MADS-box genes were detected in L0. In contrast, one gene encoding the AGAMOUS-like MADS-box protein AGL19 (unigene0027094) was down-regulated and the other six genes encoding AGAMOUS-like MADS-box proteins (unigene0018425, unigene0021927, unigene0025394, unigene0037726, unigene0047376, and unigene0202108) were up-regulated in L1.

Other TFs that can bind to the *CO* promoter include the TEOSINTE BRANCHED 1/CYCLOIDEA/PROLIFERATING CELL NUCLEAR ANTIGEN FACTOR (TCP) transcription factor family [27]. Members of this family control multiple traits in a diverse range of plant species, including flower and petal asymmetry [28]. We discovered no DEG-related TCP TFs in L0, whereas all six genes encoding TCP (unigene0005880, unigene0009351, unigene0025734, unigene0028812, unigene0030793, and unigene0033768) were up-regulated in L1 compared with NL.

### Validation of the selected DEGs by quantitative RT-PCR analysis

We considered the control of floral development genes or transcription factors that regulate downstream genes to be more important than other aspects of plant flowering processes. One cycling DoF factor (*CDF*) transcription factor gene in each of the L0 and L1 samples was verified via quantitative RT-PCR (qRT-PCR). One gene encoding response regulator-like APRR5 related to the circadian rhythm in L0 and two genes encoding the circadian clock associated protein in L1 were selected to check the mRNA levels by qRT-PCR. One and two *CONSTANS-LIKE* (*CO*) genes in L0 and L1, respectively, were chosen for real-time quantitative PCR. Furthermore, one *flowering locus T* (*FT*) gene and *HEADING DATE 3A-like* (*HD3a-like*) ortholog involved in L1 and L0 respectively, were verified by qRT-PCR. In addition, two *TCP* TF genes and a gene encoding phytochrome B in L1 were also selected for qRT-PCR (Table 3). The *UBQ* gene was used as a reference gene [29].

**Table 3:** Selected genes used for qRT-PCR

Gene ID	Function	Forward primer (5′ → 3′)	Reverse primer (5′ → 3′)
NL vs L0			
unigene0031426	Cycling DoF factor 1	GAGCAAGGAGAATGGGGATGA	GCAAAATGGCGGAACAGGAA
unigene0025406	Zinc finger protein CONSTANS-LIKE 2	TTTTGTTCGGGATGGAGTCTGA	TTGGGTGGGAATTTGAATAGG
unigene0019970	HEADING DATE 3A-like	GGCAACCCAACTGAGCGAGAA	GCCATCCCATTTGCTGACGA
unigene0045107	Response regulator-like APRR5	AAGACCGCACCCACTTTTCC	CATTCCCACTATCAGACCCACC
NL vs L1			
unigene0026114	Cycling DoF factor 3-like	GGTCGTCGCAAGAGTAAACA	CTATAAAGCGGGCTATCAGG
unigene0027129	CONSTANS-LIKE 15	TGGGAAGAAGCACCACCTCA	AGCGGGCACCACGGAATAA
unigene0028118	CONSTANS-LIKE 4	GCTTTTCGGCGACGACTATTA	TGAAGTCCAGTTCCAGGTAGGG
unigene0036436	Circadian clock associated 1	CCTCAAGATCCCCTAATCACGC	CCAAAAGGAAGCCGCAAAAC
unigene0049229	Circadian clock associated 1	CCAAGCATGGCAGCGATAG	GCTGGTTGAGTTTGGGTAAGAT
unigene0005649	Flowering locus T	AGTGGAGATCGGAGGTGATG	GCCTTGGATTCTCGTAGCA
unigene0002396	Phytochrome B	GGTCTATTCCTTTCCCACTTCG	AGAGAGCTGCTCCATCGCACT
unigene0009351	Transcription factor TCP4	CACTGGAGGAGGTGGATTTATG	GGATTGAGGGTGCGTTGC
unigene0005880	Transcription factor TCP15	GGCGGTGGTGGATTTGAC	GCCCGACCTGTTTACCTGA

*NL* control group (no light, no flowering), *L0* stage of no flowering under light treatment, *L1* flower bud stage under light treatment

**Fig. 6:**
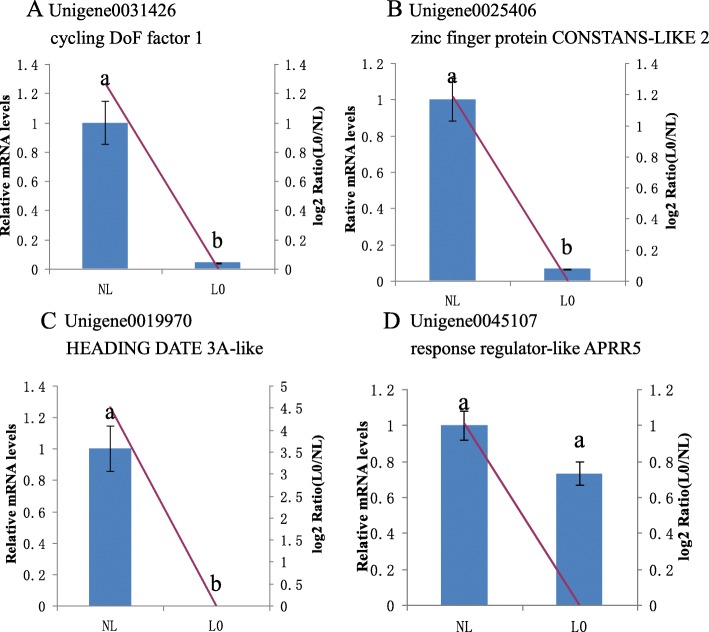
Relative expression of flower induction genes. For the NL and L0 samples, the relative expression of genes and transcription factors involved in the flowering process was determined via quantitative RT-PCR. Expression was normalized to that of *UBQ*. The transcript levels from the NL sample were set as 1. The blue columns represent the expression levels determined by qRT-PCR (left axis), whereas the lines represent the gene expression levels determined by RNA-Seq (right axis). The transcript abundances of the genes encoding **a** cycling DoF factor 1 protein, **b** zinc finger protein CONSTANS-LIKE 2, **c** HEADING DATE 3A-like, and **d** response regulator-like APRR5 were determined and compared across the time course of light-induced treatment. Data represent the mean ± SD for three replicates (*n* = 3). Different lowercase letters above the columns indicate a significant difference at *p* ≤ 0.05 between the columns according to *t* tests performed using the SPSS statistical software. Data in columns with the same letters showed no significant difference (*p* > 0.05)

**Fig. 7:**
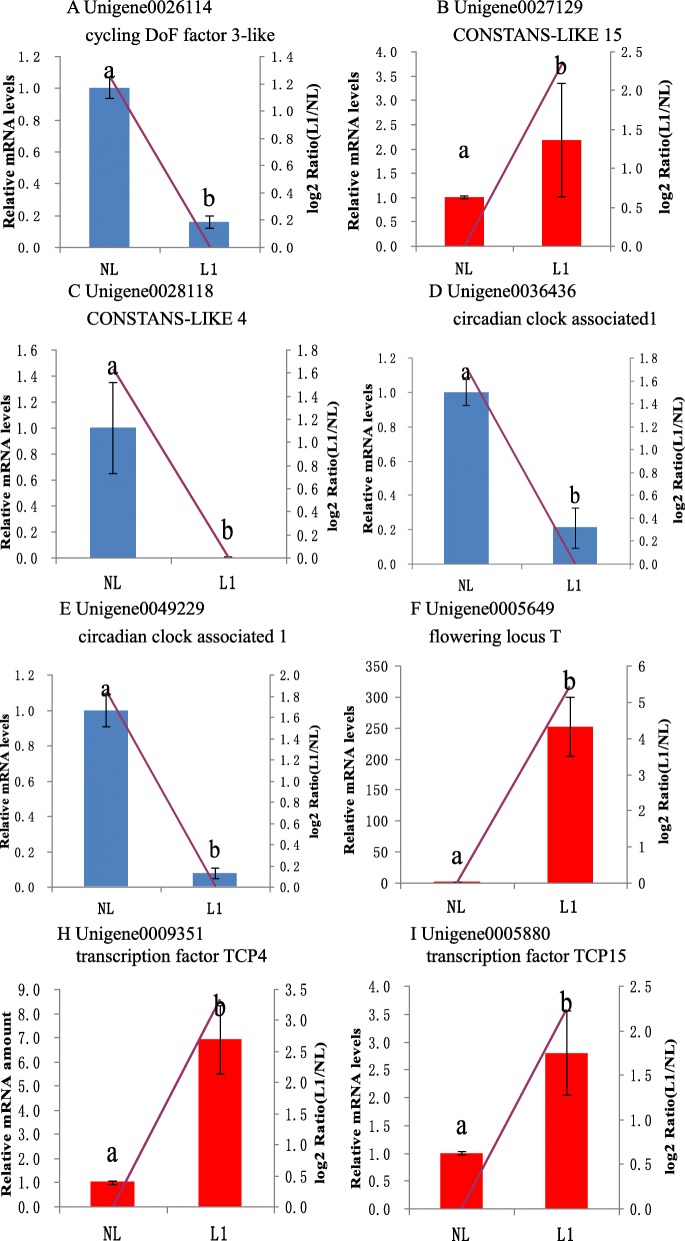
Relative expression of floral development genes and transcription factors. For the NL and L1 samples, the relative expression of genes and transcription factors involved in the flowering process was determined via quantitative RT-PCR. Expression was normalized to that of *UBQ*. The transcript levels from the NL sample were set as 1. The blue or red columns represent the expression levels determined by qRT-PCR (left axis), whereas the lines represent the gene expression levels determined by RNA-Seq (right axis). The transcript abundances of the genes encoding **a** cycling DoF factor 3-like protein, **b** CONSTANS-LIKE 15, (**c**) CONSTANS-LIKE 4, **d**, **e** circadian clock associated 1, **f** flowering locus T, **g** transcription factor TCP4, and **h** transcription factor TCP15 were determined and compared across the time course of light-induced treatment. Data represent the mean ± SD for three replicates (*n* = 3). Different lowercase letters above the columns indicate a significant difference at *p* ≤ 0.05 between the columns according to *t* tests performed using the SPSS statistical software. Data in columns with the same letters showed no significant difference (*p* > 0.05)

The qRT-PCR results from the L0 and L1 stages are shown in Figs. 6 and 7. The expression profiles of all 13 detected genes revealed a similar trend and consistent results between qRT-PCR and RNA-Seq. Three DEGs of L0 exhibited significant down-regulation in plants under light treatment compared with NL, including unigene0031426 (cycling DoF factor 1), unigene0025406 (zinc finger protein CONSTANS-LIKE 2), and unigene0019970 (HEADING DATE 3A-like). However, unigene0045107 (response regulator-like APRR5) associated with the circadian clock, displayed no remarkable decrease in its mRNA levels.

In the L1 stage, all nine DEGs exhibited significant differential expression based on the qRT-PCR results. Two genes exhibiting functional homology to *circadian clock associated 1* in the L1 stage showed a significant reduction in transcript levels under light treatment. A similar situation was found for two genes encoding TCP TF, for which the mRNA abundance was substantially enhanced in the L1 sample following light treatment. Interestingly, two *CO* genes revealed an opposite trend in expression in the L1 stage: one was up-regulated and the other was down-regulated. This finding indicates that different *CO* gene families may be subjected to different types of regulation, resulting in opposite types of expression. In addition, *CDF3-like* was down-regulated, whereas *FT* displayed a higher mRNA level in L1 during light treatment than in NL, as determined by qRT-PCR.

### Tissue-specific expression of key genes

**Fig. 8:**
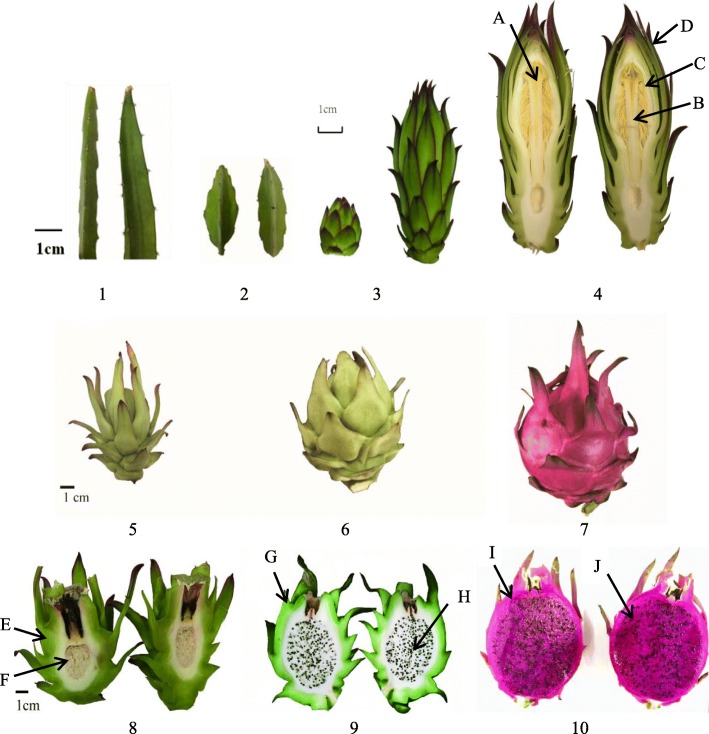
Various tissues of pitaya plants at different time points. 1: Old stem; 2: young stem; 3: small flower bud; 4: large flower bud, A: pistil, B: stamen, C: style, D: flower scales; 5: small green fruit; 6: big green fruit; 7: red pitaya; E: small green peel, F: small green flesh; 9: big green fruit, G: big green peel, H: big green flesh; 10: red fruit, I: red peel, J: red flesh

**Fig. 9:**
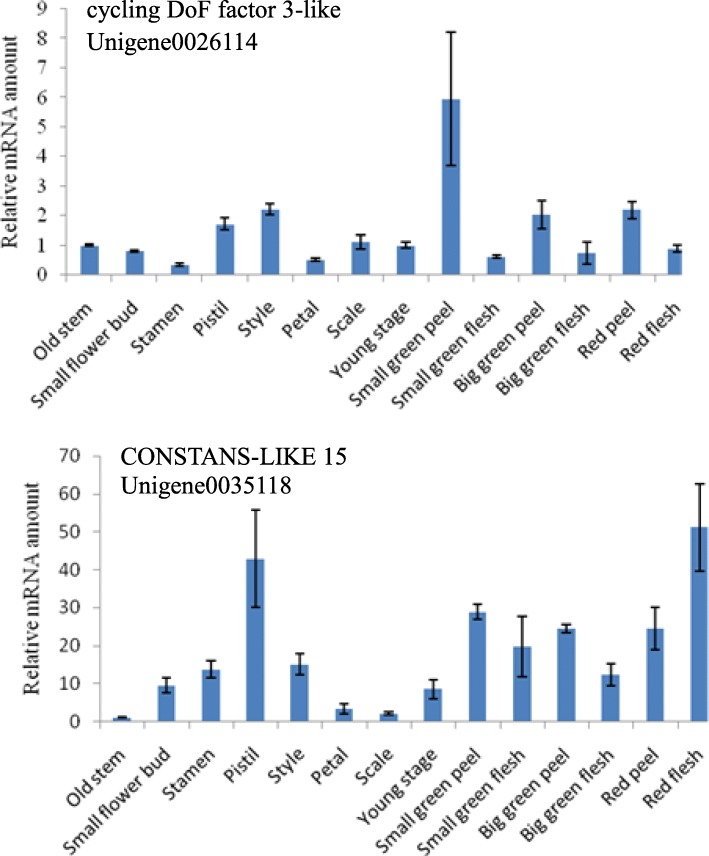
Tissue-specific expression of *CDF-3 like* (unigene0026114) and *CO-15 like* (unigene0035118). Expression was normalized to that of *UBQ*. The transcript levels from the old stem were set as 1

To further explore whether the genes associated with the flowering process of pitaya plants are tissue-specific, various growth stages of the stem, flower, and fruit as depicted in Fig. 8 were selected for analysis. We selected *CDF-3 like* (Unigene0026114) and *CO-15 like* (Unigene0035118) for the tissue-specific analysis, and the results are presented in Fig. 9. The results revealed that *CDF-3 like* displayed specific expression levels in different tissues at different time points, where its expression was increased in the pistils and styles and highest in the small green peel, compared with old stems. The expression level of *CO-15 like* was higher in the flowers and fruits than in old stems, although the specificity was not obvious.

## Discussion

Some genes involved in light-induced flowering have been extensively studied in other plants, especially the model plant *Arabidopsis*. However, no previous research in this area has been conducted for pitaya plants. Thus, it is necessary to examine the relationship between flowering and the key genes regulating this process.

Hormone regulation, signaling, and homeostasis are very important factors in the flowering process. Hormonal signaling, involving hormones such as ethylene, abscisic acid, Gibberellin and auxins, plays an important role in regulating flowering time through the regulation of *FLC*, *CO*, GAI and *FT.* Strict control of auxin homeostasis and the maintenance of an appropriate level of IAA are essential for normal growth and development. In the present study, some genes (unigene0036729 and unigene0037786) encoding auxin transporter-like protein were significantly up-regulated following light treatment. Initiation of floral primordia requires not only a local maximum level of auxins but also the activity of AUXIN RESPONSE FACTOR [30]. In our analysis, several genes (unigene0004150, unigene0021847, and unigene0032942, and others) encoding the auxin-responsive protein also exhibited up-regulation. In rice, defects along the JA signaling pathway affect not only floret opening and anther dehiscence but also spikelet development [31]. In the present study, we observed that some genes encoding the JAZ-like protein (e.g., MYC2 TFs) were involved in the JA signaling pathway. Gibberellic acid also play important role in pitaya flower induction [32], we checked three DELLA protein GAI genes (Unigene0005738, Unigene0031663, Unigene0046182) were significantly up-regulated during the flower induction and development process.

Light signals detected by the phytochrome A (phyA) and cryptochrome (cry) photoreceptors stabilize the CO protein on long days [33]. We observed that some *CO-like* genes were up-regulated, whereas others were down-regulated, under the same conditions, indicating that some *CO* genes belonging to specific families may be repressed by certain proteins or miRNAs in pitaya plants. The CONSTANS like genes were family genes with similarity sequences and conserved domains. However, they may display differently expressed patterns and play different functions even in the same pitaya samples. Photoreceptors regulate CO stability and act antagonistically to generate daily rhythms in CO abundance [34]. Moreover, a *phyB* mutation has been shown to induce an increase in CO protein abundance during the early phase of the photoperiod [34], indicating that *phyB* mutations also accelerate flowering. Thus, we can conclude that the amount of *CO* is positively regulated by the photoreceptors cry1, cry2, and phyA, whereas the mRNA expression of *CO* is inhibited by phyB. A similar conclusion was drawn from the present study. The gene encoding phyB (unigene0002396) was down-regulated, as detected by qPCR, and the expression pattern was found to be the same as that from transcriptome sequencing, suggesting that PHYB may negatively regulate *CO* throughout the pitaya flowering process.

The circadian clock gene, *CO*, which is a key transcriptional activator of the photoperiod pathway, is essential for proper photoperiodic flowering. In the current model of the *Arabidopsis* circadian clock, most components function as repressors [35]. Fujiwara et al. [36] demonstrated that the circadian clock proteins LATE ELONGATED HYPOCOTYL (LHY) and CIRCADIAN CLOCK-ASSOCIATED 1 (CCA1) repress floral transition, and in particular repress *FT* expression under long-day conditions. In our analysis, the transcriptome levels of most DEGs associated with the circadian rhythm were decreased, which is consistent with the findings of previous reports.

The *FT* gene was first identified in *A. thaliana*, in which it plays major roles in the photoperiod pathway and initiating the flowering signal in the apical meristem [37]. Unlike many other floral regulators, the deduced sequence of the FT protein suggests that it does not directly control transcription or transcript processing [38]. The expression of *FT* is affected by numerous factors, some of which act as repressors and others as positive regulators. The former class includes several MADS-box TFs [39], AP2-like proteins, and B-box proteins, whereas the latter class includes CO [40], NUCLEAR FACTOR Y, and GIGANTEA (GI) [41]. These findings are consistent with those of the present study; with the exception of one down-regulated *FT* gene (unigene0025148), all other genes were up-regulated.

CYCLING DoF FACTOR 1 (CDF1) and its homologs play an important role in floral transition by repressing the expression of floral activator genes, such as CO and FT, in *Arabidopsis* [42]. One study indicated that CDF repression operates through the formation of a CDF–TPL transcriptional complex, which binds the CO and FT promoters to reduce their expression levels throughout the morning, for seasonal flowering [43]. The results of our qRT-PCR analysis (Figs. 6 and 7) revealed down-regulated expression of two *CDF-like* genes (unigene0031426 and unigene0026114), but up-regulated expression of *CO-like 15* (unigene0027129) and *FT* (unigene0005649) genes. These findings demonstrate that CDF may repress *CO* and *FT* transcriptome expression during the pitaya flowering process.

The *TCP* gene family encodes TFs that control various aspects of the developmental and growth traits of plants. According to the structure of the conserved region, they can be classified into classes I and II in *Arabidopsis* [44], and orchestrate the transition from the vegetative to reproductive to senescent stages of the life cycle. They may be upstream and activate *SOC1* to modulate flowering [44]. In addition, TCP4 is directly associated with *CO* promoters through TCP binding sites and promotes *CO* expression around dusk, together with other *CO* activators (FLOWERING BHLH)—FBHs [45]. Furthermore, these TCPs not only interact with the flower-repressing CDFs but also physically interact with FBHs to facilitate *CO* transcription [46]. Our results revealed that two transcription factor *TCP4* genes (unigene0009351 and unigene0033768) and other *TCP* genes, such as *TCP15* and *TCP18*, were up-regulated. Moreover, the expression level of *CO*-like genes was increased.

**Fig. 10:**
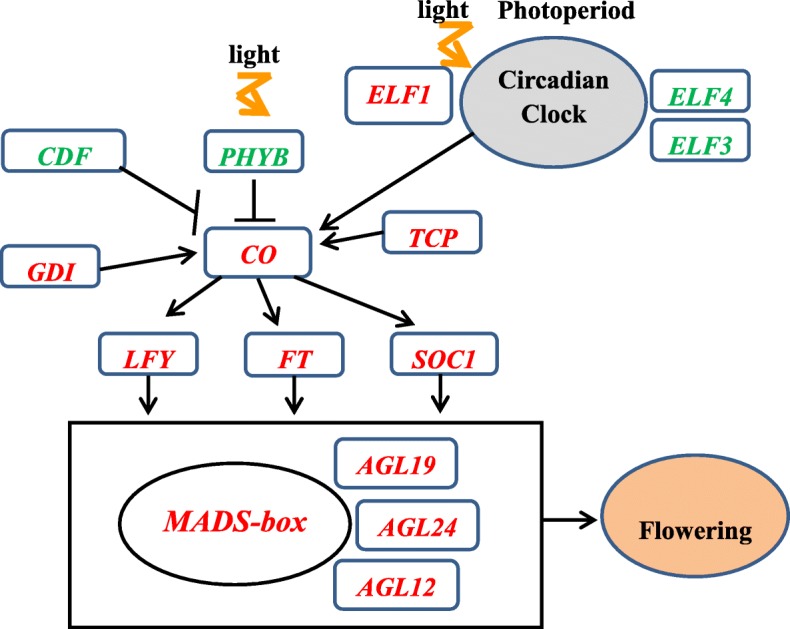
A hypothetical flower induction Pathway in long-day pitaya plants induced by supplementary lighting in short-day winter season. Note: The photoperiod promotes floral transition. Arrows indicate activation and lines ending with a vertical line indicate repression. Genes in red type indicate up-regulation and genes in green type indicate down-regulation

According to the upper discussion and analysis in general, a hypothetical flower induction pathway in long-day pitaya plants induced by supplementary lighting in short-day winter season (Fig. 10) was deduced. The signal starts from Photoperiod sensing protein including PHYB and ELF1, The circadian clock gene *CO* is a key transcriptional activator of the flower induction pathway *in pitaya.* we will do more work on subsequent research to verify the hypothesis.

## Conclusion

In summary, this study has revealed the transcriptomic profiles of pitaya flower induction under supplementary lighting, as evaluated by de novo RNA-Seq and qRT-PCR. At the transcriptomic level, we detected many DEGs in various developmental stages that mediated different functions in the time-course response to supplementary lighting-induced flowering. Such DEGs may interact with each other, and some key genes may regulate the network of floral induction pathways. The obtained findings improve our understanding of the molecular mechanisms involved in flower induction of long-day pitaya plants in short-day winter season, and should support new biotechnological advances to create novel pitaya varieties that can bloom naturally in the winter season through genetic modification or targeted gene mutation.

## Methods

### Plant material and experimental design

The plant material, “Jindu No. 1” red-fleshed pitaya plants, was purchased from the Hainan Enhong Agricultural Technology Company in Dongfang city. One-year-old pitaya plants were selected for the experiment. One group of plants (the light-treated group) were subjected to 5 h of supplementary light every night from 6:30 p.m. to 11:30 p.m. between January 1 and March 31, 2018. The control group of plants were no supplementary light. Besides the different supplementary lighting conditions, all other variables (such as watering frequency and fertilizer use) were the same for both groups.

The light treatment experiment was performed and samples were obtained at four different stages: NL, the control group (no light, no flowering); L0 (no flowering under light treatment); L1 (flower bud stage under light treatment); and L2 (one-week post-bud stage under light treatment). We used 15 W LED mixed light bulbs as the light source. The light spectrum ratio of red light to blue light was 7:1, and the major wavelength was λ = 580 nm. The luminous flux was 1500 Lm, the color temperature was 2900–3300 K and the light intensity was greater than 110 Lx within 1.5 m.

### Sample preparation and sequencing

We performed three biological replicates for each stage, each involving four mixed plant samples. Every sample was selected to be 10 cm in length from the top of the stem, and included 2 cm of tissue around the base of the thorn (Red circles in Fig. 1), The sample is synchronized by our careful visual observation to make sure that we can harvest similar size buds for every sampling stage. These samples were then immediately frozen using liquid nitrogen and stored at − 80 °C before being sent to Gene Denovo Biotechnology Co. (Guangzhou, China) for de novo and RNA-Seq using the Illumina HiSeq 4000 platform.

Raw data were filtered by removing reads that included adapter, poly-*N* > 10%, and low-quality reads to obtain clean reads as a foundation for further analysis. The Q (Phred Quality Score) = − 10 log_10_Perror; Q20 means that the proportion of Phred Quality Score was greater than 20; Q30 means that the proportion of Phred Quality Score was greater than 30; In this study, the Q20 and Q30 of all samples were > 98% and > 94% after filter (Supplementary S1). De novo assembly of the transcriptome was conducted using the short-read assembly program, Trinity [47]. The rRNA-free, high-quality clean reads were mapped to the reference transcriptome using the short-read alignment tool Bowtie2 [48], employing its default parameters. The RPKM parameter was used to determine the expression level of each gene, which was calculated as the fold change in expression in different samples. In general, a flowchart of the RNAseq process was showed on Supplemental file S12.

At present, the pitaya genome is not sequenced yet. Interpretation of proper orthologs is difficult at this moment. Hence the gene annotation was generated only by using the Genbank data as reference.

### Identification of DEGs

Genes with a false discovery rate (FDR) ≤ 0.05 and an absolute value of log_2_ ratio ≥ 1 in a given comparison were considered significantly differentially expressed genes (DEGs) that could be subjected to enrichment analysis of GO functions and KEGG pathways. All DEGs were mapped to GO terms in the Gene Ontology database (http://www.geneontology.org/), and gene numbers were calculated for every term. Significantly enriched GO terms were defined by a corrected *p* value ≤0.05. Pathway enrichment analysis, which can be calculated using the same formula as that in GO analysis, identified significantly enriched metabolic pathways or signal transduction pathways among the DEGs. Our raw data and processed data have been uploaded to the NCBI Gene Expression Omnibus (GSE125083).

### Quantitative RT-PCR

Total RNA was separately extracted from the four groups using the cetyltrimethylammonium bromide method. The resulting RNA (1 μg) was treated with gDNA Eraser to remove contaminating genomic DNA and synthesized to the first-strand complementary DNA using a PrimeScript RT reagent kit with gDNA Eraser (TaKaRa), according to the manufacturer’s instructions. Primer pairs for qRT-PCR were designed using Primer Premier 5.0 (PREMIER Biosoft International, Palo Alto, CA, USA). The cDNA reverse-transcription products were used as templates for qRT-PCR. An Applied Biosystems 7500 Real-Time PCR System (Life Technologies, USA) was used for qRT-PCR, and the reaction consisted of 10 μL of 2× SYBR Premix Ex Taq II, 0.4 μL of 50× ROX Reference Dye or Dye II, 2 μL of cDNA, 6 μL of sterilized purified water, 0.8 μL of forward primer (10 μM), and 0.8 μL of reverse primer (10 μM) in a total volume of 20 μL. No-template controls were also prepared for each primer pair. The relative quantification method (Delta-Delta cycle threshold) was used to evaluate quantitative variation between the replicates examined. We selected 13 DEGs for qRT-PCR (Table 3).

## Supplementary information

**Additional file 1: Supplemental Figure S1.** Clean reads of all samples.

**Additional file 2: Supplemental S2.** Length distribution of all unigenes.

**Additional file 3: Supplemental S3.** NL-VS-L0 GO Enrichment (Molecular Function).

**Additional file 4: Supplemental S4.** NL-VS-L0 GO Enrichment (Cellular Component).

**Additional file 5: Supplemental S5.** NL-VS-L0 GO Enrichment (Biological Process).

**Additional file 6: Supplemental S6.** NL-VS-L1 GO Enrichment (Molecular Function).

**Additional file 7: Supplemental S7.** NL-VS-L1 GO Enrichment (Cellular Component).

**Additional file 8: Supplemental S8.** NL-VS-L1 GO Enrichment (Biological Process).

**Additional file 9: Supplemental S9.** The DEGs related to plant hormone.

**Additional file 10: Supplemental S10.** The DEGs of *CO* and *FT.*

**Additional file 11: Supplemental S11.** Three main TFs involved in pitaya flowering stage.

**Additional file 12: Supplemental S12.** A flowchart of the RNAseq process.

## Data Availability

The raw data and processed data from this study have been uploaded to the NCBI Gene Expression Omnibus (GSE125083).

## Abbreviations

API: APETALA1
BR: Brassinosteroid
CAM: Crassulacean acid metabolism
CDF: Cycling DoF factor
CO: CONSTANS
cry: cryptochrome
DEG: Differentially expressed gene
FBH: FLOWERING BHLH
FD: FLOWERING LOCUS D
FDR: False discovery rate
FT: FLOWERING LOCUS T
GA: Gibberellin
GC: Guanine-cytosine
GO: Gene ontology
IAA: Indole-3-acetic acid
JA: Jasmonic acid
JAZ: Jasmonate-ZIM domain (proteins)
LD: Long day
LFY: LEAFY
phyA: phytochrome A
PRR: Pseudo response regulator
RPKM: Reads per kilobase per million reads
SOC1: SUPPRESSOR OF OVEREXPRESSION OF CONSTANS1
TCP: TEOSINTE BRANCHED 1/CYCLOIDEA/PROLIFERATING CELL NUCLEAR ANTIGEN FACTOR
TF: Transcription factor
