# Supplementary figures and images for: Transcriptomic analysis of flower induction for long-day pitaya by supplementary lighting in short-day winter season
Source: BMC Genomics. 2020 Apr 29;21:329. doi: 10.1186/s12864-020-6726-6 (PMC7191803; doi:10.1186/s12864-020-6726-6)

**Supplemental fig. 1 Clean reads of all samples**


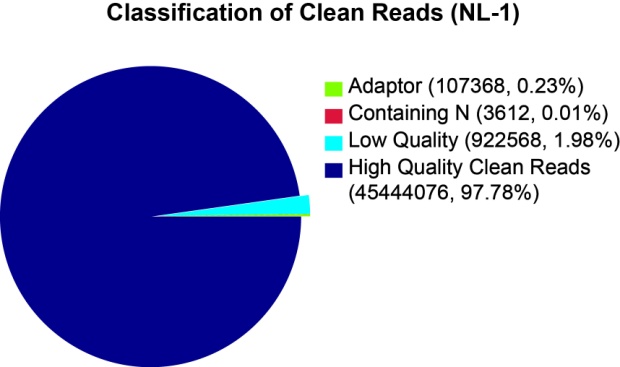

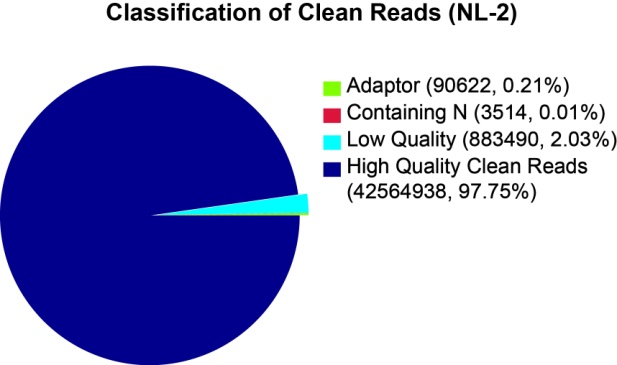

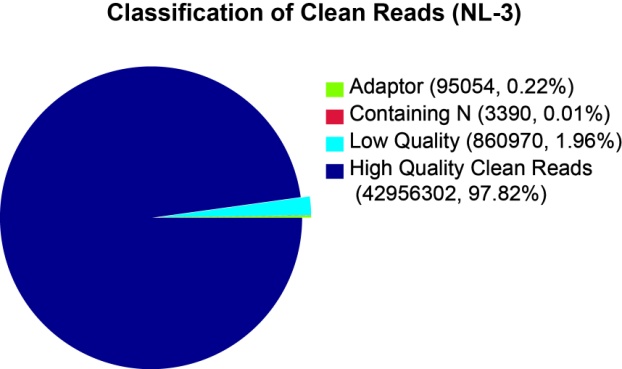

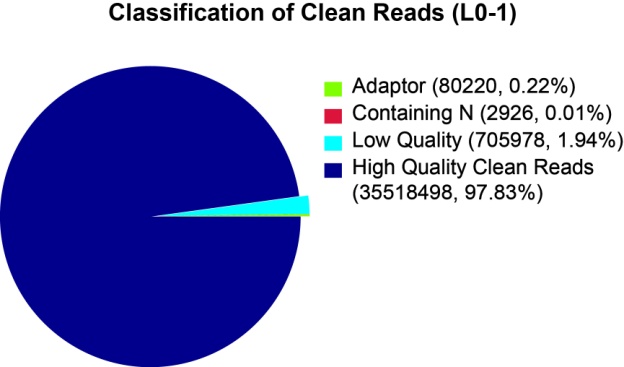

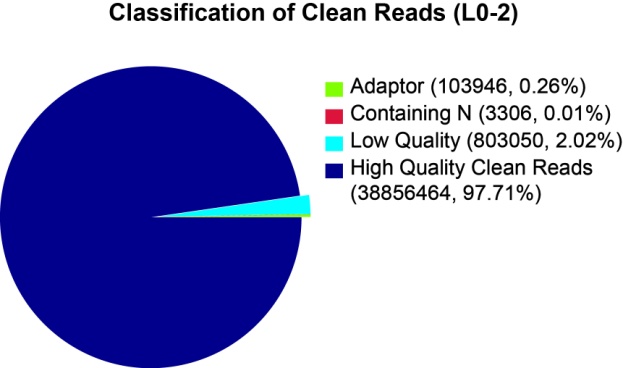

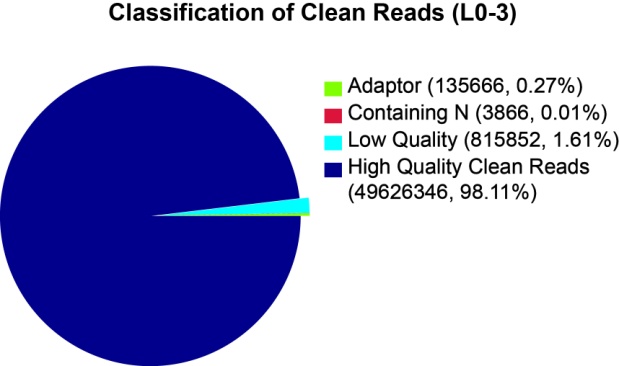

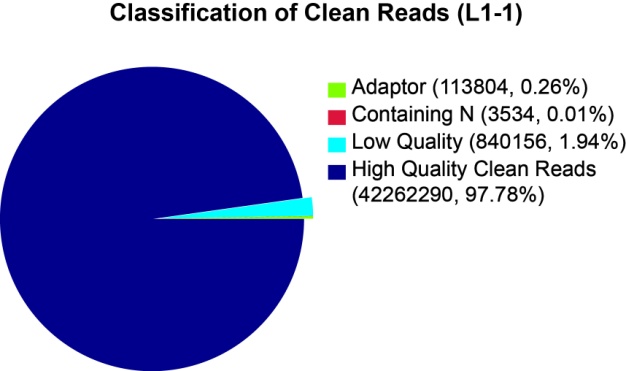

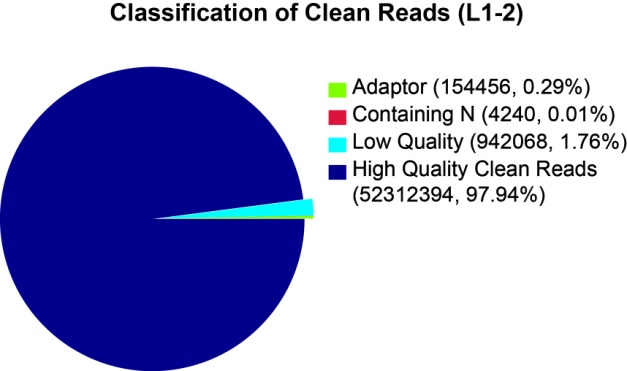

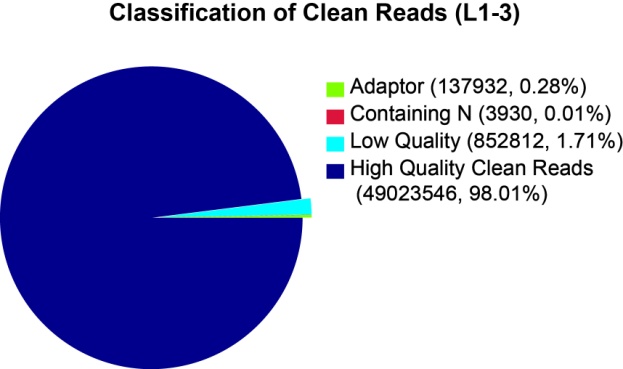

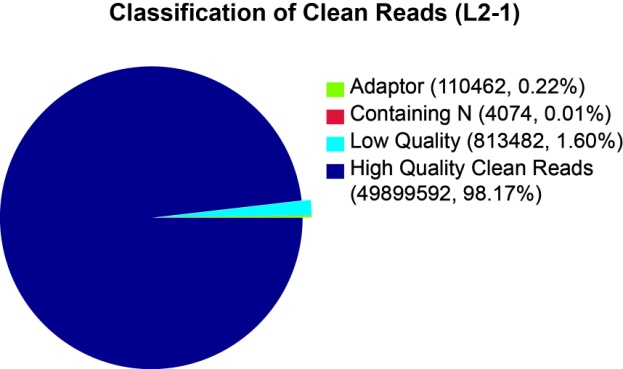

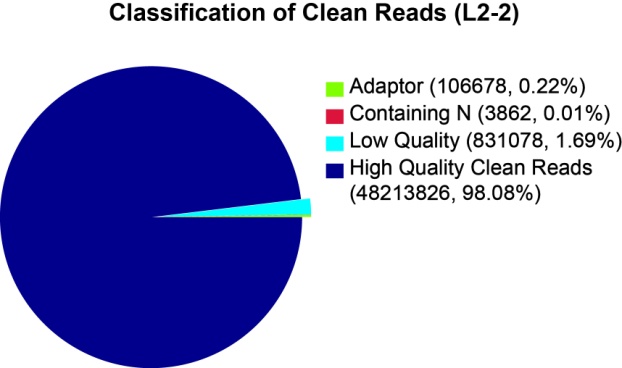

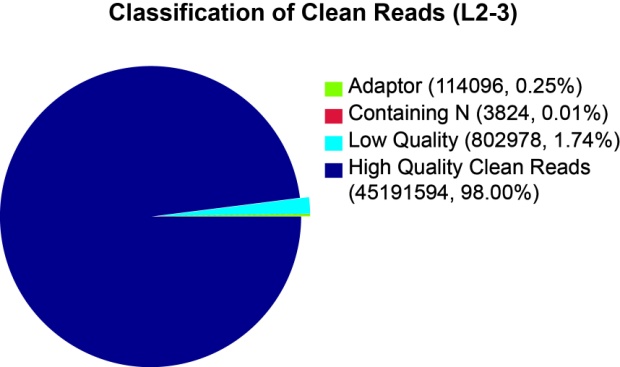

Supplement: Supplementary file 1 — Additional file 1: Supplemental Figure S1. Clean reads of all samples. [file 12864_2020_6726_MOESM1_ESM.docx]

Supplemental S2 Length distribution of all unigenes


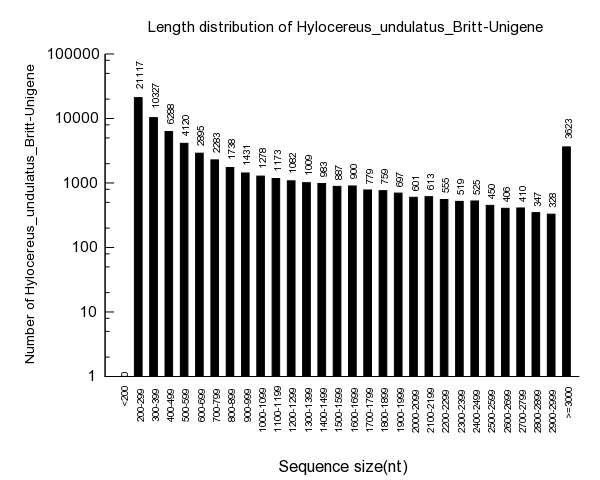

Supplement: Supplementary file 2 — Additional file 2: Supplemental S2. Length distribution of all unigenes. [file 12864_2020_6726_MOESM2_ESM.docx]
