# Supplementary material for: Transcriptomic analysis of flower induction for long-day pitaya by supplementary lighting in short-day winter season
Source: BMC Genomics. 2020 Apr 29;21:329. doi: 10.1186/s12864-020-6726-6 (PMC7191803; doi:10.1186/s12864-020-6726-6)
Supplement: Supplementary file 3 — Additional file 3: Supplemental S3. NL-VS-L0 GO Enrichment (Molecular Function). [file 12864_2020_6726_MOESM3_ESM.docx]

Supplemental S3 NL-VS-L0 GO Enrichment (Molecular Function)

| **#** | **GO ID** | **Description** | **GeneRatio (14)** | **BgRatio (3432)** | **pvalue** | **p.adjust** |
| --- | --- | --- | --- | --- | --- | --- |
| 1 | [GO:0016717](file:///E:\2018-7-3%E7%81%AB%E9%BE%99%E6%9E%9C%E8%BD%AC%E5%BD%95%E7%BB%84%E6%B5%8B%E5%BA%8F\%E5%AE%8C%E6%95%B4%E7%89%88%E6%95%B0%E6%8D%AE\GDR3855-Hylocereus_undulatus_Britt-12-RNAseq_result\4_Function\2_Group_Diff_Function\UP_DOWN\GO\NL-VS-L0.F.html#gene1) | oxidoreductase activity, acting on paired donors, with oxidation of a pair of donors resulting in the reduction of molecular oxygen to two molecules of water | 1 (7.14%) | 3 (0.09%) | 0.012191 | 0.220963 |
| 2 | [GO:0004702](file:///E:\2018-7-3%E7%81%AB%E9%BE%99%E6%9E%9C%E8%BD%AC%E5%BD%95%E7%BB%84%E6%B5%8B%E5%BA%8F\%E5%AE%8C%E6%95%B4%E7%89%88%E6%95%B0%E6%8D%AE\GDR3855-Hylocereus_undulatus_Britt-12-RNAseq_result\4_Function\2_Group_Diff_Function\UP_DOWN\GO\NL-VS-L0.F.html#gene2) | receptor signaling protein serine/threonine kinase activity | 2 (14.29%) | 44 (1.28%) | 0.013258 | 0.220963 |
| 3 | [GO:0005057](file:///E:\2018-7-3%E7%81%AB%E9%BE%99%E6%9E%9C%E8%BD%AC%E5%BD%95%E7%BB%84%E6%B5%8B%E5%BA%8F\%E5%AE%8C%E6%95%B4%E7%89%88%E6%95%B0%E6%8D%AE\GDR3855-Hylocereus_undulatus_Britt-12-RNAseq_result\4_Function\2_Group_Diff_Function\UP_DOWN\GO\NL-VS-L0.F.html#gene3) | receptor signaling protein activity | 2 (14.29%) | 44 (1.28%) | 0.013258 | 0.220963 |
| 4 | [GO:0004871](file:///E:\2018-7-3%E7%81%AB%E9%BE%99%E6%9E%9C%E8%BD%AC%E5%BD%95%E7%BB%84%E6%B5%8B%E5%BA%8F\%E5%AE%8C%E6%95%B4%E7%89%88%E6%95%B0%E6%8D%AE\GDR3855-Hylocereus_undulatus_Britt-12-RNAseq_result\4_Function\2_Group_Diff_Function\UP_DOWN\GO\NL-VS-L0.F.html#gene4) | signal transducer activity | 2 (14.29%) | 55 (1.6%) | 0.020286 | 0.253573 |
| 5 | [GO:0035250](file:///E:\2018-7-3%E7%81%AB%E9%BE%99%E6%9E%9C%E8%BD%AC%E5%BD%95%E7%BB%84%E6%B5%8B%E5%BA%8F\%E5%AE%8C%E6%95%B4%E7%89%88%E6%95%B0%E6%8D%AE\GDR3855-Hylocereus_undulatus_Britt-12-RNAseq_result\4_Function\2_Group_Diff_Function\UP_DOWN\GO\NL-VS-L0.F.html#gene5) | UDP-galactosyltransferase activity | 1 (7.14%) | 12 (0.35%) | 0.047943 | 0.348095 |
| 6 | [GO:0008378](file:///E:\2018-7-3%E7%81%AB%E9%BE%99%E6%9E%9C%E8%BD%AC%E5%BD%95%E7%BB%84%E6%B5%8B%E5%BA%8F\%E5%AE%8C%E6%95%B4%E7%89%88%E6%95%B0%E6%8D%AE\GDR3855-Hylocereus_undulatus_Britt-12-RNAseq_result\4_Function\2_Group_Diff_Function\UP_DOWN\GO\NL-VS-L0.F.html#gene6) | galactosyltransferase activity | 1 (7.14%) | 15 (0.44%) | 0.059590 | 0.348095 |
| 7 | [GO:0017111](file:///E:\2018-7-3%E7%81%AB%E9%BE%99%E6%9E%9C%E8%BD%AC%E5%BD%95%E7%BB%84%E6%B5%8B%E5%BA%8F\%E5%AE%8C%E6%95%B4%E7%89%88%E6%95%B0%E6%8D%AE\GDR3855-Hylocereus_undulatus_Britt-12-RNAseq_result\4_Function\2_Group_Diff_Function\UP_DOWN\GO\NL-VS-L0.F.html#gene7) | nucleoside-triphosphatase activity | 3 (21.43%) | 241 (7.02%) | 0.069946 | 0.348095 |
| 8 | [GO:0004674](file:///E:\2018-7-3%E7%81%AB%E9%BE%99%E6%9E%9C%E8%BD%AC%E5%BD%95%E7%BB%84%E6%B5%8B%E5%BA%8F\%E5%AE%8C%E6%95%B4%E7%89%88%E6%95%B0%E6%8D%AE\GDR3855-Hylocereus_undulatus_Britt-12-RNAseq_result\4_Function\2_Group_Diff_Function\UP_DOWN\GO\NL-VS-L0.F.html#gene8) | protein serine/threonine kinase activity | 2 (14.29%) | 121 (3.53%) | 0.085111 | 0.348095 |
| 9 | [GO:0003774](file:///E:\2018-7-3%E7%81%AB%E9%BE%99%E6%9E%9C%E8%BD%AC%E5%BD%95%E7%BB%84%E6%B5%8B%E5%BA%8F\%E5%AE%8C%E6%95%B4%E7%89%88%E6%95%B0%E6%8D%AE\GDR3855-Hylocereus_undulatus_Britt-12-RNAseq_result\4_Function\2_Group_Diff_Function\UP_DOWN\GO\NL-VS-L0.F.html#gene9) | motor activity | 1 (7.14%) | 22 (0.64%) | 0.086255 | 0.348095 |
| 10 | [GO:0016462](file:///E:\2018-7-3%E7%81%AB%E9%BE%99%E6%9E%9C%E8%BD%AC%E5%BD%95%E7%BB%84%E6%B5%8B%E5%BA%8F\%E5%AE%8C%E6%95%B4%E7%89%88%E6%95%B0%E6%8D%AE\GDR3855-Hylocereus_undulatus_Britt-12-RNAseq_result\4_Function\2_Group_Diff_Function\UP_DOWN\GO\NL-VS-L0.F.html#gene10) | pyrophosphatase activity | 3 (21.43%) | 268 (7.81%) | 0.090171 | 0.348095 |
| 11 | [GO:0016818](file:///E:\2018-7-3%E7%81%AB%E9%BE%99%E6%9E%9C%E8%BD%AC%E5%BD%95%E7%BB%84%E6%B5%8B%E5%BA%8F\%E5%AE%8C%E6%95%B4%E7%89%88%E6%95%B0%E6%8D%AE\GDR3855-Hylocereus_undulatus_Britt-12-RNAseq_result\4_Function\2_Group_Diff_Function\UP_DOWN\GO\NL-VS-L0.F.html#gene11) | hydrolase activity, acting on acid anhydrides, in phosphorus-containing anhydrides | 3 (21.43%) | 268 (7.81%) | 0.090171 | 0.348095 |
| 12 | [GO:0016817](file:///E:\2018-7-3%E7%81%AB%E9%BE%99%E6%9E%9C%E8%BD%AC%E5%BD%95%E7%BB%84%E6%B5%8B%E5%BA%8F\%E5%AE%8C%E6%95%B4%E7%89%88%E6%95%B0%E6%8D%AE\GDR3855-Hylocereus_undulatus_Britt-12-RNAseq_result\4_Function\2_Group_Diff_Function\UP_DOWN\GO\NL-VS-L0.F.html#gene12) | hydrolase activity, acting on acid anhydrides | 3 (21.43%) | 269 (7.84%) | 0.090966 | 0.348095 |
| 13 | [GO:0015631](file:///E:\2018-7-3%E7%81%AB%E9%BE%99%E6%9E%9C%E8%BD%AC%E5%BD%95%E7%BB%84%E6%B5%8B%E5%BA%8F\%E5%AE%8C%E6%95%B4%E7%89%88%E6%95%B0%E6%8D%AE\GDR3855-Hylocereus_undulatus_Britt-12-RNAseq_result\4_Function\2_Group_Diff_Function\UP_DOWN\GO\NL-VS-L0.F.html#gene13) | tubulin binding | 1 (7.14%) | 25 (0.73%) | 0.097467 | 0.348095 |
| 14 | [GO:0016831](file:///E:\2018-7-3%E7%81%AB%E9%BE%99%E6%9E%9C%E8%BD%AC%E5%BD%95%E7%BB%84%E6%B5%8B%E5%BA%8F\%E5%AE%8C%E6%95%B4%E7%89%88%E6%95%B0%E6%8D%AE\GDR3855-Hylocereus_undulatus_Britt-12-RNAseq_result\4_Function\2_Group_Diff_Function\UP_DOWN\GO\NL-VS-L0.F.html#gene14) | carboxy-lyase activity | 1 (7.14%) | 25 (0.73%) | 0.097467 | 0.348095 |
| 15 | [GO:0008135](file:///E:\2018-7-3%E7%81%AB%E9%BE%99%E6%9E%9C%E8%BD%AC%E5%BD%95%E7%BB%84%E6%B5%8B%E5%BA%8F\%E5%AE%8C%E6%95%B4%E7%89%88%E6%95%B0%E6%8D%AE\GDR3855-Hylocereus_undulatus_Britt-12-RNAseq_result\4_Function\2_Group_Diff_Function\UP_DOWN\GO\NL-VS-L0.F.html#gene15) | translation factor activity, RNA binding | 1 (7.14%) | 27 (0.79%) | 0.104870 | 0.349566 |
| 16 | [GO:0008092](file:///E:\2018-7-3%E7%81%AB%E9%BE%99%E6%9E%9C%E8%BD%AC%E5%BD%95%E7%BB%84%E6%B5%8B%E5%BA%8F\%E5%AE%8C%E6%95%B4%E7%89%88%E6%95%B0%E6%8D%AE\GDR3855-Hylocereus_undulatus_Britt-12-RNAseq_result\4_Function\2_Group_Diff_Function\UP_DOWN\GO\NL-VS-L0.F.html#gene16) | cytoskeletal protein binding | 1 (7.14%) | 39 (1.14%) | 0.148118 | 0.402397 |
| 17 | [GO:0001071](file:///E:\2018-7-3%E7%81%AB%E9%BE%99%E6%9E%9C%E8%BD%AC%E5%BD%95%E7%BB%84%E6%B5%8B%E5%BA%8F\%E5%AE%8C%E6%95%B4%E7%89%88%E6%95%B0%E6%8D%AE\GDR3855-Hylocereus_undulatus_Britt-12-RNAseq_result\4_Function\2_Group_Diff_Function\UP_DOWN\GO\NL-VS-L0.F.html#gene17) | nucleic acid binding transcription factor activity | 1 (7.14%) | 40 (1.17%) | 0.151633 | 0.402397 |
| 18 | [GO:0016787](file:///E:\2018-7-3%E7%81%AB%E9%BE%99%E6%9E%9C%E8%BD%AC%E5%BD%95%E7%BB%84%E6%B5%8B%E5%BA%8F\%E5%AE%8C%E6%95%B4%E7%89%88%E6%95%B0%E6%8D%AE\GDR3855-Hylocereus_undulatus_Britt-12-RNAseq_result\4_Function\2_Group_Diff_Function\UP_DOWN\GO\NL-VS-L0.F.html#gene18) | hydrolase activity | 5 (35.71%) | 727 (21.18%) | 0.156130 | 0.402397 |
| 19 | [GO:0036094](file:///E:\2018-7-3%E7%81%AB%E9%BE%99%E6%9E%9C%E8%BD%AC%E5%BD%95%E7%BB%84%E6%B5%8B%E5%BA%8F\%E5%AE%8C%E6%95%B4%E7%89%88%E6%95%B0%E6%8D%AE\GDR3855-Hylocereus_undulatus_Britt-12-RNAseq_result\4_Function\2_Group_Diff_Function\UP_DOWN\GO\NL-VS-L0.F.html#gene19) | small molecule binding | 5 (35.71%) | 732 (21.33%) | 0.159597 | 0.402397 |
| 20 | [GO:0003677](file:///E:\2018-7-3%E7%81%AB%E9%BE%99%E6%9E%9C%E8%BD%AC%E5%BD%95%E7%BB%84%E6%B5%8B%E5%BA%8F\%E5%AE%8C%E6%95%B4%E7%89%88%E6%95%B0%E6%8D%AE\GDR3855-Hylocereus_undulatus_Britt-12-RNAseq_result\4_Function\2_Group_Diff_Function\UP_DOWN\GO\NL-VS-L0.F.html#gene20) | DNA binding | 1 (7.14%) | 43 (1.25%) | 0.162097 | 0.402397 |
| 21 | [GO:0016830](file:///E:\2018-7-3%E7%81%AB%E9%BE%99%E6%9E%9C%E8%BD%AC%E5%BD%95%E7%BB%84%E6%B5%8B%E5%BA%8F\%E5%AE%8C%E6%95%B4%E7%89%88%E6%95%B0%E6%8D%AE\GDR3855-Hylocereus_undulatus_Britt-12-RNAseq_result\4_Function\2_Group_Diff_Function\UP_DOWN\GO\NL-VS-L0.F.html#gene21) | carbon-carbon lyase activity | 1 (7.14%) | 45 (1.31%) | 0.169007 | 0.402397 |
| 22 | [GO:0008194](file:///E:\2018-7-3%E7%81%AB%E9%BE%99%E6%9E%9C%E8%BD%AC%E5%BD%95%E7%BB%84%E6%B5%8B%E5%BA%8F\%E5%AE%8C%E6%95%B4%E7%89%88%E6%95%B0%E6%8D%AE\GDR3855-Hylocereus_undulatus_Britt-12-RNAseq_result\4_Function\2_Group_Diff_Function\UP_DOWN\GO\NL-VS-L0.F.html#gene22) | UDP-glycosyltransferase activity | 1 (7.14%) | 50 (1.46%) | 0.186050 | 0.406670 |
| 23 | [GO:0003676](file:///E:\2018-7-3%E7%81%AB%E9%BE%99%E6%9E%9C%E8%BD%AC%E5%BD%95%E7%BB%84%E6%B5%8B%E5%BA%8F\%E5%AE%8C%E6%95%B4%E7%89%88%E6%95%B0%E6%8D%AE\GDR3855-Hylocereus_undulatus_Britt-12-RNAseq_result\4_Function\2_Group_Diff_Function\UP_DOWN\GO\NL-VS-L0.F.html#gene23) | nucleic acid binding | 2 (14.29%) | 195 (5.68%) | 0.187068 | 0.406670 |
| 24 | [GO:0001883](file:///E:\2018-7-3%E7%81%AB%E9%BE%99%E6%9E%9C%E8%BD%AC%E5%BD%95%E7%BB%84%E6%B5%8B%E5%BA%8F\%E5%AE%8C%E6%95%B4%E7%89%88%E6%95%B0%E6%8D%AE\GDR3855-Hylocereus_undulatus_Britt-12-RNAseq_result\4_Function\2_Group_Diff_Function\UP_DOWN\GO\NL-VS-L0.F.html#gene24) | purine nucleoside binding | 4 (28.57%) | 607 (17.69%) | 0.224813 | 0.408063 |
| 25 | [GO:0032549](file:///E:\2018-7-3%E7%81%AB%E9%BE%99%E6%9E%9C%E8%BD%AC%E5%BD%95%E7%BB%84%E6%B5%8B%E5%BA%8F\%E5%AE%8C%E6%95%B4%E7%89%88%E6%95%B0%E6%8D%AE\GDR3855-Hylocereus_undulatus_Britt-12-RNAseq_result\4_Function\2_Group_Diff_Function\UP_DOWN\GO\NL-VS-L0.F.html#gene25) | ribonucleoside binding | 4 (28.57%) | 607 (17.69%) | 0.224813 | 0.408063 |
| 26 | [GO:0032550](file:///E:\2018-7-3%E7%81%AB%E9%BE%99%E6%9E%9C%E8%BD%AC%E5%BD%95%E7%BB%84%E6%B5%8B%E5%BA%8F\%E5%AE%8C%E6%95%B4%E7%89%88%E6%95%B0%E6%8D%AE\GDR3855-Hylocereus_undulatus_Britt-12-RNAseq_result\4_Function\2_Group_Diff_Function\UP_DOWN\GO\NL-VS-L0.F.html#gene26) | purine ribonucleoside binding | 4 (28.57%) | 607 (17.69%) | 0.224813 | 0.408063 |
| 27 | [GO:0001882](file:///E:\2018-7-3%E7%81%AB%E9%BE%99%E6%9E%9C%E8%BD%AC%E5%BD%95%E7%BB%84%E6%B5%8B%E5%BA%8F\%E5%AE%8C%E6%95%B4%E7%89%88%E6%95%B0%E6%8D%AE\GDR3855-Hylocereus_undulatus_Britt-12-RNAseq_result\4_Function\2_Group_Diff_Function\UP_DOWN\GO\NL-VS-L0.F.html#gene27) | nucleoside binding | 4 (28.57%) | 609 (17.74%) | 0.226662 | 0.408063 |
| 28 | [GO:0097367](file:///E:\2018-7-3%E7%81%AB%E9%BE%99%E6%9E%9C%E8%BD%AC%E5%BD%95%E7%BB%84%E6%B5%8B%E5%BA%8F\%E5%AE%8C%E6%95%B4%E7%89%88%E6%95%B0%E6%8D%AE\GDR3855-Hylocereus_undulatus_Britt-12-RNAseq_result\4_Function\2_Group_Diff_Function\UP_DOWN\GO\NL-VS-L0.F.html#gene28) | carbohydrate derivative binding | 4 (28.57%) | 611 (17.8%) | 0.228515 | 0.408063 |
| 29 | [GO:0003723](file:///E:\2018-7-3%E7%81%AB%E9%BE%99%E6%9E%9C%E8%BD%AC%E5%BD%95%E7%BB%84%E6%B5%8B%E5%BA%8F\%E5%AE%8C%E6%95%B4%E7%89%88%E6%95%B0%E6%8D%AE\GDR3855-Hylocereus_undulatus_Britt-12-RNAseq_result\4_Function\2_Group_Diff_Function\UP_DOWN\GO\NL-VS-L0.F.html#gene29) | RNA binding | 1 (7.14%) | 68 (1.98%) | 0.244754 | 0.421989 |
| 30 | [GO:0070011](file:///E:\2018-7-3%E7%81%AB%E9%BE%99%E6%9E%9C%E8%BD%AC%E5%BD%95%E7%BB%84%E6%B5%8B%E5%BA%8F\%E5%AE%8C%E6%95%B4%E7%89%88%E6%95%B0%E6%8D%AE\GDR3855-Hylocereus_undulatus_Britt-12-RNAseq_result\4_Function\2_Group_Diff_Function\UP_DOWN\GO\NL-VS-L0.F.html#gene30) | peptidase activity, acting on L-amino acid peptides | 1 (7.14%) | 75 (2.19%) | 0.266502 | 0.435417 |
| 31 | [GO:0016705](file:///E:\2018-7-3%E7%81%AB%E9%BE%99%E6%9E%9C%E8%BD%AC%E5%BD%95%E7%BB%84%E6%B5%8B%E5%BA%8F\%E5%AE%8C%E6%95%B4%E7%89%88%E6%95%B0%E6%8D%AE\GDR3855-Hylocereus_undulatus_Britt-12-RNAseq_result\4_Function\2_Group_Diff_Function\UP_DOWN\GO\NL-VS-L0.F.html#gene31) | oxidoreductase activity, acting on paired donors, with incorporation or reduction of molecular oxygen | 1 (7.14%) | 78 (2.27%) | 0.275643 | 0.435417 |
| 32 | [GO:0016758](file:///E:\2018-7-3%E7%81%AB%E9%BE%99%E6%9E%9C%E8%BD%AC%E5%BD%95%E7%BB%84%E6%B5%8B%E5%BA%8F\%E5%AE%8C%E6%95%B4%E7%89%88%E6%95%B0%E6%8D%AE\GDR3855-Hylocereus_undulatus_Britt-12-RNAseq_result\4_Function\2_Group_Diff_Function\UP_DOWN\GO\NL-VS-L0.F.html#gene32) | transferase activity, transferring hexosyl groups | 1 (7.14%) | 79 (2.3%) | 0.278667 | 0.435417 |
| 33 | [GO:0008233](file:///E:\2018-7-3%E7%81%AB%E9%BE%99%E6%9E%9C%E8%BD%AC%E5%BD%95%E7%BB%84%E6%B5%8B%E5%BA%8F\%E5%AE%8C%E6%95%B4%E7%89%88%E6%95%B0%E6%8D%AE\GDR3855-Hylocereus_undulatus_Britt-12-RNAseq_result\4_Function\2_Group_Diff_Function\UP_DOWN\GO\NL-VS-L0.F.html#gene33) | peptidase activity | 1 (7.14%) | 86 (2.51%) | 0.299506 | 0.453797 |
| 34 | [GO:0004672](file:///E:\2018-7-3%E7%81%AB%E9%BE%99%E6%9E%9C%E8%BD%AC%E5%BD%95%E7%BB%84%E6%B5%8B%E5%BA%8F\%E5%AE%8C%E6%95%B4%E7%89%88%E6%95%B0%E6%8D%AE\GDR3855-Hylocereus_undulatus_Britt-12-RNAseq_result\4_Function\2_Group_Diff_Function\UP_DOWN\GO\NL-VS-L0.F.html#gene34) | protein kinase activity | 2 (14.29%) | 281 (8.19%) | 0.320053 | 0.455983 |
| 35 | [GO:1901363](file:///E:\2018-7-3%E7%81%AB%E9%BE%99%E6%9E%9C%E8%BD%AC%E5%BD%95%E7%BB%84%E6%B5%8B%E5%BA%8F\%E5%AE%8C%E6%95%B4%E7%89%88%E6%95%B0%E6%8D%AE\GDR3855-Hylocereus_undulatus_Britt-12-RNAseq_result\4_Function\2_Group_Diff_Function\UP_DOWN\GO\NL-VS-L0.F.html#gene35) | heterocyclic compound binding | 5 (35.71%) | 931 (27.13%) | 0.323042 | 0.455983 |
| 36 | [GO:0016829](file:///E:\2018-7-3%E7%81%AB%E9%BE%99%E6%9E%9C%E8%BD%AC%E5%BD%95%E7%BB%84%E6%B5%8B%E5%BA%8F\%E5%AE%8C%E6%95%B4%E7%89%88%E6%95%B0%E6%8D%AE\GDR3855-Hylocereus_undulatus_Britt-12-RNAseq_result\4_Function\2_Group_Diff_Function\UP_DOWN\GO\NL-VS-L0.F.html#gene36) | lyase activity | 1 (7.14%) | 96 (2.8%) | 0.328308 | 0.455983 |
| 37 | [GO:0016773](file:///E:\2018-7-3%E7%81%AB%E9%BE%99%E6%9E%9C%E8%BD%AC%E5%BD%95%E7%BB%84%E6%B5%8B%E5%BA%8F\%E5%AE%8C%E6%95%B4%E7%89%88%E6%95%B0%E6%8D%AE\GDR3855-Hylocereus_undulatus_Britt-12-RNAseq_result\4_Function\2_Group_Diff_Function\UP_DOWN\GO\NL-VS-L0.F.html#gene37) | phosphotransferase activity, alcohol group as acceptor | 2 (14.29%) | 300 (8.74%) | 0.349611 | 0.472448 |
| 38 | [GO:0097159](file:///E:\2018-7-3%E7%81%AB%E9%BE%99%E6%9E%9C%E8%BD%AC%E5%BD%95%E7%BB%84%E6%B5%8B%E5%BA%8F\%E5%AE%8C%E6%95%B4%E7%89%88%E6%95%B0%E6%8D%AE\GDR3855-Hylocereus_undulatus_Britt-12-RNAseq_result\4_Function\2_Group_Diff_Function\UP_DOWN\GO\NL-VS-L0.F.html#gene38) | organic cyclic compound binding | 5 (35.71%) | 974 (28.38%) | 0.362997 | 0.477627 |
| 39 | [GO:0003824](file:///E:\2018-7-3%E7%81%AB%E9%BE%99%E6%9E%9C%E8%BD%AC%E5%BD%95%E7%BB%84%E6%B5%8B%E5%BA%8F\%E5%AE%8C%E6%95%B4%E7%89%88%E6%95%B0%E6%8D%AE\GDR3855-Hylocereus_undulatus_Britt-12-RNAseq_result\4_Function\2_Group_Diff_Function\UP_DOWN\GO\NL-VS-L0.F.html#gene39) | catalytic activity | 12 (85.71%) | 2677 (78%) | 0.375669 | 0.481627 |
| 40 | [GO:0016757](file:///E:\2018-7-3%E7%81%AB%E9%BE%99%E6%9E%9C%E8%BD%AC%E5%BD%95%E7%BB%84%E6%B5%8B%E5%BA%8F\%E5%AE%8C%E6%95%B4%E7%89%88%E6%95%B0%E6%8D%AE\GDR3855-Hylocereus_undulatus_Britt-12-RNAseq_result\4_Function\2_Group_Diff_Function\UP_DOWN\GO\NL-VS-L0.F.html#gene40) | transferase activity, transferring glycosyl groups | 1 (7.14%) | 122 (3.55%) | 0.398128 | 0.497659 |
| 41 | [GO:0016301](file:///E:\2018-7-3%E7%81%AB%E9%BE%99%E6%9E%9C%E8%BD%AC%E5%BD%95%E7%BB%84%E6%B5%8B%E5%BA%8F\%E5%AE%8C%E6%95%B4%E7%89%88%E6%95%B0%E6%8D%AE\GDR3855-Hylocereus_undulatus_Britt-12-RNAseq_result\4_Function\2_Group_Diff_Function\UP_DOWN\GO\NL-VS-L0.F.html#gene41) | kinase activity | 2 (14.29%) | 364 (10.61%) | 0.446468 | 0.544473 |
| 42 | [GO:0046914](file:///E:\2018-7-3%E7%81%AB%E9%BE%99%E6%9E%9C%E8%BD%AC%E5%BD%95%E7%BB%84%E6%B5%8B%E5%BA%8F\%E5%AE%8C%E6%95%B4%E7%89%88%E6%95%B0%E6%8D%AE\GDR3855-Hylocereus_undulatus_Britt-12-RNAseq_result\4_Function\2_Group_Diff_Function\UP_DOWN\GO\NL-VS-L0.F.html#gene42) | transition metal ion binding | 1 (7.14%) | 175 (5.1%) | 0.520083 | 0.619146 |
| 43 | [GO:0005488](file:///E:\2018-7-3%E7%81%AB%E9%BE%99%E6%9E%9C%E8%BD%AC%E5%BD%95%E7%BB%84%E6%B5%8B%E5%BA%8F\%E5%AE%8C%E6%95%B4%E7%89%88%E6%95%B0%E6%8D%AE\GDR3855-Hylocereus_undulatus_Britt-12-RNAseq_result\4_Function\2_Group_Diff_Function\UP_DOWN\GO\NL-VS-L0.F.html#gene43) | binding | 7 (50%) | 1688 (49.18%) | 0.580800 | 0.661542 |
| 44 | [GO:0005515](file:///E:\2018-7-3%E7%81%AB%E9%BE%99%E6%9E%9C%E8%BD%AC%E5%BD%95%E7%BB%84%E6%B5%8B%E5%BA%8F\%E5%AE%8C%E6%95%B4%E7%89%88%E6%95%B0%E6%8D%AE\GDR3855-Hylocereus_undulatus_Britt-12-RNAseq_result\4_Function\2_Group_Diff_Function\UP_DOWN\GO\NL-VS-L0.F.html#gene44) | protein binding | 1 (7.14%) | 207 (6.03%) | 0.582157 | 0.661542 |
| 45 | [GO:0043169](file:///E:\2018-7-3%E7%81%AB%E9%BE%99%E6%9E%9C%E8%BD%AC%E5%BD%95%E7%BB%84%E6%B5%8B%E5%BA%8F\%E5%AE%8C%E6%95%B4%E7%89%88%E6%95%B0%E6%8D%AE\GDR3855-Hylocereus_undulatus_Britt-12-RNAseq_result\4_Function\2_Group_Diff_Function\UP_DOWN\GO\NL-VS-L0.F.html#gene45) | cation binding | 2 (14.29%) | 502 (14.63%) | 0.629170 | 0.699078 |
| 46 | [GO:0016772](file:///E:\2018-7-3%E7%81%AB%E9%BE%99%E6%9E%9C%E8%BD%AC%E5%BD%95%E7%BB%84%E6%B5%8B%E5%BA%8F\%E5%AE%8C%E6%95%B4%E7%89%88%E6%95%B0%E6%8D%AE\GDR3855-Hylocereus_undulatus_Britt-12-RNAseq_result\4_Function\2_Group_Diff_Function\UP_DOWN\GO\NL-VS-L0.F.html#gene46) | transferase activity, transferring phosphorus-containing groups | 2 (14.29%) | 538 (15.68%) | 0.669476 | 0.727691 |
| 47 | [GO:0046872](file:///E:\2018-7-3%E7%81%AB%E9%BE%99%E6%9E%9C%E8%BD%AC%E5%BD%95%E7%BB%84%E6%B5%8B%E5%BA%8F\%E5%AE%8C%E6%95%B4%E7%89%88%E6%95%B0%E6%8D%AE\GDR3855-Hylocereus_undulatus_Britt-12-RNAseq_result\4_Function\2_Group_Diff_Function\UP_DOWN\GO\NL-VS-L0.F.html#gene47) | metal ion binding | 1 (7.14%) | 285 (8.3%) | 0.703621 | 0.735001 |
| 48 | [GO:0043167](file:///E:\2018-7-3%E7%81%AB%E9%BE%99%E6%9E%9C%E8%BD%AC%E5%BD%95%E7%BB%84%E6%B5%8B%E5%BA%8F\%E5%AE%8C%E6%95%B4%E7%89%88%E6%95%B0%E6%8D%AE\GDR3855-Hylocereus_undulatus_Britt-12-RNAseq_result\4_Function\2_Group_Diff_Function\UP_DOWN\GO\NL-VS-L0.F.html#gene48) | ion binding | 2 (14.29%) | 573 (16.7%) | 0.705601 | 0.735001 |
| 49 | [GO:0016740](file:///E:\2018-7-3%E7%81%AB%E9%BE%99%E6%9E%9C%E8%BD%AC%E5%BD%95%E7%BB%84%E6%B5%8B%E5%BA%8F\%E5%AE%8C%E6%95%B4%E7%89%88%E6%95%B0%E6%8D%AE\GDR3855-Hylocereus_undulatus_Britt-12-RNAseq_result\4_Function\2_Group_Diff_Function\UP_DOWN\GO\NL-VS-L0.F.html#gene49) | transferase activity | 3 (21.43%) | 974 (28.38%) | 0.805878 | 0.822324 |
| 50 | [GO:0016491](file:///E:\2018-7-3%E7%81%AB%E9%BE%99%E6%9E%9C%E8%BD%AC%E5%BD%95%E7%BB%84%E6%B5%8B%E5%BA%8F\%E5%AE%8C%E6%95%B4%E7%89%88%E6%95%B0%E6%8D%AE\GDR3855-Hylocereus_undulatus_Britt-12-RNAseq_result\4_Function\2_Group_Diff_Function\UP_DOWN\GO\NL-VS-L0.F.html#gene50) | oxidoreductase activity | 1 (7.14%) | 532 (15.5%) | 0.905856 | 0.905856 |
