# Supplementary material for: Transcriptomic analysis of flower induction for long-day pitaya by supplementary lighting in short-day winter season
Source: BMC Genomics. 2020 Apr 29;21:329. doi: 10.1186/s12864-020-6726-6 (PMC7191803; doi:10.1186/s12864-020-6726-6)
Supplement: Supplementary file 5 — Additional file 5: Supplemental S5. NL-VS-L0 GO Enrichment (Biological Process). [file 12864_2020_6726_MOESM5_ESM.docx]

Supplemental S5 NL-VS-L0 GO Enrichment (Biological Process)

| **#** | **GO ID** | **Description** | **GeneRatio (15)** | **BgRatio (3359)** | **pvalue** | **p.adjust** |
| --- | --- | --- | --- | --- | --- | --- |
| 1 | [GO:0001934](file:///E:\2018-7-3%E7%81%AB%E9%BE%99%E6%9E%9C%E8%BD%AC%E5%BD%95%E7%BB%84%E6%B5%8B%E5%BA%8F\%E5%AE%8C%E6%95%B4%E7%89%88%E6%95%B0%E6%8D%AE\GDR3855-Hylocereus_undulatus_Britt-12-RNAseq_result\4_Function\2_Group_Diff_Function\UP_DOWN\GO\NL-VS-L0.P.html#gene1) | positive regulation of protein phosphorylation | 2 (13.33%) | 36 (1.07%) | 0.010744 | 0.082553 |
| 2 | [GO:0010562](file:///E:\2018-7-3%E7%81%AB%E9%BE%99%E6%9E%9C%E8%BD%AC%E5%BD%95%E7%BB%84%E6%B5%8B%E5%BA%8F\%E5%AE%8C%E6%95%B4%E7%89%88%E6%95%B0%E6%8D%AE\GDR3855-Hylocereus_undulatus_Britt-12-RNAseq_result\4_Function\2_Group_Diff_Function\UP_DOWN\GO\NL-VS-L0.P.html#gene2) | positive regulation of phosphorus metabolic process | 2 (13.33%) | 36 (1.07%) | 0.010744 | 0.082553 |
| 3 | [GO:0031325](file:///E:\2018-7-3%E7%81%AB%E9%BE%99%E6%9E%9C%E8%BD%AC%E5%BD%95%E7%BB%84%E6%B5%8B%E5%BA%8F\%E5%AE%8C%E6%95%B4%E7%89%88%E6%95%B0%E6%8D%AE\GDR3855-Hylocereus_undulatus_Britt-12-RNAseq_result\4_Function\2_Group_Diff_Function\UP_DOWN\GO\NL-VS-L0.P.html#gene3) | positive regulation of cellular metabolic process | 2 (13.33%) | 36 (1.07%) | 0.010744 | 0.082553 |
| 4 | [GO:0031401](file:///E:\2018-7-3%E7%81%AB%E9%BE%99%E6%9E%9C%E8%BD%AC%E5%BD%95%E7%BB%84%E6%B5%8B%E5%BA%8F\%E5%AE%8C%E6%95%B4%E7%89%88%E6%95%B0%E6%8D%AE\GDR3855-Hylocereus_undulatus_Britt-12-RNAseq_result\4_Function\2_Group_Diff_Function\UP_DOWN\GO\NL-VS-L0.P.html#gene4) | positive regulation of protein modification process | 2 (13.33%) | 36 (1.07%) | 0.010744 | 0.082553 |
| 5 | [GO:0032147](file:///E:\2018-7-3%E7%81%AB%E9%BE%99%E6%9E%9C%E8%BD%AC%E5%BD%95%E7%BB%84%E6%B5%8B%E5%BA%8F\%E5%AE%8C%E6%95%B4%E7%89%88%E6%95%B0%E6%8D%AE\GDR3855-Hylocereus_undulatus_Britt-12-RNAseq_result\4_Function\2_Group_Diff_Function\UP_DOWN\GO\NL-VS-L0.P.html#gene5) | activation of protein kinase activity | 2 (13.33%) | 36 (1.07%) | 0.010744 | 0.082553 |
| 6 | [GO:0032270](file:///E:\2018-7-3%E7%81%AB%E9%BE%99%E6%9E%9C%E8%BD%AC%E5%BD%95%E7%BB%84%E6%B5%8B%E5%BA%8F\%E5%AE%8C%E6%95%B4%E7%89%88%E6%95%B0%E6%8D%AE\GDR3855-Hylocereus_undulatus_Britt-12-RNAseq_result\4_Function\2_Group_Diff_Function\UP_DOWN\GO\NL-VS-L0.P.html#gene6) | positive regulation of cellular protein metabolic process | 2 (13.33%) | 36 (1.07%) | 0.010744 | 0.082553 |
| 7 | [GO:0033674](file:///E:\2018-7-3%E7%81%AB%E9%BE%99%E6%9E%9C%E8%BD%AC%E5%BD%95%E7%BB%84%E6%B5%8B%E5%BA%8F\%E5%AE%8C%E6%95%B4%E7%89%88%E6%95%B0%E6%8D%AE\GDR3855-Hylocereus_undulatus_Britt-12-RNAseq_result\4_Function\2_Group_Diff_Function\UP_DOWN\GO\NL-VS-L0.P.html#gene7) | positive regulation of kinase activity | 2 (13.33%) | 36 (1.07%) | 0.010744 | 0.082553 |
| 8 | [GO:0042327](file:///E:\2018-7-3%E7%81%AB%E9%BE%99%E6%9E%9C%E8%BD%AC%E5%BD%95%E7%BB%84%E6%B5%8B%E5%BA%8F\%E5%AE%8C%E6%95%B4%E7%89%88%E6%95%B0%E6%8D%AE\GDR3855-Hylocereus_undulatus_Britt-12-RNAseq_result\4_Function\2_Group_Diff_Function\UP_DOWN\GO\NL-VS-L0.P.html#gene8) | positive regulation of phosphorylation | 2 (13.33%) | 36 (1.07%) | 0.010744 | 0.082553 |
| 9 | [GO:0043549](file:///E:\2018-7-3%E7%81%AB%E9%BE%99%E6%9E%9C%E8%BD%AC%E5%BD%95%E7%BB%84%E6%B5%8B%E5%BA%8F\%E5%AE%8C%E6%95%B4%E7%89%88%E6%95%B0%E6%8D%AE\GDR3855-Hylocereus_undulatus_Britt-12-RNAseq_result\4_Function\2_Group_Diff_Function\UP_DOWN\GO\NL-VS-L0.P.html#gene9) | regulation of kinase activity | 2 (13.33%) | 36 (1.07%) | 0.010744 | 0.082553 |
| 10 | [GO:0045859](file:///E:\2018-7-3%E7%81%AB%E9%BE%99%E6%9E%9C%E8%BD%AC%E5%BD%95%E7%BB%84%E6%B5%8B%E5%BA%8F\%E5%AE%8C%E6%95%B4%E7%89%88%E6%95%B0%E6%8D%AE\GDR3855-Hylocereus_undulatus_Britt-12-RNAseq_result\4_Function\2_Group_Diff_Function\UP_DOWN\GO\NL-VS-L0.P.html#gene10) | regulation of protein kinase activity | 2 (13.33%) | 36 (1.07%) | 0.010744 | 0.082553 |
| 11 | [GO:0045860](file:///E:\2018-7-3%E7%81%AB%E9%BE%99%E6%9E%9C%E8%BD%AC%E5%BD%95%E7%BB%84%E6%B5%8B%E5%BA%8F\%E5%AE%8C%E6%95%B4%E7%89%88%E6%95%B0%E6%8D%AE\GDR3855-Hylocereus_undulatus_Britt-12-RNAseq_result\4_Function\2_Group_Diff_Function\UP_DOWN\GO\NL-VS-L0.P.html#gene11) | positive regulation of protein kinase activity | 2 (13.33%) | 36 (1.07%) | 0.010744 | 0.082553 |
| 12 | [GO:0045937](file:///E:\2018-7-3%E7%81%AB%E9%BE%99%E6%9E%9C%E8%BD%AC%E5%BD%95%E7%BB%84%E6%B5%8B%E5%BA%8F\%E5%AE%8C%E6%95%B4%E7%89%88%E6%95%B0%E6%8D%AE\GDR3855-Hylocereus_undulatus_Britt-12-RNAseq_result\4_Function\2_Group_Diff_Function\UP_DOWN\GO\NL-VS-L0.P.html#gene12) | positive regulation of phosphate metabolic process | 2 (13.33%) | 36 (1.07%) | 0.010744 | 0.082553 |
| 13 | [GO:0051247](file:///E:\2018-7-3%E7%81%AB%E9%BE%99%E6%9E%9C%E8%BD%AC%E5%BD%95%E7%BB%84%E6%B5%8B%E5%BA%8F\%E5%AE%8C%E6%95%B4%E7%89%88%E6%95%B0%E6%8D%AE\GDR3855-Hylocereus_undulatus_Britt-12-RNAseq_result\4_Function\2_Group_Diff_Function\UP_DOWN\GO\NL-VS-L0.P.html#gene13) | positive regulation of protein metabolic process | 2 (13.33%) | 36 (1.07%) | 0.010744 | 0.082553 |
| 14 | [GO:0051338](file:///E:\2018-7-3%E7%81%AB%E9%BE%99%E6%9E%9C%E8%BD%AC%E5%BD%95%E7%BB%84%E6%B5%8B%E5%BA%8F\%E5%AE%8C%E6%95%B4%E7%89%88%E6%95%B0%E6%8D%AE\GDR3855-Hylocereus_undulatus_Britt-12-RNAseq_result\4_Function\2_Group_Diff_Function\UP_DOWN\GO\NL-VS-L0.P.html#gene14) | regulation of transferase activity | 2 (13.33%) | 36 (1.07%) | 0.010744 | 0.082553 |
| 15 | [GO:0051347](file:///E:\2018-7-3%E7%81%AB%E9%BE%99%E6%9E%9C%E8%BD%AC%E5%BD%95%E7%BB%84%E6%B5%8B%E5%BA%8F\%E5%AE%8C%E6%95%B4%E7%89%88%E6%95%B0%E6%8D%AE\GDR3855-Hylocereus_undulatus_Britt-12-RNAseq_result\4_Function\2_Group_Diff_Function\UP_DOWN\GO\NL-VS-L0.P.html#gene15) | positive regulation of transferase activity | 2 (13.33%) | 36 (1.07%) | 0.010744 | 0.082553 |
| 16 | [GO:0001932](file:///E:\2018-7-3%E7%81%AB%E9%BE%99%E6%9E%9C%E8%BD%AC%E5%BD%95%E7%BB%84%E6%B5%8B%E5%BA%8F\%E5%AE%8C%E6%95%B4%E7%89%88%E6%95%B0%E6%8D%AE\GDR3855-Hylocereus_undulatus_Britt-12-RNAseq_result\4_Function\2_Group_Diff_Function\UP_DOWN\GO\NL-VS-L0.P.html#gene16) | regulation of protein phosphorylation | 2 (13.33%) | 37 (1.1%) | 0.011329 | 0.082553 |
| 17 | [GO:0010604](file:///E:\2018-7-3%E7%81%AB%E9%BE%99%E6%9E%9C%E8%BD%AC%E5%BD%95%E7%BB%84%E6%B5%8B%E5%BA%8F\%E5%AE%8C%E6%95%B4%E7%89%88%E6%95%B0%E6%8D%AE\GDR3855-Hylocereus_undulatus_Britt-12-RNAseq_result\4_Function\2_Group_Diff_Function\UP_DOWN\GO\NL-VS-L0.P.html#gene17) | positive regulation of macromolecule metabolic process | 2 (13.33%) | 37 (1.1%) | 0.011329 | 0.082553 |
| 18 | [GO:0031399](file:///E:\2018-7-3%E7%81%AB%E9%BE%99%E6%9E%9C%E8%BD%AC%E5%BD%95%E7%BB%84%E6%B5%8B%E5%BA%8F\%E5%AE%8C%E6%95%B4%E7%89%88%E6%95%B0%E6%8D%AE\GDR3855-Hylocereus_undulatus_Britt-12-RNAseq_result\4_Function\2_Group_Diff_Function\UP_DOWN\GO\NL-VS-L0.P.html#gene18) | regulation of protein modification process | 2 (13.33%) | 37 (1.1%) | 0.011329 | 0.082553 |
| 19 | [GO:0042325](file:///E:\2018-7-3%E7%81%AB%E9%BE%99%E6%9E%9C%E8%BD%AC%E5%BD%95%E7%BB%84%E6%B5%8B%E5%BA%8F\%E5%AE%8C%E6%95%B4%E7%89%88%E6%95%B0%E6%8D%AE\GDR3855-Hylocereus_undulatus_Britt-12-RNAseq_result\4_Function\2_Group_Diff_Function\UP_DOWN\GO\NL-VS-L0.P.html#gene19) | regulation of phosphorylation | 2 (13.33%) | 37 (1.1%) | 0.011329 | 0.082553 |
| 20 | [GO:0019220](file:///E:\2018-7-3%E7%81%AB%E9%BE%99%E6%9E%9C%E8%BD%AC%E5%BD%95%E7%BB%84%E6%B5%8B%E5%BA%8F\%E5%AE%8C%E6%95%B4%E7%89%88%E6%95%B0%E6%8D%AE\GDR3855-Hylocereus_undulatus_Britt-12-RNAseq_result\4_Function\2_Group_Diff_Function\UP_DOWN\GO\NL-VS-L0.P.html#gene20) | regulation of phosphate metabolic process | 2 (13.33%) | 38 (1.13%) | 0.011927 | 0.082553 |
| 21 | [GO:0043085](file:///E:\2018-7-3%E7%81%AB%E9%BE%99%E6%9E%9C%E8%BD%AC%E5%BD%95%E7%BB%84%E6%B5%8B%E5%BA%8F\%E5%AE%8C%E6%95%B4%E7%89%88%E6%95%B0%E6%8D%AE\GDR3855-Hylocereus_undulatus_Britt-12-RNAseq_result\4_Function\2_Group_Diff_Function\UP_DOWN\GO\NL-VS-L0.P.html#gene21) | positive regulation of catalytic activity | 2 (13.33%) | 38 (1.13%) | 0.011927 | 0.082553 |
| 22 | [GO:0051174](file:///E:\2018-7-3%E7%81%AB%E9%BE%99%E6%9E%9C%E8%BD%AC%E5%BD%95%E7%BB%84%E6%B5%8B%E5%BA%8F\%E5%AE%8C%E6%95%B4%E7%89%88%E6%95%B0%E6%8D%AE\GDR3855-Hylocereus_undulatus_Britt-12-RNAseq_result\4_Function\2_Group_Diff_Function\UP_DOWN\GO\NL-VS-L0.P.html#gene22) | regulation of phosphorus metabolic process | 2 (13.33%) | 38 (1.13%) | 0.011927 | 0.082553 |
| 23 | [GO:0009893](file:///E:\2018-7-3%E7%81%AB%E9%BE%99%E6%9E%9C%E8%BD%AC%E5%BD%95%E7%BB%84%E6%B5%8B%E5%BA%8F\%E5%AE%8C%E6%95%B4%E7%89%88%E6%95%B0%E6%8D%AE\GDR3855-Hylocereus_undulatus_Britt-12-RNAseq_result\4_Function\2_Group_Diff_Function\UP_DOWN\GO\NL-VS-L0.P.html#gene23) | positive regulation of metabolic process | 2 (13.33%) | 39 (1.16%) | 0.012540 | 0.082553 |
| 24 | [GO:0048522](file:///E:\2018-7-3%E7%81%AB%E9%BE%99%E6%9E%9C%E8%BD%AC%E5%BD%95%E7%BB%84%E6%B5%8B%E5%BA%8F\%E5%AE%8C%E6%95%B4%E7%89%88%E6%95%B0%E6%8D%AE\GDR3855-Hylocereus_undulatus_Britt-12-RNAseq_result\4_Function\2_Group_Diff_Function\UP_DOWN\GO\NL-VS-L0.P.html#gene24) | positive regulation of cellular process | 2 (13.33%) | 39 (1.16%) | 0.012540 | 0.082553 |
| 25 | [GO:0044093](file:///E:\2018-7-3%E7%81%AB%E9%BE%99%E6%9E%9C%E8%BD%AC%E5%BD%95%E7%BB%84%E6%B5%8B%E5%BA%8F\%E5%AE%8C%E6%95%B4%E7%89%88%E6%95%B0%E6%8D%AE\GDR3855-Hylocereus_undulatus_Britt-12-RNAseq_result\4_Function\2_Group_Diff_Function\UP_DOWN\GO\NL-VS-L0.P.html#gene25) | positive regulation of molecular function | 2 (13.33%) | 41 (1.22%) | 0.013805 | 0.087250 |
| 26 | [GO:0032268](file:///E:\2018-7-3%E7%81%AB%E9%BE%99%E6%9E%9C%E8%BD%AC%E5%BD%95%E7%BB%84%E6%B5%8B%E5%BA%8F\%E5%AE%8C%E6%95%B4%E7%89%88%E6%95%B0%E6%8D%AE\GDR3855-Hylocereus_undulatus_Britt-12-RNAseq_result\4_Function\2_Group_Diff_Function\UP_DOWN\GO\NL-VS-L0.P.html#gene26) | regulation of cellular protein metabolic process | 2 (13.33%) | 45 (1.34%) | 0.016497 | 0.096536 |
| 27 | [GO:0051246](file:///E:\2018-7-3%E7%81%AB%E9%BE%99%E6%9E%9C%E8%BD%AC%E5%BD%95%E7%BB%84%E6%B5%8B%E5%BA%8F\%E5%AE%8C%E6%95%B4%E7%89%88%E6%95%B0%E6%8D%AE\GDR3855-Hylocereus_undulatus_Britt-12-RNAseq_result\4_Function\2_Group_Diff_Function\UP_DOWN\GO\NL-VS-L0.P.html#gene27) | regulation of protein metabolic process | 2 (13.33%) | 45 (1.34%) | 0.016497 | 0.096536 |
| 28 | [GO:0048518](file:///E:\2018-7-3%E7%81%AB%E9%BE%99%E6%9E%9C%E8%BD%AC%E5%BD%95%E7%BB%84%E6%B5%8B%E5%BA%8F\%E5%AE%8C%E6%95%B4%E7%89%88%E6%95%B0%E6%8D%AE\GDR3855-Hylocereus_undulatus_Britt-12-RNAseq_result\4_Function\2_Group_Diff_Function\UP_DOWN\GO\NL-VS-L0.P.html#gene28) | positive regulation of biological process | 2 (13.33%) | 49 (1.46%) | 0.019395 | 0.109446 |
| 29 | [GO:0009767](file:///E:\2018-7-3%E7%81%AB%E9%BE%99%E6%9E%9C%E8%BD%AC%E5%BD%95%E7%BB%84%E6%B5%8B%E5%BA%8F\%E5%AE%8C%E6%95%B4%E7%89%88%E6%95%B0%E6%8D%AE\GDR3855-Hylocereus_undulatus_Britt-12-RNAseq_result\4_Function\2_Group_Diff_Function\UP_DOWN\GO\NL-VS-L0.P.html#gene29) | photosynthetic electron transport chain | 1 (6.67%) | 6 (0.18%) | 0.026516 | 0.140299 |
| 30 | [GO:0050790](file:///E:\2018-7-3%E7%81%AB%E9%BE%99%E6%9E%9C%E8%BD%AC%E5%BD%95%E7%BB%84%E6%B5%8B%E5%BA%8F\%E5%AE%8C%E6%95%B4%E7%89%88%E6%95%B0%E6%8D%AE\GDR3855-Hylocereus_undulatus_Britt-12-RNAseq_result\4_Function\2_Group_Diff_Function\UP_DOWN\GO\NL-VS-L0.P.html#gene30) | regulation of catalytic activity | 2 (13.33%) | 58 (1.73%) | 0.026639 | 0.140299 |
| 31 | [GO:0080090](file:///E:\2018-7-3%E7%81%AB%E9%BE%99%E6%9E%9C%E8%BD%AC%E5%BD%95%E7%BB%84%E6%B5%8B%E5%BA%8F\%E5%AE%8C%E6%95%B4%E7%89%88%E6%95%B0%E6%8D%AE\GDR3855-Hylocereus_undulatus_Britt-12-RNAseq_result\4_Function\2_Group_Diff_Function\UP_DOWN\GO\NL-VS-L0.P.html#gene31) | regulation of primary metabolic process | 2 (13.33%) | 60 (1.79%) | 0.028378 | 0.144639 |
| 32 | [GO:0031323](file:///E:\2018-7-3%E7%81%AB%E9%BE%99%E6%9E%9C%E8%BD%AC%E5%BD%95%E7%BB%84%E6%B5%8B%E5%BA%8F\%E5%AE%8C%E6%95%B4%E7%89%88%E6%95%B0%E6%8D%AE\GDR3855-Hylocereus_undulatus_Britt-12-RNAseq_result\4_Function\2_Group_Diff_Function\UP_DOWN\GO\NL-VS-L0.P.html#gene32) | regulation of cellular metabolic process | 2 (13.33%) | 64 (1.91%) | 0.031992 | 0.157633 |
| 33 | [GO:0060255](file:///E:\2018-7-3%E7%81%AB%E9%BE%99%E6%9E%9C%E8%BD%AC%E5%BD%95%E7%BB%84%E6%B5%8B%E5%BA%8F\%E5%AE%8C%E6%95%B4%E7%89%88%E6%95%B0%E6%8D%AE\GDR3855-Hylocereus_undulatus_Britt-12-RNAseq_result\4_Function\2_Group_Diff_Function\UP_DOWN\GO\NL-VS-L0.P.html#gene33) | regulation of macromolecule metabolic process | 2 (13.33%) | 65 (1.94%) | 0.032923 | 0.157633 |
| 34 | [GO:0065009](file:///E:\2018-7-3%E7%81%AB%E9%BE%99%E6%9E%9C%E8%BD%AC%E5%BD%95%E7%BB%84%E6%B5%8B%E5%BA%8F\%E5%AE%8C%E6%95%B4%E7%89%88%E6%95%B0%E6%8D%AE\GDR3855-Hylocereus_undulatus_Britt-12-RNAseq_result\4_Function\2_Group_Diff_Function\UP_DOWN\GO\NL-VS-L0.P.html#gene34) | regulation of molecular function | 2 (13.33%) | 78 (2.32%) | 0.045974 | 0.213646 |
| 35 | [GO:0019684](file:///E:\2018-7-3%E7%81%AB%E9%BE%99%E6%9E%9C%E8%BD%AC%E5%BD%95%E7%BB%84%E6%B5%8B%E5%BA%8F\%E5%AE%8C%E6%95%B4%E7%89%88%E6%95%B0%E6%8D%AE\GDR3855-Hylocereus_undulatus_Britt-12-RNAseq_result\4_Function\2_Group_Diff_Function\UP_DOWN\GO\NL-VS-L0.P.html#gene35) | photosynthesis, light reaction | 1 (6.67%) | 11 (0.33%) | 0.048110 | 0.217180 |
| 36 | [GO:0015979](file:///E:\2018-7-3%E7%81%AB%E9%BE%99%E6%9E%9C%E8%BD%AC%E5%BD%95%E7%BB%84%E6%B5%8B%E5%BA%8F\%E5%AE%8C%E6%95%B4%E7%89%88%E6%95%B0%E6%8D%AE\GDR3855-Hylocereus_undulatus_Britt-12-RNAseq_result\4_Function\2_Group_Diff_Function\UP_DOWN\GO\NL-VS-L0.P.html#gene36) | photosynthesis | 1 (6.67%) | 12 (0.36%) | 0.052374 | 0.229865 |
| 37 | [GO:0043094](file:///E:\2018-7-3%E7%81%AB%E9%BE%99%E6%9E%9C%E8%BD%AC%E5%BD%95%E7%BB%84%E6%B5%8B%E5%BA%8F\%E5%AE%8C%E6%95%B4%E7%89%88%E6%95%B0%E6%8D%AE\GDR3855-Hylocereus_undulatus_Britt-12-RNAseq_result\4_Function\2_Group_Diff_Function\UP_DOWN\GO\NL-VS-L0.P.html#gene37) | cellular metabolic compound salvage | 1 (6.67%) | 14 (0.42%) | 0.060850 | 0.259848 |
| 38 | [GO:0019222](file:///E:\2018-7-3%E7%81%AB%E9%BE%99%E6%9E%9C%E8%BD%AC%E5%BD%95%E7%BB%84%E6%B5%8B%E5%BA%8F\%E5%AE%8C%E6%95%B4%E7%89%88%E6%95%B0%E6%8D%AE\GDR3855-Hylocereus_undulatus_Britt-12-RNAseq_result\4_Function\2_Group_Diff_Function\UP_DOWN\GO\NL-VS-L0.P.html#gene38) | regulation of metabolic process | 2 (13.33%) | 105 (3.13%) | 0.078012 | 0.324367 |
| 39 | [GO:0022900](file:///E:\2018-7-3%E7%81%AB%E9%BE%99%E6%9E%9C%E8%BD%AC%E5%BD%95%E7%BB%84%E6%B5%8B%E5%BA%8F\%E5%AE%8C%E6%95%B4%E7%89%88%E6%95%B0%E6%8D%AE\GDR3855-Hylocereus_undulatus_Britt-12-RNAseq_result\4_Function\2_Group_Diff_Function\UP_DOWN\GO\NL-VS-L0.P.html#gene39) | electron transport chain | 1 (6.67%) | 22 (0.65%) | 0.094052 | 0.381031 |
| 40 | [GO:0070838](file:///E:\2018-7-3%E7%81%AB%E9%BE%99%E6%9E%9C%E8%BD%AC%E5%BD%95%E7%BB%84%E6%B5%8B%E5%BA%8F\%E5%AE%8C%E6%95%B4%E7%89%88%E6%95%B0%E6%8D%AE\GDR3855-Hylocereus_undulatus_Britt-12-RNAseq_result\4_Function\2_Group_Diff_Function\UP_DOWN\GO\NL-VS-L0.P.html#gene40) | divalent metal ion transport | 1 (6.67%) | 26 (0.77%) | 0.110239 | 0.424823 |
| 41 | [GO:0072511](file:///E:\2018-7-3%E7%81%AB%E9%BE%99%E6%9E%9C%E8%BD%AC%E5%BD%95%E7%BB%84%E6%B5%8B%E5%BA%8F\%E5%AE%8C%E6%95%B4%E7%89%88%E6%95%B0%E6%8D%AE\GDR3855-Hylocereus_undulatus_Britt-12-RNAseq_result\4_Function\2_Group_Diff_Function\UP_DOWN\GO\NL-VS-L0.P.html#gene41) | divalent inorganic cation transport | 1 (6.67%) | 26 (0.77%) | 0.110239 | 0.424823 |
| 42 | [GO:0071555](file:///E:\2018-7-3%E7%81%AB%E9%BE%99%E6%9E%9C%E8%BD%AC%E5%BD%95%E7%BB%84%E6%B5%8B%E5%BA%8F\%E5%AE%8C%E6%95%B4%E7%89%88%E6%95%B0%E6%8D%AE\GDR3855-Hylocereus_undulatus_Britt-12-RNAseq_result\4_Function\2_Group_Diff_Function\UP_DOWN\GO\NL-VS-L0.P.html#gene42) | cell wall organization | 1 (6.67%) | 30 (0.89%) | 0.126155 | 0.474585 |
| 43 | [GO:0007017](file:///E:\2018-7-3%E7%81%AB%E9%BE%99%E6%9E%9C%E8%BD%AC%E5%BD%95%E7%BB%84%E6%B5%8B%E5%BA%8F\%E5%AE%8C%E6%95%B4%E7%89%88%E6%95%B0%E6%8D%AE\GDR3855-Hylocereus_undulatus_Britt-12-RNAseq_result\4_Function\2_Group_Diff_Function\UP_DOWN\GO\NL-VS-L0.P.html#gene43) | microtubule-based process | 1 (6.67%) | 31 (0.92%) | 0.130093 | 0.478016 |
| 44 | [GO:0009642](file:///E:\2018-7-3%E7%81%AB%E9%BE%99%E6%9E%9C%E8%BD%AC%E5%BD%95%E7%BB%84%E6%B5%8B%E5%BA%8F\%E5%AE%8C%E6%95%B4%E7%89%88%E6%95%B0%E6%8D%AE\GDR3855-Hylocereus_undulatus_Britt-12-RNAseq_result\4_Function\2_Group_Diff_Function\UP_DOWN\GO\NL-VS-L0.P.html#gene44) | response to light intensity | 1 (6.67%) | 33 (0.98%) | 0.137918 | 0.495251 |
| 45 | [GO:0045229](file:///E:\2018-7-3%E7%81%AB%E9%BE%99%E6%9E%9C%E8%BD%AC%E5%BD%95%E7%BB%84%E6%B5%8B%E5%BA%8F\%E5%AE%8C%E6%95%B4%E7%89%88%E6%95%B0%E6%8D%AE\GDR3855-Hylocereus_undulatus_Britt-12-RNAseq_result\4_Function\2_Group_Diff_Function\UP_DOWN\GO\NL-VS-L0.P.html#gene45) | external encapsulating structure organization | 1 (6.67%) | 35 (1.04%) | 0.145678 | 0.511490 |
| 46 | [GO:0071554](file:///E:\2018-7-3%E7%81%AB%E9%BE%99%E6%9E%9C%E8%BD%AC%E5%BD%95%E7%BB%84%E6%B5%8B%E5%BA%8F\%E5%AE%8C%E6%95%B4%E7%89%88%E6%95%B0%E6%8D%AE\GDR3855-Hylocereus_undulatus_Britt-12-RNAseq_result\4_Function\2_Group_Diff_Function\UP_DOWN\GO\NL-VS-L0.P.html#gene46) | cell wall organization or biogenesis | 1 (6.67%) | 45 (1.34%) | 0.183507 | 0.629319 |
| 47 | [GO:0006631](file:///E:\2018-7-3%E7%81%AB%E9%BE%99%E6%9E%9C%E8%BD%AC%E5%BD%95%E7%BB%84%E6%B5%8B%E5%BA%8F\%E5%AE%8C%E6%95%B4%E7%89%88%E6%95%B0%E6%8D%AE\GDR3855-Hylocereus_undulatus_Britt-12-RNAseq_result\4_Function\2_Group_Diff_Function\UP_DOWN\GO\NL-VS-L0.P.html#gene47) | fatty acid metabolic process | 1 (6.67%) | 46 (1.37%) | 0.187202 | 0.629319 |
| 48 | [GO:0055114](file:///E:\2018-7-3%E7%81%AB%E9%BE%99%E6%9E%9C%E8%BD%AC%E5%BD%95%E7%BB%84%E6%B5%8B%E5%BA%8F\%E5%AE%8C%E6%95%B4%E7%89%88%E6%95%B0%E6%8D%AE\GDR3855-Hylocereus_undulatus_Britt-12-RNAseq_result\4_Function\2_Group_Diff_Function\UP_DOWN\GO\NL-VS-L0.P.html#gene48) | oxidation-reduction process | 1 (6.67%) | 66 (1.96%) | 0.257916 | 0.778955 |
| 49 | [GO:0033554](file:///E:\2018-7-3%E7%81%AB%E9%BE%99%E6%9E%9C%E8%BD%AC%E5%BD%95%E7%BB%84%E6%B5%8B%E5%BA%8F\%E5%AE%8C%E6%95%B4%E7%89%88%E6%95%B0%E6%8D%AE\GDR3855-Hylocereus_undulatus_Britt-12-RNAseq_result\4_Function\2_Group_Diff_Function\UP_DOWN\GO\NL-VS-L0.P.html#gene49) | cellular response to stress | 1 (6.67%) | 67 (1.99%) | 0.261296 | 0.778955 |
| 50 | [GO:0032787](file:///E:\2018-7-3%E7%81%AB%E9%BE%99%E6%9E%9C%E8%BD%AC%E5%BD%95%E7%BB%84%E6%B5%8B%E5%BA%8F\%E5%AE%8C%E6%95%B4%E7%89%88%E6%95%B0%E6%8D%AE\GDR3855-Hylocereus_undulatus_Britt-12-RNAseq_result\4_Function\2_Group_Diff_Function\UP_DOWN\GO\NL-VS-L0.P.html#gene50) | monocarboxylic acid metabolic process | 1 (6.67%) | 69 (2.05%) | 0.268014 | 0.778955 |
| 51 | [GO:0009725](file:///E:\2018-7-3%E7%81%AB%E9%BE%99%E6%9E%9C%E8%BD%AC%E5%BD%95%E7%BB%84%E6%B5%8B%E5%BA%8F\%E5%AE%8C%E6%95%B4%E7%89%88%E6%95%B0%E6%8D%AE\GDR3855-Hylocereus_undulatus_Britt-12-RNAseq_result\4_Function\2_Group_Diff_Function\UP_DOWN\GO\NL-VS-L0.P.html#gene51) | response to hormone | 1 (6.67%) | 70 (2.08%) | 0.271351 | 0.778955 |
| 52 | [GO:0030001](file:///E:\2018-7-3%E7%81%AB%E9%BE%99%E6%9E%9C%E8%BD%AC%E5%BD%95%E7%BB%84%E6%B5%8B%E5%BA%8F\%E5%AE%8C%E6%95%B4%E7%89%88%E6%95%B0%E6%8D%AE\GDR3855-Hylocereus_undulatus_Britt-12-RNAseq_result\4_Function\2_Group_Diff_Function\UP_DOWN\GO\NL-VS-L0.P.html#gene52) | metal ion transport | 1 (6.67%) | 71 (2.11%) | 0.274674 | 0.778955 |
| 53 | [GO:0044249](file:///E:\2018-7-3%E7%81%AB%E9%BE%99%E6%9E%9C%E8%BD%AC%E5%BD%95%E7%BB%84%E6%B5%8B%E5%BA%8F\%E5%AE%8C%E6%95%B4%E7%89%88%E6%95%B0%E6%8D%AE\GDR3855-Hylocereus_undulatus_Britt-12-RNAseq_result\4_Function\2_Group_Diff_Function\UP_DOWN\GO\NL-VS-L0.P.html#gene53) | cellular biosynthetic process | 3 (20%) | 429 (12.77%) | 0.298346 | 0.778955 |
| 54 | [GO:0006412](file:///E:\2018-7-3%E7%81%AB%E9%BE%99%E6%9E%9C%E8%BD%AC%E5%BD%95%E7%BB%84%E6%B5%8B%E5%BA%8F\%E5%AE%8C%E6%95%B4%E7%89%88%E6%95%B0%E6%8D%AE\GDR3855-Hylocereus_undulatus_Britt-12-RNAseq_result\4_Function\2_Group_Diff_Function\UP_DOWN\GO\NL-VS-L0.P.html#gene54) | translation | 1 (6.67%) | 79 (2.35%) | 0.300755 | 0.778955 |
| 55 | [GO:0009719](file:///E:\2018-7-3%E7%81%AB%E9%BE%99%E6%9E%9C%E8%BD%AC%E5%BD%95%E7%BB%84%E6%B5%8B%E5%BA%8F\%E5%AE%8C%E6%95%B4%E7%89%88%E6%95%B0%E6%8D%AE\GDR3855-Hylocereus_undulatus_Britt-12-RNAseq_result\4_Function\2_Group_Diff_Function\UP_DOWN\GO\NL-VS-L0.P.html#gene55) | response to endogenous stimulus | 1 (6.67%) | 79 (2.35%) | 0.300755 | 0.778955 |
| 56 | [GO:0043043](file:///E:\2018-7-3%E7%81%AB%E9%BE%99%E6%9E%9C%E8%BD%AC%E5%BD%95%E7%BB%84%E6%B5%8B%E5%BA%8F\%E5%AE%8C%E6%95%B4%E7%89%88%E6%95%B0%E6%8D%AE\GDR3855-Hylocereus_undulatus_Britt-12-RNAseq_result\4_Function\2_Group_Diff_Function\UP_DOWN\GO\NL-VS-L0.P.html#gene56) | peptide biosynthetic process | 1 (6.67%) | 80 (2.38%) | 0.303952 | 0.778955 |
| 57 | [GO:0006259](file:///E:\2018-7-3%E7%81%AB%E9%BE%99%E6%9E%9C%E8%BD%AC%E5%BD%95%E7%BB%84%E6%B5%8B%E5%BA%8F\%E5%AE%8C%E6%95%B4%E7%89%88%E6%95%B0%E6%8D%AE\GDR3855-Hylocereus_undulatus_Britt-12-RNAseq_result\4_Function\2_Group_Diff_Function\UP_DOWN\GO\NL-VS-L0.P.html#gene57) | DNA metabolic process | 1 (6.67%) | 81 (2.41%) | 0.307136 | 0.778955 |
| 58 | [GO:0050794](file:///E:\2018-7-3%E7%81%AB%E9%BE%99%E6%9E%9C%E8%BD%AC%E5%BD%95%E7%BB%84%E6%B5%8B%E5%BA%8F\%E5%AE%8C%E6%95%B4%E7%89%88%E6%95%B0%E6%8D%AE\GDR3855-Hylocereus_undulatus_Britt-12-RNAseq_result\4_Function\2_Group_Diff_Function\UP_DOWN\GO\NL-VS-L0.P.html#gene58) | regulation of cellular process | 2 (13.33%) | 251 (7.47%) | 0.310227 | 0.778955 |
| 59 | [GO:0006518](file:///E:\2018-7-3%E7%81%AB%E9%BE%99%E6%9E%9C%E8%BD%AC%E5%BD%95%E7%BB%84%E6%B5%8B%E5%BA%8F\%E5%AE%8C%E6%95%B4%E7%89%88%E6%95%B0%E6%8D%AE\GDR3855-Hylocereus_undulatus_Britt-12-RNAseq_result\4_Function\2_Group_Diff_Function\UP_DOWN\GO\NL-VS-L0.P.html#gene59) | peptide metabolic process | 1 (6.67%) | 82 (2.44%) | 0.310307 | 0.778955 |
| 60 | [GO:0043604](file:///E:\2018-7-3%E7%81%AB%E9%BE%99%E6%9E%9C%E8%BD%AC%E5%BD%95%E7%BB%84%E6%B5%8B%E5%BA%8F\%E5%AE%8C%E6%95%B4%E7%89%88%E6%95%B0%E6%8D%AE\GDR3855-Hylocereus_undulatus_Britt-12-RNAseq_result\4_Function\2_Group_Diff_Function\UP_DOWN\GO\NL-VS-L0.P.html#gene60) | amide biosynthetic process | 1 (6.67%) | 82 (2.44%) | 0.310307 | 0.778955 |
| 61 | [GO:0009416](file:///E:\2018-7-3%E7%81%AB%E9%BE%99%E6%9E%9C%E8%BD%AC%E5%BD%95%E7%BB%84%E6%B5%8B%E5%BA%8F\%E5%AE%8C%E6%95%B4%E7%89%88%E6%95%B0%E6%8D%AE\GDR3855-Hylocereus_undulatus_Britt-12-RNAseq_result\4_Function\2_Group_Diff_Function\UP_DOWN\GO\NL-VS-L0.P.html#gene61) | response to light stimulus | 1 (6.67%) | 85 (2.53%) | 0.319737 | 0.778955 |
| 62 | [GO:0016485](file:///E:\2018-7-3%E7%81%AB%E9%BE%99%E6%9E%9C%E8%BD%AC%E5%BD%95%E7%BB%84%E6%B5%8B%E5%BA%8F\%E5%AE%8C%E6%95%B4%E7%89%88%E6%95%B0%E6%8D%AE\GDR3855-Hylocereus_undulatus_Britt-12-RNAseq_result\4_Function\2_Group_Diff_Function\UP_DOWN\GO\NL-VS-L0.P.html#gene62) | protein processing | 1 (6.67%) | 85 (2.53%) | 0.319737 | 0.778955 |
| 63 | [GO:0034645](file:///E:\2018-7-3%E7%81%AB%E9%BE%99%E6%9E%9C%E8%BD%AC%E5%BD%95%E7%BB%84%E6%B5%8B%E5%BA%8F\%E5%AE%8C%E6%95%B4%E7%89%88%E6%95%B0%E6%8D%AE\GDR3855-Hylocereus_undulatus_Britt-12-RNAseq_result\4_Function\2_Group_Diff_Function\UP_DOWN\GO\NL-VS-L0.P.html#gene63) | cellular macromolecule biosynthetic process | 2 (13.33%) | 259 (7.71%) | 0.323860 | 0.778955 |
| 64 | [GO:0051604](file:///E:\2018-7-3%E7%81%AB%E9%BE%99%E6%9E%9C%E8%BD%AC%E5%BD%95%E7%BB%84%E6%B5%8B%E5%BA%8F\%E5%AE%8C%E6%95%B4%E7%89%88%E6%95%B0%E6%8D%AE\GDR3855-Hylocereus_undulatus_Britt-12-RNAseq_result\4_Function\2_Group_Diff_Function\UP_DOWN\GO\NL-VS-L0.P.html#gene64) | protein maturation | 1 (6.67%) | 89 (2.65%) | 0.332124 | 0.778955 |
| 65 | [GO:0009059](file:///E:\2018-7-3%E7%81%AB%E9%BE%99%E6%9E%9C%E8%BD%AC%E5%BD%95%E7%BB%84%E6%B5%8B%E5%BA%8F\%E5%AE%8C%E6%95%B4%E7%89%88%E6%95%B0%E6%8D%AE\GDR3855-Hylocereus_undulatus_Britt-12-RNAseq_result\4_Function\2_Group_Diff_Function\UP_DOWN\GO\NL-VS-L0.P.html#gene65) | macromolecule biosynthetic process | 2 (13.33%) | 266 (7.92%) | 0.335759 | 0.778955 |
| 66 | [GO:0006950](file:///E:\2018-7-3%E7%81%AB%E9%BE%99%E6%9E%9C%E8%BD%AC%E5%BD%95%E7%BB%84%E6%B5%8B%E5%BA%8F\%E5%AE%8C%E6%95%B4%E7%89%88%E6%95%B0%E6%8D%AE\GDR3855-Hylocereus_undulatus_Britt-12-RNAseq_result\4_Function\2_Group_Diff_Function\UP_DOWN\GO\NL-VS-L0.P.html#gene66) | response to stress | 2 (13.33%) | 273 (8.13%) | 0.347620 | 0.778955 |
| 67 | [GO:0043603](file:///E:\2018-7-3%E7%81%AB%E9%BE%99%E6%9E%9C%E8%BD%AC%E5%BD%95%E7%BB%84%E6%B5%8B%E5%BA%8F\%E5%AE%8C%E6%95%B4%E7%89%88%E6%95%B0%E6%8D%AE\GDR3855-Hylocereus_undulatus_Britt-12-RNAseq_result\4_Function\2_Group_Diff_Function\UP_DOWN\GO\NL-VS-L0.P.html#gene67) | cellular amide metabolic process | 1 (6.67%) | 95 (2.83%) | 0.350310 | 0.778955 |
| 68 | [GO:0009314](file:///E:\2018-7-3%E7%81%AB%E9%BE%99%E6%9E%9C%E8%BD%AC%E5%BD%95%E7%BB%84%E6%B5%8B%E5%BA%8F\%E5%AE%8C%E6%95%B4%E7%89%88%E6%95%B0%E6%8D%AE\GDR3855-Hylocereus_undulatus_Britt-12-RNAseq_result\4_Function\2_Group_Diff_Function\UP_DOWN\GO\NL-VS-L0.P.html#gene68) | response to radiation | 1 (6.67%) | 96 (2.86%) | 0.353296 | 0.778955 |
| 69 | [GO:0009058](file:///E:\2018-7-3%E7%81%AB%E9%BE%99%E6%9E%9C%E8%BD%AC%E5%BD%95%E7%BB%84%E6%B5%8B%E5%BA%8F\%E5%AE%8C%E6%95%B4%E7%89%88%E6%95%B0%E6%8D%AE\GDR3855-Hylocereus_undulatus_Britt-12-RNAseq_result\4_Function\2_Group_Diff_Function\UP_DOWN\GO\NL-VS-L0.P.html#gene69) | biosynthetic process | 3 (20%) | 473 (14.08%) | 0.355550 | 0.778955 |
| 70 | [GO:0044255](file:///E:\2018-7-3%E7%81%AB%E9%BE%99%E6%9E%9C%E8%BD%AC%E5%BD%95%E7%BB%84%E6%B5%8B%E5%BA%8F\%E5%AE%8C%E6%95%B4%E7%89%88%E6%95%B0%E6%8D%AE\GDR3855-Hylocereus_undulatus_Britt-12-RNAseq_result\4_Function\2_Group_Diff_Function\UP_DOWN\GO\NL-VS-L0.P.html#gene70) | cellular lipid metabolic process | 1 (6.67%) | 107 (3.19%) | 0.385305 | 0.778955 |
| 71 | [GO:0010033](file:///E:\2018-7-3%E7%81%AB%E9%BE%99%E6%9E%9C%E8%BD%AC%E5%BD%95%E7%BB%84%E6%B5%8B%E5%BA%8F\%E5%AE%8C%E6%95%B4%E7%89%88%E6%95%B0%E6%8D%AE\GDR3855-Hylocereus_undulatus_Britt-12-RNAseq_result\4_Function\2_Group_Diff_Function\UP_DOWN\GO\NL-VS-L0.P.html#gene71) | response to organic substance | 1 (6.67%) | 111 (3.3%) | 0.396573 | 0.778955 |
| 72 | [GO:0050789](file:///E:\2018-7-3%E7%81%AB%E9%BE%99%E6%9E%9C%E8%BD%AC%E5%BD%95%E7%BB%84%E6%B5%8B%E5%BA%8F\%E5%AE%8C%E6%95%B4%E7%89%88%E6%95%B0%E6%8D%AE\GDR3855-Hylocereus_undulatus_Britt-12-RNAseq_result\4_Function\2_Group_Diff_Function\UP_DOWN\GO\NL-VS-L0.P.html#gene72) | regulation of biological process | 2 (13.33%) | 303 (9.02%) | 0.397841 | 0.778955 |
| 73 | [GO:0010467](file:///E:\2018-7-3%E7%81%AB%E9%BE%99%E6%9E%9C%E8%BD%AC%E5%BD%95%E7%BB%84%E6%B5%8B%E5%BA%8F\%E5%AE%8C%E6%95%B4%E7%89%88%E6%95%B0%E6%8D%AE\GDR3855-Hylocereus_undulatus_Britt-12-RNAseq_result\4_Function\2_Group_Diff_Function\UP_DOWN\GO\NL-VS-L0.P.html#gene73) | gene expression | 3 (20%) | 517 (15.39%) | 0.412947 | 0.778955 |
| 74 | [GO:0006091](file:///E:\2018-7-3%E7%81%AB%E9%BE%99%E6%9E%9C%E8%BD%AC%E5%BD%95%E7%BB%84%E6%B5%8B%E5%BA%8F\%E5%AE%8C%E6%95%B4%E7%89%88%E6%95%B0%E6%8D%AE\GDR3855-Hylocereus_undulatus_Britt-12-RNAseq_result\4_Function\2_Group_Diff_Function\UP_DOWN\GO\NL-VS-L0.P.html#gene74) | generation of precursor metabolites and energy | 1 (6.67%) | 119 (3.54%) | 0.418533 | 0.778955 |
| 75 | [GO:0044271](file:///E:\2018-7-3%E7%81%AB%E9%BE%99%E6%9E%9C%E8%BD%AC%E5%BD%95%E7%BB%84%E6%B5%8B%E5%BA%8F\%E5%AE%8C%E6%95%B4%E7%89%88%E6%95%B0%E6%8D%AE\GDR3855-Hylocereus_undulatus_Britt-12-RNAseq_result\4_Function\2_Group_Diff_Function\UP_DOWN\GO\NL-VS-L0.P.html#gene75) | cellular nitrogen compound biosynthetic process | 2 (13.33%) | 316 (9.41%) | 0.419194 | 0.778955 |
| 76 | [GO:0006812](file:///E:\2018-7-3%E7%81%AB%E9%BE%99%E6%9E%9C%E8%BD%AC%E5%BD%95%E7%BB%84%E6%B5%8B%E5%BA%8F\%E5%AE%8C%E6%95%B4%E7%89%88%E6%95%B0%E6%8D%AE\GDR3855-Hylocereus_undulatus_Britt-12-RNAseq_result\4_Function\2_Group_Diff_Function\UP_DOWN\GO\NL-VS-L0.P.html#gene76) | cation transport | 1 (6.67%) | 125 (3.72%) | 0.434511 | 0.778955 |
| 77 | [GO:0006629](file:///E:\2018-7-3%E7%81%AB%E9%BE%99%E6%9E%9C%E8%BD%AC%E5%BD%95%E7%BB%84%E6%B5%8B%E5%BA%8F\%E5%AE%8C%E6%95%B4%E7%89%88%E6%95%B0%E6%8D%AE\GDR3855-Hylocereus_undulatus_Britt-12-RNAseq_result\4_Function\2_Group_Diff_Function\UP_DOWN\GO\NL-VS-L0.P.html#gene77) | lipid metabolic process | 1 (6.67%) | 126 (3.75%) | 0.437134 | 0.778955 |
| 78 | [GO:0006508](file:///E:\2018-7-3%E7%81%AB%E9%BE%99%E6%9E%9C%E8%BD%AC%E5%BD%95%E7%BB%84%E6%B5%8B%E5%BA%8F\%E5%AE%8C%E6%95%B4%E7%89%88%E6%95%B0%E6%8D%AE\GDR3855-Hylocereus_undulatus_Britt-12-RNAseq_result\4_Function\2_Group_Diff_Function\UP_DOWN\GO\NL-VS-L0.P.html#gene78) | proteolysis | 1 (6.67%) | 132 (3.93%) | 0.452634 | 0.778955 |
| 79 | [GO:0090304](file:///E:\2018-7-3%E7%81%AB%E9%BE%99%E6%9E%9C%E8%BD%AC%E5%BD%95%E7%BB%84%E6%B5%8B%E5%BA%8F\%E5%AE%8C%E6%95%B4%E7%89%88%E6%95%B0%E6%8D%AE\GDR3855-Hylocereus_undulatus_Britt-12-RNAseq_result\4_Function\2_Group_Diff_Function\UP_DOWN\GO\NL-VS-L0.P.html#gene79) | nucleic acid metabolic process | 2 (13.33%) | 350 (10.42%) | 0.473507 | 0.778955 |
| 80 | [GO:0006139](file:///E:\2018-7-3%E7%81%AB%E9%BE%99%E6%9E%9C%E8%BD%AC%E5%BD%95%E7%BB%84%E6%B5%8B%E5%BA%8F\%E5%AE%8C%E6%95%B4%E7%89%88%E6%95%B0%E6%8D%AE\GDR3855-Hylocereus_undulatus_Britt-12-RNAseq_result\4_Function\2_Group_Diff_Function\UP_DOWN\GO\NL-VS-L0.P.html#gene80) | nucleobase-containing compound metabolic process | 3 (20%) | 568 (16.91%) | 0.478315 | 0.778955 |
| 81 | [GO:0006195](file:///E:\2018-7-3%E7%81%AB%E9%BE%99%E6%9E%9C%E8%BD%AC%E5%BD%95%E7%BB%84%E6%B5%8B%E5%BA%8F\%E5%AE%8C%E6%95%B4%E7%89%88%E6%95%B0%E6%8D%AE\GDR3855-Hylocereus_undulatus_Britt-12-RNAseq_result\4_Function\2_Group_Diff_Function\UP_DOWN\GO\NL-VS-L0.P.html#gene81) | purine nucleotide catabolic process | 1 (6.67%) | 145 (4.32%) | 0.484862 | 0.778955 |
| 82 | [GO:0009154](file:///E:\2018-7-3%E7%81%AB%E9%BE%99%E6%9E%9C%E8%BD%AC%E5%BD%95%E7%BB%84%E6%B5%8B%E5%BA%8F\%E5%AE%8C%E6%95%B4%E7%89%88%E6%95%B0%E6%8D%AE\GDR3855-Hylocereus_undulatus_Britt-12-RNAseq_result\4_Function\2_Group_Diff_Function\UP_DOWN\GO\NL-VS-L0.P.html#gene82) | purine ribonucleotide catabolic process | 1 (6.67%) | 145 (4.32%) | 0.484862 | 0.778955 |
| 83 | [GO:0009166](file:///E:\2018-7-3%E7%81%AB%E9%BE%99%E6%9E%9C%E8%BD%AC%E5%BD%95%E7%BB%84%E6%B5%8B%E5%BA%8F\%E5%AE%8C%E6%95%B4%E7%89%88%E6%95%B0%E6%8D%AE\GDR3855-Hylocereus_undulatus_Britt-12-RNAseq_result\4_Function\2_Group_Diff_Function\UP_DOWN\GO\NL-VS-L0.P.html#gene83) | nucleotide catabolic process | 1 (6.67%) | 145 (4.32%) | 0.484862 | 0.778955 |
| 84 | [GO:0009261](file:///E:\2018-7-3%E7%81%AB%E9%BE%99%E6%9E%9C%E8%BD%AC%E5%BD%95%E7%BB%84%E6%B5%8B%E5%BA%8F\%E5%AE%8C%E6%95%B4%E7%89%88%E6%95%B0%E6%8D%AE\GDR3855-Hylocereus_undulatus_Britt-12-RNAseq_result\4_Function\2_Group_Diff_Function\UP_DOWN\GO\NL-VS-L0.P.html#gene84) | ribonucleotide catabolic process | 1 (6.67%) | 145 (4.32%) | 0.484862 | 0.778955 |
| 85 | [GO:0046434](file:///E:\2018-7-3%E7%81%AB%E9%BE%99%E6%9E%9C%E8%BD%AC%E5%BD%95%E7%BB%84%E6%B5%8B%E5%BA%8F\%E5%AE%8C%E6%95%B4%E7%89%88%E6%95%B0%E6%8D%AE\GDR3855-Hylocereus_undulatus_Britt-12-RNAseq_result\4_Function\2_Group_Diff_Function\UP_DOWN\GO\NL-VS-L0.P.html#gene85) | organophosphate catabolic process | 1 (6.67%) | 145 (4.32%) | 0.484862 | 0.778955 |
| 86 | [GO:0072523](file:///E:\2018-7-3%E7%81%AB%E9%BE%99%E6%9E%9C%E8%BD%AC%E5%BD%95%E7%BB%84%E6%B5%8B%E5%BA%8F\%E5%AE%8C%E6%95%B4%E7%89%88%E6%95%B0%E6%8D%AE\GDR3855-Hylocereus_undulatus_Britt-12-RNAseq_result\4_Function\2_Group_Diff_Function\UP_DOWN\GO\NL-VS-L0.P.html#gene86) | purine-containing compound catabolic process | 1 (6.67%) | 145 (4.32%) | 0.484862 | 0.778955 |
| 87 | [GO:1901292](file:///E:\2018-7-3%E7%81%AB%E9%BE%99%E6%9E%9C%E8%BD%AC%E5%BD%95%E7%BB%84%E6%B5%8B%E5%BA%8F\%E5%AE%8C%E6%95%B4%E7%89%88%E6%95%B0%E6%8D%AE\GDR3855-Hylocereus_undulatus_Britt-12-RNAseq_result\4_Function\2_Group_Diff_Function\UP_DOWN\GO\NL-VS-L0.P.html#gene87) | nucleoside phosphate catabolic process | 1 (6.67%) | 145 (4.32%) | 0.484862 | 0.778955 |
| 88 | [GO:1901136](file:///E:\2018-7-3%E7%81%AB%E9%BE%99%E6%9E%9C%E8%BD%AC%E5%BD%95%E7%BB%84%E6%B5%8B%E5%BA%8F\%E5%AE%8C%E6%95%B4%E7%89%88%E6%95%B0%E6%8D%AE\GDR3855-Hylocereus_undulatus_Britt-12-RNAseq_result\4_Function\2_Group_Diff_Function\UP_DOWN\GO\NL-VS-L0.P.html#gene88) | carbohydrate derivative catabolic process | 1 (6.67%) | 147 (4.38%) | 0.489660 | 0.778955 |
| 89 | [GO:0034655](file:///E:\2018-7-3%E7%81%AB%E9%BE%99%E6%9E%9C%E8%BD%AC%E5%BD%95%E7%BB%84%E6%B5%8B%E5%BA%8F\%E5%AE%8C%E6%95%B4%E7%89%88%E6%95%B0%E6%8D%AE\GDR3855-Hylocereus_undulatus_Britt-12-RNAseq_result\4_Function\2_Group_Diff_Function\UP_DOWN\GO\NL-VS-L0.P.html#gene89) | nucleobase-containing compound catabolic process | 1 (6.67%) | 151 (4.5%) | 0.499131 | 0.778955 |
| 90 | [GO:1901565](file:///E:\2018-7-3%E7%81%AB%E9%BE%99%E6%9E%9C%E8%BD%AC%E5%BD%95%E7%BB%84%E6%B5%8B%E5%BA%8F\%E5%AE%8C%E6%95%B4%E7%89%88%E6%95%B0%E6%8D%AE\GDR3855-Hylocereus_undulatus_Britt-12-RNAseq_result\4_Function\2_Group_Diff_Function\UP_DOWN\GO\NL-VS-L0.P.html#gene90) | organonitrogen compound catabolic process | 1 (6.67%) | 151 (4.5%) | 0.499131 | 0.778955 |
| 91 | [GO:0050896](file:///E:\2018-7-3%E7%81%AB%E9%BE%99%E6%9E%9C%E8%BD%AC%E5%BD%95%E7%BB%84%E6%B5%8B%E5%BA%8F\%E5%AE%8C%E6%95%B4%E7%89%88%E6%95%B0%E6%8D%AE\GDR3855-Hylocereus_undulatus_Britt-12-RNAseq_result\4_Function\2_Group_Diff_Function\UP_DOWN\GO\NL-VS-L0.P.html#gene91) | response to stimulus | 3 (20%) | 585 (17.42%) | 0.499599 | 0.778955 |
| 92 | [GO:0019439](file:///E:\2018-7-3%E7%81%AB%E9%BE%99%E6%9E%9C%E8%BD%AC%E5%BD%95%E7%BB%84%E6%B5%8B%E5%BA%8F\%E5%AE%8C%E6%95%B4%E7%89%88%E6%95%B0%E6%8D%AE\GDR3855-Hylocereus_undulatus_Britt-12-RNAseq_result\4_Function\2_Group_Diff_Function\UP_DOWN\GO\NL-VS-L0.P.html#gene92) | aromatic compound catabolic process | 1 (6.67%) | 152 (4.53%) | 0.501473 | 0.778955 |
| 93 | [GO:0044270](file:///E:\2018-7-3%E7%81%AB%E9%BE%99%E6%9E%9C%E8%BD%AC%E5%BD%95%E7%BB%84%E6%B5%8B%E5%BA%8F\%E5%AE%8C%E6%95%B4%E7%89%88%E6%95%B0%E6%8D%AE\GDR3855-Hylocereus_undulatus_Britt-12-RNAseq_result\4_Function\2_Group_Diff_Function\UP_DOWN\GO\NL-VS-L0.P.html#gene93) | cellular nitrogen compound catabolic process | 1 (6.67%) | 154 (4.58%) | 0.506126 | 0.778955 |
| 94 | [GO:0046700](file:///E:\2018-7-3%E7%81%AB%E9%BE%99%E6%9E%9C%E8%BD%AC%E5%BD%95%E7%BB%84%E6%B5%8B%E5%BA%8F\%E5%AE%8C%E6%95%B4%E7%89%88%E6%95%B0%E6%8D%AE\GDR3855-Hylocereus_undulatus_Britt-12-RNAseq_result\4_Function\2_Group_Diff_Function\UP_DOWN\GO\NL-VS-L0.P.html#gene94) | heterocycle catabolic process | 1 (6.67%) | 154 (4.58%) | 0.506126 | 0.778955 |
| 95 | [GO:1901361](file:///E:\2018-7-3%E7%81%AB%E9%BE%99%E6%9E%9C%E8%BD%AC%E5%BD%95%E7%BB%84%E6%B5%8B%E5%BA%8F\%E5%AE%8C%E6%95%B4%E7%89%88%E6%95%B0%E6%8D%AE\GDR3855-Hylocereus_undulatus_Britt-12-RNAseq_result\4_Function\2_Group_Diff_Function\UP_DOWN\GO\NL-VS-L0.P.html#gene95) | organic cyclic compound catabolic process | 1 (6.67%) | 154 (4.58%) | 0.506126 | 0.778955 |
| 96 | [GO:0006351](file:///E:\2018-7-3%E7%81%AB%E9%BE%99%E6%9E%9C%E8%BD%AC%E5%BD%95%E7%BB%84%E6%B5%8B%E5%BA%8F\%E5%AE%8C%E6%95%B4%E7%89%88%E6%95%B0%E6%8D%AE\GDR3855-Hylocereus_undulatus_Britt-12-RNAseq_result\4_Function\2_Group_Diff_Function\UP_DOWN\GO\NL-VS-L0.P.html#gene96) | transcription, DNA-templated | 1 (6.67%) | 156 (4.64%) | 0.510739 | 0.778955 |
| 97 | [GO:0097659](file:///E:\2018-7-3%E7%81%AB%E9%BE%99%E6%9E%9C%E8%BD%AC%E5%BD%95%E7%BB%84%E6%B5%8B%E5%BA%8F\%E5%AE%8C%E6%95%B4%E7%89%88%E6%95%B0%E6%8D%AE\GDR3855-Hylocereus_undulatus_Britt-12-RNAseq_result\4_Function\2_Group_Diff_Function\UP_DOWN\GO\NL-VS-L0.P.html#gene97) | nucleic acid-templated transcription | 1 (6.67%) | 156 (4.64%) | 0.510739 | 0.778955 |
| 98 | [GO:0032774](file:///E:\2018-7-3%E7%81%AB%E9%BE%99%E6%9E%9C%E8%BD%AC%E5%BD%95%E7%BB%84%E6%B5%8B%E5%BA%8F\%E5%AE%8C%E6%95%B4%E7%89%88%E6%95%B0%E6%8D%AE\GDR3855-Hylocereus_undulatus_Britt-12-RNAseq_result\4_Function\2_Group_Diff_Function\UP_DOWN\GO\NL-VS-L0.P.html#gene98) | RNA biosynthetic process | 1 (6.67%) | 157 (4.67%) | 0.513030 | 0.778955 |
| 99 | [GO:0046483](file:///E:\2018-7-3%E7%81%AB%E9%BE%99%E6%9E%9C%E8%BD%AC%E5%BD%95%E7%BB%84%E6%B5%8B%E5%BA%8F\%E5%AE%8C%E6%95%B4%E7%89%88%E6%95%B0%E6%8D%AE\GDR3855-Hylocereus_undulatus_Britt-12-RNAseq_result\4_Function\2_Group_Diff_Function\UP_DOWN\GO\NL-VS-L0.P.html#gene99) | heterocycle metabolic process | 3 (20%) | 617 (18.37%) | 0.538745 | 0.778955 |
| 100 | [GO:0065007](file:///E:\2018-7-3%E7%81%AB%E9%BE%99%E6%9E%9C%E8%BD%AC%E5%BD%95%E7%BB%84%E6%B5%8B%E5%BA%8F\%E5%AE%8C%E6%95%B4%E7%89%88%E6%95%B0%E6%8D%AE\GDR3855-Hylocereus_undulatus_Britt-12-RNAseq_result\4_Function\2_Group_Diff_Function\UP_DOWN\GO\NL-VS-L0.P.html#gene100) | biological regulation | 2 (13.33%) | 400 (11.91%) | 0.548478 | 0.778955 |
| 101 | [GO:0044712](file:///E:\2018-7-3%E7%81%AB%E9%BE%99%E6%9E%9C%E8%BD%AC%E5%BD%95%E7%BB%84%E6%B5%8B%E5%BA%8F\%E5%AE%8C%E6%95%B4%E7%89%88%E6%95%B0%E6%8D%AE\GDR3855-Hylocereus_undulatus_Britt-12-RNAseq_result\4_Function\2_Group_Diff_Function\UP_DOWN\GO\NL-VS-L0.P.html#gene101) | single-organism catabolic process | 1 (6.67%) | 175 (5.21%) | 0.552598 | 0.778955 |
| 102 | [GO:0019538](file:///E:\2018-7-3%E7%81%AB%E9%BE%99%E6%9E%9C%E8%BD%AC%E5%BD%95%E7%BB%84%E6%B5%8B%E5%BA%8F\%E5%AE%8C%E6%95%B4%E7%89%88%E6%95%B0%E6%8D%AE\GDR3855-Hylocereus_undulatus_Britt-12-RNAseq_result\4_Function\2_Group_Diff_Function\UP_DOWN\GO\NL-VS-L0.P.html#gene102) | protein metabolic process | 3 (20%) | 630 (18.76%) | 0.554261 | 0.778955 |
| 103 | [GO:0006811](file:///E:\2018-7-3%E7%81%AB%E9%BE%99%E6%9E%9C%E8%BD%AC%E5%BD%95%E7%BB%84%E6%B5%8B%E5%BA%8F\%E5%AE%8C%E6%95%B4%E7%89%88%E6%95%B0%E6%8D%AE\GDR3855-Hylocereus_undulatus_Britt-12-RNAseq_result\4_Function\2_Group_Diff_Function\UP_DOWN\GO\NL-VS-L0.P.html#gene103) | ion transport | 1 (6.67%) | 176 (5.24%) | 0.554706 | 0.778955 |
| 104 | [GO:0044238](file:///E:\2018-7-3%E7%81%AB%E9%BE%99%E6%9E%9C%E8%BD%AC%E5%BD%95%E7%BB%84%E6%B5%8B%E5%BA%8F\%E5%AE%8C%E6%95%B4%E7%89%88%E6%95%B0%E6%8D%AE\GDR3855-Hylocereus_undulatus_Britt-12-RNAseq_result\4_Function\2_Group_Diff_Function\UP_DOWN\GO\NL-VS-L0.P.html#gene104) | primary metabolic process | 7 (46.67%) | 1522 (45.31%) | 0.557724 | 0.778955 |
| 105 | [GO:0006725](file:///E:\2018-7-3%E7%81%AB%E9%BE%99%E6%9E%9C%E8%BD%AC%E5%BD%95%E7%BB%84%E6%B5%8B%E5%BA%8F\%E5%AE%8C%E6%95%B4%E7%89%88%E6%95%B0%E6%8D%AE\GDR3855-Hylocereus_undulatus_Britt-12-RNAseq_result\4_Function\2_Group_Diff_Function\UP_DOWN\GO\NL-VS-L0.P.html#gene105) | cellular aromatic compound metabolic process | 3 (20%) | 634 (18.87%) | 0.558987 | 0.778955 |
| 106 | [GO:0009628](file:///E:\2018-7-3%E7%81%AB%E9%BE%99%E6%9E%9C%E8%BD%AC%E5%BD%95%E7%BB%84%E6%B5%8B%E5%BA%8F\%E5%AE%8C%E6%95%B4%E7%89%88%E6%95%B0%E6%8D%AE\GDR3855-Hylocereus_undulatus_Britt-12-RNAseq_result\4_Function\2_Group_Diff_Function\UP_DOWN\GO\NL-VS-L0.P.html#gene106) | response to abiotic stimulus | 1 (6.67%) | 180 (5.36%) | 0.563045 | 0.778955 |
| 107 | [GO:0009150](file:///E:\2018-7-3%E7%81%AB%E9%BE%99%E6%9E%9C%E8%BD%AC%E5%BD%95%E7%BB%84%E6%B5%8B%E5%BA%8F\%E5%AE%8C%E6%95%B4%E7%89%88%E6%95%B0%E6%8D%AE\GDR3855-Hylocereus_undulatus_Britt-12-RNAseq_result\4_Function\2_Group_Diff_Function\UP_DOWN\GO\NL-VS-L0.P.html#gene107) | purine ribonucleotide metabolic process | 1 (6.67%) | 184 (5.48%) | 0.571237 | 0.778955 |
| 108 | [GO:1901360](file:///E:\2018-7-3%E7%81%AB%E9%BE%99%E6%9E%9C%E8%BD%AC%E5%BD%95%E7%BB%84%E6%B5%8B%E5%BA%8F\%E5%AE%8C%E6%95%B4%E7%89%88%E6%95%B0%E6%8D%AE\GDR3855-Hylocereus_undulatus_Britt-12-RNAseq_result\4_Function\2_Group_Diff_Function\UP_DOWN\GO\NL-VS-L0.P.html#gene108) | organic cyclic compound metabolic process | 3 (20%) | 645 (19.2%) | 0.571858 | 0.778955 |
| 109 | [GO:0006163](file:///E:\2018-7-3%E7%81%AB%E9%BE%99%E6%9E%9C%E8%BD%AC%E5%BD%95%E7%BB%84%E6%B5%8B%E5%BA%8F\%E5%AE%8C%E6%95%B4%E7%89%88%E6%95%B0%E6%8D%AE\GDR3855-Hylocereus_undulatus_Britt-12-RNAseq_result\4_Function\2_Group_Diff_Function\UP_DOWN\GO\NL-VS-L0.P.html#gene109) | purine nucleotide metabolic process | 1 (6.67%) | 186 (5.54%) | 0.575280 | 0.778955 |
| 110 | [GO:0009259](file:///E:\2018-7-3%E7%81%AB%E9%BE%99%E6%9E%9C%E8%BD%AC%E5%BD%95%E7%BB%84%E6%B5%8B%E5%BA%8F\%E5%AE%8C%E6%95%B4%E7%89%88%E6%95%B0%E6%8D%AE\GDR3855-Hylocereus_undulatus_Britt-12-RNAseq_result\4_Function\2_Group_Diff_Function\UP_DOWN\GO\NL-VS-L0.P.html#gene110) | ribonucleotide metabolic process | 1 (6.67%) | 187 (5.57%) | 0.577288 | 0.778955 |
| 111 | [GO:0072521](file:///E:\2018-7-3%E7%81%AB%E9%BE%99%E6%9E%9C%E8%BD%AC%E5%BD%95%E7%BB%84%E6%B5%8B%E5%BA%8F\%E5%AE%8C%E6%95%B4%E7%89%88%E6%95%B0%E6%8D%AE\GDR3855-Hylocereus_undulatus_Britt-12-RNAseq_result\4_Function\2_Group_Diff_Function\UP_DOWN\GO\NL-VS-L0.P.html#gene111) | purine-containing compound metabolic process | 1 (6.67%) | 187 (5.57%) | 0.577288 | 0.778955 |
| 112 | [GO:0019752](file:///E:\2018-7-3%E7%81%AB%E9%BE%99%E6%9E%9C%E8%BD%AC%E5%BD%95%E7%BB%84%E6%B5%8B%E5%BA%8F\%E5%AE%8C%E6%95%B4%E7%89%88%E6%95%B0%E6%8D%AE\GDR3855-Hylocereus_undulatus_Britt-12-RNAseq_result\4_Function\2_Group_Diff_Function\UP_DOWN\GO\NL-VS-L0.P.html#gene112) | carboxylic acid metabolic process | 1 (6.67%) | 188 (5.6%) | 0.579287 | 0.778955 |
| 113 | [GO:0019693](file:///E:\2018-7-3%E7%81%AB%E9%BE%99%E6%9E%9C%E8%BD%AC%E5%BD%95%E7%BB%84%E6%B5%8B%E5%BA%8F\%E5%AE%8C%E6%95%B4%E7%89%88%E6%95%B0%E6%8D%AE\GDR3855-Hylocereus_undulatus_Britt-12-RNAseq_result\4_Function\2_Group_Diff_Function\UP_DOWN\GO\NL-VS-L0.P.html#gene113) | ribose phosphate metabolic process | 1 (6.67%) | 190 (5.66%) | 0.583258 | 0.778955 |
| 114 | [GO:0044763](file:///E:\2018-7-3%E7%81%AB%E9%BE%99%E6%9E%9C%E8%BD%AC%E5%BD%95%E7%BB%84%E6%B5%8B%E5%BA%8F\%E5%AE%8C%E6%95%B4%E7%89%88%E6%95%B0%E6%8D%AE\GDR3855-Hylocereus_undulatus_Britt-12-RNAseq_result\4_Function\2_Group_Diff_Function\UP_DOWN\GO\NL-VS-L0.P.html#gene114) | single-organism cellular process | 5 (33.33%) | 1107 (32.96%) | 0.584044 | 0.778955 |
| 115 | [GO:0044260](file:///E:\2018-7-3%E7%81%AB%E9%BE%99%E6%9E%9C%E8%BD%AC%E5%BD%95%E7%BB%84%E6%B5%8B%E5%BA%8F\%E5%AE%8C%E6%95%B4%E7%89%88%E6%95%B0%E6%8D%AE\GDR3855-Hylocereus_undulatus_Britt-12-RNAseq_result\4_Function\2_Group_Diff_Function\UP_DOWN\GO\NL-VS-L0.P.html#gene115) | cellular macromolecule metabolic process | 4 (26.67%) | 888 (26.44%) | 0.589738 | 0.778955 |
| 116 | [GO:0043436](file:///E:\2018-7-3%E7%81%AB%E9%BE%99%E6%9E%9C%E8%BD%AC%E5%BD%95%E7%BB%84%E6%B5%8B%E5%BA%8F\%E5%AE%8C%E6%95%B4%E7%89%88%E6%95%B0%E6%8D%AE\GDR3855-Hylocereus_undulatus_Britt-12-RNAseq_result\4_Function\2_Group_Diff_Function\UP_DOWN\GO\NL-VS-L0.P.html#gene116) | oxoacid metabolic process | 1 (6.67%) | 194 (5.78%) | 0.591096 | 0.778955 |
| 117 | [GO:0044710](file:///E:\2018-7-3%E7%81%AB%E9%BE%99%E6%9E%9C%E8%BD%AC%E5%BD%95%E7%BB%84%E6%B5%8B%E5%BA%8F\%E5%AE%8C%E6%95%B4%E7%89%88%E6%95%B0%E6%8D%AE\GDR3855-Hylocereus_undulatus_Britt-12-RNAseq_result\4_Function\2_Group_Diff_Function\UP_DOWN\GO\NL-VS-L0.P.html#gene117) | single-organism metabolic process | 5 (33.33%) | 1115 (33.19%) | 0.591772 | 0.778955 |
| 118 | [GO:0006082](file:///E:\2018-7-3%E7%81%AB%E9%BE%99%E6%9E%9C%E8%BD%AC%E5%BD%95%E7%BB%84%E6%B5%8B%E5%BA%8F\%E5%AE%8C%E6%95%B4%E7%89%88%E6%95%B0%E6%8D%AE\GDR3855-Hylocereus_undulatus_Britt-12-RNAseq_result\4_Function\2_Group_Diff_Function\UP_DOWN\GO\NL-VS-L0.P.html#gene118) | organic acid metabolic process | 1 (6.67%) | 195 (5.81%) | 0.593034 | 0.778955 |
| 119 | [GO:1901566](file:///E:\2018-7-3%E7%81%AB%E9%BE%99%E6%9E%9C%E8%BD%AC%E5%BD%95%E7%BB%84%E6%B5%8B%E5%BA%8F\%E5%AE%8C%E6%95%B4%E7%89%88%E6%95%B0%E6%8D%AE\GDR3855-Hylocereus_undulatus_Britt-12-RNAseq_result\4_Function\2_Group_Diff_Function\UP_DOWN\GO\NL-VS-L0.P.html#gene119) | organonitrogen compound biosynthetic process | 1 (6.67%) | 196 (5.84%) | 0.594964 | 0.778955 |
| 120 | [GO:1901576](file:///E:\2018-7-3%E7%81%AB%E9%BE%99%E6%9E%9C%E8%BD%AC%E5%BD%95%E7%BB%84%E6%B5%8B%E5%BA%8F\%E5%AE%8C%E6%95%B4%E7%89%88%E6%95%B0%E6%8D%AE\GDR3855-Hylocereus_undulatus_Britt-12-RNAseq_result\4_Function\2_Group_Diff_Function\UP_DOWN\GO\NL-VS-L0.P.html#gene120) | organic substance biosynthetic process | 2 (13.33%) | 435 (12.95%) | 0.596974 | 0.778955 |
| 121 | [GO:0034641](file:///E:\2018-7-3%E7%81%AB%E9%BE%99%E6%9E%9C%E8%BD%AC%E5%BD%95%E7%BB%84%E6%B5%8B%E5%BA%8F\%E5%AE%8C%E6%95%B4%E7%89%88%E6%95%B0%E6%8D%AE\GDR3855-Hylocereus_undulatus_Britt-12-RNAseq_result\4_Function\2_Group_Diff_Function\UP_DOWN\GO\NL-VS-L0.P.html#gene121) | cellular nitrogen compound metabolic process | 3 (20%) | 667 (19.86%) | 0.597036 | 0.778955 |
| 122 | [GO:0051716](file:///E:\2018-7-3%E7%81%AB%E9%BE%99%E6%9E%9C%E8%BD%AC%E5%BD%95%E7%BB%84%E6%B5%8B%E5%BA%8F\%E5%AE%8C%E6%95%B4%E7%89%88%E6%95%B0%E6%8D%AE\GDR3855-Hylocereus_undulatus_Britt-12-RNAseq_result\4_Function\2_Group_Diff_Function\UP_DOWN\GO\NL-VS-L0.P.html#gene122) | cellular response to stimulus | 1 (6.67%) | 211 (6.28%) | 0.622899 | 0.778955 |
| 123 | [GO:1901135](file:///E:\2018-7-3%E7%81%AB%E9%BE%99%E6%9E%9C%E8%BD%AC%E5%BD%95%E7%BB%84%E6%B5%8B%E5%BA%8F\%E5%AE%8C%E6%95%B4%E7%89%88%E6%95%B0%E6%8D%AE\GDR3855-Hylocereus_undulatus_Britt-12-RNAseq_result\4_Function\2_Group_Diff_Function\UP_DOWN\GO\NL-VS-L0.P.html#gene123) | carbohydrate derivative metabolic process | 1 (6.67%) | 211 (6.28%) | 0.622899 | 0.778955 |
| 124 | [GO:0044248](file:///E:\2018-7-3%E7%81%AB%E9%BE%99%E6%9E%9C%E8%BD%AC%E5%BD%95%E7%BB%84%E6%B5%8B%E5%BA%8F\%E5%AE%8C%E6%95%B4%E7%89%88%E6%95%B0%E6%8D%AE\GDR3855-Hylocereus_undulatus_Britt-12-RNAseq_result\4_Function\2_Group_Diff_Function\UP_DOWN\GO\NL-VS-L0.P.html#gene124) | cellular catabolic process | 1 (6.67%) | 212 (6.31%) | 0.624695 | 0.778955 |
| 125 | [GO:0034654](file:///E:\2018-7-3%E7%81%AB%E9%BE%99%E6%9E%9C%E8%BD%AC%E5%BD%95%E7%BB%84%E6%B5%8B%E5%BA%8F\%E5%AE%8C%E6%95%B4%E7%89%88%E6%95%B0%E6%8D%AE\GDR3855-Hylocereus_undulatus_Britt-12-RNAseq_result\4_Function\2_Group_Diff_Function\UP_DOWN\GO\NL-VS-L0.P.html#gene125) | nucleobase-containing compound biosynthetic process | 1 (6.67%) | 213 (6.34%) | 0.626484 | 0.778955 |
| 126 | [GO:0009117](file:///E:\2018-7-3%E7%81%AB%E9%BE%99%E6%9E%9C%E8%BD%AC%E5%BD%95%E7%BB%84%E6%B5%8B%E5%BA%8F\%E5%AE%8C%E6%95%B4%E7%89%88%E6%95%B0%E6%8D%AE\GDR3855-Hylocereus_undulatus_Britt-12-RNAseq_result\4_Function\2_Group_Diff_Function\UP_DOWN\GO\NL-VS-L0.P.html#gene126) | nucleotide metabolic process | 1 (6.67%) | 214 (6.37%) | 0.628265 | 0.778955 |
| 127 | [GO:0043170](file:///E:\2018-7-3%E7%81%AB%E9%BE%99%E6%9E%9C%E8%BD%AC%E5%BD%95%E7%BB%84%E6%B5%8B%E5%BA%8F\%E5%AE%8C%E6%95%B4%E7%89%88%E6%95%B0%E6%8D%AE\GDR3855-Hylocereus_undulatus_Britt-12-RNAseq_result\4_Function\2_Group_Diff_Function\UP_DOWN\GO\NL-VS-L0.P.html#gene127) | macromolecule metabolic process | 5 (33.33%) | 1160 (34.53%) | 0.634180 | 0.778955 |
| 128 | [GO:0006753](file:///E:\2018-7-3%E7%81%AB%E9%BE%99%E6%9E%9C%E8%BD%AC%E5%BD%95%E7%BB%84%E6%B5%8B%E5%BA%8F\%E5%AE%8C%E6%95%B4%E7%89%88%E6%95%B0%E6%8D%AE\GDR3855-Hylocereus_undulatus_Britt-12-RNAseq_result\4_Function\2_Group_Diff_Function\UP_DOWN\GO\NL-VS-L0.P.html#gene128) | nucleoside phosphate metabolic process | 1 (6.67%) | 218 (6.49%) | 0.635310 | 0.778955 |
| 129 | [GO:1901575](file:///E:\2018-7-3%E7%81%AB%E9%BE%99%E6%9E%9C%E8%BD%AC%E5%BD%95%E7%BB%84%E6%B5%8B%E5%BA%8F\%E5%AE%8C%E6%95%B4%E7%89%88%E6%95%B0%E6%8D%AE\GDR3855-Hylocereus_undulatus_Britt-12-RNAseq_result\4_Function\2_Group_Diff_Function\UP_DOWN\GO\NL-VS-L0.P.html#gene129) | organic substance catabolic process | 1 (6.67%) | 223 (6.64%) | 0.643941 | 0.778955 |
| 130 | [GO:0055086](file:///E:\2018-7-3%E7%81%AB%E9%BE%99%E6%9E%9C%E8%BD%AC%E5%BD%95%E7%BB%84%E6%B5%8B%E5%BA%8F\%E5%AE%8C%E6%95%B4%E7%89%88%E6%95%B0%E6%8D%AE\GDR3855-Hylocereus_undulatus_Britt-12-RNAseq_result\4_Function\2_Group_Diff_Function\UP_DOWN\GO\NL-VS-L0.P.html#gene130) | nucleobase-containing small molecule metabolic process | 1 (6.67%) | 226 (6.73%) | 0.649027 | 0.778955 |
| 131 | [GO:0042221](file:///E:\2018-7-3%E7%81%AB%E9%BE%99%E6%9E%9C%E8%BD%AC%E5%BD%95%E7%BB%84%E6%B5%8B%E5%BA%8F\%E5%AE%8C%E6%95%B4%E7%89%88%E6%95%B0%E6%8D%AE\GDR3855-Hylocereus_undulatus_Britt-12-RNAseq_result\4_Function\2_Group_Diff_Function\UP_DOWN\GO\NL-VS-L0.P.html#gene131) | response to chemical | 1 (6.67%) | 230 (6.85%) | 0.655704 | 0.778955 |
| 132 | [GO:0009056](file:///E:\2018-7-3%E7%81%AB%E9%BE%99%E6%9E%9C%E8%BD%AC%E5%BD%95%E7%BB%84%E6%B5%8B%E5%BA%8F\%E5%AE%8C%E6%95%B4%E7%89%88%E6%95%B0%E6%8D%AE\GDR3855-Hylocereus_undulatus_Britt-12-RNAseq_result\4_Function\2_Group_Diff_Function\UP_DOWN\GO\NL-VS-L0.P.html#gene132) | catabolic process | 1 (6.67%) | 231 (6.88%) | 0.657354 | 0.778955 |
| 133 | [GO:0044281](file:///E:\2018-7-3%E7%81%AB%E9%BE%99%E6%9E%9C%E8%BD%AC%E5%BD%95%E7%BB%84%E6%B5%8B%E5%BA%8F\%E5%AE%8C%E6%95%B4%E7%89%88%E6%95%B0%E6%8D%AE\GDR3855-Hylocereus_undulatus_Britt-12-RNAseq_result\4_Function\2_Group_Diff_Function\UP_DOWN\GO\NL-VS-L0.P.html#gene133) | small molecule metabolic process | 2 (13.33%) | 490 (14.59%) | 0.666044 | 0.778955 |
| 134 | [GO:0016043](file:///E:\2018-7-3%E7%81%AB%E9%BE%99%E6%9E%9C%E8%BD%AC%E5%BD%95%E7%BB%84%E6%B5%8B%E5%BA%8F\%E5%AE%8C%E6%95%B4%E7%89%88%E6%95%B0%E6%8D%AE\GDR3855-Hylocereus_undulatus_Britt-12-RNAseq_result\4_Function\2_Group_Diff_Function\UP_DOWN\GO\NL-VS-L0.P.html#gene134) | cellular component organization | 1 (6.67%) | 237 (7.06%) | 0.667103 | 0.778955 |
| 135 | [GO:0018130](file:///E:\2018-7-3%E7%81%AB%E9%BE%99%E6%9E%9C%E8%BD%AC%E5%BD%95%E7%BB%84%E6%B5%8B%E5%BA%8F\%E5%AE%8C%E6%95%B4%E7%89%88%E6%95%B0%E6%8D%AE\GDR3855-Hylocereus_undulatus_Britt-12-RNAseq_result\4_Function\2_Group_Diff_Function\UP_DOWN\GO\NL-VS-L0.P.html#gene135) | heterocycle biosynthetic process | 1 (6.67%) | 239 (7.12%) | 0.670295 | 0.778955 |
| 136 | [GO:0051234](file:///E:\2018-7-3%E7%81%AB%E9%BE%99%E6%9E%9C%E8%BD%AC%E5%BD%95%E7%BB%84%E6%B5%8B%E5%BA%8F\%E5%AE%8C%E6%95%B4%E7%89%88%E6%95%B0%E6%8D%AE\GDR3855-Hylocereus_undulatus_Britt-12-RNAseq_result\4_Function\2_Group_Diff_Function\UP_DOWN\GO\NL-VS-L0.P.html#gene136) | establishment of localization | 2 (13.33%) | 500 (14.89%) | 0.677649 | 0.778955 |
| 137 | [GO:0006807](file:///E:\2018-7-3%E7%81%AB%E9%BE%99%E6%9E%9C%E8%BD%AC%E5%BD%95%E7%BB%84%E6%B5%8B%E5%BA%8F\%E5%AE%8C%E6%95%B4%E7%89%88%E6%95%B0%E6%8D%AE\GDR3855-Hylocereus_undulatus_Britt-12-RNAseq_result\4_Function\2_Group_Diff_Function\UP_DOWN\GO\NL-VS-L0.P.html#gene137) | nitrogen compound metabolic process | 3 (20%) | 748 (22.27%) | 0.682550 | 0.778955 |
| 138 | [GO:0019438](file:///E:\2018-7-3%E7%81%AB%E9%BE%99%E6%9E%9C%E8%BD%AC%E5%BD%95%E7%BB%84%E6%B5%8B%E5%BA%8F\%E5%AE%8C%E6%95%B4%E7%89%88%E6%95%B0%E6%8D%AE\GDR3855-Hylocereus_undulatus_Britt-12-RNAseq_result\4_Function\2_Group_Diff_Function\UP_DOWN\GO\NL-VS-L0.P.html#gene138) | aromatic compound biosynthetic process | 1 (6.67%) | 248 (7.38%) | 0.684307 | 0.778955 |
| 139 | [GO:0051179](file:///E:\2018-7-3%E7%81%AB%E9%BE%99%E6%9E%9C%E8%BD%AC%E5%BD%95%E7%BB%84%E6%B5%8B%E5%BA%8F\%E5%AE%8C%E6%95%B4%E7%89%88%E6%95%B0%E6%8D%AE\GDR3855-Hylocereus_undulatus_Britt-12-RNAseq_result\4_Function\2_Group_Diff_Function\UP_DOWN\GO\NL-VS-L0.P.html#gene139) | localization | 2 (13.33%) | 513 (15.27%) | 0.692298 | 0.778955 |
| 140 | [GO:0019637](file:///E:\2018-7-3%E7%81%AB%E9%BE%99%E6%9E%9C%E8%BD%AC%E5%BD%95%E7%BB%84%E6%B5%8B%E5%BA%8F\%E5%AE%8C%E6%95%B4%E7%89%88%E6%95%B0%E6%8D%AE\GDR3855-Hylocereus_undulatus_Britt-12-RNAseq_result\4_Function\2_Group_Diff_Function\UP_DOWN\GO\NL-VS-L0.P.html#gene140) | organophosphate metabolic process | 1 (6.67%) | 256 (7.62%) | 0.696294 | 0.778955 |
| 141 | [GO:1901362](file:///E:\2018-7-3%E7%81%AB%E9%BE%99%E6%9E%9C%E8%BD%AC%E5%BD%95%E7%BB%84%E6%B5%8B%E5%BA%8F\%E5%AE%8C%E6%95%B4%E7%89%88%E6%95%B0%E6%8D%AE\GDR3855-Hylocereus_undulatus_Britt-12-RNAseq_result\4_Function\2_Group_Diff_Function\UP_DOWN\GO\NL-VS-L0.P.html#gene141) | organic cyclic compound biosynthetic process | 1 (6.67%) | 261 (7.77%) | 0.703569 | 0.778955 |
| 142 | [GO:0044237](file:///E:\2018-7-3%E7%81%AB%E9%BE%99%E6%9E%9C%E8%BD%AC%E5%BD%95%E7%BB%84%E6%B5%8B%E5%BA%8F\%E5%AE%8C%E6%95%B4%E7%89%88%E6%95%B0%E6%8D%AE\GDR3855-Hylocereus_undulatus_Britt-12-RNAseq_result\4_Function\2_Group_Diff_Function\UP_DOWN\GO\NL-VS-L0.P.html#gene142) | cellular metabolic process | 7 (46.67%) | 1688 (50.25%) | 0.703723 | 0.778955 |
| 143 | [GO:0016070](file:///E:\2018-7-3%E7%81%AB%E9%BE%99%E6%9E%9C%E8%BD%AC%E5%BD%95%E7%BB%84%E6%B5%8B%E5%BA%8F\%E5%AE%8C%E6%95%B4%E7%89%88%E6%95%B0%E6%8D%AE\GDR3855-Hylocereus_undulatus_Britt-12-RNAseq_result\4_Function\2_Group_Diff_Function\UP_DOWN\GO\NL-VS-L0.P.html#gene143) | RNA metabolic process | 1 (6.67%) | 262 (7.8%) | 0.705004 | 0.778955 |
| 144 | [GO:0071840](file:///E:\2018-7-3%E7%81%AB%E9%BE%99%E6%9E%9C%E8%BD%AC%E5%BD%95%E7%BB%84%E6%B5%8B%E5%BA%8F\%E5%AE%8C%E6%95%B4%E7%89%88%E6%95%B0%E6%8D%AE\GDR3855-Hylocereus_undulatus_Britt-12-RNAseq_result\4_Function\2_Group_Diff_Function\UP_DOWN\GO\NL-VS-L0.P.html#gene144) | cellular component organization or biogenesis | 1 (6.67%) | 267 (7.95%) | 0.712084 | 0.781314 |
| 145 | [GO:0044267](file:///E:\2018-7-3%E7%81%AB%E9%BE%99%E6%9E%9C%E8%BD%AC%E5%BD%95%E7%BB%84%E6%B5%8B%E5%BA%8F\%E5%AE%8C%E6%95%B4%E7%89%88%E6%95%B0%E6%8D%AE\GDR3855-Hylocereus_undulatus_Britt-12-RNAseq_result\4_Function\2_Group_Diff_Function\UP_DOWN\GO\NL-VS-L0.P.html#gene145) | cellular protein metabolic process | 2 (13.33%) | 541 (16.11%) | 0.722185 | 0.782314 |
| 146 | [GO:0044699](file:///E:\2018-7-3%E7%81%AB%E9%BE%99%E6%9E%9C%E8%BD%AC%E5%BD%95%E7%BB%84%E6%B5%8B%E5%BA%8F\%E5%AE%8C%E6%95%B4%E7%89%88%E6%95%B0%E6%8D%AE\GDR3855-Hylocereus_undulatus_Britt-12-RNAseq_result\4_Function\2_Group_Diff_Function\UP_DOWN\GO\NL-VS-L0.P.html#gene146) | single-organism process | 7 (46.67%) | 1712 (50.97%) | 0.722898 | 0.782314 |
| 147 | [GO:0009987](file:///E:\2018-7-3%E7%81%AB%E9%BE%99%E6%9E%9C%E8%BD%AC%E5%BD%95%E7%BB%84%E6%B5%8B%E5%BA%8F\%E5%AE%8C%E6%95%B4%E7%89%88%E6%95%B0%E6%8D%AE\GDR3855-Hylocereus_undulatus_Britt-12-RNAseq_result\4_Function\2_Group_Diff_Function\UP_DOWN\GO\NL-VS-L0.P.html#gene147) | cellular process | 9 (60%) | 2163 (64.39%) | 0.739039 | 0.794341 |
| 148 | [GO:0044765](file:///E:\2018-7-3%E7%81%AB%E9%BE%99%E6%9E%9C%E8%BD%AC%E5%BD%95%E7%BB%84%E6%B5%8B%E5%BA%8F\%E5%AE%8C%E6%95%B4%E7%89%88%E6%95%B0%E6%8D%AE\GDR3855-Hylocereus_undulatus_Britt-12-RNAseq_result\4_Function\2_Group_Diff_Function\UP_DOWN\GO\NL-VS-L0.P.html#gene148) | single-organism transport | 1 (6.67%) | 321 (9.56%) | 0.779084 | 0.831725 |
| 149 | [GO:1902578](file:///E:\2018-7-3%E7%81%AB%E9%BE%99%E6%9E%9C%E8%BD%AC%E5%BD%95%E7%BB%84%E6%B5%8B%E5%BA%8F\%E5%AE%8C%E6%95%B4%E7%89%88%E6%95%B0%E6%8D%AE\GDR3855-Hylocereus_undulatus_Britt-12-RNAseq_result\4_Function\2_Group_Diff_Function\UP_DOWN\GO\NL-VS-L0.P.html#gene149) | single-organism localization | 1 (6.67%) | 326 (9.71%) | 0.784487 | 0.831873 |
| 150 | [GO:0006464](file:///E:\2018-7-3%E7%81%AB%E9%BE%99%E6%9E%9C%E8%BD%AC%E5%BD%95%E7%BB%84%E6%B5%8B%E5%BA%8F\%E5%AE%8C%E6%95%B4%E7%89%88%E6%95%B0%E6%8D%AE\GDR3855-Hylocereus_undulatus_Britt-12-RNAseq_result\4_Function\2_Group_Diff_Function\UP_DOWN\GO\NL-VS-L0.P.html#gene150) | cellular protein modification process | 1 (6.67%) | 367 (10.93%) | 0.824368 | 0.865335 |
| 151 | [GO:0036211](file:///E:\2018-7-3%E7%81%AB%E9%BE%99%E6%9E%9C%E8%BD%AC%E5%BD%95%E7%BB%84%E6%B5%8B%E5%BA%8F\%E5%AE%8C%E6%95%B4%E7%89%88%E6%95%B0%E6%8D%AE\GDR3855-Hylocereus_undulatus_Britt-12-RNAseq_result\4_Function\2_Group_Diff_Function\UP_DOWN\GO\NL-VS-L0.P.html#gene151) | protein modification process | 1 (6.67%) | 370 (11.02%) | 0.826997 | 0.865335 |
| 152 | [GO:0043412](file:///E:\2018-7-3%E7%81%AB%E9%BE%99%E6%9E%9C%E8%BD%AC%E5%BD%95%E7%BB%84%E6%B5%8B%E5%BA%8F\%E5%AE%8C%E6%95%B4%E7%89%88%E6%95%B0%E6%8D%AE\GDR3855-Hylocereus_undulatus_Britt-12-RNAseq_result\4_Function\2_Group_Diff_Function\UP_DOWN\GO\NL-VS-L0.P.html#gene152) | macromolecule modification | 1 (6.67%) | 389 (11.58%) | 0.842814 | 0.876083 |
| 153 | [GO:0071704](file:///E:\2018-7-3%E7%81%AB%E9%BE%99%E6%9E%9C%E8%BD%AC%E5%BD%95%E7%BB%84%E6%B5%8B%E5%BA%8F\%E5%AE%8C%E6%95%B4%E7%89%88%E6%95%B0%E6%8D%AE\GDR3855-Hylocereus_undulatus_Britt-12-RNAseq_result\4_Function\2_Group_Diff_Function\UP_DOWN\GO\NL-VS-L0.P.html#gene153) | organic substance metabolic process | 6 (40%) | 1698 (50.55%) | 0.859537 | 0.881105 |
| 154 | [GO:1901564](file:///E:\2018-7-3%E7%81%AB%E9%BE%99%E6%9E%9C%E8%BD%AC%E5%BD%95%E7%BB%84%E6%B5%8B%E5%BA%8F\%E5%AE%8C%E6%95%B4%E7%89%88%E6%95%B0%E6%8D%AE\GDR3855-Hylocereus_undulatus_Britt-12-RNAseq_result\4_Function\2_Group_Diff_Function\UP_DOWN\GO\NL-VS-L0.P.html#gene154) | organonitrogen compound metabolic process | 1 (6.67%) | 417 (12.41%) | 0.863680 | 0.881105 |
| 155 | [GO:0006810](file:///E:\2018-7-3%E7%81%AB%E9%BE%99%E6%9E%9C%E8%BD%AC%E5%BD%95%E7%BB%84%E6%B5%8B%E5%BA%8F\%E5%AE%8C%E6%95%B4%E7%89%88%E6%95%B0%E6%8D%AE\GDR3855-Hylocereus_undulatus_Britt-12-RNAseq_result\4_Function\2_Group_Diff_Function\UP_DOWN\GO\NL-VS-L0.P.html#gene155) | transport | 1 (6.67%) | 418 (12.44%) | 0.864375 | 0.881105 |
| 156 | [GO:0006796](file:///E:\2018-7-3%E7%81%AB%E9%BE%99%E6%9E%9C%E8%BD%AC%E5%BD%95%E7%BB%84%E6%B5%8B%E5%BA%8F\%E5%AE%8C%E6%95%B4%E7%89%88%E6%95%B0%E6%8D%AE\GDR3855-Hylocereus_undulatus_Britt-12-RNAseq_result\4_Function\2_Group_Diff_Function\UP_DOWN\GO\NL-VS-L0.P.html#gene156) | phosphate-containing compound metabolic process | 1 (6.67%) | 665 (19.8%) | 0.963738 | 0.966282 |
| 157 | [GO:0008152](file:///E:\2018-7-3%E7%81%AB%E9%BE%99%E6%9E%9C%E8%BD%AC%E5%BD%95%E7%BB%84%E6%B5%8B%E5%BA%8F\%E5%AE%8C%E6%95%B4%E7%89%88%E6%95%B0%E6%8D%AE\GDR3855-Hylocereus_undulatus_Britt-12-RNAseq_result\4_Function\2_Group_Diff_Function\UP_DOWN\GO\NL-VS-L0.P.html#gene157) | metabolic process | 9 (60%) | 2599 (77.37%) | 0.965893 | 0.966282 |
| 158 | [GO:0006793](file:///E:\2018-7-3%E7%81%AB%E9%BE%99%E6%9E%9C%E8%BD%AC%E5%BD%95%E7%BB%84%E6%B5%8B%E5%BA%8F\%E5%AE%8C%E6%95%B4%E7%89%88%E6%95%B0%E6%8D%AE\GDR3855-Hylocereus_undulatus_Britt-12-RNAseq_result\4_Function\2_Group_Diff_Function\UP_DOWN\GO\NL-VS-L0.P.html#gene158) | phosphorus metabolic process | 1 (6.67%) | 678 (20.18%) | 0.966282 | 0.966282 |
