# Supplementary material for: Transcriptomic analysis of flower induction for long-day pitaya by supplementary lighting in short-day winter season
Source: BMC Genomics. 2020 Apr 29;21:329. doi: 10.1186/s12864-020-6726-6 (PMC7191803; doi:10.1186/s12864-020-6726-6)
Supplement: Supplementary file 6 — Additional file 6: Supplemental S6. NL-VS-L1 GO Enrichment (Molecular Function). [file 12864_2020_6726_MOESM6_ESM.docx]

| Supplemental S6 NL-VS-L1 GO Enrichment (Molecular Function) | | | | | | |
| --- | --- | --- | --- | --- | --- | --- |
| **#** | **GO ID** | **Description** | **GeneRatio (1220)** | **BgRatio (3432)** | **pvalue** | **p.adjust** |
| 1 | [GO:0016679](file:///E:\2018-7-3%E7%81%AB%E9%BE%99%E6%9E%9C%E8%BD%AC%E5%BD%95%E7%BB%84%E6%B5%8B%E5%BA%8F\%E5%AE%8C%E6%95%B4%E7%89%88%E6%95%B0%E6%8D%AE\GDR3855-Hylocereus_undulatus_Britt-12-RNAseq_result\4_Function\2_Group_Diff_Function\UP_DOWN\GO\NL-VS-L1.F.html#gene1) | oxidoreductase activity, acting on diphenols and related substances as donors | 10 (0.82%) | 11 (0.32%) | 0.000235 | 0.093694 |
| 2 | [GO:0016682](file:///E:\2018-7-3%E7%81%AB%E9%BE%99%E6%9E%9C%E8%BD%AC%E5%BD%95%E7%BB%84%E6%B5%8B%E5%BA%8F\%E5%AE%8C%E6%95%B4%E7%89%88%E6%95%B0%E6%8D%AE\GDR3855-Hylocereus_undulatus_Britt-12-RNAseq_result\4_Function\2_Group_Diff_Function\UP_DOWN\GO\NL-VS-L1.F.html#gene2) | oxidoreductase activity, acting on diphenols and related substances as donors, oxygen as acceptor | 9 (0.74%) | 10 (0.29%) | 0.000606 | 0.120920 |
| 3 | [GO:0016627](file:///E:\2018-7-3%E7%81%AB%E9%BE%99%E6%9E%9C%E8%BD%AC%E5%BD%95%E7%BB%84%E6%B5%8B%E5%BA%8F\%E5%AE%8C%E6%95%B4%E7%89%88%E6%95%B0%E6%8D%AE\GDR3855-Hylocereus_undulatus_Britt-12-RNAseq_result\4_Function\2_Group_Diff_Function\UP_DOWN\GO\NL-VS-L1.F.html#gene3) | oxidoreductase activity, acting on the CH-CH group of donors | 57 (4.67%) | 119 (3.47%) | 0.003229 | 0.189771 |
| 4 | [GO:0005216](file:///E:\2018-7-3%E7%81%AB%E9%BE%99%E6%9E%9C%E8%BD%AC%E5%BD%95%E7%BB%84%E6%B5%8B%E5%BA%8F\%E5%AE%8C%E6%95%B4%E7%89%88%E6%95%B0%E6%8D%AE\GDR3855-Hylocereus_undulatus_Britt-12-RNAseq_result\4_Function\2_Group_Diff_Function\UP_DOWN\GO\NL-VS-L1.F.html#gene4) | ion channel activity | 12 (0.98%) | 17 (0.5%) | 0.003428 | 0.189771 |
| 5 | [GO:0015267](file:///E:\2018-7-3%E7%81%AB%E9%BE%99%E6%9E%9C%E8%BD%AC%E5%BD%95%E7%BB%84%E6%B5%8B%E5%BA%8F\%E5%AE%8C%E6%95%B4%E7%89%88%E6%95%B0%E6%8D%AE\GDR3855-Hylocereus_undulatus_Britt-12-RNAseq_result\4_Function\2_Group_Diff_Function\UP_DOWN\GO\NL-VS-L1.F.html#gene5) | channel activity | 12 (0.98%) | 17 (0.5%) | 0.003428 | 0.189771 |
| 6 | [GO:0022803](file:///E:\2018-7-3%E7%81%AB%E9%BE%99%E6%9E%9C%E8%BD%AC%E5%BD%95%E7%BB%84%E6%B5%8B%E5%BA%8F\%E5%AE%8C%E6%95%B4%E7%89%88%E6%95%B0%E6%8D%AE\GDR3855-Hylocereus_undulatus_Britt-12-RNAseq_result\4_Function\2_Group_Diff_Function\UP_DOWN\GO\NL-VS-L1.F.html#gene6) | passive transmembrane transporter activity | 12 (0.98%) | 17 (0.5%) | 0.003428 | 0.189771 |
| 7 | [GO:0022838](file:///E:\2018-7-3%E7%81%AB%E9%BE%99%E6%9E%9C%E8%BD%AC%E5%BD%95%E7%BB%84%E6%B5%8B%E5%BA%8F\%E5%AE%8C%E6%95%B4%E7%89%88%E6%95%B0%E6%8D%AE\GDR3855-Hylocereus_undulatus_Britt-12-RNAseq_result\4_Function\2_Group_Diff_Function\UP_DOWN\GO\NL-VS-L1.F.html#gene7) | substrate-specific channel activity | 12 (0.98%) | 17 (0.5%) | 0.003428 | 0.189771 |
| 8 | [GO:0016628](file:///E:\2018-7-3%E7%81%AB%E9%BE%99%E6%9E%9C%E8%BD%AC%E5%BD%95%E7%BB%84%E6%B5%8B%E5%BA%8F\%E5%AE%8C%E6%95%B4%E7%89%88%E6%95%B0%E6%8D%AE\GDR3855-Hylocereus_undulatus_Britt-12-RNAseq_result\4_Function\2_Group_Diff_Function\UP_DOWN\GO\NL-VS-L1.F.html#gene8) | oxidoreductase activity, acting on the CH-CH group of donors, NAD or NADP as acceptor | 53 (4.34%) | 110 (3.21%) | 0.003805 | 0.189771 |
| 9 | [GO:0008509](file:///E:\2018-7-3%E7%81%AB%E9%BE%99%E6%9E%9C%E8%BD%AC%E5%BD%95%E7%BB%84%E6%B5%8B%E5%BA%8F\%E5%AE%8C%E6%95%B4%E7%89%88%E6%95%B0%E6%8D%AE\GDR3855-Hylocereus_undulatus_Britt-12-RNAseq_result\4_Function\2_Group_Diff_Function\UP_DOWN\GO\NL-VS-L1.F.html#gene9) | anion transmembrane transporter activity | 18 (1.48%) | 30 (0.87%) | 0.005260 | 0.233180 |
| 10 | [GO:0015103](file:///E:\2018-7-3%E7%81%AB%E9%BE%99%E6%9E%9C%E8%BD%AC%E5%BD%95%E7%BB%84%E6%B5%8B%E5%BA%8F\%E5%AE%8C%E6%95%B4%E7%89%88%E6%95%B0%E6%8D%AE\GDR3855-Hylocereus_undulatus_Britt-12-RNAseq_result\4_Function\2_Group_Diff_Function\UP_DOWN\GO\NL-VS-L1.F.html#gene10) | inorganic anion transmembrane transporter activity | 9 (0.74%) | 12 (0.35%) | 0.006235 | 0.248759 |
| 11 | [GO:0015926](file:///E:\2018-7-3%E7%81%AB%E9%BE%99%E6%9E%9C%E8%BD%AC%E5%BD%95%E7%BB%84%E6%B5%8B%E5%BA%8F\%E5%AE%8C%E6%95%B4%E7%89%88%E6%95%B0%E6%8D%AE\GDR3855-Hylocereus_undulatus_Britt-12-RNAseq_result\4_Function\2_Group_Diff_Function\UP_DOWN\GO\NL-VS-L1.F.html#gene11) | glucosidase activity | 12 (0.98%) | 19 (0.55%) | 0.012886 | 0.373590 |
| 12 | [GO:0046873](file:///E:\2018-7-3%E7%81%AB%E9%BE%99%E6%9E%9C%E8%BD%AC%E5%BD%95%E7%BB%84%E6%B5%8B%E5%BA%8F\%E5%AE%8C%E6%95%B4%E7%89%88%E6%95%B0%E6%8D%AE\GDR3855-Hylocereus_undulatus_Britt-12-RNAseq_result\4_Function\2_Group_Diff_Function\UP_DOWN\GO\NL-VS-L1.F.html#gene12) | metal ion transmembrane transporter activity | 12 (0.98%) | 19 (0.55%) | 0.012886 | 0.373590 |
| 13 | [GO:0016758](file:///E:\2018-7-3%E7%81%AB%E9%BE%99%E6%9E%9C%E8%BD%AC%E5%BD%95%E7%BB%84%E6%B5%8B%E5%BA%8F\%E5%AE%8C%E6%95%B4%E7%89%88%E6%95%B0%E6%8D%AE\GDR3855-Hylocereus_undulatus_Britt-12-RNAseq_result\4_Function\2_Group_Diff_Function\UP_DOWN\GO\NL-VS-L1.F.html#gene13) | transferase activity, transferring hexosyl groups | 38 (3.11%) | 79 (2.3%) | 0.013679 | 0.373590 |
| 14 | [GO:0004553](file:///E:\2018-7-3%E7%81%AB%E9%BE%99%E6%9E%9C%E8%BD%AC%E5%BD%95%E7%BB%84%E6%B5%8B%E5%BA%8F\%E5%AE%8C%E6%95%B4%E7%89%88%E6%95%B0%E6%8D%AE\GDR3855-Hylocereus_undulatus_Britt-12-RNAseq_result\4_Function\2_Group_Diff_Function\UP_DOWN\GO\NL-VS-L1.F.html#gene14) | hydrolase activity, hydrolyzing O-glycosyl compounds | 27 (2.21%) | 53 (1.54%) | 0.014707 | 0.373590 |
| 15 | [GO:0005253](file:///E:\2018-7-3%E7%81%AB%E9%BE%99%E6%9E%9C%E8%BD%AC%E5%BD%95%E7%BB%84%E6%B5%8B%E5%BA%8F\%E5%AE%8C%E6%95%B4%E7%89%88%E6%95%B0%E6%8D%AE\GDR3855-Hylocereus_undulatus_Britt-12-RNAseq_result\4_Function\2_Group_Diff_Function\UP_DOWN\GO\NL-VS-L1.F.html#gene15) | anion channel activity | 4 (0.33%) | 4 (0.12%) | 0.015917 | 0.373590 |
| 16 | [GO:0005254](file:///E:\2018-7-3%E7%81%AB%E9%BE%99%E6%9E%9C%E8%BD%AC%E5%BD%95%E7%BB%84%E6%B5%8B%E5%BA%8F\%E5%AE%8C%E6%95%B4%E7%89%88%E6%95%B0%E6%8D%AE\GDR3855-Hylocereus_undulatus_Britt-12-RNAseq_result\4_Function\2_Group_Diff_Function\UP_DOWN\GO\NL-VS-L1.F.html#gene16) | chloride channel activity | 4 (0.33%) | 4 (0.12%) | 0.015917 | 0.373590 |
| 17 | [GO:0015108](file:///E:\2018-7-3%E7%81%AB%E9%BE%99%E6%9E%9C%E8%BD%AC%E5%BD%95%E7%BB%84%E6%B5%8B%E5%BA%8F\%E5%AE%8C%E6%95%B4%E7%89%88%E6%95%B0%E6%8D%AE\GDR3855-Hylocereus_undulatus_Britt-12-RNAseq_result\4_Function\2_Group_Diff_Function\UP_DOWN\GO\NL-VS-L1.F.html#gene17) | chloride transmembrane transporter activity | 4 (0.33%) | 4 (0.12%) | 0.015917 | 0.373590 |
| 18 | [GO:0016798](file:///E:\2018-7-3%E7%81%AB%E9%BE%99%E6%9E%9C%E8%BD%AC%E5%BD%95%E7%BB%84%E6%B5%8B%E5%BA%8F\%E5%AE%8C%E6%95%B4%E7%89%88%E6%95%B0%E6%8D%AE\GDR3855-Hylocereus_undulatus_Britt-12-RNAseq_result\4_Function\2_Group_Diff_Function\UP_DOWN\GO\NL-VS-L1.F.html#gene18) | hydrolase activity, acting on glycosyl bonds | 36 (2.95%) | 76 (2.21%) | 0.021262 | 0.458546 |
| 19 | [GO:0016757](file:///E:\2018-7-3%E7%81%AB%E9%BE%99%E6%9E%9C%E8%BD%AC%E5%BD%95%E7%BB%84%E6%B5%8B%E5%BA%8F\%E5%AE%8C%E6%95%B4%E7%89%88%E6%95%B0%E6%8D%AE\GDR3855-Hylocereus_undulatus_Britt-12-RNAseq_result\4_Function\2_Group_Diff_Function\UP_DOWN\GO\NL-VS-L1.F.html#gene19) | transferase activity, transferring glycosyl groups | 54 (4.43%) | 122 (3.55%) | 0.026710 | 0.458546 |
| 20 | [GO:0016701](file:///E:\2018-7-3%E7%81%AB%E9%BE%99%E6%9E%9C%E8%BD%AC%E5%BD%95%E7%BB%84%E6%B5%8B%E5%BA%8F\%E5%AE%8C%E6%95%B4%E7%89%88%E6%95%B0%E6%8D%AE\GDR3855-Hylocereus_undulatus_Britt-12-RNAseq_result\4_Function\2_Group_Diff_Function\UP_DOWN\GO\NL-VS-L1.F.html#gene20) | oxidoreductase activity, acting on single donors with incorporation of molecular oxygen | 9 (0.74%) | 14 (0.41%) | 0.026780 | 0.458546 |
| 21 | [GO:0005372](file:///E:\2018-7-3%E7%81%AB%E9%BE%99%E6%9E%9C%E8%BD%AC%E5%BD%95%E7%BB%84%E6%B5%8B%E5%BA%8F\%E5%AE%8C%E6%95%B4%E7%89%88%E6%95%B0%E6%8D%AE\GDR3855-Hylocereus_undulatus_Britt-12-RNAseq_result\4_Function\2_Group_Diff_Function\UP_DOWN\GO\NL-VS-L1.F.html#gene21) | water transmembrane transporter activity | 6 (0.49%) | 8 (0.23%) | 0.027278 | 0.458546 |
| 22 | [GO:0022836](file:///E:\2018-7-3%E7%81%AB%E9%BE%99%E6%9E%9C%E8%BD%AC%E5%BD%95%E7%BB%84%E6%B5%8B%E5%BA%8F\%E5%AE%8C%E6%95%B4%E7%89%88%E6%95%B0%E6%8D%AE\GDR3855-Hylocereus_undulatus_Britt-12-RNAseq_result\4_Function\2_Group_Diff_Function\UP_DOWN\GO\NL-VS-L1.F.html#gene22) | gated channel activity | 6 (0.49%) | 8 (0.23%) | 0.027278 | 0.458546 |
| 23 | [GO:0046912](file:///E:\2018-7-3%E7%81%AB%E9%BE%99%E6%9E%9C%E8%BD%AC%E5%BD%95%E7%BB%84%E6%B5%8B%E5%BA%8F\%E5%AE%8C%E6%95%B4%E7%89%88%E6%95%B0%E6%8D%AE\GDR3855-Hylocereus_undulatus_Britt-12-RNAseq_result\4_Function\2_Group_Diff_Function\UP_DOWN\GO\NL-VS-L1.F.html#gene23) | transferase activity, transferring acyl groups, acyl groups converted into alkyl on transfer | 6 (0.49%) | 8 (0.23%) | 0.027278 | 0.458546 |
| 24 | [GO:0016615](file:///E:\2018-7-3%E7%81%AB%E9%BE%99%E6%9E%9C%E8%BD%AC%E5%BD%95%E7%BB%84%E6%B5%8B%E5%BA%8F\%E5%AE%8C%E6%95%B4%E7%89%88%E6%95%B0%E6%8D%AE\GDR3855-Hylocereus_undulatus_Britt-12-RNAseq_result\4_Function\2_Group_Diff_Function\UP_DOWN\GO\NL-VS-L1.F.html#gene24) | malate dehydrogenase activity | 8 (0.66%) | 12 (0.35%) | 0.027874 | 0.458546 |
| 25 | [GO:0015631](file:///E:\2018-7-3%E7%81%AB%E9%BE%99%E6%9E%9C%E8%BD%AC%E5%BD%95%E7%BB%84%E6%B5%8B%E5%BA%8F\%E5%AE%8C%E6%95%B4%E7%89%88%E6%95%B0%E6%8D%AE\GDR3855-Hylocereus_undulatus_Britt-12-RNAseq_result\4_Function\2_Group_Diff_Function\UP_DOWN\GO\NL-VS-L1.F.html#gene25) | tubulin binding | 14 (1.15%) | 25 (0.73%) | 0.028731 | 0.458546 |
| 26 | [GO:0046983](file:///E:\2018-7-3%E7%81%AB%E9%BE%99%E6%9E%9C%E8%BD%AC%E5%BD%95%E7%BB%84%E6%B5%8B%E5%BA%8F\%E5%AE%8C%E6%95%B4%E7%89%88%E6%95%B0%E6%8D%AE\GDR3855-Hylocereus_undulatus_Britt-12-RNAseq_result\4_Function\2_Group_Diff_Function\UP_DOWN\GO\NL-VS-L1.F.html#gene26) | protein dimerization activity | 20 (1.64%) | 39 (1.14%) | 0.030924 | 0.474571 |
| 27 | [GO:0005249](file:///E:\2018-7-3%E7%81%AB%E9%BE%99%E6%9E%9C%E8%BD%AC%E5%BD%95%E7%BB%84%E6%B5%8B%E5%BA%8F\%E5%AE%8C%E6%95%B4%E7%89%88%E6%95%B0%E6%8D%AE\GDR3855-Hylocereus_undulatus_Britt-12-RNAseq_result\4_Function\2_Group_Diff_Function\UP_DOWN\GO\NL-VS-L1.F.html#gene27) | voltage-gated potassium channel activity | 3 (0.25%) | 3 (0.09%) | 0.044849 | 0.594385 |
| 28 | [GO:0005267](file:///E:\2018-7-3%E7%81%AB%E9%BE%99%E6%9E%9C%E8%BD%AC%E5%BD%95%E7%BB%84%E6%B5%8B%E5%BA%8F\%E5%AE%8C%E6%95%B4%E7%89%88%E6%95%B0%E6%8D%AE\GDR3855-Hylocereus_undulatus_Britt-12-RNAseq_result\4_Function\2_Group_Diff_Function\UP_DOWN\GO\NL-VS-L1.F.html#gene28) | potassium channel activity | 3 (0.25%) | 3 (0.09%) | 0.044849 | 0.594385 |
| 29 | [GO:0015079](file:///E:\2018-7-3%E7%81%AB%E9%BE%99%E6%9E%9C%E8%BD%AC%E5%BD%95%E7%BB%84%E6%B5%8B%E5%BA%8F\%E5%AE%8C%E6%95%B4%E7%89%88%E6%95%B0%E6%8D%AE\GDR3855-Hylocereus_undulatus_Britt-12-RNAseq_result\4_Function\2_Group_Diff_Function\UP_DOWN\GO\NL-VS-L1.F.html#gene29) | potassium ion transmembrane transporter activity | 3 (0.25%) | 3 (0.09%) | 0.044849 | 0.594385 |
| 30 | [GO:0022843](file:///E:\2018-7-3%E7%81%AB%E9%BE%99%E6%9E%9C%E8%BD%AC%E5%BD%95%E7%BB%84%E6%B5%8B%E5%BA%8F\%E5%AE%8C%E6%95%B4%E7%89%88%E6%95%B0%E6%8D%AE\GDR3855-Hylocereus_undulatus_Britt-12-RNAseq_result\4_Function\2_Group_Diff_Function\UP_DOWN\GO\NL-VS-L1.F.html#gene30) | voltage-gated cation channel activity | 3 (0.25%) | 3 (0.09%) | 0.044849 | 0.594385 |
| 31 | [GO:0019203](file:///E:\2018-7-3%E7%81%AB%E9%BE%99%E6%9E%9C%E8%BD%AC%E5%BD%95%E7%BB%84%E6%B5%8B%E5%BA%8F\%E5%AE%8C%E6%95%B4%E7%89%88%E6%95%B0%E6%8D%AE\GDR3855-Hylocereus_undulatus_Britt-12-RNAseq_result\4_Function\2_Group_Diff_Function\UP_DOWN\GO\NL-VS-L1.F.html#gene31) | carbohydrate phosphatase activity | 9 (0.74%) | 15 (0.44%) | 0.046180 | 0.594385 |
| 32 | [GO:0005244](file:///E:\2018-7-3%E7%81%AB%E9%BE%99%E6%9E%9C%E8%BD%AC%E5%BD%95%E7%BB%84%E6%B5%8B%E5%BA%8F\%E5%AE%8C%E6%95%B4%E7%89%88%E6%95%B0%E6%8D%AE\GDR3855-Hylocereus_undulatus_Britt-12-RNAseq_result\4_Function\2_Group_Diff_Function\UP_DOWN\GO\NL-VS-L1.F.html#gene32) | voltage-gated ion channel activity | 4 (0.33%) | 5 (0.15%) | 0.057002 | 0.668932 |
| 33 | [GO:0016638](file:///E:\2018-7-3%E7%81%AB%E9%BE%99%E6%9E%9C%E8%BD%AC%E5%BD%95%E7%BB%84%E6%B5%8B%E5%BA%8F\%E5%AE%8C%E6%95%B4%E7%89%88%E6%95%B0%E6%8D%AE\GDR3855-Hylocereus_undulatus_Britt-12-RNAseq_result\4_Function\2_Group_Diff_Function\UP_DOWN\GO\NL-VS-L1.F.html#gene33) | oxidoreductase activity, acting on the CH-NH2 group of donors | 4 (0.33%) | 5 (0.15%) | 0.057002 | 0.668932 |
| 34 | [GO:0022832](file:///E:\2018-7-3%E7%81%AB%E9%BE%99%E6%9E%9C%E8%BD%AC%E5%BD%95%E7%BB%84%E6%B5%8B%E5%BA%8F\%E5%AE%8C%E6%95%B4%E7%89%88%E6%95%B0%E6%8D%AE\GDR3855-Hylocereus_undulatus_Britt-12-RNAseq_result\4_Function\2_Group_Diff_Function\UP_DOWN\GO\NL-VS-L1.F.html#gene34) | voltage-gated channel activity | 4 (0.33%) | 5 (0.15%) | 0.057002 | 0.668932 |
| 35 | [GO:0008092](file:///E:\2018-7-3%E7%81%AB%E9%BE%99%E6%9E%9C%E8%BD%AC%E5%BD%95%E7%BB%84%E6%B5%8B%E5%BA%8F\%E5%AE%8C%E6%95%B4%E7%89%88%E6%95%B0%E6%8D%AE\GDR3855-Hylocereus_undulatus_Britt-12-RNAseq_result\4_Function\2_Group_Diff_Function\UP_DOWN\GO\NL-VS-L1.F.html#gene35) | cytoskeletal protein binding | 19 (1.56%) | 39 (1.14%) | 0.061409 | 0.700067 |
| 36 | [GO:0016491](file:///E:\2018-7-3%E7%81%AB%E9%BE%99%E6%9E%9C%E8%BD%AC%E5%BD%95%E7%BB%84%E6%B5%8B%E5%BA%8F\%E5%AE%8C%E6%95%B4%E7%89%88%E6%95%B0%E6%8D%AE\GDR3855-Hylocereus_undulatus_Britt-12-RNAseq_result\4_Function\2_Group_Diff_Function\UP_DOWN\GO\NL-VS-L1.F.html#gene36) | oxidoreductase activity | 204 (16.72%) | 532 (15.5%) | 0.078617 | 0.861069 |
| 37 | [GO:0004683](file:///E:\2018-7-3%E7%81%AB%E9%BE%99%E6%9E%9C%E8%BD%AC%E5%BD%95%E7%BB%84%E6%B5%8B%E5%BA%8F\%E5%AE%8C%E6%95%B4%E7%89%88%E6%95%B0%E6%8D%AE\GDR3855-Hylocereus_undulatus_Britt-12-RNAseq_result\4_Function\2_Group_Diff_Function\UP_DOWN\GO\NL-VS-L1.F.html#gene37) | calmodulin-dependent protein kinase activity | 7 (0.57%) | 12 (0.35%) | 0.090899 | 0.861069 |
| 38 | [GO:0004470](file:///E:\2018-7-3%E7%81%AB%E9%BE%99%E6%9E%9C%E8%BD%AC%E5%BD%95%E7%BB%84%E6%B5%8B%E5%BA%8F\%E5%AE%8C%E6%95%B4%E7%89%88%E6%95%B0%E6%8D%AE\GDR3855-Hylocereus_undulatus_Britt-12-RNAseq_result\4_Function\2_Group_Diff_Function\UP_DOWN\GO\NL-VS-L1.F.html#gene38) | malic enzyme activity | 5 (0.41%) | 8 (0.23%) | 0.112266 | 0.861069 |
| 39 | [GO:0015925](file:///E:\2018-7-3%E7%81%AB%E9%BE%99%E6%9E%9C%E8%BD%AC%E5%BD%95%E7%BB%84%E6%B5%8B%E5%BA%8F\%E5%AE%8C%E6%95%B4%E7%89%88%E6%95%B0%E6%8D%AE\GDR3855-Hylocereus_undulatus_Britt-12-RNAseq_result\4_Function\2_Group_Diff_Function\UP_DOWN\GO\NL-VS-L1.F.html#gene39) | galactosidase activity | 5 (0.41%) | 8 (0.23%) | 0.112266 | 0.861069 |
| 40 | [GO:0003774](file:///E:\2018-7-3%E7%81%AB%E9%BE%99%E6%9E%9C%E8%BD%AC%E5%BD%95%E7%BB%84%E6%B5%8B%E5%BA%8F\%E5%AE%8C%E6%95%B4%E7%89%88%E6%95%B0%E6%8D%AE\GDR3855-Hylocereus_undulatus_Britt-12-RNAseq_result\4_Function\2_Group_Diff_Function\UP_DOWN\GO\NL-VS-L1.F.html#gene40) | motor activity | 11 (0.9%) | 22 (0.64%) | 0.116833 | 0.861069 |
| 41 | [GO:0004713](file:///E:\2018-7-3%E7%81%AB%E9%BE%99%E6%9E%9C%E8%BD%AC%E5%BD%95%E7%BB%84%E6%B5%8B%E5%BA%8F\%E5%AE%8C%E6%95%B4%E7%89%88%E6%95%B0%E6%8D%AE\GDR3855-Hylocereus_undulatus_Britt-12-RNAseq_result\4_Function\2_Group_Diff_Function\UP_DOWN\GO\NL-VS-L1.F.html#gene41) | protein tyrosine kinase activity | 11 (0.9%) | 22 (0.64%) | 0.116833 | 0.861069 |
| 42 | [GO:0005261](file:///E:\2018-7-3%E7%81%AB%E9%BE%99%E6%9E%9C%E8%BD%AC%E5%BD%95%E7%BB%84%E6%B5%8B%E5%BA%8F\%E5%AE%8C%E6%95%B4%E7%89%88%E6%95%B0%E6%8D%AE\GDR3855-Hylocereus_undulatus_Britt-12-RNAseq_result\4_Function\2_Group_Diff_Function\UP_DOWN\GO\NL-VS-L1.F.html#gene42) | cation channel activity | 4 (0.33%) | 6 (0.17%) | 0.123268 | 0.861069 |
| 43 | [GO:0004557](file:///E:\2018-7-3%E7%81%AB%E9%BE%99%E6%9E%9C%E8%BD%AC%E5%BD%95%E7%BB%84%E6%B5%8B%E5%BA%8F\%E5%AE%8C%E6%95%B4%E7%89%88%E6%95%B0%E6%8D%AE\GDR3855-Hylocereus_undulatus_Britt-12-RNAseq_result\4_Function\2_Group_Diff_Function\UP_DOWN\GO\NL-VS-L1.F.html#gene43) | alpha-galactosidase activity | 2 (0.16%) | 2 (0.06%) | 0.126298 | 0.861069 |
| 44 | [GO:0004576](file:///E:\2018-7-3%E7%81%AB%E9%BE%99%E6%9E%9C%E8%BD%AC%E5%BD%95%E7%BB%84%E6%B5%8B%E5%BA%8F\%E5%AE%8C%E6%95%B4%E7%89%88%E6%95%B0%E6%8D%AE\GDR3855-Hylocereus_undulatus_Britt-12-RNAseq_result\4_Function\2_Group_Diff_Function\UP_DOWN\GO\NL-VS-L1.F.html#gene44) | oligosaccharyl transferase activity | 2 (0.16%) | 2 (0.06%) | 0.126298 | 0.861069 |
| 45 | [GO:0004645](file:///E:\2018-7-3%E7%81%AB%E9%BE%99%E6%9E%9C%E8%BD%AC%E5%BD%95%E7%BB%84%E6%B5%8B%E5%BA%8F\%E5%AE%8C%E6%95%B4%E7%89%88%E6%95%B0%E6%8D%AE\GDR3855-Hylocereus_undulatus_Britt-12-RNAseq_result\4_Function\2_Group_Diff_Function\UP_DOWN\GO\NL-VS-L1.F.html#gene45) | phosphorylase activity | 2 (0.16%) | 2 (0.06%) | 0.126298 | 0.861069 |
| 46 | [GO:0005310](file:///E:\2018-7-3%E7%81%AB%E9%BE%99%E6%9E%9C%E8%BD%AC%E5%BD%95%E7%BB%84%E6%B5%8B%E5%BA%8F\%E5%AE%8C%E6%95%B4%E7%89%88%E6%95%B0%E6%8D%AE\GDR3855-Hylocereus_undulatus_Britt-12-RNAseq_result\4_Function\2_Group_Diff_Function\UP_DOWN\GO\NL-VS-L1.F.html#gene46) | dicarboxylic acid transmembrane transporter activity | 2 (0.16%) | 2 (0.06%) | 0.126298 | 0.861069 |
| 47 | [GO:0005375](file:///E:\2018-7-3%E7%81%AB%E9%BE%99%E6%9E%9C%E8%BD%AC%E5%BD%95%E7%BB%84%E6%B5%8B%E5%BA%8F\%E5%AE%8C%E6%95%B4%E7%89%88%E6%95%B0%E6%8D%AE\GDR3855-Hylocereus_undulatus_Britt-12-RNAseq_result\4_Function\2_Group_Diff_Function\UP_DOWN\GO\NL-VS-L1.F.html#gene47) | copper ion transmembrane transporter activity | 2 (0.16%) | 2 (0.06%) | 0.126298 | 0.861069 |
| 48 | [GO:0008080](file:///E:\2018-7-3%E7%81%AB%E9%BE%99%E6%9E%9C%E8%BD%AC%E5%BD%95%E7%BB%84%E6%B5%8B%E5%BA%8F\%E5%AE%8C%E6%95%B4%E7%89%88%E6%95%B0%E6%8D%AE\GDR3855-Hylocereus_undulatus_Britt-12-RNAseq_result\4_Function\2_Group_Diff_Function\UP_DOWN\GO\NL-VS-L1.F.html#gene48) | N-acetyltransferase activity | 2 (0.16%) | 2 (0.06%) | 0.126298 | 0.861069 |
| 49 | [GO:0008172](file:///E:\2018-7-3%E7%81%AB%E9%BE%99%E6%9E%9C%E8%BD%AC%E5%BD%95%E7%BB%84%E6%B5%8B%E5%BA%8F\%E5%AE%8C%E6%95%B4%E7%89%88%E6%95%B0%E6%8D%AE\GDR3855-Hylocereus_undulatus_Britt-12-RNAseq_result\4_Function\2_Group_Diff_Function\UP_DOWN\GO\NL-VS-L1.F.html#gene49) | S-methyltransferase activity | 2 (0.16%) | 2 (0.06%) | 0.126298 | 0.861069 |
| 50 | [GO:0015085](file:///E:\2018-7-3%E7%81%AB%E9%BE%99%E6%9E%9C%E8%BD%AC%E5%BD%95%E7%BB%84%E6%B5%8B%E5%BA%8F\%E5%AE%8C%E6%95%B4%E7%89%88%E6%95%B0%E6%8D%AE\GDR3855-Hylocereus_undulatus_Britt-12-RNAseq_result\4_Function\2_Group_Diff_Function\UP_DOWN\GO\NL-VS-L1.F.html#gene50) | calcium ion transmembrane transporter activity | 2 (0.16%) | 2 (0.06%) | 0.126298 | 0.861069 |
| 51 | [GO:0015140](file:///E:\2018-7-3%E7%81%AB%E9%BE%99%E6%9E%9C%E8%BD%AC%E5%BD%95%E7%BB%84%E6%B5%8B%E5%BA%8F\%E5%AE%8C%E6%95%B4%E7%89%88%E6%95%B0%E6%8D%AE\GDR3855-Hylocereus_undulatus_Britt-12-RNAseq_result\4_Function\2_Group_Diff_Function\UP_DOWN\GO\NL-VS-L1.F.html#gene51) | malate transmembrane transporter activity | 2 (0.16%) | 2 (0.06%) | 0.126298 | 0.861069 |
| 52 | [GO:0015556](file:///E:\2018-7-3%E7%81%AB%E9%BE%99%E6%9E%9C%E8%BD%AC%E5%BD%95%E7%BB%84%E6%B5%8B%E5%BA%8F\%E5%AE%8C%E6%95%B4%E7%89%88%E6%95%B0%E6%8D%AE\GDR3855-Hylocereus_undulatus_Britt-12-RNAseq_result\4_Function\2_Group_Diff_Function\UP_DOWN\GO\NL-VS-L1.F.html#gene52) | C4-dicarboxylate transmembrane transporter activity | 2 (0.16%) | 2 (0.06%) | 0.126298 | 0.861069 |
| 53 | [GO:0015562](file:///E:\2018-7-3%E7%81%AB%E9%BE%99%E6%9E%9C%E8%BD%AC%E5%BD%95%E7%BB%84%E6%B5%8B%E5%BA%8F\%E5%AE%8C%E6%95%B4%E7%89%88%E6%95%B0%E6%8D%AE\GDR3855-Hylocereus_undulatus_Britt-12-RNAseq_result\4_Function\2_Group_Diff_Function\UP_DOWN\GO\NL-VS-L1.F.html#gene53) | efflux transmembrane transporter activity | 2 (0.16%) | 2 (0.06%) | 0.126298 | 0.861069 |
| 54 | [GO:0016421](file:///E:\2018-7-3%E7%81%AB%E9%BE%99%E6%9E%9C%E8%BD%AC%E5%BD%95%E7%BB%84%E6%B5%8B%E5%BA%8F\%E5%AE%8C%E6%95%B4%E7%89%88%E6%95%B0%E6%8D%AE\GDR3855-Hylocereus_undulatus_Britt-12-RNAseq_result\4_Function\2_Group_Diff_Function\UP_DOWN\GO\NL-VS-L1.F.html#gene54) | CoA carboxylase activity | 2 (0.16%) | 2 (0.06%) | 0.126298 | 0.861069 |
| 55 | [GO:0016885](file:///E:\2018-7-3%E7%81%AB%E9%BE%99%E6%9E%9C%E8%BD%AC%E5%BD%95%E7%BB%84%E6%B5%8B%E5%BA%8F\%E5%AE%8C%E6%95%B4%E7%89%88%E6%95%B0%E6%8D%AE\GDR3855-Hylocereus_undulatus_Britt-12-RNAseq_result\4_Function\2_Group_Diff_Function\UP_DOWN\GO\NL-VS-L1.F.html#gene55) | ligase activity, forming carbon-carbon bonds | 2 (0.16%) | 2 (0.06%) | 0.126298 | 0.861069 |
| 56 | [GO:0043682](file:///E:\2018-7-3%E7%81%AB%E9%BE%99%E6%9E%9C%E8%BD%AC%E5%BD%95%E7%BB%84%E6%B5%8B%E5%BA%8F\%E5%AE%8C%E6%95%B4%E7%89%88%E6%95%B0%E6%8D%AE\GDR3855-Hylocereus_undulatus_Britt-12-RNAseq_result\4_Function\2_Group_Diff_Function\UP_DOWN\GO\NL-VS-L1.F.html#gene56) | copper-transporting ATPase activity | 2 (0.16%) | 2 (0.06%) | 0.126298 | 0.861069 |
| 57 | [GO:0072509](file:///E:\2018-7-3%E7%81%AB%E9%BE%99%E6%9E%9C%E8%BD%AC%E5%BD%95%E7%BB%84%E6%B5%8B%E5%BA%8F\%E5%AE%8C%E6%95%B4%E7%89%88%E6%95%B0%E6%8D%AE\GDR3855-Hylocereus_undulatus_Britt-12-RNAseq_result\4_Function\2_Group_Diff_Function\UP_DOWN\GO\NL-VS-L1.F.html#gene57) | divalent inorganic cation transmembrane transporter activity | 2 (0.16%) | 2 (0.06%) | 0.126298 | 0.861069 |
| 58 | [GO:0004558](file:///E:\2018-7-3%E7%81%AB%E9%BE%99%E6%9E%9C%E8%BD%AC%E5%BD%95%E7%BB%84%E6%B5%8B%E5%BA%8F\%E5%AE%8C%E6%95%B4%E7%89%88%E6%95%B0%E6%8D%AE\GDR3855-Hylocereus_undulatus_Britt-12-RNAseq_result\4_Function\2_Group_Diff_Function\UP_DOWN\GO\NL-VS-L1.F.html#gene58) | alpha-1,4-glucosidase activity | 3 (0.25%) | 4 (0.12%) | 0.131642 | 0.861069 |
| 59 | [GO:0004857](file:///E:\2018-7-3%E7%81%AB%E9%BE%99%E6%9E%9C%E8%BD%AC%E5%BD%95%E7%BB%84%E6%B5%8B%E5%BA%8F\%E5%AE%8C%E6%95%B4%E7%89%88%E6%95%B0%E6%8D%AE\GDR3855-Hylocereus_undulatus_Britt-12-RNAseq_result\4_Function\2_Group_Diff_Function\UP_DOWN\GO\NL-VS-L1.F.html#gene59) | enzyme inhibitor activity | 3 (0.25%) | 4 (0.12%) | 0.131642 | 0.861069 |
| 60 | [GO:0046915](file:///E:\2018-7-3%E7%81%AB%E9%BE%99%E6%9E%9C%E8%BD%AC%E5%BD%95%E7%BB%84%E6%B5%8B%E5%BA%8F\%E5%AE%8C%E6%95%B4%E7%89%88%E6%95%B0%E6%8D%AE\GDR3855-Hylocereus_undulatus_Britt-12-RNAseq_result\4_Function\2_Group_Diff_Function\UP_DOWN\GO\NL-VS-L1.F.html#gene60) | transition metal ion transmembrane transporter activity | 3 (0.25%) | 4 (0.12%) | 0.131642 | 0.861069 |
| 61 | [GO:0090599](file:///E:\2018-7-3%E7%81%AB%E9%BE%99%E6%9E%9C%E8%BD%AC%E5%BD%95%E7%BB%84%E6%B5%8B%E5%BA%8F\%E5%AE%8C%E6%95%B4%E7%89%88%E6%95%B0%E6%8D%AE\GDR3855-Hylocereus_undulatus_Britt-12-RNAseq_result\4_Function\2_Group_Diff_Function\UP_DOWN\GO\NL-VS-L1.F.html#gene61) | alpha-glucosidase activity | 3 (0.25%) | 4 (0.12%) | 0.131642 | 0.861069 |
| 62 | [GO:0005215](file:///E:\2018-7-3%E7%81%AB%E9%BE%99%E6%9E%9C%E8%BD%AC%E5%BD%95%E7%BB%84%E6%B5%8B%E5%BA%8F\%E5%AE%8C%E6%95%B4%E7%89%88%E6%95%B0%E6%8D%AE\GDR3855-Hylocereus_undulatus_Britt-12-RNAseq_result\4_Function\2_Group_Diff_Function\UP_DOWN\GO\NL-VS-L1.F.html#gene62) | transporter activity | 103 (8.44%) | 265 (7.72%) | 0.134030 | 0.862516 |
| 63 | [GO:0016831](file:///E:\2018-7-3%E7%81%AB%E9%BE%99%E6%9E%9C%E8%BD%AC%E5%BD%95%E7%BB%84%E6%B5%8B%E5%BA%8F\%E5%AE%8C%E6%95%B4%E7%89%88%E6%95%B0%E6%8D%AE\GDR3855-Hylocereus_undulatus_Britt-12-RNAseq_result\4_Function\2_Group_Diff_Function\UP_DOWN\GO\NL-VS-L1.F.html#gene63) | carboxy-lyase activity | 12 (0.98%) | 25 (0.73%) | 0.137146 | 0.862516 |
| 64 | [GO:0017076](file:///E:\2018-7-3%E7%81%AB%E9%BE%99%E6%9E%9C%E8%BD%AC%E5%BD%95%E7%BB%84%E6%B5%8B%E5%BA%8F\%E5%AE%8C%E6%95%B4%E7%89%88%E6%95%B0%E6%8D%AE\GDR3855-Hylocereus_undulatus_Britt-12-RNAseq_result\4_Function\2_Group_Diff_Function\UP_DOWN\GO\NL-VS-L1.F.html#gene64) | purine nucleotide binding | 7 (0.57%) | 13 (0.38%) | 0.138348 | 0.862516 |
| 65 | [GO:0022857](file:///E:\2018-7-3%E7%81%AB%E9%BE%99%E6%9E%9C%E8%BD%AC%E5%BD%95%E7%BB%84%E6%B5%8B%E5%BA%8F\%E5%AE%8C%E6%95%B4%E7%89%88%E6%95%B0%E6%8D%AE\GDR3855-Hylocereus_undulatus_Britt-12-RNAseq_result\4_Function\2_Group_Diff_Function\UP_DOWN\GO\NL-VS-L1.F.html#gene65) | transmembrane transporter activity | 95 (7.79%) | 244 (7.11%) | 0.140847 | 0.864581 |
| 66 | [GO:0008514](file:///E:\2018-7-3%E7%81%AB%E9%BE%99%E6%9E%9C%E8%BD%AC%E5%BD%95%E7%BB%84%E6%B5%8B%E5%BA%8F\%E5%AE%8C%E6%95%B4%E7%89%88%E6%95%B0%E6%8D%AE\GDR3855-Hylocereus_undulatus_Britt-12-RNAseq_result\4_Function\2_Group_Diff_Function\UP_DOWN\GO\NL-VS-L1.F.html#gene66) | organic anion transmembrane transporter activity | 8 (0.66%) | 16 (0.47%) | 0.170675 | 0.880068 |
| 67 | [GO:0003824](file:///E:\2018-7-3%E7%81%AB%E9%BE%99%E6%9E%9C%E8%BD%AC%E5%BD%95%E7%BB%84%E6%B5%8B%E5%BA%8F\%E5%AE%8C%E6%95%B4%E7%89%88%E6%95%B0%E6%8D%AE\GDR3855-Hylocereus_undulatus_Britt-12-RNAseq_result\4_Function\2_Group_Diff_Function\UP_DOWN\GO\NL-VS-L1.F.html#gene67) | catalytic activity | 963 (78.93%) | 2677 (78%) | 0.174414 | 0.880068 |
| 68 | [GO:0016866](file:///E:\2018-7-3%E7%81%AB%E9%BE%99%E6%9E%9C%E8%BD%AC%E5%BD%95%E7%BB%84%E6%B5%8B%E5%BA%8F\%E5%AE%8C%E6%95%B4%E7%89%88%E6%95%B0%E6%8D%AE\GDR3855-Hylocereus_undulatus_Britt-12-RNAseq_result\4_Function\2_Group_Diff_Function\UP_DOWN\GO\NL-VS-L1.F.html#gene68) | intramolecular transferase activity | 5 (0.41%) | 9 (0.26%) | 0.180804 | 0.880068 |
| 69 | [GO:0005515](file:///E:\2018-7-3%E7%81%AB%E9%BE%99%E6%9E%9C%E8%BD%AC%E5%BD%95%E7%BB%84%E6%B5%8B%E5%BA%8F\%E5%AE%8C%E6%95%B4%E7%89%88%E6%95%B0%E6%8D%AE\GDR3855-Hylocereus_undulatus_Britt-12-RNAseq_result\4_Function\2_Group_Diff_Function\UP_DOWN\GO\NL-VS-L1.F.html#gene69) | protein binding | 80 (6.56%) | 207 (6.03%) | 0.187395 | 0.880068 |
| 70 | [GO:0005342](file:///E:\2018-7-3%E7%81%AB%E9%BE%99%E6%9E%9C%E8%BD%AC%E5%BD%95%E7%BB%84%E6%B5%8B%E5%BA%8F\%E5%AE%8C%E6%95%B4%E7%89%88%E6%95%B0%E6%8D%AE\GDR3855-Hylocereus_undulatus_Britt-12-RNAseq_result\4_Function\2_Group_Diff_Function\UP_DOWN\GO\NL-VS-L1.F.html#gene70) | organic acid transmembrane transporter activity | 7 (0.57%) | 14 (0.41%) | 0.195206 | 0.880068 |
| 71 | [GO:0046943](file:///E:\2018-7-3%E7%81%AB%E9%BE%99%E6%9E%9C%E8%BD%AC%E5%BD%95%E7%BB%84%E6%B5%8B%E5%BA%8F\%E5%AE%8C%E6%95%B4%E7%89%88%E6%95%B0%E6%8D%AE\GDR3855-Hylocereus_undulatus_Britt-12-RNAseq_result\4_Function\2_Group_Diff_Function\UP_DOWN\GO\NL-VS-L1.F.html#gene71) | carboxylic acid transmembrane transporter activity | 7 (0.57%) | 14 (0.41%) | 0.195206 | 0.880068 |
| 72 | [GO:0052689](file:///E:\2018-7-3%E7%81%AB%E9%BE%99%E6%9E%9C%E8%BD%AC%E5%BD%95%E7%BB%84%E6%B5%8B%E5%BA%8F\%E5%AE%8C%E6%95%B4%E7%89%88%E6%95%B0%E6%8D%AE\GDR3855-Hylocereus_undulatus_Britt-12-RNAseq_result\4_Function\2_Group_Diff_Function\UP_DOWN\GO\NL-VS-L1.F.html#gene72) | carboxylic ester hydrolase activity | 7 (0.57%) | 14 (0.41%) | 0.195206 | 0.880068 |
| 73 | [GO:0015297](file:///E:\2018-7-3%E7%81%AB%E9%BE%99%E6%9E%9C%E8%BD%AC%E5%BD%95%E7%BB%84%E6%B5%8B%E5%BA%8F\%E5%AE%8C%E6%95%B4%E7%89%88%E6%95%B0%E6%8D%AE\GDR3855-Hylocereus_undulatus_Britt-12-RNAseq_result\4_Function\2_Group_Diff_Function\UP_DOWN\GO\NL-VS-L1.F.html#gene73) | antiporter activity | 4 (0.33%) | 7 (0.2%) | 0.208760 | 0.880068 |
| 74 | [GO:0015298](file:///E:\2018-7-3%E7%81%AB%E9%BE%99%E6%9E%9C%E8%BD%AC%E5%BD%95%E7%BB%84%E6%B5%8B%E5%BA%8F\%E5%AE%8C%E6%95%B4%E7%89%88%E6%95%B0%E6%8D%AE\GDR3855-Hylocereus_undulatus_Britt-12-RNAseq_result\4_Function\2_Group_Diff_Function\UP_DOWN\GO\NL-VS-L1.F.html#gene74) | solute:cation antiporter activity | 4 (0.33%) | 7 (0.2%) | 0.208760 | 0.880068 |
| 75 | [GO:0015491](file:///E:\2018-7-3%E7%81%AB%E9%BE%99%E6%9E%9C%E8%BD%AC%E5%BD%95%E7%BB%84%E6%B5%8B%E5%BA%8F\%E5%AE%8C%E6%95%B4%E7%89%88%E6%95%B0%E6%8D%AE\GDR3855-Hylocereus_undulatus_Britt-12-RNAseq_result\4_Function\2_Group_Diff_Function\UP_DOWN\GO\NL-VS-L1.F.html#gene75) | cation:cation antiporter activity | 4 (0.33%) | 7 (0.2%) | 0.208760 | 0.880068 |
| 76 | [GO:0016703](file:///E:\2018-7-3%E7%81%AB%E9%BE%99%E6%9E%9C%E8%BD%AC%E5%BD%95%E7%BB%84%E6%B5%8B%E5%BA%8F\%E5%AE%8C%E6%95%B4%E7%89%88%E6%95%B0%E6%8D%AE\GDR3855-Hylocereus_undulatus_Britt-12-RNAseq_result\4_Function\2_Group_Diff_Function\UP_DOWN\GO\NL-VS-L1.F.html#gene76) | oxidoreductase activity, acting on single donors with incorporation of molecular oxygen, incorporation of one atom of oxygen (internal monooxygenases or internal mixed function oxidases) | 4 (0.33%) | 7 (0.2%) | 0.208760 | 0.880068 |
| 77 | [GO:0099516](file:///E:\2018-7-3%E7%81%AB%E9%BE%99%E6%9E%9C%E8%BD%AC%E5%BD%95%E7%BB%84%E6%B5%8B%E5%BA%8F\%E5%AE%8C%E6%95%B4%E7%89%88%E6%95%B0%E6%8D%AE\GDR3855-Hylocereus_undulatus_Britt-12-RNAseq_result\4_Function\2_Group_Diff_Function\UP_DOWN\GO\NL-VS-L1.F.html#gene77) | ion antiporter activity | 4 (0.33%) | 7 (0.2%) | 0.208760 | 0.880068 |
| 78 | [GO:0016772](file:///E:\2018-7-3%E7%81%AB%E9%BE%99%E6%9E%9C%E8%BD%AC%E5%BD%95%E7%BB%84%E6%B5%8B%E5%BA%8F\%E5%AE%8C%E6%95%B4%E7%89%88%E6%95%B0%E6%8D%AE\GDR3855-Hylocereus_undulatus_Britt-12-RNAseq_result\4_Function\2_Group_Diff_Function\UP_DOWN\GO\NL-VS-L1.F.html#gene78) | transferase activity, transferring phosphorus-containing groups | 200 (16.39%) | 538 (15.68%) | 0.208801 | 0.880068 |
| 79 | [GO:0000166](file:///E:\2018-7-3%E7%81%AB%E9%BE%99%E6%9E%9C%E8%BD%AC%E5%BD%95%E7%BB%84%E6%B5%8B%E5%BA%8F\%E5%AE%8C%E6%95%B4%E7%89%88%E6%95%B0%E6%8D%AE\GDR3855-Hylocereus_undulatus_Britt-12-RNAseq_result\4_Function\2_Group_Diff_Function\UP_DOWN\GO\NL-VS-L1.F.html#gene79) | nucleotide binding | 37 (3.03%) | 93 (2.71%) | 0.223703 | 0.880068 |
| 80 | [GO:1901265](file:///E:\2018-7-3%E7%81%AB%E9%BE%99%E6%9E%9C%E8%BD%AC%E5%BD%95%E7%BB%84%E6%B5%8B%E5%BA%8F\%E5%AE%8C%E6%95%B4%E7%89%88%E6%95%B0%E6%8D%AE\GDR3855-Hylocereus_undulatus_Britt-12-RNAseq_result\4_Function\2_Group_Diff_Function\UP_DOWN\GO\NL-VS-L1.F.html#gene80) | nucleoside phosphate binding | 37 (3.03%) | 93 (2.71%) | 0.223703 | 0.880068 |
| 81 | [GO:0030234](file:///E:\2018-7-3%E7%81%AB%E9%BE%99%E6%9E%9C%E8%BD%AC%E5%BD%95%E7%BB%84%E6%B5%8B%E5%BA%8F\%E5%AE%8C%E6%95%B4%E7%89%88%E6%95%B0%E6%8D%AE\GDR3855-Hylocereus_undulatus_Britt-12-RNAseq_result\4_Function\2_Group_Diff_Function\UP_DOWN\GO\NL-VS-L1.F.html#gene81) | enzyme regulator activity | 6 (0.49%) | 12 (0.35%) | 0.224571 | 0.880068 |
| 82 | [GO:0098772](file:///E:\2018-7-3%E7%81%AB%E9%BE%99%E6%9E%9C%E8%BD%AC%E5%BD%95%E7%BB%84%E6%B5%8B%E5%BA%8F\%E5%AE%8C%E6%95%B4%E7%89%88%E6%95%B0%E6%8D%AE\GDR3855-Hylocereus_undulatus_Britt-12-RNAseq_result\4_Function\2_Group_Diff_Function\UP_DOWN\GO\NL-VS-L1.F.html#gene82) | molecular function regulator | 6 (0.49%) | 12 (0.35%) | 0.224571 | 0.880068 |
| 83 | [GO:0015662](file:///E:\2018-7-3%E7%81%AB%E9%BE%99%E6%9E%9C%E8%BD%AC%E5%BD%95%E7%BB%84%E6%B5%8B%E5%BA%8F\%E5%AE%8C%E6%95%B4%E7%89%88%E6%95%B0%E6%8D%AE\GDR3855-Hylocereus_undulatus_Britt-12-RNAseq_result\4_Function\2_Group_Diff_Function\UP_DOWN\GO\NL-VS-L1.F.html#gene83) | ATPase activity, coupled to transmembrane movement of ions, phosphorylative mechanism | 3 (0.25%) | 5 (0.15%) | 0.243603 | 0.880068 |
| 84 | [GO:0016857](file:///E:\2018-7-3%E7%81%AB%E9%BE%99%E6%9E%9C%E8%BD%AC%E5%BD%95%E7%BB%84%E6%B5%8B%E5%BA%8F\%E5%AE%8C%E6%95%B4%E7%89%88%E6%95%B0%E6%8D%AE\GDR3855-Hylocereus_undulatus_Britt-12-RNAseq_result\4_Function\2_Group_Diff_Function\UP_DOWN\GO\NL-VS-L1.F.html#gene84) | racemase and epimerase activity, acting on carbohydrates and derivatives | 3 (0.25%) | 5 (0.15%) | 0.243603 | 0.880068 |
| 85 | [GO:0031420](file:///E:\2018-7-3%E7%81%AB%E9%BE%99%E6%9E%9C%E8%BD%AC%E5%BD%95%E7%BB%84%E6%B5%8B%E5%BA%8F\%E5%AE%8C%E6%95%B4%E7%89%88%E6%95%B0%E6%8D%AE\GDR3855-Hylocereus_undulatus_Britt-12-RNAseq_result\4_Function\2_Group_Diff_Function\UP_DOWN\GO\NL-VS-L1.F.html#gene85) | alkali metal ion binding | 3 (0.25%) | 5 (0.15%) | 0.243603 | 0.880068 |
| 86 | [GO:0022892](file:///E:\2018-7-3%E7%81%AB%E9%BE%99%E6%9E%9C%E8%BD%AC%E5%BD%95%E7%BB%84%E6%B5%8B%E5%BA%8F\%E5%AE%8C%E6%95%B4%E7%89%88%E6%95%B0%E6%8D%AE\GDR3855-Hylocereus_undulatus_Britt-12-RNAseq_result\4_Function\2_Group_Diff_Function\UP_DOWN\GO\NL-VS-L1.F.html#gene86) | substrate-specific transporter activity | 65 (5.33%) | 170 (4.95%) | 0.250627 | 0.880068 |
| 87 | [GO:0016773](file:///E:\2018-7-3%E7%81%AB%E9%BE%99%E6%9E%9C%E8%BD%AC%E5%BD%95%E7%BB%84%E6%B5%8B%E5%BA%8F\%E5%AE%8C%E6%95%B4%E7%89%88%E6%95%B0%E6%8D%AE\GDR3855-Hylocereus_undulatus_Britt-12-RNAseq_result\4_Function\2_Group_Diff_Function\UP_DOWN\GO\NL-VS-L1.F.html#gene87) | phosphotransferase activity, alcohol group as acceptor | 112 (9.18%) | 300 (8.74%) | 0.268861 | 0.880068 |
| 88 | [GO:0004672](file:///E:\2018-7-3%E7%81%AB%E9%BE%99%E6%9E%9C%E8%BD%AC%E5%BD%95%E7%BB%84%E6%B5%8B%E5%BA%8F\%E5%AE%8C%E6%95%B4%E7%89%88%E6%95%B0%E6%8D%AE\GDR3855-Hylocereus_undulatus_Britt-12-RNAseq_result\4_Function\2_Group_Diff_Function\UP_DOWN\GO\NL-VS-L1.F.html#gene88) | protein kinase activity | 105 (8.61%) | 281 (8.19%) | 0.273264 | 0.880068 |
| 89 | [GO:0005198](file:///E:\2018-7-3%E7%81%AB%E9%BE%99%E6%9E%9C%E8%BD%AC%E5%BD%95%E7%BB%84%E6%B5%8B%E5%BA%8F\%E5%AE%8C%E6%95%B4%E7%89%88%E6%95%B0%E6%8D%AE\GDR3855-Hylocereus_undulatus_Britt-12-RNAseq_result\4_Function\2_Group_Diff_Function\UP_DOWN\GO\NL-VS-L1.F.html#gene89) | structural molecule activity | 18 (1.48%) | 44 (1.28%) | 0.274789 | 0.880068 |
| 90 | [GO:0016740](file:///E:\2018-7-3%E7%81%AB%E9%BE%99%E6%9E%9C%E8%BD%AC%E5%BD%95%E7%BB%84%E6%B5%8B%E5%BA%8F\%E5%AE%8C%E6%95%B4%E7%89%88%E6%95%B0%E6%8D%AE\GDR3855-Hylocereus_undulatus_Britt-12-RNAseq_result\4_Function\2_Group_Diff_Function\UP_DOWN\GO\NL-VS-L1.F.html#gene90) | transferase activity | 354 (29.02%) | 974 (28.38%) | 0.282405 | 0.880068 |
| 91 | [GO:0016620](file:///E:\2018-7-3%E7%81%AB%E9%BE%99%E6%9E%9C%E8%BD%AC%E5%BD%95%E7%BB%84%E6%B5%8B%E5%BA%8F\%E5%AE%8C%E6%95%B4%E7%89%88%E6%95%B0%E6%8D%AE\GDR3855-Hylocereus_undulatus_Britt-12-RNAseq_result\4_Function\2_Group_Diff_Function\UP_DOWN\GO\NL-VS-L1.F.html#gene91) | oxidoreductase activity, acting on the aldehyde or oxo group of donors, NAD or NADP as acceptor | 8 (0.66%) | 18 (0.52%) | 0.288102 | 0.880068 |
| 92 | [GO:0005230](file:///E:\2018-7-3%E7%81%AB%E9%BE%99%E6%9E%9C%E8%BD%AC%E5%BD%95%E7%BB%84%E6%B5%8B%E5%BA%8F\%E5%AE%8C%E6%95%B4%E7%89%88%E6%95%B0%E6%8D%AE\GDR3855-Hylocereus_undulatus_Britt-12-RNAseq_result\4_Function\2_Group_Diff_Function\UP_DOWN\GO\NL-VS-L1.F.html#gene92) | extracellular ligand-gated ion channel activity | 2 (0.16%) | 3 (0.09%) | 0.289196 | 0.880068 |
| 93 | [GO:0015276](file:///E:\2018-7-3%E7%81%AB%E9%BE%99%E6%9E%9C%E8%BD%AC%E5%BD%95%E7%BB%84%E6%B5%8B%E5%BA%8F\%E5%AE%8C%E6%95%B4%E7%89%88%E6%95%B0%E6%8D%AE\GDR3855-Hylocereus_undulatus_Britt-12-RNAseq_result\4_Function\2_Group_Diff_Function\UP_DOWN\GO\NL-VS-L1.F.html#gene93) | ligand-gated ion channel activity | 2 (0.16%) | 3 (0.09%) | 0.289196 | 0.880068 |
| 94 | [GO:0016410](file:///E:\2018-7-3%E7%81%AB%E9%BE%99%E6%9E%9C%E8%BD%AC%E5%BD%95%E7%BB%84%E6%B5%8B%E5%BA%8F\%E5%AE%8C%E6%95%B4%E7%89%88%E6%95%B0%E6%8D%AE\GDR3855-Hylocereus_undulatus_Britt-12-RNAseq_result\4_Function\2_Group_Diff_Function\UP_DOWN\GO\NL-VS-L1.F.html#gene94) | N-acyltransferase activity | 2 (0.16%) | 3 (0.09%) | 0.289196 | 0.880068 |
| 95 | [GO:0016717](file:///E:\2018-7-3%E7%81%AB%E9%BE%99%E6%9E%9C%E8%BD%AC%E5%BD%95%E7%BB%84%E6%B5%8B%E5%BA%8F\%E5%AE%8C%E6%95%B4%E7%89%88%E6%95%B0%E6%8D%AE\GDR3855-Hylocereus_undulatus_Britt-12-RNAseq_result\4_Function\2_Group_Diff_Function\UP_DOWN\GO\NL-VS-L1.F.html#gene95) | oxidoreductase activity, acting on paired donors, with oxidation of a pair of donors resulting in the reduction of molecular oxygen to two molecules of water | 2 (0.16%) | 3 (0.09%) | 0.289196 | 0.880068 |
| 96 | [GO:0016744](file:///E:\2018-7-3%E7%81%AB%E9%BE%99%E6%9E%9C%E8%BD%AC%E5%BD%95%E7%BB%84%E6%B5%8B%E5%BA%8F\%E5%AE%8C%E6%95%B4%E7%89%88%E6%95%B0%E6%8D%AE\GDR3855-Hylocereus_undulatus_Britt-12-RNAseq_result\4_Function\2_Group_Diff_Function\UP_DOWN\GO\NL-VS-L1.F.html#gene96) | transferase activity, transferring aldehyde or ketonic groups | 2 (0.16%) | 3 (0.09%) | 0.289196 | 0.880068 |
| 97 | [GO:0016824](file:///E:\2018-7-3%E7%81%AB%E9%BE%99%E6%9E%9C%E8%BD%AC%E5%BD%95%E7%BB%84%E6%B5%8B%E5%BA%8F\%E5%AE%8C%E6%95%B4%E7%89%88%E6%95%B0%E6%8D%AE\GDR3855-Hylocereus_undulatus_Britt-12-RNAseq_result\4_Function\2_Group_Diff_Function\UP_DOWN\GO\NL-VS-L1.F.html#gene97) | hydrolase activity, acting on acid halide bonds | 2 (0.16%) | 3 (0.09%) | 0.289196 | 0.880068 |
| 98 | [GO:0019120](file:///E:\2018-7-3%E7%81%AB%E9%BE%99%E6%9E%9C%E8%BD%AC%E5%BD%95%E7%BB%84%E6%B5%8B%E5%BA%8F\%E5%AE%8C%E6%95%B4%E7%89%88%E6%95%B0%E6%8D%AE\GDR3855-Hylocereus_undulatus_Britt-12-RNAseq_result\4_Function\2_Group_Diff_Function\UP_DOWN\GO\NL-VS-L1.F.html#gene98) | hydrolase activity, acting on acid halide bonds, in C-halide compounds | 2 (0.16%) | 3 (0.09%) | 0.289196 | 0.880068 |
| 99 | [GO:0022834](file:///E:\2018-7-3%E7%81%AB%E9%BE%99%E6%9E%9C%E8%BD%AC%E5%BD%95%E7%BB%84%E6%B5%8B%E5%BA%8F\%E5%AE%8C%E6%95%B4%E7%89%88%E6%95%B0%E6%8D%AE\GDR3855-Hylocereus_undulatus_Britt-12-RNAseq_result\4_Function\2_Group_Diff_Function\UP_DOWN\GO\NL-VS-L1.F.html#gene99) | ligand-gated channel activity | 2 (0.16%) | 3 (0.09%) | 0.289196 | 0.880068 |
| 100 | [GO:0042285](file:///E:\2018-7-3%E7%81%AB%E9%BE%99%E6%9E%9C%E8%BD%AC%E5%BD%95%E7%BB%84%E6%B5%8B%E5%BA%8F\%E5%AE%8C%E6%95%B4%E7%89%88%E6%95%B0%E6%8D%AE\GDR3855-Hylocereus_undulatus_Britt-12-RNAseq_result\4_Function\2_Group_Diff_Function\UP_DOWN\GO\NL-VS-L1.F.html#gene100) | xylosyltransferase activity | 2 (0.16%) | 3 (0.09%) | 0.289196 | 0.880068 |
| 101 | [GO:0099600](file:///E:\2018-7-3%E7%81%AB%E9%BE%99%E6%9E%9C%E8%BD%AC%E5%BD%95%E7%BB%84%E6%B5%8B%E5%BA%8F\%E5%AE%8C%E6%95%B4%E7%89%88%E6%95%B0%E6%8D%AE\GDR3855-Hylocereus_undulatus_Britt-12-RNAseq_result\4_Function\2_Group_Diff_Function\UP_DOWN\GO\NL-VS-L1.F.html#gene101) | transmembrane receptor activity | 2 (0.16%) | 3 (0.09%) | 0.289196 | 0.880068 |
| 102 | [GO:0046527](file:///E:\2018-7-3%E7%81%AB%E9%BE%99%E6%9E%9C%E8%BD%AC%E5%BD%95%E7%BB%84%E6%B5%8B%E5%BA%8F\%E5%AE%8C%E6%95%B4%E7%89%88%E6%95%B0%E6%8D%AE\GDR3855-Hylocereus_undulatus_Britt-12-RNAseq_result\4_Function\2_Group_Diff_Function\UP_DOWN\GO\NL-VS-L1.F.html#gene102) | glucosyltransferase activity | 14 (1.15%) | 34 (0.99%) | 0.301222 | 0.880068 |
| 103 | [GO:0016407](file:///E:\2018-7-3%E7%81%AB%E9%BE%99%E6%9E%9C%E8%BD%AC%E5%BD%95%E7%BB%84%E6%B5%8B%E5%BA%8F\%E5%AE%8C%E6%95%B4%E7%89%88%E6%95%B0%E6%8D%AE\GDR3855-Hylocereus_undulatus_Britt-12-RNAseq_result\4_Function\2_Group_Diff_Function\UP_DOWN\GO\NL-VS-L1.F.html#gene103) | acetyltransferase activity | 4 (0.33%) | 8 (0.23%) | 0.305253 | 0.880068 |
| 104 | [GO:0019900](file:///E:\2018-7-3%E7%81%AB%E9%BE%99%E6%9E%9C%E8%BD%AC%E5%BD%95%E7%BB%84%E6%B5%8B%E5%BA%8F\%E5%AE%8C%E6%95%B4%E7%89%88%E6%95%B0%E6%8D%AE\GDR3855-Hylocereus_undulatus_Britt-12-RNAseq_result\4_Function\2_Group_Diff_Function\UP_DOWN\GO\NL-VS-L1.F.html#gene104) | kinase binding | 4 (0.33%) | 8 (0.23%) | 0.305253 | 0.880068 |
| 105 | [GO:0003676](file:///E:\2018-7-3%E7%81%AB%E9%BE%99%E6%9E%9C%E8%BD%AC%E5%BD%95%E7%BB%84%E6%B5%8B%E5%BA%8F\%E5%AE%8C%E6%95%B4%E7%89%88%E6%95%B0%E6%8D%AE\GDR3855-Hylocereus_undulatus_Britt-12-RNAseq_result\4_Function\2_Group_Diff_Function\UP_DOWN\GO\NL-VS-L1.F.html#gene105) | nucleic acid binding | 73 (5.98%) | 195 (5.68%) | 0.310294 | 0.880068 |
| 106 | [GO:0016779](file:///E:\2018-7-3%E7%81%AB%E9%BE%99%E6%9E%9C%E8%BD%AC%E5%BD%95%E7%BB%84%E6%B5%8B%E5%BA%8F\%E5%AE%8C%E6%95%B4%E7%89%88%E6%95%B0%E6%8D%AE\GDR3855-Hylocereus_undulatus_Britt-12-RNAseq_result\4_Function\2_Group_Diff_Function\UP_DOWN\GO\NL-VS-L1.F.html#gene106) | nucleotidyltransferase activity | 21 (1.72%) | 53 (1.54%) | 0.312105 | 0.880068 |
| 107 | [GO:0016830](file:///E:\2018-7-3%E7%81%AB%E9%BE%99%E6%9E%9C%E8%BD%AC%E5%BD%95%E7%BB%84%E6%B5%8B%E5%BA%8F\%E5%AE%8C%E6%95%B4%E7%89%88%E6%95%B0%E6%8D%AE\GDR3855-Hylocereus_undulatus_Britt-12-RNAseq_result\4_Function\2_Group_Diff_Function\UP_DOWN\GO\NL-VS-L1.F.html#gene107) | carbon-carbon lyase activity | 18 (1.48%) | 45 (1.31%) | 0.314829 | 0.880068 |
| 108 | [GO:0016301](file:///E:\2018-7-3%E7%81%AB%E9%BE%99%E6%9E%9C%E8%BD%AC%E5%BD%95%E7%BB%84%E6%B5%8B%E5%BA%8F\%E5%AE%8C%E6%95%B4%E7%89%88%E6%95%B0%E6%8D%AE\GDR3855-Hylocereus_undulatus_Britt-12-RNAseq_result\4_Function\2_Group_Diff_Function\UP_DOWN\GO\NL-VS-L1.F.html#gene108) | kinase activity | 134 (10.98%) | 364 (10.61%) | 0.316011 | 0.880068 |
| 109 | [GO:0022891](file:///E:\2018-7-3%E7%81%AB%E9%BE%99%E6%9E%9C%E8%BD%AC%E5%BD%95%E7%BB%84%E6%B5%8B%E5%BA%8F\%E5%AE%8C%E6%95%B4%E7%89%88%E6%95%B0%E6%8D%AE\GDR3855-Hylocereus_undulatus_Britt-12-RNAseq_result\4_Function\2_Group_Diff_Function\UP_DOWN\GO\NL-VS-L1.F.html#gene109) | substrate-specific transmembrane transporter activity | 60 (4.92%) | 160 (4.66%) | 0.326516 | 0.880068 |
| 110 | [GO:0042578](file:///E:\2018-7-3%E7%81%AB%E9%BE%99%E6%9E%9C%E8%BD%AC%E5%BD%95%E7%BB%84%E6%B5%8B%E5%BA%8F\%E5%AE%8C%E6%95%B4%E7%89%88%E6%95%B0%E6%8D%AE\GDR3855-Hylocereus_undulatus_Britt-12-RNAseq_result\4_Function\2_Group_Diff_Function\UP_DOWN\GO\NL-VS-L1.F.html#gene110) | phosphoric ester hydrolase activity | 34 (2.79%) | 89 (2.59%) | 0.335025 | 0.880068 |
| 111 | [GO:0015075](file:///E:\2018-7-3%E7%81%AB%E9%BE%99%E6%9E%9C%E8%BD%AC%E5%BD%95%E7%BB%84%E6%B5%8B%E5%BA%8F\%E5%AE%8C%E6%95%B4%E7%89%88%E6%95%B0%E6%8D%AE\GDR3855-Hylocereus_undulatus_Britt-12-RNAseq_result\4_Function\2_Group_Diff_Function\UP_DOWN\GO\NL-VS-L1.F.html#gene111) | ion transmembrane transporter activity | 50 (4.1%) | 133 (3.88%) | 0.338304 | 0.880068 |
| 112 | [GO:0008422](file:///E:\2018-7-3%E7%81%AB%E9%BE%99%E6%9E%9C%E8%BD%AC%E5%BD%95%E7%BB%84%E6%B5%8B%E5%BA%8F\%E5%AE%8C%E6%95%B4%E7%89%88%E6%95%B0%E6%8D%AE\GDR3855-Hylocereus_undulatus_Britt-12-RNAseq_result\4_Function\2_Group_Diff_Function\UP_DOWN\GO\NL-VS-L1.F.html#gene112) | beta-glucosidase activity | 5 (0.41%) | 11 (0.32%) | 0.345921 | 0.880068 |
| 113 | [GO:0008483](file:///E:\2018-7-3%E7%81%AB%E9%BE%99%E6%9E%9C%E8%BD%AC%E5%BD%95%E7%BB%84%E6%B5%8B%E5%BA%8F\%E5%AE%8C%E6%95%B4%E7%89%88%E6%95%B0%E6%8D%AE\GDR3855-Hylocereus_undulatus_Britt-12-RNAseq_result\4_Function\2_Group_Diff_Function\UP_DOWN\GO\NL-VS-L1.F.html#gene113) | transaminase activity | 5 (0.41%) | 11 (0.32%) | 0.345921 | 0.880068 |
| 114 | [GO:0008135](file:///E:\2018-7-3%E7%81%AB%E9%BE%99%E6%9E%9C%E8%BD%AC%E5%BD%95%E7%BB%84%E6%B5%8B%E5%BA%8F\%E5%AE%8C%E6%95%B4%E7%89%88%E6%95%B0%E6%8D%AE\GDR3855-Hylocereus_undulatus_Britt-12-RNAseq_result\4_Function\2_Group_Diff_Function\UP_DOWN\GO\NL-VS-L1.F.html#gene114) | translation factor activity, RNA binding | 11 (0.9%) | 27 (0.79%) | 0.351984 | 0.880068 |
| 115 | [GO:0000030](file:///E:\2018-7-3%E7%81%AB%E9%BE%99%E6%9E%9C%E8%BD%AC%E5%BD%95%E7%BB%84%E6%B5%8B%E5%BA%8F\%E5%AE%8C%E6%95%B4%E7%89%88%E6%95%B0%E6%8D%AE\GDR3855-Hylocereus_undulatus_Britt-12-RNAseq_result\4_Function\2_Group_Diff_Function\UP_DOWN\GO\NL-VS-L1.F.html#gene115) | mannosyltransferase activity | 1 (0.08%) | 1 (0.03%) | 0.355478 | 0.880068 |
| 116 | [GO:0000990](file:///E:\2018-7-3%E7%81%AB%E9%BE%99%E6%9E%9C%E8%BD%AC%E5%BD%95%E7%BB%84%E6%B5%8B%E5%BA%8F\%E5%AE%8C%E6%95%B4%E7%89%88%E6%95%B0%E6%8D%AE\GDR3855-Hylocereus_undulatus_Britt-12-RNAseq_result\4_Function\2_Group_Diff_Function\UP_DOWN\GO\NL-VS-L1.F.html#gene116) | transcription factor activity, core RNA polymerase binding | 1 (0.08%) | 1 (0.03%) | 0.355478 | 0.880068 |
| 117 | [GO:0000996](file:///E:\2018-7-3%E7%81%AB%E9%BE%99%E6%9E%9C%E8%BD%AC%E5%BD%95%E7%BB%84%E6%B5%8B%E5%BA%8F\%E5%AE%8C%E6%95%B4%E7%89%88%E6%95%B0%E6%8D%AE\GDR3855-Hylocereus_undulatus_Britt-12-RNAseq_result\4_Function\2_Group_Diff_Function\UP_DOWN\GO\NL-VS-L1.F.html#gene117) | core DNA-dependent RNA polymerase binding promoter specificity activity | 1 (0.08%) | 1 (0.03%) | 0.355478 | 0.880068 |
| 118 | [GO:0003973](file:///E:\2018-7-3%E7%81%AB%E9%BE%99%E6%9E%9C%E8%BD%AC%E5%BD%95%E7%BB%84%E6%B5%8B%E5%BA%8F\%E5%AE%8C%E6%95%B4%E7%89%88%E6%95%B0%E6%8D%AE\GDR3855-Hylocereus_undulatus_Britt-12-RNAseq_result\4_Function\2_Group_Diff_Function\UP_DOWN\GO\NL-VS-L1.F.html#gene118) | (S)-2-hydroxy-acid oxidase activity | 1 (0.08%) | 1 (0.03%) | 0.355478 | 0.880068 |
| 119 | [GO:0004463](file:///E:\2018-7-3%E7%81%AB%E9%BE%99%E6%9E%9C%E8%BD%AC%E5%BD%95%E7%BB%84%E6%B5%8B%E5%BA%8F\%E5%AE%8C%E6%95%B4%E7%89%88%E6%95%B0%E6%8D%AE\GDR3855-Hylocereus_undulatus_Britt-12-RNAseq_result\4_Function\2_Group_Diff_Function\UP_DOWN\GO\NL-VS-L1.F.html#gene119) | leukotriene-A4 hydrolase activity | 1 (0.08%) | 1 (0.03%) | 0.355478 | 0.880068 |
| 120 | [GO:0004629](file:///E:\2018-7-3%E7%81%AB%E9%BE%99%E6%9E%9C%E8%BD%AC%E5%BD%95%E7%BB%84%E6%B5%8B%E5%BA%8F\%E5%AE%8C%E6%95%B4%E7%89%88%E6%95%B0%E6%8D%AE\GDR3855-Hylocereus_undulatus_Britt-12-RNAseq_result\4_Function\2_Group_Diff_Function\UP_DOWN\GO\NL-VS-L1.F.html#gene120) | phospholipase C activity | 1 (0.08%) | 1 (0.03%) | 0.355478 | 0.880068 |
| 121 | [GO:0004661](file:///E:\2018-7-3%E7%81%AB%E9%BE%99%E6%9E%9C%E8%BD%AC%E5%BD%95%E7%BB%84%E6%B5%8B%E5%BA%8F\%E5%AE%8C%E6%95%B4%E7%89%88%E6%95%B0%E6%8D%AE\GDR3855-Hylocereus_undulatus_Britt-12-RNAseq_result\4_Function\2_Group_Diff_Function\UP_DOWN\GO\NL-VS-L1.F.html#gene121) | protein geranylgeranyltransferase activity | 1 (0.08%) | 1 (0.03%) | 0.355478 | 0.880068 |
| 122 | [GO:0004866](file:///E:\2018-7-3%E7%81%AB%E9%BE%99%E6%9E%9C%E8%BD%AC%E5%BD%95%E7%BB%84%E6%B5%8B%E5%BA%8F\%E5%AE%8C%E6%95%B4%E7%89%88%E6%95%B0%E6%8D%AE\GDR3855-Hylocereus_undulatus_Britt-12-RNAseq_result\4_Function\2_Group_Diff_Function\UP_DOWN\GO\NL-VS-L1.F.html#gene122) | endopeptidase inhibitor activity | 1 (0.08%) | 1 (0.03%) | 0.355478 | 0.880068 |
| 123 | [GO:0005262](file:///E:\2018-7-3%E7%81%AB%E9%BE%99%E6%9E%9C%E8%BD%AC%E5%BD%95%E7%BB%84%E6%B5%8B%E5%BA%8F\%E5%AE%8C%E6%95%B4%E7%89%88%E6%95%B0%E6%8D%AE\GDR3855-Hylocereus_undulatus_Britt-12-RNAseq_result\4_Function\2_Group_Diff_Function\UP_DOWN\GO\NL-VS-L1.F.html#gene123) | calcium channel activity | 1 (0.08%) | 1 (0.03%) | 0.355478 | 0.880068 |
| 124 | [GO:0008017](file:///E:\2018-7-3%E7%81%AB%E9%BE%99%E6%9E%9C%E8%BD%AC%E5%BD%95%E7%BB%84%E6%B5%8B%E5%BA%8F\%E5%AE%8C%E6%95%B4%E7%89%88%E6%95%B0%E6%8D%AE\GDR3855-Hylocereus_undulatus_Britt-12-RNAseq_result\4_Function\2_Group_Diff_Function\UP_DOWN\GO\NL-VS-L1.F.html#gene124) | microtubule binding | 1 (0.08%) | 1 (0.03%) | 0.355478 | 0.880068 |
| 125 | [GO:0008026](file:///E:\2018-7-3%E7%81%AB%E9%BE%99%E6%9E%9C%E8%BD%AC%E5%BD%95%E7%BB%84%E6%B5%8B%E5%BA%8F\%E5%AE%8C%E6%95%B4%E7%89%88%E6%95%B0%E6%8D%AE\GDR3855-Hylocereus_undulatus_Britt-12-RNAseq_result\4_Function\2_Group_Diff_Function\UP_DOWN\GO\NL-VS-L1.F.html#gene125) | ATP-dependent helicase activity | 1 (0.08%) | 1 (0.03%) | 0.355478 | 0.880068 |
| 126 | [GO:0008318](file:///E:\2018-7-3%E7%81%AB%E9%BE%99%E6%9E%9C%E8%BD%AC%E5%BD%95%E7%BB%84%E6%B5%8B%E5%BA%8F\%E5%AE%8C%E6%95%B4%E7%89%88%E6%95%B0%E6%8D%AE\GDR3855-Hylocereus_undulatus_Britt-12-RNAseq_result\4_Function\2_Group_Diff_Function\UP_DOWN\GO\NL-VS-L1.F.html#gene126) | protein prenyltransferase activity | 1 (0.08%) | 1 (0.03%) | 0.355478 | 0.880068 |
| 127 | [GO:0008905](file:///E:\2018-7-3%E7%81%AB%E9%BE%99%E6%9E%9C%E8%BD%AC%E5%BD%95%E7%BB%84%E6%B5%8B%E5%BA%8F\%E5%AE%8C%E6%95%B4%E7%89%88%E6%95%B0%E6%8D%AE\GDR3855-Hylocereus_undulatus_Britt-12-RNAseq_result\4_Function\2_Group_Diff_Function\UP_DOWN\GO\NL-VS-L1.F.html#gene127) | mannose-phosphate guanylyltransferase activity | 1 (0.08%) | 1 (0.03%) | 0.355478 | 0.880068 |
| 128 | [GO:0015020](file:///E:\2018-7-3%E7%81%AB%E9%BE%99%E6%9E%9C%E8%BD%AC%E5%BD%95%E7%BB%84%E6%B5%8B%E5%BA%8F\%E5%AE%8C%E6%95%B4%E7%89%88%E6%95%B0%E6%8D%AE\GDR3855-Hylocereus_undulatus_Britt-12-RNAseq_result\4_Function\2_Group_Diff_Function\UP_DOWN\GO\NL-VS-L1.F.html#gene128) | glucuronosyltransferase activity | 1 (0.08%) | 1 (0.03%) | 0.355478 | 0.880068 |
| 129 | [GO:0015116](file:///E:\2018-7-3%E7%81%AB%E9%BE%99%E6%9E%9C%E8%BD%AC%E5%BD%95%E7%BB%84%E6%B5%8B%E5%BA%8F\%E5%AE%8C%E6%95%B4%E7%89%88%E6%95%B0%E6%8D%AE\GDR3855-Hylocereus_undulatus_Britt-12-RNAseq_result\4_Function\2_Group_Diff_Function\UP_DOWN\GO\NL-VS-L1.F.html#gene129) | sulfate transmembrane transporter activity | 1 (0.08%) | 1 (0.03%) | 0.355478 | 0.880068 |
| 130 | [GO:0015205](file:///E:\2018-7-3%E7%81%AB%E9%BE%99%E6%9E%9C%E8%BD%AC%E5%BD%95%E7%BB%84%E6%B5%8B%E5%BA%8F\%E5%AE%8C%E6%95%B4%E7%89%88%E6%95%B0%E6%8D%AE\GDR3855-Hylocereus_undulatus_Britt-12-RNAseq_result\4_Function\2_Group_Diff_Function\UP_DOWN\GO\NL-VS-L1.F.html#gene130) | nucleobase transmembrane transporter activity | 1 (0.08%) | 1 (0.03%) | 0.355478 | 0.880068 |
| 131 | [GO:0015368](file:///E:\2018-7-3%E7%81%AB%E9%BE%99%E6%9E%9C%E8%BD%AC%E5%BD%95%E7%BB%84%E6%B5%8B%E5%BA%8F\%E5%AE%8C%E6%95%B4%E7%89%88%E6%95%B0%E6%8D%AE\GDR3855-Hylocereus_undulatus_Britt-12-RNAseq_result\4_Function\2_Group_Diff_Function\UP_DOWN\GO\NL-VS-L1.F.html#gene131) | calcium:cation antiporter activity | 1 (0.08%) | 1 (0.03%) | 0.355478 | 0.880068 |
| 132 | [GO:0015563](file:///E:\2018-7-3%E7%81%AB%E9%BE%99%E6%9E%9C%E8%BD%AC%E5%BD%95%E7%BB%84%E6%B5%8B%E5%BA%8F\%E5%AE%8C%E6%95%B4%E7%89%88%E6%95%B0%E6%8D%AE\GDR3855-Hylocereus_undulatus_Britt-12-RNAseq_result\4_Function\2_Group_Diff_Function\UP_DOWN\GO\NL-VS-L1.F.html#gene132) | uptake transmembrane transporter activity | 1 (0.08%) | 1 (0.03%) | 0.355478 | 0.880068 |
| 133 | [GO:0015928](file:///E:\2018-7-3%E7%81%AB%E9%BE%99%E6%9E%9C%E8%BD%AC%E5%BD%95%E7%BB%84%E6%B5%8B%E5%BA%8F\%E5%AE%8C%E6%95%B4%E7%89%88%E6%95%B0%E6%8D%AE\GDR3855-Hylocereus_undulatus_Britt-12-RNAseq_result\4_Function\2_Group_Diff_Function\UP_DOWN\GO\NL-VS-L1.F.html#gene133) | fucosidase activity | 1 (0.08%) | 1 (0.03%) | 0.355478 | 0.880068 |
| 134 | [GO:0016623](file:///E:\2018-7-3%E7%81%AB%E9%BE%99%E6%9E%9C%E8%BD%AC%E5%BD%95%E7%BB%84%E6%B5%8B%E5%BA%8F\%E5%AE%8C%E6%95%B4%E7%89%88%E6%95%B0%E6%8D%AE\GDR3855-Hylocereus_undulatus_Britt-12-RNAseq_result\4_Function\2_Group_Diff_Function\UP_DOWN\GO\NL-VS-L1.F.html#gene134) | oxidoreductase activity, acting on the aldehyde or oxo group of donors, oxygen as acceptor | 1 (0.08%) | 1 (0.03%) | 0.355478 | 0.880068 |
| 135 | [GO:0016641](file:///E:\2018-7-3%E7%81%AB%E9%BE%99%E6%9E%9C%E8%BD%AC%E5%BD%95%E7%BB%84%E6%B5%8B%E5%BA%8F\%E5%AE%8C%E6%95%B4%E7%89%88%E6%95%B0%E6%8D%AE\GDR3855-Hylocereus_undulatus_Britt-12-RNAseq_result\4_Function\2_Group_Diff_Function\UP_DOWN\GO\NL-VS-L1.F.html#gene135) | oxidoreductase activity, acting on the CH-NH2 group of donors, oxygen as acceptor | 1 (0.08%) | 1 (0.03%) | 0.355478 | 0.880068 |
| 136 | [GO:0016755](file:///E:\2018-7-3%E7%81%AB%E9%BE%99%E6%9E%9C%E8%BD%AC%E5%BD%95%E7%BB%84%E6%B5%8B%E5%BA%8F\%E5%AE%8C%E6%95%B4%E7%89%88%E6%95%B0%E6%8D%AE\GDR3855-Hylocereus_undulatus_Britt-12-RNAseq_result\4_Function\2_Group_Diff_Function\UP_DOWN\GO\NL-VS-L1.F.html#gene136) | transferase activity, transferring amino-acyl groups | 1 (0.08%) | 1 (0.03%) | 0.355478 | 0.880068 |
| 137 | [GO:0016781](file:///E:\2018-7-3%E7%81%AB%E9%BE%99%E6%9E%9C%E8%BD%AC%E5%BD%95%E7%BB%84%E6%B5%8B%E5%BA%8F\%E5%AE%8C%E6%95%B4%E7%89%88%E6%95%B0%E6%8D%AE\GDR3855-Hylocereus_undulatus_Britt-12-RNAseq_result\4_Function\2_Group_Diff_Function\UP_DOWN\GO\NL-VS-L1.F.html#gene137) | phosphotransferase activity, paired acceptors | 1 (0.08%) | 1 (0.03%) | 0.355478 | 0.880068 |
| 138 | [GO:0016801](file:///E:\2018-7-3%E7%81%AB%E9%BE%99%E6%9E%9C%E8%BD%AC%E5%BD%95%E7%BB%84%E6%B5%8B%E5%BA%8F\%E5%AE%8C%E6%95%B4%E7%89%88%E6%95%B0%E6%8D%AE\GDR3855-Hylocereus_undulatus_Britt-12-RNAseq_result\4_Function\2_Group_Diff_Function\UP_DOWN\GO\NL-VS-L1.F.html#gene138) | hydrolase activity, acting on ether bonds | 1 (0.08%) | 1 (0.03%) | 0.355478 | 0.880068 |
| 139 | [GO:0016803](file:///E:\2018-7-3%E7%81%AB%E9%BE%99%E6%9E%9C%E8%BD%AC%E5%BD%95%E7%BB%84%E6%B5%8B%E5%BA%8F\%E5%AE%8C%E6%95%B4%E7%89%88%E6%95%B0%E6%8D%AE\GDR3855-Hylocereus_undulatus_Britt-12-RNAseq_result\4_Function\2_Group_Diff_Function\UP_DOWN\GO\NL-VS-L1.F.html#gene139) | ether hydrolase activity | 1 (0.08%) | 1 (0.03%) | 0.355478 | 0.880068 |
| 140 | [GO:0016819](file:///E:\2018-7-3%E7%81%AB%E9%BE%99%E6%9E%9C%E8%BD%AC%E5%BD%95%E7%BB%84%E6%B5%8B%E5%BA%8F\%E5%AE%8C%E6%95%B4%E7%89%88%E6%95%B0%E6%8D%AE\GDR3855-Hylocereus_undulatus_Britt-12-RNAseq_result\4_Function\2_Group_Diff_Function\UP_DOWN\GO\NL-VS-L1.F.html#gene140) | hydrolase activity, acting on acid anhydrides, in sulfonyl-containing anhydrides | 1 (0.08%) | 1 (0.03%) | 0.355478 | 0.880068 |
| 141 | [GO:0016842](file:///E:\2018-7-3%E7%81%AB%E9%BE%99%E6%9E%9C%E8%BD%AC%E5%BD%95%E7%BB%84%E6%B5%8B%E5%BA%8F\%E5%AE%8C%E6%95%B4%E7%89%88%E6%95%B0%E6%8D%AE\GDR3855-Hylocereus_undulatus_Britt-12-RNAseq_result\4_Function\2_Group_Diff_Function\UP_DOWN\GO\NL-VS-L1.F.html#gene141) | amidine-lyase activity | 1 (0.08%) | 1 (0.03%) | 0.355478 | 0.880068 |
| 142 | [GO:0018271](file:///E:\2018-7-3%E7%81%AB%E9%BE%99%E6%9E%9C%E8%BD%AC%E5%BD%95%E7%BB%84%E6%B5%8B%E5%BA%8F\%E5%AE%8C%E6%95%B4%E7%89%88%E6%95%B0%E6%8D%AE\GDR3855-Hylocereus_undulatus_Britt-12-RNAseq_result\4_Function\2_Group_Diff_Function\UP_DOWN\GO\NL-VS-L1.F.html#gene142) | biotin-protein ligase activity | 1 (0.08%) | 1 (0.03%) | 0.355478 | 0.880068 |
| 143 | [GO:0019207](file:///E:\2018-7-3%E7%81%AB%E9%BE%99%E6%9E%9C%E8%BD%AC%E5%BD%95%E7%BB%84%E6%B5%8B%E5%BA%8F\%E5%AE%8C%E6%95%B4%E7%89%88%E6%95%B0%E6%8D%AE\GDR3855-Hylocereus_undulatus_Britt-12-RNAseq_result\4_Function\2_Group_Diff_Function\UP_DOWN\GO\NL-VS-L1.F.html#gene143) | kinase regulator activity | 1 (0.08%) | 1 (0.03%) | 0.355478 | 0.880068 |
| 144 | [GO:0019887](file:///E:\2018-7-3%E7%81%AB%E9%BE%99%E6%9E%9C%E8%BD%AC%E5%BD%95%E7%BB%84%E6%B5%8B%E5%BA%8F\%E5%AE%8C%E6%95%B4%E7%89%88%E6%95%B0%E6%8D%AE\GDR3855-Hylocereus_undulatus_Britt-12-RNAseq_result\4_Function\2_Group_Diff_Function\UP_DOWN\GO\NL-VS-L1.F.html#gene144) | protein kinase regulator activity | 1 (0.08%) | 1 (0.03%) | 0.355478 | 0.880068 |
| 145 | [GO:0019904](file:///E:\2018-7-3%E7%81%AB%E9%BE%99%E6%9E%9C%E8%BD%AC%E5%BD%95%E7%BB%84%E6%B5%8B%E5%BA%8F\%E5%AE%8C%E6%95%B4%E7%89%88%E6%95%B0%E6%8D%AE\GDR3855-Hylocereus_undulatus_Britt-12-RNAseq_result\4_Function\2_Group_Diff_Function\UP_DOWN\GO\NL-VS-L1.F.html#gene145) | protein domain specific binding | 1 (0.08%) | 1 (0.03%) | 0.355478 | 0.880068 |
| 146 | [GO:0030414](file:///E:\2018-7-3%E7%81%AB%E9%BE%99%E6%9E%9C%E8%BD%AC%E5%BD%95%E7%BB%84%E6%B5%8B%E5%BA%8F\%E5%AE%8C%E6%95%B4%E7%89%88%E6%95%B0%E6%8D%AE\GDR3855-Hylocereus_undulatus_Britt-12-RNAseq_result\4_Function\2_Group_Diff_Function\UP_DOWN\GO\NL-VS-L1.F.html#gene146) | peptidase inhibitor activity | 1 (0.08%) | 1 (0.03%) | 0.355478 | 0.880068 |
| 147 | [GO:0031559](file:///E:\2018-7-3%E7%81%AB%E9%BE%99%E6%9E%9C%E8%BD%AC%E5%BD%95%E7%BB%84%E6%B5%8B%E5%BA%8F\%E5%AE%8C%E6%95%B4%E7%89%88%E6%95%B0%E6%8D%AE\GDR3855-Hylocereus_undulatus_Britt-12-RNAseq_result\4_Function\2_Group_Diff_Function\UP_DOWN\GO\NL-VS-L1.F.html#gene147) | oxidosqualene cyclase activity | 1 (0.08%) | 1 (0.03%) | 0.355478 | 0.880068 |
| 148 | [GO:0035252](file:///E:\2018-7-3%E7%81%AB%E9%BE%99%E6%9E%9C%E8%BD%AC%E5%BD%95%E7%BB%84%E6%B5%8B%E5%BA%8F\%E5%AE%8C%E6%95%B4%E7%89%88%E6%95%B0%E6%8D%AE\GDR3855-Hylocereus_undulatus_Britt-12-RNAseq_result\4_Function\2_Group_Diff_Function\UP_DOWN\GO\NL-VS-L1.F.html#gene148) | UDP-xylosyltransferase activity | 1 (0.08%) | 1 (0.03%) | 0.355478 | 0.880068 |
| 149 | [GO:0051748](file:///E:\2018-7-3%E7%81%AB%E9%BE%99%E6%9E%9C%E8%BD%AC%E5%BD%95%E7%BB%84%E6%B5%8B%E5%BA%8F\%E5%AE%8C%E6%95%B4%E7%89%88%E6%95%B0%E6%8D%AE\GDR3855-Hylocereus_undulatus_Britt-12-RNAseq_result\4_Function\2_Group_Diff_Function\UP_DOWN\GO\NL-VS-L1.F.html#gene149) | UTP-monosaccharide-1-phosphate uridylyltransferase activity | 1 (0.08%) | 1 (0.03%) | 0.355478 | 0.880068 |
| 150 | [GO:0052736](file:///E:\2018-7-3%E7%81%AB%E9%BE%99%E6%9E%9C%E8%BD%AC%E5%BD%95%E7%BB%84%E6%B5%8B%E5%BA%8F\%E5%AE%8C%E6%95%B4%E7%89%88%E6%95%B0%E6%8D%AE\GDR3855-Hylocereus_undulatus_Britt-12-RNAseq_result\4_Function\2_Group_Diff_Function\UP_DOWN\GO\NL-VS-L1.F.html#gene150) | beta-glucanase activity | 1 (0.08%) | 1 (0.03%) | 0.355478 | 0.880068 |
| 151 | [GO:0052880](file:///E:\2018-7-3%E7%81%AB%E9%BE%99%E6%9E%9C%E8%BD%AC%E5%BD%95%E7%BB%84%E6%B5%8B%E5%BA%8F\%E5%AE%8C%E6%95%B4%E7%89%88%E6%95%B0%E6%8D%AE\GDR3855-Hylocereus_undulatus_Britt-12-RNAseq_result\4_Function\2_Group_Diff_Function\UP_DOWN\GO\NL-VS-L1.F.html#gene151) | oxidoreductase activity, acting on diphenols and related substances as donors, with copper protein as acceptor | 1 (0.08%) | 1 (0.03%) | 0.355478 | 0.880068 |
| 152 | [GO:0061134](file:///E:\2018-7-3%E7%81%AB%E9%BE%99%E6%9E%9C%E8%BD%AC%E5%BD%95%E7%BB%84%E6%B5%8B%E5%BA%8F\%E5%AE%8C%E6%95%B4%E7%89%88%E6%95%B0%E6%8D%AE\GDR3855-Hylocereus_undulatus_Britt-12-RNAseq_result\4_Function\2_Group_Diff_Function\UP_DOWN\GO\NL-VS-L1.F.html#gene152) | peptidase regulator activity | 1 (0.08%) | 1 (0.03%) | 0.355478 | 0.880068 |
| 153 | [GO:0061135](file:///E:\2018-7-3%E7%81%AB%E9%BE%99%E6%9E%9C%E8%BD%AC%E5%BD%95%E7%BB%84%E6%B5%8B%E5%BA%8F\%E5%AE%8C%E6%95%B4%E7%89%88%E6%95%B0%E6%8D%AE\GDR3855-Hylocereus_undulatus_Britt-12-RNAseq_result\4_Function\2_Group_Diff_Function\UP_DOWN\GO\NL-VS-L1.F.html#gene153) | endopeptidase regulator activity | 1 (0.08%) | 1 (0.03%) | 0.355478 | 0.880068 |
| 154 | [GO:0070035](file:///E:\2018-7-3%E7%81%AB%E9%BE%99%E6%9E%9C%E8%BD%AC%E5%BD%95%E7%BB%84%E6%B5%8B%E5%BA%8F\%E5%AE%8C%E6%95%B4%E7%89%88%E6%95%B0%E6%8D%AE\GDR3855-Hylocereus_undulatus_Britt-12-RNAseq_result\4_Function\2_Group_Diff_Function\UP_DOWN\GO\NL-VS-L1.F.html#gene154) | purine NTP-dependent helicase activity | 1 (0.08%) | 1 (0.03%) | 0.355478 | 0.880068 |
| 155 | [GO:0070547](file:///E:\2018-7-3%E7%81%AB%E9%BE%99%E6%9E%9C%E8%BD%AC%E5%BD%95%E7%BB%84%E6%B5%8B%E5%BA%8F\%E5%AE%8C%E6%95%B4%E7%89%88%E6%95%B0%E6%8D%AE\GDR3855-Hylocereus_undulatus_Britt-12-RNAseq_result\4_Function\2_Group_Diff_Function\UP_DOWN\GO\NL-VS-L1.F.html#gene155) | L-tyrosine aminotransferase activity | 1 (0.08%) | 1 (0.03%) | 0.355478 | 0.880068 |
| 156 | [GO:0070568](file:///E:\2018-7-3%E7%81%AB%E9%BE%99%E6%9E%9C%E8%BD%AC%E5%BD%95%E7%BB%84%E6%B5%8B%E5%BA%8F\%E5%AE%8C%E6%95%B4%E7%89%88%E6%95%B0%E6%8D%AE\GDR3855-Hylocereus_undulatus_Britt-12-RNAseq_result\4_Function\2_Group_Diff_Function\UP_DOWN\GO\NL-VS-L1.F.html#gene156) | guanylyltransferase activity | 1 (0.08%) | 1 (0.03%) | 0.355478 | 0.880068 |
| 157 | [GO:0070569](file:///E:\2018-7-3%E7%81%AB%E9%BE%99%E6%9E%9C%E8%BD%AC%E5%BD%95%E7%BB%84%E6%B5%8B%E5%BA%8F\%E5%AE%8C%E6%95%B4%E7%89%88%E6%95%B0%E6%8D%AE\GDR3855-Hylocereus_undulatus_Britt-12-RNAseq_result\4_Function\2_Group_Diff_Function\UP_DOWN\GO\NL-VS-L1.F.html#gene157) | uridylyltransferase activity | 1 (0.08%) | 1 (0.03%) | 0.355478 | 0.880068 |
| 158 | [GO:1901682](file:///E:\2018-7-3%E7%81%AB%E9%BE%99%E6%9E%9C%E8%BD%AC%E5%BD%95%E7%BB%84%E6%B5%8B%E5%BA%8F\%E5%AE%8C%E6%95%B4%E7%89%88%E6%95%B0%E6%8D%AE\GDR3855-Hylocereus_undulatus_Britt-12-RNAseq_result\4_Function\2_Group_Diff_Function\UP_DOWN\GO\NL-VS-L1.F.html#gene158) | sulfur compound transmembrane transporter activity | 1 (0.08%) | 1 (0.03%) | 0.355478 | 0.880068 |
| 159 | [GO:0003727](file:///E:\2018-7-3%E7%81%AB%E9%BE%99%E6%9E%9C%E8%BD%AC%E5%BD%95%E7%BB%84%E6%B5%8B%E5%BA%8F\%E5%AE%8C%E6%95%B4%E7%89%88%E6%95%B0%E6%8D%AE\GDR3855-Hylocereus_undulatus_Britt-12-RNAseq_result\4_Function\2_Group_Diff_Function\UP_DOWN\GO\NL-VS-L1.F.html#gene159) | single-stranded RNA binding | 3 (0.25%) | 6 (0.17%) | 0.363938 | 0.880068 |
| 160 | [GO:0005451](file:///E:\2018-7-3%E7%81%AB%E9%BE%99%E6%9E%9C%E8%BD%AC%E5%BD%95%E7%BB%84%E6%B5%8B%E5%BA%8F\%E5%AE%8C%E6%95%B4%E7%89%88%E6%95%B0%E6%8D%AE\GDR3855-Hylocereus_undulatus_Britt-12-RNAseq_result\4_Function\2_Group_Diff_Function\UP_DOWN\GO\NL-VS-L1.F.html#gene160) | monovalent cation:proton antiporter activity | 3 (0.25%) | 6 (0.17%) | 0.363938 | 0.880068 |
| 161 | [GO:0008187](file:///E:\2018-7-3%E7%81%AB%E9%BE%99%E6%9E%9C%E8%BD%AC%E5%BD%95%E7%BB%84%E6%B5%8B%E5%BA%8F\%E5%AE%8C%E6%95%B4%E7%89%88%E6%95%B0%E6%8D%AE\GDR3855-Hylocereus_undulatus_Britt-12-RNAseq_result\4_Function\2_Group_Diff_Function\UP_DOWN\GO\NL-VS-L1.F.html#gene161) | poly-pyrimidine tract binding | 3 (0.25%) | 6 (0.17%) | 0.363938 | 0.880068 |
| 162 | [GO:0015299](file:///E:\2018-7-3%E7%81%AB%E9%BE%99%E6%9E%9C%E8%BD%AC%E5%BD%95%E7%BB%84%E6%B5%8B%E5%BA%8F\%E5%AE%8C%E6%95%B4%E7%89%88%E6%95%B0%E6%8D%AE\GDR3855-Hylocereus_undulatus_Britt-12-RNAseq_result\4_Function\2_Group_Diff_Function\UP_DOWN\GO\NL-VS-L1.F.html#gene162) | solute:proton antiporter activity | 3 (0.25%) | 6 (0.17%) | 0.363938 | 0.880068 |
| 163 | [GO:0015932](file:///E:\2018-7-3%E7%81%AB%E9%BE%99%E6%9E%9C%E8%BD%AC%E5%BD%95%E7%BB%84%E6%B5%8B%E5%BA%8F\%E5%AE%8C%E6%95%B4%E7%89%88%E6%95%B0%E6%8D%AE\GDR3855-Hylocereus_undulatus_Britt-12-RNAseq_result\4_Function\2_Group_Diff_Function\UP_DOWN\GO\NL-VS-L1.F.html#gene163) | nucleobase-containing compound transmembrane transporter activity | 3 (0.25%) | 6 (0.17%) | 0.363938 | 0.880068 |
| 164 | [GO:0016868](file:///E:\2018-7-3%E7%81%AB%E9%BE%99%E6%9E%9C%E8%BD%AC%E5%BD%95%E7%BB%84%E6%B5%8B%E5%BA%8F\%E5%AE%8C%E6%95%B4%E7%89%88%E6%95%B0%E6%8D%AE\GDR3855-Hylocereus_undulatus_Britt-12-RNAseq_result\4_Function\2_Group_Diff_Function\UP_DOWN\GO\NL-VS-L1.F.html#gene164) | intramolecular transferase activity, phosphotransferases | 3 (0.25%) | 6 (0.17%) | 0.363938 | 0.880068 |
| 165 | [GO:0052745](file:///E:\2018-7-3%E7%81%AB%E9%BE%99%E6%9E%9C%E8%BD%AC%E5%BD%95%E7%BB%84%E6%B5%8B%E5%BA%8F\%E5%AE%8C%E6%95%B4%E7%89%88%E6%95%B0%E6%8D%AE\GDR3855-Hylocereus_undulatus_Britt-12-RNAseq_result\4_Function\2_Group_Diff_Function\UP_DOWN\GO\NL-VS-L1.F.html#gene165) | inositol phosphate phosphatase activity | 3 (0.25%) | 6 (0.17%) | 0.363938 | 0.880068 |
| 166 | [GO:0035251](file:///E:\2018-7-3%E7%81%AB%E9%BE%99%E6%9E%9C%E8%BD%AC%E5%BD%95%E7%BB%84%E6%B5%8B%E5%BA%8F\%E5%AE%8C%E6%95%B4%E7%89%88%E6%95%B0%E6%8D%AE\GDR3855-Hylocereus_undulatus_Britt-12-RNAseq_result\4_Function\2_Group_Diff_Function\UP_DOWN\GO\NL-VS-L1.F.html#gene166) | UDP-glucosyltransferase activity | 9 (0.74%) | 22 (0.64%) | 0.373663 | 0.898143 |
| 167 | [GO:0050308](file:///E:\2018-7-3%E7%81%AB%E9%BE%99%E6%9E%9C%E8%BD%AC%E5%BD%95%E7%BB%84%E6%B5%8B%E5%BA%8F\%E5%AE%8C%E6%95%B4%E7%89%88%E6%95%B0%E6%8D%AE\GDR3855-Hylocereus_undulatus_Britt-12-RNAseq_result\4_Function\2_Group_Diff_Function\UP_DOWN\GO\NL-VS-L1.F.html#gene167) | sugar-phosphatase activity | 4 (0.33%) | 9 (0.26%) | 0.404813 | 0.961431 |
| 168 | [GO:0070566](file:///E:\2018-7-3%E7%81%AB%E9%BE%99%E6%9E%9C%E8%BD%AC%E5%BD%95%E7%BB%84%E6%B5%8B%E5%BA%8F\%E5%AE%8C%E6%95%B4%E7%89%88%E6%95%B0%E6%8D%AE\GDR3855-Hylocereus_undulatus_Britt-12-RNAseq_result\4_Function\2_Group_Diff_Function\UP_DOWN\GO\NL-VS-L1.F.html#gene168) | adenylyltransferase activity | 4 (0.33%) | 9 (0.26%) | 0.404813 | 0.961431 |
| 169 | [GO:0016769](file:///E:\2018-7-3%E7%81%AB%E9%BE%99%E6%9E%9C%E8%BD%AC%E5%BD%95%E7%BB%84%E6%B5%8B%E5%BA%8F\%E5%AE%8C%E6%95%B4%E7%89%88%E6%95%B0%E6%8D%AE\GDR3855-Hylocereus_undulatus_Britt-12-RNAseq_result\4_Function\2_Group_Diff_Function\UP_DOWN\GO\NL-VS-L1.F.html#gene169) | transferase activity, transferring nitrogenous groups | 5 (0.41%) | 12 (0.35%) | 0.432599 | 0.980162 |
| 170 | [GO:0046906](file:///E:\2018-7-3%E7%81%AB%E9%BE%99%E6%9E%9C%E8%BD%AC%E5%BD%95%E7%BB%84%E6%B5%8B%E5%BA%8F\%E5%AE%8C%E6%95%B4%E7%89%88%E6%95%B0%E6%8D%AE\GDR3855-Hylocereus_undulatus_Britt-12-RNAseq_result\4_Function\2_Group_Diff_Function\UP_DOWN\GO\NL-VS-L1.F.html#gene170) | tetrapyrrole binding | 26 (2.13%) | 70 (2.04%) | 0.433760 | 0.980162 |
| 171 | [GO:0008443](file:///E:\2018-7-3%E7%81%AB%E9%BE%99%E6%9E%9C%E8%BD%AC%E5%BD%95%E7%BB%84%E6%B5%8B%E5%BA%8F\%E5%AE%8C%E6%95%B4%E7%89%88%E6%95%B0%E6%8D%AE\GDR3855-Hylocereus_undulatus_Britt-12-RNAseq_result\4_Function\2_Group_Diff_Function\UP_DOWN\GO\NL-VS-L1.F.html#gene171) | phosphofructokinase activity | 2 (0.16%) | 4 (0.12%) | 0.446750 | 0.980162 |
| 172 | [GO:0015197](file:///E:\2018-7-3%E7%81%AB%E9%BE%99%E6%9E%9C%E8%BD%AC%E5%BD%95%E7%BB%84%E6%B5%8B%E5%BA%8F\%E5%AE%8C%E6%95%B4%E7%89%88%E6%95%B0%E6%8D%AE\GDR3855-Hylocereus_undulatus_Britt-12-RNAseq_result\4_Function\2_Group_Diff_Function\UP_DOWN\GO\NL-VS-L1.F.html#gene172) | peptide transporter activity | 2 (0.16%) | 4 (0.12%) | 0.446750 | 0.980162 |
| 173 | [GO:0016837](file:///E:\2018-7-3%E7%81%AB%E9%BE%99%E6%9E%9C%E8%BD%AC%E5%BD%95%E7%BB%84%E6%B5%8B%E5%BA%8F\%E5%AE%8C%E6%95%B4%E7%89%88%E6%95%B0%E6%8D%AE\GDR3855-Hylocereus_undulatus_Britt-12-RNAseq_result\4_Function\2_Group_Diff_Function\UP_DOWN\GO\NL-VS-L1.F.html#gene173) | carbon-oxygen lyase activity, acting on polysaccharides | 2 (0.16%) | 4 (0.12%) | 0.446750 | 0.980162 |
| 174 | [GO:0016884](file:///E:\2018-7-3%E7%81%AB%E9%BE%99%E6%9E%9C%E8%BD%AC%E5%BD%95%E7%BB%84%E6%B5%8B%E5%BA%8F\%E5%AE%8C%E6%95%B4%E7%89%88%E6%95%B0%E6%8D%AE\GDR3855-Hylocereus_undulatus_Britt-12-RNAseq_result\4_Function\2_Group_Diff_Function\UP_DOWN\GO\NL-VS-L1.F.html#gene174) | carbon-nitrogen ligase activity, with glutamine as amido-N-donor | 2 (0.16%) | 4 (0.12%) | 0.446750 | 0.980162 |
| 175 | [GO:0046030](file:///E:\2018-7-3%E7%81%AB%E9%BE%99%E6%9E%9C%E8%BD%AC%E5%BD%95%E7%BB%84%E6%B5%8B%E5%BA%8F\%E5%AE%8C%E6%95%B4%E7%89%88%E6%95%B0%E6%8D%AE\GDR3855-Hylocereus_undulatus_Britt-12-RNAseq_result\4_Function\2_Group_Diff_Function\UP_DOWN\GO\NL-VS-L1.F.html#gene175) | inositol trisphosphate phosphatase activity | 2 (0.16%) | 4 (0.12%) | 0.446750 | 0.980162 |
| 176 | [GO:0052743](file:///E:\2018-7-3%E7%81%AB%E9%BE%99%E6%9E%9C%E8%BD%AC%E5%BD%95%E7%BB%84%E6%B5%8B%E5%BA%8F\%E5%AE%8C%E6%95%B4%E7%89%88%E6%95%B0%E6%8D%AE\GDR3855-Hylocereus_undulatus_Britt-12-RNAseq_result\4_Function\2_Group_Diff_Function\UP_DOWN\GO\NL-VS-L1.F.html#gene176) | inositol tetrakisphosphate phosphatase activity | 2 (0.16%) | 4 (0.12%) | 0.446750 | 0.980162 |
| 177 | [GO:0015291](file:///E:\2018-7-3%E7%81%AB%E9%BE%99%E6%9E%9C%E8%BD%AC%E5%BD%95%E7%BB%84%E6%B5%8B%E5%BA%8F\%E5%AE%8C%E6%95%B4%E7%89%88%E6%95%B0%E6%8D%AE\GDR3855-Hylocereus_undulatus_Britt-12-RNAseq_result\4_Function\2_Group_Diff_Function\UP_DOWN\GO\NL-VS-L1.F.html#gene177) | secondary active transmembrane transporter activity | 10 (0.82%) | 26 (0.76%) | 0.450183 | 0.980162 |
| 178 | [GO:0001071](file:///E:\2018-7-3%E7%81%AB%E9%BE%99%E6%9E%9C%E8%BD%AC%E5%BD%95%E7%BB%84%E6%B5%8B%E5%BA%8F\%E5%AE%8C%E6%95%B4%E7%89%88%E6%95%B0%E6%8D%AE\GDR3855-Hylocereus_undulatus_Britt-12-RNAseq_result\4_Function\2_Group_Diff_Function\UP_DOWN\GO\NL-VS-L1.F.html#gene178) | nucleic acid binding transcription factor activity | 15 (1.23%) | 40 (1.17%) | 0.456681 | 0.980162 |
| 179 | [GO:0016829](file:///E:\2018-7-3%E7%81%AB%E9%BE%99%E6%9E%9C%E8%BD%AC%E5%BD%95%E7%BB%84%E6%B5%8B%E5%BA%8F\%E5%AE%8C%E6%95%B4%E7%89%88%E6%95%B0%E6%8D%AE\GDR3855-Hylocereus_undulatus_Britt-12-RNAseq_result\4_Function\2_Group_Diff_Function\UP_DOWN\GO\NL-VS-L1.F.html#gene179) | lyase activity | 35 (2.87%) | 96 (2.8%) | 0.463881 | 0.980162 |
| 180 | [GO:0060089](file:///E:\2018-7-3%E7%81%AB%E9%BE%99%E6%9E%9C%E8%BD%AC%E5%BD%95%E7%BB%84%E6%B5%8B%E5%BA%8F\%E5%AE%8C%E6%95%B4%E7%89%88%E6%95%B0%E6%8D%AE\GDR3855-Hylocereus_undulatus_Britt-12-RNAseq_result\4_Function\2_Group_Diff_Function\UP_DOWN\GO\NL-VS-L1.F.html#gene180) | molecular transducer activity | 7 (0.57%) | 18 (0.52%) | 0.470554 | 0.980162 |
| 181 | [GO:0004611](file:///E:\2018-7-3%E7%81%AB%E9%BE%99%E6%9E%9C%E8%BD%AC%E5%BD%95%E7%BB%84%E6%B5%8B%E5%BA%8F\%E5%AE%8C%E6%95%B4%E7%89%88%E6%95%B0%E6%8D%AE\GDR3855-Hylocereus_undulatus_Britt-12-RNAseq_result\4_Function\2_Group_Diff_Function\UP_DOWN\GO\NL-VS-L1.F.html#gene181) | phosphoenolpyruvate carboxykinase activity | 3 (0.25%) | 7 (0.2%) | 0.480322 | 0.980162 |
| 182 | [GO:0004872](file:///E:\2018-7-3%E7%81%AB%E9%BE%99%E6%9E%9C%E8%BD%AC%E5%BD%95%E7%BB%84%E6%B5%8B%E5%BA%8F\%E5%AE%8C%E6%95%B4%E7%89%88%E6%95%B0%E6%8D%AE\GDR3855-Hylocereus_undulatus_Britt-12-RNAseq_result\4_Function\2_Group_Diff_Function\UP_DOWN\GO\NL-VS-L1.F.html#gene182) | receptor activity | 3 (0.25%) | 7 (0.2%) | 0.480322 | 0.980162 |
| 183 | [GO:0008081](file:///E:\2018-7-3%E7%81%AB%E9%BE%99%E6%9E%9C%E8%BD%AC%E5%BD%95%E7%BB%84%E6%B5%8B%E5%BA%8F\%E5%AE%8C%E6%95%B4%E7%89%88%E6%95%B0%E6%8D%AE\GDR3855-Hylocereus_undulatus_Britt-12-RNAseq_result\4_Function\2_Group_Diff_Function\UP_DOWN\GO\NL-VS-L1.F.html#gene183) | phosphoric diester hydrolase activity | 3 (0.25%) | 7 (0.2%) | 0.480322 | 0.980162 |
| 184 | [GO:0016776](file:///E:\2018-7-3%E7%81%AB%E9%BE%99%E6%9E%9C%E8%BD%AC%E5%BD%95%E7%BB%84%E6%B5%8B%E5%BA%8F\%E5%AE%8C%E6%95%B4%E7%89%88%E6%95%B0%E6%8D%AE\GDR3855-Hylocereus_undulatus_Britt-12-RNAseq_result\4_Function\2_Group_Diff_Function\UP_DOWN\GO\NL-VS-L1.F.html#gene184) | phosphotransferase activity, phosphate group as acceptor | 3 (0.25%) | 7 (0.2%) | 0.480322 | 0.980162 |
| 185 | [GO:0016811](file:///E:\2018-7-3%E7%81%AB%E9%BE%99%E6%9E%9C%E8%BD%AC%E5%BD%95%E7%BB%84%E6%B5%8B%E5%BA%8F\%E5%AE%8C%E6%95%B4%E7%89%88%E6%95%B0%E6%8D%AE\GDR3855-Hylocereus_undulatus_Britt-12-RNAseq_result\4_Function\2_Group_Diff_Function\UP_DOWN\GO\NL-VS-L1.F.html#gene185) | hydrolase activity, acting on carbon-nitrogen (but not peptide) bonds, in linear amides | 3 (0.25%) | 7 (0.2%) | 0.480322 | 0.980162 |
| 186 | [GO:0016846](file:///E:\2018-7-3%E7%81%AB%E9%BE%99%E6%9E%9C%E8%BD%AC%E5%BD%95%E7%BB%84%E6%B5%8B%E5%BA%8F\%E5%AE%8C%E6%95%B4%E7%89%88%E6%95%B0%E6%8D%AE\GDR3855-Hylocereus_undulatus_Britt-12-RNAseq_result\4_Function\2_Group_Diff_Function\UP_DOWN\GO\NL-VS-L1.F.html#gene186) | carbon-sulfur lyase activity | 3 (0.25%) | 7 (0.2%) | 0.480322 | 0.980162 |
| 187 | [GO:0019200](file:///E:\2018-7-3%E7%81%AB%E9%BE%99%E6%9E%9C%E8%BD%AC%E5%BD%95%E7%BB%84%E6%B5%8B%E5%BA%8F\%E5%AE%8C%E6%95%B4%E7%89%88%E6%95%B0%E6%8D%AE\GDR3855-Hylocereus_undulatus_Britt-12-RNAseq_result\4_Function\2_Group_Diff_Function\UP_DOWN\GO\NL-VS-L1.F.html#gene187) | carbohydrate kinase activity | 3 (0.25%) | 7 (0.2%) | 0.480322 | 0.980162 |
| 188 | [GO:0016747](file:///E:\2018-7-3%E7%81%AB%E9%BE%99%E6%9E%9C%E8%BD%AC%E5%BD%95%E7%BB%84%E6%B5%8B%E5%BA%8F\%E5%AE%8C%E6%95%B4%E7%89%88%E6%95%B0%E6%8D%AE\GDR3855-Hylocereus_undulatus_Britt-12-RNAseq_result\4_Function\2_Group_Diff_Function\UP_DOWN\GO\NL-VS-L1.F.html#gene188) | transferase activity, transferring acyl groups other than amino-acyl groups | 13 (1.07%) | 35 (1.02%) | 0.485002 | 0.980162 |
| 189 | [GO:0016791](file:///E:\2018-7-3%E7%81%AB%E9%BE%99%E6%9E%9C%E8%BD%AC%E5%BD%95%E7%BB%84%E6%B5%8B%E5%BA%8F\%E5%AE%8C%E6%95%B4%E7%89%88%E6%95%B0%E6%8D%AE\GDR3855-Hylocereus_undulatus_Britt-12-RNAseq_result\4_Function\2_Group_Diff_Function\UP_DOWN\GO\NL-VS-L1.F.html#gene189) | phosphatase activity | 24 (1.97%) | 66 (1.92%) | 0.491188 | 0.980162 |
| 190 | [GO:0022890](file:///E:\2018-7-3%E7%81%AB%E9%BE%99%E6%9E%9C%E8%BD%AC%E5%BD%95%E7%BB%84%E6%B5%8B%E5%BA%8F\%E5%AE%8C%E6%95%B4%E7%89%88%E6%95%B0%E6%8D%AE\GDR3855-Hylocereus_undulatus_Britt-12-RNAseq_result\4_Function\2_Group_Diff_Function\UP_DOWN\GO\NL-VS-L1.F.html#gene190) | inorganic cation transmembrane transporter activity | 24 (1.97%) | 66 (1.92%) | 0.491188 | 0.980162 |
| 191 | [GO:0016759](file:///E:\2018-7-3%E7%81%AB%E9%BE%99%E6%9E%9C%E8%BD%AC%E5%BD%95%E7%BB%84%E6%B5%8B%E5%BA%8F\%E5%AE%8C%E6%95%B4%E7%89%88%E6%95%B0%E6%8D%AE\GDR3855-Hylocereus_undulatus_Britt-12-RNAseq_result\4_Function\2_Group_Diff_Function\UP_DOWN\GO\NL-VS-L1.F.html#gene191) | cellulose synthase activity | 4 (0.33%) | 10 (0.29%) | 0.501101 | 0.980162 |
| 192 | [GO:0016854](file:///E:\2018-7-3%E7%81%AB%E9%BE%99%E6%9E%9C%E8%BD%AC%E5%BD%95%E7%BB%84%E6%B5%8B%E5%BA%8F\%E5%AE%8C%E6%95%B4%E7%89%88%E6%95%B0%E6%8D%AE\GDR3855-Hylocereus_undulatus_Britt-12-RNAseq_result\4_Function\2_Group_Diff_Function\UP_DOWN\GO\NL-VS-L1.F.html#gene192) | racemase and epimerase activity | 4 (0.33%) | 10 (0.29%) | 0.501101 | 0.980162 |
| 193 | [GO:0034061](file:///E:\2018-7-3%E7%81%AB%E9%BE%99%E6%9E%9C%E8%BD%AC%E5%BD%95%E7%BB%84%E6%B5%8B%E5%BA%8F\%E5%AE%8C%E6%95%B4%E7%89%88%E6%95%B0%E6%8D%AE\GDR3855-Hylocereus_undulatus_Britt-12-RNAseq_result\4_Function\2_Group_Diff_Function\UP_DOWN\GO\NL-VS-L1.F.html#gene193) | DNA polymerase activity | 5 (0.41%) | 13 (0.38%) | 0.516427 | 0.980162 |
| 194 | [GO:0008194](file:///E:\2018-7-3%E7%81%AB%E9%BE%99%E6%9E%9C%E8%BD%AC%E5%BD%95%E7%BB%84%E6%B5%8B%E5%BA%8F\%E5%AE%8C%E6%95%B4%E7%89%88%E6%95%B0%E6%8D%AE\GDR3855-Hylocereus_undulatus_Britt-12-RNAseq_result\4_Function\2_Group_Diff_Function\UP_DOWN\GO\NL-VS-L1.F.html#gene194) | UDP-glycosyltransferase activity | 18 (1.48%) | 50 (1.46%) | 0.526869 | 0.980162 |
| 195 | [GO:0034062](file:///E:\2018-7-3%E7%81%AB%E9%BE%99%E6%9E%9C%E8%BD%AC%E5%BD%95%E7%BB%84%E6%B5%8B%E5%BA%8F\%E5%AE%8C%E6%95%B4%E7%89%88%E6%95%B0%E6%8D%AE\GDR3855-Hylocereus_undulatus_Britt-12-RNAseq_result\4_Function\2_Group_Diff_Function\UP_DOWN\GO\NL-VS-L1.F.html#gene195) | RNA polymerase activity | 6 (0.49%) | 16 (0.47%) | 0.528749 | 0.980162 |
| 196 | [GO:0016746](file:///E:\2018-7-3%E7%81%AB%E9%BE%99%E6%9E%9C%E8%BD%AC%E5%BD%95%E7%BB%84%E6%B5%8B%E5%BA%8F\%E5%AE%8C%E6%95%B4%E7%89%88%E6%95%B0%E6%8D%AE\GDR3855-Hylocereus_undulatus_Britt-12-RNAseq_result\4_Function\2_Group_Diff_Function\UP_DOWN\GO\NL-VS-L1.F.html#gene196) | transferase activity, transferring acyl groups | 21 (1.72%) | 59 (1.72%) | 0.546557 | 0.980162 |
| 197 | [GO:0003723](file:///E:\2018-7-3%E7%81%AB%E9%BE%99%E6%9E%9C%E8%BD%AC%E5%BD%95%E7%BB%84%E6%B5%8B%E5%BA%8F\%E5%AE%8C%E6%95%B4%E7%89%88%E6%95%B0%E6%8D%AE\GDR3855-Hylocereus_undulatus_Britt-12-RNAseq_result\4_Function\2_Group_Diff_Function\UP_DOWN\GO\NL-VS-L1.F.html#gene197) | RNA binding | 24 (1.97%) | 68 (1.98%) | 0.563672 | 0.980162 |
| 198 | [GO:0003779](file:///E:\2018-7-3%E7%81%AB%E9%BE%99%E6%9E%9C%E8%BD%AC%E5%BD%95%E7%BB%84%E6%B5%8B%E5%BA%8F\%E5%AE%8C%E6%95%B4%E7%89%88%E6%95%B0%E6%8D%AE\GDR3855-Hylocereus_undulatus_Britt-12-RNAseq_result\4_Function\2_Group_Diff_Function\UP_DOWN\GO\NL-VS-L1.F.html#gene198) | actin binding | 2 (0.16%) | 5 (0.15%) | 0.582181 | 0.980162 |
| 199 | [GO:0004180](file:///E:\2018-7-3%E7%81%AB%E9%BE%99%E6%9E%9C%E8%BD%AC%E5%BD%95%E7%BB%84%E6%B5%8B%E5%BA%8F\%E5%AE%8C%E6%95%B4%E7%89%88%E6%95%B0%E6%8D%AE\GDR3855-Hylocereus_undulatus_Britt-12-RNAseq_result\4_Function\2_Group_Diff_Function\UP_DOWN\GO\NL-VS-L1.F.html#gene199) | carboxypeptidase activity | 2 (0.16%) | 5 (0.15%) | 0.582181 | 0.980162 |
| 200 | [GO:0004601](file:///E:\2018-7-3%E7%81%AB%E9%BE%99%E6%9E%9C%E8%BD%AC%E5%BD%95%E7%BB%84%E6%B5%8B%E5%BA%8F\%E5%AE%8C%E6%95%B4%E7%89%88%E6%95%B0%E6%8D%AE\GDR3855-Hylocereus_undulatus_Britt-12-RNAseq_result\4_Function\2_Group_Diff_Function\UP_DOWN\GO\NL-VS-L1.F.html#gene200) | peroxidase activity | 2 (0.16%) | 5 (0.15%) | 0.582181 | 0.980162 |
| 201 | [GO:0010333](file:///E:\2018-7-3%E7%81%AB%E9%BE%99%E6%9E%9C%E8%BD%AC%E5%BD%95%E7%BB%84%E6%B5%8B%E5%BA%8F\%E5%AE%8C%E6%95%B4%E7%89%88%E6%95%B0%E6%8D%AE\GDR3855-Hylocereus_undulatus_Britt-12-RNAseq_result\4_Function\2_Group_Diff_Function\UP_DOWN\GO\NL-VS-L1.F.html#gene201) | terpene synthase activity | 2 (0.16%) | 5 (0.15%) | 0.582181 | 0.980162 |
| 202 | [GO:0015215](file:///E:\2018-7-3%E7%81%AB%E9%BE%99%E6%9E%9C%E8%BD%AC%E5%BD%95%E7%BB%84%E6%B5%8B%E5%BA%8F\%E5%AE%8C%E6%95%B4%E7%89%88%E6%95%B0%E6%8D%AE\GDR3855-Hylocereus_undulatus_Britt-12-RNAseq_result\4_Function\2_Group_Diff_Function\UP_DOWN\GO\NL-VS-L1.F.html#gene202) | nucleotide transmembrane transporter activity | 2 (0.16%) | 5 (0.15%) | 0.582181 | 0.980162 |
| 203 | [GO:0016684](file:///E:\2018-7-3%E7%81%AB%E9%BE%99%E6%9E%9C%E8%BD%AC%E5%BD%95%E7%BB%84%E6%B5%8B%E5%BA%8F\%E5%AE%8C%E6%95%B4%E7%89%88%E6%95%B0%E6%8D%AE\GDR3855-Hylocereus_undulatus_Britt-12-RNAseq_result\4_Function\2_Group_Diff_Function\UP_DOWN\GO\NL-VS-L1.F.html#gene203) | oxidoreductase activity, acting on peroxide as acceptor | 2 (0.16%) | 5 (0.15%) | 0.582181 | 0.980162 |
| 204 | [GO:0032403](file:///E:\2018-7-3%E7%81%AB%E9%BE%99%E6%9E%9C%E8%BD%AC%E5%BD%95%E7%BB%84%E6%B5%8B%E5%BA%8F\%E5%AE%8C%E6%95%B4%E7%89%88%E6%95%B0%E6%8D%AE\GDR3855-Hylocereus_undulatus_Britt-12-RNAseq_result\4_Function\2_Group_Diff_Function\UP_DOWN\GO\NL-VS-L1.F.html#gene204) | protein complex binding | 2 (0.16%) | 5 (0.15%) | 0.582181 | 0.980162 |
| 205 | [GO:0000988](file:///E:\2018-7-3%E7%81%AB%E9%BE%99%E6%9E%9C%E8%BD%AC%E5%BD%95%E7%BB%84%E6%B5%8B%E5%BA%8F\%E5%AE%8C%E6%95%B4%E7%89%88%E6%95%B0%E6%8D%AE\GDR3855-Hylocereus_undulatus_Britt-12-RNAseq_result\4_Function\2_Group_Diff_Function\UP_DOWN\GO\NL-VS-L1.F.html#gene205) | transcription factor activity, protein binding | 1 (0.08%) | 2 (0.06%) | 0.584658 | 0.980162 |
| 206 | [GO:0004659](file:///E:\2018-7-3%E7%81%AB%E9%BE%99%E6%9E%9C%E8%BD%AC%E5%BD%95%E7%BB%84%E6%B5%8B%E5%BA%8F\%E5%AE%8C%E6%95%B4%E7%89%88%E6%95%B0%E6%8D%AE\GDR3855-Hylocereus_undulatus_Britt-12-RNAseq_result\4_Function\2_Group_Diff_Function\UP_DOWN\GO\NL-VS-L1.F.html#gene206) | prenyltransferase activity | 1 (0.08%) | 2 (0.06%) | 0.584658 | 0.980162 |
| 207 | [GO:0005275](file:///E:\2018-7-3%E7%81%AB%E9%BE%99%E6%9E%9C%E8%BD%AC%E5%BD%95%E7%BB%84%E6%B5%8B%E5%BA%8F\%E5%AE%8C%E6%95%B4%E7%89%88%E6%95%B0%E6%8D%AE\GDR3855-Hylocereus_undulatus_Britt-12-RNAseq_result\4_Function\2_Group_Diff_Function\UP_DOWN\GO\NL-VS-L1.F.html#gene207) | amine transmembrane transporter activity | 1 (0.08%) | 2 (0.06%) | 0.584658 | 0.980162 |
| 208 | [GO:0005319](file:///E:\2018-7-3%E7%81%AB%E9%BE%99%E6%9E%9C%E8%BD%AC%E5%BD%95%E7%BB%84%E6%B5%8B%E5%BA%8F\%E5%AE%8C%E6%95%B4%E7%89%88%E6%95%B0%E6%8D%AE\GDR3855-Hylocereus_undulatus_Britt-12-RNAseq_result\4_Function\2_Group_Diff_Function\UP_DOWN\GO\NL-VS-L1.F.html#gene208) | lipid transporter activity | 1 (0.08%) | 2 (0.06%) | 0.584658 | 0.980162 |
| 209 | [GO:0005337](file:///E:\2018-7-3%E7%81%AB%E9%BE%99%E6%9E%9C%E8%BD%AC%E5%BD%95%E7%BB%84%E6%B5%8B%E5%BA%8F\%E5%AE%8C%E6%95%B4%E7%89%88%E6%95%B0%E6%8D%AE\GDR3855-Hylocereus_undulatus_Britt-12-RNAseq_result\4_Function\2_Group_Diff_Function\UP_DOWN\GO\NL-VS-L1.F.html#gene209) | nucleoside transmembrane transporter activity | 1 (0.08%) | 2 (0.06%) | 0.584658 | 0.980162 |
| 210 | [GO:0005346](file:///E:\2018-7-3%E7%81%AB%E9%BE%99%E6%9E%9C%E8%BD%AC%E5%BD%95%E7%BB%84%E6%B5%8B%E5%BA%8F\%E5%AE%8C%E6%95%B4%E7%89%88%E6%95%B0%E6%8D%AE\GDR3855-Hylocereus_undulatus_Britt-12-RNAseq_result\4_Function\2_Group_Diff_Function\UP_DOWN\GO\NL-VS-L1.F.html#gene210) | purine ribonucleotide transmembrane transporter activity | 1 (0.08%) | 2 (0.06%) | 0.584658 | 0.980162 |
| 211 | [GO:0005347](file:///E:\2018-7-3%E7%81%AB%E9%BE%99%E6%9E%9C%E8%BD%AC%E5%BD%95%E7%BB%84%E6%B5%8B%E5%BA%8F\%E5%AE%8C%E6%95%B4%E7%89%88%E6%95%B0%E6%8D%AE\GDR3855-Hylocereus_undulatus_Britt-12-RNAseq_result\4_Function\2_Group_Diff_Function\UP_DOWN\GO\NL-VS-L1.F.html#gene211) | ATP transmembrane transporter activity | 1 (0.08%) | 2 (0.06%) | 0.584658 | 0.980162 |
| 212 | [GO:0005402](file:///E:\2018-7-3%E7%81%AB%E9%BE%99%E6%9E%9C%E8%BD%AC%E5%BD%95%E7%BB%84%E6%B5%8B%E5%BA%8F\%E5%AE%8C%E6%95%B4%E7%89%88%E6%95%B0%E6%8D%AE\GDR3855-Hylocereus_undulatus_Britt-12-RNAseq_result\4_Function\2_Group_Diff_Function\UP_DOWN\GO\NL-VS-L1.F.html#gene212) | cation:sugar symporter activity | 1 (0.08%) | 2 (0.06%) | 0.584658 | 0.980162 |
| 213 | [GO:0005548](file:///E:\2018-7-3%E7%81%AB%E9%BE%99%E6%9E%9C%E8%BD%AC%E5%BD%95%E7%BB%84%E6%B5%8B%E5%BA%8F\%E5%AE%8C%E6%95%B4%E7%89%88%E6%95%B0%E6%8D%AE\GDR3855-Hylocereus_undulatus_Britt-12-RNAseq_result\4_Function\2_Group_Diff_Function\UP_DOWN\GO\NL-VS-L1.F.html#gene213) | phospholipid transporter activity | 1 (0.08%) | 2 (0.06%) | 0.584658 | 0.980162 |
| 214 | [GO:0008094](file:///E:\2018-7-3%E7%81%AB%E9%BE%99%E6%9E%9C%E8%BD%AC%E5%BD%95%E7%BB%84%E6%B5%8B%E5%BA%8F\%E5%AE%8C%E6%95%B4%E7%89%88%E6%95%B0%E6%8D%AE\GDR3855-Hylocereus_undulatus_Britt-12-RNAseq_result\4_Function\2_Group_Diff_Function\UP_DOWN\GO\NL-VS-L1.F.html#gene214) | DNA-dependent ATPase activity | 1 (0.08%) | 2 (0.06%) | 0.584658 | 0.980162 |
| 215 | [GO:0008234](file:///E:\2018-7-3%E7%81%AB%E9%BE%99%E6%9E%9C%E8%BD%AC%E5%BD%95%E7%BB%84%E6%B5%8B%E5%BA%8F\%E5%AE%8C%E6%95%B4%E7%89%88%E6%95%B0%E6%8D%AE\GDR3855-Hylocereus_undulatus_Britt-12-RNAseq_result\4_Function\2_Group_Diff_Function\UP_DOWN\GO\NL-VS-L1.F.html#gene215) | cysteine-type peptidase activity | 1 (0.08%) | 2 (0.06%) | 0.584658 | 0.980162 |
| 216 | [GO:0008417](file:///E:\2018-7-3%E7%81%AB%E9%BE%99%E6%9E%9C%E8%BD%AC%E5%BD%95%E7%BB%84%E6%B5%8B%E5%BA%8F\%E5%AE%8C%E6%95%B4%E7%89%88%E6%95%B0%E6%8D%AE\GDR3855-Hylocereus_undulatus_Britt-12-RNAseq_result\4_Function\2_Group_Diff_Function\UP_DOWN\GO\NL-VS-L1.F.html#gene216) | fucosyltransferase activity | 1 (0.08%) | 2 (0.06%) | 0.584658 | 0.980162 |
| 217 | [GO:0009975](file:///E:\2018-7-3%E7%81%AB%E9%BE%99%E6%9E%9C%E8%BD%AC%E5%BD%95%E7%BB%84%E6%B5%8B%E5%BA%8F\%E5%AE%8C%E6%95%B4%E7%89%88%E6%95%B0%E6%8D%AE\GDR3855-Hylocereus_undulatus_Britt-12-RNAseq_result\4_Function\2_Group_Diff_Function\UP_DOWN\GO\NL-VS-L1.F.html#gene217) | cyclase activity | 1 (0.08%) | 2 (0.06%) | 0.584658 | 0.980162 |
| 218 | [GO:0010436](file:///E:\2018-7-3%E7%81%AB%E9%BE%99%E6%9E%9C%E8%BD%AC%E5%BD%95%E7%BB%84%E6%B5%8B%E5%BA%8F\%E5%AE%8C%E6%95%B4%E7%89%88%E6%95%B0%E6%8D%AE\GDR3855-Hylocereus_undulatus_Britt-12-RNAseq_result\4_Function\2_Group_Diff_Function\UP_DOWN\GO\NL-VS-L1.F.html#gene218) | carotenoid dioxygenase activity | 1 (0.08%) | 2 (0.06%) | 0.584658 | 0.980162 |
| 219 | [GO:0015211](file:///E:\2018-7-3%E7%81%AB%E9%BE%99%E6%9E%9C%E8%BD%AC%E5%BD%95%E7%BB%84%E6%B5%8B%E5%BA%8F\%E5%AE%8C%E6%95%B4%E7%89%88%E6%95%B0%E6%8D%AE\GDR3855-Hylocereus_undulatus_Britt-12-RNAseq_result\4_Function\2_Group_Diff_Function\UP_DOWN\GO\NL-VS-L1.F.html#gene219) | purine nucleoside transmembrane transporter activity | 1 (0.08%) | 2 (0.06%) | 0.584658 | 0.980162 |
| 220 | [GO:0015293](file:///E:\2018-7-3%E7%81%AB%E9%BE%99%E6%9E%9C%E8%BD%AC%E5%BD%95%E7%BB%84%E6%B5%8B%E5%BA%8F\%E5%AE%8C%E6%95%B4%E7%89%88%E6%95%B0%E6%8D%AE\GDR3855-Hylocereus_undulatus_Britt-12-RNAseq_result\4_Function\2_Group_Diff_Function\UP_DOWN\GO\NL-VS-L1.F.html#gene220) | symporter activity | 1 (0.08%) | 2 (0.06%) | 0.584658 | 0.980162 |
| 221 | [GO:0015294](file:///E:\2018-7-3%E7%81%AB%E9%BE%99%E6%9E%9C%E8%BD%AC%E5%BD%95%E7%BB%84%E6%B5%8B%E5%BA%8F\%E5%AE%8C%E6%95%B4%E7%89%88%E6%95%B0%E6%8D%AE\GDR3855-Hylocereus_undulatus_Britt-12-RNAseq_result\4_Function\2_Group_Diff_Function\UP_DOWN\GO\NL-VS-L1.F.html#gene221) | solute:cation symporter activity | 1 (0.08%) | 2 (0.06%) | 0.584658 | 0.980162 |
| 222 | [GO:0016211](file:///E:\2018-7-3%E7%81%AB%E9%BE%99%E6%9E%9C%E8%BD%AC%E5%BD%95%E7%BB%84%E6%B5%8B%E5%BA%8F\%E5%AE%8C%E6%95%B4%E7%89%88%E6%95%B0%E6%8D%AE\GDR3855-Hylocereus_undulatus_Britt-12-RNAseq_result\4_Function\2_Group_Diff_Function\UP_DOWN\GO\NL-VS-L1.F.html#gene222) | ammonia ligase activity | 1 (0.08%) | 2 (0.06%) | 0.584658 | 0.980162 |
| 223 | [GO:0016307](file:///E:\2018-7-3%E7%81%AB%E9%BE%99%E6%9E%9C%E8%BD%AC%E5%BD%95%E7%BB%84%E6%B5%8B%E5%BA%8F\%E5%AE%8C%E6%95%B4%E7%89%88%E6%95%B0%E6%8D%AE\GDR3855-Hylocereus_undulatus_Britt-12-RNAseq_result\4_Function\2_Group_Diff_Function\UP_DOWN\GO\NL-VS-L1.F.html#gene223) | phosphatidylinositol phosphate kinase activity | 1 (0.08%) | 2 (0.06%) | 0.584658 | 0.980162 |
| 224 | [GO:0016642](file:///E:\2018-7-3%E7%81%AB%E9%BE%99%E6%9E%9C%E8%BD%AC%E5%BD%95%E7%BB%84%E6%B5%8B%E5%BA%8F\%E5%AE%8C%E6%95%B4%E7%89%88%E6%95%B0%E6%8D%AE\GDR3855-Hylocereus_undulatus_Britt-12-RNAseq_result\4_Function\2_Group_Diff_Function\UP_DOWN\GO\NL-VS-L1.F.html#gene224) | oxidoreductase activity, acting on the CH-NH2 group of donors, disulfide as acceptor | 1 (0.08%) | 2 (0.06%) | 0.584658 | 0.980162 |
| 225 | [GO:0016664](file:///E:\2018-7-3%E7%81%AB%E9%BE%99%E6%9E%9C%E8%BD%AC%E5%BD%95%E7%BB%84%E6%B5%8B%E5%BA%8F\%E5%AE%8C%E6%95%B4%E7%89%88%E6%95%B0%E6%8D%AE\GDR3855-Hylocereus_undulatus_Britt-12-RNAseq_result\4_Function\2_Group_Diff_Function\UP_DOWN\GO\NL-VS-L1.F.html#gene225) | oxidoreductase activity, acting on other nitrogenous compounds as donors, iron-sulfur protein as acceptor | 1 (0.08%) | 2 (0.06%) | 0.584658 | 0.980162 |
| 226 | [GO:0016673](file:///E:\2018-7-3%E7%81%AB%E9%BE%99%E6%9E%9C%E8%BD%AC%E5%BD%95%E7%BB%84%E6%B5%8B%E5%BA%8F\%E5%AE%8C%E6%95%B4%E7%89%88%E6%95%B0%E6%8D%AE\GDR3855-Hylocereus_undulatus_Britt-12-RNAseq_result\4_Function\2_Group_Diff_Function\UP_DOWN\GO\NL-VS-L1.F.html#gene226) | oxidoreductase activity, acting on a sulfur group of donors, iron-sulfur protein as acceptor | 1 (0.08%) | 2 (0.06%) | 0.584658 | 0.980162 |
| 227 | [GO:0016713](file:///E:\2018-7-3%E7%81%AB%E9%BE%99%E6%9E%9C%E8%BD%AC%E5%BD%95%E7%BB%84%E6%B5%8B%E5%BA%8F\%E5%AE%8C%E6%95%B4%E7%89%88%E6%95%B0%E6%8D%AE\GDR3855-Hylocereus_undulatus_Britt-12-RNAseq_result\4_Function\2_Group_Diff_Function\UP_DOWN\GO\NL-VS-L1.F.html#gene227) | oxidoreductase activity, acting on paired donors, with incorporation or reduction of molecular oxygen, reduced iron-sulfur protein as one donor, and incorporation of one atom of oxygen | 1 (0.08%) | 2 (0.06%) | 0.584658 | 0.980162 |
| 228 | [GO:0016840](file:///E:\2018-7-3%E7%81%AB%E9%BE%99%E6%9E%9C%E8%BD%AC%E5%BD%95%E7%BB%84%E6%B5%8B%E5%BA%8F\%E5%AE%8C%E6%95%B4%E7%89%88%E6%95%B0%E6%8D%AE\GDR3855-Hylocereus_undulatus_Britt-12-RNAseq_result\4_Function\2_Group_Diff_Function\UP_DOWN\GO\NL-VS-L1.F.html#gene228) | carbon-nitrogen lyase activity | 1 (0.08%) | 2 (0.06%) | 0.584658 | 0.980162 |
| 229 | [GO:0016880](file:///E:\2018-7-3%E7%81%AB%E9%BE%99%E6%9E%9C%E8%BD%AC%E5%BD%95%E7%BB%84%E6%B5%8B%E5%BA%8F\%E5%AE%8C%E6%95%B4%E7%89%88%E6%95%B0%E6%8D%AE\GDR3855-Hylocereus_undulatus_Britt-12-RNAseq_result\4_Function\2_Group_Diff_Function\UP_DOWN\GO\NL-VS-L1.F.html#gene229) | acid-ammonia (or amide) ligase activity | 1 (0.08%) | 2 (0.06%) | 0.584658 | 0.980162 |
| 230 | [GO:0016891](file:///E:\2018-7-3%E7%81%AB%E9%BE%99%E6%9E%9C%E8%BD%AC%E5%BD%95%E7%BB%84%E6%B5%8B%E5%BA%8F\%E5%AE%8C%E6%95%B4%E7%89%88%E6%95%B0%E6%8D%AE\GDR3855-Hylocereus_undulatus_Britt-12-RNAseq_result\4_Function\2_Group_Diff_Function\UP_DOWN\GO\NL-VS-L1.F.html#gene230) | endoribonuclease activity, producing 5'-phosphomonoesters | 1 (0.08%) | 2 (0.06%) | 0.584658 | 0.980162 |
| 231 | [GO:0017069](file:///E:\2018-7-3%E7%81%AB%E9%BE%99%E6%9E%9C%E8%BD%AC%E5%BD%95%E7%BB%84%E6%B5%8B%E5%BA%8F\%E5%AE%8C%E6%95%B4%E7%89%88%E6%95%B0%E6%8D%AE\GDR3855-Hylocereus_undulatus_Britt-12-RNAseq_result\4_Function\2_Group_Diff_Function\UP_DOWN\GO\NL-VS-L1.F.html#gene231) | snRNA binding | 1 (0.08%) | 2 (0.06%) | 0.584658 | 0.980162 |
| 232 | [GO:0019843](file:///E:\2018-7-3%E7%81%AB%E9%BE%99%E6%9E%9C%E8%BD%AC%E5%BD%95%E7%BB%84%E6%B5%8B%E5%BA%8F\%E5%AE%8C%E6%95%B4%E7%89%88%E6%95%B0%E6%8D%AE\GDR3855-Hylocereus_undulatus_Britt-12-RNAseq_result\4_Function\2_Group_Diff_Function\UP_DOWN\GO\NL-VS-L1.F.html#gene232) | rRNA binding | 1 (0.08%) | 2 (0.06%) | 0.584658 | 0.980162 |
| 233 | [GO:0032553](file:///E:\2018-7-3%E7%81%AB%E9%BE%99%E6%9E%9C%E8%BD%AC%E5%BD%95%E7%BB%84%E6%B5%8B%E5%BA%8F\%E5%AE%8C%E6%95%B4%E7%89%88%E6%95%B0%E6%8D%AE\GDR3855-Hylocereus_undulatus_Britt-12-RNAseq_result\4_Function\2_Group_Diff_Function\UP_DOWN\GO\NL-VS-L1.F.html#gene233) | ribonucleotide binding | 1 (0.08%) | 2 (0.06%) | 0.584658 | 0.980162 |
| 234 | [GO:0046920](file:///E:\2018-7-3%E7%81%AB%E9%BE%99%E6%9E%9C%E8%BD%AC%E5%BD%95%E7%BB%84%E6%B5%8B%E5%BA%8F\%E5%AE%8C%E6%95%B4%E7%89%88%E6%95%B0%E6%8D%AE\GDR3855-Hylocereus_undulatus_Britt-12-RNAseq_result\4_Function\2_Group_Diff_Function\UP_DOWN\GO\NL-VS-L1.F.html#gene234) | alpha-(1->3)-fucosyltransferase activity | 1 (0.08%) | 2 (0.06%) | 0.584658 | 0.980162 |
| 235 | [GO:0051002](file:///E:\2018-7-3%E7%81%AB%E9%BE%99%E6%9E%9C%E8%BD%AC%E5%BD%95%E7%BB%84%E6%B5%8B%E5%BA%8F\%E5%AE%8C%E6%95%B4%E7%89%88%E6%95%B0%E6%8D%AE\GDR3855-Hylocereus_undulatus_Britt-12-RNAseq_result\4_Function\2_Group_Diff_Function\UP_DOWN\GO\NL-VS-L1.F.html#gene235) | ligase activity, forming nitrogen-metal bonds | 1 (0.08%) | 2 (0.06%) | 0.584658 | 0.980162 |
| 236 | [GO:0051003](file:///E:\2018-7-3%E7%81%AB%E9%BE%99%E6%9E%9C%E8%BD%AC%E5%BD%95%E7%BB%84%E6%B5%8B%E5%BA%8F\%E5%AE%8C%E6%95%B4%E7%89%88%E6%95%B0%E6%8D%AE\GDR3855-Hylocereus_undulatus_Britt-12-RNAseq_result\4_Function\2_Group_Diff_Function\UP_DOWN\GO\NL-VS-L1.F.html#gene236) | ligase activity, forming nitrogen-metal bonds, forming coordination complexes | 1 (0.08%) | 2 (0.06%) | 0.584658 | 0.980162 |
| 237 | [GO:0052834](file:///E:\2018-7-3%E7%81%AB%E9%BE%99%E6%9E%9C%E8%BD%AC%E5%BD%95%E7%BB%84%E6%B5%8B%E5%BA%8F\%E5%AE%8C%E6%95%B4%E7%89%88%E6%95%B0%E6%8D%AE\GDR3855-Hylocereus_undulatus_Britt-12-RNAseq_result\4_Function\2_Group_Diff_Function\UP_DOWN\GO\NL-VS-L1.F.html#gene237) | inositol monophosphate phosphatase activity | 1 (0.08%) | 2 (0.06%) | 0.584658 | 0.980162 |
| 238 | [GO:0070546](file:///E:\2018-7-3%E7%81%AB%E9%BE%99%E6%9E%9C%E8%BD%AC%E5%BD%95%E7%BB%84%E6%B5%8B%E5%BA%8F\%E5%AE%8C%E6%95%B4%E7%89%88%E6%95%B0%E6%8D%AE\GDR3855-Hylocereus_undulatus_Britt-12-RNAseq_result\4_Function\2_Group_Diff_Function\UP_DOWN\GO\NL-VS-L1.F.html#gene238) | L-phenylalanine aminotransferase activity | 1 (0.08%) | 2 (0.06%) | 0.584658 | 0.980162 |
| 239 | [GO:0016832](file:///E:\2018-7-3%E7%81%AB%E9%BE%99%E6%9E%9C%E8%BD%AC%E5%BD%95%E7%BB%84%E6%B5%8B%E5%BA%8F\%E5%AE%8C%E6%95%B4%E7%89%88%E6%95%B0%E6%8D%AE\GDR3855-Hylocereus_undulatus_Britt-12-RNAseq_result\4_Function\2_Group_Diff_Function\UP_DOWN\GO\NL-VS-L1.F.html#gene239) | aldehyde-lyase activity | 4 (0.33%) | 11 (0.32%) | 0.589775 | 0.984604 |
| 240 | [GO:0015604](file:///E:\2018-7-3%E7%81%AB%E9%BE%99%E6%9E%9C%E8%BD%AC%E5%BD%95%E7%BB%84%E6%B5%8B%E5%BA%8F\%E5%AE%8C%E6%95%B4%E7%89%88%E6%95%B0%E6%8D%AE\GDR3855-Hylocereus_undulatus_Britt-12-RNAseq_result\4_Function\2_Group_Diff_Function\UP_DOWN\GO\NL-VS-L1.F.html#gene240) | organic phosphonate transmembrane transporter activity | 5 (0.41%) | 14 (0.41%) | 0.594481 | 0.988325 |
| 241 | [GO:0048037](file:///E:\2018-7-3%E7%81%AB%E9%BE%99%E6%9E%9C%E8%BD%AC%E5%BD%95%E7%BB%84%E6%B5%8B%E5%BA%8F\%E5%AE%8C%E6%95%B4%E7%89%88%E6%95%B0%E6%8D%AE\GDR3855-Hylocereus_undulatus_Britt-12-RNAseq_result\4_Function\2_Group_Diff_Function\UP_DOWN\GO\NL-VS-L1.F.html#gene241) | cofactor binding | 9 (0.74%) | 26 (0.76%) | 0.612648 | 0.999471 |
| 242 | [GO:0016853](file:///E:\2018-7-3%E7%81%AB%E9%BE%99%E6%9E%9C%E8%BD%AC%E5%BD%95%E7%BB%84%E6%B5%8B%E5%BA%8F\%E5%AE%8C%E6%95%B4%E7%89%88%E6%95%B0%E6%8D%AE\GDR3855-Hylocereus_undulatus_Britt-12-RNAseq_result\4_Function\2_Group_Diff_Function\UP_DOWN\GO\NL-VS-L1.F.html#gene242) | isomerase activity | 21 (1.72%) | 61 (1.78%) | 0.620884 | 0.999471 |
| 243 | [GO:0004497](file:///E:\2018-7-3%E7%81%AB%E9%BE%99%E6%9E%9C%E8%BD%AC%E5%BD%95%E7%BB%84%E6%B5%8B%E5%BA%8F\%E5%AE%8C%E6%95%B4%E7%89%88%E6%95%B0%E6%8D%AE\GDR3855-Hylocereus_undulatus_Britt-12-RNAseq_result\4_Function\2_Group_Diff_Function\UP_DOWN\GO\NL-VS-L1.F.html#gene243) | monooxygenase activity | 12 (0.98%) | 35 (1.02%) | 0.624882 | 0.999471 |
| 244 | [GO:0015077](file:///E:\2018-7-3%E7%81%AB%E9%BE%99%E6%9E%9C%E8%BD%AC%E5%BD%95%E7%BB%84%E6%B5%8B%E5%BA%8F\%E5%AE%8C%E6%95%B4%E7%89%88%E6%95%B0%E6%8D%AE\GDR3855-Hylocereus_undulatus_Britt-12-RNAseq_result\4_Function\2_Group_Diff_Function\UP_DOWN\GO\NL-VS-L1.F.html#gene244) | monovalent inorganic cation transmembrane transporter activity | 14 (1.15%) | 41 (1.19%) | 0.632433 | 0.999471 |
| 245 | [GO:0016614](file:///E:\2018-7-3%E7%81%AB%E9%BE%99%E6%9E%9C%E8%BD%AC%E5%BD%95%E7%BB%84%E6%B5%8B%E5%BA%8F\%E5%AE%8C%E6%95%B4%E7%89%88%E6%95%B0%E6%8D%AE\GDR3855-Hylocereus_undulatus_Britt-12-RNAseq_result\4_Function\2_Group_Diff_Function\UP_DOWN\GO\NL-VS-L1.F.html#gene245) | oxidoreductase activity, acting on CH-OH group of donors | 27 (2.21%) | 79 (2.3%) | 0.643027 | 0.999471 |
| 246 | [GO:0016741](file:///E:\2018-7-3%E7%81%AB%E9%BE%99%E6%9E%9C%E8%BD%AC%E5%BD%95%E7%BB%84%E6%B5%8B%E5%BA%8F\%E5%AE%8C%E6%95%B4%E7%89%88%E6%95%B0%E6%8D%AE\GDR3855-Hylocereus_undulatus_Britt-12-RNAseq_result\4_Function\2_Group_Diff_Function\UP_DOWN\GO\NL-VS-L1.F.html#gene246) | transferase activity, transferring one-carbon groups | 28 (2.3%) | 82 (2.39%) | 0.646379 | 0.999471 |
| 247 | [GO:0008378](file:///E:\2018-7-3%E7%81%AB%E9%BE%99%E6%9E%9C%E8%BD%AC%E5%BD%95%E7%BB%84%E6%B5%8B%E5%BA%8F\%E5%AE%8C%E6%95%B4%E7%89%88%E6%95%B0%E6%8D%AE\GDR3855-Hylocereus_undulatus_Britt-12-RNAseq_result\4_Function\2_Group_Diff_Function\UP_DOWN\GO\NL-VS-L1.F.html#gene247) | galactosyltransferase activity | 5 (0.41%) | 15 (0.44%) | 0.664913 | 0.999471 |
| 248 | [GO:0051213](file:///E:\2018-7-3%E7%81%AB%E9%BE%99%E6%9E%9C%E8%BD%AC%E5%BD%95%E7%BB%84%E6%B5%8B%E5%BA%8F\%E5%AE%8C%E6%95%B4%E7%89%88%E6%95%B0%E6%8D%AE\GDR3855-Hylocereus_undulatus_Britt-12-RNAseq_result\4_Function\2_Group_Diff_Function\UP_DOWN\GO\NL-VS-L1.F.html#gene248) | dioxygenase activity | 5 (0.41%) | 15 (0.44%) | 0.664913 | 0.999471 |
| 249 | [GO:0015036](file:///E:\2018-7-3%E7%81%AB%E9%BE%99%E6%9E%9C%E8%BD%AC%E5%BD%95%E7%BB%84%E6%B5%8B%E5%BA%8F\%E5%AE%8C%E6%95%B4%E7%89%88%E6%95%B0%E6%8D%AE\GDR3855-Hylocereus_undulatus_Britt-12-RNAseq_result\4_Function\2_Group_Diff_Function\UP_DOWN\GO\NL-VS-L1.F.html#gene249) | disulfide oxidoreductase activity | 9 (0.74%) | 27 (0.79%) | 0.664998 | 0.999471 |
| 250 | [GO:0016903](file:///E:\2018-7-3%E7%81%AB%E9%BE%99%E6%9E%9C%E8%BD%AC%E5%BD%95%E7%BB%84%E6%B5%8B%E5%BA%8F\%E5%AE%8C%E6%95%B4%E7%89%88%E6%95%B0%E6%8D%AE\GDR3855-Hylocereus_undulatus_Britt-12-RNAseq_result\4_Function\2_Group_Diff_Function\UP_DOWN\GO\NL-VS-L1.F.html#gene250) | oxidoreductase activity, acting on the aldehyde or oxo group of donors | 11 (0.9%) | 33 (0.96%) | 0.668107 | 0.999471 |
| 251 | [GO:0016706](file:///E:\2018-7-3%E7%81%AB%E9%BE%99%E6%9E%9C%E8%BD%AC%E5%BD%95%E7%BB%84%E6%B5%8B%E5%BA%8F\%E5%AE%8C%E6%95%B4%E7%89%88%E6%95%B0%E6%8D%AE\GDR3855-Hylocereus_undulatus_Britt-12-RNAseq_result\4_Function\2_Group_Diff_Function\UP_DOWN\GO\NL-VS-L1.F.html#gene251) | oxidoreductase activity, acting on paired donors, with incorporation or reduction of molecular oxygen, 2-oxoglutarate as one donor, and incorporation of one atom each of oxygen into both donors | 4 (0.33%) | 12 (0.35%) | 0.668363 | 0.999471 |
| 252 | [GO:0035250](file:///E:\2018-7-3%E7%81%AB%E9%BE%99%E6%9E%9C%E8%BD%AC%E5%BD%95%E7%BB%84%E6%B5%8B%E5%BA%8F\%E5%AE%8C%E6%95%B4%E7%89%88%E6%95%B0%E6%8D%AE\GDR3855-Hylocereus_undulatus_Britt-12-RNAseq_result\4_Function\2_Group_Diff_Function\UP_DOWN\GO\NL-VS-L1.F.html#gene252) | UDP-galactosyltransferase activity | 4 (0.33%) | 12 (0.35%) | 0.668363 | 0.999471 |
| 253 | [GO:1901363](file:///E:\2018-7-3%E7%81%AB%E9%BE%99%E6%9E%9C%E8%BD%AC%E5%BD%95%E7%BB%84%E6%B5%8B%E5%BA%8F\%E5%AE%8C%E6%95%B4%E7%89%88%E6%95%B0%E6%8D%AE\GDR3855-Hylocereus_undulatus_Britt-12-RNAseq_result\4_Function\2_Group_Diff_Function\UP_DOWN\GO\NL-VS-L1.F.html#gene253) | heterocyclic compound binding | 326 (26.72%) | 931 (27.13%) | 0.668462 | 0.999471 |
| 254 | [GO:0004386](file:///E:\2018-7-3%E7%81%AB%E9%BE%99%E6%9E%9C%E8%BD%AC%E5%BD%95%E7%BB%84%E6%B5%8B%E5%BA%8F\%E5%AE%8C%E6%95%B4%E7%89%88%E6%95%B0%E6%8D%AE\GDR3855-Hylocereus_undulatus_Britt-12-RNAseq_result\4_Function\2_Group_Diff_Function\UP_DOWN\GO\NL-VS-L1.F.html#gene254) | helicase activity | 3 (0.25%) | 9 (0.26%) | 0.675637 | 0.999471 |
| 255 | [GO:0016405](file:///E:\2018-7-3%E7%81%AB%E9%BE%99%E6%9E%9C%E8%BD%AC%E5%BD%95%E7%BB%84%E6%B5%8B%E5%BA%8F\%E5%AE%8C%E6%95%B4%E7%89%88%E6%95%B0%E6%8D%AE\GDR3855-Hylocereus_undulatus_Britt-12-RNAseq_result\4_Function\2_Group_Diff_Function\UP_DOWN\GO\NL-VS-L1.F.html#gene255) | CoA-ligase activity | 3 (0.25%) | 9 (0.26%) | 0.675637 | 0.999471 |
| 256 | [GO:0016763](file:///E:\2018-7-3%E7%81%AB%E9%BE%99%E6%9E%9C%E8%BD%AC%E5%BD%95%E7%BB%84%E6%B5%8B%E5%BA%8F\%E5%AE%8C%E6%95%B4%E7%89%88%E6%95%B0%E6%8D%AE\GDR3855-Hylocereus_undulatus_Britt-12-RNAseq_result\4_Function\2_Group_Diff_Function\UP_DOWN\GO\NL-VS-L1.F.html#gene256) | transferase activity, transferring pentosyl groups | 3 (0.25%) | 9 (0.26%) | 0.675637 | 0.999471 |
| 257 | [GO:0015605](file:///E:\2018-7-3%E7%81%AB%E9%BE%99%E6%9E%9C%E8%BD%AC%E5%BD%95%E7%BB%84%E6%B5%8B%E5%BA%8F\%E5%AE%8C%E6%95%B4%E7%89%88%E6%95%B0%E6%8D%AE\GDR3855-Hylocereus_undulatus_Britt-12-RNAseq_result\4_Function\2_Group_Diff_Function\UP_DOWN\GO\NL-VS-L1.F.html#gene257) | organophosphate ester transmembrane transporter activity | 2 (0.16%) | 6 (0.17%) | 0.691303 | 0.999471 |
| 258 | [GO:0016646](file:///E:\2018-7-3%E7%81%AB%E9%BE%99%E6%9E%9C%E8%BD%AC%E5%BD%95%E7%BB%84%E6%B5%8B%E5%BA%8F\%E5%AE%8C%E6%95%B4%E7%89%88%E6%95%B0%E6%8D%AE\GDR3855-Hylocereus_undulatus_Britt-12-RNAseq_result\4_Function\2_Group_Diff_Function\UP_DOWN\GO\NL-VS-L1.F.html#gene258) | oxidoreductase activity, acting on the CH-NH group of donors, NAD or NADP as acceptor | 2 (0.16%) | 6 (0.17%) | 0.691303 | 0.999471 |
| 259 | [GO:0016712](file:///E:\2018-7-3%E7%81%AB%E9%BE%99%E6%9E%9C%E8%BD%AC%E5%BD%95%E7%BB%84%E6%B5%8B%E5%BA%8F\%E5%AE%8C%E6%95%B4%E7%89%88%E6%95%B0%E6%8D%AE\GDR3855-Hylocereus_undulatus_Britt-12-RNAseq_result\4_Function\2_Group_Diff_Function\UP_DOWN\GO\NL-VS-L1.F.html#gene259) | oxidoreductase activity, acting on paired donors, with incorporation or reduction of molecular oxygen, reduced flavin or flavoprotein as one donor, and incorporation of one atom of oxygen | 2 (0.16%) | 6 (0.17%) | 0.691303 | 0.999471 |
| 260 | [GO:0016838](file:///E:\2018-7-3%E7%81%AB%E9%BE%99%E6%9E%9C%E8%BD%AC%E5%BD%95%E7%BB%84%E6%B5%8B%E5%BA%8F\%E5%AE%8C%E6%95%B4%E7%89%88%E6%95%B0%E6%8D%AE\GDR3855-Hylocereus_undulatus_Britt-12-RNAseq_result\4_Function\2_Group_Diff_Function\UP_DOWN\GO\NL-VS-L1.F.html#gene260) | carbon-oxygen lyase activity, acting on phosphates | 2 (0.16%) | 6 (0.17%) | 0.691303 | 0.999471 |
| 261 | [GO:0019205](file:///E:\2018-7-3%E7%81%AB%E9%BE%99%E6%9E%9C%E8%BD%AC%E5%BD%95%E7%BB%84%E6%B5%8B%E5%BA%8F\%E5%AE%8C%E6%95%B4%E7%89%88%E6%95%B0%E6%8D%AE\GDR3855-Hylocereus_undulatus_Britt-12-RNAseq_result\4_Function\2_Group_Diff_Function\UP_DOWN\GO\NL-VS-L1.F.html#gene261) | nucleobase-containing compound kinase activity | 2 (0.16%) | 6 (0.17%) | 0.691303 | 0.999471 |
| 262 | [GO:0019238](file:///E:\2018-7-3%E7%81%AB%E9%BE%99%E6%9E%9C%E8%BD%AC%E5%BD%95%E7%BB%84%E6%B5%8B%E5%BA%8F\%E5%AE%8C%E6%95%B4%E7%89%88%E6%95%B0%E6%8D%AE\GDR3855-Hylocereus_undulatus_Britt-12-RNAseq_result\4_Function\2_Group_Diff_Function\UP_DOWN\GO\NL-VS-L1.F.html#gene262) | cyclohydrolase activity | 2 (0.16%) | 6 (0.17%) | 0.691303 | 0.999471 |
| 263 | [GO:1901677](file:///E:\2018-7-3%E7%81%AB%E9%BE%99%E6%9E%9C%E8%BD%AC%E5%BD%95%E7%BB%84%E6%B5%8B%E5%BA%8F\%E5%AE%8C%E6%95%B4%E7%89%88%E6%95%B0%E6%8D%AE\GDR3855-Hylocereus_undulatus_Britt-12-RNAseq_result\4_Function\2_Group_Diff_Function\UP_DOWN\GO\NL-VS-L1.F.html#gene263) | phosphate transmembrane transporter activity | 2 (0.16%) | 6 (0.17%) | 0.691303 | 0.999471 |
| 264 | [GO:0070011](file:///E:\2018-7-3%E7%81%AB%E9%BE%99%E6%9E%9C%E8%BD%AC%E5%BD%95%E7%BB%84%E6%B5%8B%E5%BA%8F\%E5%AE%8C%E6%95%B4%E7%89%88%E6%95%B0%E6%8D%AE\GDR3855-Hylocereus_undulatus_Britt-12-RNAseq_result\4_Function\2_Group_Diff_Function\UP_DOWN\GO\NL-VS-L1.F.html#gene264) | peptidase activity, acting on L-amino acid peptides | 25 (2.05%) | 75 (2.19%) | 0.697919 | 0.999471 |
| 265 | [GO:0003677](file:///E:\2018-7-3%E7%81%AB%E9%BE%99%E6%9E%9C%E8%BD%AC%E5%BD%95%E7%BB%84%E6%B5%8B%E5%BA%8F\%E5%AE%8C%E6%95%B4%E7%89%88%E6%95%B0%E6%8D%AE\GDR3855-Hylocereus_undulatus_Britt-12-RNAseq_result\4_Function\2_Group_Diff_Function\UP_DOWN\GO\NL-VS-L1.F.html#gene265) | DNA binding | 14 (1.15%) | 43 (1.25%) | 0.712720 | 0.999471 |
| 266 | [GO:0019787](file:///E:\2018-7-3%E7%81%AB%E9%BE%99%E6%9E%9C%E8%BD%AC%E5%BD%95%E7%BB%84%E6%B5%8B%E5%BA%8F\%E5%AE%8C%E6%95%B4%E7%89%88%E6%95%B0%E6%8D%AE\GDR3855-Hylocereus_undulatus_Britt-12-RNAseq_result\4_Function\2_Group_Diff_Function\UP_DOWN\GO\NL-VS-L1.F.html#gene266) | ubiquitin-like protein transferase activity | 5 (0.41%) | 16 (0.47%) | 0.726806 | 0.999471 |
| 267 | [GO:0000295](file:///E:\2018-7-3%E7%81%AB%E9%BE%99%E6%9E%9C%E8%BD%AC%E5%BD%95%E7%BB%84%E6%B5%8B%E5%BA%8F\%E5%AE%8C%E6%95%B4%E7%89%88%E6%95%B0%E6%8D%AE\GDR3855-Hylocereus_undulatus_Britt-12-RNAseq_result\4_Function\2_Group_Diff_Function\UP_DOWN\GO\NL-VS-L1.F.html#gene267) | adenine nucleotide transmembrane transporter activity | 1 (0.08%) | 3 (0.09%) | 0.732389 | 0.999471 |
| 268 | [GO:0003690](file:///E:\2018-7-3%E7%81%AB%E9%BE%99%E6%9E%9C%E8%BD%AC%E5%BD%95%E7%BB%84%E6%B5%8B%E5%BA%8F\%E5%AE%8C%E6%95%B4%E7%89%88%E6%95%B0%E6%8D%AE\GDR3855-Hylocereus_undulatus_Britt-12-RNAseq_result\4_Function\2_Group_Diff_Function\UP_DOWN\GO\NL-VS-L1.F.html#gene268) | double-stranded DNA binding | 1 (0.08%) | 3 (0.09%) | 0.732389 | 0.999471 |
| 269 | [GO:0004396](file:///E:\2018-7-3%E7%81%AB%E9%BE%99%E6%9E%9C%E8%BD%AC%E5%BD%95%E7%BB%84%E6%B5%8B%E5%BA%8F\%E5%AE%8C%E6%95%B4%E7%89%88%E6%95%B0%E6%8D%AE\GDR3855-Hylocereus_undulatus_Britt-12-RNAseq_result\4_Function\2_Group_Diff_Function\UP_DOWN\GO\NL-VS-L1.F.html#gene269) | hexokinase activity | 1 (0.08%) | 3 (0.09%) | 0.732389 | 0.999471 |
| 270 | [GO:0004774](file:///E:\2018-7-3%E7%81%AB%E9%BE%99%E6%9E%9C%E8%BD%AC%E5%BD%95%E7%BB%84%E6%B5%8B%E5%BA%8F\%E5%AE%8C%E6%95%B4%E7%89%88%E6%95%B0%E6%8D%AE\GDR3855-Hylocereus_undulatus_Britt-12-RNAseq_result\4_Function\2_Group_Diff_Function\UP_DOWN\GO\NL-VS-L1.F.html#gene270) | succinate-CoA ligase activity | 1 (0.08%) | 3 (0.09%) | 0.732389 | 0.999471 |
| 271 | [GO:0008236](file:///E:\2018-7-3%E7%81%AB%E9%BE%99%E6%9E%9C%E8%BD%AC%E5%BD%95%E7%BB%84%E6%B5%8B%E5%BA%8F\%E5%AE%8C%E6%95%B4%E7%89%88%E6%95%B0%E6%8D%AE\GDR3855-Hylocereus_undulatus_Britt-12-RNAseq_result\4_Function\2_Group_Diff_Function\UP_DOWN\GO\NL-VS-L1.F.html#gene271) | serine-type peptidase activity | 1 (0.08%) | 3 (0.09%) | 0.732389 | 0.999471 |
| 272 | [GO:0009055](file:///E:\2018-7-3%E7%81%AB%E9%BE%99%E6%9E%9C%E8%BD%AC%E5%BD%95%E7%BB%84%E6%B5%8B%E5%BA%8F\%E5%AE%8C%E6%95%B4%E7%89%88%E6%95%B0%E6%8D%AE\GDR3855-Hylocereus_undulatus_Britt-12-RNAseq_result\4_Function\2_Group_Diff_Function\UP_DOWN\GO\NL-VS-L1.F.html#gene272) | electron carrier activity | 1 (0.08%) | 3 (0.09%) | 0.732389 | 0.999471 |
| 273 | [GO:0015154](file:///E:\2018-7-3%E7%81%AB%E9%BE%99%E6%9E%9C%E8%BD%AC%E5%BD%95%E7%BB%84%E6%B5%8B%E5%BA%8F\%E5%AE%8C%E6%95%B4%E7%89%88%E6%95%B0%E6%8D%AE\GDR3855-Hylocereus_undulatus_Britt-12-RNAseq_result\4_Function\2_Group_Diff_Function\UP_DOWN\GO\NL-VS-L1.F.html#gene273) | disaccharide transmembrane transporter activity | 1 (0.08%) | 3 (0.09%) | 0.732389 | 0.999471 |
| 274 | [GO:0015157](file:///E:\2018-7-3%E7%81%AB%E9%BE%99%E6%9E%9C%E8%BD%AC%E5%BD%95%E7%BB%84%E6%B5%8B%E5%BA%8F\%E5%AE%8C%E6%95%B4%E7%89%88%E6%95%B0%E6%8D%AE\GDR3855-Hylocereus_undulatus_Britt-12-RNAseq_result\4_Function\2_Group_Diff_Function\UP_DOWN\GO\NL-VS-L1.F.html#gene274) | oligosaccharide transmembrane transporter activity | 1 (0.08%) | 3 (0.09%) | 0.732389 | 0.999471 |
| 275 | [GO:0015216](file:///E:\2018-7-3%E7%81%AB%E9%BE%99%E6%9E%9C%E8%BD%AC%E5%BD%95%E7%BB%84%E6%B5%8B%E5%BA%8F\%E5%AE%8C%E6%95%B4%E7%89%88%E6%95%B0%E6%8D%AE\GDR3855-Hylocereus_undulatus_Britt-12-RNAseq_result\4_Function\2_Group_Diff_Function\UP_DOWN\GO\NL-VS-L1.F.html#gene275) | purine nucleotide transmembrane transporter activity | 1 (0.08%) | 3 (0.09%) | 0.732389 | 0.999471 |
| 276 | [GO:0016160](file:///E:\2018-7-3%E7%81%AB%E9%BE%99%E6%9E%9C%E8%BD%AC%E5%BD%95%E7%BB%84%E6%B5%8B%E5%BA%8F\%E5%AE%8C%E6%95%B4%E7%89%88%E6%95%B0%E6%8D%AE\GDR3855-Hylocereus_undulatus_Britt-12-RNAseq_result\4_Function\2_Group_Diff_Function\UP_DOWN\GO\NL-VS-L1.F.html#gene276) | amylase activity | 1 (0.08%) | 3 (0.09%) | 0.732389 | 0.999471 |
| 277 | [GO:0016454](file:///E:\2018-7-3%E7%81%AB%E9%BE%99%E6%9E%9C%E8%BD%AC%E5%BD%95%E7%BB%84%E6%B5%8B%E5%BA%8F\%E5%AE%8C%E6%95%B4%E7%89%88%E6%95%B0%E6%8D%AE\GDR3855-Hylocereus_undulatus_Britt-12-RNAseq_result\4_Function\2_Group_Diff_Function\UP_DOWN\GO\NL-VS-L1.F.html#gene277) | C-palmitoyltransferase activity | 1 (0.08%) | 3 (0.09%) | 0.732389 | 0.999471 |
| 278 | [GO:0016702](file:///E:\2018-7-3%E7%81%AB%E9%BE%99%E6%9E%9C%E8%BD%AC%E5%BD%95%E7%BB%84%E6%B5%8B%E5%BA%8F\%E5%AE%8C%E6%95%B4%E7%89%88%E6%95%B0%E6%8D%AE\GDR3855-Hylocereus_undulatus_Britt-12-RNAseq_result\4_Function\2_Group_Diff_Function\UP_DOWN\GO\NL-VS-L1.F.html#gene278) | oxidoreductase activity, acting on single donors with incorporation of molecular oxygen, incorporation of two atoms of oxygen | 1 (0.08%) | 3 (0.09%) | 0.732389 | 0.999471 |
| 279 | [GO:0016878](file:///E:\2018-7-3%E7%81%AB%E9%BE%99%E6%9E%9C%E8%BD%AC%E5%BD%95%E7%BB%84%E6%B5%8B%E5%BA%8F\%E5%AE%8C%E6%95%B4%E7%89%88%E6%95%B0%E6%8D%AE\GDR3855-Hylocereus_undulatus_Britt-12-RNAseq_result\4_Function\2_Group_Diff_Function\UP_DOWN\GO\NL-VS-L1.F.html#gene279) | acid-thiol ligase activity | 1 (0.08%) | 3 (0.09%) | 0.732389 | 0.999471 |
| 280 | [GO:0016893](file:///E:\2018-7-3%E7%81%AB%E9%BE%99%E6%9E%9C%E8%BD%AC%E5%BD%95%E7%BB%84%E6%B5%8B%E5%BA%8F\%E5%AE%8C%E6%95%B4%E7%89%88%E6%95%B0%E6%8D%AE\GDR3855-Hylocereus_undulatus_Britt-12-RNAseq_result\4_Function\2_Group_Diff_Function\UP_DOWN\GO\NL-VS-L1.F.html#gene280) | endonuclease activity, active with either ribo- or deoxyribonucleic acids and producing 5'-phosphomonoesters | 1 (0.08%) | 3 (0.09%) | 0.732389 | 0.999471 |
| 281 | [GO:0017171](file:///E:\2018-7-3%E7%81%AB%E9%BE%99%E6%9E%9C%E8%BD%AC%E5%BD%95%E7%BB%84%E6%B5%8B%E5%BA%8F\%E5%AE%8C%E6%95%B4%E7%89%88%E6%95%B0%E6%8D%AE\GDR3855-Hylocereus_undulatus_Britt-12-RNAseq_result\4_Function\2_Group_Diff_Function\UP_DOWN\GO\NL-VS-L1.F.html#gene281) | serine hydrolase activity | 1 (0.08%) | 3 (0.09%) | 0.732389 | 0.999471 |
| 282 | [GO:0019208](file:///E:\2018-7-3%E7%81%AB%E9%BE%99%E6%9E%9C%E8%BD%AC%E5%BD%95%E7%BB%84%E6%B5%8B%E5%BA%8F\%E5%AE%8C%E6%95%B4%E7%89%88%E6%95%B0%E6%8D%AE\GDR3855-Hylocereus_undulatus_Britt-12-RNAseq_result\4_Function\2_Group_Diff_Function\UP_DOWN\GO\NL-VS-L1.F.html#gene282) | phosphatase regulator activity | 1 (0.08%) | 3 (0.09%) | 0.732389 | 0.999471 |
| 283 | [GO:0019888](file:///E:\2018-7-3%E7%81%AB%E9%BE%99%E6%9E%9C%E8%BD%AC%E5%BD%95%E7%BB%84%E6%B5%8B%E5%BA%8F\%E5%AE%8C%E6%95%B4%E7%89%88%E6%95%B0%E6%8D%AE\GDR3855-Hylocereus_undulatus_Britt-12-RNAseq_result\4_Function\2_Group_Diff_Function\UP_DOWN\GO\NL-VS-L1.F.html#gene283) | protein phosphatase regulator activity | 1 (0.08%) | 3 (0.09%) | 0.732389 | 0.999471 |
| 284 | [GO:1901505](file:///E:\2018-7-3%E7%81%AB%E9%BE%99%E6%9E%9C%E8%BD%AC%E5%BD%95%E7%BB%84%E6%B5%8B%E5%BA%8F\%E5%AE%8C%E6%95%B4%E7%89%88%E6%95%B0%E6%8D%AE\GDR3855-Hylocereus_undulatus_Britt-12-RNAseq_result\4_Function\2_Group_Diff_Function\UP_DOWN\GO\NL-VS-L1.F.html#gene284) | carbohydrate derivative transporter activity | 1 (0.08%) | 3 (0.09%) | 0.732389 | 0.999471 |
| 285 | [GO:0016877](file:///E:\2018-7-3%E7%81%AB%E9%BE%99%E6%9E%9C%E8%BD%AC%E5%BD%95%E7%BB%84%E6%B5%8B%E5%BA%8F\%E5%AE%8C%E6%95%B4%E7%89%88%E6%95%B0%E6%8D%AE\GDR3855-Hylocereus_undulatus_Britt-12-RNAseq_result\4_Function\2_Group_Diff_Function\UP_DOWN\GO\NL-VS-L1.F.html#gene285) | ligase activity, forming carbon-sulfur bonds | 4 (0.33%) | 13 (0.38%) | 0.735891 | 0.999471 |
| 286 | [GO:0004702](file:///E:\2018-7-3%E7%81%AB%E9%BE%99%E6%9E%9C%E8%BD%AC%E5%BD%95%E7%BB%84%E6%B5%8B%E5%BA%8F\%E5%AE%8C%E6%95%B4%E7%89%88%E6%95%B0%E6%8D%AE\GDR3855-Hylocereus_undulatus_Britt-12-RNAseq_result\4_Function\2_Group_Diff_Function\UP_DOWN\GO\NL-VS-L1.F.html#gene286) | receptor signaling protein serine/threonine kinase activity | 14 (1.15%) | 44 (1.28%) | 0.748499 | 0.999471 |
| 287 | [GO:0005057](file:///E:\2018-7-3%E7%81%AB%E9%BE%99%E6%9E%9C%E8%BD%AC%E5%BD%95%E7%BB%84%E6%B5%8B%E5%BA%8F\%E5%AE%8C%E6%95%B4%E7%89%88%E6%95%B0%E6%8D%AE\GDR3855-Hylocereus_undulatus_Britt-12-RNAseq_result\4_Function\2_Group_Diff_Function\UP_DOWN\GO\NL-VS-L1.F.html#gene287) | receptor signaling protein activity | 14 (1.15%) | 44 (1.28%) | 0.748499 | 0.999471 |
| 288 | [GO:0016409](file:///E:\2018-7-3%E7%81%AB%E9%BE%99%E6%9E%9C%E8%BD%AC%E5%BD%95%E7%BB%84%E6%B5%8B%E5%BA%8F\%E5%AE%8C%E6%95%B4%E7%89%88%E6%95%B0%E6%8D%AE\GDR3855-Hylocereus_undulatus_Britt-12-RNAseq_result\4_Function\2_Group_Diff_Function\UP_DOWN\GO\NL-VS-L1.F.html#gene288) | palmitoyltransferase activity | 3 (0.25%) | 10 (0.29%) | 0.750439 | 0.999471 |
| 289 | [GO:0044877](file:///E:\2018-7-3%E7%81%AB%E9%BE%99%E6%9E%9C%E8%BD%AC%E5%BD%95%E7%BB%84%E6%B5%8B%E5%BA%8F\%E5%AE%8C%E6%95%B4%E7%89%88%E6%95%B0%E6%8D%AE\GDR3855-Hylocereus_undulatus_Britt-12-RNAseq_result\4_Function\2_Group_Diff_Function\UP_DOWN\GO\NL-VS-L1.F.html#gene289) | macromolecular complex binding | 3 (0.25%) | 10 (0.29%) | 0.750439 | 0.999471 |
| 290 | [GO:0016879](file:///E:\2018-7-3%E7%81%AB%E9%BE%99%E6%9E%9C%E8%BD%AC%E5%BD%95%E7%BB%84%E6%B5%8B%E5%BA%8F\%E5%AE%8C%E6%95%B4%E7%89%88%E6%95%B0%E6%8D%AE\GDR3855-Hylocereus_undulatus_Britt-12-RNAseq_result\4_Function\2_Group_Diff_Function\UP_DOWN\GO\NL-VS-L1.F.html#gene290) | ligase activity, forming carbon-nitrogen bonds | 11 (0.9%) | 35 (1.02%) | 0.751516 | 0.999471 |
| 291 | [GO:0016667](file:///E:\2018-7-3%E7%81%AB%E9%BE%99%E6%9E%9C%E8%BD%AC%E5%BD%95%E7%BB%84%E6%B5%8B%E5%BA%8F\%E5%AE%8C%E6%95%B4%E7%89%88%E6%95%B0%E6%8D%AE\GDR3855-Hylocereus_undulatus_Britt-12-RNAseq_result\4_Function\2_Group_Diff_Function\UP_DOWN\GO\NL-VS-L1.F.html#gene291) | oxidoreductase activity, acting on a sulfur group of donors | 10 (0.82%) | 32 (0.93%) | 0.753488 | 0.999471 |
| 292 | [GO:0016787](file:///E:\2018-7-3%E7%81%AB%E9%BE%99%E6%9E%9C%E8%BD%AC%E5%BD%95%E7%BB%84%E6%B5%8B%E5%BA%8F\%E5%AE%8C%E6%95%B4%E7%89%88%E6%95%B0%E6%8D%AE\GDR3855-Hylocereus_undulatus_Britt-12-RNAseq_result\4_Function\2_Group_Diff_Function\UP_DOWN\GO\NL-VS-L1.F.html#gene292) | hydrolase activity | 251 (20.57%) | 727 (21.18%) | 0.755224 | 0.999471 |
| 293 | [GO:0016835](file:///E:\2018-7-3%E7%81%AB%E9%BE%99%E6%9E%9C%E8%BD%AC%E5%BD%95%E7%BB%84%E6%B5%8B%E5%BA%8F\%E5%AE%8C%E6%95%B4%E7%89%88%E6%95%B0%E6%8D%AE\GDR3855-Hylocereus_undulatus_Britt-12-RNAseq_result\4_Function\2_Group_Diff_Function\UP_DOWN\GO\NL-VS-L1.F.html#gene293) | carbon-oxygen lyase activity | 9 (0.74%) | 29 (0.84%) | 0.756167 | 0.999471 |
| 294 | [GO:0004175](file:///E:\2018-7-3%E7%81%AB%E9%BE%99%E6%9E%9C%E8%BD%AC%E5%BD%95%E7%BB%84%E6%B5%8B%E5%BA%8F\%E5%AE%8C%E6%95%B4%E7%89%88%E6%95%B0%E6%8D%AE\GDR3855-Hylocereus_undulatus_Britt-12-RNAseq_result\4_Function\2_Group_Diff_Function\UP_DOWN\GO\NL-VS-L1.F.html#gene294) | endopeptidase activity | 6 (0.49%) | 20 (0.58%) | 0.771085 | 0.999471 |
| 295 | [GO:0008171](file:///E:\2018-7-3%E7%81%AB%E9%BE%99%E6%9E%9C%E8%BD%AC%E5%BD%95%E7%BB%84%E6%B5%8B%E5%BA%8F\%E5%AE%8C%E6%95%B4%E7%89%88%E6%95%B0%E6%8D%AE\GDR3855-Hylocereus_undulatus_Britt-12-RNAseq_result\4_Function\2_Group_Diff_Function\UP_DOWN\GO\NL-VS-L1.F.html#gene295) | O-methyltransferase activity | 2 (0.16%) | 7 (0.2%) | 0.775695 | 0.999471 |
| 296 | [GO:0015144](file:///E:\2018-7-3%E7%81%AB%E9%BE%99%E6%9E%9C%E8%BD%AC%E5%BD%95%E7%BB%84%E6%B5%8B%E5%BA%8F\%E5%AE%8C%E6%95%B4%E7%89%88%E6%95%B0%E6%8D%AE\GDR3855-Hylocereus_undulatus_Britt-12-RNAseq_result\4_Function\2_Group_Diff_Function\UP_DOWN\GO\NL-VS-L1.F.html#gene296) | carbohydrate transmembrane transporter activity | 2 (0.16%) | 7 (0.2%) | 0.775695 | 0.999471 |
| 297 | [GO:0016408](file:///E:\2018-7-3%E7%81%AB%E9%BE%99%E6%9E%9C%E8%BD%AC%E5%BD%95%E7%BB%84%E6%B5%8B%E5%BA%8F\%E5%AE%8C%E6%95%B4%E7%89%88%E6%95%B0%E6%8D%AE\GDR3855-Hylocereus_undulatus_Britt-12-RNAseq_result\4_Function\2_Group_Diff_Function\UP_DOWN\GO\NL-VS-L1.F.html#gene297) | C-acyltransferase activity | 2 (0.16%) | 7 (0.2%) | 0.775695 | 0.999471 |
| 298 | [GO:0016861](file:///E:\2018-7-3%E7%81%AB%E9%BE%99%E6%9E%9C%E8%BD%AC%E5%BD%95%E7%BB%84%E6%B5%8B%E5%BA%8F\%E5%AE%8C%E6%95%B4%E7%89%88%E6%95%B0%E6%8D%AE\GDR3855-Hylocereus_undulatus_Britt-12-RNAseq_result\4_Function\2_Group_Diff_Function\UP_DOWN\GO\NL-VS-L1.F.html#gene298) | intramolecular oxidoreductase activity, interconverting aldoses and ketoses | 2 (0.16%) | 7 (0.2%) | 0.775695 | 0.999471 |
| 299 | [GO:0030597](file:///E:\2018-7-3%E7%81%AB%E9%BE%99%E6%9E%9C%E8%BD%AC%E5%BD%95%E7%BB%84%E6%B5%8B%E5%BA%8F\%E5%AE%8C%E6%95%B4%E7%89%88%E6%95%B0%E6%8D%AE\GDR3855-Hylocereus_undulatus_Britt-12-RNAseq_result\4_Function\2_Group_Diff_Function\UP_DOWN\GO\NL-VS-L1.F.html#gene299) | RNA glycosylase activity | 2 (0.16%) | 7 (0.2%) | 0.775695 | 0.999471 |
| 300 | [GO:0051119](file:///E:\2018-7-3%E7%81%AB%E9%BE%99%E6%9E%9C%E8%BD%AC%E5%BD%95%E7%BB%84%E6%B5%8B%E5%BA%8F\%E5%AE%8C%E6%95%B4%E7%89%88%E6%95%B0%E6%8D%AE\GDR3855-Hylocereus_undulatus_Britt-12-RNAseq_result\4_Function\2_Group_Diff_Function\UP_DOWN\GO\NL-VS-L1.F.html#gene300) | sugar transmembrane transporter activity | 2 (0.16%) | 7 (0.2%) | 0.775695 | 0.999471 |
| 301 | [GO:1901476](file:///E:\2018-7-3%E7%81%AB%E9%BE%99%E6%9E%9C%E8%BD%AC%E5%BD%95%E7%BB%84%E6%B5%8B%E5%BA%8F\%E5%AE%8C%E6%95%B4%E7%89%88%E6%95%B0%E6%8D%AE\GDR3855-Hylocereus_undulatus_Britt-12-RNAseq_result\4_Function\2_Group_Diff_Function\UP_DOWN\GO\NL-VS-L1.F.html#gene301) | carbohydrate transporter activity | 2 (0.16%) | 7 (0.2%) | 0.775695 | 0.999471 |
| 302 | [GO:0016705](file:///E:\2018-7-3%E7%81%AB%E9%BE%99%E6%9E%9C%E8%BD%AC%E5%BD%95%E7%BB%84%E6%B5%8B%E5%BA%8F\%E5%AE%8C%E6%95%B4%E7%89%88%E6%95%B0%E6%8D%AE\GDR3855-Hylocereus_undulatus_Britt-12-RNAseq_result\4_Function\2_Group_Diff_Function\UP_DOWN\GO\NL-VS-L1.F.html#gene302) | oxidoreductase activity, acting on paired donors, with incorporation or reduction of molecular oxygen | 25 (2.05%) | 78 (2.27%) | 0.778590 | 0.999471 |
| 303 | [GO:0016859](file:///E:\2018-7-3%E7%81%AB%E9%BE%99%E6%9E%9C%E8%BD%AC%E5%BD%95%E7%BB%84%E6%B5%8B%E5%BA%8F\%E5%AE%8C%E6%95%B4%E7%89%88%E6%95%B0%E6%8D%AE\GDR3855-Hylocereus_undulatus_Britt-12-RNAseq_result\4_Function\2_Group_Diff_Function\UP_DOWN\GO\NL-VS-L1.F.html#gene303) | cis-trans isomerase activity | 5 (0.41%) | 17 (0.5%) | 0.779977 | 0.999471 |
| 304 | [GO:0022804](file:///E:\2018-7-3%E7%81%AB%E9%BE%99%E6%9E%9C%E8%BD%AC%E5%BD%95%E7%BB%84%E6%B5%8B%E5%BA%8F\%E5%AE%8C%E6%95%B4%E7%89%88%E6%95%B0%E6%8D%AE\GDR3855-Hylocereus_undulatus_Britt-12-RNAseq_result\4_Function\2_Group_Diff_Function\UP_DOWN\GO\NL-VS-L1.F.html#gene304) | active transmembrane transporter activity | 28 (2.3%) | 87 (2.53%) | 0.780237 | 0.999471 |
| 305 | [GO:0016788](file:///E:\2018-7-3%E7%81%AB%E9%BE%99%E6%9E%9C%E8%BD%AC%E5%BD%95%E7%BB%84%E6%B5%8B%E5%BA%8F\%E5%AE%8C%E6%95%B4%E7%89%88%E6%95%B0%E6%8D%AE\GDR3855-Hylocereus_undulatus_Britt-12-RNAseq_result\4_Function\2_Group_Diff_Function\UP_DOWN\GO\NL-VS-L1.F.html#gene305) | hydrolase activity, acting on ester bonds | 53 (4.34%) | 161 (4.69%) | 0.786764 | 0.999471 |
| 306 | [GO:0016875](file:///E:\2018-7-3%E7%81%AB%E9%BE%99%E6%9E%9C%E8%BD%AC%E5%BD%95%E7%BB%84%E6%B5%8B%E5%BA%8F\%E5%AE%8C%E6%95%B4%E7%89%88%E6%95%B0%E6%8D%AE\GDR3855-Hylocereus_undulatus_Britt-12-RNAseq_result\4_Function\2_Group_Diff_Function\UP_DOWN\GO\NL-VS-L1.F.html#gene306) | ligase activity, forming carbon-oxygen bonds | 4 (0.33%) | 14 (0.41%) | 0.792455 | 0.999471 |
| 307 | [GO:0016876](file:///E:\2018-7-3%E7%81%AB%E9%BE%99%E6%9E%9C%E8%BD%AC%E5%BD%95%E7%BB%84%E6%B5%8B%E5%BA%8F\%E5%AE%8C%E6%95%B4%E7%89%88%E6%95%B0%E6%8D%AE\GDR3855-Hylocereus_undulatus_Britt-12-RNAseq_result\4_Function\2_Group_Diff_Function\UP_DOWN\GO\NL-VS-L1.F.html#gene307) | ligase activity, forming aminoacyl-tRNA and related compounds | 4 (0.33%) | 14 (0.41%) | 0.792455 | 0.999471 |
| 308 | [GO:0004871](file:///E:\2018-7-3%E7%81%AB%E9%BE%99%E6%9E%9C%E8%BD%AC%E5%BD%95%E7%BB%84%E6%B5%8B%E5%BA%8F\%E5%AE%8C%E6%95%B4%E7%89%88%E6%95%B0%E6%8D%AE\GDR3855-Hylocereus_undulatus_Britt-12-RNAseq_result\4_Function\2_Group_Diff_Function\UP_DOWN\GO\NL-VS-L1.F.html#gene308) | signal transducer activity | 17 (1.39%) | 55 (1.6%) | 0.805905 | 0.999471 |
| 309 | [GO:0004674](file:///E:\2018-7-3%E7%81%AB%E9%BE%99%E6%9E%9C%E8%BD%AC%E5%BD%95%E7%BB%84%E6%B5%8B%E5%BA%8F\%E5%AE%8C%E6%95%B4%E7%89%88%E6%95%B0%E6%8D%AE\GDR3855-Hylocereus_undulatus_Britt-12-RNAseq_result\4_Function\2_Group_Diff_Function\UP_DOWN\GO\NL-VS-L1.F.html#gene309) | protein serine/threonine kinase activity | 39 (3.2%) | 121 (3.53%) | 0.807970 | 0.999471 |
| 310 | [GO:0097159](file:///E:\2018-7-3%E7%81%AB%E9%BE%99%E6%9E%9C%E8%BD%AC%E5%BD%95%E7%BB%84%E6%B5%8B%E5%BA%8F\%E5%AE%8C%E6%95%B4%E7%89%88%E6%95%B0%E6%8D%AE\GDR3855-Hylocereus_undulatus_Britt-12-RNAseq_result\4_Function\2_Group_Diff_Function\UP_DOWN\GO\NL-VS-L1.F.html#gene310) | organic cyclic compound binding | 335 (27.46%) | 974 (28.38%) | 0.823307 | 0.999471 |
| 311 | [GO:0016836](file:///E:\2018-7-3%E7%81%AB%E9%BE%99%E6%9E%9C%E8%BD%AC%E5%BD%95%E7%BB%84%E6%B5%8B%E5%BA%8F\%E5%AE%8C%E6%95%B4%E7%89%88%E6%95%B0%E6%8D%AE\GDR3855-Hylocereus_undulatus_Britt-12-RNAseq_result\4_Function\2_Group_Diff_Function\UP_DOWN\GO\NL-VS-L1.F.html#gene311) | hydro-lyase activity | 5 (0.41%) | 18 (0.52%) | 0.824771 | 0.999471 |
| 312 | [GO:0004521](file:///E:\2018-7-3%E7%81%AB%E9%BE%99%E6%9E%9C%E8%BD%AC%E5%BD%95%E7%BB%84%E6%B5%8B%E5%BA%8F\%E5%AE%8C%E6%95%B4%E7%89%88%E6%95%B0%E6%8D%AE\GDR3855-Hylocereus_undulatus_Britt-12-RNAseq_result\4_Function\2_Group_Diff_Function\UP_DOWN\GO\NL-VS-L1.F.html#gene312) | endoribonuclease activity | 1 (0.08%) | 4 (0.12%) | 0.827602 | 0.999471 |
| 313 | [GO:0008374](file:///E:\2018-7-3%E7%81%AB%E9%BE%99%E6%9E%9C%E8%BD%AC%E5%BD%95%E7%BB%84%E6%B5%8B%E5%BA%8F\%E5%AE%8C%E6%95%B4%E7%89%88%E6%95%B0%E6%8D%AE\GDR3855-Hylocereus_undulatus_Britt-12-RNAseq_result\4_Function\2_Group_Diff_Function\UP_DOWN\GO\NL-VS-L1.F.html#gene313) | O-acyltransferase activity | 1 (0.08%) | 4 (0.12%) | 0.827602 | 0.999471 |
| 314 | [GO:0015645](file:///E:\2018-7-3%E7%81%AB%E9%BE%99%E6%9E%9C%E8%BD%AC%E5%BD%95%E7%BB%84%E6%B5%8B%E5%BA%8F\%E5%AE%8C%E6%95%B4%E7%89%88%E6%95%B0%E6%8D%AE\GDR3855-Hylocereus_undulatus_Britt-12-RNAseq_result\4_Function\2_Group_Diff_Function\UP_DOWN\GO\NL-VS-L1.F.html#gene314) | fatty acid ligase activity | 1 (0.08%) | 4 (0.12%) | 0.827602 | 0.999471 |
| 315 | [GO:0016634](file:///E:\2018-7-3%E7%81%AB%E9%BE%99%E6%9E%9C%E8%BD%AC%E5%BD%95%E7%BB%84%E6%B5%8B%E5%BA%8F\%E5%AE%8C%E6%95%B4%E7%89%88%E6%95%B0%E6%8D%AE\GDR3855-Hylocereus_undulatus_Britt-12-RNAseq_result\4_Function\2_Group_Diff_Function\UP_DOWN\GO\NL-VS-L1.F.html#gene315) | oxidoreductase activity, acting on the CH-CH group of donors, oxygen as acceptor | 1 (0.08%) | 4 (0.12%) | 0.827602 | 0.999471 |
| 316 | [GO:0016661](file:///E:\2018-7-3%E7%81%AB%E9%BE%99%E6%9E%9C%E8%BD%AC%E5%BD%95%E7%BB%84%E6%B5%8B%E5%BA%8F\%E5%AE%8C%E6%95%B4%E7%89%88%E6%95%B0%E6%8D%AE\GDR3855-Hylocereus_undulatus_Britt-12-RNAseq_result\4_Function\2_Group_Diff_Function\UP_DOWN\GO\NL-VS-L1.F.html#gene316) | oxidoreductase activity, acting on other nitrogenous compounds as donors | 1 (0.08%) | 4 (0.12%) | 0.827602 | 0.999471 |
| 317 | [GO:0016782](file:///E:\2018-7-3%E7%81%AB%E9%BE%99%E6%9E%9C%E8%BD%AC%E5%BD%95%E7%BB%84%E6%B5%8B%E5%BA%8F\%E5%AE%8C%E6%95%B4%E7%89%88%E6%95%B0%E6%8D%AE\GDR3855-Hylocereus_undulatus_Britt-12-RNAseq_result\4_Function\2_Group_Diff_Function\UP_DOWN\GO\NL-VS-L1.F.html#gene317) | transferase activity, transferring sulfur-containing groups | 1 (0.08%) | 4 (0.12%) | 0.827602 | 0.999471 |
| 318 | [GO:0016783](file:///E:\2018-7-3%E7%81%AB%E9%BE%99%E6%9E%9C%E8%BD%AC%E5%BD%95%E7%BB%84%E6%B5%8B%E5%BA%8F\%E5%AE%8C%E6%95%B4%E7%89%88%E6%95%B0%E6%8D%AE\GDR3855-Hylocereus_undulatus_Britt-12-RNAseq_result\4_Function\2_Group_Diff_Function\UP_DOWN\GO\NL-VS-L1.F.html#gene318) | sulfurtransferase activity | 1 (0.08%) | 4 (0.12%) | 0.827602 | 0.999471 |
| 319 | [GO:0016899](file:///E:\2018-7-3%E7%81%AB%E9%BE%99%E6%9E%9C%E8%BD%AC%E5%BD%95%E7%BB%84%E6%B5%8B%E5%BA%8F\%E5%AE%8C%E6%95%B4%E7%89%88%E6%95%B0%E6%8D%AE\GDR3855-Hylocereus_undulatus_Britt-12-RNAseq_result\4_Function\2_Group_Diff_Function\UP_DOWN\GO\NL-VS-L1.F.html#gene319) | oxidoreductase activity, acting on the CH-OH group of donors, oxygen as acceptor | 1 (0.08%) | 4 (0.12%) | 0.827602 | 0.999471 |
| 320 | [GO:0038023](file:///E:\2018-7-3%E7%81%AB%E9%BE%99%E6%9E%9C%E8%BD%AC%E5%BD%95%E7%BB%84%E6%B5%8B%E5%BA%8F\%E5%AE%8C%E6%95%B4%E7%89%88%E6%95%B0%E6%8D%AE\GDR3855-Hylocereus_undulatus_Britt-12-RNAseq_result\4_Function\2_Group_Diff_Function\UP_DOWN\GO\NL-VS-L1.F.html#gene320) | signaling receptor activity | 1 (0.08%) | 4 (0.12%) | 0.827602 | 0.999471 |
| 321 | [GO:0046992](file:///E:\2018-7-3%E7%81%AB%E9%BE%99%E6%9E%9C%E8%BD%AC%E5%BD%95%E7%BB%84%E6%B5%8B%E5%BA%8F\%E5%AE%8C%E6%95%B4%E7%89%88%E6%95%B0%E6%8D%AE\GDR3855-Hylocereus_undulatus_Britt-12-RNAseq_result\4_Function\2_Group_Diff_Function\UP_DOWN\GO\NL-VS-L1.F.html#gene321) | oxidoreductase activity, acting on X-H and Y-H to form an X-Y bond | 1 (0.08%) | 4 (0.12%) | 0.827602 | 0.999471 |
| 322 | [GO:0046993](file:///E:\2018-7-3%E7%81%AB%E9%BE%99%E6%9E%9C%E8%BD%AC%E5%BD%95%E7%BB%84%E6%B5%8B%E5%BA%8F\%E5%AE%8C%E6%95%B4%E7%89%88%E6%95%B0%E6%8D%AE\GDR3855-Hylocereus_undulatus_Britt-12-RNAseq_result\4_Function\2_Group_Diff_Function\UP_DOWN\GO\NL-VS-L1.F.html#gene322) | oxidoreductase activity, acting on X-H and Y-H to form an X-Y bond, with oxygen as acceptor | 1 (0.08%) | 4 (0.12%) | 0.827602 | 0.999471 |
| 323 | [GO:0004519](file:///E:\2018-7-3%E7%81%AB%E9%BE%99%E6%9E%9C%E8%BD%AC%E5%BD%95%E7%BB%84%E6%B5%8B%E5%BA%8F\%E5%AE%8C%E6%95%B4%E7%89%88%E6%95%B0%E6%8D%AE\GDR3855-Hylocereus_undulatus_Britt-12-RNAseq_result\4_Function\2_Group_Diff_Function\UP_DOWN\GO\NL-VS-L1.F.html#gene323) | endonuclease activity | 2 (0.16%) | 8 (0.23%) | 0.839140 | 0.999471 |
| 324 | [GO:0016860](file:///E:\2018-7-3%E7%81%AB%E9%BE%99%E6%9E%9C%E8%BD%AC%E5%BD%95%E7%BB%84%E6%B5%8B%E5%BA%8F\%E5%AE%8C%E6%95%B4%E7%89%88%E6%95%B0%E6%8D%AE\GDR3855-Hylocereus_undulatus_Britt-12-RNAseq_result\4_Function\2_Group_Diff_Function\UP_DOWN\GO\NL-VS-L1.F.html#gene324) | intramolecular oxidoreductase activity | 2 (0.16%) | 8 (0.23%) | 0.839140 | 0.999471 |
| 325 | [GO:0043168](file:///E:\2018-7-3%E7%81%AB%E9%BE%99%E6%9E%9C%E8%BD%AC%E5%BD%95%E7%BB%84%E6%B5%8B%E5%BA%8F\%E5%AE%8C%E6%95%B4%E7%89%88%E6%95%B0%E6%8D%AE\GDR3855-Hylocereus_undulatus_Britt-12-RNAseq_result\4_Function\2_Group_Diff_Function\UP_DOWN\GO\NL-VS-L1.F.html#gene325) | anion binding | 19 (1.56%) | 63 (1.84%) | 0.849885 | 0.999471 |
| 326 | [GO:0016709](file:///E:\2018-7-3%E7%81%AB%E9%BE%99%E6%9E%9C%E8%BD%AC%E5%BD%95%E7%BB%84%E6%B5%8B%E5%BA%8F\%E5%AE%8C%E6%95%B4%E7%89%88%E6%95%B0%E6%8D%AE\GDR3855-Hylocereus_undulatus_Britt-12-RNAseq_result\4_Function\2_Group_Diff_Function\UP_DOWN\GO\NL-VS-L1.F.html#gene326) | oxidoreductase activity, acting on paired donors, with incorporation or reduction of molecular oxygen, NAD(P)H as one donor, and incorporation of one atom of oxygen | 5 (0.41%) | 19 (0.55%) | 0.861867 | 0.999471 |
| 327 | [GO:0016874](file:///E:\2018-7-3%E7%81%AB%E9%BE%99%E6%9E%9C%E8%BD%AC%E5%BD%95%E7%BB%84%E6%B5%8B%E5%BA%8F\%E5%AE%8C%E6%95%B4%E7%89%88%E6%95%B0%E6%8D%AE\GDR3855-Hylocereus_undulatus_Britt-12-RNAseq_result\4_Function\2_Group_Diff_Function\UP_DOWN\GO\NL-VS-L1.F.html#gene327) | ligase activity | 22 (1.8%) | 73 (2.13%) | 0.864932 | 0.999471 |
| 328 | [GO:0051540](file:///E:\2018-7-3%E7%81%AB%E9%BE%99%E6%9E%9C%E8%BD%AC%E5%BD%95%E7%BB%84%E6%B5%8B%E5%BA%8F\%E5%AE%8C%E6%95%B4%E7%89%88%E6%95%B0%E6%8D%AE\GDR3855-Hylocereus_undulatus_Britt-12-RNAseq_result\4_Function\2_Group_Diff_Function\UP_DOWN\GO\NL-VS-L1.F.html#gene328) | metal cluster binding | 11 (0.9%) | 39 (1.14%) | 0.872311 | 0.999471 |
| 329 | [GO:0008233](file:///E:\2018-7-3%E7%81%AB%E9%BE%99%E6%9E%9C%E8%BD%AC%E5%BD%95%E7%BB%84%E6%B5%8B%E5%BA%8F\%E5%AE%8C%E6%95%B4%E7%89%88%E6%95%B0%E6%8D%AE\GDR3855-Hylocereus_undulatus_Britt-12-RNAseq_result\4_Function\2_Group_Diff_Function\UP_DOWN\GO\NL-VS-L1.F.html#gene329) | peptidase activity | 26 (2.13%) | 86 (2.51%) | 0.877222 | 0.999471 |
| 330 | [GO:0004721](file:///E:\2018-7-3%E7%81%AB%E9%BE%99%E6%9E%9C%E8%BD%AC%E5%BD%95%E7%BB%84%E6%B5%8B%E5%BA%8F\%E5%AE%8C%E6%95%B4%E7%89%88%E6%95%B0%E6%8D%AE\GDR3855-Hylocereus_undulatus_Britt-12-RNAseq_result\4_Function\2_Group_Diff_Function\UP_DOWN\GO\NL-VS-L1.F.html#gene330) | phosphoprotein phosphatase activity | 6 (0.49%) | 23 (0.67%) | 0.880993 | 0.999471 |
| 331 | [GO:0008168](file:///E:\2018-7-3%E7%81%AB%E9%BE%99%E6%9E%9C%E8%BD%AC%E5%BD%95%E7%BB%84%E6%B5%8B%E5%BA%8F\%E5%AE%8C%E6%95%B4%E7%89%88%E6%95%B0%E6%8D%AE\GDR3855-Hylocereus_undulatus_Britt-12-RNAseq_result\4_Function\2_Group_Diff_Function\UP_DOWN\GO\NL-VS-L1.F.html#gene331) | methyltransferase activity | 14 (1.15%) | 49 (1.43%) | 0.881932 | 0.999471 |
| 332 | [GO:0016820](file:///E:\2018-7-3%E7%81%AB%E9%BE%99%E6%9E%9C%E8%BD%AC%E5%BD%95%E7%BB%84%E6%B5%8B%E5%BA%8F\%E5%AE%8C%E6%95%B4%E7%89%88%E6%95%B0%E6%8D%AE\GDR3855-Hylocereus_undulatus_Britt-12-RNAseq_result\4_Function\2_Group_Diff_Function\UP_DOWN\GO\NL-VS-L1.F.html#gene332) | hydrolase activity, acting on acid anhydrides, catalyzing transmembrane movement of substances | 14 (1.15%) | 49 (1.43%) | 0.881932 | 0.999471 |
| 333 | [GO:0042626](file:///E:\2018-7-3%E7%81%AB%E9%BE%99%E6%9E%9C%E8%BD%AC%E5%BD%95%E7%BB%84%E6%B5%8B%E5%BA%8F\%E5%AE%8C%E6%95%B4%E7%89%88%E6%95%B0%E6%8D%AE\GDR3855-Hylocereus_undulatus_Britt-12-RNAseq_result\4_Function\2_Group_Diff_Function\UP_DOWN\GO\NL-VS-L1.F.html#gene333) | ATPase activity, coupled to transmembrane movement of substances | 14 (1.15%) | 49 (1.43%) | 0.881932 | 0.999471 |
| 334 | [GO:0043492](file:///E:\2018-7-3%E7%81%AB%E9%BE%99%E6%9E%9C%E8%BD%AC%E5%BD%95%E7%BB%84%E6%B5%8B%E5%BA%8F\%E5%AE%8C%E6%95%B4%E7%89%88%E6%95%B0%E6%8D%AE\GDR3855-Hylocereus_undulatus_Britt-12-RNAseq_result\4_Function\2_Group_Diff_Function\UP_DOWN\GO\NL-VS-L1.F.html#gene334) | ATPase activity, coupled to movement of substances | 14 (1.15%) | 49 (1.43%) | 0.881932 | 0.999471 |
| 335 | [GO:0016616](file:///E:\2018-7-3%E7%81%AB%E9%BE%99%E6%9E%9C%E8%BD%AC%E5%BD%95%E7%BB%84%E6%B5%8B%E5%BA%8F\%E5%AE%8C%E6%95%B4%E7%89%88%E6%95%B0%E6%8D%AE\GDR3855-Hylocereus_undulatus_Britt-12-RNAseq_result\4_Function\2_Group_Diff_Function\UP_DOWN\GO\NL-VS-L1.F.html#gene335) | oxidoreductase activity, acting on the CH-OH group of donors, NAD or NADP as acceptor | 13 (1.07%) | 46 (1.34%) | 0.885387 | 0.999471 |
| 336 | [GO:0004620](file:///E:\2018-7-3%E7%81%AB%E9%BE%99%E6%9E%9C%E8%BD%AC%E5%BD%95%E7%BB%84%E6%B5%8B%E5%BA%8F\%E5%AE%8C%E6%95%B4%E7%89%88%E6%95%B0%E6%8D%AE\GDR3855-Hylocereus_undulatus_Britt-12-RNAseq_result\4_Function\2_Group_Diff_Function\UP_DOWN\GO\NL-VS-L1.F.html#gene336) | phospholipase activity | 2 (0.16%) | 9 (0.26%) | 0.885855 | 0.999471 |
| 337 | [GO:0016209](file:///E:\2018-7-3%E7%81%AB%E9%BE%99%E6%9E%9C%E8%BD%AC%E5%BD%95%E7%BB%84%E6%B5%8B%E5%BA%8F\%E5%AE%8C%E6%95%B4%E7%89%88%E6%95%B0%E6%8D%AE\GDR3855-Hylocereus_undulatus_Britt-12-RNAseq_result\4_Function\2_Group_Diff_Function\UP_DOWN\GO\NL-VS-L1.F.html#gene337) | antioxidant activity | 2 (0.16%) | 9 (0.26%) | 0.885855 | 0.999471 |
| 338 | [GO:0016298](file:///E:\2018-7-3%E7%81%AB%E9%BE%99%E6%9E%9C%E8%BD%AC%E5%BD%95%E7%BB%84%E6%B5%8B%E5%BA%8F\%E5%AE%8C%E6%95%B4%E7%89%88%E6%95%B0%E6%8D%AE\GDR3855-Hylocereus_undulatus_Britt-12-RNAseq_result\4_Function\2_Group_Diff_Function\UP_DOWN\GO\NL-VS-L1.F.html#gene338) | lipase activity | 2 (0.16%) | 9 (0.26%) | 0.885855 | 0.999471 |
| 339 | [GO:0016645](file:///E:\2018-7-3%E7%81%AB%E9%BE%99%E6%9E%9C%E8%BD%AC%E5%BD%95%E7%BB%84%E6%B5%8B%E5%BA%8F\%E5%AE%8C%E6%95%B4%E7%89%88%E6%95%B0%E6%8D%AE\GDR3855-Hylocereus_undulatus_Britt-12-RNAseq_result\4_Function\2_Group_Diff_Function\UP_DOWN\GO\NL-VS-L1.F.html#gene339) | oxidoreductase activity, acting on the CH-NH group of donors | 2 (0.16%) | 9 (0.26%) | 0.885855 | 0.999471 |
| 340 | [GO:0016799](file:///E:\2018-7-3%E7%81%AB%E9%BE%99%E6%9E%9C%E8%BD%AC%E5%BD%95%E7%BB%84%E6%B5%8B%E5%BA%8F\%E5%AE%8C%E6%95%B4%E7%89%88%E6%95%B0%E6%8D%AE\GDR3855-Hylocereus_undulatus_Britt-12-RNAseq_result\4_Function\2_Group_Diff_Function\UP_DOWN\GO\NL-VS-L1.F.html#gene340) | hydrolase activity, hydrolyzing N-glycosyl compounds | 2 (0.16%) | 9 (0.26%) | 0.885855 | 0.999471 |
| 341 | [GO:0016814](file:///E:\2018-7-3%E7%81%AB%E9%BE%99%E6%9E%9C%E8%BD%AC%E5%BD%95%E7%BB%84%E6%B5%8B%E5%BA%8F\%E5%AE%8C%E6%95%B4%E7%89%88%E6%95%B0%E6%8D%AE\GDR3855-Hylocereus_undulatus_Britt-12-RNAseq_result\4_Function\2_Group_Diff_Function\UP_DOWN\GO\NL-VS-L1.F.html#gene341) | hydrolase activity, acting on carbon-nitrogen (but not peptide) bonds, in cyclic amidines | 2 (0.16%) | 9 (0.26%) | 0.885855 | 0.999471 |
| 342 | [GO:0004177](file:///E:\2018-7-3%E7%81%AB%E9%BE%99%E6%9E%9C%E8%BD%AC%E5%BD%95%E7%BB%84%E6%B5%8B%E5%BA%8F\%E5%AE%8C%E6%95%B4%E7%89%88%E6%95%B0%E6%8D%AE\GDR3855-Hylocereus_undulatus_Britt-12-RNAseq_result\4_Function\2_Group_Diff_Function\UP_DOWN\GO\NL-VS-L1.F.html#gene342) | aminopeptidase activity | 1 (0.08%) | 5 (0.15%) | 0.888957 | 0.999471 |
| 343 | [GO:0004448](file:///E:\2018-7-3%E7%81%AB%E9%BE%99%E6%9E%9C%E8%BD%AC%E5%BD%95%E7%BB%84%E6%B5%8B%E5%BA%8F\%E5%AE%8C%E6%95%B4%E7%89%88%E6%95%B0%E6%8D%AE\GDR3855-Hylocereus_undulatus_Britt-12-RNAseq_result\4_Function\2_Group_Diff_Function\UP_DOWN\GO\NL-VS-L1.F.html#gene343) | isocitrate dehydrogenase activity | 1 (0.08%) | 5 (0.15%) | 0.888957 | 0.999471 |
| 344 | [GO:0004540](file:///E:\2018-7-3%E7%81%AB%E9%BE%99%E6%9E%9C%E8%BD%AC%E5%BD%95%E7%BB%84%E6%B5%8B%E5%BA%8F\%E5%AE%8C%E6%95%B4%E7%89%88%E6%95%B0%E6%8D%AE\GDR3855-Hylocereus_undulatus_Britt-12-RNAseq_result\4_Function\2_Group_Diff_Function\UP_DOWN\GO\NL-VS-L1.F.html#gene344) | ribonuclease activity | 1 (0.08%) | 5 (0.15%) | 0.888957 | 0.999471 |
| 345 | [GO:0004779](file:///E:\2018-7-3%E7%81%AB%E9%BE%99%E6%9E%9C%E8%BD%AC%E5%BD%95%E7%BB%84%E6%B5%8B%E5%BA%8F\%E5%AE%8C%E6%95%B4%E7%89%88%E6%95%B0%E6%8D%AE\GDR3855-Hylocereus_undulatus_Britt-12-RNAseq_result\4_Function\2_Group_Diff_Function\UP_DOWN\GO\NL-VS-L1.F.html#gene345) | sulfate adenylyltransferase activity | 1 (0.08%) | 5 (0.15%) | 0.888957 | 0.999471 |
| 346 | [GO:0008320](file:///E:\2018-7-3%E7%81%AB%E9%BE%99%E6%9E%9C%E8%BD%AC%E5%BD%95%E7%BB%84%E6%B5%8B%E5%BA%8F\%E5%AE%8C%E6%95%B4%E7%89%88%E6%95%B0%E6%8D%AE\GDR3855-Hylocereus_undulatus_Britt-12-RNAseq_result\4_Function\2_Group_Diff_Function\UP_DOWN\GO\NL-VS-L1.F.html#gene346) | protein transmembrane transporter activity | 1 (0.08%) | 5 (0.15%) | 0.888957 | 0.999471 |
| 347 | [GO:0008565](file:///E:\2018-7-3%E7%81%AB%E9%BE%99%E6%9E%9C%E8%BD%AC%E5%BD%95%E7%BB%84%E6%B5%8B%E5%BA%8F\%E5%AE%8C%E6%95%B4%E7%89%88%E6%95%B0%E6%8D%AE\GDR3855-Hylocereus_undulatus_Britt-12-RNAseq_result\4_Function\2_Group_Diff_Function\UP_DOWN\GO\NL-VS-L1.F.html#gene347) | protein transporter activity | 1 (0.08%) | 5 (0.15%) | 0.888957 | 0.999471 |
| 348 | [GO:0022884](file:///E:\2018-7-3%E7%81%AB%E9%BE%99%E6%9E%9C%E8%BD%AC%E5%BD%95%E7%BB%84%E6%B5%8B%E5%BA%8F\%E5%AE%8C%E6%95%B4%E7%89%88%E6%95%B0%E6%8D%AE\GDR3855-Hylocereus_undulatus_Britt-12-RNAseq_result\4_Function\2_Group_Diff_Function\UP_DOWN\GO\NL-VS-L1.F.html#gene348) | macromolecule transmembrane transporter activity | 1 (0.08%) | 5 (0.15%) | 0.888957 | 0.999471 |
| 349 | [GO:0043021](file:///E:\2018-7-3%E7%81%AB%E9%BE%99%E6%9E%9C%E8%BD%AC%E5%BD%95%E7%BB%84%E6%B5%8B%E5%BA%8F\%E5%AE%8C%E6%95%B4%E7%89%88%E6%95%B0%E6%8D%AE\GDR3855-Hylocereus_undulatus_Britt-12-RNAseq_result\4_Function\2_Group_Diff_Function\UP_DOWN\GO\NL-VS-L1.F.html#gene349) | ribonucleoprotein complex binding | 1 (0.08%) | 5 (0.15%) | 0.888957 | 0.999471 |
| 350 | [GO:0015078](file:///E:\2018-7-3%E7%81%AB%E9%BE%99%E6%9E%9C%E8%BD%AC%E5%BD%95%E7%BB%84%E6%B5%8B%E5%BA%8F\%E5%AE%8C%E6%95%B4%E7%89%88%E6%95%B0%E6%8D%AE\GDR3855-Hylocereus_undulatus_Britt-12-RNAseq_result\4_Function\2_Group_Diff_Function\UP_DOWN\GO\NL-VS-L1.F.html#gene350) | hydrogen ion transmembrane transporter activity | 8 (0.66%) | 30 (0.87%) | 0.889427 | 0.999471 |
| 351 | [GO:0015399](file:///E:\2018-7-3%E7%81%AB%E9%BE%99%E6%9E%9C%E8%BD%AC%E5%BD%95%E7%BB%84%E6%B5%8B%E5%BA%8F\%E5%AE%8C%E6%95%B4%E7%89%88%E6%95%B0%E6%8D%AE\GDR3855-Hylocereus_undulatus_Britt-12-RNAseq_result\4_Function\2_Group_Diff_Function\UP_DOWN\GO\NL-VS-L1.F.html#gene351) | primary active transmembrane transporter activity | 17 (1.39%) | 59 (1.72%) | 0.891615 | 0.999471 |
| 352 | [GO:0015405](file:///E:\2018-7-3%E7%81%AB%E9%BE%99%E6%9E%9C%E8%BD%AC%E5%BD%95%E7%BB%84%E6%B5%8B%E5%BA%8F\%E5%AE%8C%E6%95%B4%E7%89%88%E6%95%B0%E6%8D%AE\GDR3855-Hylocereus_undulatus_Britt-12-RNAseq_result\4_Function\2_Group_Diff_Function\UP_DOWN\GO\NL-VS-L1.F.html#gene352) | P-P-bond-hydrolysis-driven transmembrane transporter activity | 17 (1.39%) | 59 (1.72%) | 0.891615 | 0.999471 |
| 353 | [GO:0019899](file:///E:\2018-7-3%E7%81%AB%E9%BE%99%E6%9E%9C%E8%BD%AC%E5%BD%95%E7%BB%84%E6%B5%8B%E5%BA%8F\%E5%AE%8C%E6%95%B4%E7%89%88%E6%95%B0%E6%8D%AE\GDR3855-Hylocereus_undulatus_Britt-12-RNAseq_result\4_Function\2_Group_Diff_Function\UP_DOWN\GO\NL-VS-L1.F.html#gene353) | enzyme binding | 5 (0.41%) | 20 (0.58%) | 0.892128 | 0.999471 |
| 354 | [GO:0004812](file:///E:\2018-7-3%E7%81%AB%E9%BE%99%E6%9E%9C%E8%BD%AC%E5%BD%95%E7%BB%84%E6%B5%8B%E5%BA%8F\%E5%AE%8C%E6%95%B4%E7%89%88%E6%95%B0%E6%8D%AE\GDR3855-Hylocereus_undulatus_Britt-12-RNAseq_result\4_Function\2_Group_Diff_Function\UP_DOWN\GO\NL-VS-L1.F.html#gene354) | aminoacyl-tRNA ligase activity | 3 (0.25%) | 13 (0.38%) | 0.894800 | 0.999471 |
| 355 | [GO:0008238](file:///E:\2018-7-3%E7%81%AB%E9%BE%99%E6%9E%9C%E8%BD%AC%E5%BD%95%E7%BB%84%E6%B5%8B%E5%BA%8F\%E5%AE%8C%E6%95%B4%E7%89%88%E6%95%B0%E6%8D%AE\GDR3855-Hylocereus_undulatus_Britt-12-RNAseq_result\4_Function\2_Group_Diff_Function\UP_DOWN\GO\NL-VS-L1.F.html#gene355) | exopeptidase activity | 3 (0.25%) | 13 (0.38%) | 0.894800 | 0.999471 |
| 356 | [GO:0016765](file:///E:\2018-7-3%E7%81%AB%E9%BE%99%E6%9E%9C%E8%BD%AC%E5%BD%95%E7%BB%84%E6%B5%8B%E5%BA%8F\%E5%AE%8C%E6%95%B4%E7%89%88%E6%95%B0%E6%8D%AE\GDR3855-Hylocereus_undulatus_Britt-12-RNAseq_result\4_Function\2_Group_Diff_Function\UP_DOWN\GO\NL-VS-L1.F.html#gene356) | transferase activity, transferring alkyl or aryl (other than methyl) groups | 3 (0.25%) | 13 (0.38%) | 0.894800 | 0.999471 |
| 357 | [GO:0017111](file:///E:\2018-7-3%E7%81%AB%E9%BE%99%E6%9E%9C%E8%BD%AC%E5%BD%95%E7%BB%84%E6%B5%8B%E5%BA%8F\%E5%AE%8C%E6%95%B4%E7%89%88%E6%95%B0%E6%8D%AE\GDR3855-Hylocereus_undulatus_Britt-12-RNAseq_result\4_Function\2_Group_Diff_Function\UP_DOWN\GO\NL-VS-L1.F.html#gene357) | nucleoside-triphosphatase activity | 77 (6.31%) | 241 (7.02%) | 0.900414 | 0.999471 |
| 358 | [GO:0008757](file:///E:\2018-7-3%E7%81%AB%E9%BE%99%E6%9E%9C%E8%BD%AC%E5%BD%95%E7%BB%84%E6%B5%8B%E5%BA%8F\%E5%AE%8C%E6%95%B4%E7%89%88%E6%95%B0%E6%8D%AE\GDR3855-Hylocereus_undulatus_Britt-12-RNAseq_result\4_Function\2_Group_Diff_Function\UP_DOWN\GO\NL-VS-L1.F.html#gene358) | S-adenosylmethionine-dependent methyltransferase activity | 4 (0.33%) | 17 (0.5%) | 0.905776 | 0.999471 |
| 359 | [GO:0016810](file:///E:\2018-7-3%E7%81%AB%E9%BE%99%E6%9E%9C%E8%BD%AC%E5%BD%95%E7%BB%84%E6%B5%8B%E5%BA%8F\%E5%AE%8C%E6%95%B4%E7%89%88%E6%95%B0%E6%8D%AE\GDR3855-Hylocereus_undulatus_Britt-12-RNAseq_result\4_Function\2_Group_Diff_Function\UP_DOWN\GO\NL-VS-L1.F.html#gene359) | hydrolase activity, acting on carbon-nitrogen (but not peptide) bonds | 6 (0.49%) | 24 (0.7%) | 0.905977 | 0.999471 |
| 360 | [GO:0008324](file:///E:\2018-7-3%E7%81%AB%E9%BE%99%E6%9E%9C%E8%BD%AC%E5%BD%95%E7%BB%84%E6%B5%8B%E5%BA%8F\%E5%AE%8C%E6%95%B4%E7%89%88%E6%95%B0%E6%8D%AE\GDR3855-Hylocereus_undulatus_Britt-12-RNAseq_result\4_Function\2_Group_Diff_Function\UP_DOWN\GO\NL-VS-L1.F.html#gene360) | cation transmembrane transporter activity | 27 (2.21%) | 92 (2.68%) | 0.916247 | 0.999471 |
| 361 | [GO:0005543](file:///E:\2018-7-3%E7%81%AB%E9%BE%99%E6%9E%9C%E8%BD%AC%E5%BD%95%E7%BB%84%E6%B5%8B%E5%BA%8F\%E5%AE%8C%E6%95%B4%E7%89%88%E6%95%B0%E6%8D%AE\GDR3855-Hylocereus_undulatus_Britt-12-RNAseq_result\4_Function\2_Group_Diff_Function\UP_DOWN\GO\NL-VS-L1.F.html#gene361) | phospholipid binding | 1 (0.08%) | 6 (0.17%) | 0.928488 | 0.999471 |
| 362 | [GO:0008375](file:///E:\2018-7-3%E7%81%AB%E9%BE%99%E6%9E%9C%E8%BD%AC%E5%BD%95%E7%BB%84%E6%B5%8B%E5%BA%8F\%E5%AE%8C%E6%95%B4%E7%89%88%E6%95%B0%E6%8D%AE\GDR3855-Hylocereus_undulatus_Britt-12-RNAseq_result\4_Function\2_Group_Diff_Function\UP_DOWN\GO\NL-VS-L1.F.html#gene362) | acetylglucosaminyltransferase activity | 1 (0.08%) | 6 (0.17%) | 0.928488 | 0.999471 |
| 363 | [GO:0017016](file:///E:\2018-7-3%E7%81%AB%E9%BE%99%E6%9E%9C%E8%BD%AC%E5%BD%95%E7%BB%84%E6%B5%8B%E5%BA%8F\%E5%AE%8C%E6%95%B4%E7%89%88%E6%95%B0%E6%8D%AE\GDR3855-Hylocereus_undulatus_Britt-12-RNAseq_result\4_Function\2_Group_Diff_Function\UP_DOWN\GO\NL-VS-L1.F.html#gene363) | Ras GTPase binding | 1 (0.08%) | 6 (0.17%) | 0.928488 | 0.999471 |
| 364 | [GO:0030247](file:///E:\2018-7-3%E7%81%AB%E9%BE%99%E6%9E%9C%E8%BD%AC%E5%BD%95%E7%BB%84%E6%B5%8B%E5%BA%8F\%E5%AE%8C%E6%95%B4%E7%89%88%E6%95%B0%E6%8D%AE\GDR3855-Hylocereus_undulatus_Britt-12-RNAseq_result\4_Function\2_Group_Diff_Function\UP_DOWN\GO\NL-VS-L1.F.html#gene364) | polysaccharide binding | 1 (0.08%) | 6 (0.17%) | 0.928488 | 0.999471 |
| 365 | [GO:0031267](file:///E:\2018-7-3%E7%81%AB%E9%BE%99%E6%9E%9C%E8%BD%AC%E5%BD%95%E7%BB%84%E6%B5%8B%E5%BA%8F\%E5%AE%8C%E6%95%B4%E7%89%88%E6%95%B0%E6%8D%AE\GDR3855-Hylocereus_undulatus_Britt-12-RNAseq_result\4_Function\2_Group_Diff_Function\UP_DOWN\GO\NL-VS-L1.F.html#gene365) | small GTPase binding | 1 (0.08%) | 6 (0.17%) | 0.928488 | 0.999471 |
| 366 | [GO:0051020](file:///E:\2018-7-3%E7%81%AB%E9%BE%99%E6%9E%9C%E8%BD%AC%E5%BD%95%E7%BB%84%E6%B5%8B%E5%BA%8F\%E5%AE%8C%E6%95%B4%E7%89%88%E6%95%B0%E6%8D%AE\GDR3855-Hylocereus_undulatus_Britt-12-RNAseq_result\4_Function\2_Group_Diff_Function\UP_DOWN\GO\NL-VS-L1.F.html#gene366) | GTPase binding | 1 (0.08%) | 6 (0.17%) | 0.928488 | 0.999471 |
| 367 | [GO:0036094](file:///E:\2018-7-3%E7%81%AB%E9%BE%99%E6%9E%9C%E8%BD%AC%E5%BD%95%E7%BB%84%E6%B5%8B%E5%BA%8F\%E5%AE%8C%E6%95%B4%E7%89%88%E6%95%B0%E6%8D%AE\GDR3855-Hylocereus_undulatus_Britt-12-RNAseq_result\4_Function\2_Group_Diff_Function\UP_DOWN\GO\NL-VS-L1.F.html#gene367) | small molecule binding | 243 (19.92%) | 732 (21.33%) | 0.938866 | 0.999471 |
| 368 | [GO:0016887](file:///E:\2018-7-3%E7%81%AB%E9%BE%99%E6%9E%9C%E8%BD%AC%E5%BD%95%E7%BB%84%E6%B5%8B%E5%BA%8F\%E5%AE%8C%E6%95%B4%E7%89%88%E6%95%B0%E6%8D%AE\GDR3855-Hylocereus_undulatus_Britt-12-RNAseq_result\4_Function\2_Group_Diff_Function\UP_DOWN\GO\NL-VS-L1.F.html#gene368) | ATPase activity | 32 (2.62%) | 110 (3.21%) | 0.939919 | 0.999471 |
| 369 | [GO:0097367](file:///E:\2018-7-3%E7%81%AB%E9%BE%99%E6%9E%9C%E8%BD%AC%E5%BD%95%E7%BB%84%E6%B5%8B%E5%BA%8F\%E5%AE%8C%E6%95%B4%E7%89%88%E6%95%B0%E6%8D%AE\GDR3855-Hylocereus_undulatus_Britt-12-RNAseq_result\4_Function\2_Group_Diff_Function\UP_DOWN\GO\NL-VS-L1.F.html#gene369) | carbohydrate derivative binding | 201 (16.48%) | 611 (17.8%) | 0.940734 | 0.999471 |
| 370 | [GO:0051536](file:///E:\2018-7-3%E7%81%AB%E9%BE%99%E6%9E%9C%E8%BD%AC%E5%BD%95%E7%BB%84%E6%B5%8B%E5%BA%8F\%E5%AE%8C%E6%95%B4%E7%89%88%E6%95%B0%E6%8D%AE\GDR3855-Hylocereus_undulatus_Britt-12-RNAseq_result\4_Function\2_Group_Diff_Function\UP_DOWN\GO\NL-VS-L1.F.html#gene370) | iron-sulfur cluster binding | 6 (0.49%) | 26 (0.76%) | 0.942642 | 0.999471 |
| 371 | [GO:0008237](file:///E:\2018-7-3%E7%81%AB%E9%BE%99%E6%9E%9C%E8%BD%AC%E5%BD%95%E7%BB%84%E6%B5%8B%E5%BA%8F\%E5%AE%8C%E6%95%B4%E7%89%88%E6%95%B0%E6%8D%AE\GDR3855-Hylocereus_undulatus_Britt-12-RNAseq_result\4_Function\2_Group_Diff_Function\UP_DOWN\GO\NL-VS-L1.F.html#gene371) | metallopeptidase activity | 2 (0.16%) | 11 (0.32%) | 0.943936 | 0.999471 |
| 372 | [GO:0001882](file:///E:\2018-7-3%E7%81%AB%E9%BE%99%E6%9E%9C%E8%BD%AC%E5%BD%95%E7%BB%84%E6%B5%8B%E5%BA%8F\%E5%AE%8C%E6%95%B4%E7%89%88%E6%95%B0%E6%8D%AE\GDR3855-Hylocereus_undulatus_Britt-12-RNAseq_result\4_Function\2_Group_Diff_Function\UP_DOWN\GO\NL-VS-L1.F.html#gene372) | nucleoside binding | 200 (16.39%) | 609 (17.74%) | 0.944111 | 0.999471 |
| 373 | [GO:0001883](file:///E:\2018-7-3%E7%81%AB%E9%BE%99%E6%9E%9C%E8%BD%AC%E5%BD%95%E7%BB%84%E6%B5%8B%E5%BA%8F\%E5%AE%8C%E6%95%B4%E7%89%88%E6%95%B0%E6%8D%AE\GDR3855-Hylocereus_undulatus_Britt-12-RNAseq_result\4_Function\2_Group_Diff_Function\UP_DOWN\GO\NL-VS-L1.F.html#gene373) | purine nucleoside binding | 199 (16.31%) | 607 (17.69%) | 0.947344 | 0.999471 |
| 374 | [GO:0032549](file:///E:\2018-7-3%E7%81%AB%E9%BE%99%E6%9E%9C%E8%BD%AC%E5%BD%95%E7%BB%84%E6%B5%8B%E5%BA%8F\%E5%AE%8C%E6%95%B4%E7%89%88%E6%95%B0%E6%8D%AE\GDR3855-Hylocereus_undulatus_Britt-12-RNAseq_result\4_Function\2_Group_Diff_Function\UP_DOWN\GO\NL-VS-L1.F.html#gene374) | ribonucleoside binding | 199 (16.31%) | 607 (17.69%) | 0.947344 | 0.999471 |
| 375 | [GO:0032550](file:///E:\2018-7-3%E7%81%AB%E9%BE%99%E6%9E%9C%E8%BD%AC%E5%BD%95%E7%BB%84%E6%B5%8B%E5%BA%8F\%E5%AE%8C%E6%95%B4%E7%89%88%E6%95%B0%E6%8D%AE\GDR3855-Hylocereus_undulatus_Britt-12-RNAseq_result\4_Function\2_Group_Diff_Function\UP_DOWN\GO\NL-VS-L1.F.html#gene375) | purine ribonucleoside binding | 199 (16.31%) | 607 (17.69%) | 0.947344 | 0.999471 |
| 376 | [GO:0042623](file:///E:\2018-7-3%E7%81%AB%E9%BE%99%E6%9E%9C%E8%BD%AC%E5%BD%95%E7%BB%84%E6%B5%8B%E5%BA%8F\%E5%AE%8C%E6%95%B4%E7%89%88%E6%95%B0%E6%8D%AE\GDR3855-Hylocereus_undulatus_Britt-12-RNAseq_result\4_Function\2_Group_Diff_Function\UP_DOWN\GO\NL-VS-L1.F.html#gene376) | ATPase activity, coupled | 31 (2.54%) | 108 (3.15%) | 0.948413 | 0.999471 |
| 377 | [GO:0030246](file:///E:\2018-7-3%E7%81%AB%E9%BE%99%E6%9E%9C%E8%BD%AC%E5%BD%95%E7%BB%84%E6%B5%8B%E5%BA%8F\%E5%AE%8C%E6%95%B4%E7%89%88%E6%95%B0%E6%8D%AE\GDR3855-Hylocereus_undulatus_Britt-12-RNAseq_result\4_Function\2_Group_Diff_Function\UP_DOWN\GO\NL-VS-L1.F.html#gene377) | carbohydrate binding | 1 (0.08%) | 7 (0.2%) | 0.953953 | 0.999471 |
| 378 | [GO:0050664](file:///E:\2018-7-3%E7%81%AB%E9%BE%99%E6%9E%9C%E8%BD%AC%E5%BD%95%E7%BB%84%E6%B5%8B%E5%BA%8F\%E5%AE%8C%E6%95%B4%E7%89%88%E6%95%B0%E6%8D%AE\GDR3855-Hylocereus_undulatus_Britt-12-RNAseq_result\4_Function\2_Group_Diff_Function\UP_DOWN\GO\NL-VS-L1.F.html#gene378) | oxidoreductase activity, acting on NAD(P)H, oxygen as acceptor | 1 (0.08%) | 7 (0.2%) | 0.953953 | 0.999471 |
| 379 | [GO:0042625](file:///E:\2018-7-3%E7%81%AB%E9%BE%99%E6%9E%9C%E8%BD%AC%E5%BD%95%E7%BB%84%E6%B5%8B%E5%BA%8F\%E5%AE%8C%E6%95%B4%E7%89%88%E6%95%B0%E6%8D%AE\GDR3855-Hylocereus_undulatus_Britt-12-RNAseq_result\4_Function\2_Group_Diff_Function\UP_DOWN\GO\NL-VS-L1.F.html#gene379) | ATPase coupled ion transmembrane transporter activity | 7 (0.57%) | 31 (0.9%) | 0.960135 | 0.999471 |
| 380 | [GO:0016817](file:///E:\2018-7-3%E7%81%AB%E9%BE%99%E6%9E%9C%E8%BD%AC%E5%BD%95%E7%BB%84%E6%B5%8B%E5%BA%8F\%E5%AE%8C%E6%95%B4%E7%89%88%E6%95%B0%E6%8D%AE\GDR3855-Hylocereus_undulatus_Britt-12-RNAseq_result\4_Function\2_Group_Diff_Function\UP_DOWN\GO\NL-VS-L1.F.html#gene380) | hydrolase activity, acting on acid anhydrides | 83 (6.8%) | 269 (7.84%) | 0.960253 | 0.999471 |
| 381 | [GO:0004518](file:///E:\2018-7-3%E7%81%AB%E9%BE%99%E6%9E%9C%E8%BD%AC%E5%BD%95%E7%BB%84%E6%B5%8B%E5%BA%8F\%E5%AE%8C%E6%95%B4%E7%89%88%E6%95%B0%E6%8D%AE\GDR3855-Hylocereus_undulatus_Britt-12-RNAseq_result\4_Function\2_Group_Diff_Function\UP_DOWN\GO\NL-VS-L1.F.html#gene381) | nuclease activity | 5 (0.41%) | 24 (0.7%) | 0.962966 | 0.999471 |
| 382 | [GO:0019829](file:///E:\2018-7-3%E7%81%AB%E9%BE%99%E6%9E%9C%E8%BD%AC%E5%BD%95%E7%BB%84%E6%B5%8B%E5%BA%8F\%E5%AE%8C%E6%95%B4%E7%89%88%E6%95%B0%E6%8D%AE\GDR3855-Hylocereus_undulatus_Britt-12-RNAseq_result\4_Function\2_Group_Diff_Function\UP_DOWN\GO\NL-VS-L1.F.html#gene382) | cation-transporting ATPase activity | 6 (0.49%) | 28 (0.82%) | 0.965953 | 0.999471 |
| 383 | [GO:0016462](file:///E:\2018-7-3%E7%81%AB%E9%BE%99%E6%9E%9C%E8%BD%AC%E5%BD%95%E7%BB%84%E6%B5%8B%E5%BA%8F\%E5%AE%8C%E6%95%B4%E7%89%88%E6%95%B0%E6%8D%AE\GDR3855-Hylocereus_undulatus_Britt-12-RNAseq_result\4_Function\2_Group_Diff_Function\UP_DOWN\GO\NL-VS-L1.F.html#gene383) | pyrophosphatase activity | 82 (6.72%) | 268 (7.81%) | 0.967430 | 0.999471 |
| 384 | [GO:0016818](file:///E:\2018-7-3%E7%81%AB%E9%BE%99%E6%9E%9C%E8%BD%AC%E5%BD%95%E7%BB%84%E6%B5%8B%E5%BA%8F\%E5%AE%8C%E6%95%B4%E7%89%88%E6%95%B0%E6%8D%AE\GDR3855-Hylocereus_undulatus_Britt-12-RNAseq_result\4_Function\2_Group_Diff_Function\UP_DOWN\GO\NL-VS-L1.F.html#gene384) | hydrolase activity, acting on acid anhydrides, in phosphorus-containing anhydrides | 82 (6.72%) | 268 (7.81%) | 0.967430 | 0.999471 |
| 385 | [GO:0008173](file:///E:\2018-7-3%E7%81%AB%E9%BE%99%E6%9E%9C%E8%BD%AC%E5%BD%95%E7%BB%84%E6%B5%8B%E5%BA%8F\%E5%AE%8C%E6%95%B4%E7%89%88%E6%95%B0%E6%8D%AE\GDR3855-Hylocereus_undulatus_Britt-12-RNAseq_result\4_Function\2_Group_Diff_Function\UP_DOWN\GO\NL-VS-L1.F.html#gene385) | RNA methyltransferase activity | 1 (0.08%) | 8 (0.23%) | 0.970355 | 0.999471 |
| 386 | [GO:0001871](file:///E:\2018-7-3%E7%81%AB%E9%BE%99%E6%9E%9C%E8%BD%AC%E5%BD%95%E7%BB%84%E6%B5%8B%E5%BA%8F\%E5%AE%8C%E6%95%B4%E7%89%88%E6%95%B0%E6%8D%AE\GDR3855-Hylocereus_undulatus_Britt-12-RNAseq_result\4_Function\2_Group_Diff_Function\UP_DOWN\GO\NL-VS-L1.F.html#gene386) | pattern binding | 1 (0.08%) | 9 (0.26%) | 0.980918 | 0.999471 |
| 387 | [GO:0008289](file:///E:\2018-7-3%E7%81%AB%E9%BE%99%E6%9E%9C%E8%BD%AC%E5%BD%95%E7%BB%84%E6%B5%8B%E5%BA%8F\%E5%AE%8C%E6%95%B4%E7%89%88%E6%95%B0%E6%8D%AE\GDR3855-Hylocereus_undulatus_Britt-12-RNAseq_result\4_Function\2_Group_Diff_Function\UP_DOWN\GO\NL-VS-L1.F.html#gene387) | lipid binding | 1 (0.08%) | 9 (0.26%) | 0.980918 | 0.999471 |
| 388 | [GO:0031406](file:///E:\2018-7-3%E7%81%AB%E9%BE%99%E6%9E%9C%E8%BD%AC%E5%BD%95%E7%BB%84%E6%B5%8B%E5%BA%8F\%E5%AE%8C%E6%95%B4%E7%89%88%E6%95%B0%E6%8D%AE\GDR3855-Hylocereus_undulatus_Britt-12-RNAseq_result\4_Function\2_Group_Diff_Function\UP_DOWN\GO\NL-VS-L1.F.html#gene388) | carboxylic acid binding | 2 (0.16%) | 15 (0.44%) | 0.987385 | 0.999471 |
| 389 | [GO:0043177](file:///E:\2018-7-3%E7%81%AB%E9%BE%99%E6%9E%9C%E8%BD%AC%E5%BD%95%E7%BB%84%E6%B5%8B%E5%BA%8F\%E5%AE%8C%E6%95%B4%E7%89%88%E6%95%B0%E6%8D%AE\GDR3855-Hylocereus_undulatus_Britt-12-RNAseq_result\4_Function\2_Group_Diff_Function\UP_DOWN\GO\NL-VS-L1.F.html#gene389) | organic acid binding | 2 (0.16%) | 15 (0.44%) | 0.987385 | 0.999471 |
| 390 | [GO:0004527](file:///E:\2018-7-3%E7%81%AB%E9%BE%99%E6%9E%9C%E8%BD%AC%E5%BD%95%E7%BB%84%E6%B5%8B%E5%BA%8F\%E5%AE%8C%E6%95%B4%E7%89%88%E6%95%B0%E6%8D%AE\GDR3855-Hylocereus_undulatus_Britt-12-RNAseq_result\4_Function\2_Group_Diff_Function\UP_DOWN\GO\NL-VS-L1.F.html#gene390) | exonuclease activity | 1 (0.08%) | 10 (0.29%) | 0.987719 | 0.999471 |
| 391 | [GO:0043169](file:///E:\2018-7-3%E7%81%AB%E9%BE%99%E6%9E%9C%E8%BD%AC%E5%BD%95%E7%BB%84%E6%B5%8B%E5%BA%8F\%E5%AE%8C%E6%95%B4%E7%89%88%E6%95%B0%E6%8D%AE\GDR3855-Hylocereus_undulatus_Britt-12-RNAseq_result\4_Function\2_Group_Diff_Function\UP_DOWN\GO\NL-VS-L1.F.html#gene391) | cation binding | 156 (12.79%) | 502 (14.63%) | 0.990171 | 0.999471 |
| 392 | [GO:0016655](file:///E:\2018-7-3%E7%81%AB%E9%BE%99%E6%9E%9C%E8%BD%AC%E5%BD%95%E7%BB%84%E6%B5%8B%E5%BA%8F\%E5%AE%8C%E6%95%B4%E7%89%88%E6%95%B0%E6%8D%AE\GDR3855-Hylocereus_undulatus_Britt-12-RNAseq_result\4_Function\2_Group_Diff_Function\UP_DOWN\GO\NL-VS-L1.F.html#gene392) | oxidoreductase activity, acting on NAD(P)H, quinone or similar compound as acceptor | 2 (0.16%) | 17 (0.5%) | 0.994160 | 0.999471 |
| 393 | [GO:0005488](file:///E:\2018-7-3%E7%81%AB%E9%BE%99%E6%9E%9C%E8%BD%AC%E5%BD%95%E7%BB%84%E6%B5%8B%E5%BA%8F\%E5%AE%8C%E6%95%B4%E7%89%88%E6%95%B0%E6%8D%AE\GDR3855-Hylocereus_undulatus_Britt-12-RNAseq_result\4_Function\2_Group_Diff_Function\UP_DOWN\GO\NL-VS-L1.F.html#gene393) | binding | 562 (46.07%) | 1688 (49.18%) | 0.997026 | 0.999471 |
| 394 | [GO:0046914](file:///E:\2018-7-3%E7%81%AB%E9%BE%99%E6%9E%9C%E8%BD%AC%E5%BD%95%E7%BB%84%E6%B5%8B%E5%BA%8F\%E5%AE%8C%E6%95%B4%E7%89%88%E6%95%B0%E6%8D%AE\GDR3855-Hylocereus_undulatus_Britt-12-RNAseq_result\4_Function\2_Group_Diff_Function\UP_DOWN\GO\NL-VS-L1.F.html#gene394) | transition metal ion binding | 46 (3.77%) | 175 (5.1%) | 0.997148 | 0.999471 |
| 395 | [GO:0043167](file:///E:\2018-7-3%E7%81%AB%E9%BE%99%E6%9E%9C%E8%BD%AC%E5%BD%95%E7%BB%84%E6%B5%8B%E5%BA%8F\%E5%AE%8C%E6%95%B4%E7%89%88%E6%95%B0%E6%8D%AE\GDR3855-Hylocereus_undulatus_Britt-12-RNAseq_result\4_Function\2_Group_Diff_Function\UP_DOWN\GO\NL-VS-L1.F.html#gene395) | ion binding | 174 (14.26%) | 573 (16.7%) | 0.998206 | 0.999471 |
| 396 | [GO:0003954](file:///E:\2018-7-3%E7%81%AB%E9%BE%99%E6%9E%9C%E8%BD%AC%E5%BD%95%E7%BB%84%E6%B5%8B%E5%BA%8F\%E5%AE%8C%E6%95%B4%E7%89%88%E6%95%B0%E6%8D%AE\GDR3855-Hylocereus_undulatus_Britt-12-RNAseq_result\4_Function\2_Group_Diff_Function\UP_DOWN\GO\NL-VS-L1.F.html#gene396) | NADH dehydrogenase activity | 1 (0.08%) | 16 (0.47%) | 0.999130 | 0.999471 |
| 397 | [GO:0050136](file:///E:\2018-7-3%E7%81%AB%E9%BE%99%E6%9E%9C%E8%BD%AC%E5%BD%95%E7%BB%84%E6%B5%8B%E5%BA%8F\%E5%AE%8C%E6%95%B4%E7%89%88%E6%95%B0%E6%8D%AE\GDR3855-Hylocereus_undulatus_Britt-12-RNAseq_result\4_Function\2_Group_Diff_Function\UP_DOWN\GO\NL-VS-L1.F.html#gene397) | NADH dehydrogenase (quinone) activity | 1 (0.08%) | 16 (0.47%) | 0.999130 | 0.999471 |
| 398 | [GO:0046872](file:///E:\2018-7-3%E7%81%AB%E9%BE%99%E6%9E%9C%E8%BD%AC%E5%BD%95%E7%BB%84%E6%B5%8B%E5%BA%8F\%E5%AE%8C%E6%95%B4%E7%89%88%E6%95%B0%E6%8D%AE\GDR3855-Hylocereus_undulatus_Britt-12-RNAseq_result\4_Function\2_Group_Diff_Function\UP_DOWN\GO\NL-VS-L1.F.html#gene398) | metal ion binding | 77 (6.31%) | 285 (8.3%) | 0.999454 | 0.999471 |
| 399 | [GO:0016651](file:///E:\2018-7-3%E7%81%AB%E9%BE%99%E6%9E%9C%E8%BD%AC%E5%BD%95%E7%BB%84%E6%B5%8B%E5%BA%8F\%E5%AE%8C%E6%95%B4%E7%89%88%E6%95%B0%E6%8D%AE\GDR3855-Hylocereus_undulatus_Britt-12-RNAseq_result\4_Function\2_Group_Diff_Function\UP_DOWN\GO\NL-VS-L1.F.html#gene399) | oxidoreductase activity, acting on NAD(P)H | 4 (0.33%) | 33 (0.96%) | 0.999471 | 0.999471 |
