# Supplementary material for: Transcriptomic analysis of flower induction for long-day pitaya by supplementary lighting in short-day winter season
Source: BMC Genomics. 2020 Apr 29;21:329. doi: 10.1186/s12864-020-6726-6 (PMC7191803; doi:10.1186/s12864-020-6726-6)
Supplement: Supplementary file 7 — Additional file 7: Supplemental S7. NL-VS-L1 GO Enrichment (Cellular Component). [file 12864_2020_6726_MOESM7_ESM.docx]

Supplemental S7 NL-VS-L1 GO Enrichment (Cellular Component)

|  | **GO ID** | **Description** | **GeneRatio (682)** | **BgRatio (1849)** | **pvalue** | **p.adjust** |
| --- | --- | --- | --- | --- | --- | --- |
| 1 | [GO:0044436](file:///E:\2018-7-3%E7%81%AB%E9%BE%99%E6%9E%9C%E8%BD%AC%E5%BD%95%E7%BB%84%E6%B5%8B%E5%BA%8F\%E5%AE%8C%E6%95%B4%E7%89%88%E6%95%B0%E6%8D%AE\GDR3855-Hylocereus_undulatus_Britt-12-RNAseq_result\4_Function\2_Group_Diff_Function\UP_DOWN\GO\NL-VS-L1.C.html#gene1) | thylakoid part | 29 (4.25%) | 43 (2.33%) | 0.000038 | **0.004756** |
| 2 | [GO:0009521](file:///E:\2018-7-3%E7%81%AB%E9%BE%99%E6%9E%9C%E8%BD%AC%E5%BD%95%E7%BB%84%E6%B5%8B%E5%BA%8F\%E5%AE%8C%E6%95%B4%E7%89%88%E6%95%B0%E6%8D%AE\GDR3855-Hylocereus_undulatus_Britt-12-RNAseq_result\4_Function\2_Group_Diff_Function\UP_DOWN\GO\NL-VS-L1.C.html#gene2) | photosystem | 24 (3.52%) | 34 (1.84%) | 0.000059 | **0.004756** |
| 3 | [GO:0098796](file:///E:\2018-7-3%E7%81%AB%E9%BE%99%E6%9E%9C%E8%BD%AC%E5%BD%95%E7%BB%84%E6%B5%8B%E5%BA%8F\%E5%AE%8C%E6%95%B4%E7%89%88%E6%95%B0%E6%8D%AE\GDR3855-Hylocereus_undulatus_Britt-12-RNAseq_result\4_Function\2_Group_Diff_Function\UP_DOWN\GO\NL-VS-L1.C.html#gene3) | membrane protein complex | 39 (5.72%) | 65 (3.52%) | 0.000098 | **0.005174** |
| 4 | [GO:0034357](file:///E:\2018-7-3%E7%81%AB%E9%BE%99%E6%9E%9C%E8%BD%AC%E5%BD%95%E7%BB%84%E6%B5%8B%E5%BA%8F\%E5%AE%8C%E6%95%B4%E7%89%88%E6%95%B0%E6%8D%AE\GDR3855-Hylocereus_undulatus_Britt-12-RNAseq_result\4_Function\2_Group_Diff_Function\UP_DOWN\GO\NL-VS-L1.C.html#gene4) | photosynthetic membrane | 25 (3.67%) | 37 (2%) | 0.000128 | **0.005174** |
| 5 | [GO:0009579](file:///E:\2018-7-3%E7%81%AB%E9%BE%99%E6%9E%9C%E8%BD%AC%E5%BD%95%E7%BB%84%E6%B5%8B%E5%BA%8F\%E5%AE%8C%E6%95%B4%E7%89%88%E6%95%B0%E6%8D%AE\GDR3855-Hylocereus_undulatus_Britt-12-RNAseq_result\4_Function\2_Group_Diff_Function\UP_DOWN\GO\NL-VS-L1.C.html#gene5) | thylakoid | 52 (7.62%) | 96 (5.19%) | 0.000298 | **0.009647** |
| 6 | [GO:0043234](file:///E:\2018-7-3%E7%81%AB%E9%BE%99%E6%9E%9C%E8%BD%AC%E5%BD%95%E7%BB%84%E6%B5%8B%E5%BA%8F\%E5%AE%8C%E6%95%B4%E7%89%88%E6%95%B0%E6%8D%AE\GDR3855-Hylocereus_undulatus_Britt-12-RNAseq_result\4_Function\2_Group_Diff_Function\UP_DOWN\GO\NL-VS-L1.C.html#gene6) | protein complex | 79 (11.58%) | 162 (8.76%) | 0.000806 | **0.021773** |
| 7 | [GO:0044425](file:///E:\2018-7-3%E7%81%AB%E9%BE%99%E6%9E%9C%E8%BD%AC%E5%BD%95%E7%BB%84%E6%B5%8B%E5%BA%8F\%E5%AE%8C%E6%95%B4%E7%89%88%E6%95%B0%E6%8D%AE\GDR3855-Hylocereus_undulatus_Britt-12-RNAseq_result\4_Function\2_Group_Diff_Function\UP_DOWN\GO\NL-VS-L1.C.html#gene7) | membrane part | 213 (31.23%) | 503 (27.2%) | 0.001823 | **0.042187** |
| 8 | [GO:0031976](file:///E:\2018-7-3%E7%81%AB%E9%BE%99%E6%9E%9C%E8%BD%AC%E5%BD%95%E7%BB%84%E6%B5%8B%E5%BA%8F\%E5%AE%8C%E6%95%B4%E7%89%88%E6%95%B0%E6%8D%AE\GDR3855-Hylocereus_undulatus_Britt-12-RNAseq_result\4_Function\2_Group_Diff_Function\UP_DOWN\GO\NL-VS-L1.C.html#gene8) | plastid thylakoid | 35 (5.13%) | 65 (3.52%) | 0.003381 | 0.066091 |
| 9 | [GO:0005856](file:///E:\2018-7-3%E7%81%AB%E9%BE%99%E6%9E%9C%E8%BD%AC%E5%BD%95%E7%BB%84%E6%B5%8B%E5%BA%8F\%E5%AE%8C%E6%95%B4%E7%89%88%E6%95%B0%E6%8D%AE\GDR3855-Hylocereus_undulatus_Britt-12-RNAseq_result\4_Function\2_Group_Diff_Function\UP_DOWN\GO\NL-VS-L1.C.html#gene9) | cytoskeleton | 34 (4.99%) | 63 (3.41%) | 0.003672 | 0.066091 |
| 10 | [GO:0031984](file:///E:\2018-7-3%E7%81%AB%E9%BE%99%E6%9E%9C%E8%BD%AC%E5%BD%95%E7%BB%84%E6%B5%8B%E5%BA%8F\%E5%AE%8C%E6%95%B4%E7%89%88%E6%95%B0%E6%8D%AE\GDR3855-Hylocereus_undulatus_Britt-12-RNAseq_result\4_Function\2_Group_Diff_Function\UP_DOWN\GO\NL-VS-L1.C.html#gene10) | organelle subcompartment | 35 (5.13%) | 66 (3.57%) | 0.004714 | 0.076369 |
| 11 | [GO:0015630](file:///E:\2018-7-3%E7%81%AB%E9%BE%99%E6%9E%9C%E8%BD%AC%E5%BD%95%E7%BB%84%E6%B5%8B%E5%BA%8F\%E5%AE%8C%E6%95%B4%E7%89%88%E6%95%B0%E6%8D%AE\GDR3855-Hylocereus_undulatus_Britt-12-RNAseq_result\4_Function\2_Group_Diff_Function\UP_DOWN\GO\NL-VS-L1.C.html#gene11) | microtubule cytoskeleton | 29 (4.25%) | 53 (2.87%) | 0.005512 | 0.081183 |
| 12 | [GO:0032991](file:///E:\2018-7-3%E7%81%AB%E9%BE%99%E6%9E%9C%E8%BD%AC%E5%BD%95%E7%BB%84%E6%B5%8B%E5%BA%8F\%E5%AE%8C%E6%95%B4%E7%89%88%E6%95%B0%E6%8D%AE\GDR3855-Hylocereus_undulatus_Britt-12-RNAseq_result\4_Function\2_Group_Diff_Function\UP_DOWN\GO\NL-VS-L1.C.html#gene12) | macromolecular complex | 174 (25.51%) | 417 (22.55%) | 0.011886 | 0.160464 |
| 13 | [GO:0016020](file:///E:\2018-7-3%E7%81%AB%E9%BE%99%E6%9E%9C%E8%BD%AC%E5%BD%95%E7%BB%84%E6%B5%8B%E5%BA%8F\%E5%AE%8C%E6%95%B4%E7%89%88%E6%95%B0%E6%8D%AE\GDR3855-Hylocereus_undulatus_Britt-12-RNAseq_result\4_Function\2_Group_Diff_Function\UP_DOWN\GO\NL-VS-L1.C.html#gene13) | membrane | 305 (44.72%) | 770 (41.64%) | 0.022679 | 0.273825 |
| 14 | [GO:0043228](file:///E:\2018-7-3%E7%81%AB%E9%BE%99%E6%9E%9C%E8%BD%AC%E5%BD%95%E7%BB%84%E6%B5%8B%E5%BA%8F\%E5%AE%8C%E6%95%B4%E7%89%88%E6%95%B0%E6%8D%AE\GDR3855-Hylocereus_undulatus_Britt-12-RNAseq_result\4_Function\2_Group_Diff_Function\UP_DOWN\GO\NL-VS-L1.C.html#gene14) | non-membrane-bounded organelle | 94 (13.78%) | 218 (11.79%) | 0.025919 | 0.273825 |
| 15 | [GO:0043232](file:///E:\2018-7-3%E7%81%AB%E9%BE%99%E6%9E%9C%E8%BD%AC%E5%BD%95%E7%BB%84%E6%B5%8B%E5%BA%8F\%E5%AE%8C%E6%95%B4%E7%89%88%E6%95%B0%E6%8D%AE\GDR3855-Hylocereus_undulatus_Britt-12-RNAseq_result\4_Function\2_Group_Diff_Function\UP_DOWN\GO\NL-VS-L1.C.html#gene15) | intracellular non-membrane-bounded organelle | 94 (13.78%) | 218 (11.79%) | 0.025919 | 0.273825 |
| 16 | [GO:0015935](file:///E:\2018-7-3%E7%81%AB%E9%BE%99%E6%9E%9C%E8%BD%AC%E5%BD%95%E7%BB%84%E6%B5%8B%E5%BA%8F\%E5%AE%8C%E6%95%B4%E7%89%88%E6%95%B0%E6%8D%AE\GDR3855-Hylocereus_undulatus_Britt-12-RNAseq_result\4_Function\2_Group_Diff_Function\UP_DOWN\GO\NL-VS-L1.C.html#gene16) | small ribosomal subunit | 17 (2.49%) | 31 (1.68%) | 0.030421 | 0.273825 |
| 17 | [GO:0044430](file:///E:\2018-7-3%E7%81%AB%E9%BE%99%E6%9E%9C%E8%BD%AC%E5%BD%95%E7%BB%84%E6%B5%8B%E5%BA%8F\%E5%AE%8C%E6%95%B4%E7%89%88%E6%95%B0%E6%8D%AE\GDR3855-Hylocereus_undulatus_Britt-12-RNAseq_result\4_Function\2_Group_Diff_Function\UP_DOWN\GO\NL-VS-L1.C.html#gene17) | cytoskeletal part | 17 (2.49%) | 31 (1.68%) | 0.030421 | 0.273825 |
| 18 | [GO:0031224](file:///E:\2018-7-3%E7%81%AB%E9%BE%99%E6%9E%9C%E8%BD%AC%E5%BD%95%E7%BB%84%E6%B5%8B%E5%BA%8F\%E5%AE%8C%E6%95%B4%E7%89%88%E6%95%B0%E6%8D%AE\GDR3855-Hylocereus_undulatus_Britt-12-RNAseq_result\4_Function\2_Group_Diff_Function\UP_DOWN\GO\NL-VS-L1.C.html#gene18) | intrinsic component of membrane | 171 (25.07%) | 418 (22.61%) | 0.030432 | 0.273825 |
| 19 | [GO:0009507](file:///E:\2018-7-3%E7%81%AB%E9%BE%99%E6%9E%9C%E8%BD%AC%E5%BD%95%E7%BB%84%E6%B5%8B%E5%BA%8F\%E5%AE%8C%E6%95%B4%E7%89%88%E6%95%B0%E6%8D%AE\GDR3855-Hylocereus_undulatus_Britt-12-RNAseq_result\4_Function\2_Group_Diff_Function\UP_DOWN\GO\NL-VS-L1.C.html#gene19) | chloroplast | 29 (4.25%) | 59 (3.19%) | 0.033806 | 0.273825 |
| 20 | [GO:0044434](file:///E:\2018-7-3%E7%81%AB%E9%BE%99%E6%9E%9C%E8%BD%AC%E5%BD%95%E7%BB%84%E6%B5%8B%E5%BA%8F\%E5%AE%8C%E6%95%B4%E7%89%88%E6%95%B0%E6%8D%AE\GDR3855-Hylocereus_undulatus_Britt-12-RNAseq_result\4_Function\2_Group_Diff_Function\UP_DOWN\GO\NL-VS-L1.C.html#gene20) | chloroplast part | 29 (4.25%) | 59 (3.19%) | 0.033806 | 0.273825 |
| 21 | [GO:0005911](file:///E:\2018-7-3%E7%81%AB%E9%BE%99%E6%9E%9C%E8%BD%AC%E5%BD%95%E7%BB%84%E6%B5%8B%E5%BA%8F\%E5%AE%8C%E6%95%B4%E7%89%88%E6%95%B0%E6%8D%AE\GDR3855-Hylocereus_undulatus_Britt-12-RNAseq_result\4_Function\2_Group_Diff_Function\UP_DOWN\GO\NL-VS-L1.C.html#gene21) | cell-cell junction | 45 (6.6%) | 98 (5.3%) | 0.037377 | 0.288337 |
| 22 | [GO:0030054](file:///E:\2018-7-3%E7%81%AB%E9%BE%99%E6%9E%9C%E8%BD%AC%E5%BD%95%E7%BB%84%E6%B5%8B%E5%BA%8F\%E5%AE%8C%E6%95%B4%E7%89%88%E6%95%B0%E6%8D%AE\GDR3855-Hylocereus_undulatus_Britt-12-RNAseq_result\4_Function\2_Group_Diff_Function\UP_DOWN\GO\NL-VS-L1.C.html#gene22) | cell junction | 45 (6.6%) | 99 (5.35%) | 0.044903 | 0.330646 |
| 23 | [GO:0030312](file:///E:\2018-7-3%E7%81%AB%E9%BE%99%E6%9E%9C%E8%BD%AC%E5%BD%95%E7%BB%84%E6%B5%8B%E5%BA%8F\%E5%AE%8C%E6%95%B4%E7%89%88%E6%95%B0%E6%8D%AE\GDR3855-Hylocereus_undulatus_Britt-12-RNAseq_result\4_Function\2_Group_Diff_Function\UP_DOWN\GO\NL-VS-L1.C.html#gene23) | external encapsulating structure | 33 (4.84%) | 70 (3.79%) | 0.047218 | 0.332578 |
| 24 | [GO:0044427](file:///E:\2018-7-3%E7%81%AB%E9%BE%99%E6%9E%9C%E8%BD%AC%E5%BD%95%E7%BB%84%E6%B5%8B%E5%BA%8F\%E5%AE%8C%E6%95%B4%E7%89%88%E6%95%B0%E6%8D%AE\GDR3855-Hylocereus_undulatus_Britt-12-RNAseq_result\4_Function\2_Group_Diff_Function\UP_DOWN\GO\NL-VS-L1.C.html#gene24) | chromosomal part | 3 (0.44%) | 3 (0.16%) | 0.050042 | 0.337783 |
| 25 | [GO:0009534](file:///E:\2018-7-3%E7%81%AB%E9%BE%99%E6%9E%9C%E8%BD%AC%E5%BD%95%E7%BB%84%E6%B5%8B%E5%BA%8F\%E5%AE%8C%E6%95%B4%E7%89%88%E6%95%B0%E6%8D%AE\GDR3855-Hylocereus_undulatus_Britt-12-RNAseq_result\4_Function\2_Group_Diff_Function\UP_DOWN\GO\NL-VS-L1.C.html#gene25) | chloroplast thylakoid | 22 (3.23%) | 45 (2.43%) | 0.064245 | 0.404855 |
| 26 | [GO:0009522](file:///E:\2018-7-3%E7%81%AB%E9%BE%99%E6%9E%9C%E8%BD%AC%E5%BD%95%E7%BB%84%E6%B5%8B%E5%BA%8F\%E5%AE%8C%E6%95%B4%E7%89%88%E6%95%B0%E6%8D%AE\GDR3855-Hylocereus_undulatus_Britt-12-RNAseq_result\4_Function\2_Group_Diff_Function\UP_DOWN\GO\NL-VS-L1.C.html#gene26) | photosystem I | 4 (0.59%) | 5 (0.27%) | 0.064977 | 0.404855 |
| 27 | [GO:0071944](file:///E:\2018-7-3%E7%81%AB%E9%BE%99%E6%9E%9C%E8%BD%AC%E5%BD%95%E7%BB%84%E6%B5%8B%E5%BA%8F\%E5%AE%8C%E6%95%B4%E7%89%88%E6%95%B0%E6%8D%AE\GDR3855-Hylocereus_undulatus_Britt-12-RNAseq_result\4_Function\2_Group_Diff_Function\UP_DOWN\GO\NL-VS-L1.C.html#gene27) | cell periphery | 35 (5.13%) | 78 (4.22%) | 0.085818 | 0.514906 |
| 28 | [GO:0005875](file:///E:\2018-7-3%E7%81%AB%E9%BE%99%E6%9E%9C%E8%BD%AC%E5%BD%95%E7%BB%84%E6%B5%8B%E5%BA%8F\%E5%AE%8C%E6%95%B4%E7%89%88%E6%95%B0%E6%8D%AE\GDR3855-Hylocereus_undulatus_Britt-12-RNAseq_result\4_Function\2_Group_Diff_Function\UP_DOWN\GO\NL-VS-L1.C.html#gene28) | microtubule associated complex | 12 (1.76%) | 23 (1.24%) | 0.096358 | 0.550488 |
| 29 | [GO:0005618](file:///E:\2018-7-3%E7%81%AB%E9%BE%99%E6%9E%9C%E8%BD%AC%E5%BD%95%E7%BB%84%E6%B5%8B%E5%BA%8F\%E5%AE%8C%E6%95%B4%E7%89%88%E6%95%B0%E6%8D%AE\GDR3855-Hylocereus_undulatus_Britt-12-RNAseq_result\4_Function\2_Group_Diff_Function\UP_DOWN\GO\NL-VS-L1.C.html#gene29) | cell wall | 7 (1.03%) | 12 (0.65%) | 0.108202 | 0.550488 |
| 30 | [GO:0030117](file:///E:\2018-7-3%E7%81%AB%E9%BE%99%E6%9E%9C%E8%BD%AC%E5%BD%95%E7%BB%84%E6%B5%8B%E5%BA%8F\%E5%AE%8C%E6%95%B4%E7%89%88%E6%95%B0%E6%8D%AE\GDR3855-Hylocereus_undulatus_Britt-12-RNAseq_result\4_Function\2_Group_Diff_Function\UP_DOWN\GO\NL-VS-L1.C.html#gene30) | membrane coat | 5 (0.73%) | 8 (0.43%) | 0.128742 | 0.550488 |
| 31 | [GO:0048475](file:///E:\2018-7-3%E7%81%AB%E9%BE%99%E6%9E%9C%E8%BD%AC%E5%BD%95%E7%BB%84%E6%B5%8B%E5%BA%8F\%E5%AE%8C%E6%95%B4%E7%89%88%E6%95%B0%E6%8D%AE\GDR3855-Hylocereus_undulatus_Britt-12-RNAseq_result\4_Function\2_Group_Diff_Function\UP_DOWN\GO\NL-VS-L1.C.html#gene31) | coated membrane | 5 (0.73%) | 8 (0.43%) | 0.128742 | 0.550488 |
| 32 | [GO:0000785](file:///E:\2018-7-3%E7%81%AB%E9%BE%99%E6%9E%9C%E8%BD%AC%E5%BD%95%E7%BB%84%E6%B5%8B%E5%BA%8F\%E5%AE%8C%E6%95%B4%E7%89%88%E6%95%B0%E6%8D%AE\GDR3855-Hylocereus_undulatus_Britt-12-RNAseq_result\4_Function\2_Group_Diff_Function\UP_DOWN\GO\NL-VS-L1.C.html#gene32) | chromatin | 2 (0.29%) | 2 (0.11%) | 0.135923 | 0.550488 |
| 33 | [GO:0016323](file:///E:\2018-7-3%E7%81%AB%E9%BE%99%E6%9E%9C%E8%BD%AC%E5%BD%95%E7%BB%84%E6%B5%8B%E5%BA%8F\%E5%AE%8C%E6%95%B4%E7%89%88%E6%95%B0%E6%8D%AE\GDR3855-Hylocereus_undulatus_Britt-12-RNAseq_result\4_Function\2_Group_Diff_Function\UP_DOWN\GO\NL-VS-L1.C.html#gene33) | basolateral plasma membrane | 2 (0.29%) | 2 (0.11%) | 0.135923 | 0.550488 |
| 34 | [GO:0030120](file:///E:\2018-7-3%E7%81%AB%E9%BE%99%E6%9E%9C%E8%BD%AC%E5%BD%95%E7%BB%84%E6%B5%8B%E5%BA%8F\%E5%AE%8C%E6%95%B4%E7%89%88%E6%95%B0%E6%8D%AE\GDR3855-Hylocereus_undulatus_Britt-12-RNAseq_result\4_Function\2_Group_Diff_Function\UP_DOWN\GO\NL-VS-L1.C.html#gene34) | vesicle coat | 2 (0.29%) | 2 (0.11%) | 0.135923 | 0.550488 |
| 35 | [GO:0031012](file:///E:\2018-7-3%E7%81%AB%E9%BE%99%E6%9E%9C%E8%BD%AC%E5%BD%95%E7%BB%84%E6%B5%8B%E5%BA%8F\%E5%AE%8C%E6%95%B4%E7%89%88%E6%95%B0%E6%8D%AE\GDR3855-Hylocereus_undulatus_Britt-12-RNAseq_result\4_Function\2_Group_Diff_Function\UP_DOWN\GO\NL-VS-L1.C.html#gene35) | extracellular matrix | 2 (0.29%) | 2 (0.11%) | 0.135923 | 0.550488 |
| 36 | [GO:0043667](file:///E:\2018-7-3%E7%81%AB%E9%BE%99%E6%9E%9C%E8%BD%AC%E5%BD%95%E7%BB%84%E6%B5%8B%E5%BA%8F\%E5%AE%8C%E6%95%B4%E7%89%88%E6%95%B0%E6%8D%AE\GDR3855-Hylocereus_undulatus_Britt-12-RNAseq_result\4_Function\2_Group_Diff_Function\UP_DOWN\GO\NL-VS-L1.C.html#gene36) | pollen wall | 2 (0.29%) | 2 (0.11%) | 0.135923 | 0.550488 |
| 37 | [GO:0043668](file:///E:\2018-7-3%E7%81%AB%E9%BE%99%E6%9E%9C%E8%BD%AC%E5%BD%95%E7%BB%84%E6%B5%8B%E5%BA%8F\%E5%AE%8C%E6%95%B4%E7%89%88%E6%95%B0%E6%8D%AE\GDR3855-Hylocereus_undulatus_Britt-12-RNAseq_result\4_Function\2_Group_Diff_Function\UP_DOWN\GO\NL-VS-L1.C.html#gene37) | exine | 2 (0.29%) | 2 (0.11%) | 0.135923 | 0.550488 |
| 38 | [GO:0043673](file:///E:\2018-7-3%E7%81%AB%E9%BE%99%E6%9E%9C%E8%BD%AC%E5%BD%95%E7%BB%84%E6%B5%8B%E5%BA%8F\%E5%AE%8C%E6%95%B4%E7%89%88%E6%95%B0%E6%8D%AE\GDR3855-Hylocereus_undulatus_Britt-12-RNAseq_result\4_Function\2_Group_Diff_Function\UP_DOWN\GO\NL-VS-L1.C.html#gene38) | sexine | 2 (0.29%) | 2 (0.11%) | 0.135923 | 0.550488 |
| 39 | [GO:0044420](file:///E:\2018-7-3%E7%81%AB%E9%BE%99%E6%9E%9C%E8%BD%AC%E5%BD%95%E7%BB%84%E6%B5%8B%E5%BA%8F\%E5%AE%8C%E6%95%B4%E7%89%88%E6%95%B0%E6%8D%AE\GDR3855-Hylocereus_undulatus_Britt-12-RNAseq_result\4_Function\2_Group_Diff_Function\UP_DOWN\GO\NL-VS-L1.C.html#gene39) | extracellular matrix component | 2 (0.29%) | 2 (0.11%) | 0.135923 | 0.550488 |
| 40 | [GO:0098590](file:///E:\2018-7-3%E7%81%AB%E9%BE%99%E6%9E%9C%E8%BD%AC%E5%BD%95%E7%BB%84%E6%B5%8B%E5%BA%8F\%E5%AE%8C%E6%95%B4%E7%89%88%E6%95%B0%E6%8D%AE\GDR3855-Hylocereus_undulatus_Britt-12-RNAseq_result\4_Function\2_Group_Diff_Function\UP_DOWN\GO\NL-VS-L1.C.html#gene40) | plasma membrane region | 2 (0.29%) | 2 (0.11%) | 0.135923 | 0.550488 |
| 41 | [GO:0030964](file:///E:\2018-7-3%E7%81%AB%E9%BE%99%E6%9E%9C%E8%BD%AC%E5%BD%95%E7%BB%84%E6%B5%8B%E5%BA%8F\%E5%AE%8C%E6%95%B4%E7%89%88%E6%95%B0%E6%8D%AE\GDR3855-Hylocereus_undulatus_Britt-12-RNAseq_result\4_Function\2_Group_Diff_Function\UP_DOWN\GO\NL-VS-L1.C.html#gene41) | NADH dehydrogenase complex | 3 (0.44%) | 4 (0.22%) | 0.144948 | 0.559086 |
| 42 | [GO:1990204](file:///E:\2018-7-3%E7%81%AB%E9%BE%99%E6%9E%9C%E8%BD%AC%E5%BD%95%E7%BB%84%E6%B5%8B%E5%BA%8F\%E5%AE%8C%E6%95%B4%E7%89%88%E6%95%B0%E6%8D%AE\GDR3855-Hylocereus_undulatus_Britt-12-RNAseq_result\4_Function\2_Group_Diff_Function\UP_DOWN\GO\NL-VS-L1.C.html#gene42) | oxidoreductase complex | 3 (0.44%) | 4 (0.22%) | 0.144948 | 0.559086 |
| 43 | [GO:0009523](file:///E:\2018-7-3%E7%81%AB%E9%BE%99%E6%9E%9C%E8%BD%AC%E5%BD%95%E7%BB%84%E6%B5%8B%E5%BA%8F\%E5%AE%8C%E6%95%B4%E7%89%88%E6%95%B0%E6%8D%AE\GDR3855-Hylocereus_undulatus_Britt-12-RNAseq_result\4_Function\2_Group_Diff_Function\UP_DOWN\GO\NL-VS-L1.C.html#gene43) | photosystem II | 7 (1.03%) | 13 (0.7%) | 0.162372 | 0.611728 |
| 44 | [GO:0015629](file:///E:\2018-7-3%E7%81%AB%E9%BE%99%E6%9E%9C%E8%BD%AC%E5%BD%95%E7%BB%84%E6%B5%8B%E5%BA%8F\%E5%AE%8C%E6%95%B4%E7%89%88%E6%95%B0%E6%8D%AE\GDR3855-Hylocereus_undulatus_Britt-12-RNAseq_result\4_Function\2_Group_Diff_Function\UP_DOWN\GO\NL-VS-L1.C.html#gene44) | actin cytoskeleton | 5 (0.73%) | 9 (0.49%) | 0.204551 | 0.753121 |
| 45 | [GO:0000502](file:///E:\2018-7-3%E7%81%AB%E9%BE%99%E6%9E%9C%E8%BD%AC%E5%BD%95%E7%BB%84%E6%B5%8B%E5%BA%8F\%E5%AE%8C%E6%95%B4%E7%89%88%E6%95%B0%E6%8D%AE\GDR3855-Hylocereus_undulatus_Britt-12-RNAseq_result\4_Function\2_Group_Diff_Function\UP_DOWN\GO\NL-VS-L1.C.html#gene45) | proteasome complex | 4 (0.59%) | 7 (0.38%) | 0.231666 | 0.833998 |
| 46 | [GO:0019012](file:///E:\2018-7-3%E7%81%AB%E9%BE%99%E6%9E%9C%E8%BD%AC%E5%BD%95%E7%BB%84%E6%B5%8B%E5%BA%8F\%E5%AE%8C%E6%95%B4%E7%89%88%E6%95%B0%E6%8D%AE\GDR3855-Hylocereus_undulatus_Britt-12-RNAseq_result\4_Function\2_Group_Diff_Function\UP_DOWN\GO\NL-VS-L1.C.html#gene46) | virion | 8 (1.17%) | 17 (0.92%) | 0.263454 | 0.894057 |
| 47 | [GO:0044423](file:///E:\2018-7-3%E7%81%AB%E9%BE%99%E6%9E%9C%E8%BD%AC%E5%BD%95%E7%BB%84%E6%B5%8B%E5%BA%8F\%E5%AE%8C%E6%95%B4%E7%89%88%E6%95%B0%E6%8D%AE\GDR3855-Hylocereus_undulatus_Britt-12-RNAseq_result\4_Function\2_Group_Diff_Function\UP_DOWN\GO\NL-VS-L1.C.html#gene47) | virion part | 8 (1.17%) | 17 (0.92%) | 0.263454 | 0.894057 |
| 48 | [GO:0033176](file:///E:\2018-7-3%E7%81%AB%E9%BE%99%E6%9E%9C%E8%BD%AC%E5%BD%95%E7%BB%84%E6%B5%8B%E5%BA%8F\%E5%AE%8C%E6%95%B4%E7%89%88%E6%95%B0%E6%8D%AE\GDR3855-Hylocereus_undulatus_Britt-12-RNAseq_result\4_Function\2_Group_Diff_Function\UP_DOWN\GO\NL-VS-L1.C.html#gene48) | proton-transporting V-type ATPase complex | 3 (0.44%) | 5 (0.27%) | 0.264906 | 0.894057 |
| 49 | [GO:0044391](file:///E:\2018-7-3%E7%81%AB%E9%BE%99%E6%9E%9C%E8%BD%AC%E5%BD%95%E7%BB%84%E6%B5%8B%E5%BA%8F\%E5%AE%8C%E6%95%B4%E7%89%88%E6%95%B0%E6%8D%AE\GDR3855-Hylocereus_undulatus_Britt-12-RNAseq_result\4_Function\2_Group_Diff_Function\UP_DOWN\GO\NL-VS-L1.C.html#gene49) | ribosomal subunit | 34 (4.99%) | 84 (4.54%) | 0.278172 | 0.919671 |
| 50 | [GO:0098589](file:///E:\2018-7-3%E7%81%AB%E9%BE%99%E6%9E%9C%E8%BD%AC%E5%BD%95%E7%BB%84%E6%B5%8B%E5%BA%8F\%E5%AE%8C%E6%95%B4%E7%89%88%E6%95%B0%E6%8D%AE\GDR3855-Hylocereus_undulatus_Britt-12-RNAseq_result\4_Function\2_Group_Diff_Function\UP_DOWN\GO\NL-VS-L1.C.html#gene50) | membrane region | 2 (0.29%) | 3 (0.16%) | 0.307685 | 0.996898 |
| 51 | [GO:0005694](file:///E:\2018-7-3%E7%81%AB%E9%BE%99%E6%9E%9C%E8%BD%AC%E5%BD%95%E7%BB%84%E6%B5%8B%E5%BA%8F\%E5%AE%8C%E6%95%B4%E7%89%88%E6%95%B0%E6%8D%AE\GDR3855-Hylocereus_undulatus_Britt-12-RNAseq_result\4_Function\2_Group_Diff_Function\UP_DOWN\GO\NL-VS-L1.C.html#gene51) | chromosome | 4 (0.59%) | 8 (0.43%) | 0.334590 | 0.999621 |
| 52 | [GO:0009570](file:///E:\2018-7-3%E7%81%AB%E9%BE%99%E6%9E%9C%E8%BD%AC%E5%BD%95%E7%BB%84%E6%B5%8B%E5%BA%8F\%E5%AE%8C%E6%95%B4%E7%89%88%E6%95%B0%E6%8D%AE\GDR3855-Hylocereus_undulatus_Britt-12-RNAseq_result\4_Function\2_Group_Diff_Function\UP_DOWN\GO\NL-VS-L1.C.html#gene52) | chloroplast stroma | 4 (0.59%) | 8 (0.43%) | 0.334590 | 0.999621 |
| 53 | [GO:0019028](file:///E:\2018-7-3%E7%81%AB%E9%BE%99%E6%9E%9C%E8%BD%AC%E5%BD%95%E7%BB%84%E6%B5%8B%E5%BA%8F\%E5%AE%8C%E6%95%B4%E7%89%88%E6%95%B0%E6%8D%AE\GDR3855-Hylocereus_undulatus_Britt-12-RNAseq_result\4_Function\2_Group_Diff_Function\UP_DOWN\GO\NL-VS-L1.C.html#gene53) | viral capsid | 6 (0.88%) | 13 (0.7%) | 0.335153 | 0.999621 |
| 54 | [GO:0000808](file:///E:\2018-7-3%E7%81%AB%E9%BE%99%E6%9E%9C%E8%BD%AC%E5%BD%95%E7%BB%84%E6%B5%8B%E5%BA%8F\%E5%AE%8C%E6%95%B4%E7%89%88%E6%95%B0%E6%8D%AE\GDR3855-Hylocereus_undulatus_Britt-12-RNAseq_result\4_Function\2_Group_Diff_Function\UP_DOWN\GO\NL-VS-L1.C.html#gene54) | origin recognition complex | 1 (0.15%) | 1 (0.05%) | 0.368848 | 0.999621 |
| 55 | [GO:0005774](file:///E:\2018-7-3%E7%81%AB%E9%BE%99%E6%9E%9C%E8%BD%AC%E5%BD%95%E7%BB%84%E6%B5%8B%E5%BA%8F\%E5%AE%8C%E6%95%B4%E7%89%88%E6%95%B0%E6%8D%AE\GDR3855-Hylocereus_undulatus_Britt-12-RNAseq_result\4_Function\2_Group_Diff_Function\UP_DOWN\GO\NL-VS-L1.C.html#gene55) | vacuolar membrane | 1 (0.15%) | 1 (0.05%) | 0.368848 | 0.999621 |
| 56 | [GO:0031225](file:///E:\2018-7-3%E7%81%AB%E9%BE%99%E6%9E%9C%E8%BD%AC%E5%BD%95%E7%BB%84%E6%B5%8B%E5%BA%8F\%E5%AE%8C%E6%95%B4%E7%89%88%E6%95%B0%E6%8D%AE\GDR3855-Hylocereus_undulatus_Britt-12-RNAseq_result\4_Function\2_Group_Diff_Function\UP_DOWN\GO\NL-VS-L1.C.html#gene56) | anchored component of membrane | 1 (0.15%) | 1 (0.05%) | 0.368848 | 0.999621 |
| 57 | [GO:0032300](file:///E:\2018-7-3%E7%81%AB%E9%BE%99%E6%9E%9C%E8%BD%AC%E5%BD%95%E7%BB%84%E6%B5%8B%E5%BA%8F\%E5%AE%8C%E6%95%B4%E7%89%88%E6%95%B0%E6%8D%AE\GDR3855-Hylocereus_undulatus_Britt-12-RNAseq_result\4_Function\2_Group_Diff_Function\UP_DOWN\GO\NL-VS-L1.C.html#gene57) | mismatch repair complex | 1 (0.15%) | 1 (0.05%) | 0.368848 | 0.999621 |
| 58 | [GO:1990391](file:///E:\2018-7-3%E7%81%AB%E9%BE%99%E6%9E%9C%E8%BD%AC%E5%BD%95%E7%BB%84%E6%B5%8B%E5%BA%8F\%E5%AE%8C%E6%95%B4%E7%89%88%E6%95%B0%E6%8D%AE\GDR3855-Hylocereus_undulatus_Britt-12-RNAseq_result\4_Function\2_Group_Diff_Function\UP_DOWN\GO\NL-VS-L1.C.html#gene58) | DNA repair complex | 1 (0.15%) | 1 (0.05%) | 0.368848 | 0.999621 |
| 59 | [GO:0044435](file:///E:\2018-7-3%E7%81%AB%E9%BE%99%E6%9E%9C%E8%BD%AC%E5%BD%95%E7%BB%84%E6%B5%8B%E5%BA%8F\%E5%AE%8C%E6%95%B4%E7%89%88%E6%95%B0%E6%8D%AE\GDR3855-Hylocereus_undulatus_Britt-12-RNAseq_result\4_Function\2_Group_Diff_Function\UP_DOWN\GO\NL-VS-L1.C.html#gene59) | plastid part | 62 (9.09%) | 162 (8.76%) | 0.380850 | 0.999621 |
| 60 | [GO:0044455](file:///E:\2018-7-3%E7%81%AB%E9%BE%99%E6%9E%9C%E8%BD%AC%E5%BD%95%E7%BB%84%E6%B5%8B%E5%BA%8F\%E5%AE%8C%E6%95%B4%E7%89%88%E6%95%B0%E6%8D%AE\GDR3855-Hylocereus_undulatus_Britt-12-RNAseq_result\4_Function\2_Group_Diff_Function\UP_DOWN\GO\NL-VS-L1.C.html#gene60) | mitochondrial membrane part | 5 (0.73%) | 11 (0.59%) | 0.381631 | 0.999621 |
| 61 | [GO:0005576](file:///E:\2018-7-3%E7%81%AB%E9%BE%99%E6%9E%9C%E8%BD%AC%E5%BD%95%E7%BB%84%E6%B5%8B%E5%BA%8F\%E5%AE%8C%E6%95%B4%E7%89%88%E6%95%B0%E6%8D%AE\GDR3855-Hylocereus_undulatus_Britt-12-RNAseq_result\4_Function\2_Group_Diff_Function\UP_DOWN\GO\NL-VS-L1.C.html#gene61) | extracellular region | 21 (3.08%) | 53 (2.87%) | 0.387663 | 0.999621 |
| 62 | [GO:0030119](file:///E:\2018-7-3%E7%81%AB%E9%BE%99%E6%9E%9C%E8%BD%AC%E5%BD%95%E7%BB%84%E6%B5%8B%E5%BA%8F\%E5%AE%8C%E6%95%B4%E7%89%88%E6%95%B0%E6%8D%AE\GDR3855-Hylocereus_undulatus_Britt-12-RNAseq_result\4_Function\2_Group_Diff_Function\UP_DOWN\GO\NL-VS-L1.C.html#gene62) | AP-type membrane coat adaptor complex | 3 (0.44%) | 6 (0.32%) | 0.391217 | 0.999621 |
| 63 | [GO:0070993](file:///E:\2018-7-3%E7%81%AB%E9%BE%99%E6%9E%9C%E8%BD%AC%E5%BD%95%E7%BB%84%E6%B5%8B%E5%BA%8F\%E5%AE%8C%E6%95%B4%E7%89%88%E6%95%B0%E6%8D%AE\GDR3855-Hylocereus_undulatus_Britt-12-RNAseq_result\4_Function\2_Group_Diff_Function\UP_DOWN\GO\NL-VS-L1.C.html#gene63) | translation preinitiation complex | 3 (0.44%) | 6 (0.32%) | 0.391217 | 0.999621 |
| 64 | [GO:0031974](file:///E:\2018-7-3%E7%81%AB%E9%BE%99%E6%9E%9C%E8%BD%AC%E5%BD%95%E7%BB%84%E6%B5%8B%E5%BA%8F\%E5%AE%8C%E6%95%B4%E7%89%88%E6%95%B0%E6%8D%AE\GDR3855-Hylocereus_undulatus_Britt-12-RNAseq_result\4_Function\2_Group_Diff_Function\UP_DOWN\GO\NL-VS-L1.C.html#gene64) | membrane-enclosed lumen | 33 (4.84%) | 86 (4.65%) | 0.425838 | 0.999621 |
| 65 | [GO:0031981](file:///E:\2018-7-3%E7%81%AB%E9%BE%99%E6%9E%9C%E8%BD%AC%E5%BD%95%E7%BB%84%E6%B5%8B%E5%BA%8F\%E5%AE%8C%E6%95%B4%E7%89%88%E6%95%B0%E6%8D%AE\GDR3855-Hylocereus_undulatus_Britt-12-RNAseq_result\4_Function\2_Group_Diff_Function\UP_DOWN\GO\NL-VS-L1.C.html#gene65) | nuclear lumen | 33 (4.84%) | 86 (4.65%) | 0.425838 | 0.999621 |
| 66 | [GO:0043233](file:///E:\2018-7-3%E7%81%AB%E9%BE%99%E6%9E%9C%E8%BD%AC%E5%BD%95%E7%BB%84%E6%B5%8B%E5%BA%8F\%E5%AE%8C%E6%95%B4%E7%89%88%E6%95%B0%E6%8D%AE\GDR3855-Hylocereus_undulatus_Britt-12-RNAseq_result\4_Function\2_Group_Diff_Function\UP_DOWN\GO\NL-VS-L1.C.html#gene66) | organelle lumen | 33 (4.84%) | 86 (4.65%) | 0.425838 | 0.999621 |
| 67 | [GO:0070013](file:///E:\2018-7-3%E7%81%AB%E9%BE%99%E6%9E%9C%E8%BD%AC%E5%BD%95%E7%BB%84%E6%B5%8B%E5%BA%8F\%E5%AE%8C%E6%95%B4%E7%89%88%E6%95%B0%E6%8D%AE\GDR3855-Hylocereus_undulatus_Britt-12-RNAseq_result\4_Function\2_Group_Diff_Function\UP_DOWN\GO\NL-VS-L1.C.html#gene67) | intracellular organelle lumen | 33 (4.84%) | 86 (4.65%) | 0.425838 | 0.999621 |
| 68 | [GO:0005746](file:///E:\2018-7-3%E7%81%AB%E9%BE%99%E6%9E%9C%E8%BD%AC%E5%BD%95%E7%BB%84%E6%B5%8B%E5%BA%8F\%E5%AE%8C%E6%95%B4%E7%89%88%E6%95%B0%E6%8D%AE\GDR3855-Hylocereus_undulatus_Britt-12-RNAseq_result\4_Function\2_Group_Diff_Function\UP_DOWN\GO\NL-VS-L1.C.html#gene68) | mitochondrial respiratory chain | 4 (0.59%) | 9 (0.49%) | 0.438621 | 0.999621 |
| 69 | [GO:0070469](file:///E:\2018-7-3%E7%81%AB%E9%BE%99%E6%9E%9C%E8%BD%AC%E5%BD%95%E7%BB%84%E6%B5%8B%E5%BA%8F\%E5%AE%8C%E6%95%B4%E7%89%88%E6%95%B0%E6%8D%AE\GDR3855-Hylocereus_undulatus_Britt-12-RNAseq_result\4_Function\2_Group_Diff_Function\UP_DOWN\GO\NL-VS-L1.C.html#gene69) | respiratory chain | 4 (0.59%) | 9 (0.49%) | 0.438621 | 0.999621 |
| 70 | [GO:0044437](file:///E:\2018-7-3%E7%81%AB%E9%BE%99%E6%9E%9C%E8%BD%AC%E5%BD%95%E7%BB%84%E6%B5%8B%E5%BA%8F\%E5%AE%8C%E6%95%B4%E7%89%88%E6%95%B0%E6%8D%AE\GDR3855-Hylocereus_undulatus_Britt-12-RNAseq_result\4_Function\2_Group_Diff_Function\UP_DOWN\GO\NL-VS-L1.C.html#gene70) | vacuolar part | 17 (2.49%) | 44 (2.38%) | 0.460711 | 0.999621 |
| 71 | [GO:0005777](file:///E:\2018-7-3%E7%81%AB%E9%BE%99%E6%9E%9C%E8%BD%AC%E5%BD%95%E7%BB%84%E6%B5%8B%E5%BA%8F\%E5%AE%8C%E6%95%B4%E7%89%88%E6%95%B0%E6%8D%AE\GDR3855-Hylocereus_undulatus_Britt-12-RNAseq_result\4_Function\2_Group_Diff_Function\UP_DOWN\GO\NL-VS-L1.C.html#gene71) | peroxisome | 2 (0.29%) | 4 (0.22%) | 0.470421 | 0.999621 |
| 72 | [GO:0031968](file:///E:\2018-7-3%E7%81%AB%E9%BE%99%E6%9E%9C%E8%BD%AC%E5%BD%95%E7%BB%84%E6%B5%8B%E5%BA%8F\%E5%AE%8C%E6%95%B4%E7%89%88%E6%95%B0%E6%8D%AE\GDR3855-Hylocereus_undulatus_Britt-12-RNAseq_result\4_Function\2_Group_Diff_Function\UP_DOWN\GO\NL-VS-L1.C.html#gene72) | organelle outer membrane | 2 (0.29%) | 4 (0.22%) | 0.470421 | 0.999621 |
| 73 | [GO:0036338](file:///E:\2018-7-3%E7%81%AB%E9%BE%99%E6%9E%9C%E8%BD%AC%E5%BD%95%E7%BB%84%E6%B5%8B%E5%BA%8F\%E5%AE%8C%E6%95%B4%E7%89%88%E6%95%B0%E6%8D%AE\GDR3855-Hylocereus_undulatus_Britt-12-RNAseq_result\4_Function\2_Group_Diff_Function\UP_DOWN\GO\NL-VS-L1.C.html#gene73) | viral membrane | 2 (0.29%) | 4 (0.22%) | 0.470421 | 0.999621 |
| 74 | [GO:0005886](file:///E:\2018-7-3%E7%81%AB%E9%BE%99%E6%9E%9C%E8%BD%AC%E5%BD%95%E7%BB%84%E6%B5%8B%E5%BA%8F\%E5%AE%8C%E6%95%B4%E7%89%88%E6%95%B0%E6%8D%AE\GDR3855-Hylocereus_undulatus_Britt-12-RNAseq_result\4_Function\2_Group_Diff_Function\UP_DOWN\GO\NL-VS-L1.C.html#gene74) | plasma membrane | 3 (0.44%) | 7 (0.38%) | 0.510880 | 0.999621 |
| 75 | [GO:0044459](file:///E:\2018-7-3%E7%81%AB%E9%BE%99%E6%9E%9C%E8%BD%AC%E5%BD%95%E7%BB%84%E6%B5%8B%E5%BA%8F\%E5%AE%8C%E6%95%B4%E7%89%88%E6%95%B0%E6%8D%AE\GDR3855-Hylocereus_undulatus_Britt-12-RNAseq_result\4_Function\2_Group_Diff_Function\UP_DOWN\GO\NL-VS-L1.C.html#gene75) | plasma membrane part | 3 (0.44%) | 7 (0.38%) | 0.510880 | 0.999621 |
| 76 | [GO:0009532](file:///E:\2018-7-3%E7%81%AB%E9%BE%99%E6%9E%9C%E8%BD%AC%E5%BD%95%E7%BB%84%E6%B5%8B%E5%BA%8F\%E5%AE%8C%E6%95%B4%E7%89%88%E6%95%B0%E6%8D%AE\GDR3855-Hylocereus_undulatus_Britt-12-RNAseq_result\4_Function\2_Group_Diff_Function\UP_DOWN\GO\NL-VS-L1.C.html#gene76) | plastid stroma | 28 (4.11%) | 75 (4.06%) | 0.511991 | 0.999621 |
| 77 | [GO:0098805](file:///E:\2018-7-3%E7%81%AB%E9%BE%99%E6%9E%9C%E8%BD%AC%E5%BD%95%E7%BB%84%E6%B5%8B%E5%BA%8F\%E5%AE%8C%E6%95%B4%E7%89%88%E6%95%B0%E6%8D%AE\GDR3855-Hylocereus_undulatus_Britt-12-RNAseq_result\4_Function\2_Group_Diff_Function\UP_DOWN\GO\NL-VS-L1.C.html#gene77) | whole membrane | 7 (1.03%) | 18 (0.97%) | 0.518544 | 0.999621 |
| 78 | [GO:0042579](file:///E:\2018-7-3%E7%81%AB%E9%BE%99%E6%9E%9C%E8%BD%AC%E5%BD%95%E7%BB%84%E6%B5%8B%E5%BA%8F\%E5%AE%8C%E6%95%B4%E7%89%88%E6%95%B0%E6%8D%AE\GDR3855-Hylocereus_undulatus_Britt-12-RNAseq_result\4_Function\2_Group_Diff_Function\UP_DOWN\GO\NL-VS-L1.C.html#gene78) | microbody | 8 (1.17%) | 21 (1.14%) | 0.536519 | 0.999621 |
| 79 | [GO:0005794](file:///E:\2018-7-3%E7%81%AB%E9%BE%99%E6%9E%9C%E8%BD%AC%E5%BD%95%E7%BB%84%E6%B5%8B%E5%BA%8F\%E5%AE%8C%E6%95%B4%E7%89%88%E6%95%B0%E6%8D%AE\GDR3855-Hylocereus_undulatus_Britt-12-RNAseq_result\4_Function\2_Group_Diff_Function\UP_DOWN\GO\NL-VS-L1.C.html#gene79) | Golgi apparatus | 4 (0.59%) | 10 (0.54%) | 0.537168 | 0.999621 |
| 80 | [GO:0044431](file:///E:\2018-7-3%E7%81%AB%E9%BE%99%E6%9E%9C%E8%BD%AC%E5%BD%95%E7%BB%84%E6%B5%8B%E5%BA%8F\%E5%AE%8C%E6%95%B4%E7%89%88%E6%95%B0%E6%8D%AE\GDR3855-Hylocereus_undulatus_Britt-12-RNAseq_result\4_Function\2_Group_Diff_Function\UP_DOWN\GO\NL-VS-L1.C.html#gene80) | Golgi apparatus part | 4 (0.59%) | 10 (0.54%) | 0.537168 | 0.999621 |
| 81 | [GO:0005773](file:///E:\2018-7-3%E7%81%AB%E9%BE%99%E6%9E%9C%E8%BD%AC%E5%BD%95%E7%BB%84%E6%B5%8B%E5%BA%8F\%E5%AE%8C%E6%95%B4%E7%89%88%E6%95%B0%E6%8D%AE\GDR3855-Hylocereus_undulatus_Britt-12-RNAseq_result\4_Function\2_Group_Diff_Function\UP_DOWN\GO\NL-VS-L1.C.html#gene81) | vacuole | 20 (2.93%) | 54 (2.92%) | 0.542874 | 0.999621 |
| 82 | [GO:0016469](file:///E:\2018-7-3%E7%81%AB%E9%BE%99%E6%9E%9C%E8%BD%AC%E5%BD%95%E7%BB%84%E6%B5%8B%E5%BA%8F\%E5%AE%8C%E6%95%B4%E7%89%88%E6%95%B0%E6%8D%AE\GDR3855-Hylocereus_undulatus_Britt-12-RNAseq_result\4_Function\2_Group_Diff_Function\UP_DOWN\GO\NL-VS-L1.C.html#gene82) | proton-transporting two-sector ATPase complex | 7 (1.03%) | 19 (1.03%) | 0.587799 | 0.999621 |
| 83 | [GO:0005741](file:///E:\2018-7-3%E7%81%AB%E9%BE%99%E6%9E%9C%E8%BD%AC%E5%BD%95%E7%BB%84%E6%B5%8B%E5%BA%8F\%E5%AE%8C%E6%95%B4%E7%89%88%E6%95%B0%E6%8D%AE\GDR3855-Hylocereus_undulatus_Britt-12-RNAseq_result\4_Function\2_Group_Diff_Function\UP_DOWN\GO\NL-VS-L1.C.html#gene83) | mitochondrial outer membrane | 1 (0.15%) | 2 (0.11%) | 0.601773 | 0.999621 |
| 84 | [GO:0005819](file:///E:\2018-7-3%E7%81%AB%E9%BE%99%E6%9E%9C%E8%BD%AC%E5%BD%95%E7%BB%84%E6%B5%8B%E5%BA%8F\%E5%AE%8C%E6%95%B4%E7%89%88%E6%95%B0%E6%8D%AE\GDR3855-Hylocereus_undulatus_Britt-12-RNAseq_result\4_Function\2_Group_Diff_Function\UP_DOWN\GO\NL-VS-L1.C.html#gene84) | spindle | 1 (0.15%) | 2 (0.11%) | 0.601773 | 0.999621 |
| 85 | [GO:0005839](file:///E:\2018-7-3%E7%81%AB%E9%BE%99%E6%9E%9C%E8%BD%AC%E5%BD%95%E7%BB%84%E6%B5%8B%E5%BA%8F\%E5%AE%8C%E6%95%B4%E7%89%88%E6%95%B0%E6%8D%AE\GDR3855-Hylocereus_undulatus_Britt-12-RNAseq_result\4_Function\2_Group_Diff_Function\UP_DOWN\GO\NL-VS-L1.C.html#gene85) | proteasome core complex | 1 (0.15%) | 2 (0.11%) | 0.601773 | 0.999621 |
| 86 | [GO:0009527](file:///E:\2018-7-3%E7%81%AB%E9%BE%99%E6%9E%9C%E8%BD%AC%E5%BD%95%E7%BB%84%E6%B5%8B%E5%BA%8F\%E5%AE%8C%E6%95%B4%E7%89%88%E6%95%B0%E6%8D%AE\GDR3855-Hylocereus_undulatus_Britt-12-RNAseq_result\4_Function\2_Group_Diff_Function\UP_DOWN\GO\NL-VS-L1.C.html#gene86) | plastid outer membrane | 1 (0.15%) | 2 (0.11%) | 0.601773 | 0.999621 |
| 87 | [GO:0016604](file:///E:\2018-7-3%E7%81%AB%E9%BE%99%E6%9E%9C%E8%BD%AC%E5%BD%95%E7%BB%84%E6%B5%8B%E5%BA%8F\%E5%AE%8C%E6%95%B4%E7%89%88%E6%95%B0%E6%8D%AE\GDR3855-Hylocereus_undulatus_Britt-12-RNAseq_result\4_Function\2_Group_Diff_Function\UP_DOWN\GO\NL-VS-L1.C.html#gene87) | nuclear body | 1 (0.15%) | 2 (0.11%) | 0.601773 | 0.999621 |
| 88 | [GO:0022624](file:///E:\2018-7-3%E7%81%AB%E9%BE%99%E6%9E%9C%E8%BD%AC%E5%BD%95%E7%BB%84%E6%B5%8B%E5%BA%8F\%E5%AE%8C%E6%95%B4%E7%89%88%E6%95%B0%E6%8D%AE\GDR3855-Hylocereus_undulatus_Britt-12-RNAseq_result\4_Function\2_Group_Diff_Function\UP_DOWN\GO\NL-VS-L1.C.html#gene88) | proteasome accessory complex | 1 (0.15%) | 2 (0.11%) | 0.601773 | 0.999621 |
| 89 | [GO:0030684](file:///E:\2018-7-3%E7%81%AB%E9%BE%99%E6%9E%9C%E8%BD%AC%E5%BD%95%E7%BB%84%E6%B5%8B%E5%BA%8F\%E5%AE%8C%E6%95%B4%E7%89%88%E6%95%B0%E6%8D%AE\GDR3855-Hylocereus_undulatus_Britt-12-RNAseq_result\4_Function\2_Group_Diff_Function\UP_DOWN\GO\NL-VS-L1.C.html#gene89) | preribosome | 1 (0.15%) | 2 (0.11%) | 0.601773 | 0.999621 |
| 90 | [GO:0031306](file:///E:\2018-7-3%E7%81%AB%E9%BE%99%E6%9E%9C%E8%BD%AC%E5%BD%95%E7%BB%84%E6%B5%8B%E5%BA%8F\%E5%AE%8C%E6%95%B4%E7%89%88%E6%95%B0%E6%8D%AE\GDR3855-Hylocereus_undulatus_Britt-12-RNAseq_result\4_Function\2_Group_Diff_Function\UP_DOWN\GO\NL-VS-L1.C.html#gene90) | intrinsic component of mitochondrial outer membrane | 1 (0.15%) | 2 (0.11%) | 0.601773 | 0.999621 |
| 91 | [GO:0042995](file:///E:\2018-7-3%E7%81%AB%E9%BE%99%E6%9E%9C%E8%BD%AC%E5%BD%95%E7%BB%84%E6%B5%8B%E5%BA%8F\%E5%AE%8C%E6%95%B4%E7%89%88%E6%95%B0%E6%8D%AE\GDR3855-Hylocereus_undulatus_Britt-12-RNAseq_result\4_Function\2_Group_Diff_Function\UP_DOWN\GO\NL-VS-L1.C.html#gene91) | cell projection | 1 (0.15%) | 2 (0.11%) | 0.601773 | 0.999621 |
| 92 | [GO:0044438](file:///E:\2018-7-3%E7%81%AB%E9%BE%99%E6%9E%9C%E8%BD%AC%E5%BD%95%E7%BB%84%E6%B5%8B%E5%BA%8F\%E5%AE%8C%E6%95%B4%E7%89%88%E6%95%B0%E6%8D%AE\GDR3855-Hylocereus_undulatus_Britt-12-RNAseq_result\4_Function\2_Group_Diff_Function\UP_DOWN\GO\NL-VS-L1.C.html#gene92) | microbody part | 1 (0.15%) | 2 (0.11%) | 0.601773 | 0.999621 |
| 93 | [GO:0044439](file:///E:\2018-7-3%E7%81%AB%E9%BE%99%E6%9E%9C%E8%BD%AC%E5%BD%95%E7%BB%84%E6%B5%8B%E5%BA%8F\%E5%AE%8C%E6%95%B4%E7%89%88%E6%95%B0%E6%8D%AE\GDR3855-Hylocereus_undulatus_Britt-12-RNAseq_result\4_Function\2_Group_Diff_Function\UP_DOWN\GO\NL-VS-L1.C.html#gene93) | peroxisomal part | 1 (0.15%) | 2 (0.11%) | 0.601773 | 0.999621 |
| 94 | [GO:0098573](file:///E:\2018-7-3%E7%81%AB%E9%BE%99%E6%9E%9C%E8%BD%AC%E5%BD%95%E7%BB%84%E6%B5%8B%E5%BA%8F\%E5%AE%8C%E6%95%B4%E7%89%88%E6%95%B0%E6%8D%AE\GDR3855-Hylocereus_undulatus_Britt-12-RNAseq_result\4_Function\2_Group_Diff_Function\UP_DOWN\GO\NL-VS-L1.C.html#gene94) | intrinsic component of mitochondrial membrane | 1 (0.15%) | 2 (0.11%) | 0.601773 | 0.999621 |
| 95 | [GO:0005743](file:///E:\2018-7-3%E7%81%AB%E9%BE%99%E6%9E%9C%E8%BD%AC%E5%BD%95%E7%BB%84%E6%B5%8B%E5%BA%8F\%E5%AE%8C%E6%95%B4%E7%89%88%E6%95%B0%E6%8D%AE\GDR3855-Hylocereus_undulatus_Britt-12-RNAseq_result\4_Function\2_Group_Diff_Function\UP_DOWN\GO\NL-VS-L1.C.html#gene95) | mitochondrial inner membrane | 4 (0.59%) | 11 (0.59%) | 0.626046 | 0.999621 |
| 96 | [GO:0044428](file:///E:\2018-7-3%E7%81%AB%E9%BE%99%E6%9E%9C%E8%BD%AC%E5%BD%95%E7%BB%84%E6%B5%8B%E5%BA%8F\%E5%AE%8C%E6%95%B4%E7%89%88%E6%95%B0%E6%8D%AE\GDR3855-Hylocereus_undulatus_Britt-12-RNAseq_result\4_Function\2_Group_Diff_Function\UP_DOWN\GO\NL-VS-L1.C.html#gene96) | nuclear part | 34 (4.99%) | 95 (5.14%) | 0.628709 | 0.999621 |
| 97 | [GO:0031966](file:///E:\2018-7-3%E7%81%AB%E9%BE%99%E6%9E%9C%E8%BD%AC%E5%BD%95%E7%BB%84%E6%B5%8B%E5%BA%8F\%E5%AE%8C%E6%95%B4%E7%89%88%E6%95%B0%E6%8D%AE\GDR3855-Hylocereus_undulatus_Britt-12-RNAseq_result\4_Function\2_Group_Diff_Function\UP_DOWN\GO\NL-VS-L1.C.html#gene97) | mitochondrial membrane | 5 (0.73%) | 14 (0.76%) | 0.635058 | 0.999621 |
| 98 | [GO:0042175](file:///E:\2018-7-3%E7%81%AB%E9%BE%99%E6%9E%9C%E8%BD%AC%E5%BD%95%E7%BB%84%E6%B5%8B%E5%BA%8F\%E5%AE%8C%E6%95%B4%E7%89%88%E6%95%B0%E6%8D%AE\GDR3855-Hylocereus_undulatus_Britt-12-RNAseq_result\4_Function\2_Group_Diff_Function\UP_DOWN\GO\NL-VS-L1.C.html#gene98) | nuclear outer membrane-endoplasmic reticulum membrane network | 6 (0.88%) | 17 (0.92%) | 0.643599 | 0.999621 |
| 99 | [GO:0009536](file:///E:\2018-7-3%E7%81%AB%E9%BE%99%E6%9E%9C%E8%BD%AC%E5%BD%95%E7%BB%84%E6%B5%8B%E5%BA%8F\%E5%AE%8C%E6%95%B4%E7%89%88%E6%95%B0%E6%8D%AE\GDR3855-Hylocereus_undulatus_Britt-12-RNAseq_result\4_Function\2_Group_Diff_Function\UP_DOWN\GO\NL-VS-L1.C.html#gene99) | plastid | 119 (17.45%) | 330 (17.85%) | 0.656258 | 0.999621 |
| 100 | [GO:0044422](file:///E:\2018-7-3%E7%81%AB%E9%BE%99%E6%9E%9C%E8%BD%AC%E5%BD%95%E7%BB%84%E6%B5%8B%E5%BA%8F\%E5%AE%8C%E6%95%B4%E7%89%88%E6%95%B0%E6%8D%AE\GDR3855-Hylocereus_undulatus_Britt-12-RNAseq_result\4_Function\2_Group_Diff_Function\UP_DOWN\GO\NL-VS-L1.C.html#gene100) | organelle part | 157 (23.02%) | 435 (23.53%) | 0.672409 | 0.999621 |
| 101 | [GO:0044446](file:///E:\2018-7-3%E7%81%AB%E9%BE%99%E6%9E%9C%E8%BD%AC%E5%BD%95%E7%BB%84%E6%B5%8B%E5%BA%8F\%E5%AE%8C%E6%95%B4%E7%89%88%E6%95%B0%E6%8D%AE\GDR3855-Hylocereus_undulatus_Britt-12-RNAseq_result\4_Function\2_Group_Diff_Function\UP_DOWN\GO\NL-VS-L1.C.html#gene101) | intracellular organelle part | 157 (23.02%) | 435 (23.53%) | 0.672409 | 0.999621 |
| 102 | [GO:0005840](file:///E:\2018-7-3%E7%81%AB%E9%BE%99%E6%9E%9C%E8%BD%AC%E5%BD%95%E7%BB%84%E6%B5%8B%E5%BA%8F\%E5%AE%8C%E6%95%B4%E7%89%88%E6%95%B0%E6%8D%AE\GDR3855-Hylocereus_undulatus_Britt-12-RNAseq_result\4_Function\2_Group_Diff_Function\UP_DOWN\GO\NL-VS-L1.C.html#gene102) | ribosome | 42 (6.16%) | 119 (6.44%) | 0.678641 | 0.999621 |
| 103 | [GO:0005634](file:///E:\2018-7-3%E7%81%AB%E9%BE%99%E6%9E%9C%E8%BD%AC%E5%BD%95%E7%BB%84%E6%B5%8B%E5%BA%8F\%E5%AE%8C%E6%95%B4%E7%89%88%E6%95%B0%E6%8D%AE\GDR3855-Hylocereus_undulatus_Britt-12-RNAseq_result\4_Function\2_Group_Diff_Function\UP_DOWN\GO\NL-VS-L1.C.html#gene103) | nucleus | 34 (4.99%) | 97 (5.25%) | 0.686430 | 0.999621 |
| 104 | [GO:0030529](file:///E:\2018-7-3%E7%81%AB%E9%BE%99%E6%9E%9C%E8%BD%AC%E5%BD%95%E7%BB%84%E6%B5%8B%E5%BA%8F\%E5%AE%8C%E6%95%B4%E7%89%88%E6%95%B0%E6%8D%AE\GDR3855-Hylocereus_undulatus_Britt-12-RNAseq_result\4_Function\2_Group_Diff_Function\UP_DOWN\GO\NL-VS-L1.C.html#gene104) | intracellular ribonucleoprotein complex | 83 (12.17%) | 233 (12.6%) | 0.690109 | 0.999621 |
| 105 | [GO:1990904](file:///E:\2018-7-3%E7%81%AB%E9%BE%99%E6%9E%9C%E8%BD%AC%E5%BD%95%E7%BB%84%E6%B5%8B%E5%BA%8F\%E5%AE%8C%E6%95%B4%E7%89%88%E6%95%B0%E6%8D%AE\GDR3855-Hylocereus_undulatus_Britt-12-RNAseq_result\4_Function\2_Group_Diff_Function\UP_DOWN\GO\NL-VS-L1.C.html#gene105) | ribonucleoprotein complex | 83 (12.17%) | 233 (12.6%) | 0.690109 | 0.999621 |
| 106 | [GO:0000325](file:///E:\2018-7-3%E7%81%AB%E9%BE%99%E6%9E%9C%E8%BD%AC%E5%BD%95%E7%BB%84%E6%B5%8B%E5%BA%8F\%E5%AE%8C%E6%95%B4%E7%89%88%E6%95%B0%E6%8D%AE\GDR3855-Hylocereus_undulatus_Britt-12-RNAseq_result\4_Function\2_Group_Diff_Function\UP_DOWN\GO\NL-VS-L1.C.html#gene106) | plant-type vacuole | 3 (0.44%) | 9 (0.49%) | 0.705670 | 0.999621 |
| 107 | [GO:0019867](file:///E:\2018-7-3%E7%81%AB%E9%BE%99%E6%9E%9C%E8%BD%AC%E5%BD%95%E7%BB%84%E6%B5%8B%E5%BA%8F\%E5%AE%8C%E6%95%B4%E7%89%88%E6%95%B0%E6%8D%AE\GDR3855-Hylocereus_undulatus_Britt-12-RNAseq_result\4_Function\2_Group_Diff_Function\UP_DOWN\GO\NL-VS-L1.C.html#gene107) | outer membrane | 2 (0.29%) | 6 (0.32%) | 0.715539 | 0.999621 |
| 108 | [GO:0015934](file:///E:\2018-7-3%E7%81%AB%E9%BE%99%E6%9E%9C%E8%BD%AC%E5%BD%95%E7%BB%84%E6%B5%8B%E5%BA%8F\%E5%AE%8C%E6%95%B4%E7%89%88%E6%95%B0%E6%8D%AE\GDR3855-Hylocereus_undulatus_Britt-12-RNAseq_result\4_Function\2_Group_Diff_Function\UP_DOWN\GO\NL-VS-L1.C.html#gene108) | large ribosomal subunit | 9 (1.32%) | 27 (1.46%) | 0.716800 | 0.999621 |
| 109 | [GO:0000793](file:///E:\2018-7-3%E7%81%AB%E9%BE%99%E6%9E%9C%E8%BD%AC%E5%BD%95%E7%BB%84%E6%B5%8B%E5%BA%8F\%E5%AE%8C%E6%95%B4%E7%89%88%E6%95%B0%E6%8D%AE\GDR3855-Hylocereus_undulatus_Britt-12-RNAseq_result\4_Function\2_Group_Diff_Function\UP_DOWN\GO\NL-VS-L1.C.html#gene109) | condensed chromosome | 1 (0.15%) | 3 (0.16%) | 0.748817 | 0.999621 |
| 110 | [GO:0005667](file:///E:\2018-7-3%E7%81%AB%E9%BE%99%E6%9E%9C%E8%BD%AC%E5%BD%95%E7%BB%84%E6%B5%8B%E5%BA%8F\%E5%AE%8C%E6%95%B4%E7%89%88%E6%95%B0%E6%8D%AE\GDR3855-Hylocereus_undulatus_Britt-12-RNAseq_result\4_Function\2_Group_Diff_Function\UP_DOWN\GO\NL-VS-L1.C.html#gene110) | transcription factor complex | 1 (0.15%) | 3 (0.16%) | 0.748817 | 0.999621 |
| 111 | [GO:0044798](file:///E:\2018-7-3%E7%81%AB%E9%BE%99%E6%9E%9C%E8%BD%AC%E5%BD%95%E7%BB%84%E6%B5%8B%E5%BA%8F\%E5%AE%8C%E6%95%B4%E7%89%88%E6%95%B0%E6%8D%AE\GDR3855-Hylocereus_undulatus_Britt-12-RNAseq_result\4_Function\2_Group_Diff_Function\UP_DOWN\GO\NL-VS-L1.C.html#gene111) | nuclear transcription factor complex | 1 (0.15%) | 3 (0.16%) | 0.748817 | 0.999621 |
| 112 | [GO:0005740](file:///E:\2018-7-3%E7%81%AB%E9%BE%99%E6%9E%9C%E8%BD%AC%E5%BD%95%E7%BB%84%E6%B5%8B%E5%BA%8F\%E5%AE%8C%E6%95%B4%E7%89%88%E6%95%B0%E6%8D%AE\GDR3855-Hylocereus_undulatus_Britt-12-RNAseq_result\4_Function\2_Group_Diff_Function\UP_DOWN\GO\NL-VS-L1.C.html#gene112) | mitochondrial envelope | 8 (1.17%) | 25 (1.35%) | 0.760569 | 0.999621 |
| 113 | [GO:0033178](file:///E:\2018-7-3%E7%81%AB%E9%BE%99%E6%9E%9C%E8%BD%AC%E5%BD%95%E7%BB%84%E6%B5%8B%E5%BA%8F\%E5%AE%8C%E6%95%B4%E7%89%88%E6%95%B0%E6%8D%AE\GDR3855-Hylocereus_undulatus_Britt-12-RNAseq_result\4_Function\2_Group_Diff_Function\UP_DOWN\GO\NL-VS-L1.C.html#gene113) | proton-transporting two-sector ATPase complex, catalytic domain | 3 (0.44%) | 10 (0.54%) | 0.777885 | 0.999621 |
| 114 | [GO:0000139](file:///E:\2018-7-3%E7%81%AB%E9%BE%99%E6%9E%9C%E8%BD%AC%E5%BD%95%E7%BB%84%E6%B5%8B%E5%BA%8F\%E5%AE%8C%E6%95%B4%E7%89%88%E6%95%B0%E6%8D%AE\GDR3855-Hylocereus_undulatus_Britt-12-RNAseq_result\4_Function\2_Group_Diff_Function\UP_DOWN\GO\NL-VS-L1.C.html#gene114) | Golgi membrane | 2 (0.29%) | 7 (0.38%) | 0.797402 | 0.999621 |
| 115 | [GO:0009526](file:///E:\2018-7-3%E7%81%AB%E9%BE%99%E6%9E%9C%E8%BD%AC%E5%BD%95%E7%BB%84%E6%B5%8B%E5%BA%8F\%E5%AE%8C%E6%95%B4%E7%89%88%E6%95%B0%E6%8D%AE\GDR3855-Hylocereus_undulatus_Britt-12-RNAseq_result\4_Function\2_Group_Diff_Function\UP_DOWN\GO\NL-VS-L1.C.html#gene115) | plastid envelope | 24 (3.52%) | 73 (3.95%) | 0.800911 | 0.999621 |
| 116 | [GO:0016023](file:///E:\2018-7-3%E7%81%AB%E9%BE%99%E6%9E%9C%E8%BD%AC%E5%BD%95%E7%BB%84%E6%B5%8B%E5%BA%8F\%E5%AE%8C%E6%95%B4%E7%89%88%E6%95%B0%E6%8D%AE\GDR3855-Hylocereus_undulatus_Britt-12-RNAseq_result\4_Function\2_Group_Diff_Function\UP_DOWN\GO\NL-VS-L1.C.html#gene116) | cytoplasmic, membrane-bounded vesicle | 3 (0.44%) | 11 (0.59%) | 0.834824 | 0.999621 |
| 117 | [GO:0031410](file:///E:\2018-7-3%E7%81%AB%E9%BE%99%E6%9E%9C%E8%BD%AC%E5%BD%95%E7%BB%84%E6%B5%8B%E5%BA%8F\%E5%AE%8C%E6%95%B4%E7%89%88%E6%95%B0%E6%8D%AE\GDR3855-Hylocereus_undulatus_Britt-12-RNAseq_result\4_Function\2_Group_Diff_Function\UP_DOWN\GO\NL-VS-L1.C.html#gene117) | cytoplasmic vesicle | 3 (0.44%) | 11 (0.59%) | 0.834824 | 0.999621 |
| 118 | [GO:0031982](file:///E:\2018-7-3%E7%81%AB%E9%BE%99%E6%9E%9C%E8%BD%AC%E5%BD%95%E7%BB%84%E6%B5%8B%E5%BA%8F\%E5%AE%8C%E6%95%B4%E7%89%88%E6%95%B0%E6%8D%AE\GDR3855-Hylocereus_undulatus_Britt-12-RNAseq_result\4_Function\2_Group_Diff_Function\UP_DOWN\GO\NL-VS-L1.C.html#gene118) | vesicle | 3 (0.44%) | 11 (0.59%) | 0.834824 | 0.999621 |
| 119 | [GO:0031988](file:///E:\2018-7-3%E7%81%AB%E9%BE%99%E6%9E%9C%E8%BD%AC%E5%BD%95%E7%BB%84%E6%B5%8B%E5%BA%8F\%E5%AE%8C%E6%95%B4%E7%89%88%E6%95%B0%E6%8D%AE\GDR3855-Hylocereus_undulatus_Britt-12-RNAseq_result\4_Function\2_Group_Diff_Function\UP_DOWN\GO\NL-VS-L1.C.html#gene119) | membrane-bounded vesicle | 3 (0.44%) | 11 (0.59%) | 0.834824 | 0.999621 |
| 120 | [GO:0000314](file:///E:\2018-7-3%E7%81%AB%E9%BE%99%E6%9E%9C%E8%BD%AC%E5%BD%95%E7%BB%84%E6%B5%8B%E5%BA%8F\%E5%AE%8C%E6%95%B4%E7%89%88%E6%95%B0%E6%8D%AE\GDR3855-Hylocereus_undulatus_Britt-12-RNAseq_result\4_Function\2_Group_Diff_Function\UP_DOWN\GO\NL-VS-L1.C.html#gene120) | organellar small ribosomal subunit | 1 (0.15%) | 4 (0.22%) | 0.841616 | 0.999621 |
| 121 | [GO:0033177](file:///E:\2018-7-3%E7%81%AB%E9%BE%99%E6%9E%9C%E8%BD%AC%E5%BD%95%E7%BB%84%E6%B5%8B%E5%BA%8F\%E5%AE%8C%E6%95%B4%E7%89%88%E6%95%B0%E6%8D%AE\GDR3855-Hylocereus_undulatus_Britt-12-RNAseq_result\4_Function\2_Group_Diff_Function\UP_DOWN\GO\NL-VS-L1.C.html#gene121) | proton-transporting two-sector ATPase complex, proton-transporting domain | 1 (0.15%) | 4 (0.22%) | 0.841616 | 0.999621 |
| 122 | [GO:0000428](file:///E:\2018-7-3%E7%81%AB%E9%BE%99%E6%9E%9C%E8%BD%AC%E5%BD%95%E7%BB%84%E6%B5%8B%E5%BA%8F\%E5%AE%8C%E6%95%B4%E7%89%88%E6%95%B0%E6%8D%AE\GDR3855-Hylocereus_undulatus_Britt-12-RNAseq_result\4_Function\2_Group_Diff_Function\UP_DOWN\GO\NL-VS-L1.C.html#gene122) | DNA-directed RNA polymerase complex | 2 (0.29%) | 8 (0.43%) | 0.857652 | 0.999621 |
| 123 | [GO:0012506](file:///E:\2018-7-3%E7%81%AB%E9%BE%99%E6%9E%9C%E8%BD%AC%E5%BD%95%E7%BB%84%E6%B5%8B%E5%BA%8F\%E5%AE%8C%E6%95%B4%E7%89%88%E6%95%B0%E6%8D%AE\GDR3855-Hylocereus_undulatus_Britt-12-RNAseq_result\4_Function\2_Group_Diff_Function\UP_DOWN\GO\NL-VS-L1.C.html#gene123) | vesicle membrane | 2 (0.29%) | 8 (0.43%) | 0.857652 | 0.999621 |
| 124 | [GO:0016591](file:///E:\2018-7-3%E7%81%AB%E9%BE%99%E6%9E%9C%E8%BD%AC%E5%BD%95%E7%BB%84%E6%B5%8B%E5%BA%8F\%E5%AE%8C%E6%95%B4%E7%89%88%E6%95%B0%E6%8D%AE\GDR3855-Hylocereus_undulatus_Britt-12-RNAseq_result\4_Function\2_Group_Diff_Function\UP_DOWN\GO\NL-VS-L1.C.html#gene124) | DNA-directed RNA polymerase II, holoenzyme | 2 (0.29%) | 8 (0.43%) | 0.857652 | 0.999621 |
| 125 | [GO:0030135](file:///E:\2018-7-3%E7%81%AB%E9%BE%99%E6%9E%9C%E8%BD%AC%E5%BD%95%E7%BB%84%E6%B5%8B%E5%BA%8F\%E5%AE%8C%E6%95%B4%E7%89%88%E6%95%B0%E6%8D%AE\GDR3855-Hylocereus_undulatus_Britt-12-RNAseq_result\4_Function\2_Group_Diff_Function\UP_DOWN\GO\NL-VS-L1.C.html#gene125) | coated vesicle | 2 (0.29%) | 8 (0.43%) | 0.857652 | 0.999621 |
| 126 | [GO:0030659](file:///E:\2018-7-3%E7%81%AB%E9%BE%99%E6%9E%9C%E8%BD%AC%E5%BD%95%E7%BB%84%E6%B5%8B%E5%BA%8F\%E5%AE%8C%E6%95%B4%E7%89%88%E6%95%B0%E6%8D%AE\GDR3855-Hylocereus_undulatus_Britt-12-RNAseq_result\4_Function\2_Group_Diff_Function\UP_DOWN\GO\NL-VS-L1.C.html#gene126) | cytoplasmic vesicle membrane | 2 (0.29%) | 8 (0.43%) | 0.857652 | 0.999621 |
| 127 | [GO:0030662](file:///E:\2018-7-3%E7%81%AB%E9%BE%99%E6%9E%9C%E8%BD%AC%E5%BD%95%E7%BB%84%E6%B5%8B%E5%BA%8F\%E5%AE%8C%E6%95%B4%E7%89%88%E6%95%B0%E6%8D%AE\GDR3855-Hylocereus_undulatus_Britt-12-RNAseq_result\4_Function\2_Group_Diff_Function\UP_DOWN\GO\NL-VS-L1.C.html#gene127) | coated vesicle membrane | 2 (0.29%) | 8 (0.43%) | 0.857652 | 0.999621 |
| 128 | [GO:0030880](file:///E:\2018-7-3%E7%81%AB%E9%BE%99%E6%9E%9C%E8%BD%AC%E5%BD%95%E7%BB%84%E6%B5%8B%E5%BA%8F\%E5%AE%8C%E6%95%B4%E7%89%88%E6%95%B0%E6%8D%AE\GDR3855-Hylocereus_undulatus_Britt-12-RNAseq_result\4_Function\2_Group_Diff_Function\UP_DOWN\GO\NL-VS-L1.C.html#gene128) | RNA polymerase complex | 2 (0.29%) | 8 (0.43%) | 0.857652 | 0.999621 |
| 129 | [GO:0044433](file:///E:\2018-7-3%E7%81%AB%E9%BE%99%E6%9E%9C%E8%BD%AC%E5%BD%95%E7%BB%84%E6%B5%8B%E5%BA%8F\%E5%AE%8C%E6%95%B4%E7%89%88%E6%95%B0%E6%8D%AE\GDR3855-Hylocereus_undulatus_Britt-12-RNAseq_result\4_Function\2_Group_Diff_Function\UP_DOWN\GO\NL-VS-L1.C.html#gene129) | cytoplasmic vesicle part | 2 (0.29%) | 8 (0.43%) | 0.857652 | 0.999621 |
| 130 | [GO:0055029](file:///E:\2018-7-3%E7%81%AB%E9%BE%99%E6%9E%9C%E8%BD%AC%E5%BD%95%E7%BB%84%E6%B5%8B%E5%BA%8F\%E5%AE%8C%E6%95%B4%E7%89%88%E6%95%B0%E6%8D%AE\GDR3855-Hylocereus_undulatus_Britt-12-RNAseq_result\4_Function\2_Group_Diff_Function\UP_DOWN\GO\NL-VS-L1.C.html#gene130) | nuclear DNA-directed RNA polymerase complex | 2 (0.29%) | 8 (0.43%) | 0.857652 | 0.999621 |
| 131 | [GO:0098588](file:///E:\2018-7-3%E7%81%AB%E9%BE%99%E6%9E%9C%E8%BD%AC%E5%BD%95%E7%BB%84%E6%B5%8B%E5%BA%8F\%E5%AE%8C%E6%95%B4%E7%89%88%E6%95%B0%E6%8D%AE\GDR3855-Hylocereus_undulatus_Britt-12-RNAseq_result\4_Function\2_Group_Diff_Function\UP_DOWN\GO\NL-VS-L1.C.html#gene131) | bounding membrane of organelle | 8 (1.17%) | 28 (1.51%) | 0.868951 | 0.999621 |
| 132 | [GO:0005654](file:///E:\2018-7-3%E7%81%AB%E9%BE%99%E6%9E%9C%E8%BD%AC%E5%BD%95%E7%BB%84%E6%B5%8B%E5%BA%8F\%E5%AE%8C%E6%95%B4%E7%89%88%E6%95%B0%E6%8D%AE\GDR3855-Hylocereus_undulatus_Britt-12-RNAseq_result\4_Function\2_Group_Diff_Function\UP_DOWN\GO\NL-VS-L1.C.html#gene132) | nucleoplasm | 3 (0.44%) | 12 (0.65%) | 0.878708 | 0.999621 |
| 133 | [GO:0044451](file:///E:\2018-7-3%E7%81%AB%E9%BE%99%E6%9E%9C%E8%BD%AC%E5%BD%95%E7%BB%84%E6%B5%8B%E5%BA%8F\%E5%AE%8C%E6%95%B4%E7%89%88%E6%95%B0%E6%8D%AE\GDR3855-Hylocereus_undulatus_Britt-12-RNAseq_result\4_Function\2_Group_Diff_Function\UP_DOWN\GO\NL-VS-L1.C.html#gene133) | nucleoplasm part | 3 (0.44%) | 12 (0.65%) | 0.878708 | 0.999621 |
| 134 | [GO:0000313](file:///E:\2018-7-3%E7%81%AB%E9%BE%99%E6%9E%9C%E8%BD%AC%E5%BD%95%E7%BB%84%E6%B5%8B%E5%BA%8F\%E5%AE%8C%E6%95%B4%E7%89%88%E6%95%B0%E6%8D%AE\GDR3855-Hylocereus_undulatus_Britt-12-RNAseq_result\4_Function\2_Group_Diff_Function\UP_DOWN\GO\NL-VS-L1.C.html#gene134) | organellar ribosome | 1 (0.15%) | 5 (0.27%) | 0.900162 | 0.999621 |
| 135 | [GO:0005829](file:///E:\2018-7-3%E7%81%AB%E9%BE%99%E6%9E%9C%E8%BD%AC%E5%BD%95%E7%BB%84%E6%B5%8B%E5%BA%8F\%E5%AE%8C%E6%95%B4%E7%89%88%E6%95%B0%E6%8D%AE\GDR3855-Hylocereus_undulatus_Britt-12-RNAseq_result\4_Function\2_Group_Diff_Function\UP_DOWN\GO\NL-VS-L1.C.html#gene135) | cytosol | 1 (0.15%) | 5 (0.27%) | 0.900162 | 0.999621 |
| 136 | [GO:0031300](file:///E:\2018-7-3%E7%81%AB%E9%BE%99%E6%9E%9C%E8%BD%AC%E5%BD%95%E7%BB%84%E6%B5%8B%E5%BA%8F\%E5%AE%8C%E6%95%B4%E7%89%88%E6%95%B0%E6%8D%AE\GDR3855-Hylocereus_undulatus_Britt-12-RNAseq_result\4_Function\2_Group_Diff_Function\UP_DOWN\GO\NL-VS-L1.C.html#gene136) | intrinsic component of organelle membrane | 1 (0.15%) | 5 (0.27%) | 0.900162 | 0.999621 |
| 137 | [GO:0042170](file:///E:\2018-7-3%E7%81%AB%E9%BE%99%E6%9E%9C%E8%BD%AC%E5%BD%95%E7%BB%84%E6%B5%8B%E5%BA%8F\%E5%AE%8C%E6%95%B4%E7%89%88%E6%95%B0%E6%8D%AE\GDR3855-Hylocereus_undulatus_Britt-12-RNAseq_result\4_Function\2_Group_Diff_Function\UP_DOWN\GO\NL-VS-L1.C.html#gene137) | plastid membrane | 1 (0.15%) | 5 (0.27%) | 0.900162 | 0.999621 |
| 138 | [GO:0044445](file:///E:\2018-7-3%E7%81%AB%E9%BE%99%E6%9E%9C%E8%BD%AC%E5%BD%95%E7%BB%84%E6%B5%8B%E5%BA%8F\%E5%AE%8C%E6%95%B4%E7%89%88%E6%95%B0%E6%8D%AE\GDR3855-Hylocereus_undulatus_Britt-12-RNAseq_result\4_Function\2_Group_Diff_Function\UP_DOWN\GO\NL-VS-L1.C.html#gene138) | cytosolic part | 1 (0.15%) | 5 (0.27%) | 0.900162 | 0.999621 |
| 139 | [GO:0061695](file:///E:\2018-7-3%E7%81%AB%E9%BE%99%E6%9E%9C%E8%BD%AC%E5%BD%95%E7%BB%84%E6%B5%8B%E5%BA%8F\%E5%AE%8C%E6%95%B4%E7%89%88%E6%95%B0%E6%8D%AE\GDR3855-Hylocereus_undulatus_Britt-12-RNAseq_result\4_Function\2_Group_Diff_Function\UP_DOWN\GO\NL-VS-L1.C.html#gene139) | transferase complex, transferring phosphorus-containing groups | 2 (0.29%) | 9 (0.49%) | 0.901075 | 0.999621 |
| 140 | [GO:0005739](file:///E:\2018-7-3%E7%81%AB%E9%BE%99%E6%9E%9C%E8%BD%AC%E5%BD%95%E7%BB%84%E6%B5%8B%E5%BA%8F\%E5%AE%8C%E6%95%B4%E7%89%88%E6%95%B0%E6%8D%AE\GDR3855-Hylocereus_undulatus_Britt-12-RNAseq_result\4_Function\2_Group_Diff_Function\UP_DOWN\GO\NL-VS-L1.C.html#gene140) | mitochondrion | 8 (1.17%) | 31 (1.68%) | 0.933552 | 0.999621 |
| 141 | [GO:0044429](file:///E:\2018-7-3%E7%81%AB%E9%BE%99%E6%9E%9C%E8%BD%AC%E5%BD%95%E7%BB%84%E6%B5%8B%E5%BA%8F\%E5%AE%8C%E6%95%B4%E7%89%88%E6%95%B0%E6%8D%AE\GDR3855-Hylocereus_undulatus_Britt-12-RNAseq_result\4_Function\2_Group_Diff_Function\UP_DOWN\GO\NL-VS-L1.C.html#gene141) | mitochondrial part | 8 (1.17%) | 31 (1.68%) | 0.933552 | 0.999621 |
| 142 | [GO:1902494](file:///E:\2018-7-3%E7%81%AB%E9%BE%99%E6%9E%9C%E8%BD%AC%E5%BD%95%E7%BB%84%E6%B5%8B%E5%BA%8F\%E5%AE%8C%E6%95%B4%E7%89%88%E6%95%B0%E6%8D%AE\GDR3855-Hylocereus_undulatus_Britt-12-RNAseq_result\4_Function\2_Group_Diff_Function\UP_DOWN\GO\NL-VS-L1.C.html#gene142) | catalytic complex | 5 (0.73%) | 21 (1.14%) | 0.934503 | 0.999621 |
| 143 | [GO:0005783](file:///E:\2018-7-3%E7%81%AB%E9%BE%99%E6%9E%9C%E8%BD%AC%E5%BD%95%E7%BB%84%E6%B5%8B%E5%BA%8F\%E5%AE%8C%E6%95%B4%E7%89%88%E6%95%B0%E6%8D%AE\GDR3855-Hylocereus_undulatus_Britt-12-RNAseq_result\4_Function\2_Group_Diff_Function\UP_DOWN\GO\NL-VS-L1.C.html#gene143) | endoplasmic reticulum | 1 (0.15%) | 7 (0.38%) | 0.960368 | 0.999621 |
| 144 | [GO:0005789](file:///E:\2018-7-3%E7%81%AB%E9%BE%99%E6%9E%9C%E8%BD%AC%E5%BD%95%E7%BB%84%E6%B5%8B%E5%BA%8F\%E5%AE%8C%E6%95%B4%E7%89%88%E6%95%B0%E6%8D%AE\GDR3855-Hylocereus_undulatus_Britt-12-RNAseq_result\4_Function\2_Group_Diff_Function\UP_DOWN\GO\NL-VS-L1.C.html#gene144) | endoplasmic reticulum membrane | 1 (0.15%) | 7 (0.38%) | 0.960368 | 0.999621 |
| 145 | [GO:0044432](file:///E:\2018-7-3%E7%81%AB%E9%BE%99%E6%9E%9C%E8%BD%AC%E5%BD%95%E7%BB%84%E6%B5%8B%E5%BA%8F\%E5%AE%8C%E6%95%B4%E7%89%88%E6%95%B0%E6%8D%AE\GDR3855-Hylocereus_undulatus_Britt-12-RNAseq_result\4_Function\2_Group_Diff_Function\UP_DOWN\GO\NL-VS-L1.C.html#gene145) | endoplasmic reticulum part | 1 (0.15%) | 7 (0.38%) | 0.960368 | 0.999621 |
| 146 | [GO:0012505](file:///E:\2018-7-3%E7%81%AB%E9%BE%99%E6%9E%9C%E8%BD%AC%E5%BD%95%E7%BB%84%E6%B5%8B%E5%BA%8F\%E5%AE%8C%E6%95%B4%E7%89%88%E6%95%B0%E6%8D%AE\GDR3855-Hylocereus_undulatus_Britt-12-RNAseq_result\4_Function\2_Group_Diff_Function\UP_DOWN\GO\NL-VS-L1.C.html#gene146) | endomembrane system | 11 (1.61%) | 44 (2.38%) | 0.968091 | 0.999621 |
| 147 | [GO:0030133](file:///E:\2018-7-3%E7%81%AB%E9%BE%99%E6%9E%9C%E8%BD%AC%E5%BD%95%E7%BB%84%E6%B5%8B%E5%BA%8F\%E5%AE%8C%E6%95%B4%E7%89%88%E6%95%B0%E6%8D%AE\GDR3855-Hylocereus_undulatus_Britt-12-RNAseq_result\4_Function\2_Group_Diff_Function\UP_DOWN\GO\NL-VS-L1.C.html#gene147) | transport vesicle | 1 (0.15%) | 8 (0.43%) | 0.975042 | 0.999621 |
| 148 | [GO:0044444](file:///E:\2018-7-3%E7%81%AB%E9%BE%99%E6%9E%9C%E8%BD%AC%E5%BD%95%E7%BB%84%E6%B5%8B%E5%BA%8F\%E5%AE%8C%E6%95%B4%E7%89%88%E6%95%B0%E6%8D%AE\GDR3855-Hylocereus_undulatus_Britt-12-RNAseq_result\4_Function\2_Group_Diff_Function\UP_DOWN\GO\NL-VS-L1.C.html#gene148) | cytoplasmic part | 223 (32.7%) | 658 (35.59%) | 0.979239 | 0.999621 |
| 149 | [GO:0031967](file:///E:\2018-7-3%E7%81%AB%E9%BE%99%E6%9E%9C%E8%BD%AC%E5%BD%95%E7%BB%84%E6%B5%8B%E5%BA%8F\%E5%AE%8C%E6%95%B4%E7%89%88%E6%95%B0%E6%8D%AE\GDR3855-Hylocereus_undulatus_Britt-12-RNAseq_result\4_Function\2_Group_Diff_Function\UP_DOWN\GO\NL-VS-L1.C.html#gene149) | organelle envelope | 36 (5.28%) | 125 (6.76%) | 0.980505 | 0.999621 |
| 150 | [GO:0031975](file:///E:\2018-7-3%E7%81%AB%E9%BE%99%E6%9E%9C%E8%BD%AC%E5%BD%95%E7%BB%84%E6%B5%8B%E5%BA%8F\%E5%AE%8C%E6%95%B4%E7%89%88%E6%95%B0%E6%8D%AE\GDR3855-Hylocereus_undulatus_Britt-12-RNAseq_result\4_Function\2_Group_Diff_Function\UP_DOWN\GO\NL-VS-L1.C.html#gene150) | envelope | 36 (5.28%) | 126 (6.81%) | 0.983401 | 0.999621 |
| 151 | [GO:0005737](file:///E:\2018-7-3%E7%81%AB%E9%BE%99%E6%9E%9C%E8%BD%AC%E5%BD%95%E7%BB%84%E6%B5%8B%E5%BA%8F\%E5%AE%8C%E6%95%B4%E7%89%88%E6%95%B0%E6%8D%AE\GDR3855-Hylocereus_undulatus_Britt-12-RNAseq_result\4_Function\2_Group_Diff_Function\UP_DOWN\GO\NL-VS-L1.C.html#gene151) | cytoplasm | 223 (32.7%) | 661 (35.75%) | 0.984148 | 0.999621 |
| 152 | [GO:0043229](file:///E:\2018-7-3%E7%81%AB%E9%BE%99%E6%9E%9C%E8%BD%AC%E5%BD%95%E7%BB%84%E6%B5%8B%E5%BA%8F\%E5%AE%8C%E6%95%B4%E7%89%88%E6%95%B0%E6%8D%AE\GDR3855-Hylocereus_undulatus_Britt-12-RNAseq_result\4_Function\2_Group_Diff_Function\UP_DOWN\GO\NL-VS-L1.C.html#gene152) | intracellular organelle | 335 (49.12%) | 971 (52.51%) | 0.988771 | 0.999621 |
| 153 | [GO:0019866](file:///E:\2018-7-3%E7%81%AB%E9%BE%99%E6%9E%9C%E8%BD%AC%E5%BD%95%E7%BB%84%E6%B5%8B%E5%BA%8F\%E5%AE%8C%E6%95%B4%E7%89%88%E6%95%B0%E6%8D%AE\GDR3855-Hylocereus_undulatus_Britt-12-RNAseq_result\4_Function\2_Group_Diff_Function\UP_DOWN\GO\NL-VS-L1.C.html#gene153) | organelle inner membrane | 8 (1.17%) | 38 (2.06%) | 0.989252 | 0.999621 |
| 154 | [GO:0043226](file:///E:\2018-7-3%E7%81%AB%E9%BE%99%E6%9E%9C%E8%BD%AC%E5%BD%95%E7%BB%84%E6%B5%8B%E5%BA%8F\%E5%AE%8C%E6%95%B4%E7%89%88%E6%95%B0%E6%8D%AE\GDR3855-Hylocereus_undulatus_Britt-12-RNAseq_result\4_Function\2_Group_Diff_Function\UP_DOWN\GO\NL-VS-L1.C.html#gene154) | organelle | 335 (49.12%) | 973 (52.62%) | 0.990712 | 0.999621 |
| 155 | [GO:0031090](file:///E:\2018-7-3%E7%81%AB%E9%BE%99%E6%9E%9C%E8%BD%AC%E5%BD%95%E7%BB%84%E6%B5%8B%E5%BA%8F\%E5%AE%8C%E6%95%B4%E7%89%88%E6%95%B0%E6%8D%AE\GDR3855-Hylocereus_undulatus_Britt-12-RNAseq_result\4_Function\2_Group_Diff_Function\UP_DOWN\GO\NL-VS-L1.C.html#gene155) | organelle membrane | 16 (2.35%) | 68 (3.68%) | 0.994257 | 0.999621 |
| 156 | [GO:1990234](file:///E:\2018-7-3%E7%81%AB%E9%BE%99%E6%9E%9C%E8%BD%AC%E5%BD%95%E7%BB%84%E6%B5%8B%E5%BA%8F\%E5%AE%8C%E6%95%B4%E7%89%88%E6%95%B0%E6%8D%AE\GDR3855-Hylocereus_undulatus_Britt-12-RNAseq_result\4_Function\2_Group_Diff_Function\UP_DOWN\GO\NL-VS-L1.C.html#gene156) | transferase complex | 2 (0.29%) | 17 (0.92%) | 0.995756 | 0.999621 |
| 157 | [GO:0005623](file:///E:\2018-7-3%E7%81%AB%E9%BE%99%E6%9E%9C%E8%BD%AC%E5%BD%95%E7%BB%84%E6%B5%8B%E5%BA%8F\%E5%AE%8C%E6%95%B4%E7%89%88%E6%95%B0%E6%8D%AE\GDR3855-Hylocereus_undulatus_Britt-12-RNAseq_result\4_Function\2_Group_Diff_Function\UP_DOWN\GO\NL-VS-L1.C.html#gene157) | cell | 475 (69.65%) | 1353 (73.17%) | 0.996085 | 0.999621 |
| 158 | [GO:0044464](file:///E:\2018-7-3%E7%81%AB%E9%BE%99%E6%9E%9C%E8%BD%AC%E5%BD%95%E7%BB%84%E6%B5%8B%E5%BA%8F\%E5%AE%8C%E6%95%B4%E7%89%88%E6%95%B0%E6%8D%AE\GDR3855-Hylocereus_undulatus_Britt-12-RNAseq_result\4_Function\2_Group_Diff_Function\UP_DOWN\GO\NL-VS-L1.C.html#gene158) | cell part | 475 (69.65%) | 1353 (73.17%) | 0.996085 | 0.999621 |
| 159 | [GO:0043231](file:///E:\2018-7-3%E7%81%AB%E9%BE%99%E6%9E%9C%E8%BD%AC%E5%BD%95%E7%BB%84%E6%B5%8B%E5%BA%8F\%E5%AE%8C%E6%95%B4%E7%89%88%E6%95%B0%E6%8D%AE\GDR3855-Hylocereus_undulatus_Britt-12-RNAseq_result\4_Function\2_Group_Diff_Function\UP_DOWN\GO\NL-VS-L1.C.html#gene159) | intracellular membrane-bounded organelle | 280 (41.06%) | 838 (45.32%) | 0.997945 | 0.999621 |
| 160 | [GO:0044424](file:///E:\2018-7-3%E7%81%AB%E9%BE%99%E6%9E%9C%E8%BD%AC%E5%BD%95%E7%BB%84%E6%B5%8B%E5%BA%8F\%E5%AE%8C%E6%95%B4%E7%89%88%E6%95%B0%E6%8D%AE\GDR3855-Hylocereus_undulatus_Britt-12-RNAseq_result\4_Function\2_Group_Diff_Function\UP_DOWN\GO\NL-VS-L1.C.html#gene160) | intracellular part | 437 (64.08%) | 1266 (68.47%) | 0.999176 | 0.999621 |
| 161 | [GO:0043227](file:///E:\2018-7-3%E7%81%AB%E9%BE%99%E6%9E%9C%E8%BD%AC%E5%BD%95%E7%BB%84%E6%B5%8B%E5%BA%8F\%E5%AE%8C%E6%95%B4%E7%89%88%E6%95%B0%E6%8D%AE\GDR3855-Hylocereus_undulatus_Britt-12-RNAseq_result\4_Function\2_Group_Diff_Function\UP_DOWN\GO\NL-VS-L1.C.html#gene161) | membrane-bounded organelle | 282 (41.35%) | 854 (46.19%) | 0.999409 | 0.999621 |
| 162 | [GO:0005622](file:///E:\2018-7-3%E7%81%AB%E9%BE%99%E6%9E%9C%E8%BD%AC%E5%BD%95%E7%BB%84%E6%B5%8B%E5%BA%8F\%E5%AE%8C%E6%95%B4%E7%89%88%E6%95%B0%E6%8D%AE\GDR3855-Hylocereus_undulatus_Britt-12-RNAseq_result\4_Function\2_Group_Diff_Function\UP_DOWN\GO\NL-VS-L1.C.html#gene162) | intracellular | 441 (64.66%) | 1282 (69.33%) | 0.999621 | 0.999621 |
