# Supplementary material for: Transcriptomic analysis of flower induction for long-day pitaya by supplementary lighting in short-day winter season
Source: BMC Genomics. 2020 Apr 29;21:329. doi: 10.1186/s12864-020-6726-6 (PMC7191803; doi:10.1186/s12864-020-6726-6)
Supplement: Supplementary file 8 — Additional file 8: Supplemental S8. NL-VS-L1 GO Enrichment (Biological Process). [file 12864_2020_6726_MOESM8_ESM.docx]

| Supplemental S8 NL-VS-L1 GO Enrichment (Biological Process) | | | | | | |
| --- | --- | --- | --- | --- | --- | --- |
| **#** | **GO ID** | **Description** | **GeneRatio (1234)** | **BgRatio (3359)** | **pvalue** | **p.adjust** |
| 1 | [GO:0009767](file:///E:\2018-7-3%E7%81%AB%E9%BE%99%E6%9E%9C%E8%BD%AC%E5%BD%95%E7%BB%84%E6%B5%8B%E5%BA%8F\%E5%AE%8C%E6%95%B4%E7%89%88%E6%95%B0%E6%8D%AE\GDR3855-Hylocereus_undulatus_Britt-12-RNAseq_result\4_Function\2_Group_Diff_Function\UP_DOWN\GO\NL-VS-L1.P.html#gene1) | photosynthetic electron transport chain | 6 (0.49%) | 6 (0.18%) | 0.002439 | 0.999992 |
| 2 | [GO:0034728](file:///E:\2018-7-3%E7%81%AB%E9%BE%99%E6%9E%9C%E8%BD%AC%E5%BD%95%E7%BB%84%E6%B5%8B%E5%BA%8F\%E5%AE%8C%E6%95%B4%E7%89%88%E6%95%B0%E6%8D%AE\GDR3855-Hylocereus_undulatus_Britt-12-RNAseq_result\4_Function\2_Group_Diff_Function\UP_DOWN\GO\NL-VS-L1.P.html#gene2) | nucleosome organization | 12 (0.97%) | 18 (0.54%) | 0.009422 | 0.999992 |
| 3 | [GO:0071824](file:///E:\2018-7-3%E7%81%AB%E9%BE%99%E6%9E%9C%E8%BD%AC%E5%BD%95%E7%BB%84%E6%B5%8B%E5%BA%8F\%E5%AE%8C%E6%95%B4%E7%89%88%E6%95%B0%E6%8D%AE\GDR3855-Hylocereus_undulatus_Britt-12-RNAseq_result\4_Function\2_Group_Diff_Function\UP_DOWN\GO\NL-VS-L1.P.html#gene3) | protein-DNA complex subunit organization | 12 (0.97%) | 18 (0.54%) | 0.009422 | 0.999992 |
| 4 | [GO:0005982](file:///E:\2018-7-3%E7%81%AB%E9%BE%99%E6%9E%9C%E8%BD%AC%E5%BD%95%E7%BB%84%E6%B5%8B%E5%BA%8F\%E5%AE%8C%E6%95%B4%E7%89%88%E6%95%B0%E6%8D%AE\GDR3855-Hylocereus_undulatus_Britt-12-RNAseq_result\4_Function\2_Group_Diff_Function\UP_DOWN\GO\NL-VS-L1.P.html#gene4) | starch metabolic process | 6 (0.49%) | 7 (0.21%) | 0.011715 | 0.999992 |
| 5 | [GO:0007017](file:///E:\2018-7-3%E7%81%AB%E9%BE%99%E6%9E%9C%E8%BD%AC%E5%BD%95%E7%BB%84%E6%B5%8B%E5%BA%8F\%E5%AE%8C%E6%95%B4%E7%89%88%E6%95%B0%E6%8D%AE\GDR3855-Hylocereus_undulatus_Britt-12-RNAseq_result\4_Function\2_Group_Diff_Function\UP_DOWN\GO\NL-VS-L1.P.html#gene5) | microtubule-based process | 18 (1.46%) | 31 (0.92%) | 0.012336 | 0.999992 |
| 6 | [GO:0007010](file:///E:\2018-7-3%E7%81%AB%E9%BE%99%E6%9E%9C%E8%BD%AC%E5%BD%95%E7%BB%84%E6%B5%8B%E5%BA%8F\%E5%AE%8C%E6%95%B4%E7%89%88%E6%95%B0%E6%8D%AE\GDR3855-Hylocereus_undulatus_Britt-12-RNAseq_result\4_Function\2_Group_Diff_Function\UP_DOWN\GO\NL-VS-L1.P.html#gene6) | cytoskeleton organization | 13 (1.05%) | 21 (0.63%) | 0.016467 | 0.999992 |
| 7 | [GO:0005984](file:///E:\2018-7-3%E7%81%AB%E9%BE%99%E6%9E%9C%E8%BD%AC%E5%BD%95%E7%BB%84%E6%B5%8B%E5%BA%8F\%E5%AE%8C%E6%95%B4%E7%89%88%E6%95%B0%E6%8D%AE\GDR3855-Hylocereus_undulatus_Britt-12-RNAseq_result\4_Function\2_Group_Diff_Function\UP_DOWN\GO\NL-VS-L1.P.html#gene7) | disaccharide metabolic process | 8 (0.65%) | 11 (0.33%) | 0.016722 | 0.999992 |
| 8 | [GO:0019684](file:///E:\2018-7-3%E7%81%AB%E9%BE%99%E6%9E%9C%E8%BD%AC%E5%BD%95%E7%BB%84%E6%B5%8B%E5%BA%8F\%E5%AE%8C%E6%95%B4%E7%89%88%E6%95%B0%E6%8D%AE\GDR3855-Hylocereus_undulatus_Britt-12-RNAseq_result\4_Function\2_Group_Diff_Function\UP_DOWN\GO\NL-VS-L1.P.html#gene8) | photosynthesis, light reaction | 8 (0.65%) | 11 (0.33%) | 0.016722 | 0.999992 |
| 9 | [GO:0016052](file:///E:\2018-7-3%E7%81%AB%E9%BE%99%E6%9E%9C%E8%BD%AC%E5%BD%95%E7%BB%84%E6%B5%8B%E5%BA%8F\%E5%AE%8C%E6%95%B4%E7%89%88%E6%95%B0%E6%8D%AE\GDR3855-Hylocereus_undulatus_Britt-12-RNAseq_result\4_Function\2_Group_Diff_Function\UP_DOWN\GO\NL-VS-L1.P.html#gene9) | carbohydrate catabolic process | 10 (0.81%) | 15 (0.45%) | 0.017807 | 0.999992 |
| 10 | [GO:0000271](file:///E:\2018-7-3%E7%81%AB%E9%BE%99%E6%9E%9C%E8%BD%AC%E5%BD%95%E7%BB%84%E6%B5%8B%E5%BA%8F\%E5%AE%8C%E6%95%B4%E7%89%88%E6%95%B0%E6%8D%AE\GDR3855-Hylocereus_undulatus_Britt-12-RNAseq_result\4_Function\2_Group_Diff_Function\UP_DOWN\GO\NL-VS-L1.P.html#gene10) | polysaccharide biosynthetic process | 4 (0.32%) | 4 (0.12%) | 0.018159 | 0.999992 |
| 11 | [GO:0006528](file:///E:\2018-7-3%E7%81%AB%E9%BE%99%E6%9E%9C%E8%BD%AC%E5%BD%95%E7%BB%84%E6%B5%8B%E5%BA%8F\%E5%AE%8C%E6%95%B4%E7%89%88%E6%95%B0%E6%8D%AE\GDR3855-Hylocereus_undulatus_Britt-12-RNAseq_result\4_Function\2_Group_Diff_Function\UP_DOWN\GO\NL-VS-L1.P.html#gene11) | asparagine metabolic process | 4 (0.32%) | 4 (0.12%) | 0.018159 | 0.999992 |
| 12 | [GO:0009926](file:///E:\2018-7-3%E7%81%AB%E9%BE%99%E6%9E%9C%E8%BD%AC%E5%BD%95%E7%BB%84%E6%B5%8B%E5%BA%8F\%E5%AE%8C%E6%95%B4%E7%89%88%E6%95%B0%E6%8D%AE\GDR3855-Hylocereus_undulatus_Britt-12-RNAseq_result\4_Function\2_Group_Diff_Function\UP_DOWN\GO\NL-VS-L1.P.html#gene12) | auxin polar transport | 4 (0.32%) | 4 (0.12%) | 0.018159 | 0.999992 |
| 13 | [GO:0016051](file:///E:\2018-7-3%E7%81%AB%E9%BE%99%E6%9E%9C%E8%BD%AC%E5%BD%95%E7%BB%84%E6%B5%8B%E5%BA%8F\%E5%AE%8C%E6%95%B4%E7%89%88%E6%95%B0%E6%8D%AE\GDR3855-Hylocereus_undulatus_Britt-12-RNAseq_result\4_Function\2_Group_Diff_Function\UP_DOWN\GO\NL-VS-L1.P.html#gene13) | carbohydrate biosynthetic process | 4 (0.32%) | 4 (0.12%) | 0.018159 | 0.999992 |
| 14 | [GO:0033692](file:///E:\2018-7-3%E7%81%AB%E9%BE%99%E6%9E%9C%E8%BD%AC%E5%BD%95%E7%BB%84%E6%B5%8B%E5%BA%8F\%E5%AE%8C%E6%95%B4%E7%89%88%E6%95%B0%E6%8D%AE\GDR3855-Hylocereus_undulatus_Britt-12-RNAseq_result\4_Function\2_Group_Diff_Function\UP_DOWN\GO\NL-VS-L1.P.html#gene14) | cellular polysaccharide biosynthetic process | 4 (0.32%) | 4 (0.12%) | 0.018159 | 0.999992 |
| 15 | [GO:0034637](file:///E:\2018-7-3%E7%81%AB%E9%BE%99%E6%9E%9C%E8%BD%AC%E5%BD%95%E7%BB%84%E6%B5%8B%E5%BA%8F\%E5%AE%8C%E6%95%B4%E7%89%88%E6%95%B0%E6%8D%AE\GDR3855-Hylocereus_undulatus_Britt-12-RNAseq_result\4_Function\2_Group_Diff_Function\UP_DOWN\GO\NL-VS-L1.P.html#gene15) | cellular carbohydrate biosynthetic process | 4 (0.32%) | 4 (0.12%) | 0.018159 | 0.999992 |
| 16 | [GO:0045229](file:///E:\2018-7-3%E7%81%AB%E9%BE%99%E6%9E%9C%E8%BD%AC%E5%BD%95%E7%BB%84%E6%B5%8B%E5%BA%8F\%E5%AE%8C%E6%95%B4%E7%89%88%E6%95%B0%E6%8D%AE\GDR3855-Hylocereus_undulatus_Britt-12-RNAseq_result\4_Function\2_Group_Diff_Function\UP_DOWN\GO\NL-VS-L1.P.html#gene16) | external encapsulating structure organization | 19 (1.54%) | 35 (1.04%) | 0.025064 | 0.999992 |
| 17 | [GO:0005991](file:///E:\2018-7-3%E7%81%AB%E9%BE%99%E6%9E%9C%E8%BD%AC%E5%BD%95%E7%BB%84%E6%B5%8B%E5%BA%8F\%E5%AE%8C%E6%95%B4%E7%89%88%E6%95%B0%E6%8D%AE\GDR3855-Hylocereus_undulatus_Britt-12-RNAseq_result\4_Function\2_Group_Diff_Function\UP_DOWN\GO\NL-VS-L1.P.html#gene17) | trehalose metabolic process | 5 (0.41%) | 6 (0.18%) | 0.027747 | 0.999992 |
| 18 | [GO:0006820](file:///E:\2018-7-3%E7%81%AB%E9%BE%99%E6%9E%9C%E8%BD%AC%E5%BD%95%E7%BB%84%E6%B5%8B%E5%BA%8F\%E5%AE%8C%E6%95%B4%E7%89%88%E6%95%B0%E6%8D%AE\GDR3855-Hylocereus_undulatus_Britt-12-RNAseq_result\4_Function\2_Group_Diff_Function\UP_DOWN\GO\NL-VS-L1.P.html#gene18) | anion transport | 21 (1.7%) | 40 (1.19%) | 0.029431 | 0.999992 |
| 19 | [GO:0000226](file:///E:\2018-7-3%E7%81%AB%E9%BE%99%E6%9E%9C%E8%BD%AC%E5%BD%95%E7%BB%84%E6%B5%8B%E5%BA%8F\%E5%AE%8C%E6%95%B4%E7%89%88%E6%95%B0%E6%8D%AE\GDR3855-Hylocereus_undulatus_Britt-12-RNAseq_result\4_Function\2_Group_Diff_Function\UP_DOWN\GO\NL-VS-L1.P.html#gene19) | microtubule cytoskeleton organization | 6 (0.49%) | 8 (0.24%) | 0.032288 | 0.999992 |
| 20 | [GO:0009311](file:///E:\2018-7-3%E7%81%AB%E9%BE%99%E6%9E%9C%E8%BD%AC%E5%BD%95%E7%BB%84%E6%B5%8B%E5%BA%8F\%E5%AE%8C%E6%95%B4%E7%89%88%E6%95%B0%E6%8D%AE\GDR3855-Hylocereus_undulatus_Britt-12-RNAseq_result\4_Function\2_Group_Diff_Function\UP_DOWN\GO\NL-VS-L1.P.html#gene20) | oligosaccharide metabolic process | 8 (0.65%) | 12 (0.36%) | 0.034156 | 0.999992 |
| 21 | [GO:0015979](file:///E:\2018-7-3%E7%81%AB%E9%BE%99%E6%9E%9C%E8%BD%AC%E5%BD%95%E7%BB%84%E6%B5%8B%E5%BA%8F\%E5%AE%8C%E6%95%B4%E7%89%88%E6%95%B0%E6%8D%AE\GDR3855-Hylocereus_undulatus_Britt-12-RNAseq_result\4_Function\2_Group_Diff_Function\UP_DOWN\GO\NL-VS-L1.P.html#gene21) | photosynthesis | 8 (0.65%) | 12 (0.36%) | 0.034156 | 0.999992 |
| 22 | [GO:0044262](file:///E:\2018-7-3%E7%81%AB%E9%BE%99%E6%9E%9C%E8%BD%AC%E5%BD%95%E7%BB%84%E6%B5%8B%E5%BA%8F\%E5%AE%8C%E6%95%B4%E7%89%88%E6%95%B0%E6%8D%AE\GDR3855-Hylocereus_undulatus_Britt-12-RNAseq_result\4_Function\2_Group_Diff_Function\UP_DOWN\GO\NL-VS-L1.P.html#gene22) | cellular carbohydrate metabolic process | 31 (2.51%) | 64 (1.91%) | 0.035172 | 0.999992 |
| 23 | [GO:0071555](file:///E:\2018-7-3%E7%81%AB%E9%BE%99%E6%9E%9C%E8%BD%AC%E5%BD%95%E7%BB%84%E6%B5%8B%E5%BA%8F\%E5%AE%8C%E6%95%B4%E7%89%88%E6%95%B0%E6%8D%AE\GDR3855-Hylocereus_undulatus_Britt-12-RNAseq_result\4_Function\2_Group_Diff_Function\UP_DOWN\GO\NL-VS-L1.P.html#gene23) | cell wall organization | 16 (1.3%) | 30 (0.89%) | 0.046232 | 0.999992 |
| 24 | [GO:0005977](file:///E:\2018-7-3%E7%81%AB%E9%BE%99%E6%9E%9C%E8%BD%AC%E5%BD%95%E7%BB%84%E6%B5%8B%E5%BA%8F\%E5%AE%8C%E6%95%B4%E7%89%88%E6%95%B0%E6%8D%AE\GDR3855-Hylocereus_undulatus_Britt-12-RNAseq_result\4_Function\2_Group_Diff_Function\UP_DOWN\GO\NL-VS-L1.P.html#gene24) | glycogen metabolic process | 3 (0.24%) | 3 (0.09%) | 0.049505 | 0.999992 |
| 25 | [GO:0006112](file:///E:\2018-7-3%E7%81%AB%E9%BE%99%E6%9E%9C%E8%BD%AC%E5%BD%95%E7%BB%84%E6%B5%8B%E5%BA%8F\%E5%AE%8C%E6%95%B4%E7%89%88%E6%95%B0%E6%8D%AE\GDR3855-Hylocereus_undulatus_Britt-12-RNAseq_result\4_Function\2_Group_Diff_Function\UP_DOWN\GO\NL-VS-L1.P.html#gene25) | energy reserve metabolic process | 3 (0.24%) | 3 (0.09%) | 0.049505 | 0.999992 |
| 26 | [GO:0009414](file:///E:\2018-7-3%E7%81%AB%E9%BE%99%E6%9E%9C%E8%BD%AC%E5%BD%95%E7%BB%84%E6%B5%8B%E5%BA%8F\%E5%AE%8C%E6%95%B4%E7%89%88%E6%95%B0%E6%8D%AE\GDR3855-Hylocereus_undulatus_Britt-12-RNAseq_result\4_Function\2_Group_Diff_Function\UP_DOWN\GO\NL-VS-L1.P.html#gene26) | response to water deprivation | 3 (0.24%) | 3 (0.09%) | 0.049505 | 0.999992 |
| 27 | [GO:0009808](file:///E:\2018-7-3%E7%81%AB%E9%BE%99%E6%9E%9C%E8%BD%AC%E5%BD%95%E7%BB%84%E6%B5%8B%E5%BA%8F\%E5%AE%8C%E6%95%B4%E7%89%88%E6%95%B0%E6%8D%AE\GDR3855-Hylocereus_undulatus_Britt-12-RNAseq_result\4_Function\2_Group_Diff_Function\UP_DOWN\GO\NL-VS-L1.P.html#gene27) | lignin metabolic process | 3 (0.24%) | 3 (0.09%) | 0.049505 | 0.999992 |
| 28 | [GO:0010074](file:///E:\2018-7-3%E7%81%AB%E9%BE%99%E6%9E%9C%E8%BD%AC%E5%BD%95%E7%BB%84%E6%B5%8B%E5%BA%8F\%E5%AE%8C%E6%95%B4%E7%89%88%E6%95%B0%E6%8D%AE\GDR3855-Hylocereus_undulatus_Britt-12-RNAseq_result\4_Function\2_Group_Diff_Function\UP_DOWN\GO\NL-VS-L1.P.html#gene28) | maintenance of meristem identity | 3 (0.24%) | 3 (0.09%) | 0.049505 | 0.999992 |
| 29 | [GO:0015977](file:///E:\2018-7-3%E7%81%AB%E9%BE%99%E6%9E%9C%E8%BD%AC%E5%BD%95%E7%BB%84%E6%B5%8B%E5%BA%8F\%E5%AE%8C%E6%95%B4%E7%89%88%E6%95%B0%E6%8D%AE\GDR3855-Hylocereus_undulatus_Britt-12-RNAseq_result\4_Function\2_Group_Diff_Function\UP_DOWN\GO\NL-VS-L1.P.html#gene29) | carbon fixation | 3 (0.24%) | 3 (0.09%) | 0.049505 | 0.999992 |
| 30 | [GO:0019827](file:///E:\2018-7-3%E7%81%AB%E9%BE%99%E6%9E%9C%E8%BD%AC%E5%BD%95%E7%BB%84%E6%B5%8B%E5%BA%8F\%E5%AE%8C%E6%95%B4%E7%89%88%E6%95%B0%E6%8D%AE\GDR3855-Hylocereus_undulatus_Britt-12-RNAseq_result\4_Function\2_Group_Diff_Function\UP_DOWN\GO\NL-VS-L1.P.html#gene30) | stem cell population maintenance | 3 (0.24%) | 3 (0.09%) | 0.049505 | 0.999992 |
| 31 | [GO:0035434](file:///E:\2018-7-3%E7%81%AB%E9%BE%99%E6%9E%9C%E8%BD%AC%E5%BD%95%E7%BB%84%E6%B5%8B%E5%BA%8F\%E5%AE%8C%E6%95%B4%E7%89%88%E6%95%B0%E6%8D%AE\GDR3855-Hylocereus_undulatus_Britt-12-RNAseq_result\4_Function\2_Group_Diff_Function\UP_DOWN\GO\NL-VS-L1.P.html#gene31) | copper ion transmembrane transport | 3 (0.24%) | 3 (0.09%) | 0.049505 | 0.999992 |
| 32 | [GO:0042545](file:///E:\2018-7-3%E7%81%AB%E9%BE%99%E6%9E%9C%E8%BD%AC%E5%BD%95%E7%BB%84%E6%B5%8B%E5%BA%8F\%E5%AE%8C%E6%95%B4%E7%89%88%E6%95%B0%E6%8D%AE\GDR3855-Hylocereus_undulatus_Britt-12-RNAseq_result\4_Function\2_Group_Diff_Function\UP_DOWN\GO\NL-VS-L1.P.html#gene32) | cell wall modification | 3 (0.24%) | 3 (0.09%) | 0.049505 | 0.999992 |
| 33 | [GO:0052646](file:///E:\2018-7-3%E7%81%AB%E9%BE%99%E6%9E%9C%E8%BD%AC%E5%BD%95%E7%BB%84%E6%B5%8B%E5%BA%8F\%E5%AE%8C%E6%95%B4%E7%89%88%E6%95%B0%E6%8D%AE\GDR3855-Hylocereus_undulatus_Britt-12-RNAseq_result\4_Function\2_Group_Diff_Function\UP_DOWN\GO\NL-VS-L1.P.html#gene33) | alditol phosphate metabolic process | 3 (0.24%) | 3 (0.09%) | 0.049505 | 0.999992 |
| 34 | [GO:0098727](file:///E:\2018-7-3%E7%81%AB%E9%BE%99%E6%9E%9C%E8%BD%AC%E5%BD%95%E7%BB%84%E6%B5%8B%E5%BA%8F\%E5%AE%8C%E6%95%B4%E7%89%88%E6%95%B0%E6%8D%AE\GDR3855-Hylocereus_undulatus_Britt-12-RNAseq_result\4_Function\2_Group_Diff_Function\UP_DOWN\GO\NL-VS-L1.P.html#gene34) | maintenance of cell number | 3 (0.24%) | 3 (0.09%) | 0.049505 | 0.999992 |
| 35 | [GO:0008213](file:///E:\2018-7-3%E7%81%AB%E9%BE%99%E6%9E%9C%E8%BD%AC%E5%BD%95%E7%BB%84%E6%B5%8B%E5%BA%8F\%E5%AE%8C%E6%95%B4%E7%89%88%E6%95%B0%E6%8D%AE\GDR3855-Hylocereus_undulatus_Britt-12-RNAseq_result\4_Function\2_Group_Diff_Function\UP_DOWN\GO\NL-VS-L1.P.html#gene35) | protein alkylation | 8 (0.65%) | 13 (0.39%) | 0.060671 | 0.999992 |
| 36 | [GO:0009066](file:///E:\2018-7-3%E7%81%AB%E9%BE%99%E6%9E%9C%E8%BD%AC%E5%BD%95%E7%BB%84%E6%B5%8B%E5%BA%8F\%E5%AE%8C%E6%95%B4%E7%89%88%E6%95%B0%E6%8D%AE\GDR3855-Hylocereus_undulatus_Britt-12-RNAseq_result\4_Function\2_Group_Diff_Function\UP_DOWN\GO\NL-VS-L1.P.html#gene36) | aspartate family amino acid metabolic process | 8 (0.65%) | 13 (0.39%) | 0.060671 | 0.999992 |
| 37 | [GO:0071822](file:///E:\2018-7-3%E7%81%AB%E9%BE%99%E6%9E%9C%E8%BD%AC%E5%BD%95%E7%BB%84%E6%B5%8B%E5%BA%8F\%E5%AE%8C%E6%95%B4%E7%89%88%E6%95%B0%E6%8D%AE\GDR3855-Hylocereus_undulatus_Britt-12-RNAseq_result\4_Function\2_Group_Diff_Function\UP_DOWN\GO\NL-VS-L1.P.html#gene37) | protein complex subunit organization | 32 (2.59%) | 69 (2.05%) | 0.061757 | 0.999992 |
| 38 | [GO:0006825](file:///E:\2018-7-3%E7%81%AB%E9%BE%99%E6%9E%9C%E8%BD%AC%E5%BD%95%E7%BB%84%E6%B5%8B%E5%BA%8F\%E5%AE%8C%E6%95%B4%E7%89%88%E6%95%B0%E6%8D%AE\GDR3855-Hylocereus_undulatus_Britt-12-RNAseq_result\4_Function\2_Group_Diff_Function\UP_DOWN\GO\NL-VS-L1.P.html#gene38) | copper ion transport | 4 (0.32%) | 5 (0.15%) | 0.064164 | 0.999992 |
| 39 | [GO:0009737](file:///E:\2018-7-3%E7%81%AB%E9%BE%99%E6%9E%9C%E8%BD%AC%E5%BD%95%E7%BB%84%E6%B5%8B%E5%BA%8F\%E5%AE%8C%E6%95%B4%E7%89%88%E6%95%B0%E6%8D%AE\GDR3855-Hylocereus_undulatus_Britt-12-RNAseq_result\4_Function\2_Group_Diff_Function\UP_DOWN\GO\NL-VS-L1.P.html#gene39) | response to abscisic acid | 4 (0.32%) | 5 (0.15%) | 0.064164 | 0.999992 |
| 40 | [GO:0097305](file:///E:\2018-7-3%E7%81%AB%E9%BE%99%E6%9E%9C%E8%BD%AC%E5%BD%95%E7%BB%84%E6%B5%8B%E5%BA%8F\%E5%AE%8C%E6%95%B4%E7%89%88%E6%95%B0%E6%8D%AE\GDR3855-Hylocereus_undulatus_Britt-12-RNAseq_result\4_Function\2_Group_Diff_Function\UP_DOWN\GO\NL-VS-L1.P.html#gene40) | response to alcohol | 4 (0.32%) | 5 (0.15%) | 0.064164 | 0.999992 |
| 41 | [GO:0006007](file:///E:\2018-7-3%E7%81%AB%E9%BE%99%E6%9E%9C%E8%BD%AC%E5%BD%95%E7%BB%84%E6%B5%8B%E5%BA%8F\%E5%AE%8C%E6%95%B4%E7%89%88%E6%95%B0%E6%8D%AE\GDR3855-Hylocereus_undulatus_Britt-12-RNAseq_result\4_Function\2_Group_Diff_Function\UP_DOWN\GO\NL-VS-L1.P.html#gene41) | glucose catabolic process | 7 (0.57%) | 11 (0.33%) | 0.064292 | 0.999992 |
| 42 | [GO:0006479](file:///E:\2018-7-3%E7%81%AB%E9%BE%99%E6%9E%9C%E8%BD%AC%E5%BD%95%E7%BB%84%E6%B5%8B%E5%BA%8F\%E5%AE%8C%E6%95%B4%E7%89%88%E6%95%B0%E6%8D%AE\GDR3855-Hylocereus_undulatus_Britt-12-RNAseq_result\4_Function\2_Group_Diff_Function\UP_DOWN\GO\NL-VS-L1.P.html#gene42) | protein methylation | 7 (0.57%) | 11 (0.33%) | 0.064292 | 0.999992 |
| 43 | [GO:0019320](file:///E:\2018-7-3%E7%81%AB%E9%BE%99%E6%9E%9C%E8%BD%AC%E5%BD%95%E7%BB%84%E6%B5%8B%E5%BA%8F\%E5%AE%8C%E6%95%B4%E7%89%88%E6%95%B0%E6%8D%AE\GDR3855-Hylocereus_undulatus_Britt-12-RNAseq_result\4_Function\2_Group_Diff_Function\UP_DOWN\GO\NL-VS-L1.P.html#gene43) | hexose catabolic process | 7 (0.57%) | 11 (0.33%) | 0.064292 | 0.999992 |
| 44 | [GO:0044724](file:///E:\2018-7-3%E7%81%AB%E9%BE%99%E6%9E%9C%E8%BD%AC%E5%BD%95%E7%BB%84%E6%B5%8B%E5%BA%8F\%E5%AE%8C%E6%95%B4%E7%89%88%E6%95%B0%E6%8D%AE\GDR3855-Hylocereus_undulatus_Britt-12-RNAseq_result\4_Function\2_Group_Diff_Function\UP_DOWN\GO\NL-VS-L1.P.html#gene44) | single-organism carbohydrate catabolic process | 7 (0.57%) | 11 (0.33%) | 0.064292 | 0.999992 |
| 45 | [GO:0046365](file:///E:\2018-7-3%E7%81%AB%E9%BE%99%E6%9E%9C%E8%BD%AC%E5%BD%95%E7%BB%84%E6%B5%8B%E5%BA%8F\%E5%AE%8C%E6%95%B4%E7%89%88%E6%95%B0%E6%8D%AE\GDR3855-Hylocereus_undulatus_Britt-12-RNAseq_result\4_Function\2_Group_Diff_Function\UP_DOWN\GO\NL-VS-L1.P.html#gene45) | monosaccharide catabolic process | 7 (0.57%) | 11 (0.33%) | 0.064292 | 0.999992 |
| 46 | [GO:0044092](file:///E:\2018-7-3%E7%81%AB%E9%BE%99%E6%9E%9C%E8%BD%AC%E5%BD%95%E7%BB%84%E6%B5%8B%E5%BA%8F\%E5%AE%8C%E6%95%B4%E7%89%88%E6%95%B0%E6%8D%AE\GDR3855-Hylocereus_undulatus_Britt-12-RNAseq_result\4_Function\2_Group_Diff_Function\UP_DOWN\GO\NL-VS-L1.P.html#gene46) | negative regulation of molecular function | 12 (0.97%) | 22 (0.65%) | 0.066825 | 0.999992 |
| 47 | [GO:0006091](file:///E:\2018-7-3%E7%81%AB%E9%BE%99%E6%9E%9C%E8%BD%AC%E5%BD%95%E7%BB%84%E6%B5%8B%E5%BA%8F\%E5%AE%8C%E6%95%B4%E7%89%88%E6%95%B0%E6%8D%AE\GDR3855-Hylocereus_undulatus_Britt-12-RNAseq_result\4_Function\2_Group_Diff_Function\UP_DOWN\GO\NL-VS-L1.P.html#gene47) | generation of precursor metabolites and energy | 52 (4.21%) | 119 (3.54%) | 0.067023 | 0.999992 |
| 48 | [GO:0015698](file:///E:\2018-7-3%E7%81%AB%E9%BE%99%E6%9E%9C%E8%BD%AC%E5%BD%95%E7%BB%84%E6%B5%8B%E5%BA%8F\%E5%AE%8C%E6%95%B4%E7%89%88%E6%95%B0%E6%8D%AE\GDR3855-Hylocereus_undulatus_Britt-12-RNAseq_result\4_Function\2_Group_Diff_Function\UP_DOWN\GO\NL-VS-L1.P.html#gene48) | inorganic anion transport | 10 (0.81%) | 18 (0.54%) | 0.080525 | 0.999992 |
| 49 | [GO:0043648](file:///E:\2018-7-3%E7%81%AB%E9%BE%99%E6%9E%9C%E8%BD%AC%E5%BD%95%E7%BB%84%E6%B5%8B%E5%BA%8F\%E5%AE%8C%E6%95%B4%E7%89%88%E6%95%B0%E6%8D%AE\GDR3855-Hylocereus_undulatus_Britt-12-RNAseq_result\4_Function\2_Group_Diff_Function\UP_DOWN\GO\NL-VS-L1.P.html#gene49) | dicarboxylic acid metabolic process | 10 (0.81%) | 18 (0.54%) | 0.080525 | 0.999992 |
| 50 | [GO:0005976](file:///E:\2018-7-3%E7%81%AB%E9%BE%99%E6%9E%9C%E8%BD%AC%E5%BD%95%E7%BB%84%E6%B5%8B%E5%BA%8F\%E5%AE%8C%E6%95%B4%E7%89%88%E6%95%B0%E6%8D%AE\GDR3855-Hylocereus_undulatus_Britt-12-RNAseq_result\4_Function\2_Group_Diff_Function\UP_DOWN\GO\NL-VS-L1.P.html#gene50) | polysaccharide metabolic process | 31 (2.51%) | 68 (2.02%) | 0.081640 | 0.999992 |
| 51 | [GO:0006468](file:///E:\2018-7-3%E7%81%AB%E9%BE%99%E6%9E%9C%E8%BD%AC%E5%BD%95%E7%BB%84%E6%B5%8B%E5%BA%8F\%E5%AE%8C%E6%95%B4%E7%89%88%E6%95%B0%E6%8D%AE\GDR3855-Hylocereus_undulatus_Britt-12-RNAseq_result\4_Function\2_Group_Diff_Function\UP_DOWN\GO\NL-VS-L1.P.html#gene51) | protein phosphorylation | 31 (2.51%) | 68 (2.02%) | 0.081640 | 0.999992 |
| 52 | [GO:0034220](file:///E:\2018-7-3%E7%81%AB%E9%BE%99%E6%9E%9C%E8%BD%AC%E5%BD%95%E7%BB%84%E6%B5%8B%E5%BA%8F\%E5%AE%8C%E6%95%B4%E7%89%88%E6%95%B0%E6%8D%AE\GDR3855-Hylocereus_undulatus_Britt-12-RNAseq_result\4_Function\2_Group_Diff_Function\UP_DOWN\GO\NL-VS-L1.P.html#gene52) | ion transmembrane transport | 17 (1.38%) | 35 (1.04%) | 0.100917 | 0.999992 |
| 53 | [GO:0016053](file:///E:\2018-7-3%E7%81%AB%E9%BE%99%E6%9E%9C%E8%BD%AC%E5%BD%95%E7%BB%84%E6%B5%8B%E5%BA%8F\%E5%AE%8C%E6%95%B4%E7%89%88%E6%95%B0%E6%8D%AE\GDR3855-Hylocereus_undulatus_Britt-12-RNAseq_result\4_Function\2_Group_Diff_Function\UP_DOWN\GO\NL-VS-L1.P.html#gene53) | organic acid biosynthetic process | 19 (1.54%) | 40 (1.19%) | 0.105779 | 0.999992 |
| 54 | [GO:0046394](file:///E:\2018-7-3%E7%81%AB%E9%BE%99%E6%9E%9C%E8%BD%AC%E5%BD%95%E7%BB%84%E6%B5%8B%E5%BA%8F\%E5%AE%8C%E6%95%B4%E7%89%88%E6%95%B0%E6%8D%AE\GDR3855-Hylocereus_undulatus_Britt-12-RNAseq_result\4_Function\2_Group_Diff_Function\UP_DOWN\GO\NL-VS-L1.P.html#gene54) | carboxylic acid biosynthetic process | 19 (1.54%) | 40 (1.19%) | 0.105779 | 0.999992 |
| 55 | [GO:0005975](file:///E:\2018-7-3%E7%81%AB%E9%BE%99%E6%9E%9C%E8%BD%AC%E5%BD%95%E7%BB%84%E6%B5%8B%E5%BA%8F\%E5%AE%8C%E6%95%B4%E7%89%88%E6%95%B0%E6%8D%AE\GDR3855-Hylocereus_undulatus_Britt-12-RNAseq_result\4_Function\2_Group_Diff_Function\UP_DOWN\GO\NL-VS-L1.P.html#gene55) | carbohydrate metabolic process | 64 (5.19%) | 153 (4.55%) | 0.105956 | 0.999992 |
| 56 | [GO:0015849](file:///E:\2018-7-3%E7%81%AB%E9%BE%99%E6%9E%9C%E8%BD%AC%E5%BD%95%E7%BB%84%E6%B5%8B%E5%BA%8F\%E5%AE%8C%E6%95%B4%E7%89%88%E6%95%B0%E6%8D%AE\GDR3855-Hylocereus_undulatus_Britt-12-RNAseq_result\4_Function\2_Group_Diff_Function\UP_DOWN\GO\NL-VS-L1.P.html#gene56) | organic acid transport | 7 (0.57%) | 12 (0.36%) | 0.106483 | 0.999992 |
| 57 | [GO:0042044](file:///E:\2018-7-3%E7%81%AB%E9%BE%99%E6%9E%9C%E8%BD%AC%E5%BD%95%E7%BB%84%E6%B5%8B%E5%BA%8F\%E5%AE%8C%E6%95%B4%E7%89%88%E6%95%B0%E6%8D%AE\GDR3855-Hylocereus_undulatus_Britt-12-RNAseq_result\4_Function\2_Group_Diff_Function\UP_DOWN\GO\NL-VS-L1.P.html#gene57) | fluid transport | 7 (0.57%) | 12 (0.36%) | 0.106483 | 0.999992 |
| 58 | [GO:0046942](file:///E:\2018-7-3%E7%81%AB%E9%BE%99%E6%9E%9C%E8%BD%AC%E5%BD%95%E7%BB%84%E6%B5%8B%E5%BA%8F\%E5%AE%8C%E6%95%B4%E7%89%88%E6%95%B0%E6%8D%AE\GDR3855-Hylocereus_undulatus_Britt-12-RNAseq_result\4_Function\2_Group_Diff_Function\UP_DOWN\GO\NL-VS-L1.P.html#gene58) | carboxylic acid transport | 7 (0.57%) | 12 (0.36%) | 0.106483 | 0.999992 |
| 59 | [GO:0071554](file:///E:\2018-7-3%E7%81%AB%E9%BE%99%E6%9E%9C%E8%BD%AC%E5%BD%95%E7%BB%84%E6%B5%8B%E5%BA%8F\%E5%AE%8C%E6%95%B4%E7%89%88%E6%95%B0%E6%8D%AE\GDR3855-Hylocereus_undulatus_Britt-12-RNAseq_result\4_Function\2_Group_Diff_Function\UP_DOWN\GO\NL-VS-L1.P.html#gene59) | cell wall organization or biogenesis | 21 (1.7%) | 45 (1.34%) | 0.109355 | 0.999992 |
| 60 | [GO:0009642](file:///E:\2018-7-3%E7%81%AB%E9%BE%99%E6%9E%9C%E8%BD%AC%E5%BD%95%E7%BB%84%E6%B5%8B%E5%BA%8F\%E5%AE%8C%E6%95%B4%E7%89%88%E6%95%B0%E6%8D%AE\GDR3855-Hylocereus_undulatus_Britt-12-RNAseq_result\4_Function\2_Group_Diff_Function\UP_DOWN\GO\NL-VS-L1.P.html#gene60) | response to light intensity | 16 (1.3%) | 33 (0.98%) | 0.111317 | 0.999992 |
| 61 | [GO:0044264](file:///E:\2018-7-3%E7%81%AB%E9%BE%99%E6%9E%9C%E8%BD%AC%E5%BD%95%E7%BB%84%E6%B5%8B%E5%BA%8F\%E5%AE%8C%E6%95%B4%E7%89%88%E6%95%B0%E6%8D%AE\GDR3855-Hylocereus_undulatus_Britt-12-RNAseq_result\4_Function\2_Group_Diff_Function\UP_DOWN\GO\NL-VS-L1.P.html#gene61) | cellular polysaccharide metabolic process | 23 (1.86%) | 50 (1.49%) | 0.111947 | 0.999992 |
| 62 | [GO:0000097](file:///E:\2018-7-3%E7%81%AB%E9%BE%99%E6%9E%9C%E8%BD%AC%E5%BD%95%E7%BB%84%E6%B5%8B%E5%BA%8F\%E5%AE%8C%E6%95%B4%E7%89%88%E6%95%B0%E6%8D%AE\GDR3855-Hylocereus_undulatus_Britt-12-RNAseq_result\4_Function\2_Group_Diff_Function\UP_DOWN\GO\NL-VS-L1.P.html#gene62) | sulfur amino acid biosynthetic process | 13 (1.05%) | 26 (0.77%) | 0.115373 | 0.999992 |
| 63 | [GO:0048827](file:///E:\2018-7-3%E7%81%AB%E9%BE%99%E6%9E%9C%E8%BD%AC%E5%BD%95%E7%BB%84%E6%B5%8B%E5%BA%8F\%E5%AE%8C%E6%95%B4%E7%89%88%E6%95%B0%E6%8D%AE\GDR3855-Hylocereus_undulatus_Britt-12-RNAseq_result\4_Function\2_Group_Diff_Function\UP_DOWN\GO\NL-VS-L1.P.html#gene63) | phyllome development | 6 (0.49%) | 10 (0.3%) | 0.116577 | 0.999992 |
| 64 | [GO:1903047](file:///E:\2018-7-3%E7%81%AB%E9%BE%99%E6%9E%9C%E8%BD%AC%E5%BD%95%E7%BB%84%E6%B5%8B%E5%BA%8F\%E5%AE%8C%E6%95%B4%E7%89%88%E6%95%B0%E6%8D%AE\GDR3855-Hylocereus_undulatus_Britt-12-RNAseq_result\4_Function\2_Group_Diff_Function\UP_DOWN\GO\NL-VS-L1.P.html#gene64) | mitotic cell cycle process | 5 (0.41%) | 8 (0.24%) | 0.127052 | 0.999992 |
| 65 | [GO:0008652](file:///E:\2018-7-3%E7%81%AB%E9%BE%99%E6%9E%9C%E8%BD%AC%E5%BD%95%E7%BB%84%E6%B5%8B%E5%BA%8F\%E5%AE%8C%E6%95%B4%E7%89%88%E6%95%B0%E6%8D%AE\GDR3855-Hylocereus_undulatus_Britt-12-RNAseq_result\4_Function\2_Group_Diff_Function\UP_DOWN\GO\NL-VS-L1.P.html#gene65) | cellular amino acid biosynthetic process | 17 (1.38%) | 36 (1.07%) | 0.128191 | 0.999992 |
| 66 | [GO:0030243](file:///E:\2018-7-3%E7%81%AB%E9%BE%99%E6%9E%9C%E8%BD%AC%E5%BD%95%E7%BB%84%E6%B5%8B%E5%BA%8F\%E5%AE%8C%E6%95%B4%E7%89%88%E6%95%B0%E6%8D%AE\GDR3855-Hylocereus_undulatus_Britt-12-RNAseq_result\4_Function\2_Group_Diff_Function\UP_DOWN\GO\NL-VS-L1.P.html#gene66) | cellulose metabolic process | 9 (0.73%) | 17 (0.51%) | 0.128589 | 0.999992 |
| 67 | [GO:0055085](file:///E:\2018-7-3%E7%81%AB%E9%BE%99%E6%9E%9C%E8%BD%AC%E5%BD%95%E7%BB%84%E6%B5%8B%E5%BA%8F\%E5%AE%8C%E6%95%B4%E7%89%88%E6%95%B0%E6%8D%AE\GDR3855-Hylocereus_undulatus_Britt-12-RNAseq_result\4_Function\2_Group_Diff_Function\UP_DOWN\GO\NL-VS-L1.P.html#gene67) | transmembrane transport | 19 (1.54%) | 41 (1.22%) | 0.131805 | 0.999992 |
| 68 | [GO:0000086](file:///E:\2018-7-3%E7%81%AB%E9%BE%99%E6%9E%9C%E8%BD%AC%E5%BD%95%E7%BB%84%E6%B5%8B%E5%BA%8F\%E5%AE%8C%E6%95%B4%E7%89%88%E6%95%B0%E6%8D%AE\GDR3855-Hylocereus_undulatus_Britt-12-RNAseq_result\4_Function\2_Group_Diff_Function\UP_DOWN\GO\NL-VS-L1.P.html#gene68) | G2/M transition of mitotic cell cycle | 2 (0.16%) | 2 (0.06%) | 0.134892 | 0.999992 |
| 69 | [GO:0002831](file:///E:\2018-7-3%E7%81%AB%E9%BE%99%E6%9E%9C%E8%BD%AC%E5%BD%95%E7%BB%84%E6%B5%8B%E5%BA%8F\%E5%AE%8C%E6%95%B4%E7%89%88%E6%95%B0%E6%8D%AE\GDR3855-Hylocereus_undulatus_Britt-12-RNAseq_result\4_Function\2_Group_Diff_Function\UP_DOWN\GO\NL-VS-L1.P.html#gene69) | regulation of response to biotic stimulus | 2 (0.16%) | 2 (0.06%) | 0.134892 | 0.999992 |
| 70 | [GO:0006072](file:///E:\2018-7-3%E7%81%AB%E9%BE%99%E6%9E%9C%E8%BD%AC%E5%BD%95%E7%BB%84%E6%B5%8B%E5%BA%8F\%E5%AE%8C%E6%95%B4%E7%89%88%E6%95%B0%E6%8D%AE\GDR3855-Hylocereus_undulatus_Britt-12-RNAseq_result\4_Function\2_Group_Diff_Function\UP_DOWN\GO\NL-VS-L1.P.html#gene70) | glycerol-3-phosphate metabolic process | 2 (0.16%) | 2 (0.06%) | 0.134892 | 0.999992 |
| 71 | [GO:0006529](file:///E:\2018-7-3%E7%81%AB%E9%BE%99%E6%9E%9C%E8%BD%AC%E5%BD%95%E7%BB%84%E6%B5%8B%E5%BA%8F\%E5%AE%8C%E6%95%B4%E7%89%88%E6%95%B0%E6%8D%AE\GDR3855-Hylocereus_undulatus_Britt-12-RNAseq_result\4_Function\2_Group_Diff_Function\UP_DOWN\GO\NL-VS-L1.P.html#gene71) | asparagine biosynthetic process | 2 (0.16%) | 2 (0.06%) | 0.134892 | 0.999992 |
| 72 | [GO:0006536](file:///E:\2018-7-3%E7%81%AB%E9%BE%99%E6%9E%9C%E8%BD%AC%E5%BD%95%E7%BB%84%E6%B5%8B%E5%BA%8F\%E5%AE%8C%E6%95%B4%E7%89%88%E6%95%B0%E6%8D%AE\GDR3855-Hylocereus_undulatus_Britt-12-RNAseq_result\4_Function\2_Group_Diff_Function\UP_DOWN\GO\NL-VS-L1.P.html#gene72) | glutamate metabolic process | 2 (0.16%) | 2 (0.06%) | 0.134892 | 0.999992 |
| 73 | [GO:0006570](file:///E:\2018-7-3%E7%81%AB%E9%BE%99%E6%9E%9C%E8%BD%AC%E5%BD%95%E7%BB%84%E6%B5%8B%E5%BA%8F\%E5%AE%8C%E6%95%B4%E7%89%88%E6%95%B0%E6%8D%AE\GDR3855-Hylocereus_undulatus_Britt-12-RNAseq_result\4_Function\2_Group_Diff_Function\UP_DOWN\GO\NL-VS-L1.P.html#gene73) | tyrosine metabolic process | 2 (0.16%) | 2 (0.06%) | 0.134892 | 0.999992 |
| 74 | [GO:0006772](file:///E:\2018-7-3%E7%81%AB%E9%BE%99%E6%9E%9C%E8%BD%AC%E5%BD%95%E7%BB%84%E6%B5%8B%E5%BA%8F\%E5%AE%8C%E6%95%B4%E7%89%88%E6%95%B0%E6%8D%AE\GDR3855-Hylocereus_undulatus_Britt-12-RNAseq_result\4_Function\2_Group_Diff_Function\UP_DOWN\GO\NL-VS-L1.P.html#gene74) | thiamine metabolic process | 2 (0.16%) | 2 (0.06%) | 0.134892 | 0.999992 |
| 75 | [GO:0006835](file:///E:\2018-7-3%E7%81%AB%E9%BE%99%E6%9E%9C%E8%BD%AC%E5%BD%95%E7%BB%84%E6%B5%8B%E5%BA%8F\%E5%AE%8C%E6%95%B4%E7%89%88%E6%95%B0%E6%8D%AE\GDR3855-Hylocereus_undulatus_Britt-12-RNAseq_result\4_Function\2_Group_Diff_Function\UP_DOWN\GO\NL-VS-L1.P.html#gene75) | dicarboxylic acid transport | 2 (0.16%) | 2 (0.06%) | 0.134892 | 0.999992 |
| 76 | [GO:0009225](file:///E:\2018-7-3%E7%81%AB%E9%BE%99%E6%9E%9C%E8%BD%AC%E5%BD%95%E7%BB%84%E6%B5%8B%E5%BA%8F\%E5%AE%8C%E6%95%B4%E7%89%88%E6%95%B0%E6%8D%AE\GDR3855-Hylocereus_undulatus_Britt-12-RNAseq_result\4_Function\2_Group_Diff_Function\UP_DOWN\GO\NL-VS-L1.P.html#gene76) | nucleotide-sugar metabolic process | 2 (0.16%) | 2 (0.06%) | 0.134892 | 0.999992 |
| 77 | [GO:0009553](file:///E:\2018-7-3%E7%81%AB%E9%BE%99%E6%9E%9C%E8%BD%AC%E5%BD%95%E7%BB%84%E6%B5%8B%E5%BA%8F\%E5%AE%8C%E6%95%B4%E7%89%88%E6%95%B0%E6%8D%AE\GDR3855-Hylocereus_undulatus_Britt-12-RNAseq_result\4_Function\2_Group_Diff_Function\UP_DOWN\GO\NL-VS-L1.P.html#gene77) | embryo sac development | 2 (0.16%) | 2 (0.06%) | 0.134892 | 0.999992 |
| 78 | [GO:0009734](file:///E:\2018-7-3%E7%81%AB%E9%BE%99%E6%9E%9C%E8%BD%AC%E5%BD%95%E7%BB%84%E6%B5%8B%E5%BA%8F\%E5%AE%8C%E6%95%B4%E7%89%88%E6%95%B0%E6%8D%AE\GDR3855-Hylocereus_undulatus_Britt-12-RNAseq_result\4_Function\2_Group_Diff_Function\UP_DOWN\GO\NL-VS-L1.P.html#gene78) | auxin-activated signaling pathway | 2 (0.16%) | 2 (0.06%) | 0.134892 | 0.999992 |
| 79 | [GO:0009827](file:///E:\2018-7-3%E7%81%AB%E9%BE%99%E6%9E%9C%E8%BD%AC%E5%BD%95%E7%BB%84%E6%B5%8B%E5%BA%8F\%E5%AE%8C%E6%95%B4%E7%89%88%E6%95%B0%E6%8D%AE\GDR3855-Hylocereus_undulatus_Britt-12-RNAseq_result\4_Function\2_Group_Diff_Function\UP_DOWN\GO\NL-VS-L1.P.html#gene79) | plant-type cell wall modification | 2 (0.16%) | 2 (0.06%) | 0.134892 | 0.999992 |
| 80 | [GO:0010016](file:///E:\2018-7-3%E7%81%AB%E9%BE%99%E6%9E%9C%E8%BD%AC%E5%BD%95%E7%BB%84%E6%B5%8B%E5%BA%8F\%E5%AE%8C%E6%95%B4%E7%89%88%E6%95%B0%E6%8D%AE\GDR3855-Hylocereus_undulatus_Britt-12-RNAseq_result\4_Function\2_Group_Diff_Function\UP_DOWN\GO\NL-VS-L1.P.html#gene80) | shoot system morphogenesis | 2 (0.16%) | 2 (0.06%) | 0.134892 | 0.999992 |
| 81 | [GO:0010119](file:///E:\2018-7-3%E7%81%AB%E9%BE%99%E6%9E%9C%E8%BD%AC%E5%BD%95%E7%BB%84%E6%B5%8B%E5%BA%8F\%E5%AE%8C%E6%95%B4%E7%89%88%E6%95%B0%E6%8D%AE\GDR3855-Hylocereus_undulatus_Britt-12-RNAseq_result\4_Function\2_Group_Diff_Function\UP_DOWN\GO\NL-VS-L1.P.html#gene81) | regulation of stomatal movement | 2 (0.16%) | 2 (0.06%) | 0.134892 | 0.999992 |
| 82 | [GO:0010638](file:///E:\2018-7-3%E7%81%AB%E9%BE%99%E6%9E%9C%E8%BD%AC%E5%BD%95%E7%BB%84%E6%B5%8B%E5%BA%8F\%E5%AE%8C%E6%95%B4%E7%89%88%E6%95%B0%E6%8D%AE\GDR3855-Hylocereus_undulatus_Britt-12-RNAseq_result\4_Function\2_Group_Diff_Function\UP_DOWN\GO\NL-VS-L1.P.html#gene82) | positive regulation of organelle organization | 2 (0.16%) | 2 (0.06%) | 0.134892 | 0.999992 |
| 83 | [GO:0015695](file:///E:\2018-7-3%E7%81%AB%E9%BE%99%E6%9E%9C%E8%BD%AC%E5%BD%95%E7%BB%84%E6%B5%8B%E5%BA%8F\%E5%AE%8C%E6%95%B4%E7%89%88%E6%95%B0%E6%8D%AE\GDR3855-Hylocereus_undulatus_Britt-12-RNAseq_result\4_Function\2_Group_Diff_Function\UP_DOWN\GO\NL-VS-L1.P.html#gene83) | organic cation transport | 2 (0.16%) | 2 (0.06%) | 0.134892 | 0.999992 |
| 84 | [GO:0015740](file:///E:\2018-7-3%E7%81%AB%E9%BE%99%E6%9E%9C%E8%BD%AC%E5%BD%95%E7%BB%84%E6%B5%8B%E5%BA%8F\%E5%AE%8C%E6%95%B4%E7%89%88%E6%95%B0%E6%8D%AE\GDR3855-Hylocereus_undulatus_Britt-12-RNAseq_result\4_Function\2_Group_Diff_Function\UP_DOWN\GO\NL-VS-L1.P.html#gene84) | C4-dicarboxylate transport | 2 (0.16%) | 2 (0.06%) | 0.134892 | 0.999992 |
| 85 | [GO:0015743](file:///E:\2018-7-3%E7%81%AB%E9%BE%99%E6%9E%9C%E8%BD%AC%E5%BD%95%E7%BB%84%E6%B5%8B%E5%BA%8F\%E5%AE%8C%E6%95%B4%E7%89%88%E6%95%B0%E6%8D%AE\GDR3855-Hylocereus_undulatus_Britt-12-RNAseq_result\4_Function\2_Group_Diff_Function\UP_DOWN\GO\NL-VS-L1.P.html#gene85) | malate transport | 2 (0.16%) | 2 (0.06%) | 0.134892 | 0.999992 |
| 86 | [GO:0030833](file:///E:\2018-7-3%E7%81%AB%E9%BE%99%E6%9E%9C%E8%BD%AC%E5%BD%95%E7%BB%84%E6%B5%8B%E5%BA%8F\%E5%AE%8C%E6%95%B4%E7%89%88%E6%95%B0%E6%8D%AE\GDR3855-Hylocereus_undulatus_Britt-12-RNAseq_result\4_Function\2_Group_Diff_Function\UP_DOWN\GO\NL-VS-L1.P.html#gene86) | regulation of actin filament polymerization | 2 (0.16%) | 2 (0.06%) | 0.134892 | 0.999992 |
| 87 | [GO:0030838](file:///E:\2018-7-3%E7%81%AB%E9%BE%99%E6%9E%9C%E8%BD%AC%E5%BD%95%E7%BB%84%E6%B5%8B%E5%BA%8F\%E5%AE%8C%E6%95%B4%E7%89%88%E6%95%B0%E6%8D%AE\GDR3855-Hylocereus_undulatus_Britt-12-RNAseq_result\4_Function\2_Group_Diff_Function\UP_DOWN\GO\NL-VS-L1.P.html#gene87) | positive regulation of actin filament polymerization | 2 (0.16%) | 2 (0.06%) | 0.134892 | 0.999992 |
| 88 | [GO:0031334](file:///E:\2018-7-3%E7%81%AB%E9%BE%99%E6%9E%9C%E8%BD%AC%E5%BD%95%E7%BB%84%E6%B5%8B%E5%BA%8F\%E5%AE%8C%E6%95%B4%E7%89%88%E6%95%B0%E6%8D%AE\GDR3855-Hylocereus_undulatus_Britt-12-RNAseq_result\4_Function\2_Group_Diff_Function\UP_DOWN\GO\NL-VS-L1.P.html#gene88) | positive regulation of protein complex assembly | 2 (0.16%) | 2 (0.06%) | 0.134892 | 0.999992 |
| 89 | [GO:0032271](file:///E:\2018-7-3%E7%81%AB%E9%BE%99%E6%9E%9C%E8%BD%AC%E5%BD%95%E7%BB%84%E6%B5%8B%E5%BA%8F\%E5%AE%8C%E6%95%B4%E7%89%88%E6%95%B0%E6%8D%AE\GDR3855-Hylocereus_undulatus_Britt-12-RNAseq_result\4_Function\2_Group_Diff_Function\UP_DOWN\GO\NL-VS-L1.P.html#gene89) | regulation of protein polymerization | 2 (0.16%) | 2 (0.06%) | 0.134892 | 0.999992 |
| 90 | [GO:0032273](file:///E:\2018-7-3%E7%81%AB%E9%BE%99%E6%9E%9C%E8%BD%AC%E5%BD%95%E7%BB%84%E6%B5%8B%E5%BA%8F\%E5%AE%8C%E6%95%B4%E7%89%88%E6%95%B0%E6%8D%AE\GDR3855-Hylocereus_undulatus_Britt-12-RNAseq_result\4_Function\2_Group_Diff_Function\UP_DOWN\GO\NL-VS-L1.P.html#gene90) | positive regulation of protein polymerization | 2 (0.16%) | 2 (0.06%) | 0.134892 | 0.999992 |
| 91 | [GO:0040034](file:///E:\2018-7-3%E7%81%AB%E9%BE%99%E6%9E%9C%E8%BD%AC%E5%BD%95%E7%BB%84%E6%B5%8B%E5%BA%8F\%E5%AE%8C%E6%95%B4%E7%89%88%E6%95%B0%E6%8D%AE\GDR3855-Hylocereus_undulatus_Britt-12-RNAseq_result\4_Function\2_Group_Diff_Function\UP_DOWN\GO\NL-VS-L1.P.html#gene91) | regulation of development, heterochronic | 2 (0.16%) | 2 (0.06%) | 0.134892 | 0.999992 |
| 92 | [GO:0043254](file:///E:\2018-7-3%E7%81%AB%E9%BE%99%E6%9E%9C%E8%BD%AC%E5%BD%95%E7%BB%84%E6%B5%8B%E5%BA%8F\%E5%AE%8C%E6%95%B4%E7%89%88%E6%95%B0%E6%8D%AE\GDR3855-Hylocereus_undulatus_Britt-12-RNAseq_result\4_Function\2_Group_Diff_Function\UP_DOWN\GO\NL-VS-L1.P.html#gene92) | regulation of protein complex assembly | 2 (0.16%) | 2 (0.06%) | 0.134892 | 0.999992 |
| 93 | [GO:0044087](file:///E:\2018-7-3%E7%81%AB%E9%BE%99%E6%9E%9C%E8%BD%AC%E5%BD%95%E7%BB%84%E6%B5%8B%E5%BA%8F\%E5%AE%8C%E6%95%B4%E7%89%88%E6%95%B0%E6%8D%AE\GDR3855-Hylocereus_undulatus_Britt-12-RNAseq_result\4_Function\2_Group_Diff_Function\UP_DOWN\GO\NL-VS-L1.P.html#gene93) | regulation of cellular component biogenesis | 2 (0.16%) | 2 (0.06%) | 0.134892 | 0.999992 |
| 94 | [GO:0044089](file:///E:\2018-7-3%E7%81%AB%E9%BE%99%E6%9E%9C%E8%BD%AC%E5%BD%95%E7%BB%84%E6%B5%8B%E5%BA%8F\%E5%AE%8C%E6%95%B4%E7%89%88%E6%95%B0%E6%8D%AE\GDR3855-Hylocereus_undulatus_Britt-12-RNAseq_result\4_Function\2_Group_Diff_Function\UP_DOWN\GO\NL-VS-L1.P.html#gene94) | positive regulation of cellular component biogenesis | 2 (0.16%) | 2 (0.06%) | 0.134892 | 0.999992 |
| 95 | [GO:0044770](file:///E:\2018-7-3%E7%81%AB%E9%BE%99%E6%9E%9C%E8%BD%AC%E5%BD%95%E7%BB%84%E6%B5%8B%E5%BA%8F\%E5%AE%8C%E6%95%B4%E7%89%88%E6%95%B0%E6%8D%AE\GDR3855-Hylocereus_undulatus_Britt-12-RNAseq_result\4_Function\2_Group_Diff_Function\UP_DOWN\GO\NL-VS-L1.P.html#gene95) | cell cycle phase transition | 2 (0.16%) | 2 (0.06%) | 0.134892 | 0.999992 |
| 96 | [GO:0044772](file:///E:\2018-7-3%E7%81%AB%E9%BE%99%E6%9E%9C%E8%BD%AC%E5%BD%95%E7%BB%84%E6%B5%8B%E5%BA%8F\%E5%AE%8C%E6%95%B4%E7%89%88%E6%95%B0%E6%8D%AE\GDR3855-Hylocereus_undulatus_Britt-12-RNAseq_result\4_Function\2_Group_Diff_Function\UP_DOWN\GO\NL-VS-L1.P.html#gene96) | mitotic cell cycle phase transition | 2 (0.16%) | 2 (0.06%) | 0.134892 | 0.999992 |
| 97 | [GO:0044839](file:///E:\2018-7-3%E7%81%AB%E9%BE%99%E6%9E%9C%E8%BD%AC%E5%BD%95%E7%BB%84%E6%B5%8B%E5%BA%8F\%E5%AE%8C%E6%95%B4%E7%89%88%E6%95%B0%E6%8D%AE\GDR3855-Hylocereus_undulatus_Britt-12-RNAseq_result\4_Function\2_Group_Diff_Function\UP_DOWN\GO\NL-VS-L1.P.html#gene97) | cell cycle G2/M phase transition | 2 (0.16%) | 2 (0.06%) | 0.134892 | 0.999992 |
| 98 | [GO:0045010](file:///E:\2018-7-3%E7%81%AB%E9%BE%99%E6%9E%9C%E8%BD%AC%E5%BD%95%E7%BB%84%E6%B5%8B%E5%BA%8F\%E5%AE%8C%E6%95%B4%E7%89%88%E6%95%B0%E6%8D%AE\GDR3855-Hylocereus_undulatus_Britt-12-RNAseq_result\4_Function\2_Group_Diff_Function\UP_DOWN\GO\NL-VS-L1.P.html#gene98) | actin nucleation | 2 (0.16%) | 2 (0.06%) | 0.134892 | 0.999992 |
| 99 | [GO:0048506](file:///E:\2018-7-3%E7%81%AB%E9%BE%99%E6%9E%9C%E8%BD%AC%E5%BD%95%E7%BB%84%E6%B5%8B%E5%BA%8F\%E5%AE%8C%E6%95%B4%E7%89%88%E6%95%B0%E6%8D%AE\GDR3855-Hylocereus_undulatus_Britt-12-RNAseq_result\4_Function\2_Group_Diff_Function\UP_DOWN\GO\NL-VS-L1.P.html#gene99) | regulation of timing of meristematic phase transition | 2 (0.16%) | 2 (0.06%) | 0.134892 | 0.999992 |
| 100 | [GO:0048509](file:///E:\2018-7-3%E7%81%AB%E9%BE%99%E6%9E%9C%E8%BD%AC%E5%BD%95%E7%BB%84%E6%B5%8B%E5%BA%8F\%E5%AE%8C%E6%95%B4%E7%89%88%E6%95%B0%E6%8D%AE\GDR3855-Hylocereus_undulatus_Britt-12-RNAseq_result\4_Function\2_Group_Diff_Function\UP_DOWN\GO\NL-VS-L1.P.html#gene100) | regulation of meristem development | 2 (0.16%) | 2 (0.06%) | 0.134892 | 0.999992 |
| 101 | [GO:0051130](file:///E:\2018-7-3%E7%81%AB%E9%BE%99%E6%9E%9C%E8%BD%AC%E5%BD%95%E7%BB%84%E6%B5%8B%E5%BA%8F\%E5%AE%8C%E6%95%B4%E7%89%88%E6%95%B0%E6%8D%AE\GDR3855-Hylocereus_undulatus_Britt-12-RNAseq_result\4_Function\2_Group_Diff_Function\UP_DOWN\GO\NL-VS-L1.P.html#gene101) | positive regulation of cellular component organization | 2 (0.16%) | 2 (0.06%) | 0.134892 | 0.999992 |
| 102 | [GO:0051495](file:///E:\2018-7-3%E7%81%AB%E9%BE%99%E6%9E%9C%E8%BD%AC%E5%BD%95%E7%BB%84%E6%B5%8B%E5%BA%8F\%E5%AE%8C%E6%95%B4%E7%89%88%E6%95%B0%E6%8D%AE\GDR3855-Hylocereus_undulatus_Britt-12-RNAseq_result\4_Function\2_Group_Diff_Function\UP_DOWN\GO\NL-VS-L1.P.html#gene102) | positive regulation of cytoskeleton organization | 2 (0.16%) | 2 (0.06%) | 0.134892 | 0.999992 |
| 103 | [GO:0071365](file:///E:\2018-7-3%E7%81%AB%E9%BE%99%E6%9E%9C%E8%BD%AC%E5%BD%95%E7%BB%84%E6%B5%8B%E5%BA%8F\%E5%AE%8C%E6%95%B4%E7%89%88%E6%95%B0%E6%8D%AE\GDR3855-Hylocereus_undulatus_Britt-12-RNAseq_result\4_Function\2_Group_Diff_Function\UP_DOWN\GO\NL-VS-L1.P.html#gene103) | cellular response to auxin stimulus | 2 (0.16%) | 2 (0.06%) | 0.134892 | 0.999992 |
| 104 | [GO:0009617](file:///E:\2018-7-3%E7%81%AB%E9%BE%99%E6%9E%9C%E8%BD%AC%E5%BD%95%E7%BB%84%E6%B5%8B%E5%BA%8F\%E5%AE%8C%E6%95%B4%E7%89%88%E6%95%B0%E6%8D%AE\GDR3855-Hylocereus_undulatus_Britt-12-RNAseq_result\4_Function\2_Group_Diff_Function\UP_DOWN\GO\NL-VS-L1.P.html#gene104) | response to bacterium | 14 (1.13%) | 29 (0.86%) | 0.135953 | 0.999992 |
| 105 | [GO:0009793](file:///E:\2018-7-3%E7%81%AB%E9%BE%99%E6%9E%9C%E8%BD%AC%E5%BD%95%E7%BB%84%E6%B5%8B%E5%BA%8F\%E5%AE%8C%E6%95%B4%E7%89%88%E6%95%B0%E6%8D%AE\GDR3855-Hylocereus_undulatus_Britt-12-RNAseq_result\4_Function\2_Group_Diff_Function\UP_DOWN\GO\NL-VS-L1.P.html#gene105) | embryo development ending in seed dormancy | 4 (0.32%) | 6 (0.18%) | 0.136999 | 0.999992 |
| 106 | [GO:0010154](file:///E:\2018-7-3%E7%81%AB%E9%BE%99%E6%9E%9C%E8%BD%AC%E5%BD%95%E7%BB%84%E6%B5%8B%E5%BA%8F\%E5%AE%8C%E6%95%B4%E7%89%88%E6%95%B0%E6%8D%AE\GDR3855-Hylocereus_undulatus_Britt-12-RNAseq_result\4_Function\2_Group_Diff_Function\UP_DOWN\GO\NL-VS-L1.P.html#gene106) | fruit development | 4 (0.32%) | 6 (0.18%) | 0.136999 | 0.999992 |
| 107 | [GO:0043473](file:///E:\2018-7-3%E7%81%AB%E9%BE%99%E6%9E%9C%E8%BD%AC%E5%BD%95%E7%BB%84%E6%B5%8B%E5%BA%8F\%E5%AE%8C%E6%95%B4%E7%89%88%E6%95%B0%E6%8D%AE\GDR3855-Hylocereus_undulatus_Britt-12-RNAseq_result\4_Function\2_Group_Diff_Function\UP_DOWN\GO\NL-VS-L1.P.html#gene107) | pigmentation | 4 (0.32%) | 6 (0.18%) | 0.136999 | 0.999992 |
| 108 | [GO:0043476](file:///E:\2018-7-3%E7%81%AB%E9%BE%99%E6%9E%9C%E8%BD%AC%E5%BD%95%E7%BB%84%E6%B5%8B%E5%BA%8F\%E5%AE%8C%E6%95%B4%E7%89%88%E6%95%B0%E6%8D%AE\GDR3855-Hylocereus_undulatus_Britt-12-RNAseq_result\4_Function\2_Group_Diff_Function\UP_DOWN\GO\NL-VS-L1.P.html#gene108) | pigment accumulation | 4 (0.32%) | 6 (0.18%) | 0.136999 | 0.999992 |
| 109 | [GO:0043478](file:///E:\2018-7-3%E7%81%AB%E9%BE%99%E6%9E%9C%E8%BD%AC%E5%BD%95%E7%BB%84%E6%B5%8B%E5%BA%8F\%E5%AE%8C%E6%95%B4%E7%89%88%E6%95%B0%E6%8D%AE\GDR3855-Hylocereus_undulatus_Britt-12-RNAseq_result\4_Function\2_Group_Diff_Function\UP_DOWN\GO\NL-VS-L1.P.html#gene109) | pigment accumulation in response to UV light | 4 (0.32%) | 6 (0.18%) | 0.136999 | 0.999992 |
| 110 | [GO:0043479](file:///E:\2018-7-3%E7%81%AB%E9%BE%99%E6%9E%9C%E8%BD%AC%E5%BD%95%E7%BB%84%E6%B5%8B%E5%BA%8F\%E5%AE%8C%E6%95%B4%E7%89%88%E6%95%B0%E6%8D%AE\GDR3855-Hylocereus_undulatus_Britt-12-RNAseq_result\4_Function\2_Group_Diff_Function\UP_DOWN\GO\NL-VS-L1.P.html#gene110) | pigment accumulation in tissues in response to UV light | 4 (0.32%) | 6 (0.18%) | 0.136999 | 0.999992 |
| 111 | [GO:0043480](file:///E:\2018-7-3%E7%81%AB%E9%BE%99%E6%9E%9C%E8%BD%AC%E5%BD%95%E7%BB%84%E6%B5%8B%E5%BA%8F\%E5%AE%8C%E6%95%B4%E7%89%88%E6%95%B0%E6%8D%AE\GDR3855-Hylocereus_undulatus_Britt-12-RNAseq_result\4_Function\2_Group_Diff_Function\UP_DOWN\GO\NL-VS-L1.P.html#gene111) | pigment accumulation in tissues | 4 (0.32%) | 6 (0.18%) | 0.136999 | 0.999992 |
| 112 | [GO:0048316](file:///E:\2018-7-3%E7%81%AB%E9%BE%99%E6%9E%9C%E8%BD%AC%E5%BD%95%E7%BB%84%E6%B5%8B%E5%BA%8F\%E5%AE%8C%E6%95%B4%E7%89%88%E6%95%B0%E6%8D%AE\GDR3855-Hylocereus_undulatus_Britt-12-RNAseq_result\4_Function\2_Group_Diff_Function\UP_DOWN\GO\NL-VS-L1.P.html#gene112) | seed development | 4 (0.32%) | 6 (0.18%) | 0.136999 | 0.999992 |
| 113 | [GO:0022900](file:///E:\2018-7-3%E7%81%AB%E9%BE%99%E6%9E%9C%E8%BD%AC%E5%BD%95%E7%BB%84%E6%B5%8B%E5%BA%8F\%E5%AE%8C%E6%95%B4%E7%89%88%E6%95%B0%E6%8D%AE\GDR3855-Hylocereus_undulatus_Britt-12-RNAseq_result\4_Function\2_Group_Diff_Function\UP_DOWN\GO\NL-VS-L1.P.html#gene113) | electron transport chain | 11 (0.89%) | 22 (0.65%) | 0.142075 | 0.999992 |
| 114 | [GO:0009698](file:///E:\2018-7-3%E7%81%AB%E9%BE%99%E6%9E%9C%E8%BD%AC%E5%BD%95%E7%BB%84%E6%B5%8B%E5%BA%8F\%E5%AE%8C%E6%95%B4%E7%89%88%E6%95%B0%E6%8D%AE\GDR3855-Hylocereus_undulatus_Britt-12-RNAseq_result\4_Function\2_Group_Diff_Function\UP_DOWN\GO\NL-VS-L1.P.html#gene114) | phenylpropanoid metabolic process | 8 (0.65%) | 15 (0.45%) | 0.143186 | 0.999992 |
| 115 | [GO:0000272](file:///E:\2018-7-3%E7%81%AB%E9%BE%99%E6%9E%9C%E8%BD%AC%E5%BD%95%E7%BB%84%E6%B5%8B%E5%BA%8F\%E5%AE%8C%E6%95%B4%E7%89%88%E6%95%B0%E6%8D%AE\GDR3855-Hylocereus_undulatus_Britt-12-RNAseq_result\4_Function\2_Group_Diff_Function\UP_DOWN\GO\NL-VS-L1.P.html#gene115) | polysaccharide catabolic process | 3 (0.24%) | 4 (0.12%) | 0.143543 | 0.999992 |
| 116 | [GO:0009415](file:///E:\2018-7-3%E7%81%AB%E9%BE%99%E6%9E%9C%E8%BD%AC%E5%BD%95%E7%BB%84%E6%B5%8B%E5%BA%8F\%E5%AE%8C%E6%95%B4%E7%89%88%E6%95%B0%E6%8D%AE\GDR3855-Hylocereus_undulatus_Britt-12-RNAseq_result\4_Function\2_Group_Diff_Function\UP_DOWN\GO\NL-VS-L1.P.html#gene116) | response to water | 3 (0.24%) | 4 (0.12%) | 0.143543 | 0.999992 |
| 117 | [GO:0009696](file:///E:\2018-7-3%E7%81%AB%E9%BE%99%E6%9E%9C%E8%BD%AC%E5%BD%95%E7%BB%84%E6%B5%8B%E5%BA%8F\%E5%AE%8C%E6%95%B4%E7%89%88%E6%95%B0%E6%8D%AE\GDR3855-Hylocereus_undulatus_Britt-12-RNAseq_result\4_Function\2_Group_Diff_Function\UP_DOWN\GO\NL-VS-L1.P.html#gene117) | salicylic acid metabolic process | 3 (0.24%) | 4 (0.12%) | 0.143543 | 0.999992 |
| 118 | [GO:0009733](file:///E:\2018-7-3%E7%81%AB%E9%BE%99%E6%9E%9C%E8%BD%AC%E5%BD%95%E7%BB%84%E6%B5%8B%E5%BA%8F\%E5%AE%8C%E6%95%B4%E7%89%88%E6%95%B0%E6%8D%AE\GDR3855-Hylocereus_undulatus_Britt-12-RNAseq_result\4_Function\2_Group_Diff_Function\UP_DOWN\GO\NL-VS-L1.P.html#gene118) | response to auxin | 3 (0.24%) | 4 (0.12%) | 0.143543 | 0.999992 |
| 119 | [GO:0018958](file:///E:\2018-7-3%E7%81%AB%E9%BE%99%E6%9E%9C%E8%BD%AC%E5%BD%95%E7%BB%84%E6%B5%8B%E5%BA%8F\%E5%AE%8C%E6%95%B4%E7%89%88%E6%95%B0%E6%8D%AE\GDR3855-Hylocereus_undulatus_Britt-12-RNAseq_result\4_Function\2_Group_Diff_Function\UP_DOWN\GO\NL-VS-L1.P.html#gene119) | phenol-containing compound metabolic process | 3 (0.24%) | 4 (0.12%) | 0.143543 | 0.999992 |
| 120 | [GO:0042537](file:///E:\2018-7-3%E7%81%AB%E9%BE%99%E6%9E%9C%E8%BD%AC%E5%BD%95%E7%BB%84%E6%B5%8B%E5%BA%8F\%E5%AE%8C%E6%95%B4%E7%89%88%E6%95%B0%E6%8D%AE\GDR3855-Hylocereus_undulatus_Britt-12-RNAseq_result\4_Function\2_Group_Diff_Function\UP_DOWN\GO\NL-VS-L1.P.html#gene120) | benzene-containing compound metabolic process | 3 (0.24%) | 4 (0.12%) | 0.143543 | 0.999992 |
| 121 | [GO:0048646](file:///E:\2018-7-3%E7%81%AB%E9%BE%99%E6%9E%9C%E8%BD%AC%E5%BD%95%E7%BB%84%E6%B5%8B%E5%BA%8F\%E5%AE%8C%E6%95%B4%E7%89%88%E6%95%B0%E6%8D%AE\GDR3855-Hylocereus_undulatus_Britt-12-RNAseq_result\4_Function\2_Group_Diff_Function\UP_DOWN\GO\NL-VS-L1.P.html#gene121) | anatomical structure formation involved in morphogenesis | 3 (0.24%) | 4 (0.12%) | 0.143543 | 0.999992 |
| 122 | [GO:1990066](file:///E:\2018-7-3%E7%81%AB%E9%BE%99%E6%9E%9C%E8%BD%AC%E5%BD%95%E7%BB%84%E6%B5%8B%E5%BA%8F\%E5%AE%8C%E6%95%B4%E7%89%88%E6%95%B0%E6%8D%AE\GDR3855-Hylocereus_undulatus_Britt-12-RNAseq_result\4_Function\2_Group_Diff_Function\UP_DOWN\GO\NL-VS-L1.P.html#gene122) | energy quenching | 3 (0.24%) | 4 (0.12%) | 0.143543 | 0.999992 |
| 123 | [GO:0006325](file:///E:\2018-7-3%E7%81%AB%E9%BE%99%E6%9E%9C%E8%BD%AC%E5%BD%95%E7%BB%84%E6%B5%8B%E5%BA%8F\%E5%AE%8C%E6%95%B4%E7%89%88%E6%95%B0%E6%8D%AE\GDR3855-Hylocereus_undulatus_Britt-12-RNAseq_result\4_Function\2_Group_Diff_Function\UP_DOWN\GO\NL-VS-L1.P.html#gene123) | chromatin organization | 22 (1.78%) | 49 (1.46%) | 0.148308 | 0.999992 |
| 124 | [GO:0015711](file:///E:\2018-7-3%E7%81%AB%E9%BE%99%E6%9E%9C%E8%BD%AC%E5%BD%95%E7%BB%84%E6%B5%8B%E5%BA%8F\%E5%AE%8C%E6%95%B4%E7%89%88%E6%95%B0%E6%8D%AE\GDR3855-Hylocereus_undulatus_Britt-12-RNAseq_result\4_Function\2_Group_Diff_Function\UP_DOWN\GO\NL-VS-L1.P.html#gene124) | organic anion transport | 10 (0.81%) | 20 (0.6%) | 0.158197 | 0.999992 |
| 125 | [GO:0043414](file:///E:\2018-7-3%E7%81%AB%E9%BE%99%E6%9E%9C%E8%BD%AC%E5%BD%95%E7%BB%84%E6%B5%8B%E5%BA%8F\%E5%AE%8C%E6%95%B4%E7%89%88%E6%95%B0%E6%8D%AE\GDR3855-Hylocereus_undulatus_Britt-12-RNAseq_result\4_Function\2_Group_Diff_Function\UP_DOWN\GO\NL-VS-L1.P.html#gene125) | macromolecule methylation | 10 (0.81%) | 20 (0.6%) | 0.158197 | 0.999992 |
| 126 | [GO:0033993](file:///E:\2018-7-3%E7%81%AB%E9%BE%99%E6%9E%9C%E8%BD%AC%E5%BD%95%E7%BB%84%E6%B5%8B%E5%BA%8F\%E5%AE%8C%E6%95%B4%E7%89%88%E6%95%B0%E6%8D%AE\GDR3855-Hylocereus_undulatus_Britt-12-RNAseq_result\4_Function\2_Group_Diff_Function\UP_DOWN\GO\NL-VS-L1.P.html#gene126) | response to lipid | 7 (0.57%) | 13 (0.39%) | 0.159930 | 0.999992 |
| 127 | [GO:0043436](file:///E:\2018-7-3%E7%81%AB%E9%BE%99%E6%9E%9C%E8%BD%AC%E5%BD%95%E7%BB%84%E6%B5%8B%E5%BA%8F\%E5%AE%8C%E6%95%B4%E7%89%88%E6%95%B0%E6%8D%AE\GDR3855-Hylocereus_undulatus_Britt-12-RNAseq_result\4_Function\2_Group_Diff_Function\UP_DOWN\GO\NL-VS-L1.P.html#gene127) | oxoacid metabolic process | 78 (6.32%) | 194 (5.78%) | 0.169446 | 0.999992 |
| 128 | [GO:0006811](file:///E:\2018-7-3%E7%81%AB%E9%BE%99%E6%9E%9C%E8%BD%AC%E5%BD%95%E7%BB%84%E6%B5%8B%E5%BA%8F\%E5%AE%8C%E6%95%B4%E7%89%88%E6%95%B0%E6%8D%AE\GDR3855-Hylocereus_undulatus_Britt-12-RNAseq_result\4_Function\2_Group_Diff_Function\UP_DOWN\GO\NL-VS-L1.P.html#gene128) | ion transport | 71 (5.75%) | 176 (5.24%) | 0.173809 | 0.999992 |
| 129 | [GO:0009267](file:///E:\2018-7-3%E7%81%AB%E9%BE%99%E6%9E%9C%E8%BD%AC%E5%BD%95%E7%BB%84%E6%B5%8B%E5%BA%8F\%E5%AE%8C%E6%95%B4%E7%89%88%E6%95%B0%E6%8D%AE\GDR3855-Hylocereus_undulatus_Britt-12-RNAseq_result\4_Function\2_Group_Diff_Function\UP_DOWN\GO\NL-VS-L1.P.html#gene129) | cellular response to starvation | 9 (0.73%) | 18 (0.54%) | 0.176654 | 0.999992 |
| 130 | [GO:0042594](file:///E:\2018-7-3%E7%81%AB%E9%BE%99%E6%9E%9C%E8%BD%AC%E5%BD%95%E7%BB%84%E6%B5%8B%E5%BA%8F\%E5%AE%8C%E6%95%B4%E7%89%88%E6%95%B0%E6%8D%AE\GDR3855-Hylocereus_undulatus_Britt-12-RNAseq_result\4_Function\2_Group_Diff_Function\UP_DOWN\GO\NL-VS-L1.P.html#gene130) | response to starvation | 9 (0.73%) | 18 (0.54%) | 0.176654 | 0.999992 |
| 131 | [GO:0043933](file:///E:\2018-7-3%E7%81%AB%E9%BE%99%E6%9E%9C%E8%BD%AC%E5%BD%95%E7%BB%84%E6%B5%8B%E5%BA%8F\%E5%AE%8C%E6%95%B4%E7%89%88%E6%95%B0%E6%8D%AE\GDR3855-Hylocereus_undulatus_Britt-12-RNAseq_result\4_Function\2_Group_Diff_Function\UP_DOWN\GO\NL-VS-L1.P.html#gene131) | macromolecular complex subunit organization | 42 (3.4%) | 101 (3.01%) | 0.178135 | 0.999992 |
| 132 | [GO:0009914](file:///E:\2018-7-3%E7%81%AB%E9%BE%99%E6%9E%9C%E8%BD%AC%E5%BD%95%E7%BB%84%E6%B5%8B%E5%BA%8F\%E5%AE%8C%E6%95%B4%E7%89%88%E6%95%B0%E6%8D%AE\GDR3855-Hylocereus_undulatus_Britt-12-RNAseq_result\4_Function\2_Group_Diff_Function\UP_DOWN\GO\NL-VS-L1.P.html#gene132) | hormone transport | 6 (0.49%) | 11 (0.33%) | 0.179320 | 0.999992 |
| 133 | [GO:0060918](file:///E:\2018-7-3%E7%81%AB%E9%BE%99%E6%9E%9C%E8%BD%AC%E5%BD%95%E7%BB%84%E6%B5%8B%E5%BA%8F\%E5%AE%8C%E6%95%B4%E7%89%88%E6%95%B0%E6%8D%AE\GDR3855-Hylocereus_undulatus_Britt-12-RNAseq_result\4_Function\2_Group_Diff_Function\UP_DOWN\GO\NL-VS-L1.P.html#gene133) | auxin transport | 6 (0.49%) | 11 (0.33%) | 0.179320 | 0.999992 |
| 134 | [GO:0044723](file:///E:\2018-7-3%E7%81%AB%E9%BE%99%E6%9E%9C%E8%BD%AC%E5%BD%95%E7%BB%84%E6%B5%8B%E5%BA%8F\%E5%AE%8C%E6%95%B4%E7%89%88%E6%95%B0%E6%8D%AE\GDR3855-Hylocereus_undulatus_Britt-12-RNAseq_result\4_Function\2_Group_Diff_Function\UP_DOWN\GO\NL-VS-L1.P.html#gene134) | single-organism carbohydrate metabolic process | 33 (2.67%) | 78 (2.32%) | 0.179998 | 0.999992 |
| 135 | [GO:0006082](file:///E:\2018-7-3%E7%81%AB%E9%BE%99%E6%9E%9C%E8%BD%AC%E5%BD%95%E7%BB%84%E6%B5%8B%E5%BA%8F\%E5%AE%8C%E6%95%B4%E7%89%88%E6%95%B0%E6%8D%AE\GDR3855-Hylocereus_undulatus_Britt-12-RNAseq_result\4_Function\2_Group_Diff_Function\UP_DOWN\GO\NL-VS-L1.P.html#gene135) | organic acid metabolic process | 78 (6.32%) | 195 (5.81%) | 0.184478 | 0.999992 |
| 136 | [GO:0098655](file:///E:\2018-7-3%E7%81%AB%E9%BE%99%E6%9E%9C%E8%BD%AC%E5%BD%95%E7%BB%84%E6%B5%8B%E5%BA%8F\%E5%AE%8C%E6%95%B4%E7%89%88%E6%95%B0%E6%8D%AE\GDR3855-Hylocereus_undulatus_Britt-12-RNAseq_result\4_Function\2_Group_Diff_Function\UP_DOWN\GO\NL-VS-L1.P.html#gene136) | cation transmembrane transport | 11 (0.89%) | 23 (0.68%) | 0.185774 | 0.999992 |
| 137 | [GO:0098660](file:///E:\2018-7-3%E7%81%AB%E9%BE%99%E6%9E%9C%E8%BD%AC%E5%BD%95%E7%BB%84%E6%B5%8B%E5%BA%8F\%E5%AE%8C%E6%95%B4%E7%89%88%E6%95%B0%E6%8D%AE\GDR3855-Hylocereus_undulatus_Britt-12-RNAseq_result\4_Function\2_Group_Diff_Function\UP_DOWN\GO\NL-VS-L1.P.html#gene137) | inorganic ion transmembrane transport | 11 (0.89%) | 23 (0.68%) | 0.185774 | 0.999992 |
| 138 | [GO:0098662](file:///E:\2018-7-3%E7%81%AB%E9%BE%99%E6%9E%9C%E8%BD%AC%E5%BD%95%E7%BB%84%E6%B5%8B%E5%BA%8F\%E5%AE%8C%E6%95%B4%E7%89%88%E6%95%B0%E6%8D%AE\GDR3855-Hylocereus_undulatus_Britt-12-RNAseq_result\4_Function\2_Group_Diff_Function\UP_DOWN\GO\NL-VS-L1.P.html#gene138) | inorganic cation transmembrane transport | 11 (0.89%) | 23 (0.68%) | 0.185774 | 0.999992 |
| 139 | [GO:0009605](file:///E:\2018-7-3%E7%81%AB%E9%BE%99%E6%9E%9C%E8%BD%AC%E5%BD%95%E7%BB%84%E6%B5%8B%E5%BA%8F\%E5%AE%8C%E6%95%B4%E7%89%88%E6%95%B0%E6%8D%AE\GDR3855-Hylocereus_undulatus_Britt-12-RNAseq_result\4_Function\2_Group_Diff_Function\UP_DOWN\GO\NL-VS-L1.P.html#gene139) | response to external stimulus | 43 (3.48%) | 104 (3.1%) | 0.187031 | 0.999992 |
| 140 | [GO:0051707](file:///E:\2018-7-3%E7%81%AB%E9%BE%99%E6%9E%9C%E8%BD%AC%E5%BD%95%E7%BB%84%E6%B5%8B%E5%BA%8F\%E5%AE%8C%E6%95%B4%E7%89%88%E6%95%B0%E6%8D%AE\GDR3855-Hylocereus_undulatus_Britt-12-RNAseq_result\4_Function\2_Group_Diff_Function\UP_DOWN\GO\NL-VS-L1.P.html#gene140) | response to other organism | 27 (2.19%) | 63 (1.88%) | 0.187374 | 0.999992 |
| 141 | [GO:1901700](file:///E:\2018-7-3%E7%81%AB%E9%BE%99%E6%9E%9C%E8%BD%AC%E5%BD%95%E7%BB%84%E6%B5%8B%E5%BA%8F\%E5%AE%8C%E6%95%B4%E7%89%88%E6%95%B0%E6%8D%AE\GDR3855-Hylocereus_undulatus_Britt-12-RNAseq_result\4_Function\2_Group_Diff_Function\UP_DOWN\GO\NL-VS-L1.P.html#gene141) | response to oxygen-containing compound | 27 (2.19%) | 63 (1.88%) | 0.187374 | 0.999992 |
| 142 | [GO:0044042](file:///E:\2018-7-3%E7%81%AB%E9%BE%99%E6%9E%9C%E8%BD%AC%E5%BD%95%E7%BB%84%E6%B5%8B%E5%BA%8F\%E5%AE%8C%E6%95%B4%E7%89%88%E6%95%B0%E6%8D%AE\GDR3855-Hylocereus_undulatus_Britt-12-RNAseq_result\4_Function\2_Group_Diff_Function\UP_DOWN\GO\NL-VS-L1.P.html#gene142) | glucan metabolic process | 23 (1.86%) | 53 (1.58%) | 0.191370 | 0.999992 |
| 143 | [GO:0044283](file:///E:\2018-7-3%E7%81%AB%E9%BE%99%E6%9E%9C%E8%BD%AC%E5%BD%95%E7%BB%84%E6%B5%8B%E5%BA%8F\%E5%AE%8C%E6%95%B4%E7%89%88%E6%95%B0%E6%8D%AE\GDR3855-Hylocereus_undulatus_Britt-12-RNAseq_result\4_Function\2_Group_Diff_Function\UP_DOWN\GO\NL-VS-L1.P.html#gene143) | small molecule biosynthetic process | 23 (1.86%) | 53 (1.58%) | 0.191370 | 0.999992 |
| 144 | [GO:0030001](file:///E:\2018-7-3%E7%81%AB%E9%BE%99%E6%9E%9C%E8%BD%AC%E5%BD%95%E7%BB%84%E6%B5%8B%E5%BA%8F\%E5%AE%8C%E6%95%B4%E7%89%88%E6%95%B0%E6%8D%AE\GDR3855-Hylocereus_undulatus_Britt-12-RNAseq_result\4_Function\2_Group_Diff_Function\UP_DOWN\GO\NL-VS-L1.P.html#gene144) | metal ion transport | 30 (2.43%) | 71 (2.11%) | 0.196860 | 0.999992 |
| 145 | [GO:0019752](file:///E:\2018-7-3%E7%81%AB%E9%BE%99%E6%9E%9C%E8%BD%AC%E5%BD%95%E7%BB%84%E6%B5%8B%E5%BA%8F\%E5%AE%8C%E6%95%B4%E7%89%88%E6%95%B0%E6%8D%AE\GDR3855-Hylocereus_undulatus_Britt-12-RNAseq_result\4_Function\2_Group_Diff_Function\UP_DOWN\GO\NL-VS-L1.P.html#gene145) | carboxylic acid metabolic process | 75 (6.08%) | 188 (5.6%) | 0.198273 | 0.999992 |
| 146 | [GO:0016571](file:///E:\2018-7-3%E7%81%AB%E9%BE%99%E6%9E%9C%E8%BD%AC%E5%BD%95%E7%BB%84%E6%B5%8B%E5%BA%8F\%E5%AE%8C%E6%95%B4%E7%89%88%E6%95%B0%E6%8D%AE\GDR3855-Hylocereus_undulatus_Britt-12-RNAseq_result\4_Function\2_Group_Diff_Function\UP_DOWN\GO\NL-VS-L1.P.html#gene146) | histone methylation | 5 (0.41%) | 9 (0.27%) | 0.202064 | 0.999992 |
| 147 | [GO:0018904](file:///E:\2018-7-3%E7%81%AB%E9%BE%99%E6%9E%9C%E8%BD%AC%E5%BD%95%E7%BB%84%E6%B5%8B%E5%BA%8F\%E5%AE%8C%E6%95%B4%E7%89%88%E6%95%B0%E6%8D%AE\GDR3855-Hylocereus_undulatus_Britt-12-RNAseq_result\4_Function\2_Group_Diff_Function\UP_DOWN\GO\NL-VS-L1.P.html#gene147) | ether metabolic process | 5 (0.41%) | 9 (0.27%) | 0.202064 | 0.999992 |
| 148 | [GO:0030029](file:///E:\2018-7-3%E7%81%AB%E9%BE%99%E6%9E%9C%E8%BD%AC%E5%BD%95%E7%BB%84%E6%B5%8B%E5%BA%8F\%E5%AE%8C%E6%95%B4%E7%89%88%E6%95%B0%E6%8D%AE\GDR3855-Hylocereus_undulatus_Britt-12-RNAseq_result\4_Function\2_Group_Diff_Function\UP_DOWN\GO\NL-VS-L1.P.html#gene148) | actin filament-based process | 5 (0.41%) | 9 (0.27%) | 0.202064 | 0.999992 |
| 149 | [GO:0030036](file:///E:\2018-7-3%E7%81%AB%E9%BE%99%E6%9E%9C%E8%BD%AC%E5%BD%95%E7%BB%84%E6%B5%8B%E5%BA%8F\%E5%AE%8C%E6%95%B4%E7%89%88%E6%95%B0%E6%8D%AE\GDR3855-Hylocereus_undulatus_Britt-12-RNAseq_result\4_Function\2_Group_Diff_Function\UP_DOWN\GO\NL-VS-L1.P.html#gene149) | actin cytoskeleton organization | 5 (0.41%) | 9 (0.27%) | 0.202064 | 0.999992 |
| 150 | [GO:0009607](file:///E:\2018-7-3%E7%81%AB%E9%BE%99%E6%9E%9C%E8%BD%AC%E5%BD%95%E7%BB%84%E6%B5%8B%E5%BA%8F\%E5%AE%8C%E6%95%B4%E7%89%88%E6%95%B0%E6%8D%AE\GDR3855-Hylocereus_undulatus_Britt-12-RNAseq_result\4_Function\2_Group_Diff_Function\UP_DOWN\GO\NL-VS-L1.P.html#gene150) | response to biotic stimulus | 27 (2.19%) | 64 (1.91%) | 0.215833 | 0.999992 |
| 151 | [GO:0043207](file:///E:\2018-7-3%E7%81%AB%E9%BE%99%E6%9E%9C%E8%BD%AC%E5%BD%95%E7%BB%84%E6%B5%8B%E5%BA%8F\%E5%AE%8C%E6%95%B4%E7%89%88%E6%95%B0%E6%8D%AE\GDR3855-Hylocereus_undulatus_Britt-12-RNAseq_result\4_Function\2_Group_Diff_Function\UP_DOWN\GO\NL-VS-L1.P.html#gene151) | response to external biotic stimulus | 27 (2.19%) | 64 (1.91%) | 0.215833 | 0.999992 |
| 152 | [GO:1902589](file:///E:\2018-7-3%E7%81%AB%E9%BE%99%E6%9E%9C%E8%BD%AC%E5%BD%95%E7%BB%84%E6%B5%8B%E5%BA%8F\%E5%AE%8C%E6%95%B4%E7%89%88%E6%95%B0%E6%8D%AE\GDR3855-Hylocereus_undulatus_Britt-12-RNAseq_result\4_Function\2_Group_Diff_Function\UP_DOWN\GO\NL-VS-L1.P.html#gene152) | single-organism organelle organization | 23 (1.86%) | 54 (1.61%) | 0.222928 | 0.999992 |
| 153 | [GO:0033043](file:///E:\2018-7-3%E7%81%AB%E9%BE%99%E6%9E%9C%E8%BD%AC%E5%BD%95%E7%BB%84%E6%B5%8B%E5%BA%8F\%E5%AE%8C%E6%95%B4%E7%89%88%E6%95%B0%E6%8D%AE\GDR3855-Hylocereus_undulatus_Britt-12-RNAseq_result\4_Function\2_Group_Diff_Function\UP_DOWN\GO\NL-VS-L1.P.html#gene153) | regulation of organelle organization | 4 (0.32%) | 7 (0.21%) | 0.229232 | 0.999992 |
| 154 | [GO:0048569](file:///E:\2018-7-3%E7%81%AB%E9%BE%99%E6%9E%9C%E8%BD%AC%E5%BD%95%E7%BB%84%E6%B5%8B%E5%BA%8F\%E5%AE%8C%E6%95%B4%E7%89%88%E6%95%B0%E6%8D%AE\GDR3855-Hylocereus_undulatus_Britt-12-RNAseq_result\4_Function\2_Group_Diff_Function\UP_DOWN\GO\NL-VS-L1.P.html#gene154) | post-embryonic organ development | 4 (0.32%) | 7 (0.21%) | 0.229232 | 0.999992 |
| 155 | [GO:0016310](file:///E:\2018-7-3%E7%81%AB%E9%BE%99%E6%9E%9C%E8%BD%AC%E5%BD%95%E7%BB%84%E6%B5%8B%E5%BA%8F\%E5%AE%8C%E6%95%B4%E7%89%88%E6%95%B0%E6%8D%AE\GDR3855-Hylocereus_undulatus_Britt-12-RNAseq_result\4_Function\2_Group_Diff_Function\UP_DOWN\GO\NL-VS-L1.P.html#gene155) | phosphorylation | 43 (3.48%) | 106 (3.16%) | 0.231982 | 0.999992 |
| 156 | [GO:0043623](file:///E:\2018-7-3%E7%81%AB%E9%BE%99%E6%9E%9C%E8%BD%AC%E5%BD%95%E7%BB%84%E6%B5%8B%E5%BA%8F\%E5%AE%8C%E6%95%B4%E7%89%88%E6%95%B0%E6%8D%AE\GDR3855-Hylocereus_undulatus_Britt-12-RNAseq_result\4_Function\2_Group_Diff_Function\UP_DOWN\GO\NL-VS-L1.P.html#gene156) | cellular protein complex assembly | 17 (1.38%) | 39 (1.16%) | 0.232053 | 0.999992 |
| 157 | [GO:0000096](file:///E:\2018-7-3%E7%81%AB%E9%BE%99%E6%9E%9C%E8%BD%AC%E5%BD%95%E7%BB%84%E6%B5%8B%E5%BA%8F\%E5%AE%8C%E6%95%B4%E7%89%88%E6%95%B0%E6%8D%AE\GDR3855-Hylocereus_undulatus_Britt-12-RNAseq_result\4_Function\2_Group_Diff_Function\UP_DOWN\GO\NL-VS-L1.P.html#gene157) | sulfur amino acid metabolic process | 13 (1.05%) | 29 (0.86%) | 0.235242 | 0.999992 |
| 158 | [GO:0006073](file:///E:\2018-7-3%E7%81%AB%E9%BE%99%E6%9E%9C%E8%BD%AC%E5%BD%95%E7%BB%84%E6%B5%8B%E5%BA%8F\%E5%AE%8C%E6%95%B4%E7%89%88%E6%95%B0%E6%8D%AE\GDR3855-Hylocereus_undulatus_Britt-12-RNAseq_result\4_Function\2_Group_Diff_Function\UP_DOWN\GO\NL-VS-L1.P.html#gene158) | cellular glucan metabolic process | 20 (1.62%) | 47 (1.4%) | 0.246013 | 0.999992 |
| 159 | [GO:0000278](file:///E:\2018-7-3%E7%81%AB%E9%BE%99%E6%9E%9C%E8%BD%AC%E5%BD%95%E7%BB%84%E6%B5%8B%E5%BA%8F\%E5%AE%8C%E6%95%B4%E7%89%88%E6%95%B0%E6%8D%AE\GDR3855-Hylocereus_undulatus_Britt-12-RNAseq_result\4_Function\2_Group_Diff_Function\UP_DOWN\GO\NL-VS-L1.P.html#gene159) | mitotic cell cycle | 6 (0.49%) | 12 (0.36%) | 0.252157 | 0.999992 |
| 160 | [GO:0006633](file:///E:\2018-7-3%E7%81%AB%E9%BE%99%E6%9E%9C%E8%BD%AC%E5%BD%95%E7%BB%84%E6%B5%8B%E5%BA%8F\%E5%AE%8C%E6%95%B4%E7%89%88%E6%95%B0%E6%8D%AE\GDR3855-Hylocereus_undulatus_Britt-12-RNAseq_result\4_Function\2_Group_Diff_Function\UP_DOWN\GO\NL-VS-L1.P.html#gene160) | fatty acid biosynthetic process | 3 (0.24%) | 5 (0.15%) | 0.262612 | 0.999992 |
| 161 | [GO:0007264](file:///E:\2018-7-3%E7%81%AB%E9%BE%99%E6%9E%9C%E8%BD%AC%E5%BD%95%E7%BB%84%E6%B5%8B%E5%BA%8F\%E5%AE%8C%E6%95%B4%E7%89%88%E6%95%B0%E6%8D%AE\GDR3855-Hylocereus_undulatus_Britt-12-RNAseq_result\4_Function\2_Group_Diff_Function\UP_DOWN\GO\NL-VS-L1.P.html#gene161) | small GTPase mediated signal transduction | 3 (0.24%) | 5 (0.15%) | 0.262612 | 0.999992 |
| 162 | [GO:0007265](file:///E:\2018-7-3%E7%81%AB%E9%BE%99%E6%9E%9C%E8%BD%AC%E5%BD%95%E7%BB%84%E6%B5%8B%E5%BA%8F\%E5%AE%8C%E6%95%B4%E7%89%88%E6%95%B0%E6%8D%AE\GDR3855-Hylocereus_undulatus_Britt-12-RNAseq_result\4_Function\2_Group_Diff_Function\UP_DOWN\GO\NL-VS-L1.P.html#gene162) | Ras protein signal transduction | 3 (0.24%) | 5 (0.15%) | 0.262612 | 0.999992 |
| 163 | [GO:0008272](file:///E:\2018-7-3%E7%81%AB%E9%BE%99%E6%9E%9C%E8%BD%AC%E5%BD%95%E7%BB%84%E6%B5%8B%E5%BA%8F\%E5%AE%8C%E6%95%B4%E7%89%88%E6%95%B0%E6%8D%AE\GDR3855-Hylocereus_undulatus_Britt-12-RNAseq_result\4_Function\2_Group_Diff_Function\UP_DOWN\GO\NL-VS-L1.P.html#gene163) | sulfate transport | 3 (0.24%) | 5 (0.15%) | 0.262612 | 0.999992 |
| 164 | [GO:0009694](file:///E:\2018-7-3%E7%81%AB%E9%BE%99%E6%9E%9C%E8%BD%AC%E5%BD%95%E7%BB%84%E6%B5%8B%E5%BA%8F\%E5%AE%8C%E6%95%B4%E7%89%88%E6%95%B0%E6%8D%AE\GDR3855-Hylocereus_undulatus_Britt-12-RNAseq_result\4_Function\2_Group_Diff_Function\UP_DOWN\GO\NL-VS-L1.P.html#gene164) | jasmonic acid metabolic process | 3 (0.24%) | 5 (0.15%) | 0.262612 | 0.999992 |
| 165 | [GO:0016072](file:///E:\2018-7-3%E7%81%AB%E9%BE%99%E6%9E%9C%E8%BD%AC%E5%BD%95%E7%BB%84%E6%B5%8B%E5%BA%8F\%E5%AE%8C%E6%95%B4%E7%89%88%E6%95%B0%E6%8D%AE\GDR3855-Hylocereus_undulatus_Britt-12-RNAseq_result\4_Function\2_Group_Diff_Function\UP_DOWN\GO\NL-VS-L1.P.html#gene165) | rRNA metabolic process | 3 (0.24%) | 5 (0.15%) | 0.262612 | 0.999992 |
| 166 | [GO:0019321](file:///E:\2018-7-3%E7%81%AB%E9%BE%99%E6%9E%9C%E8%BD%AC%E5%BD%95%E7%BB%84%E6%B5%8B%E5%BA%8F\%E5%AE%8C%E6%95%B4%E7%89%88%E6%95%B0%E6%8D%AE\GDR3855-Hylocereus_undulatus_Britt-12-RNAseq_result\4_Function\2_Group_Diff_Function\UP_DOWN\GO\NL-VS-L1.P.html#gene166) | pentose metabolic process | 3 (0.24%) | 5 (0.15%) | 0.262612 | 0.999992 |
| 167 | [GO:0032011](file:///E:\2018-7-3%E7%81%AB%E9%BE%99%E6%9E%9C%E8%BD%AC%E5%BD%95%E7%BB%84%E6%B5%8B%E5%BA%8F\%E5%AE%8C%E6%95%B4%E7%89%88%E6%95%B0%E6%8D%AE\GDR3855-Hylocereus_undulatus_Britt-12-RNAseq_result\4_Function\2_Group_Diff_Function\UP_DOWN\GO\NL-VS-L1.P.html#gene167) | ARF protein signal transduction | 3 (0.24%) | 5 (0.15%) | 0.262612 | 0.999992 |
| 168 | [GO:0032507](file:///E:\2018-7-3%E7%81%AB%E9%BE%99%E6%9E%9C%E8%BD%AC%E5%BD%95%E7%BB%84%E6%B5%8B%E5%BA%8F\%E5%AE%8C%E6%95%B4%E7%89%88%E6%95%B0%E6%8D%AE\GDR3855-Hylocereus_undulatus_Britt-12-RNAseq_result\4_Function\2_Group_Diff_Function\UP_DOWN\GO\NL-VS-L1.P.html#gene168) | maintenance of protein location in cell | 3 (0.24%) | 5 (0.15%) | 0.262612 | 0.999992 |
| 169 | [GO:0045185](file:///E:\2018-7-3%E7%81%AB%E9%BE%99%E6%9E%9C%E8%BD%AC%E5%BD%95%E7%BB%84%E6%B5%8B%E5%BA%8F\%E5%AE%8C%E6%95%B4%E7%89%88%E6%95%B0%E6%8D%AE\GDR3855-Hylocereus_undulatus_Britt-12-RNAseq_result\4_Function\2_Group_Diff_Function\UP_DOWN\GO\NL-VS-L1.P.html#gene169) | maintenance of protein location | 3 (0.24%) | 5 (0.15%) | 0.262612 | 0.999992 |
| 170 | [GO:0051220](file:///E:\2018-7-3%E7%81%AB%E9%BE%99%E6%9E%9C%E8%BD%AC%E5%BD%95%E7%BB%84%E6%B5%8B%E5%BA%8F\%E5%AE%8C%E6%95%B4%E7%89%88%E6%95%B0%E6%8D%AE\GDR3855-Hylocereus_undulatus_Britt-12-RNAseq_result\4_Function\2_Group_Diff_Function\UP_DOWN\GO\NL-VS-L1.P.html#gene170) | cytoplasmic sequestering of protein | 3 (0.24%) | 5 (0.15%) | 0.262612 | 0.999992 |
| 171 | [GO:0051235](file:///E:\2018-7-3%E7%81%AB%E9%BE%99%E6%9E%9C%E8%BD%AC%E5%BD%95%E7%BB%84%E6%B5%8B%E5%BA%8F\%E5%AE%8C%E6%95%B4%E7%89%88%E6%95%B0%E6%8D%AE\GDR3855-Hylocereus_undulatus_Britt-12-RNAseq_result\4_Function\2_Group_Diff_Function\UP_DOWN\GO\NL-VS-L1.P.html#gene171) | maintenance of location | 3 (0.24%) | 5 (0.15%) | 0.262612 | 0.999992 |
| 172 | [GO:0051651](file:///E:\2018-7-3%E7%81%AB%E9%BE%99%E6%9E%9C%E8%BD%AC%E5%BD%95%E7%BB%84%E6%B5%8B%E5%BA%8F\%E5%AE%8C%E6%95%B4%E7%89%88%E6%95%B0%E6%8D%AE\GDR3855-Hylocereus_undulatus_Britt-12-RNAseq_result\4_Function\2_Group_Diff_Function\UP_DOWN\GO\NL-VS-L1.P.html#gene172) | maintenance of location in cell | 3 (0.24%) | 5 (0.15%) | 0.262612 | 0.999992 |
| 173 | [GO:0072330](file:///E:\2018-7-3%E7%81%AB%E9%BE%99%E6%9E%9C%E8%BD%AC%E5%BD%95%E7%BB%84%E6%B5%8B%E5%BA%8F\%E5%AE%8C%E6%95%B4%E7%89%88%E6%95%B0%E6%8D%AE\GDR3855-Hylocereus_undulatus_Britt-12-RNAseq_result\4_Function\2_Group_Diff_Function\UP_DOWN\GO\NL-VS-L1.P.html#gene173) | monocarboxylic acid biosynthetic process | 3 (0.24%) | 5 (0.15%) | 0.262612 | 0.999992 |
| 174 | [GO:0072348](file:///E:\2018-7-3%E7%81%AB%E9%BE%99%E6%9E%9C%E8%BD%AC%E5%BD%95%E7%BB%84%E6%B5%8B%E5%BA%8F\%E5%AE%8C%E6%95%B4%E7%89%88%E6%95%B0%E6%8D%AE\GDR3855-Hylocereus_undulatus_Britt-12-RNAseq_result\4_Function\2_Group_Diff_Function\UP_DOWN\GO\NL-VS-L1.P.html#gene174) | sulfur compound transport | 3 (0.24%) | 5 (0.15%) | 0.262612 | 0.999992 |
| 175 | [GO:0034622](file:///E:\2018-7-3%E7%81%AB%E9%BE%99%E6%9E%9C%E8%BD%AC%E5%BD%95%E7%BB%84%E6%B5%8B%E5%BA%8F\%E5%AE%8C%E6%95%B4%E7%89%88%E6%95%B0%E6%8D%AE\GDR3855-Hylocereus_undulatus_Britt-12-RNAseq_result\4_Function\2_Group_Diff_Function\UP_DOWN\GO\NL-VS-L1.P.html#gene175) | cellular macromolecular complex assembly | 17 (1.38%) | 40 (1.19%) | 0.272882 | 0.999992 |
| 176 | [GO:0055114](file:///E:\2018-7-3%E7%81%AB%E9%BE%99%E6%9E%9C%E8%BD%AC%E5%BD%95%E7%BB%84%E6%B5%8B%E5%BA%8F\%E5%AE%8C%E6%95%B4%E7%89%88%E6%95%B0%E6%8D%AE\GDR3855-Hylocereus_undulatus_Britt-12-RNAseq_result\4_Function\2_Group_Diff_Function\UP_DOWN\GO\NL-VS-L1.P.html#gene176) | oxidation-reduction process | 27 (2.19%) | 66 (1.96%) | 0.278279 | 0.999992 |
| 177 | [GO:0071840](file:///E:\2018-7-3%E7%81%AB%E9%BE%99%E6%9E%9C%E8%BD%AC%E5%BD%95%E7%BB%84%E6%B5%8B%E5%BA%8F\%E5%AE%8C%E6%95%B4%E7%89%88%E6%95%B0%E6%8D%AE\GDR3855-Hylocereus_undulatus_Britt-12-RNAseq_result\4_Function\2_Group_Diff_Function\UP_DOWN\GO\NL-VS-L1.P.html#gene177) | cellular component organization or biogenesis | 103 (8.35%) | 267 (7.95%) | 0.278637 | 0.999992 |
| 178 | [GO:0009064](file:///E:\2018-7-3%E7%81%AB%E9%BE%99%E6%9E%9C%E8%BD%AC%E5%BD%95%E7%BB%84%E6%B5%8B%E5%BA%8F\%E5%AE%8C%E6%95%B4%E7%89%88%E6%95%B0%E6%8D%AE\GDR3855-Hylocereus_undulatus_Britt-12-RNAseq_result\4_Function\2_Group_Diff_Function\UP_DOWN\GO\NL-VS-L1.P.html#gene178) | glutamine family amino acid metabolic process | 5 (0.41%) | 10 (0.3%) | 0.287550 | 0.999992 |
| 179 | [GO:0009746](file:///E:\2018-7-3%E7%81%AB%E9%BE%99%E6%9E%9C%E8%BD%AC%E5%BD%95%E7%BB%84%E6%B5%8B%E5%BA%8F\%E5%AE%8C%E6%95%B4%E7%89%88%E6%95%B0%E6%8D%AE\GDR3855-Hylocereus_undulatus_Britt-12-RNAseq_result\4_Function\2_Group_Diff_Function\UP_DOWN\GO\NL-VS-L1.P.html#gene179) | response to hexose | 5 (0.41%) | 10 (0.3%) | 0.287550 | 0.999992 |
| 180 | [GO:0034284](file:///E:\2018-7-3%E7%81%AB%E9%BE%99%E6%9E%9C%E8%BD%AC%E5%BD%95%E7%BB%84%E6%B5%8B%E5%BA%8F\%E5%AE%8C%E6%95%B4%E7%89%88%E6%95%B0%E6%8D%AE\GDR3855-Hylocereus_undulatus_Britt-12-RNAseq_result\4_Function\2_Group_Diff_Function\UP_DOWN\GO\NL-VS-L1.P.html#gene180) | response to monosaccharide | 5 (0.41%) | 10 (0.3%) | 0.287550 | 0.999992 |
| 181 | [GO:0016570](file:///E:\2018-7-3%E7%81%AB%E9%BE%99%E6%9E%9C%E8%BD%AC%E5%BD%95%E7%BB%84%E6%B5%8B%E5%BA%8F\%E5%AE%8C%E6%95%B4%E7%89%88%E6%95%B0%E6%8D%AE\GDR3855-Hylocereus_undulatus_Britt-12-RNAseq_result\4_Function\2_Group_Diff_Function\UP_DOWN\GO\NL-VS-L1.P.html#gene181) | histone modification | 9 (0.73%) | 20 (0.6%) | 0.291380 | 0.999992 |
| 182 | [GO:0031668](file:///E:\2018-7-3%E7%81%AB%E9%BE%99%E6%9E%9C%E8%BD%AC%E5%BD%95%E7%BB%84%E6%B5%8B%E5%BA%8F\%E5%AE%8C%E6%95%B4%E7%89%88%E6%95%B0%E6%8D%AE\GDR3855-Hylocereus_undulatus_Britt-12-RNAseq_result\4_Function\2_Group_Diff_Function\UP_DOWN\GO\NL-VS-L1.P.html#gene182) | cellular response to extracellular stimulus | 9 (0.73%) | 20 (0.6%) | 0.291380 | 0.999992 |
| 183 | [GO:0031669](file:///E:\2018-7-3%E7%81%AB%E9%BE%99%E6%9E%9C%E8%BD%AC%E5%BD%95%E7%BB%84%E6%B5%8B%E5%BA%8F\%E5%AE%8C%E6%95%B4%E7%89%88%E6%95%B0%E6%8D%AE\GDR3855-Hylocereus_undulatus_Britt-12-RNAseq_result\4_Function\2_Group_Diff_Function\UP_DOWN\GO\NL-VS-L1.P.html#gene183) | cellular response to nutrient levels | 9 (0.73%) | 20 (0.6%) | 0.291380 | 0.999992 |
| 184 | [GO:0048229](file:///E:\2018-7-3%E7%81%AB%E9%BE%99%E6%9E%9C%E8%BD%AC%E5%BD%95%E7%BB%84%E6%B5%8B%E5%BA%8F\%E5%AE%8C%E6%95%B4%E7%89%88%E6%95%B0%E6%8D%AE\GDR3855-Hylocereus_undulatus_Britt-12-RNAseq_result\4_Function\2_Group_Diff_Function\UP_DOWN\GO\NL-VS-L1.P.html#gene184) | gametophyte development | 9 (0.73%) | 20 (0.6%) | 0.291380 | 0.999992 |
| 185 | [GO:0071496](file:///E:\2018-7-3%E7%81%AB%E9%BE%99%E6%9E%9C%E8%BD%AC%E5%BD%95%E7%BB%84%E6%B5%8B%E5%BA%8F\%E5%AE%8C%E6%95%B4%E7%89%88%E6%95%B0%E6%8D%AE\GDR3855-Hylocereus_undulatus_Britt-12-RNAseq_result\4_Function\2_Group_Diff_Function\UP_DOWN\GO\NL-VS-L1.P.html#gene185) | cellular response to external stimulus | 9 (0.73%) | 20 (0.6%) | 0.291380 | 0.999992 |
| 186 | [GO:0009743](file:///E:\2018-7-3%E7%81%AB%E9%BE%99%E6%9E%9C%E8%BD%AC%E5%BD%95%E7%BB%84%E6%B5%8B%E5%BA%8F\%E5%AE%8C%E6%95%B4%E7%89%88%E6%95%B0%E6%8D%AE\GDR3855-Hylocereus_undulatus_Britt-12-RNAseq_result\4_Function\2_Group_Diff_Function\UP_DOWN\GO\NL-VS-L1.P.html#gene186) | response to carbohydrate | 7 (0.57%) | 15 (0.45%) | 0.292444 | 0.999992 |
| 187 | [GO:0009416](file:///E:\2018-7-3%E7%81%AB%E9%BE%99%E6%9E%9C%E8%BD%AC%E5%BD%95%E7%BB%84%E6%B5%8B%E5%BA%8F\%E5%AE%8C%E6%95%B4%E7%89%88%E6%95%B0%E6%8D%AE\GDR3855-Hylocereus_undulatus_Britt-12-RNAseq_result\4_Function\2_Group_Diff_Function\UP_DOWN\GO\NL-VS-L1.P.html#gene187) | response to light stimulus | 34 (2.76%) | 85 (2.53%) | 0.299870 | 0.999992 |
| 188 | [GO:0006084](file:///E:\2018-7-3%E7%81%AB%E9%BE%99%E6%9E%9C%E8%BD%AC%E5%BD%95%E7%BB%84%E6%B5%8B%E5%BA%8F\%E5%AE%8C%E6%95%B4%E7%89%88%E6%95%B0%E6%8D%AE\GDR3855-Hylocereus_undulatus_Britt-12-RNAseq_result\4_Function\2_Group_Diff_Function\UP_DOWN\GO\NL-VS-L1.P.html#gene188) | acetyl-CoA metabolic process | 2 (0.16%) | 3 (0.09%) | 0.305668 | 0.999992 |
| 189 | [GO:0006304](file:///E:\2018-7-3%E7%81%AB%E9%BE%99%E6%9E%9C%E8%BD%AC%E5%BD%95%E7%BB%84%E6%B5%8B%E5%BA%8F\%E5%AE%8C%E6%95%B4%E7%89%88%E6%95%B0%E6%8D%AE\GDR3855-Hylocereus_undulatus_Britt-12-RNAseq_result\4_Function\2_Group_Diff_Function\UP_DOWN\GO\NL-VS-L1.P.html#gene189) | DNA modification | 2 (0.16%) | 3 (0.09%) | 0.305668 | 0.999992 |
| 190 | [GO:0006305](file:///E:\2018-7-3%E7%81%AB%E9%BE%99%E6%9E%9C%E8%BD%AC%E5%BD%95%E7%BB%84%E6%B5%8B%E5%BA%8F\%E5%AE%8C%E6%95%B4%E7%89%88%E6%95%B0%E6%8D%AE\GDR3855-Hylocereus_undulatus_Britt-12-RNAseq_result\4_Function\2_Group_Diff_Function\UP_DOWN\GO\NL-VS-L1.P.html#gene190) | DNA alkylation | 2 (0.16%) | 3 (0.09%) | 0.305668 | 0.999992 |
| 191 | [GO:0006306](file:///E:\2018-7-3%E7%81%AB%E9%BE%99%E6%9E%9C%E8%BD%AC%E5%BD%95%E7%BB%84%E6%B5%8B%E5%BA%8F\%E5%AE%8C%E6%95%B4%E7%89%88%E6%95%B0%E6%8D%AE\GDR3855-Hylocereus_undulatus_Britt-12-RNAseq_result\4_Function\2_Group_Diff_Function\UP_DOWN\GO\NL-VS-L1.P.html#gene191) | DNA methylation | 2 (0.16%) | 3 (0.09%) | 0.305668 | 0.999992 |
| 192 | [GO:0006813](file:///E:\2018-7-3%E7%81%AB%E9%BE%99%E6%9E%9C%E8%BD%AC%E5%BD%95%E7%BB%84%E6%B5%8B%E5%BA%8F\%E5%AE%8C%E6%95%B4%E7%89%88%E6%95%B0%E6%8D%AE\GDR3855-Hylocereus_undulatus_Britt-12-RNAseq_result\4_Function\2_Group_Diff_Function\UP_DOWN\GO\NL-VS-L1.P.html#gene192) | potassium ion transport | 2 (0.16%) | 3 (0.09%) | 0.305668 | 0.999992 |
| 193 | [GO:0009738](file:///E:\2018-7-3%E7%81%AB%E9%BE%99%E6%9E%9C%E8%BD%AC%E5%BD%95%E7%BB%84%E6%B5%8B%E5%BA%8F\%E5%AE%8C%E6%95%B4%E7%89%88%E6%95%B0%E6%8D%AE\GDR3855-Hylocereus_undulatus_Britt-12-RNAseq_result\4_Function\2_Group_Diff_Function\UP_DOWN\GO\NL-VS-L1.P.html#gene193) | abscisic acid-activated signaling pathway | 2 (0.16%) | 3 (0.09%) | 0.305668 | 0.999992 |
| 194 | [GO:0009886](file:///E:\2018-7-3%E7%81%AB%E9%BE%99%E6%9E%9C%E8%BD%AC%E5%BD%95%E7%BB%84%E6%B5%8B%E5%BA%8F\%E5%AE%8C%E6%95%B4%E7%89%88%E6%95%B0%E6%8D%AE\GDR3855-Hylocereus_undulatus_Britt-12-RNAseq_result\4_Function\2_Group_Diff_Function\UP_DOWN\GO\NL-VS-L1.P.html#gene194) | post-embryonic morphogenesis | 2 (0.16%) | 3 (0.09%) | 0.305668 | 0.999992 |
| 195 | [GO:0016441](file:///E:\2018-7-3%E7%81%AB%E9%BE%99%E6%9E%9C%E8%BD%AC%E5%BD%95%E7%BB%84%E6%B5%8B%E5%BA%8F\%E5%AE%8C%E6%95%B4%E7%89%88%E6%95%B0%E6%8D%AE\GDR3855-Hylocereus_undulatus_Britt-12-RNAseq_result\4_Function\2_Group_Diff_Function\UP_DOWN\GO\NL-VS-L1.P.html#gene195) | posttranscriptional gene silencing | 2 (0.16%) | 3 (0.09%) | 0.305668 | 0.999992 |
| 196 | [GO:0031047](file:///E:\2018-7-3%E7%81%AB%E9%BE%99%E6%9E%9C%E8%BD%AC%E5%BD%95%E7%BB%84%E6%B5%8B%E5%BA%8F\%E5%AE%8C%E6%95%B4%E7%89%88%E6%95%B0%E6%8D%AE\GDR3855-Hylocereus_undulatus_Britt-12-RNAseq_result\4_Function\2_Group_Diff_Function\UP_DOWN\GO\NL-VS-L1.P.html#gene196) | gene silencing by RNA | 2 (0.16%) | 3 (0.09%) | 0.305668 | 0.999992 |
| 197 | [GO:0035194](file:///E:\2018-7-3%E7%81%AB%E9%BE%99%E6%9E%9C%E8%BD%AC%E5%BD%95%E7%BB%84%E6%B5%8B%E5%BA%8F\%E5%AE%8C%E6%95%B4%E7%89%88%E6%95%B0%E6%8D%AE\GDR3855-Hylocereus_undulatus_Britt-12-RNAseq_result\4_Function\2_Group_Diff_Function\UP_DOWN\GO\NL-VS-L1.P.html#gene197) | posttranscriptional gene silencing by RNA | 2 (0.16%) | 3 (0.09%) | 0.305668 | 0.999992 |
| 198 | [GO:0042723](file:///E:\2018-7-3%E7%81%AB%E9%BE%99%E6%9E%9C%E8%BD%AC%E5%BD%95%E7%BB%84%E6%B5%8B%E5%BA%8F\%E5%AE%8C%E6%95%B4%E7%89%88%E6%95%B0%E6%8D%AE\GDR3855-Hylocereus_undulatus_Britt-12-RNAseq_result\4_Function\2_Group_Diff_Function\UP_DOWN\GO\NL-VS-L1.P.html#gene198) | thiamine-containing compound metabolic process | 2 (0.16%) | 3 (0.09%) | 0.305668 | 0.999992 |
| 199 | [GO:0044728](file:///E:\2018-7-3%E7%81%AB%E9%BE%99%E6%9E%9C%E8%BD%AC%E5%BD%95%E7%BB%84%E6%B5%8B%E5%BA%8F\%E5%AE%8C%E6%95%B4%E7%89%88%E6%95%B0%E6%8D%AE\GDR3855-Hylocereus_undulatus_Britt-12-RNAseq_result\4_Function\2_Group_Diff_Function\UP_DOWN\GO\NL-VS-L1.P.html#gene199) | DNA methylation or demethylation | 2 (0.16%) | 3 (0.09%) | 0.305668 | 0.999992 |
| 200 | [GO:0071215](file:///E:\2018-7-3%E7%81%AB%E9%BE%99%E6%9E%9C%E8%BD%AC%E5%BD%95%E7%BB%84%E6%B5%8B%E5%BA%8F\%E5%AE%8C%E6%95%B4%E7%89%88%E6%95%B0%E6%8D%AE\GDR3855-Hylocereus_undulatus_Britt-12-RNAseq_result\4_Function\2_Group_Diff_Function\UP_DOWN\GO\NL-VS-L1.P.html#gene200) | cellular response to abscisic acid stimulus | 2 (0.16%) | 3 (0.09%) | 0.305668 | 0.999992 |
| 201 | [GO:0071804](file:///E:\2018-7-3%E7%81%AB%E9%BE%99%E6%9E%9C%E8%BD%AC%E5%BD%95%E7%BB%84%E6%B5%8B%E5%BA%8F\%E5%AE%8C%E6%95%B4%E7%89%88%E6%95%B0%E6%8D%AE\GDR3855-Hylocereus_undulatus_Britt-12-RNAseq_result\4_Function\2_Group_Diff_Function\UP_DOWN\GO\NL-VS-L1.P.html#gene201) | cellular potassium ion transport | 2 (0.16%) | 3 (0.09%) | 0.305668 | 0.999992 |
| 202 | [GO:0071805](file:///E:\2018-7-3%E7%81%AB%E9%BE%99%E6%9E%9C%E8%BD%AC%E5%BD%95%E7%BB%84%E6%B5%8B%E5%BA%8F\%E5%AE%8C%E6%95%B4%E7%89%88%E6%95%B0%E6%8D%AE\GDR3855-Hylocereus_undulatus_Britt-12-RNAseq_result\4_Function\2_Group_Diff_Function\UP_DOWN\GO\NL-VS-L1.P.html#gene202) | potassium ion transmembrane transport | 2 (0.16%) | 3 (0.09%) | 0.305668 | 0.999992 |
| 203 | [GO:0097306](file:///E:\2018-7-3%E7%81%AB%E9%BE%99%E6%9E%9C%E8%BD%AC%E5%BD%95%E7%BB%84%E6%B5%8B%E5%BA%8F\%E5%AE%8C%E6%95%B4%E7%89%88%E6%95%B0%E6%8D%AE\GDR3855-Hylocereus_undulatus_Britt-12-RNAseq_result\4_Function\2_Group_Diff_Function\UP_DOWN\GO\NL-VS-L1.P.html#gene203) | cellular response to alcohol | 2 (0.16%) | 3 (0.09%) | 0.305668 | 0.999992 |
| 204 | [GO:0071705](file:///E:\2018-7-3%E7%81%AB%E9%BE%99%E6%9E%9C%E8%BD%AC%E5%BD%95%E7%BB%84%E6%B5%8B%E5%BA%8F\%E5%AE%8C%E6%95%B4%E7%89%88%E6%95%B0%E6%8D%AE\GDR3855-Hylocereus_undulatus_Britt-12-RNAseq_result\4_Function\2_Group_Diff_Function\UP_DOWN\GO\NL-VS-L1.P.html#gene204) | nitrogen compound transport | 12 (0.97%) | 28 (0.83%) | 0.312058 | 0.999992 |
| 205 | [GO:0051704](file:///E:\2018-7-3%E7%81%AB%E9%BE%99%E6%9E%9C%E8%BD%AC%E5%BD%95%E7%BB%84%E6%B5%8B%E5%BA%8F\%E5%AE%8C%E6%95%B4%E7%89%88%E6%95%B0%E6%8D%AE\GDR3855-Hylocereus_undulatus_Britt-12-RNAseq_result\4_Function\2_Group_Diff_Function\UP_DOWN\GO\NL-VS-L1.P.html#gene205) | multi-organism process | 30 (2.43%) | 75 (2.23%) | 0.315870 | 0.999992 |
| 206 | [GO:0000041](file:///E:\2018-7-3%E7%81%AB%E9%BE%99%E6%9E%9C%E8%BD%AC%E5%BD%95%E7%BB%84%E6%B5%8B%E5%BA%8F\%E5%AE%8C%E6%95%B4%E7%89%88%E6%95%B0%E6%8D%AE\GDR3855-Hylocereus_undulatus_Britt-12-RNAseq_result\4_Function\2_Group_Diff_Function\UP_DOWN\GO\NL-VS-L1.P.html#gene206) | transition metal ion transport | 8 (0.65%) | 18 (0.54%) | 0.325974 | 0.999992 |
| 207 | [GO:0006457](file:///E:\2018-7-3%E7%81%AB%E9%BE%99%E6%9E%9C%E8%BD%AC%E5%BD%95%E7%BB%84%E6%B5%8B%E5%BA%8F\%E5%AE%8C%E6%95%B4%E7%89%88%E6%95%B0%E6%8D%AE\GDR3855-Hylocereus_undulatus_Britt-12-RNAseq_result\4_Function\2_Group_Diff_Function\UP_DOWN\GO\NL-VS-L1.P.html#gene207) | protein folding | 4 (0.32%) | 8 (0.24%) | 0.331413 | 0.999992 |
| 208 | [GO:0006766](file:///E:\2018-7-3%E7%81%AB%E9%BE%99%E6%9E%9C%E8%BD%AC%E5%BD%95%E7%BB%84%E6%B5%8B%E5%BA%8F\%E5%AE%8C%E6%95%B4%E7%89%88%E6%95%B0%E6%8D%AE\GDR3855-Hylocereus_undulatus_Britt-12-RNAseq_result\4_Function\2_Group_Diff_Function\UP_DOWN\GO\NL-VS-L1.P.html#gene208) | vitamin metabolic process | 4 (0.32%) | 8 (0.24%) | 0.331413 | 0.999992 |
| 209 | [GO:0006767](file:///E:\2018-7-3%E7%81%AB%E9%BE%99%E6%9E%9C%E8%BD%AC%E5%BD%95%E7%BB%84%E6%B5%8B%E5%BA%8F\%E5%AE%8C%E6%95%B4%E7%89%88%E6%95%B0%E6%8D%AE\GDR3855-Hylocereus_undulatus_Britt-12-RNAseq_result\4_Function\2_Group_Diff_Function\UP_DOWN\GO\NL-VS-L1.P.html#gene209) | water-soluble vitamin metabolic process | 4 (0.32%) | 8 (0.24%) | 0.331413 | 0.999992 |
| 210 | [GO:0009067](file:///E:\2018-7-3%E7%81%AB%E9%BE%99%E6%9E%9C%E8%BD%AC%E5%BD%95%E7%BB%84%E6%B5%8B%E5%BA%8F\%E5%AE%8C%E6%95%B4%E7%89%88%E6%95%B0%E6%8D%AE\GDR3855-Hylocereus_undulatus_Britt-12-RNAseq_result\4_Function\2_Group_Diff_Function\UP_DOWN\GO\NL-VS-L1.P.html#gene210) | aspartate family amino acid biosynthetic process | 4 (0.32%) | 8 (0.24%) | 0.331413 | 0.999992 |
| 211 | [GO:0009790](file:///E:\2018-7-3%E7%81%AB%E9%BE%99%E6%9E%9C%E8%BD%AC%E5%BD%95%E7%BB%84%E6%B5%8B%E5%BA%8F\%E5%AE%8C%E6%95%B4%E7%89%88%E6%95%B0%E6%8D%AE\GDR3855-Hylocereus_undulatus_Britt-12-RNAseq_result\4_Function\2_Group_Diff_Function\UP_DOWN\GO\NL-VS-L1.P.html#gene211) | embryo development | 4 (0.32%) | 8 (0.24%) | 0.331413 | 0.999992 |
| 212 | [GO:0022607](file:///E:\2018-7-3%E7%81%AB%E9%BE%99%E6%9E%9C%E8%BD%AC%E5%BD%95%E7%BB%84%E6%B5%8B%E5%BA%8F\%E5%AE%8C%E6%95%B4%E7%89%88%E6%95%B0%E6%8D%AE\GDR3855-Hylocereus_undulatus_Britt-12-RNAseq_result\4_Function\2_Group_Diff_Function\UP_DOWN\GO\NL-VS-L1.P.html#gene212) | cellular component assembly | 21 (1.7%) | 52 (1.55%) | 0.339133 | 0.999992 |
| 213 | [GO:0032259](file:///E:\2018-7-3%E7%81%AB%E9%BE%99%E6%9E%9C%E8%BD%AC%E5%BD%95%E7%BB%84%E6%B5%8B%E5%BA%8F\%E5%AE%8C%E6%95%B4%E7%89%88%E6%95%B0%E6%8D%AE\GDR3855-Hylocereus_undulatus_Britt-12-RNAseq_result\4_Function\2_Group_Diff_Function\UP_DOWN\GO\NL-VS-L1.P.html#gene213) | methylation | 11 (0.89%) | 26 (0.77%) | 0.344009 | 0.999992 |
| 214 | [GO:0044282](file:///E:\2018-7-3%E7%81%AB%E9%BE%99%E6%9E%9C%E8%BD%AC%E5%BD%95%E7%BB%84%E6%B5%8B%E5%BA%8F\%E5%AE%8C%E6%95%B4%E7%89%88%E6%95%B0%E6%8D%AE\GDR3855-Hylocereus_undulatus_Britt-12-RNAseq_result\4_Function\2_Group_Diff_Function\UP_DOWN\GO\NL-VS-L1.P.html#gene214) | small molecule catabolic process | 11 (0.89%) | 26 (0.77%) | 0.344009 | 0.999992 |
| 215 | [GO:0099402](file:///E:\2018-7-3%E7%81%AB%E9%BE%99%E6%9E%9C%E8%BD%AC%E5%BD%95%E7%BB%84%E6%B5%8B%E5%BA%8F\%E5%AE%8C%E6%95%B4%E7%89%88%E6%95%B0%E6%8D%AE\GDR3855-Hylocereus_undulatus_Britt-12-RNAseq_result\4_Function\2_Group_Diff_Function\UP_DOWN\GO\NL-VS-L1.P.html#gene215) | plant organ development | 11 (0.89%) | 26 (0.77%) | 0.344009 | 0.999992 |
| 216 | [GO:0044085](file:///E:\2018-7-3%E7%81%AB%E9%BE%99%E6%9E%9C%E8%BD%AC%E5%BD%95%E7%BB%84%E6%B5%8B%E5%BA%8F\%E5%AE%8C%E6%95%B4%E7%89%88%E6%95%B0%E6%8D%AE\GDR3855-Hylocereus_undulatus_Britt-12-RNAseq_result\4_Function\2_Group_Diff_Function\UP_DOWN\GO\NL-VS-L1.P.html#gene216) | cellular component biogenesis | 36 (2.92%) | 92 (2.74%) | 0.351667 | 0.999992 |
| 217 | [GO:0044272](file:///E:\2018-7-3%E7%81%AB%E9%BE%99%E6%9E%9C%E8%BD%AC%E5%BD%95%E7%BB%84%E6%B5%8B%E5%BA%8F\%E5%AE%8C%E6%95%B4%E7%89%88%E6%95%B0%E6%8D%AE\GDR3855-Hylocereus_undulatus_Britt-12-RNAseq_result\4_Function\2_Group_Diff_Function\UP_DOWN\GO\NL-VS-L1.P.html#gene217) | sulfur compound biosynthetic process | 14 (1.13%) | 34 (1.01%) | 0.354296 | 0.999992 |
| 218 | [GO:0006006](file:///E:\2018-7-3%E7%81%AB%E9%BE%99%E6%9E%9C%E8%BD%AC%E5%BD%95%E7%BB%84%E6%B5%8B%E5%BA%8F\%E5%AE%8C%E6%95%B4%E7%89%88%E6%95%B0%E6%8D%AE\GDR3855-Hylocereus_undulatus_Britt-12-RNAseq_result\4_Function\2_Group_Diff_Function\UP_DOWN\GO\NL-VS-L1.P.html#gene218) | glucose metabolic process | 9 (0.73%) | 21 (0.63%) | 0.354620 | 0.999992 |
| 219 | [GO:0009991](file:///E:\2018-7-3%E7%81%AB%E9%BE%99%E6%9E%9C%E8%BD%AC%E5%BD%95%E7%BB%84%E6%B5%8B%E5%BA%8F\%E5%AE%8C%E6%95%B4%E7%89%88%E6%95%B0%E6%8D%AE\GDR3855-Hylocereus_undulatus_Britt-12-RNAseq_result\4_Function\2_Group_Diff_Function\UP_DOWN\GO\NL-VS-L1.P.html#gene219) | response to extracellular stimulus | 9 (0.73%) | 21 (0.63%) | 0.354620 | 0.999992 |
| 220 | [GO:0016569](file:///E:\2018-7-3%E7%81%AB%E9%BE%99%E6%9E%9C%E8%BD%AC%E5%BD%95%E7%BB%84%E6%B5%8B%E5%BA%8F\%E5%AE%8C%E6%95%B4%E7%89%88%E6%95%B0%E6%8D%AE\GDR3855-Hylocereus_undulatus_Britt-12-RNAseq_result\4_Function\2_Group_Diff_Function\UP_DOWN\GO\NL-VS-L1.P.html#gene220) | covalent chromatin modification | 9 (0.73%) | 21 (0.63%) | 0.354620 | 0.999992 |
| 221 | [GO:0031667](file:///E:\2018-7-3%E7%81%AB%E9%BE%99%E6%9E%9C%E8%BD%AC%E5%BD%95%E7%BB%84%E6%B5%8B%E5%BA%8F\%E5%AE%8C%E6%95%B4%E7%89%88%E6%95%B0%E6%8D%AE\GDR3855-Hylocereus_undulatus_Britt-12-RNAseq_result\4_Function\2_Group_Diff_Function\UP_DOWN\GO\NL-VS-L1.P.html#gene221) | response to nutrient levels | 9 (0.73%) | 21 (0.63%) | 0.354620 | 0.999992 |
| 222 | [GO:0016043](file:///E:\2018-7-3%E7%81%AB%E9%BE%99%E6%9E%9C%E8%BD%AC%E5%BD%95%E7%BB%84%E6%B5%8B%E5%BA%8F\%E5%AE%8C%E6%95%B4%E7%89%88%E6%95%B0%E6%8D%AE\GDR3855-Hylocereus_undulatus_Britt-12-RNAseq_result\4_Function\2_Group_Diff_Function\UP_DOWN\GO\NL-VS-L1.P.html#gene222) | cellular component organization | 90 (7.29%) | 237 (7.06%) | 0.365180 | 0.999992 |
| 223 | [GO:0051273](file:///E:\2018-7-3%E7%81%AB%E9%BE%99%E6%9E%9C%E8%BD%AC%E5%BD%95%E7%BB%84%E6%B5%8B%E5%BA%8F\%E5%AE%8C%E6%95%B4%E7%89%88%E6%95%B0%E6%8D%AE\GDR3855-Hylocereus_undulatus_Britt-12-RNAseq_result\4_Function\2_Group_Diff_Function\UP_DOWN\GO\NL-VS-L1.P.html#gene223) | beta-glucan metabolic process | 12 (0.97%) | 29 (0.86%) | 0.366281 | 0.999992 |
| 224 | [GO:0000070](file:///E:\2018-7-3%E7%81%AB%E9%BE%99%E6%9E%9C%E8%BD%AC%E5%BD%95%E7%BB%84%E6%B5%8B%E5%BA%8F\%E5%AE%8C%E6%95%B4%E7%89%88%E6%95%B0%E6%8D%AE\GDR3855-Hylocereus_undulatus_Britt-12-RNAseq_result\4_Function\2_Group_Diff_Function\UP_DOWN\GO\NL-VS-L1.P.html#gene224) | mitotic sister chromatid segregation | 1 (0.08%) | 1 (0.03%) | 0.367371 | 0.999992 |
| 225 | [GO:0000741](file:///E:\2018-7-3%E7%81%AB%E9%BE%99%E6%9E%9C%E8%BD%AC%E5%BD%95%E7%BB%84%E6%B5%8B%E5%BA%8F\%E5%AE%8C%E6%95%B4%E7%89%88%E6%95%B0%E6%8D%AE\GDR3855-Hylocereus_undulatus_Britt-12-RNAseq_result\4_Function\2_Group_Diff_Function\UP_DOWN\GO\NL-VS-L1.P.html#gene225) | karyogamy | 1 (0.08%) | 1 (0.03%) | 0.367371 | 0.999992 |
| 226 | [GO:0002697](file:///E:\2018-7-3%E7%81%AB%E9%BE%99%E6%9E%9C%E8%BD%AC%E5%BD%95%E7%BB%84%E6%B5%8B%E5%BA%8F\%E5%AE%8C%E6%95%B4%E7%89%88%E6%95%B0%E6%8D%AE\GDR3855-Hylocereus_undulatus_Britt-12-RNAseq_result\4_Function\2_Group_Diff_Function\UP_DOWN\GO\NL-VS-L1.P.html#gene226) | regulation of immune effector process | 1 (0.08%) | 1 (0.03%) | 0.367371 | 0.999992 |
| 227 | [GO:0006549](file:///E:\2018-7-3%E7%81%AB%E9%BE%99%E6%9E%9C%E8%BD%AC%E5%BD%95%E7%BB%84%E6%B5%8B%E5%BA%8F\%E5%AE%8C%E6%95%B4%E7%89%88%E6%95%B0%E6%8D%AE\GDR3855-Hylocereus_undulatus_Britt-12-RNAseq_result\4_Function\2_Group_Diff_Function\UP_DOWN\GO\NL-VS-L1.P.html#gene227) | isoleucine metabolic process | 1 (0.08%) | 1 (0.03%) | 0.367371 | 0.999992 |
| 228 | [GO:0006566](file:///E:\2018-7-3%E7%81%AB%E9%BE%99%E6%9E%9C%E8%BD%AC%E5%BD%95%E7%BB%84%E6%B5%8B%E5%BA%8F\%E5%AE%8C%E6%95%B4%E7%89%88%E6%95%B0%E6%8D%AE\GDR3855-Hylocereus_undulatus_Britt-12-RNAseq_result\4_Function\2_Group_Diff_Function\UP_DOWN\GO\NL-VS-L1.P.html#gene228) | threonine metabolic process | 1 (0.08%) | 1 (0.03%) | 0.367371 | 0.999992 |
| 229 | [GO:0006626](file:///E:\2018-7-3%E7%81%AB%E9%BE%99%E6%9E%9C%E8%BD%AC%E5%BD%95%E7%BB%84%E6%B5%8B%E5%BA%8F\%E5%AE%8C%E6%95%B4%E7%89%88%E6%95%B0%E6%8D%AE\GDR3855-Hylocereus_undulatus_Britt-12-RNAseq_result\4_Function\2_Group_Diff_Function\UP_DOWN\GO\NL-VS-L1.P.html#gene229) | protein targeting to mitochondrion | 1 (0.08%) | 1 (0.03%) | 0.367371 | 0.999992 |
| 230 | [GO:0006636](file:///E:\2018-7-3%E7%81%AB%E9%BE%99%E6%9E%9C%E8%BD%AC%E5%BD%95%E7%BB%84%E6%B5%8B%E5%BA%8F\%E5%AE%8C%E6%95%B4%E7%89%88%E6%95%B0%E6%8D%AE\GDR3855-Hylocereus_undulatus_Britt-12-RNAseq_result\4_Function\2_Group_Diff_Function\UP_DOWN\GO\NL-VS-L1.P.html#gene230) | unsaturated fatty acid biosynthetic process | 1 (0.08%) | 1 (0.03%) | 0.367371 | 0.999992 |
| 231 | [GO:0006658](file:///E:\2018-7-3%E7%81%AB%E9%BE%99%E6%9E%9C%E8%BD%AC%E5%BD%95%E7%BB%84%E6%B5%8B%E5%BA%8F\%E5%AE%8C%E6%95%B4%E7%89%88%E6%95%B0%E6%8D%AE\GDR3855-Hylocereus_undulatus_Britt-12-RNAseq_result\4_Function\2_Group_Diff_Function\UP_DOWN\GO\NL-VS-L1.P.html#gene231) | phosphatidylserine metabolic process | 1 (0.08%) | 1 (0.03%) | 0.367371 | 0.999992 |
| 232 | [GO:0006768](file:///E:\2018-7-3%E7%81%AB%E9%BE%99%E6%9E%9C%E8%BD%AC%E5%BD%95%E7%BB%84%E6%B5%8B%E5%BA%8F\%E5%AE%8C%E6%95%B4%E7%89%88%E6%95%B0%E6%8D%AE\GDR3855-Hylocereus_undulatus_Britt-12-RNAseq_result\4_Function\2_Group_Diff_Function\UP_DOWN\GO\NL-VS-L1.P.html#gene232) | biotin metabolic process | 1 (0.08%) | 1 (0.03%) | 0.367371 | 0.999992 |
| 233 | [GO:0006997](file:///E:\2018-7-3%E7%81%AB%E9%BE%99%E6%9E%9C%E8%BD%AC%E5%BD%95%E7%BB%84%E6%B5%8B%E5%BA%8F\%E5%AE%8C%E6%95%B4%E7%89%88%E6%95%B0%E6%8D%AE\GDR3855-Hylocereus_undulatus_Britt-12-RNAseq_result\4_Function\2_Group_Diff_Function\UP_DOWN\GO\NL-VS-L1.P.html#gene233) | nucleus organization | 1 (0.08%) | 1 (0.03%) | 0.367371 | 0.999992 |
| 234 | [GO:0007067](file:///E:\2018-7-3%E7%81%AB%E9%BE%99%E6%9E%9C%E8%BD%AC%E5%BD%95%E7%BB%84%E6%B5%8B%E5%BA%8F\%E5%AE%8C%E6%95%B4%E7%89%88%E6%95%B0%E6%8D%AE\GDR3855-Hylocereus_undulatus_Britt-12-RNAseq_result\4_Function\2_Group_Diff_Function\UP_DOWN\GO\NL-VS-L1.P.html#gene234) | mitotic nuclear division | 1 (0.08%) | 1 (0.03%) | 0.367371 | 0.999992 |
| 235 | [GO:0007088](file:///E:\2018-7-3%E7%81%AB%E9%BE%99%E6%9E%9C%E8%BD%AC%E5%BD%95%E7%BB%84%E6%B5%8B%E5%BA%8F\%E5%AE%8C%E6%95%B4%E7%89%88%E6%95%B0%E6%8D%AE\GDR3855-Hylocereus_undulatus_Britt-12-RNAseq_result\4_Function\2_Group_Diff_Function\UP_DOWN\GO\NL-VS-L1.P.html#gene235) | regulation of mitotic nuclear division | 1 (0.08%) | 1 (0.03%) | 0.367371 | 0.999992 |
| 236 | [GO:0009226](file:///E:\2018-7-3%E7%81%AB%E9%BE%99%E6%9E%9C%E8%BD%AC%E5%BD%95%E7%BB%84%E6%B5%8B%E5%BA%8F\%E5%AE%8C%E6%95%B4%E7%89%88%E6%95%B0%E6%8D%AE\GDR3855-Hylocereus_undulatus_Britt-12-RNAseq_result\4_Function\2_Group_Diff_Function\UP_DOWN\GO\NL-VS-L1.P.html#gene236) | nucleotide-sugar biosynthetic process | 1 (0.08%) | 1 (0.03%) | 0.367371 | 0.999992 |
| 237 | [GO:0009250](file:///E:\2018-7-3%E7%81%AB%E9%BE%99%E6%9E%9C%E8%BD%AC%E5%BD%95%E7%BB%84%E6%B5%8B%E5%BA%8F\%E5%AE%8C%E6%95%B4%E7%89%88%E6%95%B0%E6%8D%AE\GDR3855-Hylocereus_undulatus_Britt-12-RNAseq_result\4_Function\2_Group_Diff_Function\UP_DOWN\GO\NL-VS-L1.P.html#gene237) | glucan biosynthetic process | 1 (0.08%) | 1 (0.03%) | 0.367371 | 0.999992 |
| 238 | [GO:0009612](file:///E:\2018-7-3%E7%81%AB%E9%BE%99%E6%9E%9C%E8%BD%AC%E5%BD%95%E7%BB%84%E6%B5%8B%E5%BA%8F\%E5%AE%8C%E6%95%B4%E7%89%88%E6%95%B0%E6%8D%AE\GDR3855-Hylocereus_undulatus_Britt-12-RNAseq_result\4_Function\2_Group_Diff_Function\UP_DOWN\GO\NL-VS-L1.P.html#gene238) | response to mechanical stimulus | 1 (0.08%) | 1 (0.03%) | 0.367371 | 0.999992 |
| 239 | [GO:0009799](file:///E:\2018-7-3%E7%81%AB%E9%BE%99%E6%9E%9C%E8%BD%AC%E5%BD%95%E7%BB%84%E6%B5%8B%E5%BA%8F\%E5%AE%8C%E6%95%B4%E7%89%88%E6%95%B0%E6%8D%AE\GDR3855-Hylocereus_undulatus_Britt-12-RNAseq_result\4_Function\2_Group_Diff_Function\UP_DOWN\GO\NL-VS-L1.P.html#gene239) | specification of symmetry | 1 (0.08%) | 1 (0.03%) | 0.367371 | 0.999992 |
| 240 | [GO:0009965](file:///E:\2018-7-3%E7%81%AB%E9%BE%99%E6%9E%9C%E8%BD%AC%E5%BD%95%E7%BB%84%E6%B5%8B%E5%BA%8F\%E5%AE%8C%E6%95%B4%E7%89%88%E6%95%B0%E6%8D%AE\GDR3855-Hylocereus_undulatus_Britt-12-RNAseq_result\4_Function\2_Group_Diff_Function\UP_DOWN\GO\NL-VS-L1.P.html#gene240) | leaf morphogenesis | 1 (0.08%) | 1 (0.03%) | 0.367371 | 0.999992 |
| 241 | [GO:0010036](file:///E:\2018-7-3%E7%81%AB%E9%BE%99%E6%9E%9C%E8%BD%AC%E5%BD%95%E7%BB%84%E6%B5%8B%E5%BA%8F\%E5%AE%8C%E6%95%B4%E7%89%88%E6%95%B0%E6%8D%AE\GDR3855-Hylocereus_undulatus_Britt-12-RNAseq_result\4_Function\2_Group_Diff_Function\UP_DOWN\GO\NL-VS-L1.P.html#gene241) | response to boron-containing substance | 1 (0.08%) | 1 (0.03%) | 0.367371 | 0.999992 |
| 242 | [GO:0010103](file:///E:\2018-7-3%E7%81%AB%E9%BE%99%E6%9E%9C%E8%BD%AC%E5%BD%95%E7%BB%84%E6%B5%8B%E5%BA%8F\%E5%AE%8C%E6%95%B4%E7%89%88%E6%95%B0%E6%8D%AE\GDR3855-Hylocereus_undulatus_Britt-12-RNAseq_result\4_Function\2_Group_Diff_Function\UP_DOWN\GO\NL-VS-L1.P.html#gene242) | stomatal complex morphogenesis | 1 (0.08%) | 1 (0.03%) | 0.367371 | 0.999992 |
| 243 | [GO:0010191](file:///E:\2018-7-3%E7%81%AB%E9%BE%99%E6%9E%9C%E8%BD%AC%E5%BD%95%E7%BB%84%E6%B5%8B%E5%BA%8F\%E5%AE%8C%E6%95%B4%E7%89%88%E6%95%B0%E6%8D%AE\GDR3855-Hylocereus_undulatus_Britt-12-RNAseq_result\4_Function\2_Group_Diff_Function\UP_DOWN\GO\NL-VS-L1.P.html#gene243) | mucilage metabolic process | 1 (0.08%) | 1 (0.03%) | 0.367371 | 0.999992 |
| 244 | [GO:0010192](file:///E:\2018-7-3%E7%81%AB%E9%BE%99%E6%9E%9C%E8%BD%AC%E5%BD%95%E7%BB%84%E6%B5%8B%E5%BA%8F\%E5%AE%8C%E6%95%B4%E7%89%88%E6%95%B0%E6%8D%AE\GDR3855-Hylocereus_undulatus_Britt-12-RNAseq_result\4_Function\2_Group_Diff_Function\UP_DOWN\GO\NL-VS-L1.P.html#gene244) | mucilage biosynthetic process | 1 (0.08%) | 1 (0.03%) | 0.367371 | 0.999992 |
| 245 | [GO:0010207](file:///E:\2018-7-3%E7%81%AB%E9%BE%99%E6%9E%9C%E8%BD%AC%E5%BD%95%E7%BB%84%E6%B5%8B%E5%BA%8F\%E5%AE%8C%E6%95%B4%E7%89%88%E6%95%B0%E6%8D%AE\GDR3855-Hylocereus_undulatus_Britt-12-RNAseq_result\4_Function\2_Group_Diff_Function\UP_DOWN\GO\NL-VS-L1.P.html#gene245) | photosystem II assembly | 1 (0.08%) | 1 (0.03%) | 0.367371 | 0.999992 |
| 246 | [GO:0010208](file:///E:\2018-7-3%E7%81%AB%E9%BE%99%E6%9E%9C%E8%BD%AC%E5%BD%95%E7%BB%84%E6%B5%8B%E5%BA%8F\%E5%AE%8C%E6%95%B4%E7%89%88%E6%95%B0%E6%8D%AE\GDR3855-Hylocereus_undulatus_Britt-12-RNAseq_result\4_Function\2_Group_Diff_Function\UP_DOWN\GO\NL-VS-L1.P.html#gene246) | pollen wall assembly | 1 (0.08%) | 1 (0.03%) | 0.367371 | 0.999992 |
| 247 | [GO:0010374](file:///E:\2018-7-3%E7%81%AB%E9%BE%99%E6%9E%9C%E8%BD%AC%E5%BD%95%E7%BB%84%E6%B5%8B%E5%BA%8F\%E5%AE%8C%E6%95%B4%E7%89%88%E6%95%B0%E6%8D%AE\GDR3855-Hylocereus_undulatus_Britt-12-RNAseq_result\4_Function\2_Group_Diff_Function\UP_DOWN\GO\NL-VS-L1.P.html#gene247) | stomatal complex development | 1 (0.08%) | 1 (0.03%) | 0.367371 | 0.999992 |
| 248 | [GO:0010413](file:///E:\2018-7-3%E7%81%AB%E9%BE%99%E6%9E%9C%E8%BD%AC%E5%BD%95%E7%BB%84%E6%B5%8B%E5%BA%8F\%E5%AE%8C%E6%95%B4%E7%89%88%E6%95%B0%E6%8D%AE\GDR3855-Hylocereus_undulatus_Britt-12-RNAseq_result\4_Function\2_Group_Diff_Function\UP_DOWN\GO\NL-VS-L1.P.html#gene248) | glucuronoxylan metabolic process | 1 (0.08%) | 1 (0.03%) | 0.367371 | 0.999992 |
| 249 | [GO:0010639](file:///E:\2018-7-3%E7%81%AB%E9%BE%99%E6%9E%9C%E8%BD%AC%E5%BD%95%E7%BB%84%E6%B5%8B%E5%BA%8F\%E5%AE%8C%E6%95%B4%E7%89%88%E6%95%B0%E6%8D%AE\GDR3855-Hylocereus_undulatus_Britt-12-RNAseq_result\4_Function\2_Group_Diff_Function\UP_DOWN\GO\NL-VS-L1.P.html#gene249) | negative regulation of organelle organization | 1 (0.08%) | 1 (0.03%) | 0.367371 | 0.999992 |
| 250 | [GO:0010927](file:///E:\2018-7-3%E7%81%AB%E9%BE%99%E6%9E%9C%E8%BD%AC%E5%BD%95%E7%BB%84%E6%B5%8B%E5%BA%8F\%E5%AE%8C%E6%95%B4%E7%89%88%E6%95%B0%E6%8D%AE\GDR3855-Hylocereus_undulatus_Britt-12-RNAseq_result\4_Function\2_Group_Diff_Function\UP_DOWN\GO\NL-VS-L1.P.html#gene250) | cellular component assembly involved in morphogenesis | 1 (0.08%) | 1 (0.03%) | 0.367371 | 0.999992 |
| 251 | [GO:0015837](file:///E:\2018-7-3%E7%81%AB%E9%BE%99%E6%9E%9C%E8%BD%AC%E5%BD%95%E7%BB%84%E6%B5%8B%E5%BA%8F\%E5%AE%8C%E6%95%B4%E7%89%88%E6%95%B0%E6%8D%AE\GDR3855-Hylocereus_undulatus_Britt-12-RNAseq_result\4_Function\2_Group_Diff_Function\UP_DOWN\GO\NL-VS-L1.P.html#gene251) | amine transport | 1 (0.08%) | 1 (0.03%) | 0.367371 | 0.999992 |
| 252 | [GO:0015843](file:///E:\2018-7-3%E7%81%AB%E9%BE%99%E6%9E%9C%E8%BD%AC%E5%BD%95%E7%BB%84%E6%B5%8B%E5%BA%8F\%E5%AE%8C%E6%95%B4%E7%89%88%E6%95%B0%E6%8D%AE\GDR3855-Hylocereus_undulatus_Britt-12-RNAseq_result\4_Function\2_Group_Diff_Function\UP_DOWN\GO\NL-VS-L1.P.html#gene252) | methylammonium transport | 1 (0.08%) | 1 (0.03%) | 0.367371 | 0.999992 |
| 253 | [GO:0015846](file:///E:\2018-7-3%E7%81%AB%E9%BE%99%E6%9E%9C%E8%BD%AC%E5%BD%95%E7%BB%84%E6%B5%8B%E5%BA%8F\%E5%AE%8C%E6%95%B4%E7%89%88%E6%95%B0%E6%8D%AE\GDR3855-Hylocereus_undulatus_Britt-12-RNAseq_result\4_Function\2_Group_Diff_Function\UP_DOWN\GO\NL-VS-L1.P.html#gene253) | polyamine transport | 1 (0.08%) | 1 (0.03%) | 0.367371 | 0.999992 |
| 254 | [GO:0015936](file:///E:\2018-7-3%E7%81%AB%E9%BE%99%E6%9E%9C%E8%BD%AC%E5%BD%95%E7%BB%84%E6%B5%8B%E5%BA%8F\%E5%AE%8C%E6%95%B4%E7%89%88%E6%95%B0%E6%8D%AE\GDR3855-Hylocereus_undulatus_Britt-12-RNAseq_result\4_Function\2_Group_Diff_Function\UP_DOWN\GO\NL-VS-L1.P.html#gene254) | coenzyme A metabolic process | 1 (0.08%) | 1 (0.03%) | 0.367371 | 0.999992 |
| 255 | [GO:0015937](file:///E:\2018-7-3%E7%81%AB%E9%BE%99%E6%9E%9C%E8%BD%AC%E5%BD%95%E7%BB%84%E6%B5%8B%E5%BA%8F\%E5%AE%8C%E6%95%B4%E7%89%88%E6%95%B0%E6%8D%AE\GDR3855-Hylocereus_undulatus_Britt-12-RNAseq_result\4_Function\2_Group_Diff_Function\UP_DOWN\GO\NL-VS-L1.P.html#gene255) | coenzyme A biosynthetic process | 1 (0.08%) | 1 (0.03%) | 0.367371 | 0.999992 |
| 256 | [GO:0018065](file:///E:\2018-7-3%E7%81%AB%E9%BE%99%E6%9E%9C%E8%BD%AC%E5%BD%95%E7%BB%84%E6%B5%8B%E5%BA%8F\%E5%AE%8C%E6%95%B4%E7%89%88%E6%95%B0%E6%8D%AE\GDR3855-Hylocereus_undulatus_Britt-12-RNAseq_result\4_Function\2_Group_Diff_Function\UP_DOWN\GO\NL-VS-L1.P.html#gene256) | protein-cofactor linkage | 1 (0.08%) | 1 (0.03%) | 0.367371 | 0.999992 |
| 257 | [GO:0018131](file:///E:\2018-7-3%E7%81%AB%E9%BE%99%E6%9E%9C%E8%BD%AC%E5%BD%95%E7%BB%84%E6%B5%8B%E5%BA%8F\%E5%AE%8C%E6%95%B4%E7%89%88%E6%95%B0%E6%8D%AE\GDR3855-Hylocereus_undulatus_Britt-12-RNAseq_result\4_Function\2_Group_Diff_Function\UP_DOWN\GO\NL-VS-L1.P.html#gene257) | oxazole or thiazole biosynthetic process | 1 (0.08%) | 1 (0.03%) | 0.367371 | 0.999992 |
| 258 | [GO:0019217](file:///E:\2018-7-3%E7%81%AB%E9%BE%99%E6%9E%9C%E8%BD%AC%E5%BD%95%E7%BB%84%E6%B5%8B%E5%BA%8F\%E5%AE%8C%E6%95%B4%E7%89%88%E6%95%B0%E6%8D%AE\GDR3855-Hylocereus_undulatus_Britt-12-RNAseq_result\4_Function\2_Group_Diff_Function\UP_DOWN\GO\NL-VS-L1.P.html#gene258) | regulation of fatty acid metabolic process | 1 (0.08%) | 1 (0.03%) | 0.367371 | 0.999992 |
| 259 | [GO:0019499](file:///E:\2018-7-3%E7%81%AB%E9%BE%99%E6%9E%9C%E8%BD%AC%E5%BD%95%E7%BB%84%E6%B5%8B%E5%BA%8F\%E5%AE%8C%E6%95%B4%E7%89%88%E6%95%B0%E6%8D%AE\GDR3855-Hylocereus_undulatus_Britt-12-RNAseq_result\4_Function\2_Group_Diff_Function\UP_DOWN\GO\NL-VS-L1.P.html#gene259) | cyanide metabolic process | 1 (0.08%) | 1 (0.03%) | 0.367371 | 0.999992 |
| 260 | [GO:0019566](file:///E:\2018-7-3%E7%81%AB%E9%BE%99%E6%9E%9C%E8%BD%AC%E5%BD%95%E7%BB%84%E6%B5%8B%E5%BA%8F\%E5%AE%8C%E6%95%B4%E7%89%88%E6%95%B0%E6%8D%AE\GDR3855-Hylocereus_undulatus_Britt-12-RNAseq_result\4_Function\2_Group_Diff_Function\UP_DOWN\GO\NL-VS-L1.P.html#gene260) | arabinose metabolic process | 1 (0.08%) | 1 (0.03%) | 0.367371 | 0.999992 |
| 261 | [GO:0019755](file:///E:\2018-7-3%E7%81%AB%E9%BE%99%E6%9E%9C%E8%BD%AC%E5%BD%95%E7%BB%84%E6%B5%8B%E5%BA%8F\%E5%AE%8C%E6%95%B4%E7%89%88%E6%95%B0%E6%8D%AE\GDR3855-Hylocereus_undulatus_Britt-12-RNAseq_result\4_Function\2_Group_Diff_Function\UP_DOWN\GO\NL-VS-L1.P.html#gene261) | one-carbon compound transport | 1 (0.08%) | 1 (0.03%) | 0.367371 | 0.999992 |
| 262 | [GO:0030198](file:///E:\2018-7-3%E7%81%AB%E9%BE%99%E6%9E%9C%E8%BD%AC%E5%BD%95%E7%BB%84%E6%B5%8B%E5%BA%8F\%E5%AE%8C%E6%95%B4%E7%89%88%E6%95%B0%E6%8D%AE\GDR3855-Hylocereus_undulatus_Britt-12-RNAseq_result\4_Function\2_Group_Diff_Function\UP_DOWN\GO\NL-VS-L1.P.html#gene262) | extracellular matrix organization | 1 (0.08%) | 1 (0.03%) | 0.367371 | 0.999992 |
| 263 | [GO:0030834](file:///E:\2018-7-3%E7%81%AB%E9%BE%99%E6%9E%9C%E8%BD%AC%E5%BD%95%E7%BB%84%E6%B5%8B%E5%BA%8F\%E5%AE%8C%E6%95%B4%E7%89%88%E6%95%B0%E6%8D%AE\GDR3855-Hylocereus_undulatus_Britt-12-RNAseq_result\4_Function\2_Group_Diff_Function\UP_DOWN\GO\NL-VS-L1.P.html#gene263) | regulation of actin filament depolymerization | 1 (0.08%) | 1 (0.03%) | 0.367371 | 0.999992 |
| 264 | [GO:0030835](file:///E:\2018-7-3%E7%81%AB%E9%BE%99%E6%9E%9C%E8%BD%AC%E5%BD%95%E7%BB%84%E6%B5%8B%E5%BA%8F\%E5%AE%8C%E6%95%B4%E7%89%88%E6%95%B0%E6%8D%AE\GDR3855-Hylocereus_undulatus_Britt-12-RNAseq_result\4_Function\2_Group_Diff_Function\UP_DOWN\GO\NL-VS-L1.P.html#gene264) | negative regulation of actin filament depolymerization | 1 (0.08%) | 1 (0.03%) | 0.367371 | 0.999992 |
| 265 | [GO:0031023](file:///E:\2018-7-3%E7%81%AB%E9%BE%99%E6%9E%9C%E8%BD%AC%E5%BD%95%E7%BB%84%E6%B5%8B%E5%BA%8F\%E5%AE%8C%E6%95%B4%E7%89%88%E6%95%B0%E6%8D%AE\GDR3855-Hylocereus_undulatus_Britt-12-RNAseq_result\4_Function\2_Group_Diff_Function\UP_DOWN\GO\NL-VS-L1.P.html#gene265) | microtubule organizing center organization | 1 (0.08%) | 1 (0.03%) | 0.367371 | 0.999992 |
| 266 | [GO:0032101](file:///E:\2018-7-3%E7%81%AB%E9%BE%99%E6%9E%9C%E8%BD%AC%E5%BD%95%E7%BB%84%E6%B5%8B%E5%BA%8F\%E5%AE%8C%E6%95%B4%E7%89%88%E6%95%B0%E6%8D%AE\GDR3855-Hylocereus_undulatus_Britt-12-RNAseq_result\4_Function\2_Group_Diff_Function\UP_DOWN\GO\NL-VS-L1.P.html#gene266) | regulation of response to external stimulus | 1 (0.08%) | 1 (0.03%) | 0.367371 | 0.999992 |
| 267 | [GO:0033559](file:///E:\2018-7-3%E7%81%AB%E9%BE%99%E6%9E%9C%E8%BD%AC%E5%BD%95%E7%BB%84%E6%B5%8B%E5%BA%8F\%E5%AE%8C%E6%95%B4%E7%89%88%E6%95%B0%E6%8D%AE\GDR3855-Hylocereus_undulatus_Britt-12-RNAseq_result\4_Function\2_Group_Diff_Function\UP_DOWN\GO\NL-VS-L1.P.html#gene267) | unsaturated fatty acid metabolic process | 1 (0.08%) | 1 (0.03%) | 0.367371 | 0.999992 |
| 268 | [GO:0033865](file:///E:\2018-7-3%E7%81%AB%E9%BE%99%E6%9E%9C%E8%BD%AC%E5%BD%95%E7%BB%84%E6%B5%8B%E5%BA%8F\%E5%AE%8C%E6%95%B4%E7%89%88%E6%95%B0%E6%8D%AE\GDR3855-Hylocereus_undulatus_Britt-12-RNAseq_result\4_Function\2_Group_Diff_Function\UP_DOWN\GO\NL-VS-L1.P.html#gene268) | nucleoside bisphosphate metabolic process | 1 (0.08%) | 1 (0.03%) | 0.367371 | 0.999992 |
| 269 | [GO:0033866](file:///E:\2018-7-3%E7%81%AB%E9%BE%99%E6%9E%9C%E8%BD%AC%E5%BD%95%E7%BB%84%E6%B5%8B%E5%BA%8F\%E5%AE%8C%E6%95%B4%E7%89%88%E6%95%B0%E6%8D%AE\GDR3855-Hylocereus_undulatus_Britt-12-RNAseq_result\4_Function\2_Group_Diff_Function\UP_DOWN\GO\NL-VS-L1.P.html#gene269) | nucleoside bisphosphate biosynthetic process | 1 (0.08%) | 1 (0.03%) | 0.367371 | 0.999992 |
| 270 | [GO:0033875](file:///E:\2018-7-3%E7%81%AB%E9%BE%99%E6%9E%9C%E8%BD%AC%E5%BD%95%E7%BB%84%E6%B5%8B%E5%BA%8F\%E5%AE%8C%E6%95%B4%E7%89%88%E6%95%B0%E6%8D%AE\GDR3855-Hylocereus_undulatus_Britt-12-RNAseq_result\4_Function\2_Group_Diff_Function\UP_DOWN\GO\NL-VS-L1.P.html#gene270) | ribonucleoside bisphosphate metabolic process | 1 (0.08%) | 1 (0.03%) | 0.367371 | 0.999992 |
| 271 | [GO:0034030](file:///E:\2018-7-3%E7%81%AB%E9%BE%99%E6%9E%9C%E8%BD%AC%E5%BD%95%E7%BB%84%E6%B5%8B%E5%BA%8F\%E5%AE%8C%E6%95%B4%E7%89%88%E6%95%B0%E6%8D%AE\GDR3855-Hylocereus_undulatus_Britt-12-RNAseq_result\4_Function\2_Group_Diff_Function\UP_DOWN\GO\NL-VS-L1.P.html#gene271) | ribonucleoside bisphosphate biosynthetic process | 1 (0.08%) | 1 (0.03%) | 0.367371 | 0.999992 |
| 272 | [GO:0034032](file:///E:\2018-7-3%E7%81%AB%E9%BE%99%E6%9E%9C%E8%BD%AC%E5%BD%95%E7%BB%84%E6%B5%8B%E5%BA%8F\%E5%AE%8C%E6%95%B4%E7%89%88%E6%95%B0%E6%8D%AE\GDR3855-Hylocereus_undulatus_Britt-12-RNAseq_result\4_Function\2_Group_Diff_Function\UP_DOWN\GO\NL-VS-L1.P.html#gene272) | purine nucleoside bisphosphate metabolic process | 1 (0.08%) | 1 (0.03%) | 0.367371 | 0.999992 |
| 273 | [GO:0034033](file:///E:\2018-7-3%E7%81%AB%E9%BE%99%E6%9E%9C%E8%BD%AC%E5%BD%95%E7%BB%84%E6%B5%8B%E5%BA%8F\%E5%AE%8C%E6%95%B4%E7%89%88%E6%95%B0%E6%8D%AE\GDR3855-Hylocereus_undulatus_Britt-12-RNAseq_result\4_Function\2_Group_Diff_Function\UP_DOWN\GO\NL-VS-L1.P.html#gene273) | purine nucleoside bisphosphate biosynthetic process | 1 (0.08%) | 1 (0.03%) | 0.367371 | 0.999992 |
| 274 | [GO:0034330](file:///E:\2018-7-3%E7%81%AB%E9%BE%99%E6%9E%9C%E8%BD%AC%E5%BD%95%E7%BB%84%E6%B5%8B%E5%BA%8F\%E5%AE%8C%E6%95%B4%E7%89%88%E6%95%B0%E6%8D%AE\GDR3855-Hylocereus_undulatus_Britt-12-RNAseq_result\4_Function\2_Group_Diff_Function\UP_DOWN\GO\NL-VS-L1.P.html#gene274) | cell junction organization | 1 (0.08%) | 1 (0.03%) | 0.367371 | 0.999992 |
| 275 | [GO:0042304](file:///E:\2018-7-3%E7%81%AB%E9%BE%99%E6%9E%9C%E8%BD%AC%E5%BD%95%E7%BB%84%E6%B5%8B%E5%BA%8F\%E5%AE%8C%E6%95%B4%E7%89%88%E6%95%B0%E6%8D%AE\GDR3855-Hylocereus_undulatus_Britt-12-RNAseq_result\4_Function\2_Group_Diff_Function\UP_DOWN\GO\NL-VS-L1.P.html#gene275) | regulation of fatty acid biosynthetic process | 1 (0.08%) | 1 (0.03%) | 0.367371 | 0.999992 |
| 276 | [GO:0042350](file:///E:\2018-7-3%E7%81%AB%E9%BE%99%E6%9E%9C%E8%BD%AC%E5%BD%95%E7%BB%84%E6%B5%8B%E5%BA%8F\%E5%AE%8C%E6%95%B4%E7%89%88%E6%95%B0%E6%8D%AE\GDR3855-Hylocereus_undulatus_Britt-12-RNAseq_result\4_Function\2_Group_Diff_Function\UP_DOWN\GO\NL-VS-L1.P.html#gene276) | GDP-L-fucose biosynthetic process | 1 (0.08%) | 1 (0.03%) | 0.367371 | 0.999992 |
| 277 | [GO:0042939](file:///E:\2018-7-3%E7%81%AB%E9%BE%99%E6%9E%9C%E8%BD%AC%E5%BD%95%E7%BB%84%E6%B5%8B%E5%BA%8F\%E5%AE%8C%E6%95%B4%E7%89%88%E6%95%B0%E6%8D%AE\GDR3855-Hylocereus_undulatus_Britt-12-RNAseq_result\4_Function\2_Group_Diff_Function\UP_DOWN\GO\NL-VS-L1.P.html#gene277) | tripeptide transport | 1 (0.08%) | 1 (0.03%) | 0.367371 | 0.999992 |
| 278 | [GO:0043062](file:///E:\2018-7-3%E7%81%AB%E9%BE%99%E6%9E%9C%E8%BD%AC%E5%BD%95%E7%BB%84%E6%B5%8B%E5%BA%8F\%E5%AE%8C%E6%95%B4%E7%89%88%E6%95%B0%E6%8D%AE\GDR3855-Hylocereus_undulatus_Britt-12-RNAseq_result\4_Function\2_Group_Diff_Function\UP_DOWN\GO\NL-VS-L1.P.html#gene278) | extracellular structure organization | 1 (0.08%) | 1 (0.03%) | 0.367371 | 0.999992 |
| 279 | [GO:0043242](file:///E:\2018-7-3%E7%81%AB%E9%BE%99%E6%9E%9C%E8%BD%AC%E5%BD%95%E7%BB%84%E6%B5%8B%E5%BA%8F\%E5%AE%8C%E6%95%B4%E7%89%88%E6%95%B0%E6%8D%AE\GDR3855-Hylocereus_undulatus_Britt-12-RNAseq_result\4_Function\2_Group_Diff_Function\UP_DOWN\GO\NL-VS-L1.P.html#gene279) | negative regulation of protein complex disassembly | 1 (0.08%) | 1 (0.03%) | 0.367371 | 0.999992 |
| 280 | [GO:0043244](file:///E:\2018-7-3%E7%81%AB%E9%BE%99%E6%9E%9C%E8%BD%AC%E5%BD%95%E7%BB%84%E6%B5%8B%E5%BA%8F\%E5%AE%8C%E6%95%B4%E7%89%88%E6%95%B0%E6%8D%AE\GDR3855-Hylocereus_undulatus_Britt-12-RNAseq_result\4_Function\2_Group_Diff_Function\UP_DOWN\GO\NL-VS-L1.P.html#gene280) | regulation of protein complex disassembly | 1 (0.08%) | 1 (0.03%) | 0.367371 | 0.999992 |
| 281 | [GO:0045216](file:///E:\2018-7-3%E7%81%AB%E9%BE%99%E6%9E%9C%E8%BD%AC%E5%BD%95%E7%BB%84%E6%B5%8B%E5%BA%8F\%E5%AE%8C%E6%95%B4%E7%89%88%E6%95%B0%E6%8D%AE\GDR3855-Hylocereus_undulatus_Britt-12-RNAseq_result\4_Function\2_Group_Diff_Function\UP_DOWN\GO\NL-VS-L1.P.html#gene281) | cell-cell junction organization | 1 (0.08%) | 1 (0.03%) | 0.367371 | 0.999992 |
| 282 | [GO:0046368](file:///E:\2018-7-3%E7%81%AB%E9%BE%99%E6%9E%9C%E8%BD%AC%E5%BD%95%E7%BB%84%E6%B5%8B%E5%BA%8F\%E5%AE%8C%E6%95%B4%E7%89%88%E6%95%B0%E6%8D%AE\GDR3855-Hylocereus_undulatus_Britt-12-RNAseq_result\4_Function\2_Group_Diff_Function\UP_DOWN\GO\NL-VS-L1.P.html#gene282) | GDP-L-fucose metabolic process | 1 (0.08%) | 1 (0.03%) | 0.367371 | 0.999992 |
| 283 | [GO:0046471](file:///E:\2018-7-3%E7%81%AB%E9%BE%99%E6%9E%9C%E8%BD%AC%E5%BD%95%E7%BB%84%E6%B5%8B%E5%BA%8F\%E5%AE%8C%E6%95%B4%E7%89%88%E6%95%B0%E6%8D%AE\GDR3855-Hylocereus_undulatus_Britt-12-RNAseq_result\4_Function\2_Group_Diff_Function\UP_DOWN\GO\NL-VS-L1.P.html#gene283) | phosphatidylglycerol metabolic process | 1 (0.08%) | 1 (0.03%) | 0.367371 | 0.999992 |
| 284 | [GO:0046484](file:///E:\2018-7-3%E7%81%AB%E9%BE%99%E6%9E%9C%E8%BD%AC%E5%BD%95%E7%BB%84%E6%B5%8B%E5%BA%8F\%E5%AE%8C%E6%95%B4%E7%89%88%E6%95%B0%E6%8D%AE\GDR3855-Hylocereus_undulatus_Britt-12-RNAseq_result\4_Function\2_Group_Diff_Function\UP_DOWN\GO\NL-VS-L1.P.html#gene284) | oxazole or thiazole metabolic process | 1 (0.08%) | 1 (0.03%) | 0.367371 | 0.999992 |
| 285 | [GO:0048232](file:///E:\2018-7-3%E7%81%AB%E9%BE%99%E6%9E%9C%E8%BD%AC%E5%BD%95%E7%BB%84%E6%B5%8B%E5%BA%8F\%E5%AE%8C%E6%95%B4%E7%89%88%E6%95%B0%E6%8D%AE\GDR3855-Hylocereus_undulatus_Britt-12-RNAseq_result\4_Function\2_Group_Diff_Function\UP_DOWN\GO\NL-VS-L1.P.html#gene285) | male gamete generation | 1 (0.08%) | 1 (0.03%) | 0.367371 | 0.999992 |
| 286 | [GO:0048284](file:///E:\2018-7-3%E7%81%AB%E9%BE%99%E6%9E%9C%E8%BD%AC%E5%BD%95%E7%BB%84%E6%B5%8B%E5%BA%8F\%E5%AE%8C%E6%95%B4%E7%89%88%E6%95%B0%E6%8D%AE\GDR3855-Hylocereus_undulatus_Britt-12-RNAseq_result\4_Function\2_Group_Diff_Function\UP_DOWN\GO\NL-VS-L1.P.html#gene286) | organelle fusion | 1 (0.08%) | 1 (0.03%) | 0.367371 | 0.999992 |
| 287 | [GO:0048366](file:///E:\2018-7-3%E7%81%AB%E9%BE%99%E6%9E%9C%E8%BD%AC%E5%BD%95%E7%BB%84%E6%B5%8B%E5%BA%8F\%E5%AE%8C%E6%95%B4%E7%89%88%E6%95%B0%E6%8D%AE\GDR3855-Hylocereus_undulatus_Britt-12-RNAseq_result\4_Function\2_Group_Diff_Function\UP_DOWN\GO\NL-VS-L1.P.html#gene287) | leaf development | 1 (0.08%) | 1 (0.03%) | 0.367371 | 0.999992 |
| 288 | [GO:0050688](file:///E:\2018-7-3%E7%81%AB%E9%BE%99%E6%9E%9C%E8%BD%AC%E5%BD%95%E7%BB%84%E6%B5%8B%E5%BA%8F\%E5%AE%8C%E6%95%B4%E7%89%88%E6%95%B0%E6%8D%AE\GDR3855-Hylocereus_undulatus_Britt-12-RNAseq_result\4_Function\2_Group_Diff_Function\UP_DOWN\GO\NL-VS-L1.P.html#gene288) | regulation of defense response to virus | 1 (0.08%) | 1 (0.03%) | 0.367371 | 0.999992 |
| 289 | [GO:0050832](file:///E:\2018-7-3%E7%81%AB%E9%BE%99%E6%9E%9C%E8%BD%AC%E5%BD%95%E7%BB%84%E6%B5%8B%E5%BA%8F\%E5%AE%8C%E6%95%B4%E7%89%88%E6%95%B0%E6%8D%AE\GDR3855-Hylocereus_undulatus_Britt-12-RNAseq_result\4_Function\2_Group_Diff_Function\UP_DOWN\GO\NL-VS-L1.P.html#gene289) | defense response to fungus | 1 (0.08%) | 1 (0.03%) | 0.367371 | 0.999992 |
| 290 | [GO:0051129](file:///E:\2018-7-3%E7%81%AB%E9%BE%99%E6%9E%9C%E8%BD%AC%E5%BD%95%E7%BB%84%E6%B5%8B%E5%BA%8F\%E5%AE%8C%E6%95%B4%E7%89%88%E6%95%B0%E6%8D%AE\GDR3855-Hylocereus_undulatus_Britt-12-RNAseq_result\4_Function\2_Group_Diff_Function\UP_DOWN\GO\NL-VS-L1.P.html#gene290) | negative regulation of cellular component organization | 1 (0.08%) | 1 (0.03%) | 0.367371 | 0.999992 |
| 291 | [GO:0051302](file:///E:\2018-7-3%E7%81%AB%E9%BE%99%E6%9E%9C%E8%BD%AC%E5%BD%95%E7%BB%84%E6%B5%8B%E5%BA%8F\%E5%AE%8C%E6%95%B4%E7%89%88%E6%95%B0%E6%8D%AE\GDR3855-Hylocereus_undulatus_Britt-12-RNAseq_result\4_Function\2_Group_Diff_Function\UP_DOWN\GO\NL-VS-L1.P.html#gene291) | regulation of cell division | 1 (0.08%) | 1 (0.03%) | 0.367371 | 0.999992 |
| 292 | [GO:0051494](file:///E:\2018-7-3%E7%81%AB%E9%BE%99%E6%9E%9C%E8%BD%AC%E5%BD%95%E7%BB%84%E6%B5%8B%E5%BA%8F\%E5%AE%8C%E6%95%B4%E7%89%88%E6%95%B0%E6%8D%AE\GDR3855-Hylocereus_undulatus_Britt-12-RNAseq_result\4_Function\2_Group_Diff_Function\UP_DOWN\GO\NL-VS-L1.P.html#gene292) | negative regulation of cytoskeleton organization | 1 (0.08%) | 1 (0.03%) | 0.367371 | 0.999992 |
| 293 | [GO:0051783](file:///E:\2018-7-3%E7%81%AB%E9%BE%99%E6%9E%9C%E8%BD%AC%E5%BD%95%E7%BB%84%E6%B5%8B%E5%BA%8F\%E5%AE%8C%E6%95%B4%E7%89%88%E6%95%B0%E6%8D%AE\GDR3855-Hylocereus_undulatus_Britt-12-RNAseq_result\4_Function\2_Group_Diff_Function\UP_DOWN\GO\NL-VS-L1.P.html#gene293) | regulation of nuclear division | 1 (0.08%) | 1 (0.03%) | 0.367371 | 0.999992 |
| 294 | [GO:0052386](file:///E:\2018-7-3%E7%81%AB%E9%BE%99%E6%9E%9C%E8%BD%AC%E5%BD%95%E7%BB%84%E6%B5%8B%E5%BA%8F\%E5%AE%8C%E6%95%B4%E7%89%88%E6%95%B0%E6%8D%AE\GDR3855-Hylocereus_undulatus_Britt-12-RNAseq_result\4_Function\2_Group_Diff_Function\UP_DOWN\GO\NL-VS-L1.P.html#gene294) | cell wall thickening | 1 (0.08%) | 1 (0.03%) | 0.367371 | 0.999992 |
| 295 | [GO:0070585](file:///E:\2018-7-3%E7%81%AB%E9%BE%99%E6%9E%9C%E8%BD%AC%E5%BD%95%E7%BB%84%E6%B5%8B%E5%BA%8F\%E5%AE%8C%E6%95%B4%E7%89%88%E6%95%B0%E6%8D%AE\GDR3855-Hylocereus_undulatus_Britt-12-RNAseq_result\4_Function\2_Group_Diff_Function\UP_DOWN\GO\NL-VS-L1.P.html#gene295) | protein localization to mitochondrion | 1 (0.08%) | 1 (0.03%) | 0.367371 | 0.999992 |
| 296 | [GO:0072655](file:///E:\2018-7-3%E7%81%AB%E9%BE%99%E6%9E%9C%E8%BD%AC%E5%BD%95%E7%BB%84%E6%B5%8B%E5%BA%8F\%E5%AE%8C%E6%95%B4%E7%89%88%E6%95%B0%E6%8D%AE\GDR3855-Hylocereus_undulatus_Britt-12-RNAseq_result\4_Function\2_Group_Diff_Function\UP_DOWN\GO\NL-VS-L1.P.html#gene296) | establishment of protein localization to mitochondrion | 1 (0.08%) | 1 (0.03%) | 0.367371 | 0.999992 |
| 297 | [GO:0085029](file:///E:\2018-7-3%E7%81%AB%E9%BE%99%E6%9E%9C%E8%BD%AC%E5%BD%95%E7%BB%84%E6%B5%8B%E5%BA%8F\%E5%AE%8C%E6%95%B4%E7%89%88%E6%95%B0%E6%8D%AE\GDR3855-Hylocereus_undulatus_Britt-12-RNAseq_result\4_Function\2_Group_Diff_Function\UP_DOWN\GO\NL-VS-L1.P.html#gene297) | extracellular matrix assembly | 1 (0.08%) | 1 (0.03%) | 0.367371 | 0.999992 |
| 298 | [GO:0090626](file:///E:\2018-7-3%E7%81%AB%E9%BE%99%E6%9E%9C%E8%BD%AC%E5%BD%95%E7%BB%84%E6%B5%8B%E5%BA%8F\%E5%AE%8C%E6%95%B4%E7%89%88%E6%95%B0%E6%8D%AE\GDR3855-Hylocereus_undulatus_Britt-12-RNAseq_result\4_Function\2_Group_Diff_Function\UP_DOWN\GO\NL-VS-L1.P.html#gene298) | plant epidermis morphogenesis | 1 (0.08%) | 1 (0.03%) | 0.367371 | 0.999992 |
| 299 | [GO:1901879](file:///E:\2018-7-3%E7%81%AB%E9%BE%99%E6%9E%9C%E8%BD%AC%E5%BD%95%E7%BB%84%E6%B5%8B%E5%BA%8F\%E5%AE%8C%E6%95%B4%E7%89%88%E6%95%B0%E6%8D%AE\GDR3855-Hylocereus_undulatus_Britt-12-RNAseq_result\4_Function\2_Group_Diff_Function\UP_DOWN\GO\NL-VS-L1.P.html#gene299) | regulation of protein depolymerization | 1 (0.08%) | 1 (0.03%) | 0.367371 | 0.999992 |
| 300 | [GO:1901880](file:///E:\2018-7-3%E7%81%AB%E9%BE%99%E6%9E%9C%E8%BD%AC%E5%BD%95%E7%BB%84%E6%B5%8B%E5%BA%8F\%E5%AE%8C%E6%95%B4%E7%89%88%E6%95%B0%E6%8D%AE\GDR3855-Hylocereus_undulatus_Britt-12-RNAseq_result\4_Function\2_Group_Diff_Function\UP_DOWN\GO\NL-VS-L1.P.html#gene300) | negative regulation of protein depolymerization | 1 (0.08%) | 1 (0.03%) | 0.367371 | 0.999992 |
| 301 | [GO:0051276](file:///E:\2018-7-3%E7%81%AB%E9%BE%99%E6%9E%9C%E8%BD%AC%E5%BD%95%E7%BB%84%E6%B5%8B%E5%BA%8F\%E5%AE%8C%E6%95%B4%E7%89%88%E6%95%B0%E6%8D%AE\GDR3855-Hylocereus_undulatus_Britt-12-RNAseq_result\4_Function\2_Group_Diff_Function\UP_DOWN\GO\NL-VS-L1.P.html#gene301) | chromosome organization | 26 (2.11%) | 66 (1.96%) | 0.369663 | 0.999992 |
| 302 | [GO:0070271](file:///E:\2018-7-3%E7%81%AB%E9%BE%99%E6%9E%9C%E8%BD%AC%E5%BD%95%E7%BB%84%E6%B5%8B%E5%BA%8F\%E5%AE%8C%E6%95%B4%E7%89%88%E6%95%B0%E6%8D%AE\GDR3855-Hylocereus_undulatus_Britt-12-RNAseq_result\4_Function\2_Group_Diff_Function\UP_DOWN\GO\NL-VS-L1.P.html#gene302) | protein complex biogenesis | 18 (1.46%) | 45 (1.34%) | 0.377061 | 0.999992 |
| 303 | [GO:0071396](file:///E:\2018-7-3%E7%81%AB%E9%BE%99%E6%9E%9C%E8%BD%AC%E5%BD%95%E7%BB%84%E6%B5%8B%E5%BA%8F\%E5%AE%8C%E6%95%B4%E7%89%88%E6%95%B0%E6%8D%AE\GDR3855-Hylocereus_undulatus_Britt-12-RNAseq_result\4_Function\2_Group_Diff_Function\UP_DOWN\GO\NL-VS-L1.P.html#gene303) | cellular response to lipid | 5 (0.41%) | 11 (0.33%) | 0.377741 | 0.999992 |
| 304 | [GO:0006650](file:///E:\2018-7-3%E7%81%AB%E9%BE%99%E6%9E%9C%E8%BD%AC%E5%BD%95%E7%BB%84%E6%B5%8B%E5%BA%8F\%E5%AE%8C%E6%95%B4%E7%89%88%E6%95%B0%E6%8D%AE\GDR3855-Hylocereus_undulatus_Britt-12-RNAseq_result\4_Function\2_Group_Diff_Function\UP_DOWN\GO\NL-VS-L1.P.html#gene304) | glycerophospholipid metabolic process | 10 (0.81%) | 24 (0.71%) | 0.379596 | 0.999992 |
| 305 | [GO:0046486](file:///E:\2018-7-3%E7%81%AB%E9%BE%99%E6%9E%9C%E8%BD%AC%E5%BD%95%E7%BB%84%E6%B5%8B%E5%BA%8F\%E5%AE%8C%E6%95%B4%E7%89%88%E6%95%B0%E6%8D%AE\GDR3855-Hylocereus_undulatus_Britt-12-RNAseq_result\4_Function\2_Group_Diff_Function\UP_DOWN\GO\NL-VS-L1.P.html#gene305) | glycerolipid metabolic process | 10 (0.81%) | 24 (0.71%) | 0.379596 | 0.999992 |
| 306 | [GO:0000160](file:///E:\2018-7-3%E7%81%AB%E9%BE%99%E6%9E%9C%E8%BD%AC%E5%BD%95%E7%BB%84%E6%B5%8B%E5%BA%8F\%E5%AE%8C%E6%95%B4%E7%89%88%E6%95%B0%E6%8D%AE\GDR3855-Hylocereus_undulatus_Britt-12-RNAseq_result\4_Function\2_Group_Diff_Function\UP_DOWN\GO\NL-VS-L1.P.html#gene306) | phosphorelay signal transduction system | 3 (0.24%) | 6 (0.18%) | 0.388224 | 0.999992 |
| 307 | [GO:0002252](file:///E:\2018-7-3%E7%81%AB%E9%BE%99%E6%9E%9C%E8%BD%AC%E5%BD%95%E7%BB%84%E6%B5%8B%E5%BA%8F\%E5%AE%8C%E6%95%B4%E7%89%88%E6%95%B0%E6%8D%AE\GDR3855-Hylocereus_undulatus_Britt-12-RNAseq_result\4_Function\2_Group_Diff_Function\UP_DOWN\GO\NL-VS-L1.P.html#gene307) | immune effector process | 3 (0.24%) | 6 (0.18%) | 0.388224 | 0.999992 |
| 308 | [GO:0006413](file:///E:\2018-7-3%E7%81%AB%E9%BE%99%E6%9E%9C%E8%BD%AC%E5%BD%95%E7%BB%84%E6%B5%8B%E5%BA%8F\%E5%AE%8C%E6%95%B4%E7%89%88%E6%95%B0%E6%8D%AE\GDR3855-Hylocereus_undulatus_Britt-12-RNAseq_result\4_Function\2_Group_Diff_Function\UP_DOWN\GO\NL-VS-L1.P.html#gene308) | translational initiation | 3 (0.24%) | 6 (0.18%) | 0.388224 | 0.999992 |
| 309 | [GO:0008064](file:///E:\2018-7-3%E7%81%AB%E9%BE%99%E6%9E%9C%E8%BD%AC%E5%BD%95%E7%BB%84%E6%B5%8B%E5%BA%8F\%E5%AE%8C%E6%95%B4%E7%89%88%E6%95%B0%E6%8D%AE\GDR3855-Hylocereus_undulatus_Britt-12-RNAseq_result\4_Function\2_Group_Diff_Function\UP_DOWN\GO\NL-VS-L1.P.html#gene309) | regulation of actin polymerization or depolymerization | 3 (0.24%) | 6 (0.18%) | 0.388224 | 0.999992 |
| 310 | [GO:0015833](file:///E:\2018-7-3%E7%81%AB%E9%BE%99%E6%9E%9C%E8%BD%AC%E5%BD%95%E7%BB%84%E6%B5%8B%E5%BA%8F\%E5%AE%8C%E6%95%B4%E7%89%88%E6%95%B0%E6%8D%AE\GDR3855-Hylocereus_undulatus_Britt-12-RNAseq_result\4_Function\2_Group_Diff_Function\UP_DOWN\GO\NL-VS-L1.P.html#gene310) | peptide transport | 3 (0.24%) | 6 (0.18%) | 0.388224 | 0.999992 |
| 311 | [GO:0018022](file:///E:\2018-7-3%E7%81%AB%E9%BE%99%E6%9E%9C%E8%BD%AC%E5%BD%95%E7%BB%84%E6%B5%8B%E5%BA%8F\%E5%AE%8C%E6%95%B4%E7%89%88%E6%95%B0%E6%8D%AE\GDR3855-Hylocereus_undulatus_Britt-12-RNAseq_result\4_Function\2_Group_Diff_Function\UP_DOWN\GO\NL-VS-L1.P.html#gene311) | peptidyl-lysine methylation | 3 (0.24%) | 6 (0.18%) | 0.388224 | 0.999992 |
| 312 | [GO:0030832](file:///E:\2018-7-3%E7%81%AB%E9%BE%99%E6%9E%9C%E8%BD%AC%E5%BD%95%E7%BB%84%E6%B5%8B%E5%BA%8F\%E5%AE%8C%E6%95%B4%E7%89%88%E6%95%B0%E6%8D%AE\GDR3855-Hylocereus_undulatus_Britt-12-RNAseq_result\4_Function\2_Group_Diff_Function\UP_DOWN\GO\NL-VS-L1.P.html#gene312) | regulation of actin filament length | 3 (0.24%) | 6 (0.18%) | 0.388224 | 0.999992 |
| 313 | [GO:0032956](file:///E:\2018-7-3%E7%81%AB%E9%BE%99%E6%9E%9C%E8%BD%AC%E5%BD%95%E7%BB%84%E6%B5%8B%E5%BA%8F\%E5%AE%8C%E6%95%B4%E7%89%88%E6%95%B0%E6%8D%AE\GDR3855-Hylocereus_undulatus_Britt-12-RNAseq_result\4_Function\2_Group_Diff_Function\UP_DOWN\GO\NL-VS-L1.P.html#gene313) | regulation of actin cytoskeleton organization | 3 (0.24%) | 6 (0.18%) | 0.388224 | 0.999992 |
| 314 | [GO:0032970](file:///E:\2018-7-3%E7%81%AB%E9%BE%99%E6%9E%9C%E8%BD%AC%E5%BD%95%E7%BB%84%E6%B5%8B%E5%BA%8F\%E5%AE%8C%E6%95%B4%E7%89%88%E6%95%B0%E6%8D%AE\GDR3855-Hylocereus_undulatus_Britt-12-RNAseq_result\4_Function\2_Group_Diff_Function\UP_DOWN\GO\NL-VS-L1.P.html#gene314) | regulation of actin filament-based process | 3 (0.24%) | 6 (0.18%) | 0.388224 | 0.999992 |
| 315 | [GO:0034968](file:///E:\2018-7-3%E7%81%AB%E9%BE%99%E6%9E%9C%E8%BD%AC%E5%BD%95%E7%BB%84%E6%B5%8B%E5%BA%8F\%E5%AE%8C%E6%95%B4%E7%89%88%E6%95%B0%E6%8D%AE\GDR3855-Hylocereus_undulatus_Britt-12-RNAseq_result\4_Function\2_Group_Diff_Function\UP_DOWN\GO\NL-VS-L1.P.html#gene315) | histone lysine methylation | 3 (0.24%) | 6 (0.18%) | 0.388224 | 0.999992 |
| 316 | [GO:0043401](file:///E:\2018-7-3%E7%81%AB%E9%BE%99%E6%9E%9C%E8%BD%AC%E5%BD%95%E7%BB%84%E6%B5%8B%E5%BA%8F\%E5%AE%8C%E6%95%B4%E7%89%88%E6%95%B0%E6%8D%AE\GDR3855-Hylocereus_undulatus_Britt-12-RNAseq_result\4_Function\2_Group_Diff_Function\UP_DOWN\GO\NL-VS-L1.P.html#gene316) | steroid hormone mediated signaling pathway | 3 (0.24%) | 6 (0.18%) | 0.388224 | 0.999992 |
| 317 | [GO:0048437](file:///E:\2018-7-3%E7%81%AB%E9%BE%99%E6%9E%9C%E8%BD%AC%E5%BD%95%E7%BB%84%E6%B5%8B%E5%BA%8F\%E5%AE%8C%E6%95%B4%E7%89%88%E6%95%B0%E6%8D%AE\GDR3855-Hylocereus_undulatus_Britt-12-RNAseq_result\4_Function\2_Group_Diff_Function\UP_DOWN\GO\NL-VS-L1.P.html#gene317) | floral organ development | 3 (0.24%) | 6 (0.18%) | 0.388224 | 0.999992 |
| 318 | [GO:0048528](file:///E:\2018-7-3%E7%81%AB%E9%BE%99%E6%9E%9C%E8%BD%AC%E5%BD%95%E7%BB%84%E6%B5%8B%E5%BA%8F\%E5%AE%8C%E6%95%B4%E7%89%88%E6%95%B0%E6%8D%AE\GDR3855-Hylocereus_undulatus_Britt-12-RNAseq_result\4_Function\2_Group_Diff_Function\UP_DOWN\GO\NL-VS-L1.P.html#gene318) | post-embryonic root development | 3 (0.24%) | 6 (0.18%) | 0.388224 | 0.999992 |
| 319 | [GO:0048545](file:///E:\2018-7-3%E7%81%AB%E9%BE%99%E6%9E%9C%E8%BD%AC%E5%BD%95%E7%BB%84%E6%B5%8B%E5%BA%8F\%E5%AE%8C%E6%95%B4%E7%89%88%E6%95%B0%E6%8D%AE\GDR3855-Hylocereus_undulatus_Britt-12-RNAseq_result\4_Function\2_Group_Diff_Function\UP_DOWN\GO\NL-VS-L1.P.html#gene319) | response to steroid hormone | 3 (0.24%) | 6 (0.18%) | 0.388224 | 0.999992 |
| 320 | [GO:0051239](file:///E:\2018-7-3%E7%81%AB%E9%BE%99%E6%9E%9C%E8%BD%AC%E5%BD%95%E7%BB%84%E6%B5%8B%E5%BA%8F\%E5%AE%8C%E6%95%B4%E7%89%88%E6%95%B0%E6%8D%AE\GDR3855-Hylocereus_undulatus_Britt-12-RNAseq_result\4_Function\2_Group_Diff_Function\UP_DOWN\GO\NL-VS-L1.P.html#gene320) | regulation of multicellular organismal process | 3 (0.24%) | 6 (0.18%) | 0.388224 | 0.999992 |
| 321 | [GO:0051493](file:///E:\2018-7-3%E7%81%AB%E9%BE%99%E6%9E%9C%E8%BD%AC%E5%BD%95%E7%BB%84%E6%B5%8B%E5%BA%8F\%E5%AE%8C%E6%95%B4%E7%89%88%E6%95%B0%E6%8D%AE\GDR3855-Hylocereus_undulatus_Britt-12-RNAseq_result\4_Function\2_Group_Diff_Function\UP_DOWN\GO\NL-VS-L1.P.html#gene321) | regulation of cytoskeleton organization | 3 (0.24%) | 6 (0.18%) | 0.388224 | 0.999992 |
| 322 | [GO:0071383](file:///E:\2018-7-3%E7%81%AB%E9%BE%99%E6%9E%9C%E8%BD%AC%E5%BD%95%E7%BB%84%E6%B5%8B%E5%BA%8F\%E5%AE%8C%E6%95%B4%E7%89%88%E6%95%B0%E6%8D%AE\GDR3855-Hylocereus_undulatus_Britt-12-RNAseq_result\4_Function\2_Group_Diff_Function\UP_DOWN\GO\NL-VS-L1.P.html#gene322) | cellular response to steroid hormone stimulus | 3 (0.24%) | 6 (0.18%) | 0.388224 | 0.999992 |
| 323 | [GO:0071407](file:///E:\2018-7-3%E7%81%AB%E9%BE%99%E6%9E%9C%E8%BD%AC%E5%BD%95%E7%BB%84%E6%B5%8B%E5%BA%8F\%E5%AE%8C%E6%95%B4%E7%89%88%E6%95%B0%E6%8D%AE\GDR3855-Hylocereus_undulatus_Britt-12-RNAseq_result\4_Function\2_Group_Diff_Function\UP_DOWN\GO\NL-VS-L1.P.html#gene323) | cellular response to organic cyclic compound | 3 (0.24%) | 6 (0.18%) | 0.388224 | 0.999992 |
| 324 | [GO:2000026](file:///E:\2018-7-3%E7%81%AB%E9%BE%99%E6%9E%9C%E8%BD%AC%E5%BD%95%E7%BB%84%E6%B5%8B%E5%BA%8F\%E5%AE%8C%E6%95%B4%E7%89%88%E6%95%B0%E6%8D%AE\GDR3855-Hylocereus_undulatus_Britt-12-RNAseq_result\4_Function\2_Group_Diff_Function\UP_DOWN\GO\NL-VS-L1.P.html#gene324) | regulation of multicellular organismal development | 3 (0.24%) | 6 (0.18%) | 0.388224 | 0.999992 |
| 325 | [GO:0009314](file:///E:\2018-7-3%E7%81%AB%E9%BE%99%E6%9E%9C%E8%BD%AC%E5%BD%95%E7%BB%84%E6%B5%8B%E5%BA%8F\%E5%AE%8C%E6%95%B4%E7%89%88%E6%95%B0%E6%8D%AE\GDR3855-Hylocereus_undulatus_Britt-12-RNAseq_result\4_Function\2_Group_Diff_Function\UP_DOWN\GO\NL-VS-L1.P.html#gene325) | response to radiation | 37 (3%) | 96 (2.86%) | 0.392481 | 0.999992 |
| 326 | [GO:0009755](file:///E:\2018-7-3%E7%81%AB%E9%BE%99%E6%9E%9C%E8%BD%AC%E5%BD%95%E7%BB%84%E6%B5%8B%E5%BA%8F\%E5%AE%8C%E6%95%B4%E7%89%88%E6%95%B0%E6%8D%AE\GDR3855-Hylocereus_undulatus_Britt-12-RNAseq_result\4_Function\2_Group_Diff_Function\UP_DOWN\GO\NL-VS-L1.P.html#gene326) | hormone-mediated signaling pathway | 14 (1.13%) | 35 (1.04%) | 0.405011 | 0.999992 |
| 327 | [GO:0032870](file:///E:\2018-7-3%E7%81%AB%E9%BE%99%E6%9E%9C%E8%BD%AC%E5%BD%95%E7%BB%84%E6%B5%8B%E5%BA%8F\%E5%AE%8C%E6%95%B4%E7%89%88%E6%95%B0%E6%8D%AE\GDR3855-Hylocereus_undulatus_Britt-12-RNAseq_result\4_Function\2_Group_Diff_Function\UP_DOWN\GO\NL-VS-L1.P.html#gene327) | cellular response to hormone stimulus | 14 (1.13%) | 35 (1.04%) | 0.405011 | 0.999992 |
| 328 | [GO:0071495](file:///E:\2018-7-3%E7%81%AB%E9%BE%99%E6%9E%9C%E8%BD%AC%E5%BD%95%E7%BB%84%E6%B5%8B%E5%BA%8F\%E5%AE%8C%E6%95%B4%E7%89%88%E6%95%B0%E6%8D%AE\GDR3855-Hylocereus_undulatus_Britt-12-RNAseq_result\4_Function\2_Group_Diff_Function\UP_DOWN\GO\NL-VS-L1.P.html#gene328) | cellular response to endogenous stimulus | 14 (1.13%) | 35 (1.04%) | 0.405011 | 0.999992 |
| 329 | [GO:0006694](file:///E:\2018-7-3%E7%81%AB%E9%BE%99%E6%9E%9C%E8%BD%AC%E5%BD%95%E7%BB%84%E6%B5%8B%E5%BA%8F\%E5%AE%8C%E6%95%B4%E7%89%88%E6%95%B0%E6%8D%AE\GDR3855-Hylocereus_undulatus_Britt-12-RNAseq_result\4_Function\2_Group_Diff_Function\UP_DOWN\GO\NL-VS-L1.P.html#gene329) | steroid biosynthetic process | 6 (0.49%) | 14 (0.42%) | 0.412524 | 0.999992 |
| 330 | [GO:0008202](file:///E:\2018-7-3%E7%81%AB%E9%BE%99%E6%9E%9C%E8%BD%AC%E5%BD%95%E7%BB%84%E6%B5%8B%E5%BA%8F\%E5%AE%8C%E6%95%B4%E7%89%88%E6%95%B0%E6%8D%AE\GDR3855-Hylocereus_undulatus_Britt-12-RNAseq_result\4_Function\2_Group_Diff_Function\UP_DOWN\GO\NL-VS-L1.P.html#gene330) | steroid metabolic process | 6 (0.49%) | 14 (0.42%) | 0.412524 | 0.999992 |
| 331 | [GO:0009812](file:///E:\2018-7-3%E7%81%AB%E9%BE%99%E6%9E%9C%E8%BD%AC%E5%BD%95%E7%BB%84%E6%B5%8B%E5%BA%8F\%E5%AE%8C%E6%95%B4%E7%89%88%E6%95%B0%E6%8D%AE\GDR3855-Hylocereus_undulatus_Britt-12-RNAseq_result\4_Function\2_Group_Diff_Function\UP_DOWN\GO\NL-VS-L1.P.html#gene331) | flavonoid metabolic process | 6 (0.49%) | 14 (0.42%) | 0.412524 | 0.999992 |
| 332 | [GO:0043094](file:///E:\2018-7-3%E7%81%AB%E9%BE%99%E6%9E%9C%E8%BD%AC%E5%BD%95%E7%BB%84%E6%B5%8B%E5%BA%8F\%E5%AE%8C%E6%95%B4%E7%89%88%E6%95%B0%E6%8D%AE\GDR3855-Hylocereus_undulatus_Britt-12-RNAseq_result\4_Function\2_Group_Diff_Function\UP_DOWN\GO\NL-VS-L1.P.html#gene332) | cellular metabolic compound salvage | 6 (0.49%) | 14 (0.42%) | 0.412524 | 0.999992 |
| 333 | [GO:0048511](file:///E:\2018-7-3%E7%81%AB%E9%BE%99%E6%9E%9C%E8%BD%AC%E5%BD%95%E7%BB%84%E6%B5%8B%E5%BA%8F\%E5%AE%8C%E6%95%B4%E7%89%88%E6%95%B0%E6%8D%AE\GDR3855-Hylocereus_undulatus_Britt-12-RNAseq_result\4_Function\2_Group_Diff_Function\UP_DOWN\GO\NL-VS-L1.P.html#gene333) | rhythmic process | 6 (0.49%) | 14 (0.42%) | 0.412524 | 0.999992 |
| 334 | [GO:0071669](file:///E:\2018-7-3%E7%81%AB%E9%BE%99%E6%9E%9C%E8%BD%AC%E5%BD%95%E7%BB%84%E6%B5%8B%E5%BA%8F\%E5%AE%8C%E6%95%B4%E7%89%88%E6%95%B0%E6%8D%AE\GDR3855-Hylocereus_undulatus_Britt-12-RNAseq_result\4_Function\2_Group_Diff_Function\UP_DOWN\GO\NL-VS-L1.P.html#gene334) | plant-type cell wall organization or biogenesis | 6 (0.49%) | 14 (0.42%) | 0.412524 | 0.999992 |
| 335 | [GO:1902578](file:///E:\2018-7-3%E7%81%AB%E9%BE%99%E6%9E%9C%E8%BD%AC%E5%BD%95%E7%BB%84%E6%B5%8B%E5%BA%8F\%E5%AE%8C%E6%95%B4%E7%89%88%E6%95%B0%E6%8D%AE\GDR3855-Hylocereus_undulatus_Britt-12-RNAseq_result\4_Function\2_Group_Diff_Function\UP_DOWN\GO\NL-VS-L1.P.html#gene335) | single-organism localization | 122 (9.89%) | 326 (9.71%) | 0.415242 | 0.999992 |
| 336 | [GO:0009639](file:///E:\2018-7-3%E7%81%AB%E9%BE%99%E6%9E%9C%E8%BD%AC%E5%BD%95%E7%BB%84%E6%B5%8B%E5%BA%8F\%E5%AE%8C%E6%95%B4%E7%89%88%E6%95%B0%E6%8D%AE\GDR3855-Hylocereus_undulatus_Britt-12-RNAseq_result\4_Function\2_Group_Diff_Function\UP_DOWN\GO\NL-VS-L1.P.html#gene336) | response to red or far red light | 9 (0.73%) | 22 (0.65%) | 0.419287 | 0.999992 |
| 337 | [GO:0016568](file:///E:\2018-7-3%E7%81%AB%E9%BE%99%E6%9E%9C%E8%BD%AC%E5%BD%95%E7%BB%84%E6%B5%8B%E5%BA%8F\%E5%AE%8C%E6%95%B4%E7%89%88%E6%95%B0%E6%8D%AE\GDR3855-Hylocereus_undulatus_Britt-12-RNAseq_result\4_Function\2_Group_Diff_Function\UP_DOWN\GO\NL-VS-L1.P.html#gene337) | chromatin modification | 9 (0.73%) | 22 (0.65%) | 0.419287 | 0.999992 |
| 338 | [GO:0006631](file:///E:\2018-7-3%E7%81%AB%E9%BE%99%E6%9E%9C%E8%BD%AC%E5%BD%95%E7%BB%84%E6%B5%8B%E5%BA%8F\%E5%AE%8C%E6%95%B4%E7%89%88%E6%95%B0%E6%8D%AE\GDR3855-Hylocereus_undulatus_Britt-12-RNAseq_result\4_Function\2_Group_Diff_Function\UP_DOWN\GO\NL-VS-L1.P.html#gene338) | fatty acid metabolic process | 18 (1.46%) | 46 (1.37%) | 0.421668 | 0.999992 |
| 339 | [GO:0006818](file:///E:\2018-7-3%E7%81%AB%E9%BE%99%E6%9E%9C%E8%BD%AC%E5%BD%95%E7%BB%84%E6%B5%8B%E5%BA%8F\%E5%AE%8C%E6%95%B4%E7%89%88%E6%95%B0%E6%8D%AE\GDR3855-Hylocereus_undulatus_Britt-12-RNAseq_result\4_Function\2_Group_Diff_Function\UP_DOWN\GO\NL-VS-L1.P.html#gene339) | hydrogen transport | 18 (1.46%) | 46 (1.37%) | 0.421668 | 0.999992 |
| 340 | [GO:0010035](file:///E:\2018-7-3%E7%81%AB%E9%BE%99%E6%9E%9C%E8%BD%AC%E5%BD%95%E7%BB%84%E6%B5%8B%E5%BA%8F\%E5%AE%8C%E6%95%B4%E7%89%88%E6%95%B0%E6%8D%AE\GDR3855-Hylocereus_undulatus_Britt-12-RNAseq_result\4_Function\2_Group_Diff_Function\UP_DOWN\GO\NL-VS-L1.P.html#gene340) | response to inorganic substance | 28 (2.27%) | 73 (2.17%) | 0.429621 | 0.999992 |
| 341 | [GO:0002682](file:///E:\2018-7-3%E7%81%AB%E9%BE%99%E6%9E%9C%E8%BD%AC%E5%BD%95%E7%BB%84%E6%B5%8B%E5%BA%8F\%E5%AE%8C%E6%95%B4%E7%89%88%E6%95%B0%E6%8D%AE\GDR3855-Hylocereus_undulatus_Britt-12-RNAseq_result\4_Function\2_Group_Diff_Function\UP_DOWN\GO\NL-VS-L1.P.html#gene341) | regulation of immune system process | 4 (0.32%) | 9 (0.27%) | 0.434892 | 0.999992 |
| 342 | [GO:0006090](file:///E:\2018-7-3%E7%81%AB%E9%BE%99%E6%9E%9C%E8%BD%AC%E5%BD%95%E7%BB%84%E6%B5%8B%E5%BA%8F\%E5%AE%8C%E6%95%B4%E7%89%88%E6%95%B0%E6%8D%AE\GDR3855-Hylocereus_undulatus_Britt-12-RNAseq_result\4_Function\2_Group_Diff_Function\UP_DOWN\GO\NL-VS-L1.P.html#gene342) | pyruvate metabolic process | 4 (0.32%) | 9 (0.27%) | 0.434892 | 0.999992 |
| 343 | [GO:0007389](file:///E:\2018-7-3%E7%81%AB%E9%BE%99%E6%9E%9C%E8%BD%AC%E5%BD%95%E7%BB%84%E6%B5%8B%E5%BA%8F\%E5%AE%8C%E6%95%B4%E7%89%88%E6%95%B0%E6%8D%AE\GDR3855-Hylocereus_undulatus_Britt-12-RNAseq_result\4_Function\2_Group_Diff_Function\UP_DOWN\GO\NL-VS-L1.P.html#gene343) | pattern specification process | 4 (0.32%) | 9 (0.27%) | 0.434892 | 0.999992 |
| 344 | [GO:0009072](file:///E:\2018-7-3%E7%81%AB%E9%BE%99%E6%9E%9C%E8%BD%AC%E5%BD%95%E7%BB%84%E6%B5%8B%E5%BA%8F\%E5%AE%8C%E6%95%B4%E7%89%88%E6%95%B0%E6%8D%AE\GDR3855-Hylocereus_undulatus_Britt-12-RNAseq_result\4_Function\2_Group_Diff_Function\UP_DOWN\GO\NL-VS-L1.P.html#gene344) | aromatic amino acid family metabolic process | 4 (0.32%) | 9 (0.27%) | 0.434892 | 0.999992 |
| 345 | [GO:0009658](file:///E:\2018-7-3%E7%81%AB%E9%BE%99%E6%9E%9C%E8%BD%AC%E5%BD%95%E7%BB%84%E6%B5%8B%E5%BA%8F\%E5%AE%8C%E6%95%B4%E7%89%88%E6%95%B0%E6%8D%AE\GDR3855-Hylocereus_undulatus_Britt-12-RNAseq_result\4_Function\2_Group_Diff_Function\UP_DOWN\GO\NL-VS-L1.P.html#gene345) | chloroplast organization | 4 (0.32%) | 9 (0.27%) | 0.434892 | 0.999992 |
| 346 | [GO:0009664](file:///E:\2018-7-3%E7%81%AB%E9%BE%99%E6%9E%9C%E8%BD%AC%E5%BD%95%E7%BB%84%E6%B5%8B%E5%BA%8F\%E5%AE%8C%E6%95%B4%E7%89%88%E6%95%B0%E6%8D%AE\GDR3855-Hylocereus_undulatus_Britt-12-RNAseq_result\4_Function\2_Group_Diff_Function\UP_DOWN\GO\NL-VS-L1.P.html#gene346) | plant-type cell wall organization | 4 (0.32%) | 9 (0.27%) | 0.434892 | 0.999992 |
| 347 | [GO:0015748](file:///E:\2018-7-3%E7%81%AB%E9%BE%99%E6%9E%9C%E8%BD%AC%E5%BD%95%E7%BB%84%E6%B5%8B%E5%BA%8F\%E5%AE%8C%E6%95%B4%E7%89%88%E6%95%B0%E6%8D%AE\GDR3855-Hylocereus_undulatus_Britt-12-RNAseq_result\4_Function\2_Group_Diff_Function\UP_DOWN\GO\NL-VS-L1.P.html#gene347) | organophosphate ester transport | 4 (0.32%) | 9 (0.27%) | 0.434892 | 0.999992 |
| 348 | [GO:0031347](file:///E:\2018-7-3%E7%81%AB%E9%BE%99%E6%9E%9C%E8%BD%AC%E5%BD%95%E7%BB%84%E6%B5%8B%E5%BA%8F\%E5%AE%8C%E6%95%B4%E7%89%88%E6%95%B0%E6%8D%AE\GDR3855-Hylocereus_undulatus_Britt-12-RNAseq_result\4_Function\2_Group_Diff_Function\UP_DOWN\GO\NL-VS-L1.P.html#gene348) | regulation of defense response | 4 (0.32%) | 9 (0.27%) | 0.434892 | 0.999992 |
| 349 | [GO:0034285](file:///E:\2018-7-3%E7%81%AB%E9%BE%99%E6%9E%9C%E8%BD%AC%E5%BD%95%E7%BB%84%E6%B5%8B%E5%BA%8F\%E5%AE%8C%E6%95%B4%E7%89%88%E6%95%B0%E6%8D%AE\GDR3855-Hylocereus_undulatus_Britt-12-RNAseq_result\4_Function\2_Group_Diff_Function\UP_DOWN\GO\NL-VS-L1.P.html#gene349) | response to disaccharide | 4 (0.32%) | 9 (0.27%) | 0.434892 | 0.999992 |
| 350 | [GO:0048588](file:///E:\2018-7-3%E7%81%AB%E9%BE%99%E6%9E%9C%E8%BD%AC%E5%BD%95%E7%BB%84%E6%B5%8B%E5%BA%8F\%E5%AE%8C%E6%95%B4%E7%89%88%E6%95%B0%E6%8D%AE\GDR3855-Hylocereus_undulatus_Britt-12-RNAseq_result\4_Function\2_Group_Diff_Function\UP_DOWN\GO\NL-VS-L1.P.html#gene350) | developmental cell growth | 4 (0.32%) | 9 (0.27%) | 0.434892 | 0.999992 |
| 351 | [GO:1901607](file:///E:\2018-7-3%E7%81%AB%E9%BE%99%E6%9E%9C%E8%BD%AC%E5%BD%95%E7%BB%84%E6%B5%8B%E5%BA%8F\%E5%AE%8C%E6%95%B4%E7%89%88%E6%95%B0%E6%8D%AE\GDR3855-Hylocereus_undulatus_Britt-12-RNAseq_result\4_Function\2_Group_Diff_Function\UP_DOWN\GO\NL-VS-L1.P.html#gene351) | alpha-amino acid biosynthetic process | 4 (0.32%) | 9 (0.27%) | 0.434892 | 0.999992 |
| 352 | [GO:0002376](file:///E:\2018-7-3%E7%81%AB%E9%BE%99%E6%9E%9C%E8%BD%AC%E5%BD%95%E7%BB%84%E6%B5%8B%E5%BA%8F\%E5%AE%8C%E6%95%B4%E7%89%88%E6%95%B0%E6%8D%AE\GDR3855-Hylocereus_undulatus_Britt-12-RNAseq_result\4_Function\2_Group_Diff_Function\UP_DOWN\GO\NL-VS-L1.P.html#gene352) | immune system process | 13 (1.05%) | 33 (0.98%) | 0.439596 | 0.999992 |
| 353 | [GO:0006644](file:///E:\2018-7-3%E7%81%AB%E9%BE%99%E6%9E%9C%E8%BD%AC%E5%BD%95%E7%BB%84%E6%B5%8B%E5%BA%8F\%E5%AE%8C%E6%95%B4%E7%89%88%E6%95%B0%E6%8D%AE\GDR3855-Hylocereus_undulatus_Britt-12-RNAseq_result\4_Function\2_Group_Diff_Function\UP_DOWN\GO\NL-VS-L1.P.html#gene353) | phospholipid metabolic process | 13 (1.05%) | 33 (0.98%) | 0.439596 | 0.999992 |
| 354 | [GO:0006260](file:///E:\2018-7-3%E7%81%AB%E9%BE%99%E6%9E%9C%E8%BD%AC%E5%BD%95%E7%BB%84%E6%B5%8B%E5%BA%8F\%E5%AE%8C%E6%95%B4%E7%89%88%E6%95%B0%E6%8D%AE\GDR3855-Hylocereus_undulatus_Britt-12-RNAseq_result\4_Function\2_Group_Diff_Function\UP_DOWN\GO\NL-VS-L1.P.html#gene354) | DNA replication | 7 (0.57%) | 17 (0.51%) | 0.440368 | 0.999992 |
| 355 | [GO:0022402](file:///E:\2018-7-3%E7%81%AB%E9%BE%99%E6%9E%9C%E8%BD%AC%E5%BD%95%E7%BB%84%E6%B5%8B%E5%BA%8F\%E5%AE%8C%E6%95%B4%E7%89%88%E6%95%B0%E6%8D%AE\GDR3855-Hylocereus_undulatus_Britt-12-RNAseq_result\4_Function\2_Group_Diff_Function\UP_DOWN\GO\NL-VS-L1.P.html#gene355) | cell cycle process | 7 (0.57%) | 17 (0.51%) | 0.440368 | 0.999992 |
| 356 | [GO:0009657](file:///E:\2018-7-3%E7%81%AB%E9%BE%99%E6%9E%9C%E8%BD%AC%E5%BD%95%E7%BB%84%E6%B5%8B%E5%BA%8F\%E5%AE%8C%E6%95%B4%E7%89%88%E6%95%B0%E6%8D%AE\GDR3855-Hylocereus_undulatus_Britt-12-RNAseq_result\4_Function\2_Group_Diff_Function\UP_DOWN\GO\NL-VS-L1.P.html#gene356) | plastid organization | 10 (0.81%) | 25 (0.74%) | 0.440721 | 0.999992 |
| 357 | [GO:0006790](file:///E:\2018-7-3%E7%81%AB%E9%BE%99%E6%9E%9C%E8%BD%AC%E5%BD%95%E7%BB%84%E6%B5%8B%E5%BA%8F\%E5%AE%8C%E6%95%B4%E7%89%88%E6%95%B0%E6%8D%AE\GDR3855-Hylocereus_undulatus_Britt-12-RNAseq_result\4_Function\2_Group_Diff_Function\UP_DOWN\GO\NL-VS-L1.P.html#gene357) | sulfur compound metabolic process | 20 (1.62%) | 52 (1.55%) | 0.449400 | 0.999992 |
| 358 | [GO:0006461](file:///E:\2018-7-3%E7%81%AB%E9%BE%99%E6%9E%9C%E8%BD%AC%E5%BD%95%E7%BB%84%E6%B5%8B%E5%BA%8F\%E5%AE%8C%E6%95%B4%E7%89%88%E6%95%B0%E6%8D%AE\GDR3855-Hylocereus_undulatus_Britt-12-RNAseq_result\4_Function\2_Group_Diff_Function\UP_DOWN\GO\NL-VS-L1.P.html#gene358) | protein complex assembly | 17 (1.38%) | 44 (1.31%) | 0.452641 | 0.999992 |
| 359 | [GO:0006812](file:///E:\2018-7-3%E7%81%AB%E9%BE%99%E6%9E%9C%E8%BD%AC%E5%BD%95%E7%BB%84%E6%B5%8B%E5%BA%8F\%E5%AE%8C%E6%95%B4%E7%89%88%E6%95%B0%E6%8D%AE\GDR3855-Hylocereus_undulatus_Britt-12-RNAseq_result\4_Function\2_Group_Diff_Function\UP_DOWN\GO\NL-VS-L1.P.html#gene359) | cation transport | 47 (3.81%) | 125 (3.72%) | 0.453429 | 0.999992 |
| 360 | [GO:0051179](file:///E:\2018-7-3%E7%81%AB%E9%BE%99%E6%9E%9C%E8%BD%AC%E5%BD%95%E7%BB%84%E6%B5%8B%E5%BA%8F\%E5%AE%8C%E6%95%B4%E7%89%88%E6%95%B0%E6%8D%AE\GDR3855-Hylocereus_undulatus_Britt-12-RNAseq_result\4_Function\2_Group_Diff_Function\UP_DOWN\GO\NL-VS-L1.P.html#gene360) | localization | 190 (15.4%) | 513 (15.27%) | 0.457655 | 0.999992 |
| 361 | [GO:0051234](file:///E:\2018-7-3%E7%81%AB%E9%BE%99%E6%9E%9C%E8%BD%AC%E5%BD%95%E7%BB%84%E6%B5%8B%E5%BA%8F\%E5%AE%8C%E6%95%B4%E7%89%88%E6%95%B0%E6%8D%AE\GDR3855-Hylocereus_undulatus_Britt-12-RNAseq_result\4_Function\2_Group_Diff_Function\UP_DOWN\GO\NL-VS-L1.P.html#gene361) | establishment of localization | 185 (14.99%) | 500 (14.89%) | 0.466139 | 0.999992 |
| 362 | [GO:0065003](file:///E:\2018-7-3%E7%81%AB%E9%BE%99%E6%9E%9C%E8%BD%AC%E5%BD%95%E7%BB%84%E6%B5%8B%E5%BA%8F\%E5%AE%8C%E6%95%B4%E7%89%88%E6%95%B0%E6%8D%AE\GDR3855-Hylocereus_undulatus_Britt-12-RNAseq_result\4_Function\2_Group_Diff_Function\UP_DOWN\GO\NL-VS-L1.P.html#gene362) | macromolecular complex assembly | 18 (1.46%) | 47 (1.4%) | 0.466453 | 0.999992 |
| 363 | [GO:0009699](file:///E:\2018-7-3%E7%81%AB%E9%BE%99%E6%9E%9C%E8%BD%AC%E5%BD%95%E7%BB%84%E6%B5%8B%E5%BA%8F\%E5%AE%8C%E6%95%B4%E7%89%88%E6%95%B0%E6%8D%AE\GDR3855-Hylocereus_undulatus_Britt-12-RNAseq_result\4_Function\2_Group_Diff_Function\UP_DOWN\GO\NL-VS-L1.P.html#gene363) | phenylpropanoid biosynthetic process | 5 (0.41%) | 12 (0.36%) | 0.467444 | 0.999992 |
| 364 | [GO:0035966](file:///E:\2018-7-3%E7%81%AB%E9%BE%99%E6%9E%9C%E8%BD%AC%E5%BD%95%E7%BB%84%E6%B5%8B%E5%BA%8F\%E5%AE%8C%E6%95%B4%E7%89%88%E6%95%B0%E6%8D%AE\GDR3855-Hylocereus_undulatus_Britt-12-RNAseq_result\4_Function\2_Group_Diff_Function\UP_DOWN\GO\NL-VS-L1.P.html#gene364) | response to topologically incorrect protein | 5 (0.41%) | 12 (0.36%) | 0.467444 | 0.999992 |
| 365 | [GO:0000281](file:///E:\2018-7-3%E7%81%AB%E9%BE%99%E6%9E%9C%E8%BD%AC%E5%BD%95%E7%BB%84%E6%B5%8B%E5%BA%8F\%E5%AE%8C%E6%95%B4%E7%89%88%E6%95%B0%E6%8D%AE\GDR3855-Hylocereus_undulatus_Britt-12-RNAseq_result\4_Function\2_Group_Diff_Function\UP_DOWN\GO\NL-VS-L1.P.html#gene365) | mitotic cytokinesis | 2 (0.16%) | 4 (0.12%) | 0.467792 | 0.999992 |
| 366 | [GO:0000911](file:///E:\2018-7-3%E7%81%AB%E9%BE%99%E6%9E%9C%E8%BD%AC%E5%BD%95%E7%BB%84%E6%B5%8B%E5%BA%8F\%E5%AE%8C%E6%95%B4%E7%89%88%E6%95%B0%E6%8D%AE\GDR3855-Hylocereus_undulatus_Britt-12-RNAseq_result\4_Function\2_Group_Diff_Function\UP_DOWN\GO\NL-VS-L1.P.html#gene366) | cytokinesis by cell plate formation | 2 (0.16%) | 4 (0.12%) | 0.467792 | 0.999992 |
| 367 | [GO:0006862](file:///E:\2018-7-3%E7%81%AB%E9%BE%99%E6%9E%9C%E8%BD%AC%E5%BD%95%E7%BB%84%E6%B5%8B%E5%BA%8F\%E5%AE%8C%E6%95%B4%E7%89%88%E6%95%B0%E6%8D%AE\GDR3855-Hylocereus_undulatus_Britt-12-RNAseq_result\4_Function\2_Group_Diff_Function\UP_DOWN\GO\NL-VS-L1.P.html#gene367) | nucleotide transport | 2 (0.16%) | 4 (0.12%) | 0.467792 | 0.999992 |
| 368 | [GO:0006865](file:///E:\2018-7-3%E7%81%AB%E9%BE%99%E6%9E%9C%E8%BD%AC%E5%BD%95%E7%BB%84%E6%B5%8B%E5%BA%8F\%E5%AE%8C%E6%95%B4%E7%89%88%E6%95%B0%E6%8D%AE\GDR3855-Hylocereus_undulatus_Britt-12-RNAseq_result\4_Function\2_Group_Diff_Function\UP_DOWN\GO\NL-VS-L1.P.html#gene368) | amino acid transport | 2 (0.16%) | 4 (0.12%) | 0.467792 | 0.999992 |
| 369 | [GO:0008037](file:///E:\2018-7-3%E7%81%AB%E9%BE%99%E6%9E%9C%E8%BD%AC%E5%BD%95%E7%BB%84%E6%B5%8B%E5%BA%8F\%E5%AE%8C%E6%95%B4%E7%89%88%E6%95%B0%E6%8D%AE\GDR3855-Hylocereus_undulatus_Britt-12-RNAseq_result\4_Function\2_Group_Diff_Function\UP_DOWN\GO\NL-VS-L1.P.html#gene369) | cell recognition | 2 (0.16%) | 4 (0.12%) | 0.467792 | 0.999992 |
| 370 | [GO:0009081](file:///E:\2018-7-3%E7%81%AB%E9%BE%99%E6%9E%9C%E8%BD%AC%E5%BD%95%E7%BB%84%E6%B5%8B%E5%BA%8F\%E5%AE%8C%E6%95%B4%E7%89%88%E6%95%B0%E6%8D%AE\GDR3855-Hylocereus_undulatus_Britt-12-RNAseq_result\4_Function\2_Group_Diff_Function\UP_DOWN\GO\NL-VS-L1.P.html#gene370) | branched-chain amino acid metabolic process | 2 (0.16%) | 4 (0.12%) | 0.467792 | 0.999992 |
| 371 | [GO:0009555](file:///E:\2018-7-3%E7%81%AB%E9%BE%99%E6%9E%9C%E8%BD%AC%E5%BD%95%E7%BB%84%E6%B5%8B%E5%BA%8F\%E5%AE%8C%E6%95%B4%E7%89%88%E6%95%B0%E6%8D%AE\GDR3855-Hylocereus_undulatus_Britt-12-RNAseq_result\4_Function\2_Group_Diff_Function\UP_DOWN\GO\NL-VS-L1.P.html#gene371) | pollen development | 2 (0.16%) | 4 (0.12%) | 0.467792 | 0.999992 |
| 372 | [GO:0009933](file:///E:\2018-7-3%E7%81%AB%E9%BE%99%E6%9E%9C%E8%BD%AC%E5%BD%95%E7%BB%84%E6%B5%8B%E5%BA%8F\%E5%AE%8C%E6%95%B4%E7%89%88%E6%95%B0%E6%8D%AE\GDR3855-Hylocereus_undulatus_Britt-12-RNAseq_result\4_Function\2_Group_Diff_Function\UP_DOWN\GO\NL-VS-L1.P.html#gene372) | meristem structural organization | 2 (0.16%) | 4 (0.12%) | 0.467792 | 0.999992 |
| 373 | [GO:0010608](file:///E:\2018-7-3%E7%81%AB%E9%BE%99%E6%9E%9C%E8%BD%AC%E5%BD%95%E7%BB%84%E6%B5%8B%E5%BA%8F\%E5%AE%8C%E6%95%B4%E7%89%88%E6%95%B0%E6%8D%AE\GDR3855-Hylocereus_undulatus_Britt-12-RNAseq_result\4_Function\2_Group_Diff_Function\UP_DOWN\GO\NL-VS-L1.P.html#gene373) | posttranscriptional regulation of gene expression | 2 (0.16%) | 4 (0.12%) | 0.467792 | 0.999992 |
| 374 | [GO:0015696](file:///E:\2018-7-3%E7%81%AB%E9%BE%99%E6%9E%9C%E8%BD%AC%E5%BD%95%E7%BB%84%E6%B5%8B%E5%BA%8F\%E5%AE%8C%E6%95%B4%E7%89%88%E6%95%B0%E6%8D%AE\GDR3855-Hylocereus_undulatus_Britt-12-RNAseq_result\4_Function\2_Group_Diff_Function\UP_DOWN\GO\NL-VS-L1.P.html#gene374) | ammonium transport | 2 (0.16%) | 4 (0.12%) | 0.467792 | 0.999992 |
| 375 | [GO:0032506](file:///E:\2018-7-3%E7%81%AB%E9%BE%99%E6%9E%9C%E8%BD%AC%E5%BD%95%E7%BB%84%E6%B5%8B%E5%BA%8F\%E5%AE%8C%E6%95%B4%E7%89%88%E6%95%B0%E6%8D%AE\GDR3855-Hylocereus_undulatus_Britt-12-RNAseq_result\4_Function\2_Group_Diff_Function\UP_DOWN\GO\NL-VS-L1.P.html#gene375) | cytokinetic process | 2 (0.16%) | 4 (0.12%) | 0.467792 | 0.999992 |
| 376 | [GO:0044403](file:///E:\2018-7-3%E7%81%AB%E9%BE%99%E6%9E%9C%E8%BD%AC%E5%BD%95%E7%BB%84%E6%B5%8B%E5%BA%8F\%E5%AE%8C%E6%95%B4%E7%89%88%E6%95%B0%E6%8D%AE\GDR3855-Hylocereus_undulatus_Britt-12-RNAseq_result\4_Function\2_Group_Diff_Function\UP_DOWN\GO\NL-VS-L1.P.html#gene376) | symbiosis, encompassing mutualism through parasitism | 2 (0.16%) | 4 (0.12%) | 0.467792 | 0.999992 |
| 377 | [GO:0044419](file:///E:\2018-7-3%E7%81%AB%E9%BE%99%E6%9E%9C%E8%BD%AC%E5%BD%95%E7%BB%84%E6%B5%8B%E5%BA%8F\%E5%AE%8C%E6%95%B4%E7%89%88%E6%95%B0%E6%8D%AE\GDR3855-Hylocereus_undulatus_Britt-12-RNAseq_result\4_Function\2_Group_Diff_Function\UP_DOWN\GO\NL-VS-L1.P.html#gene377) | interspecies interaction between organisms | 2 (0.16%) | 4 (0.12%) | 0.467792 | 0.999992 |
| 378 | [GO:0048532](file:///E:\2018-7-3%E7%81%AB%E9%BE%99%E6%9E%9C%E8%BD%AC%E5%BD%95%E7%BB%84%E6%B5%8B%E5%BA%8F\%E5%AE%8C%E6%95%B4%E7%89%88%E6%95%B0%E6%8D%AE\GDR3855-Hylocereus_undulatus_Britt-12-RNAseq_result\4_Function\2_Group_Diff_Function\UP_DOWN\GO\NL-VS-L1.P.html#gene378) | anatomical structure arrangement | 2 (0.16%) | 4 (0.12%) | 0.467792 | 0.999992 |
| 379 | [GO:1902410](file:///E:\2018-7-3%E7%81%AB%E9%BE%99%E6%9E%9C%E8%BD%AC%E5%BD%95%E7%BB%84%E6%B5%8B%E5%BA%8F\%E5%AE%8C%E6%95%B4%E7%89%88%E6%95%B0%E6%8D%AE\GDR3855-Hylocereus_undulatus_Britt-12-RNAseq_result\4_Function\2_Group_Diff_Function\UP_DOWN\GO\NL-VS-L1.P.html#gene379) | mitotic cytokinetic process | 2 (0.16%) | 4 (0.12%) | 0.467792 | 0.999992 |
| 380 | [GO:0010038](file:///E:\2018-7-3%E7%81%AB%E9%BE%99%E6%9E%9C%E8%BD%AC%E5%BD%95%E7%BB%84%E6%B5%8B%E5%BA%8F\%E5%AE%8C%E6%95%B4%E7%89%88%E6%95%B0%E6%8D%AE\GDR3855-Hylocereus_undulatus_Britt-12-RNAseq_result\4_Function\2_Group_Diff_Function\UP_DOWN\GO\NL-VS-L1.P.html#gene380) | response to metal ion | 25 (2.03%) | 66 (1.96%) | 0.469603 | 0.999992 |
| 381 | [GO:0044765](file:///E:\2018-7-3%E7%81%AB%E9%BE%99%E6%9E%9C%E8%BD%AC%E5%BD%95%E7%BB%84%E6%B5%8B%E5%BA%8F\%E5%AE%8C%E6%95%B4%E7%89%88%E6%95%B0%E6%8D%AE\GDR3855-Hylocereus_undulatus_Britt-12-RNAseq_result\4_Function\2_Group_Diff_Function\UP_DOWN\GO\NL-VS-L1.P.html#gene381) | single-organism transport | 119 (9.64%) | 321 (9.56%) | 0.470433 | 0.999992 |
| 382 | [GO:0015672](file:///E:\2018-7-3%E7%81%AB%E9%BE%99%E6%9E%9C%E8%BD%AC%E5%BD%95%E7%BB%84%E6%B5%8B%E5%BA%8F\%E5%AE%8C%E6%95%B4%E7%89%88%E6%95%B0%E6%8D%AE\GDR3855-Hylocereus_undulatus_Britt-12-RNAseq_result\4_Function\2_Group_Diff_Function\UP_DOWN\GO\NL-VS-L1.P.html#gene382) | monovalent inorganic cation transport | 19 (1.54%) | 50 (1.49%) | 0.479441 | 0.999992 |
| 383 | [GO:0032787](file:///E:\2018-7-3%E7%81%AB%E9%BE%99%E6%9E%9C%E8%BD%AC%E5%BD%95%E7%BB%84%E6%B5%8B%E5%BA%8F\%E5%AE%8C%E6%95%B4%E7%89%88%E6%95%B0%E6%8D%AE\GDR3855-Hylocereus_undulatus_Britt-12-RNAseq_result\4_Function\2_Group_Diff_Function\UP_DOWN\GO\NL-VS-L1.P.html#gene383) | monocarboxylic acid metabolic process | 26 (2.11%) | 69 (2.05%) | 0.480498 | 0.999992 |
| 384 | [GO:0071310](file:///E:\2018-7-3%E7%81%AB%E9%BE%99%E6%9E%9C%E8%BD%AC%E5%BD%95%E7%BB%84%E6%B5%8B%E5%BA%8F\%E5%AE%8C%E6%95%B4%E7%89%88%E6%95%B0%E6%8D%AE\GDR3855-Hylocereus_undulatus_Britt-12-RNAseq_result\4_Function\2_Group_Diff_Function\UP_DOWN\GO\NL-VS-L1.P.html#gene384) | cellular response to organic substance | 16 (1.3%) | 42 (1.25%) | 0.485377 | 0.999992 |
| 385 | [GO:0009668](file:///E:\2018-7-3%E7%81%AB%E9%BE%99%E6%9E%9C%E8%BD%AC%E5%BD%95%E7%BB%84%E6%B5%8B%E5%BA%8F\%E5%AE%8C%E6%95%B4%E7%89%88%E6%95%B0%E6%8D%AE\GDR3855-Hylocereus_undulatus_Britt-12-RNAseq_result\4_Function\2_Group_Diff_Function\UP_DOWN\GO\NL-VS-L1.P.html#gene385) | plastid membrane organization | 6 (0.49%) | 15 (0.45%) | 0.492576 | 0.999992 |
| 386 | [GO:0010817](file:///E:\2018-7-3%E7%81%AB%E9%BE%99%E6%9E%9C%E8%BD%AC%E5%BD%95%E7%BB%84%E6%B5%8B%E5%BA%8F\%E5%AE%8C%E6%95%B4%E7%89%88%E6%95%B0%E6%8D%AE\GDR3855-Hylocereus_undulatus_Britt-12-RNAseq_result\4_Function\2_Group_Diff_Function\UP_DOWN\GO\NL-VS-L1.P.html#gene386) | regulation of hormone levels | 6 (0.49%) | 15 (0.45%) | 0.492576 | 0.999992 |
| 387 | [GO:0032535](file:///E:\2018-7-3%E7%81%AB%E9%BE%99%E6%9E%9C%E8%BD%AC%E5%BD%95%E7%BB%84%E6%B5%8B%E5%BA%8F\%E5%AE%8C%E6%95%B4%E7%89%88%E6%95%B0%E6%8D%AE\GDR3855-Hylocereus_undulatus_Britt-12-RNAseq_result\4_Function\2_Group_Diff_Function\UP_DOWN\GO\NL-VS-L1.P.html#gene387) | regulation of cellular component size | 6 (0.49%) | 15 (0.45%) | 0.492576 | 0.999992 |
| 388 | [GO:0048364](file:///E:\2018-7-3%E7%81%AB%E9%BE%99%E6%9E%9C%E8%BD%AC%E5%BD%95%E7%BB%84%E6%B5%8B%E5%BA%8F\%E5%AE%8C%E6%95%B4%E7%89%88%E6%95%B0%E6%8D%AE\GDR3855-Hylocereus_undulatus_Britt-12-RNAseq_result\4_Function\2_Group_Diff_Function\UP_DOWN\GO\NL-VS-L1.P.html#gene388) | root development | 6 (0.49%) | 15 (0.45%) | 0.492576 | 0.999992 |
| 389 | [GO:0090066](file:///E:\2018-7-3%E7%81%AB%E9%BE%99%E6%9E%9C%E8%BD%AC%E5%BD%95%E7%BB%84%E6%B5%8B%E5%BA%8F\%E5%AE%8C%E6%95%B4%E7%89%88%E6%95%B0%E6%8D%AE\GDR3855-Hylocereus_undulatus_Britt-12-RNAseq_result\4_Function\2_Group_Diff_Function\UP_DOWN\GO\NL-VS-L1.P.html#gene389) | regulation of anatomical structure size | 6 (0.49%) | 15 (0.45%) | 0.492576 | 0.999992 |
| 390 | [GO:0008610](file:///E:\2018-7-3%E7%81%AB%E9%BE%99%E6%9E%9C%E8%BD%AC%E5%BD%95%E7%BB%84%E6%B5%8B%E5%BA%8F\%E5%AE%8C%E6%95%B4%E7%89%88%E6%95%B0%E6%8D%AE\GDR3855-Hylocereus_undulatus_Britt-12-RNAseq_result\4_Function\2_Group_Diff_Function\UP_DOWN\GO\NL-VS-L1.P.html#gene390) | lipid biosynthetic process | 14 (1.13%) | 37 (1.1%) | 0.506668 | 0.999992 |
| 391 | [GO:0007015](file:///E:\2018-7-3%E7%81%AB%E9%BE%99%E6%9E%9C%E8%BD%AC%E5%BD%95%E7%BB%84%E6%B5%8B%E5%BA%8F\%E5%AE%8C%E6%95%B4%E7%89%88%E6%95%B0%E6%8D%AE\GDR3855-Hylocereus_undulatus_Britt-12-RNAseq_result\4_Function\2_Group_Diff_Function\UP_DOWN\GO\NL-VS-L1.P.html#gene391) | actin filament organization | 3 (0.24%) | 7 (0.21%) | 0.507468 | 0.999992 |
| 392 | [GO:0009626](file:///E:\2018-7-3%E7%81%AB%E9%BE%99%E6%9E%9C%E8%BD%AC%E5%BD%95%E7%BB%84%E6%B5%8B%E5%BA%8F\%E5%AE%8C%E6%95%B4%E7%89%88%E6%95%B0%E6%8D%AE\GDR3855-Hylocereus_undulatus_Britt-12-RNAseq_result\4_Function\2_Group_Diff_Function\UP_DOWN\GO\NL-VS-L1.P.html#gene392) | plant-type hypersensitive response | 3 (0.24%) | 7 (0.21%) | 0.507468 | 0.999992 |
| 393 | [GO:0009629](file:///E:\2018-7-3%E7%81%AB%E9%BE%99%E6%9E%9C%E8%BD%AC%E5%BD%95%E7%BB%84%E6%B5%8B%E5%BA%8F\%E5%AE%8C%E6%95%B4%E7%89%88%E6%95%B0%E6%8D%AE\GDR3855-Hylocereus_undulatus_Britt-12-RNAseq_result\4_Function\2_Group_Diff_Function\UP_DOWN\GO\NL-VS-L1.P.html#gene393) | response to gravity | 3 (0.24%) | 7 (0.21%) | 0.507468 | 0.999992 |
| 394 | [GO:0015893](file:///E:\2018-7-3%E7%81%AB%E9%BE%99%E6%9E%9C%E8%BD%AC%E5%BD%95%E7%BB%84%E6%B5%8B%E5%BA%8F\%E5%AE%8C%E6%95%B4%E7%89%88%E6%95%B0%E6%8D%AE\GDR3855-Hylocereus_undulatus_Britt-12-RNAseq_result\4_Function\2_Group_Diff_Function\UP_DOWN\GO\NL-VS-L1.P.html#gene394) | drug transport | 3 (0.24%) | 7 (0.21%) | 0.507468 | 0.999992 |
| 395 | [GO:0018193](file:///E:\2018-7-3%E7%81%AB%E9%BE%99%E6%9E%9C%E8%BD%AC%E5%BD%95%E7%BB%84%E6%B5%8B%E5%BA%8F\%E5%AE%8C%E6%95%B4%E7%89%88%E6%95%B0%E6%8D%AE\GDR3855-Hylocereus_undulatus_Britt-12-RNAseq_result\4_Function\2_Group_Diff_Function\UP_DOWN\GO\NL-VS-L1.P.html#gene395) | peptidyl-amino acid modification | 3 (0.24%) | 7 (0.21%) | 0.507468 | 0.999992 |
| 396 | [GO:0018205](file:///E:\2018-7-3%E7%81%AB%E9%BE%99%E6%9E%9C%E8%BD%AC%E5%BD%95%E7%BB%84%E6%B5%8B%E5%BA%8F\%E5%AE%8C%E6%95%B4%E7%89%88%E6%95%B0%E6%8D%AE\GDR3855-Hylocereus_undulatus_Britt-12-RNAseq_result\4_Function\2_Group_Diff_Function\UP_DOWN\GO\NL-VS-L1.P.html#gene396) | peptidyl-lysine modification | 3 (0.24%) | 7 (0.21%) | 0.507468 | 0.999992 |
| 397 | [GO:0032269](file:///E:\2018-7-3%E7%81%AB%E9%BE%99%E6%9E%9C%E8%BD%AC%E5%BD%95%E7%BB%84%E6%B5%8B%E5%BA%8F\%E5%AE%8C%E6%95%B4%E7%89%88%E6%95%B0%E6%8D%AE\GDR3855-Hylocereus_undulatus_Britt-12-RNAseq_result\4_Function\2_Group_Diff_Function\UP_DOWN\GO\NL-VS-L1.P.html#gene397) | negative regulation of cellular protein metabolic process | 3 (0.24%) | 7 (0.21%) | 0.507468 | 0.999992 |
| 398 | [GO:0034050](file:///E:\2018-7-3%E7%81%AB%E9%BE%99%E6%9E%9C%E8%BD%AC%E5%BD%95%E7%BB%84%E6%B5%8B%E5%BA%8F\%E5%AE%8C%E6%95%B4%E7%89%88%E6%95%B0%E6%8D%AE\GDR3855-Hylocereus_undulatus_Britt-12-RNAseq_result\4_Function\2_Group_Diff_Function\UP_DOWN\GO\NL-VS-L1.P.html#gene398) | host programmed cell death induced by symbiont | 3 (0.24%) | 7 (0.21%) | 0.507468 | 0.999992 |
| 399 | [GO:0042493](file:///E:\2018-7-3%E7%81%AB%E9%BE%99%E6%9E%9C%E8%BD%AC%E5%BD%95%E7%BB%84%E6%B5%8B%E5%BA%8F\%E5%AE%8C%E6%95%B4%E7%89%88%E6%95%B0%E6%8D%AE\GDR3855-Hylocereus_undulatus_Britt-12-RNAseq_result\4_Function\2_Group_Diff_Function\UP_DOWN\GO\NL-VS-L1.P.html#gene399) | response to drug | 3 (0.24%) | 7 (0.21%) | 0.507468 | 0.999992 |
| 400 | [GO:0043248](file:///E:\2018-7-3%E7%81%AB%E9%BE%99%E6%9E%9C%E8%BD%AC%E5%BD%95%E7%BB%84%E6%B5%8B%E5%BA%8F\%E5%AE%8C%E6%95%B4%E7%89%88%E6%95%B0%E6%8D%AE\GDR3855-Hylocereus_undulatus_Britt-12-RNAseq_result\4_Function\2_Group_Diff_Function\UP_DOWN\GO\NL-VS-L1.P.html#gene400) | proteasome assembly | 3 (0.24%) | 7 (0.21%) | 0.507468 | 0.999992 |
| 401 | [GO:0051248](file:///E:\2018-7-3%E7%81%AB%E9%BE%99%E6%9E%9C%E8%BD%AC%E5%BD%95%E7%BB%84%E6%B5%8B%E5%BA%8F\%E5%AE%8C%E6%95%B4%E7%89%88%E6%95%B0%E6%8D%AE\GDR3855-Hylocereus_undulatus_Britt-12-RNAseq_result\4_Function\2_Group_Diff_Function\UP_DOWN\GO\NL-VS-L1.P.html#gene401) | negative regulation of protein metabolic process | 3 (0.24%) | 7 (0.21%) | 0.507468 | 0.999992 |
| 402 | [GO:0051726](file:///E:\2018-7-3%E7%81%AB%E9%BE%99%E6%9E%9C%E8%BD%AC%E5%BD%95%E7%BB%84%E6%B5%8B%E5%BA%8F\%E5%AE%8C%E6%95%B4%E7%89%88%E6%95%B0%E6%8D%AE\GDR3855-Hylocereus_undulatus_Britt-12-RNAseq_result\4_Function\2_Group_Diff_Function\UP_DOWN\GO\NL-VS-L1.P.html#gene402) | regulation of cell cycle | 3 (0.24%) | 7 (0.21%) | 0.507468 | 0.999992 |
| 403 | [GO:0065009](file:///E:\2018-7-3%E7%81%AB%E9%BE%99%E6%9E%9C%E8%BD%AC%E5%BD%95%E7%BB%84%E6%B5%8B%E5%BA%8F\%E5%AE%8C%E6%95%B4%E7%89%88%E6%95%B0%E6%8D%AE\GDR3855-Hylocereus_undulatus_Britt-12-RNAseq_result\4_Function\2_Group_Diff_Function\UP_DOWN\GO\NL-VS-L1.P.html#gene403) | regulation of molecular function | 29 (2.35%) | 78 (2.32%) | 0.510656 | 0.999992 |
| 404 | [GO:0009620](file:///E:\2018-7-3%E7%81%AB%E9%BE%99%E6%9E%9C%E8%BD%AC%E5%BD%95%E7%BB%84%E6%B5%8B%E5%BA%8F\%E5%AE%8C%E6%95%B4%E7%89%88%E6%95%B0%E6%8D%AE\GDR3855-Hylocereus_undulatus_Britt-12-RNAseq_result\4_Function\2_Group_Diff_Function\UP_DOWN\GO\NL-VS-L1.P.html#gene404) | response to fungus | 7 (0.57%) | 18 (0.54%) | 0.513164 | 0.999992 |
| 405 | [GO:0006520](file:///E:\2018-7-3%E7%81%AB%E9%BE%99%E6%9E%9C%E8%BD%AC%E5%BD%95%E7%BB%84%E6%B5%8B%E5%BA%8F\%E5%AE%8C%E6%95%B4%E7%89%88%E6%95%B0%E6%8D%AE\GDR3855-Hylocereus_undulatus_Britt-12-RNAseq_result\4_Function\2_Group_Diff_Function\UP_DOWN\GO\NL-VS-L1.P.html#gene405) | cellular amino acid metabolic process | 37 (3%) | 100 (2.98%) | 0.516398 | 0.999992 |
| 406 | [GO:0019748](file:///E:\2018-7-3%E7%81%AB%E9%BE%99%E6%9E%9C%E8%BD%AC%E5%BD%95%E7%BB%84%E6%B5%8B%E5%BA%8F\%E5%AE%8C%E6%95%B4%E7%89%88%E6%95%B0%E6%8D%AE\GDR3855-Hylocereus_undulatus_Britt-12-RNAseq_result\4_Function\2_Group_Diff_Function\UP_DOWN\GO\NL-VS-L1.P.html#gene406) | secondary metabolic process | 11 (0.89%) | 29 (0.86%) | 0.516857 | 0.999992 |
| 407 | [GO:0001101](file:///E:\2018-7-3%E7%81%AB%E9%BE%99%E6%9E%9C%E8%BD%AC%E5%BD%95%E7%BB%84%E6%B5%8B%E5%BA%8F\%E5%AE%8C%E6%95%B4%E7%89%88%E6%95%B0%E6%8D%AE\GDR3855-Hylocereus_undulatus_Britt-12-RNAseq_result\4_Function\2_Group_Diff_Function\UP_DOWN\GO\NL-VS-L1.P.html#gene407) | response to acid chemical | 26 (2.11%) | 70 (2.08%) | 0.517304 | 0.999992 |
| 408 | [GO:0048507](file:///E:\2018-7-3%E7%81%AB%E9%BE%99%E6%9E%9C%E8%BD%AC%E5%BD%95%E7%BB%84%E6%B5%8B%E5%BA%8F\%E5%AE%8C%E6%95%B4%E7%89%88%E6%95%B0%E6%8D%AE\GDR3855-Hylocereus_undulatus_Britt-12-RNAseq_result\4_Function\2_Group_Diff_Function\UP_DOWN\GO\NL-VS-L1.P.html#gene408) | meristem development | 8 (0.65%) | 21 (0.63%) | 0.530688 | 0.999992 |
| 409 | [GO:0070887](file:///E:\2018-7-3%E7%81%AB%E9%BE%99%E6%9E%9C%E8%BD%AC%E5%BD%95%E7%BB%84%E6%B5%8B%E5%BA%8F\%E5%AE%8C%E6%95%B4%E7%89%88%E6%95%B0%E6%8D%AE\GDR3855-Hylocereus_undulatus_Britt-12-RNAseq_result\4_Function\2_Group_Diff_Function\UP_DOWN\GO\NL-VS-L1.P.html#gene409) | cellular response to chemical stimulus | 16 (1.3%) | 43 (1.28%) | 0.532118 | 0.999992 |
| 410 | [GO:0006996](file:///E:\2018-7-3%E7%81%AB%E9%BE%99%E6%9E%9C%E8%BD%AC%E5%BD%95%E7%BB%84%E6%B5%8B%E5%BA%8F\%E5%AE%8C%E6%95%B4%E7%89%88%E6%95%B0%E6%8D%AE\GDR3855-Hylocereus_undulatus_Britt-12-RNAseq_result\4_Function\2_Group_Diff_Function\UP_DOWN\GO\NL-VS-L1.P.html#gene410) | organelle organization | 54 (4.38%) | 147 (4.38%) | 0.532289 | 0.999992 |
| 411 | [GO:0009813](file:///E:\2018-7-3%E7%81%AB%E9%BE%99%E6%9E%9C%E8%BD%AC%E5%BD%95%E7%BB%84%E6%B5%8B%E5%BA%8F\%E5%AE%8C%E6%95%B4%E7%89%88%E6%95%B0%E6%8D%AE\GDR3855-Hylocereus_undulatus_Britt-12-RNAseq_result\4_Function\2_Group_Diff_Function\UP_DOWN\GO\NL-VS-L1.P.html#gene411) | flavonoid biosynthetic process | 4 (0.32%) | 10 (0.3%) | 0.533120 | 0.999992 |
| 412 | [GO:0065008](file:///E:\2018-7-3%E7%81%AB%E9%BE%99%E6%9E%9C%E8%BD%AC%E5%BD%95%E7%BB%84%E6%B5%8B%E5%BA%8F\%E5%AE%8C%E6%95%B4%E7%89%88%E6%95%B0%E6%8D%AE\GDR3855-Hylocereus_undulatus_Britt-12-RNAseq_result\4_Function\2_Group_Diff_Function\UP_DOWN\GO\NL-VS-L1.P.html#gene412) | regulation of biological quality | 36 (2.92%) | 98 (2.92%) | 0.539012 | 0.999992 |
| 413 | [GO:0006955](file:///E:\2018-7-3%E7%81%AB%E9%BE%99%E6%9E%9C%E8%BD%AC%E5%BD%95%E7%BB%84%E6%B5%8B%E5%BA%8F\%E5%AE%8C%E6%95%B4%E7%89%88%E6%95%B0%E6%8D%AE\GDR3855-Hylocereus_undulatus_Britt-12-RNAseq_result\4_Function\2_Group_Diff_Function\UP_DOWN\GO\NL-VS-L1.P.html#gene413) | immune response | 9 (0.73%) | 24 (0.71%) | 0.546007 | 0.999992 |
| 414 | [GO:0045087](file:///E:\2018-7-3%E7%81%AB%E9%BE%99%E6%9E%9C%E8%BD%AC%E5%BD%95%E7%BB%84%E6%B5%8B%E5%BA%8F\%E5%AE%8C%E6%95%B4%E7%89%88%E6%95%B0%E6%8D%AE\GDR3855-Hylocereus_undulatus_Britt-12-RNAseq_result\4_Function\2_Group_Diff_Function\UP_DOWN\GO\NL-VS-L1.P.html#gene414) | innate immune response | 9 (0.73%) | 24 (0.71%) | 0.546007 | 0.999992 |
| 415 | [GO:0048468](file:///E:\2018-7-3%E7%81%AB%E9%BE%99%E6%9E%9C%E8%BD%AC%E5%BD%95%E7%BB%84%E6%B5%8B%E5%BA%8F\%E5%AE%8C%E6%95%B4%E7%89%88%E6%95%B0%E6%8D%AE\GDR3855-Hylocereus_undulatus_Britt-12-RNAseq_result\4_Function\2_Group_Diff_Function\UP_DOWN\GO\NL-VS-L1.P.html#gene415) | cell development | 9 (0.73%) | 24 (0.71%) | 0.546007 | 0.999992 |
| 416 | [GO:0006637](file:///E:\2018-7-3%E7%81%AB%E9%BE%99%E6%9E%9C%E8%BD%AC%E5%BD%95%E7%BB%84%E6%B5%8B%E5%BA%8F\%E5%AE%8C%E6%95%B4%E7%89%88%E6%95%B0%E6%8D%AE\GDR3855-Hylocereus_undulatus_Britt-12-RNAseq_result\4_Function\2_Group_Diff_Function\UP_DOWN\GO\NL-VS-L1.P.html#gene416) | acyl-CoA metabolic process | 5 (0.41%) | 13 (0.39%) | 0.552590 | 0.999992 |
| 417 | [GO:0009814](file:///E:\2018-7-3%E7%81%AB%E9%BE%99%E6%9E%9C%E8%BD%AC%E5%BD%95%E7%BB%84%E6%B5%8B%E5%BA%8F\%E5%AE%8C%E6%95%B4%E7%89%88%E6%95%B0%E6%8D%AE\GDR3855-Hylocereus_undulatus_Britt-12-RNAseq_result\4_Function\2_Group_Diff_Function\UP_DOWN\GO\NL-VS-L1.P.html#gene417) | defense response, incompatible interaction | 5 (0.41%) | 13 (0.39%) | 0.552590 | 0.999992 |
| 418 | [GO:0012501](file:///E:\2018-7-3%E7%81%AB%E9%BE%99%E6%9E%9C%E8%BD%AC%E5%BD%95%E7%BB%84%E6%B5%8B%E5%BA%8F\%E5%AE%8C%E6%95%B4%E7%89%88%E6%95%B0%E6%8D%AE\GDR3855-Hylocereus_undulatus_Britt-12-RNAseq_result\4_Function\2_Group_Diff_Function\UP_DOWN\GO\NL-VS-L1.P.html#gene418) | programmed cell death | 5 (0.41%) | 13 (0.39%) | 0.552590 | 0.999992 |
| 419 | [GO:0035383](file:///E:\2018-7-3%E7%81%AB%E9%BE%99%E6%9E%9C%E8%BD%AC%E5%BD%95%E7%BB%84%E6%B5%8B%E5%BA%8F\%E5%AE%8C%E6%95%B4%E7%89%88%E6%95%B0%E6%8D%AE\GDR3855-Hylocereus_undulatus_Britt-12-RNAseq_result\4_Function\2_Group_Diff_Function\UP_DOWN\GO\NL-VS-L1.P.html#gene419) | thioester metabolic process | 5 (0.41%) | 13 (0.39%) | 0.552590 | 0.999992 |
| 420 | [GO:0046488](file:///E:\2018-7-3%E7%81%AB%E9%BE%99%E6%9E%9C%E8%BD%AC%E5%BD%95%E7%BB%84%E6%B5%8B%E5%BA%8F\%E5%AE%8C%E6%95%B4%E7%89%88%E6%95%B0%E6%8D%AE\GDR3855-Hylocereus_undulatus_Britt-12-RNAseq_result\4_Function\2_Group_Diff_Function\UP_DOWN\GO\NL-VS-L1.P.html#gene420) | phosphatidylinositol metabolic process | 5 (0.41%) | 13 (0.39%) | 0.552590 | 0.999992 |
| 421 | [GO:0055082](file:///E:\2018-7-3%E7%81%AB%E9%BE%99%E6%9E%9C%E8%BD%AC%E5%BD%95%E7%BB%84%E6%B5%8B%E5%BA%8F\%E5%AE%8C%E6%95%B4%E7%89%88%E6%95%B0%E6%8D%AE\GDR3855-Hylocereus_undulatus_Britt-12-RNAseq_result\4_Function\2_Group_Diff_Function\UP_DOWN\GO\NL-VS-L1.P.html#gene421) | cellular chemical homeostasis | 5 (0.41%) | 13 (0.39%) | 0.552590 | 0.999992 |
| 422 | [GO:1901605](file:///E:\2018-7-3%E7%81%AB%E9%BE%99%E6%9E%9C%E8%BD%AC%E5%BD%95%E7%BB%84%E6%B5%8B%E5%BA%8F\%E5%AE%8C%E6%95%B4%E7%89%88%E6%95%B0%E6%8D%AE\GDR3855-Hylocereus_undulatus_Britt-12-RNAseq_result\4_Function\2_Group_Diff_Function\UP_DOWN\GO\NL-VS-L1.P.html#gene422) | alpha-amino acid metabolic process | 22 (1.78%) | 60 (1.79%) | 0.553673 | 0.999992 |
| 423 | [GO:0042221](file:///E:\2018-7-3%E7%81%AB%E9%BE%99%E6%9E%9C%E8%BD%AC%E5%BD%95%E7%BB%84%E6%B5%8B%E5%BA%8F\%E5%AE%8C%E6%95%B4%E7%89%88%E6%95%B0%E6%8D%AE\GDR3855-Hylocereus_undulatus_Britt-12-RNAseq_result\4_Function\2_Group_Diff_Function\UP_DOWN\GO\NL-VS-L1.P.html#gene423) | response to chemical | 84 (6.81%) | 230 (6.85%) | 0.553970 | 0.999992 |
| 424 | [GO:0048518](file:///E:\2018-7-3%E7%81%AB%E9%BE%99%E6%9E%9C%E8%BD%AC%E5%BD%95%E7%BB%84%E6%B5%8B%E5%BA%8F\%E5%AE%8C%E6%95%B4%E7%89%88%E6%95%B0%E6%8D%AE\GDR3855-Hylocereus_undulatus_Britt-12-RNAseq_result\4_Function\2_Group_Diff_Function\UP_DOWN\GO\NL-VS-L1.P.html#gene424) | positive regulation of biological process | 18 (1.46%) | 49 (1.46%) | 0.554360 | 0.999992 |
| 425 | [GO:0016265](file:///E:\2018-7-3%E7%81%AB%E9%BE%99%E6%9E%9C%E8%BD%AC%E5%BD%95%E7%BB%84%E6%B5%8B%E5%BA%8F\%E5%AE%8C%E6%95%B4%E7%89%88%E6%95%B0%E6%8D%AE\GDR3855-Hylocereus_undulatus_Britt-12-RNAseq_result\4_Function\2_Group_Diff_Function\UP_DOWN\GO\NL-VS-L1.P.html#gene425) | death | 6 (0.49%) | 16 (0.48%) | 0.568560 | 0.999992 |
| 426 | [GO:0098542](file:///E:\2018-7-3%E7%81%AB%E9%BE%99%E6%9E%9C%E8%BD%AC%E5%BD%95%E7%BB%84%E6%B5%8B%E5%BA%8F\%E5%AE%8C%E6%95%B4%E7%89%88%E6%95%B0%E6%8D%AE\GDR3855-Hylocereus_undulatus_Britt-12-RNAseq_result\4_Function\2_Group_Diff_Function\UP_DOWN\GO\NL-VS-L1.P.html#gene426) | defense response to other organism | 7 (0.57%) | 19 (0.57%) | 0.582256 | 0.999992 |
| 427 | [GO:0009411](file:///E:\2018-7-3%E7%81%AB%E9%BE%99%E6%9E%9C%E8%BD%AC%E5%BD%95%E7%BB%84%E6%B5%8B%E5%BA%8F\%E5%AE%8C%E6%95%B4%E7%89%88%E6%95%B0%E6%8D%AE\GDR3855-Hylocereus_undulatus_Britt-12-RNAseq_result\4_Function\2_Group_Diff_Function\UP_DOWN\GO\NL-VS-L1.P.html#gene427) | response to UV | 8 (0.65%) | 22 (0.65%) | 0.594346 | 0.999992 |
| 428 | [GO:0009628](file:///E:\2018-7-3%E7%81%AB%E9%BE%99%E6%9E%9C%E8%BD%AC%E5%BD%95%E7%BB%84%E6%B5%8B%E5%BA%8F\%E5%AE%8C%E6%95%B4%E7%89%88%E6%95%B0%E6%8D%AE\GDR3855-Hylocereus_undulatus_Britt-12-RNAseq_result\4_Function\2_Group_Diff_Function\UP_DOWN\GO\NL-VS-L1.P.html#gene428) | response to abiotic stimulus | 65 (5.27%) | 180 (5.36%) | 0.599697 | 0.999992 |
| 429 | [GO:0000724](file:///E:\2018-7-3%E7%81%AB%E9%BE%99%E6%9E%9C%E8%BD%AC%E5%BD%95%E7%BB%84%E6%B5%8B%E5%BA%8F\%E5%AE%8C%E6%95%B4%E7%89%88%E6%95%B0%E6%8D%AE\GDR3855-Hylocereus_undulatus_Britt-12-RNAseq_result\4_Function\2_Group_Diff_Function\UP_DOWN\GO\NL-VS-L1.P.html#gene429) | double-strand break repair via homologous recombination | 1 (0.08%) | 2 (0.06%) | 0.599850 | 0.999992 |
| 430 | [GO:0001678](file:///E:\2018-7-3%E7%81%AB%E9%BE%99%E6%9E%9C%E8%BD%AC%E5%BD%95%E7%BB%84%E6%B5%8B%E5%BA%8F\%E5%AE%8C%E6%95%B4%E7%89%88%E6%95%B0%E6%8D%AE\GDR3855-Hylocereus_undulatus_Britt-12-RNAseq_result\4_Function\2_Group_Diff_Function\UP_DOWN\GO\NL-VS-L1.P.html#gene430) | cellular glucose homeostasis | 1 (0.08%) | 2 (0.06%) | 0.599850 | 0.999992 |
| 431 | [GO:0005985](file:///E:\2018-7-3%E7%81%AB%E9%BE%99%E6%9E%9C%E8%BD%AC%E5%BD%95%E7%BB%84%E6%B5%8B%E5%BA%8F\%E5%AE%8C%E6%95%B4%E7%89%88%E6%95%B0%E6%8D%AE\GDR3855-Hylocereus_undulatus_Britt-12-RNAseq_result\4_Function\2_Group_Diff_Function\UP_DOWN\GO\NL-VS-L1.P.html#gene431) | sucrose metabolic process | 1 (0.08%) | 2 (0.06%) | 0.599850 | 0.999992 |
| 432 | [GO:0006026](file:///E:\2018-7-3%E7%81%AB%E9%BE%99%E6%9E%9C%E8%BD%AC%E5%BD%95%E7%BB%84%E6%B5%8B%E5%BA%8F\%E5%AE%8C%E6%95%B4%E7%89%88%E6%95%B0%E6%8D%AE\GDR3855-Hylocereus_undulatus_Britt-12-RNAseq_result\4_Function\2_Group_Diff_Function\UP_DOWN\GO\NL-VS-L1.P.html#gene432) | aminoglycan catabolic process | 1 (0.08%) | 2 (0.06%) | 0.599850 | 0.999992 |
| 433 | [GO:0006144](file:///E:\2018-7-3%E7%81%AB%E9%BE%99%E6%9E%9C%E8%BD%AC%E5%BD%95%E7%BB%84%E6%B5%8B%E5%BA%8F\%E5%AE%8C%E6%95%B4%E7%89%88%E6%95%B0%E6%8D%AE\GDR3855-Hylocereus_undulatus_Britt-12-RNAseq_result\4_Function\2_Group_Diff_Function\UP_DOWN\GO\NL-VS-L1.P.html#gene433) | purine nucleobase metabolic process | 1 (0.08%) | 2 (0.06%) | 0.599850 | 0.999992 |
| 434 | [GO:0006261](file:///E:\2018-7-3%E7%81%AB%E9%BE%99%E6%9E%9C%E8%BD%AC%E5%BD%95%E7%BB%84%E6%B5%8B%E5%BA%8F\%E5%AE%8C%E6%95%B4%E7%89%88%E6%95%B0%E6%8D%AE\GDR3855-Hylocereus_undulatus_Britt-12-RNAseq_result\4_Function\2_Group_Diff_Function\UP_DOWN\GO\NL-VS-L1.P.html#gene434) | DNA-dependent DNA replication | 1 (0.08%) | 2 (0.06%) | 0.599850 | 0.999992 |
| 435 | [GO:0006323](file:///E:\2018-7-3%E7%81%AB%E9%BE%99%E6%9E%9C%E8%BD%AC%E5%BD%95%E7%BB%84%E6%B5%8B%E5%BA%8F\%E5%AE%8C%E6%95%B4%E7%89%88%E6%95%B0%E6%8D%AE\GDR3855-Hylocereus_undulatus_Britt-12-RNAseq_result\4_Function\2_Group_Diff_Function\UP_DOWN\GO\NL-VS-L1.P.html#gene435) | DNA packaging | 1 (0.08%) | 2 (0.06%) | 0.599850 | 0.999992 |
| 436 | [GO:0006333](file:///E:\2018-7-3%E7%81%AB%E9%BE%99%E6%9E%9C%E8%BD%AC%E5%BD%95%E7%BB%84%E6%B5%8B%E5%BA%8F\%E5%AE%8C%E6%95%B4%E7%89%88%E6%95%B0%E6%8D%AE\GDR3855-Hylocereus_undulatus_Britt-12-RNAseq_result\4_Function\2_Group_Diff_Function\UP_DOWN\GO\NL-VS-L1.P.html#gene436) | chromatin assembly or disassembly | 1 (0.08%) | 2 (0.06%) | 0.599850 | 0.999992 |
| 437 | [GO:0006402](file:///E:\2018-7-3%E7%81%AB%E9%BE%99%E6%9E%9C%E8%BD%AC%E5%BD%95%E7%BB%84%E6%B5%8B%E5%BA%8F\%E5%AE%8C%E6%95%B4%E7%89%88%E6%95%B0%E6%8D%AE\GDR3855-Hylocereus_undulatus_Britt-12-RNAseq_result\4_Function\2_Group_Diff_Function\UP_DOWN\GO\NL-VS-L1.P.html#gene437) | mRNA catabolic process | 1 (0.08%) | 2 (0.06%) | 0.599850 | 0.999992 |
| 438 | [GO:0006551](file:///E:\2018-7-3%E7%81%AB%E9%BE%99%E6%9E%9C%E8%BD%AC%E5%BD%95%E7%BB%84%E6%B5%8B%E5%BA%8F\%E5%AE%8C%E6%95%B4%E7%89%88%E6%95%B0%E6%8D%AE\GDR3855-Hylocereus_undulatus_Britt-12-RNAseq_result\4_Function\2_Group_Diff_Function\UP_DOWN\GO\NL-VS-L1.P.html#gene438) | leucine metabolic process | 1 (0.08%) | 2 (0.06%) | 0.599850 | 0.999992 |
| 439 | [GO:0006560](file:///E:\2018-7-3%E7%81%AB%E9%BE%99%E6%9E%9C%E8%BD%AC%E5%BD%95%E7%BB%84%E6%B5%8B%E5%BA%8F\%E5%AE%8C%E6%95%B4%E7%89%88%E6%95%B0%E6%8D%AE\GDR3855-Hylocereus_undulatus_Britt-12-RNAseq_result\4_Function\2_Group_Diff_Function\UP_DOWN\GO\NL-VS-L1.P.html#gene439) | proline metabolic process | 1 (0.08%) | 2 (0.06%) | 0.599850 | 0.999992 |
| 440 | [GO:0006897](file:///E:\2018-7-3%E7%81%AB%E9%BE%99%E6%9E%9C%E8%BD%AC%E5%BD%95%E7%BB%84%E6%B5%8B%E5%BA%8F\%E5%AE%8C%E6%95%B4%E7%89%88%E6%95%B0%E6%8D%AE\GDR3855-Hylocereus_undulatus_Britt-12-RNAseq_result\4_Function\2_Group_Diff_Function\UP_DOWN\GO\NL-VS-L1.P.html#gene440) | endocytosis | 1 (0.08%) | 2 (0.06%) | 0.599850 | 0.999992 |
| 441 | [GO:0006914](file:///E:\2018-7-3%E7%81%AB%E9%BE%99%E6%9E%9C%E8%BD%AC%E5%BD%95%E7%BB%84%E6%B5%8B%E5%BA%8F\%E5%AE%8C%E6%95%B4%E7%89%88%E6%95%B0%E6%8D%AE\GDR3855-Hylocereus_undulatus_Britt-12-RNAseq_result\4_Function\2_Group_Diff_Function\UP_DOWN\GO\NL-VS-L1.P.html#gene441) | autophagy | 1 (0.08%) | 2 (0.06%) | 0.599850 | 0.999992 |
| 442 | [GO:0007005](file:///E:\2018-7-3%E7%81%AB%E9%BE%99%E6%9E%9C%E8%BD%AC%E5%BD%95%E7%BB%84%E6%B5%8B%E5%BA%8F\%E5%AE%8C%E6%95%B4%E7%89%88%E6%95%B0%E6%8D%AE\GDR3855-Hylocereus_undulatus_Britt-12-RNAseq_result\4_Function\2_Group_Diff_Function\UP_DOWN\GO\NL-VS-L1.P.html#gene442) | mitochondrion organization | 1 (0.08%) | 2 (0.06%) | 0.599850 | 0.999992 |
| 443 | [GO:0007276](file:///E:\2018-7-3%E7%81%AB%E9%BE%99%E6%9E%9C%E8%BD%AC%E5%BD%95%E7%BB%84%E6%B5%8B%E5%BA%8F\%E5%AE%8C%E6%95%B4%E7%89%88%E6%95%B0%E6%8D%AE\GDR3855-Hylocereus_undulatus_Britt-12-RNAseq_result\4_Function\2_Group_Diff_Function\UP_DOWN\GO\NL-VS-L1.P.html#gene443) | gamete generation | 1 (0.08%) | 2 (0.06%) | 0.599850 | 0.999992 |
| 444 | [GO:0007346](file:///E:\2018-7-3%E7%81%AB%E9%BE%99%E6%9E%9C%E8%BD%AC%E5%BD%95%E7%BB%84%E6%B5%8B%E5%BA%8F\%E5%AE%8C%E6%95%B4%E7%89%88%E6%95%B0%E6%8D%AE\GDR3855-Hylocereus_undulatus_Britt-12-RNAseq_result\4_Function\2_Group_Diff_Function\UP_DOWN\GO\NL-VS-L1.P.html#gene444) | regulation of mitotic cell cycle | 1 (0.08%) | 2 (0.06%) | 0.599850 | 0.999992 |
| 445 | [GO:0008300](file:///E:\2018-7-3%E7%81%AB%E9%BE%99%E6%9E%9C%E8%BD%AC%E5%BD%95%E7%BB%84%E6%B5%8B%E5%BA%8F\%E5%AE%8C%E6%95%B4%E7%89%88%E6%95%B0%E6%8D%AE\GDR3855-Hylocereus_undulatus_Britt-12-RNAseq_result\4_Function\2_Group_Diff_Function\UP_DOWN\GO\NL-VS-L1.P.html#gene445) | isoprenoid catabolic process | 1 (0.08%) | 2 (0.06%) | 0.599850 | 0.999992 |
| 446 | [GO:0009292](file:///E:\2018-7-3%E7%81%AB%E9%BE%99%E6%9E%9C%E8%BD%AC%E5%BD%95%E7%BB%84%E6%B5%8B%E5%BA%8F\%E5%AE%8C%E6%95%B4%E7%89%88%E6%95%B0%E6%8D%AE\GDR3855-Hylocereus_undulatus_Britt-12-RNAseq_result\4_Function\2_Group_Diff_Function\UP_DOWN\GO\NL-VS-L1.P.html#gene446) | genetic transfer | 1 (0.08%) | 2 (0.06%) | 0.599850 | 0.999992 |
| 447 | [GO:0009625](file:///E:\2018-7-3%E7%81%AB%E9%BE%99%E6%9E%9C%E8%BD%AC%E5%BD%95%E7%BB%84%E6%B5%8B%E5%BA%8F\%E5%AE%8C%E6%95%B4%E7%89%88%E6%95%B0%E6%8D%AE\GDR3855-Hylocereus_undulatus_Britt-12-RNAseq_result\4_Function\2_Group_Diff_Function\UP_DOWN\GO\NL-VS-L1.P.html#gene447) | response to insect | 1 (0.08%) | 2 (0.06%) | 0.599850 | 0.999992 |
| 448 | [GO:0009646](file:///E:\2018-7-3%E7%81%AB%E9%BE%99%E6%9E%9C%E8%BD%AC%E5%BD%95%E7%BB%84%E6%B5%8B%E5%BA%8F\%E5%AE%8C%E6%95%B4%E7%89%88%E6%95%B0%E6%8D%AE\GDR3855-Hylocereus_undulatus_Britt-12-RNAseq_result\4_Function\2_Group_Diff_Function\UP_DOWN\GO\NL-VS-L1.P.html#gene448) | response to absence of light | 1 (0.08%) | 2 (0.06%) | 0.599850 | 0.999992 |
| 449 | [GO:0009692](file:///E:\2018-7-3%E7%81%AB%E9%BE%99%E6%9E%9C%E8%BD%AC%E5%BD%95%E7%BB%84%E6%B5%8B%E5%BA%8F\%E5%AE%8C%E6%95%B4%E7%89%88%E6%95%B0%E6%8D%AE\GDR3855-Hylocereus_undulatus_Britt-12-RNAseq_result\4_Function\2_Group_Diff_Function\UP_DOWN\GO\NL-VS-L1.P.html#gene449) | ethylene metabolic process | 1 (0.08%) | 2 (0.06%) | 0.599850 | 0.999992 |
| 450 | [GO:0009718](file:///E:\2018-7-3%E7%81%AB%E9%BE%99%E6%9E%9C%E8%BD%AC%E5%BD%95%E7%BB%84%E6%B5%8B%E5%BA%8F\%E5%AE%8C%E6%95%B4%E7%89%88%E6%95%B0%E6%8D%AE\GDR3855-Hylocereus_undulatus_Britt-12-RNAseq_result\4_Function\2_Group_Diff_Function\UP_DOWN\GO\NL-VS-L1.P.html#gene450) | anthocyanin-containing compound biosynthetic process | 1 (0.08%) | 2 (0.06%) | 0.599850 | 0.999992 |
| 451 | [GO:0009757](file:///E:\2018-7-3%E7%81%AB%E9%BE%99%E6%9E%9C%E8%BD%AC%E5%BD%95%E7%BB%84%E6%B5%8B%E5%BA%8F\%E5%AE%8C%E6%95%B4%E7%89%88%E6%95%B0%E6%8D%AE\GDR3855-Hylocereus_undulatus_Britt-12-RNAseq_result\4_Function\2_Group_Diff_Function\UP_DOWN\GO\NL-VS-L1.P.html#gene451) | hexose mediated signaling | 1 (0.08%) | 2 (0.06%) | 0.599850 | 0.999992 |
| 452 | [GO:0009887](file:///E:\2018-7-3%E7%81%AB%E9%BE%99%E6%9E%9C%E8%BD%AC%E5%BD%95%E7%BB%84%E6%B5%8B%E5%BA%8F\%E5%AE%8C%E6%95%B4%E7%89%88%E6%95%B0%E6%8D%AE\GDR3855-Hylocereus_undulatus_Britt-12-RNAseq_result\4_Function\2_Group_Diff_Function\UP_DOWN\GO\NL-VS-L1.P.html#gene452) | organ morphogenesis | 1 (0.08%) | 2 (0.06%) | 0.599850 | 0.999992 |
| 453 | [GO:0009902](file:///E:\2018-7-3%E7%81%AB%E9%BE%99%E6%9E%9C%E8%BD%AC%E5%BD%95%E7%BB%84%E6%B5%8B%E5%BA%8F\%E5%AE%8C%E6%95%B4%E7%89%88%E6%95%B0%E6%8D%AE\GDR3855-Hylocereus_undulatus_Britt-12-RNAseq_result\4_Function\2_Group_Diff_Function\UP_DOWN\GO\NL-VS-L1.P.html#gene453) | chloroplast relocation | 1 (0.08%) | 2 (0.06%) | 0.599850 | 0.999992 |
| 454 | [GO:0010182](file:///E:\2018-7-3%E7%81%AB%E9%BE%99%E6%9E%9C%E8%BD%AC%E5%BD%95%E7%BB%84%E6%B5%8B%E5%BA%8F\%E5%AE%8C%E6%95%B4%E7%89%88%E6%95%B0%E6%8D%AE\GDR3855-Hylocereus_undulatus_Britt-12-RNAseq_result\4_Function\2_Group_Diff_Function\UP_DOWN\GO\NL-VS-L1.P.html#gene454) | sugar mediated signaling pathway | 1 (0.08%) | 2 (0.06%) | 0.599850 | 0.999992 |
| 455 | [GO:0010225](file:///E:\2018-7-3%E7%81%AB%E9%BE%99%E6%9E%9C%E8%BD%AC%E5%BD%95%E7%BB%84%E6%B5%8B%E5%BA%8F\%E5%AE%8C%E6%95%B4%E7%89%88%E6%95%B0%E6%8D%AE\GDR3855-Hylocereus_undulatus_Britt-12-RNAseq_result\4_Function\2_Group_Diff_Function\UP_DOWN\GO\NL-VS-L1.P.html#gene455) | response to UV-C | 1 (0.08%) | 2 (0.06%) | 0.599850 | 0.999992 |
| 456 | [GO:0010564](file:///E:\2018-7-3%E7%81%AB%E9%BE%99%E6%9E%9C%E8%BD%AC%E5%BD%95%E7%BB%84%E6%B5%8B%E5%BA%8F\%E5%AE%8C%E6%95%B4%E7%89%88%E6%95%B0%E6%8D%AE\GDR3855-Hylocereus_undulatus_Britt-12-RNAseq_result\4_Function\2_Group_Diff_Function\UP_DOWN\GO\NL-VS-L1.P.html#gene456) | regulation of cell cycle process | 1 (0.08%) | 2 (0.06%) | 0.599850 | 0.999992 |
| 457 | [GO:0010565](file:///E:\2018-7-3%E7%81%AB%E9%BE%99%E6%9E%9C%E8%BD%AC%E5%BD%95%E7%BB%84%E6%B5%8B%E5%BA%8F\%E5%AE%8C%E6%95%B4%E7%89%88%E6%95%B0%E6%8D%AE\GDR3855-Hylocereus_undulatus_Britt-12-RNAseq_result\4_Function\2_Group_Diff_Function\UP_DOWN\GO\NL-VS-L1.P.html#gene457) | regulation of cellular ketone metabolic process | 1 (0.08%) | 2 (0.06%) | 0.599850 | 0.999992 |
| 458 | [GO:0015718](file:///E:\2018-7-3%E7%81%AB%E9%BE%99%E6%9E%9C%E8%BD%AC%E5%BD%95%E7%BB%84%E6%B5%8B%E5%BA%8F\%E5%AE%8C%E6%95%B4%E7%89%88%E6%95%B0%E6%8D%AE\GDR3855-Hylocereus_undulatus_Britt-12-RNAseq_result\4_Function\2_Group_Diff_Function\UP_DOWN\GO\NL-VS-L1.P.html#gene458) | monocarboxylic acid transport | 1 (0.08%) | 2 (0.06%) | 0.599850 | 0.999992 |
| 459 | [GO:0015851](file:///E:\2018-7-3%E7%81%AB%E9%BE%99%E6%9E%9C%E8%BD%AC%E5%BD%95%E7%BB%84%E6%B5%8B%E5%BA%8F\%E5%AE%8C%E6%95%B4%E7%89%88%E6%95%B0%E6%8D%AE\GDR3855-Hylocereus_undulatus_Britt-12-RNAseq_result\4_Function\2_Group_Diff_Function\UP_DOWN\GO\NL-VS-L1.P.html#gene459) | nucleobase transport | 1 (0.08%) | 2 (0.06%) | 0.599850 | 0.999992 |
| 460 | [GO:0015858](file:///E:\2018-7-3%E7%81%AB%E9%BE%99%E6%9E%9C%E8%BD%AC%E5%BD%95%E7%BB%84%E6%B5%8B%E5%BA%8F\%E5%AE%8C%E6%95%B4%E7%89%88%E6%95%B0%E6%8D%AE\GDR3855-Hylocereus_undulatus_Britt-12-RNAseq_result\4_Function\2_Group_Diff_Function\UP_DOWN\GO\NL-VS-L1.P.html#gene460) | nucleoside transport | 1 (0.08%) | 2 (0.06%) | 0.599850 | 0.999992 |
| 461 | [GO:0015865](file:///E:\2018-7-3%E7%81%AB%E9%BE%99%E6%9E%9C%E8%BD%AC%E5%BD%95%E7%BB%84%E6%B5%8B%E5%BA%8F\%E5%AE%8C%E6%95%B4%E7%89%88%E6%95%B0%E6%8D%AE\GDR3855-Hylocereus_undulatus_Britt-12-RNAseq_result\4_Function\2_Group_Diff_Function\UP_DOWN\GO\NL-VS-L1.P.html#gene461) | purine nucleotide transport | 1 (0.08%) | 2 (0.06%) | 0.599850 | 0.999992 |
| 462 | [GO:0015914](file:///E:\2018-7-3%E7%81%AB%E9%BE%99%E6%9E%9C%E8%BD%AC%E5%BD%95%E7%BB%84%E6%B5%8B%E5%BA%8F\%E5%AE%8C%E6%95%B4%E7%89%88%E6%95%B0%E6%8D%AE\GDR3855-Hylocereus_undulatus_Britt-12-RNAseq_result\4_Function\2_Group_Diff_Function\UP_DOWN\GO\NL-VS-L1.P.html#gene462) | phospholipid transport | 1 (0.08%) | 2 (0.06%) | 0.599850 | 0.999992 |
| 463 | [GO:0016110](file:///E:\2018-7-3%E7%81%AB%E9%BE%99%E6%9E%9C%E8%BD%AC%E5%BD%95%E7%BB%84%E6%B5%8B%E5%BA%8F\%E5%AE%8C%E6%95%B4%E7%89%88%E6%95%B0%E6%8D%AE\GDR3855-Hylocereus_undulatus_Britt-12-RNAseq_result\4_Function\2_Group_Diff_Function\UP_DOWN\GO\NL-VS-L1.P.html#gene463) | tetraterpenoid catabolic process | 1 (0.08%) | 2 (0.06%) | 0.599850 | 0.999992 |
| 464 | [GO:0016115](file:///E:\2018-7-3%E7%81%AB%E9%BE%99%E6%9E%9C%E8%BD%AC%E5%BD%95%E7%BB%84%E6%B5%8B%E5%BA%8F\%E5%AE%8C%E6%95%B4%E7%89%88%E6%95%B0%E6%8D%AE\GDR3855-Hylocereus_undulatus_Britt-12-RNAseq_result\4_Function\2_Group_Diff_Function\UP_DOWN\GO\NL-VS-L1.P.html#gene464) | terpenoid catabolic process | 1 (0.08%) | 2 (0.06%) | 0.599850 | 0.999992 |
| 465 | [GO:0016118](file:///E:\2018-7-3%E7%81%AB%E9%BE%99%E6%9E%9C%E8%BD%AC%E5%BD%95%E7%BB%84%E6%B5%8B%E5%BA%8F\%E5%AE%8C%E6%95%B4%E7%89%88%E6%95%B0%E6%8D%AE\GDR3855-Hylocereus_undulatus_Britt-12-RNAseq_result\4_Function\2_Group_Diff_Function\UP_DOWN\GO\NL-VS-L1.P.html#gene465) | carotenoid catabolic process | 1 (0.08%) | 2 (0.06%) | 0.599850 | 0.999992 |
| 466 | [GO:0017148](file:///E:\2018-7-3%E7%81%AB%E9%BE%99%E6%9E%9C%E8%BD%AC%E5%BD%95%E7%BB%84%E6%B5%8B%E5%BA%8F\%E5%AE%8C%E6%95%B4%E7%89%88%E6%95%B0%E6%8D%AE\GDR3855-Hylocereus_undulatus_Britt-12-RNAseq_result\4_Function\2_Group_Diff_Function\UP_DOWN\GO\NL-VS-L1.P.html#gene466) | negative regulation of translation | 1 (0.08%) | 2 (0.06%) | 0.599850 | 0.999992 |
| 467 | [GO:0019216](file:///E:\2018-7-3%E7%81%AB%E9%BE%99%E6%9E%9C%E8%BD%AC%E5%BD%95%E7%BB%84%E6%B5%8B%E5%BA%8F\%E5%AE%8C%E6%95%B4%E7%89%88%E6%95%B0%E6%8D%AE\GDR3855-Hylocereus_undulatus_Britt-12-RNAseq_result\4_Function\2_Group_Diff_Function\UP_DOWN\GO\NL-VS-L1.P.html#gene467) | regulation of lipid metabolic process | 1 (0.08%) | 2 (0.06%) | 0.599850 | 0.999992 |
| 468 | [GO:0019750](file:///E:\2018-7-3%E7%81%AB%E9%BE%99%E6%9E%9C%E8%BD%AC%E5%BD%95%E7%BB%84%E6%B5%8B%E5%BA%8F\%E5%AE%8C%E6%95%B4%E7%89%88%E6%95%B0%E6%8D%AE\GDR3855-Hylocereus_undulatus_Britt-12-RNAseq_result\4_Function\2_Group_Diff_Function\UP_DOWN\GO\NL-VS-L1.P.html#gene468) | chloroplast localization | 1 (0.08%) | 2 (0.06%) | 0.599850 | 0.999992 |
| 469 | [GO:0031497](file:///E:\2018-7-3%E7%81%AB%E9%BE%99%E6%9E%9C%E8%BD%AC%E5%BD%95%E7%BB%84%E6%B5%8B%E5%BA%8F\%E5%AE%8C%E6%95%B4%E7%89%88%E6%95%B0%E6%8D%AE\GDR3855-Hylocereus_undulatus_Britt-12-RNAseq_result\4_Function\2_Group_Diff_Function\UP_DOWN\GO\NL-VS-L1.P.html#gene469) | chromatin assembly | 1 (0.08%) | 2 (0.06%) | 0.599850 | 0.999992 |
| 470 | [GO:0033500](file:///E:\2018-7-3%E7%81%AB%E9%BE%99%E6%9E%9C%E8%BD%AC%E5%BD%95%E7%BB%84%E6%B5%8B%E5%BA%8F\%E5%AE%8C%E6%95%B4%E7%89%88%E6%95%B0%E6%8D%AE\GDR3855-Hylocereus_undulatus_Britt-12-RNAseq_result\4_Function\2_Group_Diff_Function\UP_DOWN\GO\NL-VS-L1.P.html#gene470) | carbohydrate homeostasis | 1 (0.08%) | 2 (0.06%) | 0.599850 | 0.999992 |
| 471 | [GO:0034249](file:///E:\2018-7-3%E7%81%AB%E9%BE%99%E6%9E%9C%E8%BD%AC%E5%BD%95%E7%BB%84%E6%B5%8B%E5%BA%8F\%E5%AE%8C%E6%95%B4%E7%89%88%E6%95%B0%E6%8D%AE\GDR3855-Hylocereus_undulatus_Britt-12-RNAseq_result\4_Function\2_Group_Diff_Function\UP_DOWN\GO\NL-VS-L1.P.html#gene471) | negative regulation of cellular amide metabolic process | 1 (0.08%) | 2 (0.06%) | 0.599850 | 0.999992 |
| 472 | [GO:0035195](file:///E:\2018-7-3%E7%81%AB%E9%BE%99%E6%9E%9C%E8%BD%AC%E5%BD%95%E7%BB%84%E6%B5%8B%E5%BA%8F\%E5%AE%8C%E6%95%B4%E7%89%88%E6%95%B0%E6%8D%AE\GDR3855-Hylocereus_undulatus_Britt-12-RNAseq_result\4_Function\2_Group_Diff_Function\UP_DOWN\GO\NL-VS-L1.P.html#gene472) | gene silencing by miRNA | 1 (0.08%) | 2 (0.06%) | 0.599850 | 0.999992 |
| 473 | [GO:0035821](file:///E:\2018-7-3%E7%81%AB%E9%BE%99%E6%9E%9C%E8%BD%AC%E5%BD%95%E7%BB%84%E6%B5%8B%E5%BA%8F\%E5%AE%8C%E6%95%B4%E7%89%88%E6%95%B0%E6%8D%AE\GDR3855-Hylocereus_undulatus_Britt-12-RNAseq_result\4_Function\2_Group_Diff_Function\UP_DOWN\GO\NL-VS-L1.P.html#gene473) | modification of morphology or physiology of other organism | 1 (0.08%) | 2 (0.06%) | 0.599850 | 0.999992 |
| 474 | [GO:0042126](file:///E:\2018-7-3%E7%81%AB%E9%BE%99%E6%9E%9C%E8%BD%AC%E5%BD%95%E7%BB%84%E6%B5%8B%E5%BA%8F\%E5%AE%8C%E6%95%B4%E7%89%88%E6%95%B0%E6%8D%AE\GDR3855-Hylocereus_undulatus_Britt-12-RNAseq_result\4_Function\2_Group_Diff_Function\UP_DOWN\GO\NL-VS-L1.P.html#gene474) | nitrate metabolic process | 1 (0.08%) | 2 (0.06%) | 0.599850 | 0.999992 |
| 475 | [GO:0042593](file:///E:\2018-7-3%E7%81%AB%E9%BE%99%E6%9E%9C%E8%BD%AC%E5%BD%95%E7%BB%84%E6%B5%8B%E5%BA%8F\%E5%AE%8C%E6%95%B4%E7%89%88%E6%95%B0%E6%8D%AE\GDR3855-Hylocereus_undulatus_Britt-12-RNAseq_result\4_Function\2_Group_Diff_Function\UP_DOWN\GO\NL-VS-L1.P.html#gene475) | glucose homeostasis | 1 (0.08%) | 2 (0.06%) | 0.599850 | 0.999992 |
| 476 | [GO:0043101](file:///E:\2018-7-3%E7%81%AB%E9%BE%99%E6%9E%9C%E8%BD%AC%E5%BD%95%E7%BB%84%E6%B5%8B%E5%BA%8F\%E5%AE%8C%E6%95%B4%E7%89%88%E6%95%B0%E6%8D%AE\GDR3855-Hylocereus_undulatus_Britt-12-RNAseq_result\4_Function\2_Group_Diff_Function\UP_DOWN\GO\NL-VS-L1.P.html#gene476) | purine-containing compound salvage | 1 (0.08%) | 2 (0.06%) | 0.599850 | 0.999992 |
| 477 | [GO:0043449](file:///E:\2018-7-3%E7%81%AB%E9%BE%99%E6%9E%9C%E8%BD%AC%E5%BD%95%E7%BB%84%E6%B5%8B%E5%BA%8F\%E5%AE%8C%E6%95%B4%E7%89%88%E6%95%B0%E6%8D%AE\GDR3855-Hylocereus_undulatus_Britt-12-RNAseq_result\4_Function\2_Group_Diff_Function\UP_DOWN\GO\NL-VS-L1.P.html#gene477) | cellular alkene metabolic process | 1 (0.08%) | 2 (0.06%) | 0.599850 | 0.999992 |
| 478 | [GO:0044003](file:///E:\2018-7-3%E7%81%AB%E9%BE%99%E6%9E%9C%E8%BD%AC%E5%BD%95%E7%BB%84%E6%B5%8B%E5%BA%8F\%E5%AE%8C%E6%95%B4%E7%89%88%E6%95%B0%E6%8D%AE\GDR3855-Hylocereus_undulatus_Britt-12-RNAseq_result\4_Function\2_Group_Diff_Function\UP_DOWN\GO\NL-VS-L1.P.html#gene478) | modification by symbiont of host morphology or physiology | 1 (0.08%) | 2 (0.06%) | 0.599850 | 0.999992 |
| 479 | [GO:0044786](file:///E:\2018-7-3%E7%81%AB%E9%BE%99%E6%9E%9C%E8%BD%AC%E5%BD%95%E7%BB%84%E6%B5%8B%E5%BA%8F\%E5%AE%8C%E6%95%B4%E7%89%88%E6%95%B0%E6%8D%AE\GDR3855-Hylocereus_undulatus_Britt-12-RNAseq_result\4_Function\2_Group_Diff_Function\UP_DOWN\GO\NL-VS-L1.P.html#gene479) | cell cycle DNA replication | 1 (0.08%) | 2 (0.06%) | 0.599850 | 0.999992 |
| 480 | [GO:0046283](file:///E:\2018-7-3%E7%81%AB%E9%BE%99%E6%9E%9C%E8%BD%AC%E5%BD%95%E7%BB%84%E6%B5%8B%E5%BA%8F\%E5%AE%8C%E6%95%B4%E7%89%88%E6%95%B0%E6%8D%AE\GDR3855-Hylocereus_undulatus_Britt-12-RNAseq_result\4_Function\2_Group_Diff_Function\UP_DOWN\GO\NL-VS-L1.P.html#gene480) | anthocyanin-containing compound metabolic process | 1 (0.08%) | 2 (0.06%) | 0.599850 | 0.999992 |
| 481 | [GO:0046341](file:///E:\2018-7-3%E7%81%AB%E9%BE%99%E6%9E%9C%E8%BD%AC%E5%BD%95%E7%BB%84%E6%B5%8B%E5%BA%8F\%E5%AE%8C%E6%95%B4%E7%89%88%E6%95%B0%E6%8D%AE\GDR3855-Hylocereus_undulatus_Britt-12-RNAseq_result\4_Function\2_Group_Diff_Function\UP_DOWN\GO\NL-VS-L1.P.html#gene481) | CDP-diacylglycerol metabolic process | 1 (0.08%) | 2 (0.06%) | 0.599850 | 0.999992 |
| 482 | [GO:0046890](file:///E:\2018-7-3%E7%81%AB%E9%BE%99%E6%9E%9C%E8%BD%AC%E5%BD%95%E7%BB%84%E6%B5%8B%E5%BA%8F\%E5%AE%8C%E6%95%B4%E7%89%88%E6%95%B0%E6%8D%AE\GDR3855-Hylocereus_undulatus_Britt-12-RNAseq_result\4_Function\2_Group_Diff_Function\UP_DOWN\GO\NL-VS-L1.P.html#gene482) | regulation of lipid biosynthetic process | 1 (0.08%) | 2 (0.06%) | 0.599850 | 0.999992 |
| 483 | [GO:0048017](file:///E:\2018-7-3%E7%81%AB%E9%BE%99%E6%9E%9C%E8%BD%AC%E5%BD%95%E7%BB%84%E6%B5%8B%E5%BA%8F\%E5%AE%8C%E6%95%B4%E7%89%88%E6%95%B0%E6%8D%AE\GDR3855-Hylocereus_undulatus_Britt-12-RNAseq_result\4_Function\2_Group_Diff_Function\UP_DOWN\GO\NL-VS-L1.P.html#gene483) | inositol lipid-mediated signaling | 1 (0.08%) | 2 (0.06%) | 0.599850 | 0.999992 |
| 484 | [GO:0051341](file:///E:\2018-7-3%E7%81%AB%E9%BE%99%E6%9E%9C%E8%BD%AC%E5%BD%95%E7%BB%84%E6%B5%8B%E5%BA%8F\%E5%AE%8C%E6%95%B4%E7%89%88%E6%95%B0%E6%8D%AE\GDR3855-Hylocereus_undulatus_Britt-12-RNAseq_result\4_Function\2_Group_Diff_Function\UP_DOWN\GO\NL-VS-L1.P.html#gene484) | regulation of oxidoreductase activity | 1 (0.08%) | 2 (0.06%) | 0.599850 | 0.999992 |
| 485 | [GO:0051353](file:///E:\2018-7-3%E7%81%AB%E9%BE%99%E6%9E%9C%E8%BD%AC%E5%BD%95%E7%BB%84%E6%B5%8B%E5%BA%8F\%E5%AE%8C%E6%95%B4%E7%89%88%E6%95%B0%E6%8D%AE\GDR3855-Hylocereus_undulatus_Britt-12-RNAseq_result\4_Function\2_Group_Diff_Function\UP_DOWN\GO\NL-VS-L1.P.html#gene485) | positive regulation of oxidoreductase activity | 1 (0.08%) | 2 (0.06%) | 0.599850 | 0.999992 |
| 486 | [GO:0051644](file:///E:\2018-7-3%E7%81%AB%E9%BE%99%E6%9E%9C%E8%BD%AC%E5%BD%95%E7%BB%84%E6%B5%8B%E5%BA%8F\%E5%AE%8C%E6%95%B4%E7%89%88%E6%95%B0%E6%8D%AE\GDR3855-Hylocereus_undulatus_Britt-12-RNAseq_result\4_Function\2_Group_Diff_Function\UP_DOWN\GO\NL-VS-L1.P.html#gene486) | plastid localization | 1 (0.08%) | 2 (0.06%) | 0.599850 | 0.999992 |
| 487 | [GO:0051656](file:///E:\2018-7-3%E7%81%AB%E9%BE%99%E6%9E%9C%E8%BD%AC%E5%BD%95%E7%BB%84%E6%B5%8B%E5%BA%8F\%E5%AE%8C%E6%95%B4%E7%89%88%E6%95%B0%E6%8D%AE\GDR3855-Hylocereus_undulatus_Britt-12-RNAseq_result\4_Function\2_Group_Diff_Function\UP_DOWN\GO\NL-VS-L1.P.html#gene487) | establishment of organelle localization | 1 (0.08%) | 2 (0.06%) | 0.599850 | 0.999992 |
| 488 | [GO:0051667](file:///E:\2018-7-3%E7%81%AB%E9%BE%99%E6%9E%9C%E8%BD%AC%E5%BD%95%E7%BB%84%E6%B5%8B%E5%BA%8F\%E5%AE%8C%E6%95%B4%E7%89%88%E6%95%B0%E6%8D%AE\GDR3855-Hylocereus_undulatus_Britt-12-RNAseq_result\4_Function\2_Group_Diff_Function\UP_DOWN\GO\NL-VS-L1.P.html#gene488) | establishment of plastid localization | 1 (0.08%) | 2 (0.06%) | 0.599850 | 0.999992 |
| 489 | [GO:0051701](file:///E:\2018-7-3%E7%81%AB%E9%BE%99%E6%9E%9C%E8%BD%AC%E5%BD%95%E7%BB%84%E6%B5%8B%E5%BA%8F\%E5%AE%8C%E6%95%B4%E7%89%88%E6%95%B0%E6%8D%AE\GDR3855-Hylocereus_undulatus_Britt-12-RNAseq_result\4_Function\2_Group_Diff_Function\UP_DOWN\GO\NL-VS-L1.P.html#gene489) | interaction with host | 1 (0.08%) | 2 (0.06%) | 0.599850 | 0.999992 |
| 490 | [GO:0051817](file:///E:\2018-7-3%E7%81%AB%E9%BE%99%E6%9E%9C%E8%BD%AC%E5%BD%95%E7%BB%84%E6%B5%8B%E5%BA%8F\%E5%AE%8C%E6%95%B4%E7%89%88%E6%95%B0%E6%8D%AE\GDR3855-Hylocereus_undulatus_Britt-12-RNAseq_result\4_Function\2_Group_Diff_Function\UP_DOWN\GO\NL-VS-L1.P.html#gene490) | modification of morphology or physiology of other organism involved in symbiotic interaction | 1 (0.08%) | 2 (0.06%) | 0.599850 | 0.999992 |
| 491 | [GO:0052031](file:///E:\2018-7-3%E7%81%AB%E9%BE%99%E6%9E%9C%E8%BD%AC%E5%BD%95%E7%BB%84%E6%B5%8B%E5%BA%8F\%E5%AE%8C%E6%95%B4%E7%89%88%E6%95%B0%E6%8D%AE\GDR3855-Hylocereus_undulatus_Britt-12-RNAseq_result\4_Function\2_Group_Diff_Function\UP_DOWN\GO\NL-VS-L1.P.html#gene491) | modulation by symbiont of host defense response | 1 (0.08%) | 2 (0.06%) | 0.599850 | 0.999992 |
| 492 | [GO:0052166](file:///E:\2018-7-3%E7%81%AB%E9%BE%99%E6%9E%9C%E8%BD%AC%E5%BD%95%E7%BB%84%E6%B5%8B%E5%BA%8F\%E5%AE%8C%E6%95%B4%E7%89%88%E6%95%B0%E6%8D%AE\GDR3855-Hylocereus_undulatus_Britt-12-RNAseq_result\4_Function\2_Group_Diff_Function\UP_DOWN\GO\NL-VS-L1.P.html#gene492) | positive regulation by symbiont of host innate immune response | 1 (0.08%) | 2 (0.06%) | 0.599850 | 0.999992 |
| 493 | [GO:0052167](file:///E:\2018-7-3%E7%81%AB%E9%BE%99%E6%9E%9C%E8%BD%AC%E5%BD%95%E7%BB%84%E6%B5%8B%E5%BA%8F\%E5%AE%8C%E6%95%B4%E7%89%88%E6%95%B0%E6%8D%AE\GDR3855-Hylocereus_undulatus_Britt-12-RNAseq_result\4_Function\2_Group_Diff_Function\UP_DOWN\GO\NL-VS-L1.P.html#gene493) | modulation by symbiont of host innate immune response | 1 (0.08%) | 2 (0.06%) | 0.599850 | 0.999992 |
| 494 | [GO:0052173](file:///E:\2018-7-3%E7%81%AB%E9%BE%99%E6%9E%9C%E8%BD%AC%E5%BD%95%E7%BB%84%E6%B5%8B%E5%BA%8F\%E5%AE%8C%E6%95%B4%E7%89%88%E6%95%B0%E6%8D%AE\GDR3855-Hylocereus_undulatus_Britt-12-RNAseq_result\4_Function\2_Group_Diff_Function\UP_DOWN\GO\NL-VS-L1.P.html#gene494) | response to defenses of other organism involved in symbiotic interaction | 1 (0.08%) | 2 (0.06%) | 0.599850 | 0.999992 |
| 495 | [GO:0052200](file:///E:\2018-7-3%E7%81%AB%E9%BE%99%E6%9E%9C%E8%BD%AC%E5%BD%95%E7%BB%84%E6%B5%8B%E5%BA%8F\%E5%AE%8C%E6%95%B4%E7%89%88%E6%95%B0%E6%8D%AE\GDR3855-Hylocereus_undulatus_Britt-12-RNAseq_result\4_Function\2_Group_Diff_Function\UP_DOWN\GO\NL-VS-L1.P.html#gene495) | response to host defenses | 1 (0.08%) | 2 (0.06%) | 0.599850 | 0.999992 |
| 496 | [GO:0052255](file:///E:\2018-7-3%E7%81%AB%E9%BE%99%E6%9E%9C%E8%BD%AC%E5%BD%95%E7%BB%84%E6%B5%8B%E5%BA%8F\%E5%AE%8C%E6%95%B4%E7%89%88%E6%95%B0%E6%8D%AE\GDR3855-Hylocereus_undulatus_Britt-12-RNAseq_result\4_Function\2_Group_Diff_Function\UP_DOWN\GO\NL-VS-L1.P.html#gene496) | modulation by organism of defense response of other organism involved in symbiotic interaction | 1 (0.08%) | 2 (0.06%) | 0.599850 | 0.999992 |
| 497 | [GO:0052305](file:///E:\2018-7-3%E7%81%AB%E9%BE%99%E6%9E%9C%E8%BD%AC%E5%BD%95%E7%BB%84%E6%B5%8B%E5%BA%8F\%E5%AE%8C%E6%95%B4%E7%89%88%E6%95%B0%E6%8D%AE\GDR3855-Hylocereus_undulatus_Britt-12-RNAseq_result\4_Function\2_Group_Diff_Function\UP_DOWN\GO\NL-VS-L1.P.html#gene497) | positive regulation by organism of innate immune response in other organism involved in symbiotic interaction | 1 (0.08%) | 2 (0.06%) | 0.599850 | 0.999992 |
| 498 | [GO:0052306](file:///E:\2018-7-3%E7%81%AB%E9%BE%99%E6%9E%9C%E8%BD%AC%E5%BD%95%E7%BB%84%E6%B5%8B%E5%BA%8F\%E5%AE%8C%E6%95%B4%E7%89%88%E6%95%B0%E6%8D%AE\GDR3855-Hylocereus_undulatus_Britt-12-RNAseq_result\4_Function\2_Group_Diff_Function\UP_DOWN\GO\NL-VS-L1.P.html#gene498) | modulation by organism of innate immune response in other organism involved in symbiotic interaction | 1 (0.08%) | 2 (0.06%) | 0.599850 | 0.999992 |
| 499 | [GO:0052509](file:///E:\2018-7-3%E7%81%AB%E9%BE%99%E6%9E%9C%E8%BD%AC%E5%BD%95%E7%BB%84%E6%B5%8B%E5%BA%8F\%E5%AE%8C%E6%95%B4%E7%89%88%E6%95%B0%E6%8D%AE\GDR3855-Hylocereus_undulatus_Britt-12-RNAseq_result\4_Function\2_Group_Diff_Function\UP_DOWN\GO\NL-VS-L1.P.html#gene499) | positive regulation by symbiont of host defense response | 1 (0.08%) | 2 (0.06%) | 0.599850 | 0.999992 |
| 500 | [GO:0052510](file:///E:\2018-7-3%E7%81%AB%E9%BE%99%E6%9E%9C%E8%BD%AC%E5%BD%95%E7%BB%84%E6%B5%8B%E5%BA%8F\%E5%AE%8C%E6%95%B4%E7%89%88%E6%95%B0%E6%8D%AE\GDR3855-Hylocereus_undulatus_Britt-12-RNAseq_result\4_Function\2_Group_Diff_Function\UP_DOWN\GO\NL-VS-L1.P.html#gene500) | positive regulation by organism of defense response of other organism involved in symbiotic interaction | 1 (0.08%) | 2 (0.06%) | 0.599850 | 0.999992 |
| 501 | [GO:0052552](file:///E:\2018-7-3%E7%81%AB%E9%BE%99%E6%9E%9C%E8%BD%AC%E5%BD%95%E7%BB%84%E6%B5%8B%E5%BA%8F\%E5%AE%8C%E6%95%B4%E7%89%88%E6%95%B0%E6%8D%AE\GDR3855-Hylocereus_undulatus_Britt-12-RNAseq_result\4_Function\2_Group_Diff_Function\UP_DOWN\GO\NL-VS-L1.P.html#gene501) | modulation by organism of immune response of other organism involved in symbiotic interaction | 1 (0.08%) | 2 (0.06%) | 0.599850 | 0.999992 |
| 502 | [GO:0052553](file:///E:\2018-7-3%E7%81%AB%E9%BE%99%E6%9E%9C%E8%BD%AC%E5%BD%95%E7%BB%84%E6%B5%8B%E5%BA%8F\%E5%AE%8C%E6%95%B4%E7%89%88%E6%95%B0%E6%8D%AE\GDR3855-Hylocereus_undulatus_Britt-12-RNAseq_result\4_Function\2_Group_Diff_Function\UP_DOWN\GO\NL-VS-L1.P.html#gene502) | modulation by symbiont of host immune response | 1 (0.08%) | 2 (0.06%) | 0.599850 | 0.999992 |
| 503 | [GO:0052555](file:///E:\2018-7-3%E7%81%AB%E9%BE%99%E6%9E%9C%E8%BD%AC%E5%BD%95%E7%BB%84%E6%B5%8B%E5%BA%8F\%E5%AE%8C%E6%95%B4%E7%89%88%E6%95%B0%E6%8D%AE\GDR3855-Hylocereus_undulatus_Britt-12-RNAseq_result\4_Function\2_Group_Diff_Function\UP_DOWN\GO\NL-VS-L1.P.html#gene503) | positive regulation by organism of immune response of other organism involved in symbiotic interaction | 1 (0.08%) | 2 (0.06%) | 0.599850 | 0.999992 |
| 504 | [GO:0052556](file:///E:\2018-7-3%E7%81%AB%E9%BE%99%E6%9E%9C%E8%BD%AC%E5%BD%95%E7%BB%84%E6%B5%8B%E5%BA%8F\%E5%AE%8C%E6%95%B4%E7%89%88%E6%95%B0%E6%8D%AE\GDR3855-Hylocereus_undulatus_Britt-12-RNAseq_result\4_Function\2_Group_Diff_Function\UP_DOWN\GO\NL-VS-L1.P.html#gene504) | positive regulation by symbiont of host immune response | 1 (0.08%) | 2 (0.06%) | 0.599850 | 0.999992 |
| 505 | [GO:0052564](file:///E:\2018-7-3%E7%81%AB%E9%BE%99%E6%9E%9C%E8%BD%AC%E5%BD%95%E7%BB%84%E6%B5%8B%E5%BA%8F\%E5%AE%8C%E6%95%B4%E7%89%88%E6%95%B0%E6%8D%AE\GDR3855-Hylocereus_undulatus_Britt-12-RNAseq_result\4_Function\2_Group_Diff_Function\UP_DOWN\GO\NL-VS-L1.P.html#gene505) | response to immune response of other organism involved in symbiotic interaction | 1 (0.08%) | 2 (0.06%) | 0.599850 | 0.999992 |
| 506 | [GO:0052572](file:///E:\2018-7-3%E7%81%AB%E9%BE%99%E6%9E%9C%E8%BD%AC%E5%BD%95%E7%BB%84%E6%B5%8B%E5%BA%8F\%E5%AE%8C%E6%95%B4%E7%89%88%E6%95%B0%E6%8D%AE\GDR3855-Hylocereus_undulatus_Britt-12-RNAseq_result\4_Function\2_Group_Diff_Function\UP_DOWN\GO\NL-VS-L1.P.html#gene506) | response to host immune response | 1 (0.08%) | 2 (0.06%) | 0.599850 | 0.999992 |
| 507 | [GO:0071265](file:///E:\2018-7-3%E7%81%AB%E9%BE%99%E6%9E%9C%E8%BD%AC%E5%BD%95%E7%BB%84%E6%B5%8B%E5%BA%8F\%E5%AE%8C%E6%95%B4%E7%89%88%E6%95%B0%E6%8D%AE\GDR3855-Hylocereus_undulatus_Britt-12-RNAseq_result\4_Function\2_Group_Diff_Function\UP_DOWN\GO\NL-VS-L1.P.html#gene507) | L-methionine biosynthetic process | 1 (0.08%) | 2 (0.06%) | 0.599850 | 0.999992 |
| 508 | [GO:0071326](file:///E:\2018-7-3%E7%81%AB%E9%BE%99%E6%9E%9C%E8%BD%AC%E5%BD%95%E7%BB%84%E6%B5%8B%E5%BA%8F\%E5%AE%8C%E6%95%B4%E7%89%88%E6%95%B0%E6%8D%AE\GDR3855-Hylocereus_undulatus_Britt-12-RNAseq_result\4_Function\2_Group_Diff_Function\UP_DOWN\GO\NL-VS-L1.P.html#gene508) | cellular response to monosaccharide stimulus | 1 (0.08%) | 2 (0.06%) | 0.599850 | 0.999992 |
| 509 | [GO:0071331](file:///E:\2018-7-3%E7%81%AB%E9%BE%99%E6%9E%9C%E8%BD%AC%E5%BD%95%E7%BB%84%E6%B5%8B%E5%BA%8F\%E5%AE%8C%E6%95%B4%E7%89%88%E6%95%B0%E6%8D%AE\GDR3855-Hylocereus_undulatus_Britt-12-RNAseq_result\4_Function\2_Group_Diff_Function\UP_DOWN\GO\NL-VS-L1.P.html#gene509) | cellular response to hexose stimulus | 1 (0.08%) | 2 (0.06%) | 0.599850 | 0.999992 |
| 510 | [GO:0075136](file:///E:\2018-7-3%E7%81%AB%E9%BE%99%E6%9E%9C%E8%BD%AC%E5%BD%95%E7%BB%84%E6%B5%8B%E5%BA%8F\%E5%AE%8C%E6%95%B4%E7%89%88%E6%95%B0%E6%8D%AE\GDR3855-Hylocereus_undulatus_Britt-12-RNAseq_result\4_Function\2_Group_Diff_Function\UP_DOWN\GO\NL-VS-L1.P.html#gene510) | response to host | 1 (0.08%) | 2 (0.06%) | 0.599850 | 0.999992 |
| 511 | [GO:1900673](file:///E:\2018-7-3%E7%81%AB%E9%BE%99%E6%9E%9C%E8%BD%AC%E5%BD%95%E7%BB%84%E6%B5%8B%E5%BA%8F\%E5%AE%8C%E6%95%B4%E7%89%88%E6%95%B0%E6%8D%AE\GDR3855-Hylocereus_undulatus_Britt-12-RNAseq_result\4_Function\2_Group_Diff_Function\UP_DOWN\GO\NL-VS-L1.P.html#gene511) | olefin metabolic process | 1 (0.08%) | 2 (0.06%) | 0.599850 | 0.999992 |
| 512 | [GO:1901264](file:///E:\2018-7-3%E7%81%AB%E9%BE%99%E6%9E%9C%E8%BD%AC%E5%BD%95%E7%BB%84%E6%B5%8B%E5%BA%8F\%E5%AE%8C%E6%95%B4%E7%89%88%E6%95%B0%E6%8D%AE\GDR3855-Hylocereus_undulatus_Britt-12-RNAseq_result\4_Function\2_Group_Diff_Function\UP_DOWN\GO\NL-VS-L1.P.html#gene512) | carbohydrate derivative transport | 1 (0.08%) | 2 (0.06%) | 0.599850 | 0.999992 |
| 513 | [GO:2001057](file:///E:\2018-7-3%E7%81%AB%E9%BE%99%E6%9E%9C%E8%BD%AC%E5%BD%95%E7%BB%84%E6%B5%8B%E5%BA%8F\%E5%AE%8C%E6%95%B4%E7%89%88%E6%95%B0%E6%8D%AE\GDR3855-Hylocereus_undulatus_Britt-12-RNAseq_result\4_Function\2_Group_Diff_Function\UP_DOWN\GO\NL-VS-L1.P.html#gene513) | reactive nitrogen species metabolic process | 1 (0.08%) | 2 (0.06%) | 0.599850 | 0.999992 |
| 514 | [GO:0048522](file:///E:\2018-7-3%E7%81%AB%E9%BE%99%E6%9E%9C%E8%BD%AC%E5%BD%95%E7%BB%84%E6%B5%8B%E5%BA%8F\%E5%AE%8C%E6%95%B4%E7%89%88%E6%95%B0%E6%8D%AE\GDR3855-Hylocereus_undulatus_Britt-12-RNAseq_result\4_Function\2_Group_Diff_Function\UP_DOWN\GO\NL-VS-L1.P.html#gene514) | positive regulation of cellular process | 14 (1.13%) | 39 (1.16%) | 0.603470 | 0.999992 |
| 515 | [GO:0000398](file:///E:\2018-7-3%E7%81%AB%E9%BE%99%E6%9E%9C%E8%BD%AC%E5%BD%95%E7%BB%84%E6%B5%8B%E5%BA%8F\%E5%AE%8C%E6%95%B4%E7%89%88%E6%95%B0%E6%8D%AE\GDR3855-Hylocereus_undulatus_Britt-12-RNAseq_result\4_Function\2_Group_Diff_Function\UP_DOWN\GO\NL-VS-L1.P.html#gene515) | mRNA splicing, via spliceosome | 2 (0.16%) | 5 (0.15%) | 0.604579 | 0.999992 |
| 516 | [GO:0000910](file:///E:\2018-7-3%E7%81%AB%E9%BE%99%E6%9E%9C%E8%BD%AC%E5%BD%95%E7%BB%84%E6%B5%8B%E5%BA%8F\%E5%AE%8C%E6%95%B4%E7%89%88%E6%95%B0%E6%8D%AE\GDR3855-Hylocereus_undulatus_Britt-12-RNAseq_result\4_Function\2_Group_Diff_Function\UP_DOWN\GO\NL-VS-L1.P.html#gene516) | cytokinesis | 2 (0.16%) | 5 (0.15%) | 0.604579 | 0.999992 |
| 517 | [GO:0006020](file:///E:\2018-7-3%E7%81%AB%E9%BE%99%E6%9E%9C%E8%BD%AC%E5%BD%95%E7%BB%84%E6%B5%8B%E5%BA%8F\%E5%AE%8C%E6%95%B4%E7%89%88%E6%95%B0%E6%8D%AE\GDR3855-Hylocereus_undulatus_Britt-12-RNAseq_result\4_Function\2_Group_Diff_Function\UP_DOWN\GO\NL-VS-L1.P.html#gene517) | inositol metabolic process | 2 (0.16%) | 5 (0.15%) | 0.604579 | 0.999992 |
| 518 | [GO:0006814](file:///E:\2018-7-3%E7%81%AB%E9%BE%99%E6%9E%9C%E8%BD%AC%E5%BD%95%E7%BB%84%E6%B5%8B%E5%BA%8F\%E5%AE%8C%E6%95%B4%E7%89%88%E6%95%B0%E6%8D%AE\GDR3855-Hylocereus_undulatus_Britt-12-RNAseq_result\4_Function\2_Group_Diff_Function\UP_DOWN\GO\NL-VS-L1.P.html#gene518) | sodium ion transport | 2 (0.16%) | 5 (0.15%) | 0.604579 | 0.999992 |
| 519 | [GO:0009756](file:///E:\2018-7-3%E7%81%AB%E9%BE%99%E6%9E%9C%E8%BD%AC%E5%BD%95%E7%BB%84%E6%B5%8B%E5%BA%8F\%E5%AE%8C%E6%95%B4%E7%89%88%E6%95%B0%E6%8D%AE\GDR3855-Hylocereus_undulatus_Britt-12-RNAseq_result\4_Function\2_Group_Diff_Function\UP_DOWN\GO\NL-VS-L1.P.html#gene519) | carbohydrate mediated signaling | 2 (0.16%) | 5 (0.15%) | 0.604579 | 0.999992 |
| 520 | [GO:0009832](file:///E:\2018-7-3%E7%81%AB%E9%BE%99%E6%9E%9C%E8%BD%AC%E5%BD%95%E7%BB%84%E6%B5%8B%E5%BA%8F\%E5%AE%8C%E6%95%B4%E7%89%88%E6%95%B0%E6%8D%AE\GDR3855-Hylocereus_undulatus_Britt-12-RNAseq_result\4_Function\2_Group_Diff_Function\UP_DOWN\GO\NL-VS-L1.P.html#gene520) | plant-type cell wall biogenesis | 2 (0.16%) | 5 (0.15%) | 0.604579 | 0.999992 |
| 521 | [GO:0009845](file:///E:\2018-7-3%E7%81%AB%E9%BE%99%E6%9E%9C%E8%BD%AC%E5%BD%95%E7%BB%84%E6%B5%8B%E5%BA%8F\%E5%AE%8C%E6%95%B4%E7%89%88%E6%95%B0%E6%8D%AE\GDR3855-Hylocereus_undulatus_Britt-12-RNAseq_result\4_Function\2_Group_Diff_Function\UP_DOWN\GO\NL-VS-L1.P.html#gene521) | seed germination | 2 (0.16%) | 5 (0.15%) | 0.604579 | 0.999992 |
| 522 | [GO:0010466](file:///E:\2018-7-3%E7%81%AB%E9%BE%99%E6%9E%9C%E8%BD%AC%E5%BD%95%E7%BB%84%E6%B5%8B%E5%BA%8F\%E5%AE%8C%E6%95%B4%E7%89%88%E6%95%B0%E6%8D%AE\GDR3855-Hylocereus_undulatus_Britt-12-RNAseq_result\4_Function\2_Group_Diff_Function\UP_DOWN\GO\NL-VS-L1.P.html#gene522) | negative regulation of peptidase activity | 2 (0.16%) | 5 (0.15%) | 0.604579 | 0.999992 |
| 523 | [GO:0030162](file:///E:\2018-7-3%E7%81%AB%E9%BE%99%E6%9E%9C%E8%BD%AC%E5%BD%95%E7%BB%84%E6%B5%8B%E5%BA%8F\%E5%AE%8C%E6%95%B4%E7%89%88%E6%95%B0%E6%8D%AE\GDR3855-Hylocereus_undulatus_Britt-12-RNAseq_result\4_Function\2_Group_Diff_Function\UP_DOWN\GO\NL-VS-L1.P.html#gene523) | regulation of proteolysis | 2 (0.16%) | 5 (0.15%) | 0.604579 | 0.999992 |
| 524 | [GO:0043086](file:///E:\2018-7-3%E7%81%AB%E9%BE%99%E6%9E%9C%E8%BD%AC%E5%BD%95%E7%BB%84%E6%B5%8B%E5%BA%8F\%E5%AE%8C%E6%95%B4%E7%89%88%E6%95%B0%E6%8D%AE\GDR3855-Hylocereus_undulatus_Britt-12-RNAseq_result\4_Function\2_Group_Diff_Function\UP_DOWN\GO\NL-VS-L1.P.html#gene524) | negative regulation of catalytic activity | 2 (0.16%) | 5 (0.15%) | 0.604579 | 0.999992 |
| 525 | [GO:0045017](file:///E:\2018-7-3%E7%81%AB%E9%BE%99%E6%9E%9C%E8%BD%AC%E5%BD%95%E7%BB%84%E6%B5%8B%E5%BA%8F\%E5%AE%8C%E6%95%B4%E7%89%88%E6%95%B0%E6%8D%AE\GDR3855-Hylocereus_undulatus_Britt-12-RNAseq_result\4_Function\2_Group_Diff_Function\UP_DOWN\GO\NL-VS-L1.P.html#gene525) | glycerolipid biosynthetic process | 2 (0.16%) | 5 (0.15%) | 0.604579 | 0.999992 |
| 526 | [GO:0045861](file:///E:\2018-7-3%E7%81%AB%E9%BE%99%E6%9E%9C%E8%BD%AC%E5%BD%95%E7%BB%84%E6%B5%8B%E5%BA%8F\%E5%AE%8C%E6%95%B4%E7%89%88%E6%95%B0%E6%8D%AE\GDR3855-Hylocereus_undulatus_Britt-12-RNAseq_result\4_Function\2_Group_Diff_Function\UP_DOWN\GO\NL-VS-L1.P.html#gene526) | negative regulation of proteolysis | 2 (0.16%) | 5 (0.15%) | 0.604579 | 0.999992 |
| 527 | [GO:0046474](file:///E:\2018-7-3%E7%81%AB%E9%BE%99%E6%9E%9C%E8%BD%AC%E5%BD%95%E7%BB%84%E6%B5%8B%E5%BA%8F\%E5%AE%8C%E6%95%B4%E7%89%88%E6%95%B0%E6%8D%AE\GDR3855-Hylocereus_undulatus_Britt-12-RNAseq_result\4_Function\2_Group_Diff_Function\UP_DOWN\GO\NL-VS-L1.P.html#gene527) | glycerophospholipid biosynthetic process | 2 (0.16%) | 5 (0.15%) | 0.604579 | 0.999992 |
| 528 | [GO:0051346](file:///E:\2018-7-3%E7%81%AB%E9%BE%99%E6%9E%9C%E8%BD%AC%E5%BD%95%E7%BB%84%E6%B5%8B%E5%BA%8F\%E5%AE%8C%E6%95%B4%E7%89%88%E6%95%B0%E6%8D%AE\GDR3855-Hylocereus_undulatus_Britt-12-RNAseq_result\4_Function\2_Group_Diff_Function\UP_DOWN\GO\NL-VS-L1.P.html#gene528) | negative regulation of hydrolase activity | 2 (0.16%) | 5 (0.15%) | 0.604579 | 0.999992 |
| 529 | [GO:0052547](file:///E:\2018-7-3%E7%81%AB%E9%BE%99%E6%9E%9C%E8%BD%AC%E5%BD%95%E7%BB%84%E6%B5%8B%E5%BA%8F\%E5%AE%8C%E6%95%B4%E7%89%88%E6%95%B0%E6%8D%AE\GDR3855-Hylocereus_undulatus_Britt-12-RNAseq_result\4_Function\2_Group_Diff_Function\UP_DOWN\GO\NL-VS-L1.P.html#gene529) | regulation of peptidase activity | 2 (0.16%) | 5 (0.15%) | 0.604579 | 0.999992 |
| 530 | [GO:0055067](file:///E:\2018-7-3%E7%81%AB%E9%BE%99%E6%9E%9C%E8%BD%AC%E5%BD%95%E7%BB%84%E6%B5%8B%E5%BA%8F\%E5%AE%8C%E6%95%B4%E7%89%88%E6%95%B0%E6%8D%AE\GDR3855-Hylocereus_undulatus_Britt-12-RNAseq_result\4_Function\2_Group_Diff_Function\UP_DOWN\GO\NL-VS-L1.P.html#gene530) | monovalent inorganic cation homeostasis | 2 (0.16%) | 5 (0.15%) | 0.604579 | 0.999992 |
| 531 | [GO:0061640](file:///E:\2018-7-3%E7%81%AB%E9%BE%99%E6%9E%9C%E8%BD%AC%E5%BD%95%E7%BB%84%E6%B5%8B%E5%BA%8F\%E5%AE%8C%E6%95%B4%E7%89%88%E6%95%B0%E6%8D%AE\GDR3855-Hylocereus_undulatus_Britt-12-RNAseq_result\4_Function\2_Group_Diff_Function\UP_DOWN\GO\NL-VS-L1.P.html#gene531) | cytoskeleton-dependent cytokinesis | 2 (0.16%) | 5 (0.15%) | 0.604579 | 0.999992 |
| 532 | [GO:0071322](file:///E:\2018-7-3%E7%81%AB%E9%BE%99%E6%9E%9C%E8%BD%AC%E5%BD%95%E7%BB%84%E6%B5%8B%E5%BA%8F\%E5%AE%8C%E6%95%B4%E7%89%88%E6%95%B0%E6%8D%AE\GDR3855-Hylocereus_undulatus_Britt-12-RNAseq_result\4_Function\2_Group_Diff_Function\UP_DOWN\GO\NL-VS-L1.P.html#gene532) | cellular response to carbohydrate stimulus | 2 (0.16%) | 5 (0.15%) | 0.604579 | 0.999992 |
| 533 | [GO:0090351](file:///E:\2018-7-3%E7%81%AB%E9%BE%99%E6%9E%9C%E8%BD%AC%E5%BD%95%E7%BB%84%E6%B5%8B%E5%BA%8F\%E5%AE%8C%E6%95%B4%E7%89%88%E6%95%B0%E6%8D%AE\GDR3855-Hylocereus_undulatus_Britt-12-RNAseq_result\4_Function\2_Group_Diff_Function\UP_DOWN\GO\NL-VS-L1.P.html#gene533) | seedling development | 2 (0.16%) | 5 (0.15%) | 0.604579 | 0.999992 |
| 534 | [GO:0000902](file:///E:\2018-7-3%E7%81%AB%E9%BE%99%E6%9E%9C%E8%BD%AC%E5%BD%95%E7%BB%84%E6%B5%8B%E5%BA%8F\%E5%AE%8C%E6%95%B4%E7%89%88%E6%95%B0%E6%8D%AE\GDR3855-Hylocereus_undulatus_Britt-12-RNAseq_result\4_Function\2_Group_Diff_Function\UP_DOWN\GO\NL-VS-L1.P.html#gene534) | cell morphogenesis | 9 (0.73%) | 25 (0.74%) | 0.605231 | 0.999992 |
| 535 | [GO:0033554](file:///E:\2018-7-3%E7%81%AB%E9%BE%99%E6%9E%9C%E8%BD%AC%E5%BD%95%E7%BB%84%E6%B5%8B%E5%BA%8F\%E5%AE%8C%E6%95%B4%E7%89%88%E6%95%B0%E6%8D%AE\GDR3855-Hylocereus_undulatus_Britt-12-RNAseq_result\4_Function\2_Group_Diff_Function\UP_DOWN\GO\NL-VS-L1.P.html#gene535) | cellular response to stress | 24 (1.94%) | 67 (1.99%) | 0.608233 | 0.999992 |
| 536 | [GO:0006952](file:///E:\2018-7-3%E7%81%AB%E9%BE%99%E6%9E%9C%E8%BD%AC%E5%BD%95%E7%BB%84%E6%B5%8B%E5%BA%8F\%E5%AE%8C%E6%95%B4%E7%89%88%E6%95%B0%E6%8D%AE\GDR3855-Hylocereus_undulatus_Britt-12-RNAseq_result\4_Function\2_Group_Diff_Function\UP_DOWN\GO\NL-VS-L1.P.html#gene536) | defense response | 15 (1.22%) | 42 (1.25%) | 0.612564 | 0.999992 |
| 537 | [GO:0002684](file:///E:\2018-7-3%E7%81%AB%E9%BE%99%E6%9E%9C%E8%BD%AC%E5%BD%95%E7%BB%84%E6%B5%8B%E5%BA%8F\%E5%AE%8C%E6%95%B4%E7%89%88%E6%95%B0%E6%8D%AE\GDR3855-Hylocereus_undulatus_Britt-12-RNAseq_result\4_Function\2_Group_Diff_Function\UP_DOWN\GO\NL-VS-L1.P.html#gene537) | positive regulation of immune system process | 3 (0.24%) | 8 (0.24%) | 0.613101 | 0.999992 |
| 538 | [GO:0003002](file:///E:\2018-7-3%E7%81%AB%E9%BE%99%E6%9E%9C%E8%BD%AC%E5%BD%95%E7%BB%84%E6%B5%8B%E5%BA%8F\%E5%AE%8C%E6%95%B4%E7%89%88%E6%95%B0%E6%8D%AE\GDR3855-Hylocereus_undulatus_Britt-12-RNAseq_result\4_Function\2_Group_Diff_Function\UP_DOWN\GO\NL-VS-L1.P.html#gene538) | regionalization | 3 (0.24%) | 8 (0.24%) | 0.613101 | 0.999992 |
| 539 | [GO:0007166](file:///E:\2018-7-3%E7%81%AB%E9%BE%99%E6%9E%9C%E8%BD%AC%E5%BD%95%E7%BB%84%E6%B5%8B%E5%BA%8F\%E5%AE%8C%E6%95%B4%E7%89%88%E6%95%B0%E6%8D%AE\GDR3855-Hylocereus_undulatus_Britt-12-RNAseq_result\4_Function\2_Group_Diff_Function\UP_DOWN\GO\NL-VS-L1.P.html#gene539) | cell surface receptor signaling pathway | 3 (0.24%) | 8 (0.24%) | 0.613101 | 0.999992 |
| 540 | [GO:0015931](file:///E:\2018-7-3%E7%81%AB%E9%BE%99%E6%9E%9C%E8%BD%AC%E5%BD%95%E7%BB%84%E6%B5%8B%E5%BA%8F\%E5%AE%8C%E6%95%B4%E7%89%88%E6%95%B0%E6%8D%AE\GDR3855-Hylocereus_undulatus_Britt-12-RNAseq_result\4_Function\2_Group_Diff_Function\UP_DOWN\GO\NL-VS-L1.P.html#gene540) | nucleobase-containing compound transport | 3 (0.24%) | 8 (0.24%) | 0.613101 | 0.999992 |
| 541 | [GO:0031349](file:///E:\2018-7-3%E7%81%AB%E9%BE%99%E6%9E%9C%E8%BD%AC%E5%BD%95%E7%BB%84%E6%B5%8B%E5%BA%8F\%E5%AE%8C%E6%95%B4%E7%89%88%E6%95%B0%E6%8D%AE\GDR3855-Hylocereus_undulatus_Britt-12-RNAseq_result\4_Function\2_Group_Diff_Function\UP_DOWN\GO\NL-VS-L1.P.html#gene541) | positive regulation of defense response | 3 (0.24%) | 8 (0.24%) | 0.613101 | 0.999992 |
| 542 | [GO:0042546](file:///E:\2018-7-3%E7%81%AB%E9%BE%99%E6%9E%9C%E8%BD%AC%E5%BD%95%E7%BB%84%E6%B5%8B%E5%BA%8F\%E5%AE%8C%E6%95%B4%E7%89%88%E6%95%B0%E6%8D%AE\GDR3855-Hylocereus_undulatus_Britt-12-RNAseq_result\4_Function\2_Group_Diff_Function\UP_DOWN\GO\NL-VS-L1.P.html#gene542) | cell wall biogenesis | 3 (0.24%) | 8 (0.24%) | 0.613101 | 0.999992 |
| 543 | [GO:0045088](file:///E:\2018-7-3%E7%81%AB%E9%BE%99%E6%9E%9C%E8%BD%AC%E5%BD%95%E7%BB%84%E6%B5%8B%E5%BA%8F\%E5%AE%8C%E6%95%B4%E7%89%88%E6%95%B0%E6%8D%AE\GDR3855-Hylocereus_undulatus_Britt-12-RNAseq_result\4_Function\2_Group_Diff_Function\UP_DOWN\GO\NL-VS-L1.P.html#gene543) | regulation of innate immune response | 3 (0.24%) | 8 (0.24%) | 0.613101 | 0.999992 |
| 544 | [GO:0045089](file:///E:\2018-7-3%E7%81%AB%E9%BE%99%E6%9E%9C%E8%BD%AC%E5%BD%95%E7%BB%84%E6%B5%8B%E5%BA%8F\%E5%AE%8C%E6%95%B4%E7%89%88%E6%95%B0%E6%8D%AE\GDR3855-Hylocereus_undulatus_Britt-12-RNAseq_result\4_Function\2_Group_Diff_Function\UP_DOWN\GO\NL-VS-L1.P.html#gene544) | positive regulation of innate immune response | 3 (0.24%) | 8 (0.24%) | 0.613101 | 0.999992 |
| 545 | [GO:0048584](file:///E:\2018-7-3%E7%81%AB%E9%BE%99%E6%9E%9C%E8%BD%AC%E5%BD%95%E7%BB%84%E6%B5%8B%E5%BA%8F\%E5%AE%8C%E6%95%B4%E7%89%88%E6%95%B0%E6%8D%AE\GDR3855-Hylocereus_undulatus_Britt-12-RNAseq_result\4_Function\2_Group_Diff_Function\UP_DOWN\GO\NL-VS-L1.P.html#gene545) | positive regulation of response to stimulus | 3 (0.24%) | 8 (0.24%) | 0.613101 | 0.999992 |
| 546 | [GO:0050776](file:///E:\2018-7-3%E7%81%AB%E9%BE%99%E6%9E%9C%E8%BD%AC%E5%BD%95%E7%BB%84%E6%B5%8B%E5%BA%8F\%E5%AE%8C%E6%95%B4%E7%89%88%E6%95%B0%E6%8D%AE\GDR3855-Hylocereus_undulatus_Britt-12-RNAseq_result\4_Function\2_Group_Diff_Function\UP_DOWN\GO\NL-VS-L1.P.html#gene546) | regulation of immune response | 3 (0.24%) | 8 (0.24%) | 0.613101 | 0.999992 |
| 547 | [GO:0050778](file:///E:\2018-7-3%E7%81%AB%E9%BE%99%E6%9E%9C%E8%BD%AC%E5%BD%95%E7%BB%84%E6%B5%8B%E5%BA%8F\%E5%AE%8C%E6%95%B4%E7%89%88%E6%95%B0%E6%8D%AE\GDR3855-Hylocereus_undulatus_Britt-12-RNAseq_result\4_Function\2_Group_Diff_Function\UP_DOWN\GO\NL-VS-L1.P.html#gene547) | positive regulation of immune response | 3 (0.24%) | 8 (0.24%) | 0.613101 | 0.999992 |
| 548 | [GO:0032989](file:///E:\2018-7-3%E7%81%AB%E9%BE%99%E6%9E%9C%E8%BD%AC%E5%BD%95%E7%BB%84%E6%B5%8B%E5%BA%8F\%E5%AE%8C%E6%95%B4%E7%89%88%E6%95%B0%E6%8D%AE\GDR3855-Hylocereus_undulatus_Britt-12-RNAseq_result\4_Function\2_Group_Diff_Function\UP_DOWN\GO\NL-VS-L1.P.html#gene548) | cellular component morphogenesis | 10 (0.81%) | 28 (0.83%) | 0.615174 | 0.999992 |
| 549 | [GO:0019725](file:///E:\2018-7-3%E7%81%AB%E9%BE%99%E6%9E%9C%E8%BD%AC%E5%BD%95%E7%BB%84%E6%B5%8B%E5%BA%8F\%E5%AE%8C%E6%95%B4%E7%89%88%E6%95%B0%E6%8D%AE\GDR3855-Hylocereus_undulatus_Britt-12-RNAseq_result\4_Function\2_Group_Diff_Function\UP_DOWN\GO\NL-VS-L1.P.html#gene549) | cellular homeostasis | 16 (1.3%) | 45 (1.34%) | 0.621122 | 0.999992 |
| 550 | [GO:0032268](file:///E:\2018-7-3%E7%81%AB%E9%BE%99%E6%9E%9C%E8%BD%AC%E5%BD%95%E7%BB%84%E6%B5%8B%E5%BA%8F\%E5%AE%8C%E6%95%B4%E7%89%88%E6%95%B0%E6%8D%AE\GDR3855-Hylocereus_undulatus_Britt-12-RNAseq_result\4_Function\2_Group_Diff_Function\UP_DOWN\GO\NL-VS-L1.P.html#gene550) | regulation of cellular protein metabolic process | 16 (1.3%) | 45 (1.34%) | 0.621122 | 0.999992 |
| 551 | [GO:0051246](file:///E:\2018-7-3%E7%81%AB%E9%BE%99%E6%9E%9C%E8%BD%AC%E5%BD%95%E7%BB%84%E6%B5%8B%E5%BA%8F\%E5%AE%8C%E6%95%B4%E7%89%88%E6%95%B0%E6%8D%AE\GDR3855-Hylocereus_undulatus_Britt-12-RNAseq_result\4_Function\2_Group_Diff_Function\UP_DOWN\GO\NL-VS-L1.P.html#gene551) | regulation of protein metabolic process | 16 (1.3%) | 45 (1.34%) | 0.621122 | 0.999992 |
| 552 | [GO:0006873](file:///E:\2018-7-3%E7%81%AB%E9%BE%99%E6%9E%9C%E8%BD%AC%E5%BD%95%E7%BB%84%E6%B5%8B%E5%BA%8F\%E5%AE%8C%E6%95%B4%E7%89%88%E6%95%B0%E6%8D%AE\GDR3855-Hylocereus_undulatus_Britt-12-RNAseq_result\4_Function\2_Group_Diff_Function\UP_DOWN\GO\NL-VS-L1.P.html#gene552) | cellular ion homeostasis | 4 (0.32%) | 11 (0.33%) | 0.621908 | 0.999992 |
| 553 | [GO:0016128](file:///E:\2018-7-3%E7%81%AB%E9%BE%99%E6%9E%9C%E8%BD%AC%E5%BD%95%E7%BB%84%E6%B5%8B%E5%BA%8F\%E5%AE%8C%E6%95%B4%E7%89%88%E6%95%B0%E6%8D%AE\GDR3855-Hylocereus_undulatus_Britt-12-RNAseq_result\4_Function\2_Group_Diff_Function\UP_DOWN\GO\NL-VS-L1.P.html#gene553) | phytosteroid metabolic process | 4 (0.32%) | 11 (0.33%) | 0.621908 | 0.999992 |
| 554 | [GO:0016129](file:///E:\2018-7-3%E7%81%AB%E9%BE%99%E6%9E%9C%E8%BD%AC%E5%BD%95%E7%BB%84%E6%B5%8B%E5%BA%8F\%E5%AE%8C%E6%95%B4%E7%89%88%E6%95%B0%E6%8D%AE\GDR3855-Hylocereus_undulatus_Britt-12-RNAseq_result\4_Function\2_Group_Diff_Function\UP_DOWN\GO\NL-VS-L1.P.html#gene554) | phytosteroid biosynthetic process | 4 (0.32%) | 11 (0.33%) | 0.621908 | 0.999992 |
| 555 | [GO:0044711](file:///E:\2018-7-3%E7%81%AB%E9%BE%99%E6%9E%9C%E8%BD%AC%E5%BD%95%E7%BB%84%E6%B5%8B%E5%BA%8F\%E5%AE%8C%E6%95%B4%E7%89%88%E6%95%B0%E6%8D%AE\GDR3855-Hylocereus_undulatus_Britt-12-RNAseq_result\4_Function\2_Group_Diff_Function\UP_DOWN\GO\NL-VS-L1.P.html#gene555) | single-organism biosynthetic process | 57 (4.62%) | 159 (4.73%) | 0.624012 | 0.999992 |
| 556 | [GO:0022613](file:///E:\2018-7-3%E7%81%AB%E9%BE%99%E6%9E%9C%E8%BD%AC%E5%BD%95%E7%BB%84%E6%B5%8B%E5%BA%8F\%E5%AE%8C%E6%95%B4%E7%89%88%E6%95%B0%E6%8D%AE\GDR3855-Hylocereus_undulatus_Britt-12-RNAseq_result\4_Function\2_Group_Diff_Function\UP_DOWN\GO\NL-VS-L1.P.html#gene556) | ribonucleoprotein complex biogenesis | 11 (0.89%) | 31 (0.92%) | 0.624357 | 0.999992 |
| 557 | [GO:0048878](file:///E:\2018-7-3%E7%81%AB%E9%BE%99%E6%9E%9C%E8%BD%AC%E5%BD%95%E7%BB%84%E6%B5%8B%E5%BA%8F\%E5%AE%8C%E6%95%B4%E7%89%88%E6%95%B0%E6%8D%AE\GDR3855-Hylocereus_undulatus_Britt-12-RNAseq_result\4_Function\2_Group_Diff_Function\UP_DOWN\GO\NL-VS-L1.P.html#gene557) | chemical homeostasis | 11 (0.89%) | 31 (0.92%) | 0.624357 | 0.999992 |
| 558 | [GO:0006629](file:///E:\2018-7-3%E7%81%AB%E9%BE%99%E6%9E%9C%E8%BD%AC%E5%BD%95%E7%BB%84%E6%B5%8B%E5%BA%8F\%E5%AE%8C%E6%95%B4%E7%89%88%E6%95%B0%E6%8D%AE\GDR3855-Hylocereus_undulatus_Britt-12-RNAseq_result\4_Function\2_Group_Diff_Function\UP_DOWN\GO\NL-VS-L1.P.html#gene558) | lipid metabolic process | 45 (3.65%) | 126 (3.75%) | 0.629252 | 0.999992 |
| 559 | [GO:0008219](file:///E:\2018-7-3%E7%81%AB%E9%BE%99%E6%9E%9C%E8%BD%AC%E5%BD%95%E7%BB%84%E6%B5%8B%E5%BA%8F\%E5%AE%8C%E6%95%B4%E7%89%88%E6%95%B0%E6%8D%AE\GDR3855-Hylocereus_undulatus_Britt-12-RNAseq_result\4_Function\2_Group_Diff_Function\UP_DOWN\GO\NL-VS-L1.P.html#gene559) | cell death | 5 (0.41%) | 14 (0.42%) | 0.630405 | 0.999992 |
| 560 | [GO:0016458](file:///E:\2018-7-3%E7%81%AB%E9%BE%99%E6%9E%9C%E8%BD%AC%E5%BD%95%E7%BB%84%E6%B5%8B%E5%BA%8F\%E5%AE%8C%E6%95%B4%E7%89%88%E6%95%B0%E6%8D%AE\GDR3855-Hylocereus_undulatus_Britt-12-RNAseq_result\4_Function\2_Group_Diff_Function\UP_DOWN\GO\NL-VS-L1.P.html#gene560) | gene silencing | 5 (0.41%) | 14 (0.42%) | 0.630405 | 0.999992 |
| 561 | [GO:1902600](file:///E:\2018-7-3%E7%81%AB%E9%BE%99%E6%9E%9C%E8%BD%AC%E5%BD%95%E7%BB%84%E6%B5%8B%E5%BA%8F\%E5%AE%8C%E6%95%B4%E7%89%88%E6%95%B0%E6%8D%AE\GDR3855-Hylocereus_undulatus_Britt-12-RNAseq_result\4_Function\2_Group_Diff_Function\UP_DOWN\GO\NL-VS-L1.P.html#gene561) | hydrogen ion transmembrane transport | 6 (0.49%) | 17 (0.51%) | 0.638482 | 0.999992 |
| 562 | [GO:0001932](file:///E:\2018-7-3%E7%81%AB%E9%BE%99%E6%9E%9C%E8%BD%AC%E5%BD%95%E7%BB%84%E6%B5%8B%E5%BA%8F\%E5%AE%8C%E6%95%B4%E7%89%88%E6%95%B0%E6%8D%AE\GDR3855-Hylocereus_undulatus_Britt-12-RNAseq_result\4_Function\2_Group_Diff_Function\UP_DOWN\GO\NL-VS-L1.P.html#gene562) | regulation of protein phosphorylation | 13 (1.05%) | 37 (1.1%) | 0.640931 | 0.999992 |
| 563 | [GO:0031399](file:///E:\2018-7-3%E7%81%AB%E9%BE%99%E6%9E%9C%E8%BD%AC%E5%BD%95%E7%BB%84%E6%B5%8B%E5%BA%8F\%E5%AE%8C%E6%95%B4%E7%89%88%E6%95%B0%E6%8D%AE\GDR3855-Hylocereus_undulatus_Britt-12-RNAseq_result\4_Function\2_Group_Diff_Function\UP_DOWN\GO\NL-VS-L1.P.html#gene563) | regulation of protein modification process | 13 (1.05%) | 37 (1.1%) | 0.640931 | 0.999992 |
| 564 | [GO:0042325](file:///E:\2018-7-3%E7%81%AB%E9%BE%99%E6%9E%9C%E8%BD%AC%E5%BD%95%E7%BB%84%E6%B5%8B%E5%BA%8F\%E5%AE%8C%E6%95%B4%E7%89%88%E6%95%B0%E6%8D%AE\GDR3855-Hylocereus_undulatus_Britt-12-RNAseq_result\4_Function\2_Group_Diff_Function\UP_DOWN\GO\NL-VS-L1.P.html#gene564) | regulation of phosphorylation | 13 (1.05%) | 37 (1.1%) | 0.640931 | 0.999992 |
| 565 | [GO:0044255](file:///E:\2018-7-3%E7%81%AB%E9%BE%99%E6%9E%9C%E8%BD%AC%E5%BD%95%E7%BB%84%E6%B5%8B%E5%BA%8F\%E5%AE%8C%E6%95%B4%E7%89%88%E6%95%B0%E6%8D%AE\GDR3855-Hylocereus_undulatus_Britt-12-RNAseq_result\4_Function\2_Group_Diff_Function\UP_DOWN\GO\NL-VS-L1.P.html#gene565) | cellular lipid metabolic process | 38 (3.08%) | 107 (3.19%) | 0.640963 | 0.999992 |
| 566 | [GO:0009892](file:///E:\2018-7-3%E7%81%AB%E9%BE%99%E6%9E%9C%E8%BD%AC%E5%BD%95%E7%BB%84%E6%B5%8B%E5%BA%8F\%E5%AE%8C%E6%95%B4%E7%89%88%E6%95%B0%E6%8D%AE\GDR3855-Hylocereus_undulatus_Britt-12-RNAseq_result\4_Function\2_Group_Diff_Function\UP_DOWN\GO\NL-VS-L1.P.html#gene566) | negative regulation of metabolic process | 7 (0.57%) | 20 (0.6%) | 0.646140 | 0.999992 |
| 567 | [GO:0010605](file:///E:\2018-7-3%E7%81%AB%E9%BE%99%E6%9E%9C%E8%BD%AC%E5%BD%95%E7%BB%84%E6%B5%8B%E5%BA%8F\%E5%AE%8C%E6%95%B4%E7%89%88%E6%95%B0%E6%8D%AE\GDR3855-Hylocereus_undulatus_Britt-12-RNAseq_result\4_Function\2_Group_Diff_Function\UP_DOWN\GO\NL-VS-L1.P.html#gene567) | negative regulation of macromolecule metabolic process | 7 (0.57%) | 20 (0.6%) | 0.646140 | 0.999992 |
| 568 | [GO:0015992](file:///E:\2018-7-3%E7%81%AB%E9%BE%99%E6%9E%9C%E8%BD%AC%E5%BD%95%E7%BB%84%E6%B5%8B%E5%BA%8F\%E5%AE%8C%E6%95%B4%E7%89%88%E6%95%B0%E6%8D%AE\GDR3855-Hylocereus_undulatus_Britt-12-RNAseq_result\4_Function\2_Group_Diff_Function\UP_DOWN\GO\NL-VS-L1.P.html#gene568) | proton transport | 14 (1.13%) | 40 (1.19%) | 0.648494 | 0.999992 |
| 569 | [GO:0015980](file:///E:\2018-7-3%E7%81%AB%E9%BE%99%E6%9E%9C%E8%BD%AC%E5%BD%95%E7%BB%84%E6%B5%8B%E5%BA%8F\%E5%AE%8C%E6%95%B4%E7%89%88%E6%95%B0%E6%8D%AE\GDR3855-Hylocereus_undulatus_Britt-12-RNAseq_result\4_Function\2_Group_Diff_Function\UP_DOWN\GO\NL-VS-L1.P.html#gene569) | energy derivation by oxidation of organic compounds | 15 (1.22%) | 43 (1.28%) | 0.655659 | 0.999992 |
| 570 | [GO:1901615](file:///E:\2018-7-3%E7%81%AB%E9%BE%99%E6%9E%9C%E8%BD%AC%E5%BD%95%E7%BB%84%E6%B5%8B%E5%BA%8F\%E5%AE%8C%E6%95%B4%E7%89%88%E6%95%B0%E6%8D%AE\GDR3855-Hylocereus_undulatus_Britt-12-RNAseq_result\4_Function\2_Group_Diff_Function\UP_DOWN\GO\NL-VS-L1.P.html#gene570) | organic hydroxy compound metabolic process | 9 (0.73%) | 26 (0.77%) | 0.660324 | 0.999992 |
| 571 | [GO:0048869](file:///E:\2018-7-3%E7%81%AB%E9%BE%99%E6%9E%9C%E8%BD%AC%E5%BD%95%E7%BB%84%E6%B5%8B%E5%BA%8F\%E5%AE%8C%E6%95%B4%E7%89%88%E6%95%B0%E6%8D%AE\GDR3855-Hylocereus_undulatus_Britt-12-RNAseq_result\4_Function\2_Group_Diff_Function\UP_DOWN\GO\NL-VS-L1.P.html#gene571) | cellular developmental process | 16 (1.3%) | 46 (1.37%) | 0.662474 | 0.999992 |
| 572 | [GO:0042592](file:///E:\2018-7-3%E7%81%AB%E9%BE%99%E6%9E%9C%E8%BD%AC%E5%BD%95%E7%BB%84%E6%B5%8B%E5%BA%8F\%E5%AE%8C%E6%95%B4%E7%89%88%E6%95%B0%E6%8D%AE\GDR3855-Hylocereus_undulatus_Britt-12-RNAseq_result\4_Function\2_Group_Diff_Function\UP_DOWN\GO\NL-VS-L1.P.html#gene572) | homeostatic process | 23 (1.86%) | 66 (1.96%) | 0.670422 | 0.999992 |
| 573 | [GO:0051604](file:///E:\2018-7-3%E7%81%AB%E9%BE%99%E6%9E%9C%E8%BD%AC%E5%BD%95%E7%BB%84%E6%B5%8B%E5%BA%8F\%E5%AE%8C%E6%95%B4%E7%89%88%E6%95%B0%E6%8D%AE\GDR3855-Hylocereus_undulatus_Britt-12-RNAseq_result\4_Function\2_Group_Diff_Function\UP_DOWN\GO\NL-VS-L1.P.html#gene573) | protein maturation | 31 (2.51%) | 89 (2.65%) | 0.685052 | 0.999992 |
| 574 | [GO:0019220](file:///E:\2018-7-3%E7%81%AB%E9%BE%99%E6%9E%9C%E8%BD%AC%E5%BD%95%E7%BB%84%E6%B5%8B%E5%BA%8F\%E5%AE%8C%E6%95%B4%E7%89%88%E6%95%B0%E6%8D%AE\GDR3855-Hylocereus_undulatus_Britt-12-RNAseq_result\4_Function\2_Group_Diff_Function\UP_DOWN\GO\NL-VS-L1.P.html#gene574) | regulation of phosphate metabolic process | 13 (1.05%) | 38 (1.13%) | 0.685086 | 0.999992 |
| 575 | [GO:0043085](file:///E:\2018-7-3%E7%81%AB%E9%BE%99%E6%9E%9C%E8%BD%AC%E5%BD%95%E7%BB%84%E6%B5%8B%E5%BA%8F\%E5%AE%8C%E6%95%B4%E7%89%88%E6%95%B0%E6%8D%AE\GDR3855-Hylocereus_undulatus_Britt-12-RNAseq_result\4_Function\2_Group_Diff_Function\UP_DOWN\GO\NL-VS-L1.P.html#gene575) | positive regulation of catalytic activity | 13 (1.05%) | 38 (1.13%) | 0.685086 | 0.999992 |
| 576 | [GO:0051174](file:///E:\2018-7-3%E7%81%AB%E9%BE%99%E6%9E%9C%E8%BD%AC%E5%BD%95%E7%BB%84%E6%B5%8B%E5%BA%8F\%E5%AE%8C%E6%95%B4%E7%89%88%E6%95%B0%E6%8D%AE\GDR3855-Hylocereus_undulatus_Britt-12-RNAseq_result\4_Function\2_Group_Diff_Function\UP_DOWN\GO\NL-VS-L1.P.html#gene576) | regulation of phosphorus metabolic process | 13 (1.05%) | 38 (1.13%) | 0.685086 | 0.999992 |
| 577 | [GO:0044093](file:///E:\2018-7-3%E7%81%AB%E9%BE%99%E6%9E%9C%E8%BD%AC%E5%BD%95%E7%BB%84%E6%B5%8B%E5%BA%8F\%E5%AE%8C%E6%95%B4%E7%89%88%E6%95%B0%E6%8D%AE\GDR3855-Hylocereus_undulatus_Britt-12-RNAseq_result\4_Function\2_Group_Diff_Function\UP_DOWN\GO\NL-VS-L1.P.html#gene577) | positive regulation of molecular function | 14 (1.13%) | 41 (1.22%) | 0.690678 | 0.999992 |
| 578 | [GO:0042743](file:///E:\2018-7-3%E7%81%AB%E9%BE%99%E6%9E%9C%E8%BD%AC%E5%BD%95%E7%BB%84%E6%B5%8B%E5%BA%8F\%E5%AE%8C%E6%95%B4%E7%89%88%E6%95%B0%E6%8D%AE\GDR3855-Hylocereus_undulatus_Britt-12-RNAseq_result\4_Function\2_Group_Diff_Function\UP_DOWN\GO\NL-VS-L1.P.html#gene578) | hydrogen peroxide metabolic process | 4 (0.32%) | 12 (0.36%) | 0.699139 | 0.999992 |
| 579 | [GO:0048589](file:///E:\2018-7-3%E7%81%AB%E9%BE%99%E6%9E%9C%E8%BD%AC%E5%BD%95%E7%BB%84%E6%B5%8B%E5%BA%8F\%E5%AE%8C%E6%95%B4%E7%89%88%E6%95%B0%E6%8D%AE\GDR3855-Hylocereus_undulatus_Britt-12-RNAseq_result\4_Function\2_Group_Diff_Function\UP_DOWN\GO\NL-VS-L1.P.html#gene579) | developmental growth | 4 (0.32%) | 12 (0.36%) | 0.699139 | 0.999992 |
| 580 | [GO:0080134](file:///E:\2018-7-3%E7%81%AB%E9%BE%99%E6%9E%9C%E8%BD%AC%E5%BD%95%E7%BB%84%E6%B5%8B%E5%BA%8F\%E5%AE%8C%E6%95%B4%E7%89%88%E6%95%B0%E6%8D%AE\GDR3855-Hylocereus_undulatus_Britt-12-RNAseq_result\4_Function\2_Group_Diff_Function\UP_DOWN\GO\NL-VS-L1.P.html#gene580) | regulation of response to stress | 4 (0.32%) | 12 (0.36%) | 0.699139 | 0.999992 |
| 581 | [GO:1901701](file:///E:\2018-7-3%E7%81%AB%E9%BE%99%E6%9E%9C%E8%BD%AC%E5%BD%95%E7%BB%84%E6%B5%8B%E5%BA%8F\%E5%AE%8C%E6%95%B4%E7%89%88%E6%95%B0%E6%8D%AE\GDR3855-Hylocereus_undulatus_Britt-12-RNAseq_result\4_Function\2_Group_Diff_Function\UP_DOWN\GO\NL-VS-L1.P.html#gene581) | cellular response to oxygen-containing compound | 4 (0.32%) | 12 (0.36%) | 0.699139 | 0.999992 |
| 582 | [GO:0010073](file:///E:\2018-7-3%E7%81%AB%E9%BE%99%E6%9E%9C%E8%BD%AC%E5%BD%95%E7%BB%84%E6%B5%8B%E5%BA%8F\%E5%AE%8C%E6%95%B4%E7%89%88%E6%95%B0%E6%8D%AE\GDR3855-Hylocereus_undulatus_Britt-12-RNAseq_result\4_Function\2_Group_Diff_Function\UP_DOWN\GO\NL-VS-L1.P.html#gene582) | meristem maintenance | 5 (0.41%) | 15 (0.45%) | 0.699320 | 0.999992 |
| 583 | [GO:0010629](file:///E:\2018-7-3%E7%81%AB%E9%BE%99%E6%9E%9C%E8%BD%AC%E5%BD%95%E7%BB%84%E6%B5%8B%E5%BA%8F\%E5%AE%8C%E6%95%B4%E7%89%88%E6%95%B0%E6%8D%AE\GDR3855-Hylocereus_undulatus_Britt-12-RNAseq_result\4_Function\2_Group_Diff_Function\UP_DOWN\GO\NL-VS-L1.P.html#gene583) | negative regulation of gene expression | 5 (0.41%) | 15 (0.45%) | 0.699320 | 0.999992 |
| 584 | [GO:0033014](file:///E:\2018-7-3%E7%81%AB%E9%BE%99%E6%9E%9C%E8%BD%AC%E5%BD%95%E7%BB%84%E6%B5%8B%E5%BA%8F\%E5%AE%8C%E6%95%B4%E7%89%88%E6%95%B0%E6%8D%AE\GDR3855-Hylocereus_undulatus_Britt-12-RNAseq_result\4_Function\2_Group_Diff_Function\UP_DOWN\GO\NL-VS-L1.P.html#gene584) | tetrapyrrole biosynthetic process | 5 (0.41%) | 15 (0.45%) | 0.699320 | 0.999992 |
| 585 | [GO:0046165](file:///E:\2018-7-3%E7%81%AB%E9%BE%99%E6%9E%9C%E8%BD%AC%E5%BD%95%E7%BB%84%E6%B5%8B%E5%BA%8F\%E5%AE%8C%E6%95%B4%E7%89%88%E6%95%B0%E6%8D%AE\GDR3855-Hylocereus_undulatus_Britt-12-RNAseq_result\4_Function\2_Group_Diff_Function\UP_DOWN\GO\NL-VS-L1.P.html#gene585) | alcohol biosynthetic process | 5 (0.41%) | 15 (0.45%) | 0.699320 | 0.999992 |
| 586 | [GO:0051128](file:///E:\2018-7-3%E7%81%AB%E9%BE%99%E6%9E%9C%E8%BD%AC%E5%BD%95%E7%BB%84%E6%B5%8B%E5%BA%8F\%E5%AE%8C%E6%95%B4%E7%89%88%E6%95%B0%E6%8D%AE\GDR3855-Hylocereus_undulatus_Britt-12-RNAseq_result\4_Function\2_Group_Diff_Function\UP_DOWN\GO\NL-VS-L1.P.html#gene586) | regulation of cellular component organization | 5 (0.41%) | 15 (0.45%) | 0.699320 | 0.999992 |
| 587 | [GO:0006066](file:///E:\2018-7-3%E7%81%AB%E9%BE%99%E6%9E%9C%E8%BD%AC%E5%BD%95%E7%BB%84%E6%B5%8B%E5%BA%8F\%E5%AE%8C%E6%95%B4%E7%89%88%E6%95%B0%E6%8D%AE\GDR3855-Hylocereus_undulatus_Britt-12-RNAseq_result\4_Function\2_Group_Diff_Function\UP_DOWN\GO\NL-VS-L1.P.html#gene587) | alcohol metabolic process | 6 (0.49%) | 18 (0.54%) | 0.701141 | 0.999992 |
| 588 | [GO:0048583](file:///E:\2018-7-3%E7%81%AB%E9%BE%99%E6%9E%9C%E8%BD%AC%E5%BD%95%E7%BB%84%E6%B5%8B%E5%BA%8F\%E5%AE%8C%E6%95%B4%E7%89%88%E6%95%B0%E6%8D%AE\GDR3855-Hylocereus_undulatus_Britt-12-RNAseq_result\4_Function\2_Group_Diff_Function\UP_DOWN\GO\NL-VS-L1.P.html#gene588) | regulation of response to stimulus | 6 (0.49%) | 18 (0.54%) | 0.701141 | 0.999992 |
| 589 | [GO:0016071](file:///E:\2018-7-3%E7%81%AB%E9%BE%99%E6%9E%9C%E8%BD%AC%E5%BD%95%E7%BB%84%E6%B5%8B%E5%BA%8F\%E5%AE%8C%E6%95%B4%E7%89%88%E6%95%B0%E6%8D%AE\GDR3855-Hylocereus_undulatus_Britt-12-RNAseq_result\4_Function\2_Group_Diff_Function\UP_DOWN\GO\NL-VS-L1.P.html#gene589) | mRNA metabolic process | 3 (0.24%) | 9 (0.27%) | 0.702206 | 0.999992 |
| 590 | [GO:0040029](file:///E:\2018-7-3%E7%81%AB%E9%BE%99%E6%9E%9C%E8%BD%AC%E5%BD%95%E7%BB%84%E6%B5%8B%E5%BA%8F\%E5%AE%8C%E6%95%B4%E7%89%88%E6%95%B0%E6%8D%AE\GDR3855-Hylocereus_undulatus_Britt-12-RNAseq_result\4_Function\2_Group_Diff_Function\UP_DOWN\GO\NL-VS-L1.P.html#gene590) | regulation of gene expression, epigenetic | 3 (0.24%) | 9 (0.27%) | 0.702206 | 0.999992 |
| 591 | [GO:0042886](file:///E:\2018-7-3%E7%81%AB%E9%BE%99%E6%9E%9C%E8%BD%AC%E5%BD%95%E7%BB%84%E6%B5%8B%E5%BA%8F\%E5%AE%8C%E6%95%B4%E7%89%88%E6%95%B0%E6%8D%AE\GDR3855-Hylocereus_undulatus_Britt-12-RNAseq_result\4_Function\2_Group_Diff_Function\UP_DOWN\GO\NL-VS-L1.P.html#gene591) | amide transport | 3 (0.24%) | 9 (0.27%) | 0.702206 | 0.999992 |
| 592 | [GO:0070085](file:///E:\2018-7-3%E7%81%AB%E9%BE%99%E6%9E%9C%E8%BD%AC%E5%BD%95%E7%BB%84%E6%B5%8B%E5%BA%8F\%E5%AE%8C%E6%95%B4%E7%89%88%E6%95%B0%E6%8D%AE\GDR3855-Hylocereus_undulatus_Britt-12-RNAseq_result\4_Function\2_Group_Diff_Function\UP_DOWN\GO\NL-VS-L1.P.html#gene592) | glycosylation | 3 (0.24%) | 9 (0.27%) | 0.702206 | 0.999992 |
| 593 | [GO:0090558](file:///E:\2018-7-3%E7%81%AB%E9%BE%99%E6%9E%9C%E8%BD%AC%E5%BD%95%E7%BB%84%E6%B5%8B%E5%BA%8F\%E5%AE%8C%E6%95%B4%E7%89%88%E6%95%B0%E6%8D%AE\GDR3855-Hylocereus_undulatus_Britt-12-RNAseq_result\4_Function\2_Group_Diff_Function\UP_DOWN\GO\NL-VS-L1.P.html#gene593) | plant epidermis development | 3 (0.24%) | 9 (0.27%) | 0.702206 | 0.999992 |
| 594 | [GO:0010468](file:///E:\2018-7-3%E7%81%AB%E9%BE%99%E6%9E%9C%E8%BD%AC%E5%BD%95%E7%BB%84%E6%B5%8B%E5%BA%8F\%E5%AE%8C%E6%95%B4%E7%89%88%E6%95%B0%E6%8D%AE\GDR3855-Hylocereus_undulatus_Britt-12-RNAseq_result\4_Function\2_Group_Diff_Function\UP_DOWN\GO\NL-VS-L1.P.html#gene594) | regulation of gene expression | 7 (0.57%) | 21 (0.63%) | 0.703866 | 0.999992 |
| 595 | [GO:0009060](file:///E:\2018-7-3%E7%81%AB%E9%BE%99%E6%9E%9C%E8%BD%AC%E5%BD%95%E7%BB%84%E6%B5%8B%E5%BA%8F\%E5%AE%8C%E6%95%B4%E7%89%88%E6%95%B0%E6%8D%AE\GDR3855-Hylocereus_undulatus_Britt-12-RNAseq_result\4_Function\2_Group_Diff_Function\UP_DOWN\GO\NL-VS-L1.P.html#gene595) | aerobic respiration | 8 (0.65%) | 24 (0.71%) | 0.707109 | 0.999992 |
| 596 | [GO:0006810](file:///E:\2018-7-3%E7%81%AB%E9%BE%99%E6%9E%9C%E8%BD%AC%E5%BD%95%E7%BB%84%E6%B5%8B%E5%BA%8F\%E5%AE%8C%E6%95%B4%E7%89%88%E6%95%B0%E6%8D%AE\GDR3855-Hylocereus_undulatus_Britt-12-RNAseq_result\4_Function\2_Group_Diff_Function\UP_DOWN\GO\NL-VS-L1.P.html#gene596) | transport | 149 (12.07%) | 418 (12.44%) | 0.707532 | 0.999992 |
| 597 | [GO:0002218](file:///E:\2018-7-3%E7%81%AB%E9%BE%99%E6%9E%9C%E8%BD%AC%E5%BD%95%E7%BB%84%E6%B5%8B%E5%BA%8F\%E5%AE%8C%E6%95%B4%E7%89%88%E6%95%B0%E6%8D%AE\GDR3855-Hylocereus_undulatus_Britt-12-RNAseq_result\4_Function\2_Group_Diff_Function\UP_DOWN\GO\NL-VS-L1.P.html#gene597) | activation of innate immune response | 2 (0.16%) | 6 (0.18%) | 0.712757 | 0.999992 |
| 598 | [GO:0002253](file:///E:\2018-7-3%E7%81%AB%E9%BE%99%E6%9E%9C%E8%BD%AC%E5%BD%95%E7%BB%84%E6%B5%8B%E5%BA%8F\%E5%AE%8C%E6%95%B4%E7%89%88%E6%95%B0%E6%8D%AE\GDR3855-Hylocereus_undulatus_Britt-12-RNAseq_result\4_Function\2_Group_Diff_Function\UP_DOWN\GO\NL-VS-L1.P.html#gene598) | activation of immune response | 2 (0.16%) | 6 (0.18%) | 0.712757 | 0.999992 |
| 599 | [GO:0006563](file:///E:\2018-7-3%E7%81%AB%E9%BE%99%E6%9E%9C%E8%BD%AC%E5%BD%95%E7%BB%84%E6%B5%8B%E5%BA%8F\%E5%AE%8C%E6%95%B4%E7%89%88%E6%95%B0%E6%8D%AE\GDR3855-Hylocereus_undulatus_Britt-12-RNAseq_result\4_Function\2_Group_Diff_Function\UP_DOWN\GO\NL-VS-L1.P.html#gene599) | L-serine metabolic process | 2 (0.16%) | 6 (0.18%) | 0.712757 | 0.999992 |
| 600 | [GO:0006984](file:///E:\2018-7-3%E7%81%AB%E9%BE%99%E6%9E%9C%E8%BD%AC%E5%BD%95%E7%BB%84%E6%B5%8B%E5%BA%8F\%E5%AE%8C%E6%95%B4%E7%89%88%E6%95%B0%E6%8D%AE\GDR3855-Hylocereus_undulatus_Britt-12-RNAseq_result\4_Function\2_Group_Diff_Function\UP_DOWN\GO\NL-VS-L1.P.html#gene600) | ER-nucleus signaling pathway | 2 (0.16%) | 6 (0.18%) | 0.712757 | 0.999992 |
| 601 | [GO:0008654](file:///E:\2018-7-3%E7%81%AB%E9%BE%99%E6%9E%9C%E8%BD%AC%E5%BD%95%E7%BB%84%E6%B5%8B%E5%BA%8F\%E5%AE%8C%E6%95%B4%E7%89%88%E6%95%B0%E6%8D%AE\GDR3855-Hylocereus_undulatus_Britt-12-RNAseq_result\4_Function\2_Group_Diff_Function\UP_DOWN\GO\NL-VS-L1.P.html#gene601) | phospholipid biosynthetic process | 2 (0.16%) | 6 (0.18%) | 0.712757 | 0.999992 |
| 602 | [GO:0009132](file:///E:\2018-7-3%E7%81%AB%E9%BE%99%E6%9E%9C%E8%BD%AC%E5%BD%95%E7%BB%84%E6%B5%8B%E5%BA%8F\%E5%AE%8C%E6%95%B4%E7%89%88%E6%95%B0%E6%8D%AE\GDR3855-Hylocereus_undulatus_Britt-12-RNAseq_result\4_Function\2_Group_Diff_Function\UP_DOWN\GO\NL-VS-L1.P.html#gene602) | nucleoside diphosphate metabolic process | 2 (0.16%) | 6 (0.18%) | 0.712757 | 0.999992 |
| 603 | [GO:0009630](file:///E:\2018-7-3%E7%81%AB%E9%BE%99%E6%9E%9C%E8%BD%AC%E5%BD%95%E7%BB%84%E6%B5%8B%E5%BA%8F\%E5%AE%8C%E6%95%B4%E7%89%88%E6%95%B0%E6%8D%AE\GDR3855-Hylocereus_undulatus_Britt-12-RNAseq_result\4_Function\2_Group_Diff_Function\UP_DOWN\GO\NL-VS-L1.P.html#gene603) | gravitropism | 2 (0.16%) | 6 (0.18%) | 0.712757 | 0.999992 |
| 604 | [GO:0010118](file:///E:\2018-7-3%E7%81%AB%E9%BE%99%E6%9E%9C%E8%BD%AC%E5%BD%95%E7%BB%84%E6%B5%8B%E5%BA%8F\%E5%AE%8C%E6%95%B4%E7%89%88%E6%95%B0%E6%8D%AE\GDR3855-Hylocereus_undulatus_Britt-12-RNAseq_result\4_Function\2_Group_Diff_Function\UP_DOWN\GO\NL-VS-L1.P.html#gene604) | stomatal movement | 2 (0.16%) | 6 (0.18%) | 0.712757 | 0.999992 |
| 605 | [GO:0019751](file:///E:\2018-7-3%E7%81%AB%E9%BE%99%E6%9E%9C%E8%BD%AC%E5%BD%95%E7%BB%84%E6%B5%8B%E5%BA%8F\%E5%AE%8C%E6%95%B4%E7%89%88%E6%95%B0%E6%8D%AE\GDR3855-Hylocereus_undulatus_Britt-12-RNAseq_result\4_Function\2_Group_Diff_Function\UP_DOWN\GO\NL-VS-L1.P.html#gene605) | polyol metabolic process | 2 (0.16%) | 6 (0.18%) | 0.712757 | 0.999992 |
| 606 | [GO:0051301](file:///E:\2018-7-3%E7%81%AB%E9%BE%99%E6%9E%9C%E8%BD%AC%E5%BD%95%E7%BB%84%E6%B5%8B%E5%BA%8F\%E5%AE%8C%E6%95%B4%E7%89%88%E6%95%B0%E6%8D%AE\GDR3855-Hylocereus_undulatus_Britt-12-RNAseq_result\4_Function\2_Group_Diff_Function\UP_DOWN\GO\NL-VS-L1.P.html#gene606) | cell division | 2 (0.16%) | 6 (0.18%) | 0.712757 | 0.999992 |
| 607 | [GO:0071229](file:///E:\2018-7-3%E7%81%AB%E9%BE%99%E6%9E%9C%E8%BD%AC%E5%BD%95%E7%BB%84%E6%B5%8B%E5%BA%8F\%E5%AE%8C%E6%95%B4%E7%89%88%E6%95%B0%E6%8D%AE\GDR3855-Hylocereus_undulatus_Britt-12-RNAseq_result\4_Function\2_Group_Diff_Function\UP_DOWN\GO\NL-VS-L1.P.html#gene607) | cellular response to acid chemical | 2 (0.16%) | 6 (0.18%) | 0.712757 | 0.999992 |
| 608 | [GO:0022622](file:///E:\2018-7-3%E7%81%AB%E9%BE%99%E6%9E%9C%E8%BD%AC%E5%BD%95%E7%BB%84%E6%B5%8B%E5%BA%8F\%E5%AE%8C%E6%95%B4%E7%89%88%E6%95%B0%E6%8D%AE\GDR3855-Hylocereus_undulatus_Britt-12-RNAseq_result\4_Function\2_Group_Diff_Function\UP_DOWN\GO\NL-VS-L1.P.html#gene608) | root system development | 10 (0.81%) | 30 (0.89%) | 0.714373 | 0.999992 |
| 609 | [GO:0030154](file:///E:\2018-7-3%E7%81%AB%E9%BE%99%E6%9E%9C%E8%BD%AC%E5%BD%95%E7%BB%84%E6%B5%8B%E5%BA%8F\%E5%AE%8C%E6%95%B4%E7%89%88%E6%95%B0%E6%8D%AE\GDR3855-Hylocereus_undulatus_Britt-12-RNAseq_result\4_Function\2_Group_Diff_Function\UP_DOWN\GO\NL-VS-L1.P.html#gene609) | cell differentiation | 11 (0.89%) | 33 (0.98%) | 0.718184 | 0.999992 |
| 610 | [GO:0001934](file:///E:\2018-7-3%E7%81%AB%E9%BE%99%E6%9E%9C%E8%BD%AC%E5%BD%95%E7%BB%84%E6%B5%8B%E5%BA%8F\%E5%AE%8C%E6%95%B4%E7%89%88%E6%95%B0%E6%8D%AE\GDR3855-Hylocereus_undulatus_Britt-12-RNAseq_result\4_Function\2_Group_Diff_Function\UP_DOWN\GO\NL-VS-L1.P.html#gene610) | positive regulation of protein phosphorylation | 12 (0.97%) | 36 (1.07%) | 0.722036 | 0.999992 |
| 611 | [GO:0010562](file:///E:\2018-7-3%E7%81%AB%E9%BE%99%E6%9E%9C%E8%BD%AC%E5%BD%95%E7%BB%84%E6%B5%8B%E5%BA%8F\%E5%AE%8C%E6%95%B4%E7%89%88%E6%95%B0%E6%8D%AE\GDR3855-Hylocereus_undulatus_Britt-12-RNAseq_result\4_Function\2_Group_Diff_Function\UP_DOWN\GO\NL-VS-L1.P.html#gene611) | positive regulation of phosphorus metabolic process | 12 (0.97%) | 36 (1.07%) | 0.722036 | 0.999992 |
| 612 | [GO:0031325](file:///E:\2018-7-3%E7%81%AB%E9%BE%99%E6%9E%9C%E8%BD%AC%E5%BD%95%E7%BB%84%E6%B5%8B%E5%BA%8F\%E5%AE%8C%E6%95%B4%E7%89%88%E6%95%B0%E6%8D%AE\GDR3855-Hylocereus_undulatus_Britt-12-RNAseq_result\4_Function\2_Group_Diff_Function\UP_DOWN\GO\NL-VS-L1.P.html#gene612) | positive regulation of cellular metabolic process | 12 (0.97%) | 36 (1.07%) | 0.722036 | 0.999992 |
| 613 | [GO:0031401](file:///E:\2018-7-3%E7%81%AB%E9%BE%99%E6%9E%9C%E8%BD%AC%E5%BD%95%E7%BB%84%E6%B5%8B%E5%BA%8F\%E5%AE%8C%E6%95%B4%E7%89%88%E6%95%B0%E6%8D%AE\GDR3855-Hylocereus_undulatus_Britt-12-RNAseq_result\4_Function\2_Group_Diff_Function\UP_DOWN\GO\NL-VS-L1.P.html#gene613) | positive regulation of protein modification process | 12 (0.97%) | 36 (1.07%) | 0.722036 | 0.999992 |
| 614 | [GO:0032147](file:///E:\2018-7-3%E7%81%AB%E9%BE%99%E6%9E%9C%E8%BD%AC%E5%BD%95%E7%BB%84%E6%B5%8B%E5%BA%8F\%E5%AE%8C%E6%95%B4%E7%89%88%E6%95%B0%E6%8D%AE\GDR3855-Hylocereus_undulatus_Britt-12-RNAseq_result\4_Function\2_Group_Diff_Function\UP_DOWN\GO\NL-VS-L1.P.html#gene614) | activation of protein kinase activity | 12 (0.97%) | 36 (1.07%) | 0.722036 | 0.999992 |
| 615 | [GO:0032270](file:///E:\2018-7-3%E7%81%AB%E9%BE%99%E6%9E%9C%E8%BD%AC%E5%BD%95%E7%BB%84%E6%B5%8B%E5%BA%8F\%E5%AE%8C%E6%95%B4%E7%89%88%E6%95%B0%E6%8D%AE\GDR3855-Hylocereus_undulatus_Britt-12-RNAseq_result\4_Function\2_Group_Diff_Function\UP_DOWN\GO\NL-VS-L1.P.html#gene615) | positive regulation of cellular protein metabolic process | 12 (0.97%) | 36 (1.07%) | 0.722036 | 0.999992 |
| 616 | [GO:0033674](file:///E:\2018-7-3%E7%81%AB%E9%BE%99%E6%9E%9C%E8%BD%AC%E5%BD%95%E7%BB%84%E6%B5%8B%E5%BA%8F\%E5%AE%8C%E6%95%B4%E7%89%88%E6%95%B0%E6%8D%AE\GDR3855-Hylocereus_undulatus_Britt-12-RNAseq_result\4_Function\2_Group_Diff_Function\UP_DOWN\GO\NL-VS-L1.P.html#gene616) | positive regulation of kinase activity | 12 (0.97%) | 36 (1.07%) | 0.722036 | 0.999992 |
| 617 | [GO:0042327](file:///E:\2018-7-3%E7%81%AB%E9%BE%99%E6%9E%9C%E8%BD%AC%E5%BD%95%E7%BB%84%E6%B5%8B%E5%BA%8F\%E5%AE%8C%E6%95%B4%E7%89%88%E6%95%B0%E6%8D%AE\GDR3855-Hylocereus_undulatus_Britt-12-RNAseq_result\4_Function\2_Group_Diff_Function\UP_DOWN\GO\NL-VS-L1.P.html#gene617) | positive regulation of phosphorylation | 12 (0.97%) | 36 (1.07%) | 0.722036 | 0.999992 |
| 618 | [GO:0043549](file:///E:\2018-7-3%E7%81%AB%E9%BE%99%E6%9E%9C%E8%BD%AC%E5%BD%95%E7%BB%84%E6%B5%8B%E5%BA%8F\%E5%AE%8C%E6%95%B4%E7%89%88%E6%95%B0%E6%8D%AE\GDR3855-Hylocereus_undulatus_Britt-12-RNAseq_result\4_Function\2_Group_Diff_Function\UP_DOWN\GO\NL-VS-L1.P.html#gene618) | regulation of kinase activity | 12 (0.97%) | 36 (1.07%) | 0.722036 | 0.999992 |
| 619 | [GO:0045859](file:///E:\2018-7-3%E7%81%AB%E9%BE%99%E6%9E%9C%E8%BD%AC%E5%BD%95%E7%BB%84%E6%B5%8B%E5%BA%8F\%E5%AE%8C%E6%95%B4%E7%89%88%E6%95%B0%E6%8D%AE\GDR3855-Hylocereus_undulatus_Britt-12-RNAseq_result\4_Function\2_Group_Diff_Function\UP_DOWN\GO\NL-VS-L1.P.html#gene619) | regulation of protein kinase activity | 12 (0.97%) | 36 (1.07%) | 0.722036 | 0.999992 |
| 620 | [GO:0045860](file:///E:\2018-7-3%E7%81%AB%E9%BE%99%E6%9E%9C%E8%BD%AC%E5%BD%95%E7%BB%84%E6%B5%8B%E5%BA%8F\%E5%AE%8C%E6%95%B4%E7%89%88%E6%95%B0%E6%8D%AE\GDR3855-Hylocereus_undulatus_Britt-12-RNAseq_result\4_Function\2_Group_Diff_Function\UP_DOWN\GO\NL-VS-L1.P.html#gene620) | positive regulation of protein kinase activity | 12 (0.97%) | 36 (1.07%) | 0.722036 | 0.999992 |
| 621 | [GO:0045937](file:///E:\2018-7-3%E7%81%AB%E9%BE%99%E6%9E%9C%E8%BD%AC%E5%BD%95%E7%BB%84%E6%B5%8B%E5%BA%8F\%E5%AE%8C%E6%95%B4%E7%89%88%E6%95%B0%E6%8D%AE\GDR3855-Hylocereus_undulatus_Britt-12-RNAseq_result\4_Function\2_Group_Diff_Function\UP_DOWN\GO\NL-VS-L1.P.html#gene621) | positive regulation of phosphate metabolic process | 12 (0.97%) | 36 (1.07%) | 0.722036 | 0.999992 |
| 622 | [GO:0051247](file:///E:\2018-7-3%E7%81%AB%E9%BE%99%E6%9E%9C%E8%BD%AC%E5%BD%95%E7%BB%84%E6%B5%8B%E5%BA%8F\%E5%AE%8C%E6%95%B4%E7%89%88%E6%95%B0%E6%8D%AE\GDR3855-Hylocereus_undulatus_Britt-12-RNAseq_result\4_Function\2_Group_Diff_Function\UP_DOWN\GO\NL-VS-L1.P.html#gene622) | positive regulation of protein metabolic process | 12 (0.97%) | 36 (1.07%) | 0.722036 | 0.999992 |
| 623 | [GO:0051338](file:///E:\2018-7-3%E7%81%AB%E9%BE%99%E6%9E%9C%E8%BD%AC%E5%BD%95%E7%BB%84%E6%B5%8B%E5%BA%8F\%E5%AE%8C%E6%95%B4%E7%89%88%E6%95%B0%E6%8D%AE\GDR3855-Hylocereus_undulatus_Britt-12-RNAseq_result\4_Function\2_Group_Diff_Function\UP_DOWN\GO\NL-VS-L1.P.html#gene623) | regulation of transferase activity | 12 (0.97%) | 36 (1.07%) | 0.722036 | 0.999992 |
| 624 | [GO:0051347](file:///E:\2018-7-3%E7%81%AB%E9%BE%99%E6%9E%9C%E8%BD%AC%E5%BD%95%E7%BB%84%E6%B5%8B%E5%BA%8F\%E5%AE%8C%E6%95%B4%E7%89%88%E6%95%B0%E6%8D%AE\GDR3855-Hylocereus_undulatus_Britt-12-RNAseq_result\4_Function\2_Group_Diff_Function\UP_DOWN\GO\NL-VS-L1.P.html#gene624) | positive regulation of transferase activity | 12 (0.97%) | 36 (1.07%) | 0.722036 | 0.999992 |
| 625 | [GO:0009893](file:///E:\2018-7-3%E7%81%AB%E9%BE%99%E6%9E%9C%E8%BD%AC%E5%BD%95%E7%BB%84%E6%B5%8B%E5%BA%8F\%E5%AE%8C%E6%95%B4%E7%89%88%E6%95%B0%E6%8D%AE\GDR3855-Hylocereus_undulatus_Britt-12-RNAseq_result\4_Function\2_Group_Diff_Function\UP_DOWN\GO\NL-VS-L1.P.html#gene625) | positive regulation of metabolic process | 13 (1.05%) | 39 (1.16%) | 0.725894 | 0.999992 |
| 626 | [GO:0060255](file:///E:\2018-7-3%E7%81%AB%E9%BE%99%E6%9E%9C%E8%BD%AC%E5%BD%95%E7%BB%84%E6%B5%8B%E5%BA%8F\%E5%AE%8C%E6%95%B4%E7%89%88%E6%95%B0%E6%8D%AE\GDR3855-Hylocereus_undulatus_Britt-12-RNAseq_result\4_Function\2_Group_Diff_Function\UP_DOWN\GO\NL-VS-L1.P.html#gene626) | regulation of macromolecule metabolic process | 22 (1.78%) | 65 (1.94%) | 0.729306 | 0.999992 |
| 627 | [GO:0016485](file:///E:\2018-7-3%E7%81%AB%E9%BE%99%E6%9E%9C%E8%BD%AC%E5%BD%95%E7%BB%84%E6%B5%8B%E5%BA%8F\%E5%AE%8C%E6%95%B4%E7%89%88%E6%95%B0%E6%8D%AE\GDR3855-Hylocereus_undulatus_Britt-12-RNAseq_result\4_Function\2_Group_Diff_Function\UP_DOWN\GO\NL-VS-L1.P.html#gene627) | protein processing | 29 (2.35%) | 85 (2.53%) | 0.730741 | 0.999992 |
| 628 | [GO:0005996](file:///E:\2018-7-3%E7%81%AB%E9%BE%99%E6%9E%9C%E8%BD%AC%E5%BD%95%E7%BB%84%E6%B5%8B%E5%BA%8F\%E5%AE%8C%E6%95%B4%E7%89%88%E6%95%B0%E6%8D%AE\GDR3855-Hylocereus_undulatus_Britt-12-RNAseq_result\4_Function\2_Group_Diff_Function\UP_DOWN\GO\NL-VS-L1.P.html#gene628) | monosaccharide metabolic process | 15 (1.22%) | 45 (1.34%) | 0.733543 | 0.999992 |
| 629 | [GO:0008104](file:///E:\2018-7-3%E7%81%AB%E9%BE%99%E6%9E%9C%E8%BD%AC%E5%BD%95%E7%BB%84%E6%B5%8B%E5%BA%8F\%E5%AE%8C%E6%95%B4%E7%89%88%E6%95%B0%E6%8D%AE\GDR3855-Hylocereus_undulatus_Britt-12-RNAseq_result\4_Function\2_Group_Diff_Function\UP_DOWN\GO\NL-VS-L1.P.html#gene629) | protein localization | 43 (3.48%) | 125 (3.72%) | 0.739615 | 0.999992 |
| 630 | [GO:0010033](file:///E:\2018-7-3%E7%81%AB%E9%BE%99%E6%9E%9C%E8%BD%AC%E5%BD%95%E7%BB%84%E6%B5%8B%E5%BA%8F\%E5%AE%8C%E6%95%B4%E7%89%88%E6%95%B0%E6%8D%AE\GDR3855-Hylocereus_undulatus_Britt-12-RNAseq_result\4_Function\2_Group_Diff_Function\UP_DOWN\GO\NL-VS-L1.P.html#gene630) | response to organic substance | 38 (3.08%) | 111 (3.3%) | 0.742590 | 0.999992 |
| 631 | [GO:0000154](file:///E:\2018-7-3%E7%81%AB%E9%BE%99%E6%9E%9C%E8%BD%AC%E5%BD%95%E7%BB%84%E6%B5%8B%E5%BA%8F\%E5%AE%8C%E6%95%B4%E7%89%88%E6%95%B0%E6%8D%AE\GDR3855-Hylocereus_undulatus_Britt-12-RNAseq_result\4_Function\2_Group_Diff_Function\UP_DOWN\GO\NL-VS-L1.P.html#gene631) | rRNA modification | 1 (0.08%) | 3 (0.09%) | 0.746941 | 0.999992 |
| 632 | [GO:0000723](file:///E:\2018-7-3%E7%81%AB%E9%BE%99%E6%9E%9C%E8%BD%AC%E5%BD%95%E7%BB%84%E6%B5%8B%E5%BA%8F\%E5%AE%8C%E6%95%B4%E7%89%88%E6%95%B0%E6%8D%AE\GDR3855-Hylocereus_undulatus_Britt-12-RNAseq_result\4_Function\2_Group_Diff_Function\UP_DOWN\GO\NL-VS-L1.P.html#gene632) | telomere maintenance | 1 (0.08%) | 3 (0.09%) | 0.746941 | 0.999992 |
| 633 | [GO:0000725](file:///E:\2018-7-3%E7%81%AB%E9%BE%99%E6%9E%9C%E8%BD%AC%E5%BD%95%E7%BB%84%E6%B5%8B%E5%BA%8F\%E5%AE%8C%E6%95%B4%E7%89%88%E6%95%B0%E6%8D%AE\GDR3855-Hylocereus_undulatus_Britt-12-RNAseq_result\4_Function\2_Group_Diff_Function\UP_DOWN\GO\NL-VS-L1.P.html#gene633) | recombinational repair | 1 (0.08%) | 3 (0.09%) | 0.746941 | 0.999992 |
| 634 | [GO:0000819](file:///E:\2018-7-3%E7%81%AB%E9%BE%99%E6%9E%9C%E8%BD%AC%E5%BD%95%E7%BB%84%E6%B5%8B%E5%BA%8F\%E5%AE%8C%E6%95%B4%E7%89%88%E6%95%B0%E6%8D%AE\GDR3855-Hylocereus_undulatus_Britt-12-RNAseq_result\4_Function\2_Group_Diff_Function\UP_DOWN\GO\NL-VS-L1.P.html#gene634) | sister chromatid segregation | 1 (0.08%) | 3 (0.09%) | 0.746941 | 0.999992 |
| 635 | [GO:0000919](file:///E:\2018-7-3%E7%81%AB%E9%BE%99%E6%9E%9C%E8%BD%AC%E5%BD%95%E7%BB%84%E6%B5%8B%E5%BA%8F\%E5%AE%8C%E6%95%B4%E7%89%88%E6%95%B0%E6%8D%AE\GDR3855-Hylocereus_undulatus_Britt-12-RNAseq_result\4_Function\2_Group_Diff_Function\UP_DOWN\GO\NL-VS-L1.P.html#gene635) | cell plate assembly | 1 (0.08%) | 3 (0.09%) | 0.746941 | 0.999992 |
| 636 | [GO:0001558](file:///E:\2018-7-3%E7%81%AB%E9%BE%99%E6%9E%9C%E8%BD%AC%E5%BD%95%E7%BB%84%E6%B5%8B%E5%BA%8F\%E5%AE%8C%E6%95%B4%E7%89%88%E6%95%B0%E6%8D%AE\GDR3855-Hylocereus_undulatus_Britt-12-RNAseq_result\4_Function\2_Group_Diff_Function\UP_DOWN\GO\NL-VS-L1.P.html#gene636) | regulation of cell growth | 1 (0.08%) | 3 (0.09%) | 0.746941 | 0.999992 |
| 637 | [GO:0006022](file:///E:\2018-7-3%E7%81%AB%E9%BE%99%E6%9E%9C%E8%BD%AC%E5%BD%95%E7%BB%84%E6%B5%8B%E5%BA%8F\%E5%AE%8C%E6%95%B4%E7%89%88%E6%95%B0%E6%8D%AE\GDR3855-Hylocereus_undulatus_Britt-12-RNAseq_result\4_Function\2_Group_Diff_Function\UP_DOWN\GO\NL-VS-L1.P.html#gene637) | aminoglycan metabolic process | 1 (0.08%) | 3 (0.09%) | 0.746941 | 0.999992 |
| 638 | [GO:0006098](file:///E:\2018-7-3%E7%81%AB%E9%BE%99%E6%9E%9C%E8%BD%AC%E5%BD%95%E7%BB%84%E6%B5%8B%E5%BA%8F\%E5%AE%8C%E6%95%B4%E7%89%88%E6%95%B0%E6%8D%AE\GDR3855-Hylocereus_undulatus_Britt-12-RNAseq_result\4_Function\2_Group_Diff_Function\UP_DOWN\GO\NL-VS-L1.P.html#gene638) | pentose-phosphate shunt | 1 (0.08%) | 3 (0.09%) | 0.746941 | 0.999992 |
| 639 | [GO:0006302](file:///E:\2018-7-3%E7%81%AB%E9%BE%99%E6%9E%9C%E8%BD%AC%E5%BD%95%E7%BB%84%E6%B5%8B%E5%BA%8F\%E5%AE%8C%E6%95%B4%E7%89%88%E6%95%B0%E6%8D%AE\GDR3855-Hylocereus_undulatus_Britt-12-RNAseq_result\4_Function\2_Group_Diff_Function\UP_DOWN\GO\NL-VS-L1.P.html#gene639) | double-strand break repair | 1 (0.08%) | 3 (0.09%) | 0.746941 | 0.999992 |
| 640 | [GO:0006364](file:///E:\2018-7-3%E7%81%AB%E9%BE%99%E6%9E%9C%E8%BD%AC%E5%BD%95%E7%BB%84%E6%B5%8B%E5%BA%8F\%E5%AE%8C%E6%95%B4%E7%89%88%E6%95%B0%E6%8D%AE\GDR3855-Hylocereus_undulatus_Britt-12-RNAseq_result\4_Function\2_Group_Diff_Function\UP_DOWN\GO\NL-VS-L1.P.html#gene640) | rRNA processing | 1 (0.08%) | 3 (0.09%) | 0.746941 | 0.999992 |
| 641 | [GO:0006401](file:///E:\2018-7-3%E7%81%AB%E9%BE%99%E6%9E%9C%E8%BD%AC%E5%BD%95%E7%BB%84%E6%B5%8B%E5%BA%8F\%E5%AE%8C%E6%95%B4%E7%89%88%E6%95%B0%E6%8D%AE\GDR3855-Hylocereus_undulatus_Britt-12-RNAseq_result\4_Function\2_Group_Diff_Function\UP_DOWN\GO\NL-VS-L1.P.html#gene641) | RNA catabolic process | 1 (0.08%) | 3 (0.09%) | 0.746941 | 0.999992 |
| 642 | [GO:0006417](file:///E:\2018-7-3%E7%81%AB%E9%BE%99%E6%9E%9C%E8%BD%AC%E5%BD%95%E7%BB%84%E6%B5%8B%E5%BA%8F\%E5%AE%8C%E6%95%B4%E7%89%88%E6%95%B0%E6%8D%AE\GDR3855-Hylocereus_undulatus_Britt-12-RNAseq_result\4_Function\2_Group_Diff_Function\UP_DOWN\GO\NL-VS-L1.P.html#gene642) | regulation of translation | 1 (0.08%) | 3 (0.09%) | 0.746941 | 0.999992 |
| 643 | [GO:0006497](file:///E:\2018-7-3%E7%81%AB%E9%BE%99%E6%9E%9C%E8%BD%AC%E5%BD%95%E7%BB%84%E6%B5%8B%E5%BA%8F\%E5%AE%8C%E6%95%B4%E7%89%88%E6%95%B0%E6%8D%AE\GDR3855-Hylocereus_undulatus_Britt-12-RNAseq_result\4_Function\2_Group_Diff_Function\UP_DOWN\GO\NL-VS-L1.P.html#gene643) | protein lipidation | 1 (0.08%) | 3 (0.09%) | 0.746941 | 0.999992 |
| 644 | [GO:0006525](file:///E:\2018-7-3%E7%81%AB%E9%BE%99%E6%9E%9C%E8%BD%AC%E5%BD%95%E7%BB%84%E6%B5%8B%E5%BA%8F\%E5%AE%8C%E6%95%B4%E7%89%88%E6%95%B0%E6%8D%AE\GDR3855-Hylocereus_undulatus_Britt-12-RNAseq_result\4_Function\2_Group_Diff_Function\UP_DOWN\GO\NL-VS-L1.P.html#gene644) | arginine metabolic process | 1 (0.08%) | 3 (0.09%) | 0.746941 | 0.999992 |
| 645 | [GO:0006553](file:///E:\2018-7-3%E7%81%AB%E9%BE%99%E6%9E%9C%E8%BD%AC%E5%BD%95%E7%BB%84%E6%B5%8B%E5%BA%8F\%E5%AE%8C%E6%95%B4%E7%89%88%E6%95%B0%E6%8D%AE\GDR3855-Hylocereus_undulatus_Britt-12-RNAseq_result\4_Function\2_Group_Diff_Function\UP_DOWN\GO\NL-VS-L1.P.html#gene645) | lysine metabolic process | 1 (0.08%) | 3 (0.09%) | 0.746941 | 0.999992 |
| 646 | [GO:0006555](file:///E:\2018-7-3%E7%81%AB%E9%BE%99%E6%9E%9C%E8%BD%AC%E5%BD%95%E7%BB%84%E6%B5%8B%E5%BA%8F\%E5%AE%8C%E6%95%B4%E7%89%88%E6%95%B0%E6%8D%AE\GDR3855-Hylocereus_undulatus_Britt-12-RNAseq_result\4_Function\2_Group_Diff_Function\UP_DOWN\GO\NL-VS-L1.P.html#gene646) | methionine metabolic process | 1 (0.08%) | 3 (0.09%) | 0.746941 | 0.999992 |
| 647 | [GO:0006771](file:///E:\2018-7-3%E7%81%AB%E9%BE%99%E6%9E%9C%E8%BD%AC%E5%BD%95%E7%BB%84%E6%B5%8B%E5%BA%8F\%E5%AE%8C%E6%95%B4%E7%89%88%E6%95%B0%E6%8D%AE\GDR3855-Hylocereus_undulatus_Britt-12-RNAseq_result\4_Function\2_Group_Diff_Function\UP_DOWN\GO\NL-VS-L1.P.html#gene647) | riboflavin metabolic process | 1 (0.08%) | 3 (0.09%) | 0.746941 | 0.999992 |
| 648 | [GO:0006857](file:///E:\2018-7-3%E7%81%AB%E9%BE%99%E6%9E%9C%E8%BD%AC%E5%BD%95%E7%BB%84%E6%B5%8B%E5%BA%8F\%E5%AE%8C%E6%95%B4%E7%89%88%E6%95%B0%E6%8D%AE\GDR3855-Hylocereus_undulatus_Britt-12-RNAseq_result\4_Function\2_Group_Diff_Function\UP_DOWN\GO\NL-VS-L1.P.html#gene648) | oligopeptide transport | 1 (0.08%) | 3 (0.09%) | 0.746941 | 0.999992 |
| 649 | [GO:0006869](file:///E:\2018-7-3%E7%81%AB%E9%BE%99%E6%9E%9C%E8%BD%AC%E5%BD%95%E7%BB%84%E6%B5%8B%E5%BA%8F\%E5%AE%8C%E6%95%B4%E7%89%88%E6%95%B0%E6%8D%AE\GDR3855-Hylocereus_undulatus_Britt-12-RNAseq_result\4_Function\2_Group_Diff_Function\UP_DOWN\GO\NL-VS-L1.P.html#gene649) | lipid transport | 1 (0.08%) | 3 (0.09%) | 0.746941 | 0.999992 |
| 650 | [GO:0009085](file:///E:\2018-7-3%E7%81%AB%E9%BE%99%E6%9E%9C%E8%BD%AC%E5%BD%95%E7%BB%84%E6%B5%8B%E5%BA%8F\%E5%AE%8C%E6%95%B4%E7%89%88%E6%95%B0%E6%8D%AE\GDR3855-Hylocereus_undulatus_Britt-12-RNAseq_result\4_Function\2_Group_Diff_Function\UP_DOWN\GO\NL-VS-L1.P.html#gene650) | lysine biosynthetic process | 1 (0.08%) | 3 (0.09%) | 0.746941 | 0.999992 |
| 651 | [GO:0009086](file:///E:\2018-7-3%E7%81%AB%E9%BE%99%E6%9E%9C%E8%BD%AC%E5%BD%95%E7%BB%84%E6%B5%8B%E5%BA%8F\%E5%AE%8C%E6%95%B4%E7%89%88%E6%95%B0%E6%8D%AE\GDR3855-Hylocereus_undulatus_Britt-12-RNAseq_result\4_Function\2_Group_Diff_Function\UP_DOWN\GO\NL-VS-L1.P.html#gene651) | methionine biosynthetic process | 1 (0.08%) | 3 (0.09%) | 0.746941 | 0.999992 |
| 652 | [GO:0009608](file:///E:\2018-7-3%E7%81%AB%E9%BE%99%E6%9E%9C%E8%BD%AC%E5%BD%95%E7%BB%84%E6%B5%8B%E5%BA%8F\%E5%AE%8C%E6%95%B4%E7%89%88%E6%95%B0%E6%8D%AE\GDR3855-Hylocereus_undulatus_Britt-12-RNAseq_result\4_Function\2_Group_Diff_Function\UP_DOWN\GO\NL-VS-L1.P.html#gene652) | response to symbiont | 1 (0.08%) | 3 (0.09%) | 0.746941 | 0.999992 |
| 653 | [GO:0015669](file:///E:\2018-7-3%E7%81%AB%E9%BE%99%E6%9E%9C%E8%BD%AC%E5%BD%95%E7%BB%84%E6%B5%8B%E5%BA%8F\%E5%AE%8C%E6%95%B4%E7%89%88%E6%95%B0%E6%8D%AE\GDR3855-Hylocereus_undulatus_Britt-12-RNAseq_result\4_Function\2_Group_Diff_Function\UP_DOWN\GO\NL-VS-L1.P.html#gene653) | gas transport | 1 (0.08%) | 3 (0.09%) | 0.746941 | 0.999992 |
| 654 | [GO:0015766](file:///E:\2018-7-3%E7%81%AB%E9%BE%99%E6%9E%9C%E8%BD%AC%E5%BD%95%E7%BB%84%E6%B5%8B%E5%BA%8F\%E5%AE%8C%E6%95%B4%E7%89%88%E6%95%B0%E6%8D%AE\GDR3855-Hylocereus_undulatus_Britt-12-RNAseq_result\4_Function\2_Group_Diff_Function\UP_DOWN\GO\NL-VS-L1.P.html#gene654) | disaccharide transport | 1 (0.08%) | 3 (0.09%) | 0.746941 | 0.999992 |
| 655 | [GO:0015772](file:///E:\2018-7-3%E7%81%AB%E9%BE%99%E6%9E%9C%E8%BD%AC%E5%BD%95%E7%BB%84%E6%B5%8B%E5%BA%8F\%E5%AE%8C%E6%95%B4%E7%89%88%E6%95%B0%E6%8D%AE\GDR3855-Hylocereus_undulatus_Britt-12-RNAseq_result\4_Function\2_Group_Diff_Function\UP_DOWN\GO\NL-VS-L1.P.html#gene655) | oligosaccharide transport | 1 (0.08%) | 3 (0.09%) | 0.746941 | 0.999992 |
| 656 | [GO:0015985](file:///E:\2018-7-3%E7%81%AB%E9%BE%99%E6%9E%9C%E8%BD%AC%E5%BD%95%E7%BB%84%E6%B5%8B%E5%BA%8F\%E5%AE%8C%E6%95%B4%E7%89%88%E6%95%B0%E6%8D%AE\GDR3855-Hylocereus_undulatus_Britt-12-RNAseq_result\4_Function\2_Group_Diff_Function\UP_DOWN\GO\NL-VS-L1.P.html#gene656) | energy coupled proton transport, down electrochemical gradient | 1 (0.08%) | 3 (0.09%) | 0.746941 | 0.999992 |
| 657 | [GO:0015986](file:///E:\2018-7-3%E7%81%AB%E9%BE%99%E6%9E%9C%E8%BD%AC%E5%BD%95%E7%BB%84%E6%B5%8B%E5%BA%8F\%E5%AE%8C%E6%95%B4%E7%89%88%E6%95%B0%E6%8D%AE\GDR3855-Hylocereus_undulatus_Britt-12-RNAseq_result\4_Function\2_Group_Diff_Function\UP_DOWN\GO\NL-VS-L1.P.html#gene657) | ATP synthesis coupled proton transport | 1 (0.08%) | 3 (0.09%) | 0.746941 | 0.999992 |
| 658 | [GO:0016119](file:///E:\2018-7-3%E7%81%AB%E9%BE%99%E6%9E%9C%E8%BD%AC%E5%BD%95%E7%BB%84%E6%B5%8B%E5%BA%8F\%E5%AE%8C%E6%95%B4%E7%89%88%E6%95%B0%E6%8D%AE\GDR3855-Hylocereus_undulatus_Britt-12-RNAseq_result\4_Function\2_Group_Diff_Function\UP_DOWN\GO\NL-VS-L1.P.html#gene658) | carotene metabolic process | 1 (0.08%) | 3 (0.09%) | 0.746941 | 0.999992 |
| 659 | [GO:0019682](file:///E:\2018-7-3%E7%81%AB%E9%BE%99%E6%9E%9C%E8%BD%AC%E5%BD%95%E7%BB%84%E6%B5%8B%E5%BA%8F\%E5%AE%8C%E6%95%B4%E7%89%88%E6%95%B0%E6%8D%AE\GDR3855-Hylocereus_undulatus_Britt-12-RNAseq_result\4_Function\2_Group_Diff_Function\UP_DOWN\GO\NL-VS-L1.P.html#gene659) | glyceraldehyde-3-phosphate metabolic process | 1 (0.08%) | 3 (0.09%) | 0.746941 | 0.999992 |
| 660 | [GO:0019932](file:///E:\2018-7-3%E7%81%AB%E9%BE%99%E6%9E%9C%E8%BD%AC%E5%BD%95%E7%BB%84%E6%B5%8B%E5%BA%8F\%E5%AE%8C%E6%95%B4%E7%89%88%E6%95%B0%E6%8D%AE\GDR3855-Hylocereus_undulatus_Britt-12-RNAseq_result\4_Function\2_Group_Diff_Function\UP_DOWN\GO\NL-VS-L1.P.html#gene660) | second-messenger-mediated signaling | 1 (0.08%) | 3 (0.09%) | 0.746941 | 0.999992 |
| 661 | [GO:0019953](file:///E:\2018-7-3%E7%81%AB%E9%BE%99%E6%9E%9C%E8%BD%AC%E5%BD%95%E7%BB%84%E6%B5%8B%E5%BA%8F\%E5%AE%8C%E6%95%B4%E7%89%88%E6%95%B0%E6%8D%AE\GDR3855-Hylocereus_undulatus_Britt-12-RNAseq_result\4_Function\2_Group_Diff_Function\UP_DOWN\GO\NL-VS-L1.P.html#gene661) | sexual reproduction | 1 (0.08%) | 3 (0.09%) | 0.746941 | 0.999992 |
| 662 | [GO:0022610](file:///E:\2018-7-3%E7%81%AB%E9%BE%99%E6%9E%9C%E8%BD%AC%E5%BD%95%E7%BB%84%E6%B5%8B%E5%BA%8F\%E5%AE%8C%E6%95%B4%E7%89%88%E6%95%B0%E6%8D%AE\GDR3855-Hylocereus_undulatus_Britt-12-RNAseq_result\4_Function\2_Group_Diff_Function\UP_DOWN\GO\NL-VS-L1.P.html#gene662) | biological adhesion | 1 (0.08%) | 3 (0.09%) | 0.746941 | 0.999992 |
| 663 | [GO:0030002](file:///E:\2018-7-3%E7%81%AB%E9%BE%99%E6%9E%9C%E8%BD%AC%E5%BD%95%E7%BB%84%E6%B5%8B%E5%BA%8F\%E5%AE%8C%E6%95%B4%E7%89%88%E6%95%B0%E6%8D%AE\GDR3855-Hylocereus_undulatus_Britt-12-RNAseq_result\4_Function\2_Group_Diff_Function\UP_DOWN\GO\NL-VS-L1.P.html#gene663) | cellular anion homeostasis | 1 (0.08%) | 3 (0.09%) | 0.746941 | 0.999992 |
| 664 | [GO:0032200](file:///E:\2018-7-3%E7%81%AB%E9%BE%99%E6%9E%9C%E8%BD%AC%E5%BD%95%E7%BB%84%E6%B5%8B%E5%BA%8F\%E5%AE%8C%E6%95%B4%E7%89%88%E6%95%B0%E6%8D%AE\GDR3855-Hylocereus_undulatus_Britt-12-RNAseq_result\4_Function\2_Group_Diff_Function\UP_DOWN\GO\NL-VS-L1.P.html#gene664) | telomere organization | 1 (0.08%) | 3 (0.09%) | 0.746941 | 0.999992 |
| 665 | [GO:0032958](file:///E:\2018-7-3%E7%81%AB%E9%BE%99%E6%9E%9C%E8%BD%AC%E5%BD%95%E7%BB%84%E6%B5%8B%E5%BA%8F\%E5%AE%8C%E6%95%B4%E7%89%88%E6%95%B0%E6%8D%AE\GDR3855-Hylocereus_undulatus_Britt-12-RNAseq_result\4_Function\2_Group_Diff_Function\UP_DOWN\GO\NL-VS-L1.P.html#gene665) | inositol phosphate biosynthetic process | 1 (0.08%) | 3 (0.09%) | 0.746941 | 0.999992 |
| 666 | [GO:0034248](file:///E:\2018-7-3%E7%81%AB%E9%BE%99%E6%9E%9C%E8%BD%AC%E5%BD%95%E7%BB%84%E6%B5%8B%E5%BA%8F\%E5%AE%8C%E6%95%B4%E7%89%88%E6%95%B0%E6%8D%AE\GDR3855-Hylocereus_undulatus_Britt-12-RNAseq_result\4_Function\2_Group_Diff_Function\UP_DOWN\GO\NL-VS-L1.P.html#gene666) | regulation of cellular amide metabolic process | 1 (0.08%) | 3 (0.09%) | 0.746941 | 0.999992 |
| 667 | [GO:0040008](file:///E:\2018-7-3%E7%81%AB%E9%BE%99%E6%9E%9C%E8%BD%AC%E5%BD%95%E7%BB%84%E6%B5%8B%E5%BA%8F\%E5%AE%8C%E6%95%B4%E7%89%88%E6%95%B0%E6%8D%AE\GDR3855-Hylocereus_undulatus_Britt-12-RNAseq_result\4_Function\2_Group_Diff_Function\UP_DOWN\GO\NL-VS-L1.P.html#gene667) | regulation of growth | 1 (0.08%) | 3 (0.09%) | 0.746941 | 0.999992 |
| 668 | [GO:0042157](file:///E:\2018-7-3%E7%81%AB%E9%BE%99%E6%9E%9C%E8%BD%AC%E5%BD%95%E7%BB%84%E6%B5%8B%E5%BA%8F\%E5%AE%8C%E6%95%B4%E7%89%88%E6%95%B0%E6%8D%AE\GDR3855-Hylocereus_undulatus_Britt-12-RNAseq_result\4_Function\2_Group_Diff_Function\UP_DOWN\GO\NL-VS-L1.P.html#gene668) | lipoprotein metabolic process | 1 (0.08%) | 3 (0.09%) | 0.746941 | 0.999992 |
| 669 | [GO:0042158](file:///E:\2018-7-3%E7%81%AB%E9%BE%99%E6%9E%9C%E8%BD%AC%E5%BD%95%E7%BB%84%E6%B5%8B%E5%BA%8F\%E5%AE%8C%E6%95%B4%E7%89%88%E6%95%B0%E6%8D%AE\GDR3855-Hylocereus_undulatus_Britt-12-RNAseq_result\4_Function\2_Group_Diff_Function\UP_DOWN\GO\NL-VS-L1.P.html#gene669) | lipoprotein biosynthetic process | 1 (0.08%) | 3 (0.09%) | 0.746941 | 0.999992 |
| 670 | [GO:0042214](file:///E:\2018-7-3%E7%81%AB%E9%BE%99%E6%9E%9C%E8%BD%AC%E5%BD%95%E7%BB%84%E6%B5%8B%E5%BA%8F\%E5%AE%8C%E6%95%B4%E7%89%88%E6%95%B0%E6%8D%AE\GDR3855-Hylocereus_undulatus_Britt-12-RNAseq_result\4_Function\2_Group_Diff_Function\UP_DOWN\GO\NL-VS-L1.P.html#gene670) | terpene metabolic process | 1 (0.08%) | 3 (0.09%) | 0.746941 | 0.999992 |
| 671 | [GO:0042726](file:///E:\2018-7-3%E7%81%AB%E9%BE%99%E6%9E%9C%E8%BD%AC%E5%BD%95%E7%BB%84%E6%B5%8B%E5%BA%8F\%E5%AE%8C%E6%95%B4%E7%89%88%E6%95%B0%E6%8D%AE\GDR3855-Hylocereus_undulatus_Britt-12-RNAseq_result\4_Function\2_Group_Diff_Function\UP_DOWN\GO\NL-VS-L1.P.html#gene671) | flavin-containing compound metabolic process | 1 (0.08%) | 3 (0.09%) | 0.746941 | 0.999992 |
| 672 | [GO:0043647](file:///E:\2018-7-3%E7%81%AB%E9%BE%99%E6%9E%9C%E8%BD%AC%E5%BD%95%E7%BB%84%E6%B5%8B%E5%BA%8F\%E5%AE%8C%E6%95%B4%E7%89%88%E6%95%B0%E6%8D%AE\GDR3855-Hylocereus_undulatus_Britt-12-RNAseq_result\4_Function\2_Group_Diff_Function\UP_DOWN\GO\NL-VS-L1.P.html#gene672) | inositol phosphate metabolic process | 1 (0.08%) | 3 (0.09%) | 0.746941 | 0.999992 |
| 673 | [GO:0043900](file:///E:\2018-7-3%E7%81%AB%E9%BE%99%E6%9E%9C%E8%BD%AC%E5%BD%95%E7%BB%84%E6%B5%8B%E5%BA%8F\%E5%AE%8C%E6%95%B4%E7%89%88%E6%95%B0%E6%8D%AE\GDR3855-Hylocereus_undulatus_Britt-12-RNAseq_result\4_Function\2_Group_Diff_Function\UP_DOWN\GO\NL-VS-L1.P.html#gene673) | regulation of multi-organism process | 1 (0.08%) | 3 (0.09%) | 0.746941 | 0.999992 |
| 674 | [GO:0044764](file:///E:\2018-7-3%E7%81%AB%E9%BE%99%E6%9E%9C%E8%BD%AC%E5%BD%95%E7%BB%84%E6%B5%8B%E5%BA%8F\%E5%AE%8C%E6%95%B4%E7%89%88%E6%95%B0%E6%8D%AE\GDR3855-Hylocereus_undulatus_Britt-12-RNAseq_result\4_Function\2_Group_Diff_Function\UP_DOWN\GO\NL-VS-L1.P.html#gene674) | multi-organism cellular process | 1 (0.08%) | 3 (0.09%) | 0.746941 | 0.999992 |
| 675 | [GO:0046173](file:///E:\2018-7-3%E7%81%AB%E9%BE%99%E6%9E%9C%E8%BD%AC%E5%BD%95%E7%BB%84%E6%B5%8B%E5%BA%8F\%E5%AE%8C%E6%95%B4%E7%89%88%E6%95%B0%E6%8D%AE\GDR3855-Hylocereus_undulatus_Britt-12-RNAseq_result\4_Function\2_Group_Diff_Function\UP_DOWN\GO\NL-VS-L1.P.html#gene675) | polyol biosynthetic process | 1 (0.08%) | 3 (0.09%) | 0.746941 | 0.999992 |
| 676 | [GO:0051156](file:///E:\2018-7-3%E7%81%AB%E9%BE%99%E6%9E%9C%E8%BD%AC%E5%BD%95%E7%BB%84%E6%B5%8B%E5%BA%8F\%E5%AE%8C%E6%95%B4%E7%89%88%E6%95%B0%E6%8D%AE\GDR3855-Hylocereus_undulatus_Britt-12-RNAseq_result\4_Function\2_Group_Diff_Function\UP_DOWN\GO\NL-VS-L1.P.html#gene676) | glucose 6-phosphate metabolic process | 1 (0.08%) | 3 (0.09%) | 0.746941 | 0.999992 |
| 677 | [GO:0055088](file:///E:\2018-7-3%E7%81%AB%E9%BE%99%E6%9E%9C%E8%BD%AC%E5%BD%95%E7%BB%84%E6%B5%8B%E5%BA%8F\%E5%AE%8C%E6%95%B4%E7%89%88%E6%95%B0%E6%8D%AE\GDR3855-Hylocereus_undulatus_Britt-12-RNAseq_result\4_Function\2_Group_Diff_Function\UP_DOWN\GO\NL-VS-L1.P.html#gene677) | lipid homeostasis | 1 (0.08%) | 3 (0.09%) | 0.746941 | 0.999992 |
| 678 | [GO:0060249](file:///E:\2018-7-3%E7%81%AB%E9%BE%99%E6%9E%9C%E8%BD%AC%E5%BD%95%E7%BB%84%E6%B5%8B%E5%BA%8F\%E5%AE%8C%E6%95%B4%E7%89%88%E6%95%B0%E6%8D%AE\GDR3855-Hylocereus_undulatus_Britt-12-RNAseq_result\4_Function\2_Group_Diff_Function\UP_DOWN\GO\NL-VS-L1.P.html#gene678) | anatomical structure homeostasis | 1 (0.08%) | 3 (0.09%) | 0.746941 | 0.999992 |
| 679 | [GO:0072502](file:///E:\2018-7-3%E7%81%AB%E9%BE%99%E6%9E%9C%E8%BD%AC%E5%BD%95%E7%BB%84%E6%B5%8B%E5%BA%8F\%E5%AE%8C%E6%95%B4%E7%89%88%E6%95%B0%E6%8D%AE\GDR3855-Hylocereus_undulatus_Britt-12-RNAseq_result\4_Function\2_Group_Diff_Function\UP_DOWN\GO\NL-VS-L1.P.html#gene679) | cellular trivalent inorganic anion homeostasis | 1 (0.08%) | 3 (0.09%) | 0.746941 | 0.999992 |
| 680 | [GO:0072506](file:///E:\2018-7-3%E7%81%AB%E9%BE%99%E6%9E%9C%E8%BD%AC%E5%BD%95%E7%BB%84%E6%B5%8B%E5%BA%8F\%E5%AE%8C%E6%95%B4%E7%89%88%E6%95%B0%E6%8D%AE\GDR3855-Hylocereus_undulatus_Britt-12-RNAseq_result\4_Function\2_Group_Diff_Function\UP_DOWN\GO\NL-VS-L1.P.html#gene680) | trivalent inorganic anion homeostasis | 1 (0.08%) | 3 (0.09%) | 0.746941 | 0.999992 |
| 681 | [GO:0006720](file:///E:\2018-7-3%E7%81%AB%E9%BE%99%E6%9E%9C%E8%BD%AC%E5%BD%95%E7%BB%84%E6%B5%8B%E5%BA%8F\%E5%AE%8C%E6%95%B4%E7%89%88%E6%95%B0%E6%8D%AE\GDR3855-Hylocereus_undulatus_Britt-12-RNAseq_result\4_Function\2_Group_Diff_Function\UP_DOWN\GO\NL-VS-L1.P.html#gene681) | isoprenoid metabolic process | 8 (0.65%) | 25 (0.74%) | 0.755051 | 0.999992 |
| 682 | [GO:0048519](file:///E:\2018-7-3%E7%81%AB%E9%BE%99%E6%9E%9C%E8%BD%AC%E5%BD%95%E7%BB%84%E6%B5%8B%E5%BA%8F\%E5%AE%8C%E6%95%B4%E7%89%88%E6%95%B0%E6%8D%AE\GDR3855-Hylocereus_undulatus_Britt-12-RNAseq_result\4_Function\2_Group_Diff_Function\UP_DOWN\GO\NL-VS-L1.P.html#gene682) | negative regulation of biological process | 8 (0.65%) | 25 (0.74%) | 0.755051 | 0.999992 |
| 683 | [GO:0044763](file:///E:\2018-7-3%E7%81%AB%E9%BE%99%E6%9E%9C%E8%BD%AC%E5%BD%95%E7%BB%84%E6%B5%8B%E5%BA%8F\%E5%AE%8C%E6%95%B4%E7%89%88%E6%95%B0%E6%8D%AE\GDR3855-Hylocereus_undulatus_Britt-12-RNAseq_result\4_Function\2_Group_Diff_Function\UP_DOWN\GO\NL-VS-L1.P.html#gene683) | single-organism cellular process | 398 (32.25%) | 1107 (32.96%) | 0.757527 | 0.999992 |
| 684 | [GO:0009908](file:///E:\2018-7-3%E7%81%AB%E9%BE%99%E6%9E%9C%E8%BD%AC%E5%BD%95%E7%BB%84%E6%B5%8B%E5%BA%8F\%E5%AE%8C%E6%95%B4%E7%89%88%E6%95%B0%E6%8D%AE\GDR3855-Hylocereus_undulatus_Britt-12-RNAseq_result\4_Function\2_Group_Diff_Function\UP_DOWN\GO\NL-VS-L1.P.html#gene684) | flower development | 5 (0.41%) | 16 (0.48%) | 0.758756 | 0.999992 |
| 685 | [GO:0015988](file:///E:\2018-7-3%E7%81%AB%E9%BE%99%E6%9E%9C%E8%BD%AC%E5%BD%95%E7%BB%84%E6%B5%8B%E5%BA%8F\%E5%AE%8C%E6%95%B4%E7%89%88%E6%95%B0%E6%8D%AE\GDR3855-Hylocereus_undulatus_Britt-12-RNAseq_result\4_Function\2_Group_Diff_Function\UP_DOWN\GO\NL-VS-L1.P.html#gene685) | energy coupled proton transmembrane transport, against electrochemical gradient | 5 (0.41%) | 16 (0.48%) | 0.758756 | 0.999992 |
| 686 | [GO:0048513](file:///E:\2018-7-3%E7%81%AB%E9%BE%99%E6%9E%9C%E8%BD%AC%E5%BD%95%E7%BB%84%E6%B5%8B%E5%BA%8F\%E5%AE%8C%E6%95%B4%E7%89%88%E6%95%B0%E6%8D%AE\GDR3855-Hylocereus_undulatus_Britt-12-RNAseq_result\4_Function\2_Group_Diff_Function\UP_DOWN\GO\NL-VS-L1.P.html#gene686) | animal organ development | 5 (0.41%) | 16 (0.48%) | 0.758756 | 0.999992 |
| 687 | [GO:0010604](file:///E:\2018-7-3%E7%81%AB%E9%BE%99%E6%9E%9C%E8%BD%AC%E5%BD%95%E7%BB%84%E6%B5%8B%E5%BA%8F\%E5%AE%8C%E6%95%B4%E7%89%88%E6%95%B0%E6%8D%AE\GDR3855-Hylocereus_undulatus_Britt-12-RNAseq_result\4_Function\2_Group_Diff_Function\UP_DOWN\GO\NL-VS-L1.P.html#gene687) | positive regulation of macromolecule metabolic process | 12 (0.97%) | 37 (1.1%) | 0.760967 | 0.999992 |
| 688 | [GO:0000904](file:///E:\2018-7-3%E7%81%AB%E9%BE%99%E6%9E%9C%E8%BD%AC%E5%BD%95%E7%BB%84%E6%B5%8B%E5%BA%8F\%E5%AE%8C%E6%95%B4%E7%89%88%E6%95%B0%E6%8D%AE\GDR3855-Hylocereus_undulatus_Britt-12-RNAseq_result\4_Function\2_Group_Diff_Function\UP_DOWN\GO\NL-VS-L1.P.html#gene688) | cell morphogenesis involved in differentiation | 4 (0.32%) | 13 (0.39%) | 0.764272 | 0.999992 |
| 689 | [GO:0006575](file:///E:\2018-7-3%E7%81%AB%E9%BE%99%E6%9E%9C%E8%BD%AC%E5%BD%95%E7%BB%84%E6%B5%8B%E5%BA%8F\%E5%AE%8C%E6%95%B4%E7%89%88%E6%95%B0%E6%8D%AE\GDR3855-Hylocereus_undulatus_Britt-12-RNAseq_result\4_Function\2_Group_Diff_Function\UP_DOWN\GO\NL-VS-L1.P.html#gene689) | cellular modified amino acid metabolic process | 4 (0.32%) | 13 (0.39%) | 0.764272 | 0.999992 |
| 690 | [GO:0006779](file:///E:\2018-7-3%E7%81%AB%E9%BE%99%E6%9E%9C%E8%BD%AC%E5%BD%95%E7%BB%84%E6%B5%8B%E5%BA%8F\%E5%AE%8C%E6%95%B4%E7%89%88%E6%95%B0%E6%8D%AE\GDR3855-Hylocereus_undulatus_Britt-12-RNAseq_result\4_Function\2_Group_Diff_Function\UP_DOWN\GO\NL-VS-L1.P.html#gene690) | porphyrin-containing compound biosynthetic process | 4 (0.32%) | 13 (0.39%) | 0.764272 | 0.999992 |
| 691 | [GO:0016049](file:///E:\2018-7-3%E7%81%AB%E9%BE%99%E6%9E%9C%E8%BD%AC%E5%BD%95%E7%BB%84%E6%B5%8B%E5%BA%8F\%E5%AE%8C%E6%95%B4%E7%89%88%E6%95%B0%E6%8D%AE\GDR3855-Hylocereus_undulatus_Britt-12-RNAseq_result\4_Function\2_Group_Diff_Function\UP_DOWN\GO\NL-VS-L1.P.html#gene691) | cell growth | 4 (0.32%) | 13 (0.39%) | 0.764272 | 0.999992 |
| 692 | [GO:0006074](file:///E:\2018-7-3%E7%81%AB%E9%BE%99%E6%9E%9C%E8%BD%AC%E5%BD%95%E7%BB%84%E6%B5%8B%E5%BA%8F\%E5%AE%8C%E6%95%B4%E7%89%88%E6%95%B0%E6%8D%AE\GDR3855-Hylocereus_undulatus_Britt-12-RNAseq_result\4_Function\2_Group_Diff_Function\UP_DOWN\GO\NL-VS-L1.P.html#gene692) | (1->3)-beta-D-glucan metabolic process | 3 (0.24%) | 10 (0.3%) | 0.774671 | 0.999992 |
| 693 | [GO:0006511](file:///E:\2018-7-3%E7%81%AB%E9%BE%99%E6%9E%9C%E8%BD%AC%E5%BD%95%E7%BB%84%E6%B5%8B%E5%BA%8F\%E5%AE%8C%E6%95%B4%E7%89%88%E6%95%B0%E6%8D%AE\GDR3855-Hylocereus_undulatus_Britt-12-RNAseq_result\4_Function\2_Group_Diff_Function\UP_DOWN\GO\NL-VS-L1.P.html#gene693) | ubiquitin-dependent protein catabolic process | 3 (0.24%) | 10 (0.3%) | 0.774671 | 0.999992 |
| 694 | [GO:0014070](file:///E:\2018-7-3%E7%81%AB%E9%BE%99%E6%9E%9C%E8%BD%AC%E5%BD%95%E7%BB%84%E6%B5%8B%E5%BA%8F\%E5%AE%8C%E6%95%B4%E7%89%88%E6%95%B0%E6%8D%AE\GDR3855-Hylocereus_undulatus_Britt-12-RNAseq_result\4_Function\2_Group_Diff_Function\UP_DOWN\GO\NL-VS-L1.P.html#gene694) | response to organic cyclic compound | 3 (0.24%) | 10 (0.3%) | 0.774671 | 0.999992 |
| 695 | [GO:0035556](file:///E:\2018-7-3%E7%81%AB%E9%BE%99%E6%9E%9C%E8%BD%AC%E5%BD%95%E7%BB%84%E6%B5%8B%E5%BA%8F\%E5%AE%8C%E6%95%B4%E7%89%88%E6%95%B0%E6%8D%AE\GDR3855-Hylocereus_undulatus_Britt-12-RNAseq_result\4_Function\2_Group_Diff_Function\UP_DOWN\GO\NL-VS-L1.P.html#gene695) | intracellular signal transduction | 19 (1.54%) | 58 (1.73%) | 0.778195 | 0.999992 |
| 696 | [GO:0050790](file:///E:\2018-7-3%E7%81%AB%E9%BE%99%E6%9E%9C%E8%BD%AC%E5%BD%95%E7%BB%84%E6%B5%8B%E5%BA%8F\%E5%AE%8C%E6%95%B4%E7%89%88%E6%95%B0%E6%8D%AE\GDR3855-Hylocereus_undulatus_Britt-12-RNAseq_result\4_Function\2_Group_Diff_Function\UP_DOWN\GO\NL-VS-L1.P.html#gene696) | regulation of catalytic activity | 19 (1.54%) | 58 (1.73%) | 0.778195 | 0.999992 |
| 697 | [GO:0050896](file:///E:\2018-7-3%E7%81%AB%E9%BE%99%E6%9E%9C%E8%BD%AC%E5%BD%95%E7%BB%84%E6%B5%8B%E5%BA%8F\%E5%AE%8C%E6%95%B4%E7%89%88%E6%95%B0%E6%8D%AE\GDR3855-Hylocereus_undulatus_Britt-12-RNAseq_result\4_Function\2_Group_Diff_Function\UP_DOWN\GO\NL-VS-L1.P.html#gene697) | response to stimulus | 207 (16.77%) | 585 (17.42%) | 0.786066 | 0.999992 |
| 698 | [GO:0006397](file:///E:\2018-7-3%E7%81%AB%E9%BE%99%E6%9E%9C%E8%BD%AC%E5%BD%95%E7%BB%84%E6%B5%8B%E5%BA%8F\%E5%AE%8C%E6%95%B4%E7%89%88%E6%95%B0%E6%8D%AE\GDR3855-Hylocereus_undulatus_Britt-12-RNAseq_result\4_Function\2_Group_Diff_Function\UP_DOWN\GO\NL-VS-L1.P.html#gene698) | mRNA processing | 2 (0.16%) | 7 (0.21%) | 0.794873 | 0.999992 |
| 699 | [GO:0006760](file:///E:\2018-7-3%E7%81%AB%E9%BE%99%E6%9E%9C%E8%BD%AC%E5%BD%95%E7%BB%84%E6%B5%8B%E5%BA%8F\%E5%AE%8C%E6%95%B4%E7%89%88%E6%95%B0%E6%8D%AE\GDR3855-Hylocereus_undulatus_Britt-12-RNAseq_result\4_Function\2_Group_Diff_Function\UP_DOWN\GO\NL-VS-L1.P.html#gene699) | folic acid-containing compound metabolic process | 2 (0.16%) | 7 (0.21%) | 0.794873 | 0.999992 |
| 700 | [GO:0009409](file:///E:\2018-7-3%E7%81%AB%E9%BE%99%E6%9E%9C%E8%BD%AC%E5%BD%95%E7%BB%84%E6%B5%8B%E5%BA%8F\%E5%AE%8C%E6%95%B4%E7%89%88%E6%95%B0%E6%8D%AE\GDR3855-Hylocereus_undulatus_Britt-12-RNAseq_result\4_Function\2_Group_Diff_Function\UP_DOWN\GO\NL-VS-L1.P.html#gene700) | response to cold | 2 (0.16%) | 7 (0.21%) | 0.794873 | 0.999992 |
| 701 | [GO:0042558](file:///E:\2018-7-3%E7%81%AB%E9%BE%99%E6%9E%9C%E8%BD%AC%E5%BD%95%E7%BB%84%E6%B5%8B%E5%BA%8F\%E5%AE%8C%E6%95%B4%E7%89%88%E6%95%B0%E6%8D%AE\GDR3855-Hylocereus_undulatus_Britt-12-RNAseq_result\4_Function\2_Group_Diff_Function\UP_DOWN\GO\NL-VS-L1.P.html#gene701) | pteridine-containing compound metabolic process | 2 (0.16%) | 7 (0.21%) | 0.794873 | 0.999992 |
| 702 | [GO:0055065](file:///E:\2018-7-3%E7%81%AB%E9%BE%99%E6%9E%9C%E8%BD%AC%E5%BD%95%E7%BB%84%E6%B5%8B%E5%BA%8F\%E5%AE%8C%E6%95%B4%E7%89%88%E6%95%B0%E6%8D%AE\GDR3855-Hylocereus_undulatus_Britt-12-RNAseq_result\4_Function\2_Group_Diff_Function\UP_DOWN\GO\NL-VS-L1.P.html#gene702) | metal ion homeostasis | 2 (0.16%) | 7 (0.21%) | 0.794873 | 0.999992 |
| 703 | [GO:0034660](file:///E:\2018-7-3%E7%81%AB%E9%BE%99%E6%9E%9C%E8%BD%AC%E5%BD%95%E7%BB%84%E6%B5%8B%E5%BA%8F\%E5%AE%8C%E6%95%B4%E7%89%88%E6%95%B0%E6%8D%AE\GDR3855-Hylocereus_undulatus_Britt-12-RNAseq_result\4_Function\2_Group_Diff_Function\UP_DOWN\GO\NL-VS-L1.P.html#gene703) | ncRNA metabolic process | 10 (0.81%) | 32 (0.95%) | 0.795424 | 0.999992 |
| 704 | [GO:0048608](file:///E:\2018-7-3%E7%81%AB%E9%BE%99%E6%9E%9C%E8%BD%AC%E5%BD%95%E7%BB%84%E6%B5%8B%E5%BA%8F\%E5%AE%8C%E6%95%B4%E7%89%88%E6%95%B0%E6%8D%AE\GDR3855-Hylocereus_undulatus_Britt-12-RNAseq_result\4_Function\2_Group_Diff_Function\UP_DOWN\GO\NL-VS-L1.P.html#gene704) | reproductive structure development | 10 (0.81%) | 32 (0.95%) | 0.795424 | 0.999992 |
| 705 | [GO:0061458](file:///E:\2018-7-3%E7%81%AB%E9%BE%99%E6%9E%9C%E8%BD%AC%E5%BD%95%E7%BB%84%E6%B5%8B%E5%BA%8F\%E5%AE%8C%E6%95%B4%E7%89%88%E6%95%B0%E6%8D%AE\GDR3855-Hylocereus_undulatus_Britt-12-RNAseq_result\4_Function\2_Group_Diff_Function\UP_DOWN\GO\NL-VS-L1.P.html#gene705) | reproductive system development | 10 (0.81%) | 32 (0.95%) | 0.795424 | 0.999992 |
| 706 | [GO:0006979](file:///E:\2018-7-3%E7%81%AB%E9%BE%99%E6%9E%9C%E8%BD%AC%E5%BD%95%E7%BB%84%E6%B5%8B%E5%BA%8F\%E5%AE%8C%E6%95%B4%E7%89%88%E6%95%B0%E6%8D%AE\GDR3855-Hylocereus_undulatus_Britt-12-RNAseq_result\4_Function\2_Group_Diff_Function\UP_DOWN\GO\NL-VS-L1.P.html#gene706) | response to oxidative stress | 11 (0.89%) | 35 (1.04%) | 0.795493 | 0.999992 |
| 707 | [GO:0090567](file:///E:\2018-7-3%E7%81%AB%E9%BE%99%E6%9E%9C%E8%BD%AC%E5%BD%95%E7%BB%84%E6%B5%8B%E5%BA%8F\%E5%AE%8C%E6%95%B4%E7%89%88%E6%95%B0%E6%8D%AE\GDR3855-Hylocereus_undulatus_Britt-12-RNAseq_result\4_Function\2_Group_Diff_Function\UP_DOWN\GO\NL-VS-L1.P.html#gene707) | reproductive shoot system development | 9 (0.73%) | 29 (0.86%) | 0.795914 | 0.999992 |
| 708 | [GO:0043412](file:///E:\2018-7-3%E7%81%AB%E9%BE%99%E6%9E%9C%E8%BD%AC%E5%BD%95%E7%BB%84%E6%B5%8B%E5%BA%8F\%E5%AE%8C%E6%95%B4%E7%89%88%E6%95%B0%E6%8D%AE\GDR3855-Hylocereus_undulatus_Britt-12-RNAseq_result\4_Function\2_Group_Diff_Function\UP_DOWN\GO\NL-VS-L1.P.html#gene708) | macromolecule modification | 136 (11.02%) | 389 (11.58%) | 0.795956 | 0.999992 |
| 709 | [GO:0048367](file:///E:\2018-7-3%E7%81%AB%E9%BE%99%E6%9E%9C%E8%BD%AC%E5%BD%95%E7%BB%84%E6%B5%8B%E5%BA%8F\%E5%AE%8C%E6%95%B4%E7%89%88%E6%95%B0%E6%8D%AE\GDR3855-Hylocereus_undulatus_Britt-12-RNAseq_result\4_Function\2_Group_Diff_Function\UP_DOWN\GO\NL-VS-L1.P.html#gene709) | shoot system development | 12 (0.97%) | 38 (1.13%) | 0.795989 | 0.999992 |
| 710 | [GO:0006464](file:///E:\2018-7-3%E7%81%AB%E9%BE%99%E6%9E%9C%E8%BD%AC%E5%BD%95%E7%BB%84%E6%B5%8B%E5%BA%8F\%E5%AE%8C%E6%95%B4%E7%89%88%E6%95%B0%E6%8D%AE\GDR3855-Hylocereus_undulatus_Britt-12-RNAseq_result\4_Function\2_Group_Diff_Function\UP_DOWN\GO\NL-VS-L1.P.html#gene710) | cellular protein modification process | 128 (10.37%) | 367 (10.93%) | 0.799320 | 0.999992 |
| 711 | [GO:0036211](file:///E:\2018-7-3%E7%81%AB%E9%BE%99%E6%9E%9C%E8%BD%AC%E5%BD%95%E7%BB%84%E6%B5%8B%E5%BA%8F\%E5%AE%8C%E6%95%B4%E7%89%88%E6%95%B0%E6%8D%AE\GDR3855-Hylocereus_undulatus_Britt-12-RNAseq_result\4_Function\2_Group_Diff_Function\UP_DOWN\GO\NL-VS-L1.P.html#gene711) | protein modification process | 129 (10.45%) | 370 (11.02%) | 0.801765 | 0.999992 |
| 712 | [GO:0044550](file:///E:\2018-7-3%E7%81%AB%E9%BE%99%E6%9E%9C%E8%BD%AC%E5%BD%95%E7%BB%84%E6%B5%8B%E5%BA%8F\%E5%AE%8C%E6%95%B4%E7%89%88%E6%95%B0%E6%8D%AE\GDR3855-Hylocereus_undulatus_Britt-12-RNAseq_result\4_Function\2_Group_Diff_Function\UP_DOWN\GO\NL-VS-L1.P.html#gene712) | secondary metabolite biosynthetic process | 6 (0.49%) | 20 (0.6%) | 0.803100 | 0.999992 |
| 713 | [GO:0044802](file:///E:\2018-7-3%E7%81%AB%E9%BE%99%E6%9E%9C%E8%BD%AC%E5%BD%95%E7%BB%84%E6%B5%8B%E5%BA%8F\%E5%AE%8C%E6%95%B4%E7%89%88%E6%95%B0%E6%8D%AE\GDR3855-Hylocereus_undulatus_Britt-12-RNAseq_result\4_Function\2_Group_Diff_Function\UP_DOWN\GO\NL-VS-L1.P.html#gene713) | single-organism membrane organization | 6 (0.49%) | 20 (0.6%) | 0.803100 | 0.999992 |
| 714 | [GO:0051336](file:///E:\2018-7-3%E7%81%AB%E9%BE%99%E6%9E%9C%E8%BD%AC%E5%BD%95%E7%BB%84%E6%B5%8B%E5%BA%8F\%E5%AE%8C%E6%95%B4%E7%89%88%E6%95%B0%E6%8D%AE\GDR3855-Hylocereus_undulatus_Britt-12-RNAseq_result\4_Function\2_Group_Diff_Function\UP_DOWN\GO\NL-VS-L1.P.html#gene714) | regulation of hydrolase activity | 6 (0.49%) | 20 (0.6%) | 0.803100 | 0.999992 |
| 715 | [GO:0061024](file:///E:\2018-7-3%E7%81%AB%E9%BE%99%E6%9E%9C%E8%BD%AC%E5%BD%95%E7%BB%84%E6%B5%8B%E5%BA%8F\%E5%AE%8C%E6%95%B4%E7%89%88%E6%95%B0%E6%8D%AE\GDR3855-Hylocereus_undulatus_Britt-12-RNAseq_result\4_Function\2_Group_Diff_Function\UP_DOWN\GO\NL-VS-L1.P.html#gene715) | membrane organization | 6 (0.49%) | 20 (0.6%) | 0.803100 | 0.999992 |
| 716 | [GO:0033036](file:///E:\2018-7-3%E7%81%AB%E9%BE%99%E6%9E%9C%E8%BD%AC%E5%BD%95%E7%BB%84%E6%B5%8B%E5%BA%8F\%E5%AE%8C%E6%95%B4%E7%89%88%E6%95%B0%E6%8D%AE\GDR3855-Hylocereus_undulatus_Britt-12-RNAseq_result\4_Function\2_Group_Diff_Function\UP_DOWN\GO\NL-VS-L1.P.html#gene716) | macromolecule localization | 45 (3.65%) | 134 (3.99%) | 0.805823 | 0.999992 |
| 717 | [GO:1901617](file:///E:\2018-7-3%E7%81%AB%E9%BE%99%E6%9E%9C%E8%BD%AC%E5%BD%95%E7%BB%84%E6%B5%8B%E5%BA%8F\%E5%AE%8C%E6%95%B4%E7%89%88%E6%95%B0%E6%8D%AE\GDR3855-Hylocereus_undulatus_Britt-12-RNAseq_result\4_Function\2_Group_Diff_Function\UP_DOWN\GO\NL-VS-L1.P.html#gene717) | organic hydroxy compound biosynthetic process | 5 (0.41%) | 17 (0.51%) | 0.808870 | 0.999992 |
| 718 | [GO:0007154](file:///E:\2018-7-3%E7%81%AB%E9%BE%99%E6%9E%9C%E8%BD%AC%E5%BD%95%E7%BB%84%E6%B5%8B%E5%BA%8F\%E5%AE%8C%E6%95%B4%E7%89%88%E6%95%B0%E6%8D%AE\GDR3855-Hylocereus_undulatus_Britt-12-RNAseq_result\4_Function\2_Group_Diff_Function\UP_DOWN\GO\NL-VS-L1.P.html#gene718) | cell communication | 65 (5.27%) | 191 (5.69%) | 0.809076 | 0.999992 |
| 719 | [GO:0051716](file:///E:\2018-7-3%E7%81%AB%E9%BE%99%E6%9E%9C%E8%BD%AC%E5%BD%95%E7%BB%84%E6%B5%8B%E5%BA%8F\%E5%AE%8C%E6%95%B4%E7%89%88%E6%95%B0%E6%8D%AE\GDR3855-Hylocereus_undulatus_Britt-12-RNAseq_result\4_Function\2_Group_Diff_Function\UP_DOWN\GO\NL-VS-L1.P.html#gene719) | cellular response to stimulus | 72 (5.83%) | 211 (6.28%) | 0.812205 | 0.999992 |
| 720 | [GO:0006950](file:///E:\2018-7-3%E7%81%AB%E9%BE%99%E6%9E%9C%E8%BD%AC%E5%BD%95%E7%BB%84%E6%B5%8B%E5%BA%8F\%E5%AE%8C%E6%95%B4%E7%89%88%E6%95%B0%E6%8D%AE\GDR3855-Hylocereus_undulatus_Britt-12-RNAseq_result\4_Function\2_Group_Diff_Function\UP_DOWN\GO\NL-VS-L1.P.html#gene720) | response to stress | 94 (7.62%) | 273 (8.13%) | 0.812891 | 0.999992 |
| 721 | [GO:0043087](file:///E:\2018-7-3%E7%81%AB%E9%BE%99%E6%9E%9C%E8%BD%AC%E5%BD%95%E7%BB%84%E6%B5%8B%E5%BA%8F\%E5%AE%8C%E6%95%B4%E7%89%88%E6%95%B0%E6%8D%AE\GDR3855-Hylocereus_undulatus_Britt-12-RNAseq_result\4_Function\2_Group_Diff_Function\UP_DOWN\GO\NL-VS-L1.P.html#gene721) | regulation of GTPase activity | 4 (0.32%) | 14 (0.42%) | 0.817819 | 0.999992 |
| 722 | [GO:0009653](file:///E:\2018-7-3%E7%81%AB%E9%BE%99%E6%9E%9C%E8%BD%AC%E5%BD%95%E7%BB%84%E6%B5%8B%E5%BA%8F\%E5%AE%8C%E6%95%B4%E7%89%88%E6%95%B0%E6%8D%AE\GDR3855-Hylocereus_undulatus_Britt-12-RNAseq_result\4_Function\2_Group_Diff_Function\UP_DOWN\GO\NL-VS-L1.P.html#gene722) | anatomical structure morphogenesis | 14 (1.13%) | 45 (1.34%) | 0.826956 | 0.999992 |
| 723 | [GO:0044702](file:///E:\2018-7-3%E7%81%AB%E9%BE%99%E6%9E%9C%E8%BD%AC%E5%BD%95%E7%BB%84%E6%B5%8B%E5%BA%8F\%E5%AE%8C%E6%95%B4%E7%89%88%E6%95%B0%E6%8D%AE\GDR3855-Hylocereus_undulatus_Britt-12-RNAseq_result\4_Function\2_Group_Diff_Function\UP_DOWN\GO\NL-VS-L1.P.html#gene723) | single organism reproductive process | 12 (0.97%) | 39 (1.16%) | 0.827142 | 0.999992 |
| 724 | [GO:0043603](file:///E:\2018-7-3%E7%81%AB%E9%BE%99%E6%9E%9C%E8%BD%AC%E5%BD%95%E7%BB%84%E6%B5%8B%E5%BA%8F\%E5%AE%8C%E6%95%B4%E7%89%88%E6%95%B0%E6%8D%AE\GDR3855-Hylocereus_undulatus_Britt-12-RNAseq_result\4_Function\2_Group_Diff_Function\UP_DOWN\GO\NL-VS-L1.P.html#gene724) | cellular amide metabolic process | 31 (2.51%) | 95 (2.83%) | 0.828713 | 0.999992 |
| 725 | [GO:0000302](file:///E:\2018-7-3%E7%81%AB%E9%BE%99%E6%9E%9C%E8%BD%AC%E5%BD%95%E7%BB%84%E6%B5%8B%E5%BA%8F\%E5%AE%8C%E6%95%B4%E7%89%88%E6%95%B0%E6%8D%AE\GDR3855-Hylocereus_undulatus_Britt-12-RNAseq_result\4_Function\2_Group_Diff_Function\UP_DOWN\GO\NL-VS-L1.P.html#gene725) | response to reactive oxygen species | 10 (0.81%) | 33 (0.98%) | 0.829006 | 0.999992 |
| 726 | [GO:0006221](file:///E:\2018-7-3%E7%81%AB%E9%BE%99%E6%9E%9C%E8%BD%AC%E5%BD%95%E7%BB%84%E6%B5%8B%E5%BA%8F\%E5%AE%8C%E6%95%B4%E7%89%88%E6%95%B0%E6%8D%AE\GDR3855-Hylocereus_undulatus_Britt-12-RNAseq_result\4_Function\2_Group_Diff_Function\UP_DOWN\GO\NL-VS-L1.P.html#gene726) | pyrimidine nucleotide biosynthetic process | 3 (0.24%) | 11 (0.33%) | 0.831958 | 0.999992 |
| 727 | [GO:0016108](file:///E:\2018-7-3%E7%81%AB%E9%BE%99%E6%9E%9C%E8%BD%AC%E5%BD%95%E7%BB%84%E6%B5%8B%E5%BA%8F\%E5%AE%8C%E6%95%B4%E7%89%88%E6%95%B0%E6%8D%AE\GDR3855-Hylocereus_undulatus_Britt-12-RNAseq_result\4_Function\2_Group_Diff_Function\UP_DOWN\GO\NL-VS-L1.P.html#gene727) | tetraterpenoid metabolic process | 3 (0.24%) | 11 (0.33%) | 0.831958 | 0.999992 |
| 728 | [GO:0031324](file:///E:\2018-7-3%E7%81%AB%E9%BE%99%E6%9E%9C%E8%BD%AC%E5%BD%95%E7%BB%84%E6%B5%8B%E5%BA%8F\%E5%AE%8C%E6%95%B4%E7%89%88%E6%95%B0%E6%8D%AE\GDR3855-Hylocereus_undulatus_Britt-12-RNAseq_result\4_Function\2_Group_Diff_Function\UP_DOWN\GO\NL-VS-L1.P.html#gene728) | negative regulation of cellular metabolic process | 3 (0.24%) | 11 (0.33%) | 0.831958 | 0.999992 |
| 729 | [GO:0042254](file:///E:\2018-7-3%E7%81%AB%E9%BE%99%E6%9E%9C%E8%BD%AC%E5%BD%95%E7%BB%84%E6%B5%8B%E5%BA%8F\%E5%AE%8C%E6%95%B4%E7%89%88%E6%95%B0%E6%8D%AE\GDR3855-Hylocereus_undulatus_Britt-12-RNAseq_result\4_Function\2_Group_Diff_Function\UP_DOWN\GO\NL-VS-L1.P.html#gene729) | ribosome biogenesis | 3 (0.24%) | 11 (0.33%) | 0.831958 | 0.999992 |
| 730 | [GO:0048285](file:///E:\2018-7-3%E7%81%AB%E9%BE%99%E6%9E%9C%E8%BD%AC%E5%BD%95%E7%BB%84%E6%B5%8B%E5%BA%8F\%E5%AE%8C%E6%95%B4%E7%89%88%E6%95%B0%E6%8D%AE\GDR3855-Hylocereus_undulatus_Britt-12-RNAseq_result\4_Function\2_Group_Diff_Function\UP_DOWN\GO\NL-VS-L1.P.html#gene730) | organelle fission | 3 (0.24%) | 11 (0.33%) | 0.831958 | 0.999992 |
| 731 | [GO:0071103](file:///E:\2018-7-3%E7%81%AB%E9%BE%99%E6%9E%9C%E8%BD%AC%E5%BD%95%E7%BB%84%E6%B5%8B%E5%BA%8F\%E5%AE%8C%E6%95%B4%E7%89%88%E6%95%B0%E6%8D%AE\GDR3855-Hylocereus_undulatus_Britt-12-RNAseq_result\4_Function\2_Group_Diff_Function\UP_DOWN\GO\NL-VS-L1.P.html#gene731) | DNA conformation change | 3 (0.24%) | 11 (0.33%) | 0.831958 | 0.999992 |
| 732 | [GO:0072528](file:///E:\2018-7-3%E7%81%AB%E9%BE%99%E6%9E%9C%E8%BD%AC%E5%BD%95%E7%BB%84%E6%B5%8B%E5%BA%8F\%E5%AE%8C%E6%95%B4%E7%89%88%E6%95%B0%E6%8D%AE\GDR3855-Hylocereus_undulatus_Britt-12-RNAseq_result\4_Function\2_Group_Diff_Function\UP_DOWN\GO\NL-VS-L1.P.html#gene732) | pyrimidine-containing compound biosynthetic process | 3 (0.24%) | 11 (0.33%) | 0.831958 | 0.999992 |
| 733 | [GO:0050801](file:///E:\2018-7-3%E7%81%AB%E9%BE%99%E6%9E%9C%E8%BD%AC%E5%BD%95%E7%BB%84%E6%B5%8B%E5%BA%8F\%E5%AE%8C%E6%95%B4%E7%89%88%E6%95%B0%E6%8D%AE\GDR3855-Hylocereus_undulatus_Britt-12-RNAseq_result\4_Function\2_Group_Diff_Function\UP_DOWN\GO\NL-VS-L1.P.html#gene733) | ion homeostasis | 7 (0.57%) | 24 (0.71%) | 0.837417 | 0.999992 |
| 734 | [GO:0006259](file:///E:\2018-7-3%E7%81%AB%E9%BE%99%E6%9E%9C%E8%BD%AC%E5%BD%95%E7%BB%84%E6%B5%8B%E5%BA%8F\%E5%AE%8C%E6%95%B4%E7%89%88%E6%95%B0%E6%8D%AE\GDR3855-Hylocereus_undulatus_Britt-12-RNAseq_result\4_Function\2_Group_Diff_Function\UP_DOWN\GO\NL-VS-L1.P.html#gene734) | DNA metabolic process | 26 (2.11%) | 81 (2.41%) | 0.839683 | 0.999992 |
| 735 | [GO:0006739](file:///E:\2018-7-3%E7%81%AB%E9%BE%99%E6%9E%9C%E8%BD%AC%E5%BD%95%E7%BB%84%E6%B5%8B%E5%BA%8F\%E5%AE%8C%E6%95%B4%E7%89%88%E6%95%B0%E6%8D%AE\GDR3855-Hylocereus_undulatus_Britt-12-RNAseq_result\4_Function\2_Group_Diff_Function\UP_DOWN\GO\NL-VS-L1.P.html#gene735) | NADP metabolic process | 1 (0.08%) | 4 (0.12%) | 0.839991 | 0.999992 |
| 736 | [GO:0007602](file:///E:\2018-7-3%E7%81%AB%E9%BE%99%E6%9E%9C%E8%BD%AC%E5%BD%95%E7%BB%84%E6%B5%8B%E5%BA%8F\%E5%AE%8C%E6%95%B4%E7%89%88%E6%95%B0%E6%8D%AE\GDR3855-Hylocereus_undulatus_Britt-12-RNAseq_result\4_Function\2_Group_Diff_Function\UP_DOWN\GO\NL-VS-L1.P.html#gene736) | phototransduction | 1 (0.08%) | 4 (0.12%) | 0.839991 | 0.999992 |
| 737 | [GO:0008544](file:///E:\2018-7-3%E7%81%AB%E9%BE%99%E6%9E%9C%E8%BD%AC%E5%BD%95%E7%BB%84%E6%B5%8B%E5%BA%8F\%E5%AE%8C%E6%95%B4%E7%89%88%E6%95%B0%E6%8D%AE\GDR3855-Hylocereus_undulatus_Britt-12-RNAseq_result\4_Function\2_Group_Diff_Function\UP_DOWN\GO\NL-VS-L1.P.html#gene737) | epidermis development | 1 (0.08%) | 4 (0.12%) | 0.839991 | 0.999992 |
| 738 | [GO:0009581](file:///E:\2018-7-3%E7%81%AB%E9%BE%99%E6%9E%9C%E8%BD%AC%E5%BD%95%E7%BB%84%E6%B5%8B%E5%BA%8F\%E5%AE%8C%E6%95%B4%E7%89%88%E6%95%B0%E6%8D%AE\GDR3855-Hylocereus_undulatus_Britt-12-RNAseq_result\4_Function\2_Group_Diff_Function\UP_DOWN\GO\NL-VS-L1.P.html#gene738) | detection of external stimulus | 1 (0.08%) | 4 (0.12%) | 0.839991 | 0.999992 |
| 739 | [GO:0009582](file:///E:\2018-7-3%E7%81%AB%E9%BE%99%E6%9E%9C%E8%BD%AC%E5%BD%95%E7%BB%84%E6%B5%8B%E5%BA%8F\%E5%AE%8C%E6%95%B4%E7%89%88%E6%95%B0%E6%8D%AE\GDR3855-Hylocereus_undulatus_Britt-12-RNAseq_result\4_Function\2_Group_Diff_Function\UP_DOWN\GO\NL-VS-L1.P.html#gene739) | detection of abiotic stimulus | 1 (0.08%) | 4 (0.12%) | 0.839991 | 0.999992 |
| 740 | [GO:0009583](file:///E:\2018-7-3%E7%81%AB%E9%BE%99%E6%9E%9C%E8%BD%AC%E5%BD%95%E7%BB%84%E6%B5%8B%E5%BA%8F\%E5%AE%8C%E6%95%B4%E7%89%88%E6%95%B0%E6%8D%AE\GDR3855-Hylocereus_undulatus_Britt-12-RNAseq_result\4_Function\2_Group_Diff_Function\UP_DOWN\GO\NL-VS-L1.P.html#gene740) | detection of light stimulus | 1 (0.08%) | 4 (0.12%) | 0.839991 | 0.999992 |
| 741 | [GO:0009627](file:///E:\2018-7-3%E7%81%AB%E9%BE%99%E6%9E%9C%E8%BD%AC%E5%BD%95%E7%BB%84%E6%B5%8B%E5%BA%8F\%E5%AE%8C%E6%95%B4%E7%89%88%E6%95%B0%E6%8D%AE\GDR3855-Hylocereus_undulatus_Britt-12-RNAseq_result\4_Function\2_Group_Diff_Function\UP_DOWN\GO\NL-VS-L1.P.html#gene741) | systemic acquired resistance | 1 (0.08%) | 4 (0.12%) | 0.839991 | 0.999992 |
| 742 | [GO:0009913](file:///E:\2018-7-3%E7%81%AB%E9%BE%99%E6%9E%9C%E8%BD%AC%E5%BD%95%E7%BB%84%E6%B5%8B%E5%BA%8F\%E5%AE%8C%E6%95%B4%E7%89%88%E6%95%B0%E6%8D%AE\GDR3855-Hylocereus_undulatus_Britt-12-RNAseq_result\4_Function\2_Group_Diff_Function\UP_DOWN\GO\NL-VS-L1.P.html#gene742) | epidermal cell differentiation | 1 (0.08%) | 4 (0.12%) | 0.839991 | 0.999992 |
| 743 | [GO:0016116](file:///E:\2018-7-3%E7%81%AB%E9%BE%99%E6%9E%9C%E8%BD%AC%E5%BD%95%E7%BB%84%E6%B5%8B%E5%BA%8F\%E5%AE%8C%E6%95%B4%E7%89%88%E6%95%B0%E6%8D%AE\GDR3855-Hylocereus_undulatus_Britt-12-RNAseq_result\4_Function\2_Group_Diff_Function\UP_DOWN\GO\NL-VS-L1.P.html#gene743) | carotenoid metabolic process | 1 (0.08%) | 4 (0.12%) | 0.839991 | 0.999992 |
| 744 | [GO:0016567](file:///E:\2018-7-3%E7%81%AB%E9%BE%99%E6%9E%9C%E8%BD%AC%E5%BD%95%E7%BB%84%E6%B5%8B%E5%BA%8F\%E5%AE%8C%E6%95%B4%E7%89%88%E6%95%B0%E6%8D%AE\GDR3855-Hylocereus_undulatus_Britt-12-RNAseq_result\4_Function\2_Group_Diff_Function\UP_DOWN\GO\NL-VS-L1.P.html#gene744) | protein ubiquitination | 1 (0.08%) | 4 (0.12%) | 0.839991 | 0.999992 |
| 745 | [GO:0030855](file:///E:\2018-7-3%E7%81%AB%E9%BE%99%E6%9E%9C%E8%BD%AC%E5%BD%95%E7%BB%84%E6%B5%8B%E5%BA%8F\%E5%AE%8C%E6%95%B4%E7%89%88%E6%95%B0%E6%8D%AE\GDR3855-Hylocereus_undulatus_Britt-12-RNAseq_result\4_Function\2_Group_Diff_Function\UP_DOWN\GO\NL-VS-L1.P.html#gene745) | epithelial cell differentiation | 1 (0.08%) | 4 (0.12%) | 0.839991 | 0.999992 |
| 746 | [GO:0032504](file:///E:\2018-7-3%E7%81%AB%E9%BE%99%E6%9E%9C%E8%BD%AC%E5%BD%95%E7%BB%84%E6%B5%8B%E5%BA%8F\%E5%AE%8C%E6%95%B4%E7%89%88%E6%95%B0%E6%8D%AE\GDR3855-Hylocereus_undulatus_Britt-12-RNAseq_result\4_Function\2_Group_Diff_Function\UP_DOWN\GO\NL-VS-L1.P.html#gene746) | multicellular organism reproduction | 1 (0.08%) | 4 (0.12%) | 0.839991 | 0.999992 |
| 747 | [GO:0032879](file:///E:\2018-7-3%E7%81%AB%E9%BE%99%E6%9E%9C%E8%BD%AC%E5%BD%95%E7%BB%84%E6%B5%8B%E5%BA%8F\%E5%AE%8C%E6%95%B4%E7%89%88%E6%95%B0%E6%8D%AE\GDR3855-Hylocereus_undulatus_Britt-12-RNAseq_result\4_Function\2_Group_Diff_Function\UP_DOWN\GO\NL-VS-L1.P.html#gene747) | regulation of localization | 1 (0.08%) | 4 (0.12%) | 0.839991 | 0.999992 |
| 748 | [GO:0045682](file:///E:\2018-7-3%E7%81%AB%E9%BE%99%E6%9E%9C%E8%BD%AC%E5%BD%95%E7%BB%84%E6%B5%8B%E5%BA%8F\%E5%AE%8C%E6%95%B4%E7%89%88%E6%95%B0%E6%8D%AE\GDR3855-Hylocereus_undulatus_Britt-12-RNAseq_result\4_Function\2_Group_Diff_Function\UP_DOWN\GO\NL-VS-L1.P.html#gene748) | regulation of epidermis development | 1 (0.08%) | 4 (0.12%) | 0.839991 | 0.999992 |
| 749 | [GO:0048609](file:///E:\2018-7-3%E7%81%AB%E9%BE%99%E6%9E%9C%E8%BD%AC%E5%BD%95%E7%BB%84%E6%B5%8B%E5%BA%8F\%E5%AE%8C%E6%95%B4%E7%89%88%E6%95%B0%E6%8D%AE\GDR3855-Hylocereus_undulatus_Britt-12-RNAseq_result\4_Function\2_Group_Diff_Function\UP_DOWN\GO\NL-VS-L1.P.html#gene749) | multicellular organismal reproductive process | 1 (0.08%) | 4 (0.12%) | 0.839991 | 0.999992 |
| 750 | [GO:0051049](file:///E:\2018-7-3%E7%81%AB%E9%BE%99%E6%9E%9C%E8%BD%AC%E5%BD%95%E7%BB%84%E6%B5%8B%E5%BA%8F\%E5%AE%8C%E6%95%B4%E7%89%88%E6%95%B0%E6%8D%AE\GDR3855-Hylocereus_undulatus_Britt-12-RNAseq_result\4_Function\2_Group_Diff_Function\UP_DOWN\GO\NL-VS-L1.P.html#gene750) | regulation of transport | 1 (0.08%) | 4 (0.12%) | 0.839991 | 0.999992 |
| 751 | [GO:0051552](file:///E:\2018-7-3%E7%81%AB%E9%BE%99%E6%9E%9C%E8%BD%AC%E5%BD%95%E7%BB%84%E6%B5%8B%E5%BA%8F\%E5%AE%8C%E6%95%B4%E7%89%88%E6%95%B0%E6%8D%AE\GDR3855-Hylocereus_undulatus_Britt-12-RNAseq_result\4_Function\2_Group_Diff_Function\UP_DOWN\GO\NL-VS-L1.P.html#gene751) | flavone metabolic process | 1 (0.08%) | 4 (0.12%) | 0.839991 | 0.999992 |
| 752 | [GO:0051553](file:///E:\2018-7-3%E7%81%AB%E9%BE%99%E6%9E%9C%E8%BD%AC%E5%BD%95%E7%BB%84%E6%B5%8B%E5%BA%8F\%E5%AE%8C%E6%95%B4%E7%89%88%E6%95%B0%E6%8D%AE\GDR3855-Hylocereus_undulatus_Britt-12-RNAseq_result\4_Function\2_Group_Diff_Function\UP_DOWN\GO\NL-VS-L1.P.html#gene752) | flavone biosynthetic process | 1 (0.08%) | 4 (0.12%) | 0.839991 | 0.999992 |
| 753 | [GO:0051606](file:///E:\2018-7-3%E7%81%AB%E9%BE%99%E6%9E%9C%E8%BD%AC%E5%BD%95%E7%BB%84%E6%B5%8B%E5%BA%8F\%E5%AE%8C%E6%95%B4%E7%89%88%E6%95%B0%E6%8D%AE\GDR3855-Hylocereus_undulatus_Britt-12-RNAseq_result\4_Function\2_Group_Diff_Function\UP_DOWN\GO\NL-VS-L1.P.html#gene753) | detection of stimulus | 1 (0.08%) | 4 (0.12%) | 0.839991 | 0.999992 |
| 754 | [GO:0051640](file:///E:\2018-7-3%E7%81%AB%E9%BE%99%E6%9E%9C%E8%BD%AC%E5%BD%95%E7%BB%84%E6%B5%8B%E5%BA%8F\%E5%AE%8C%E6%95%B4%E7%89%88%E6%95%B0%E6%8D%AE\GDR3855-Hylocereus_undulatus_Britt-12-RNAseq_result\4_Function\2_Group_Diff_Function\UP_DOWN\GO\NL-VS-L1.P.html#gene754) | organelle localization | 1 (0.08%) | 4 (0.12%) | 0.839991 | 0.999992 |
| 755 | [GO:0055081](file:///E:\2018-7-3%E7%81%AB%E9%BE%99%E6%9E%9C%E8%BD%AC%E5%BD%95%E7%BB%84%E6%B5%8B%E5%BA%8F\%E5%AE%8C%E6%95%B4%E7%89%88%E6%95%B0%E6%8D%AE\GDR3855-Hylocereus_undulatus_Britt-12-RNAseq_result\4_Function\2_Group_Diff_Function\UP_DOWN\GO\NL-VS-L1.P.html#gene755) | anion homeostasis | 1 (0.08%) | 4 (0.12%) | 0.839991 | 0.999992 |
| 756 | [GO:0060429](file:///E:\2018-7-3%E7%81%AB%E9%BE%99%E6%9E%9C%E8%BD%AC%E5%BD%95%E7%BB%84%E6%B5%8B%E5%BA%8F\%E5%AE%8C%E6%95%B4%E7%89%88%E6%95%B0%E6%8D%AE\GDR3855-Hylocereus_undulatus_Britt-12-RNAseq_result\4_Function\2_Group_Diff_Function\UP_DOWN\GO\NL-VS-L1.P.html#gene756) | epithelium development | 1 (0.08%) | 4 (0.12%) | 0.839991 | 0.999992 |
| 757 | [GO:0098813](file:///E:\2018-7-3%E7%81%AB%E9%BE%99%E6%9E%9C%E8%BD%AC%E5%BD%95%E7%BB%84%E6%B5%8B%E5%BA%8F\%E5%AE%8C%E6%95%B4%E7%89%88%E6%95%B0%E6%8D%AE\GDR3855-Hylocereus_undulatus_Britt-12-RNAseq_result\4_Function\2_Group_Diff_Function\UP_DOWN\GO\NL-VS-L1.P.html#gene757) | nuclear chromosome segregation | 1 (0.08%) | 4 (0.12%) | 0.839991 | 0.999992 |
| 758 | [GO:0009451](file:///E:\2018-7-3%E7%81%AB%E9%BE%99%E6%9E%9C%E8%BD%AC%E5%BD%95%E7%BB%84%E6%B5%8B%E5%BA%8F\%E5%AE%8C%E6%95%B4%E7%89%88%E6%95%B0%E6%8D%AE\GDR3855-Hylocereus_undulatus_Britt-12-RNAseq_result\4_Function\2_Group_Diff_Function\UP_DOWN\GO\NL-VS-L1.P.html#gene758) | RNA modification | 6 (0.49%) | 21 (0.63%) | 0.842793 | 0.999992 |
| 759 | [GO:0016311](file:///E:\2018-7-3%E7%81%AB%E9%BE%99%E6%9E%9C%E8%BD%AC%E5%BD%95%E7%BB%84%E6%B5%8B%E5%BA%8F\%E5%AE%8C%E6%95%B4%E7%89%88%E6%95%B0%E6%8D%AE\GDR3855-Hylocereus_undulatus_Britt-12-RNAseq_result\4_Function\2_Group_Diff_Function\UP_DOWN\GO\NL-VS-L1.P.html#gene759) | dephosphorylation | 6 (0.49%) | 21 (0.63%) | 0.842793 | 0.999992 |
| 760 | [GO:0007165](file:///E:\2018-7-3%E7%81%AB%E9%BE%99%E6%9E%9C%E8%BD%AC%E5%BD%95%E7%BB%84%E6%B5%8B%E5%BA%8F\%E5%AE%8C%E6%95%B4%E7%89%88%E6%95%B0%E6%8D%AE\GDR3855-Hylocereus_undulatus_Britt-12-RNAseq_result\4_Function\2_Group_Diff_Function\UP_DOWN\GO\NL-VS-L1.P.html#gene760) | signal transduction | 49 (3.97%) | 148 (4.41%) | 0.847148 | 0.999992 |
| 761 | [GO:0023052](file:///E:\2018-7-3%E7%81%AB%E9%BE%99%E6%9E%9C%E8%BD%AC%E5%BD%95%E7%BB%84%E6%B5%8B%E5%BA%8F\%E5%AE%8C%E6%95%B4%E7%89%88%E6%95%B0%E6%8D%AE\GDR3855-Hylocereus_undulatus_Britt-12-RNAseq_result\4_Function\2_Group_Diff_Function\UP_DOWN\GO\NL-VS-L1.P.html#gene761) | signaling | 49 (3.97%) | 148 (4.41%) | 0.847148 | 0.999992 |
| 762 | [GO:0044700](file:///E:\2018-7-3%E7%81%AB%E9%BE%99%E6%9E%9C%E8%BD%AC%E5%BD%95%E7%BB%84%E6%B5%8B%E5%BA%8F\%E5%AE%8C%E6%95%B4%E7%89%88%E6%95%B0%E6%8D%AE\GDR3855-Hylocereus_undulatus_Britt-12-RNAseq_result\4_Function\2_Group_Diff_Function\UP_DOWN\GO\NL-VS-L1.P.html#gene762) | single organism signaling | 49 (3.97%) | 148 (4.41%) | 0.847148 | 0.999992 |
| 763 | [GO:0071702](file:///E:\2018-7-3%E7%81%AB%E9%BE%99%E6%9E%9C%E8%BD%AC%E5%BD%95%E7%BB%84%E6%B5%8B%E5%BA%8F\%E5%AE%8C%E6%95%B4%E7%89%88%E6%95%B0%E6%8D%AE\GDR3855-Hylocereus_undulatus_Britt-12-RNAseq_result\4_Function\2_Group_Diff_Function\UP_DOWN\GO\NL-VS-L1.P.html#gene763) | organic substance transport | 41 (3.32%) | 125 (3.72%) | 0.847460 | 0.999992 |
| 764 | [GO:0044767](file:///E:\2018-7-3%E7%81%AB%E9%BE%99%E6%9E%9C%E8%BD%AC%E5%BD%95%E7%BB%84%E6%B5%8B%E5%BA%8F\%E5%AE%8C%E6%95%B4%E7%89%88%E6%95%B0%E6%8D%AE\GDR3855-Hylocereus_undulatus_Britt-12-RNAseq_result\4_Function\2_Group_Diff_Function\UP_DOWN\GO\NL-VS-L1.P.html#gene764) | single-organism developmental process | 50 (4.05%) | 151 (4.5%) | 0.849031 | 0.999992 |
| 765 | [GO:0006470](file:///E:\2018-7-3%E7%81%AB%E9%BE%99%E6%9E%9C%E8%BD%AC%E5%BD%95%E7%BB%84%E6%B5%8B%E5%BA%8F\%E5%AE%8C%E6%95%B4%E7%89%88%E6%95%B0%E6%8D%AE\GDR3855-Hylocereus_undulatus_Britt-12-RNAseq_result\4_Function\2_Group_Diff_Function\UP_DOWN\GO\NL-VS-L1.P.html#gene765) | protein dephosphorylation | 5 (0.41%) | 18 (0.54%) | 0.850305 | 0.999992 |
| 766 | [GO:0019318](file:///E:\2018-7-3%E7%81%AB%E9%BE%99%E6%9E%9C%E8%BD%AC%E5%BD%95%E7%BB%84%E6%B5%8B%E5%BA%8F\%E5%AE%8C%E6%95%B4%E7%89%88%E6%95%B0%E6%8D%AE\GDR3855-Hylocereus_undulatus_Britt-12-RNAseq_result\4_Function\2_Group_Diff_Function\UP_DOWN\GO\NL-VS-L1.P.html#gene766) | hexose metabolic process | 12 (0.97%) | 40 (1.19%) | 0.854563 | 0.999992 |
| 767 | [GO:0009725](file:///E:\2018-7-3%E7%81%AB%E9%BE%99%E6%9E%9C%E8%BD%AC%E5%BD%95%E7%BB%84%E6%B5%8B%E5%BA%8F\%E5%AE%8C%E6%95%B4%E7%89%88%E6%95%B0%E6%8D%AE\GDR3855-Hylocereus_undulatus_Britt-12-RNAseq_result\4_Function\2_Group_Diff_Function\UP_DOWN\GO\NL-VS-L1.P.html#gene767) | response to hormone | 22 (1.78%) | 70 (2.08%) | 0.854940 | 0.999992 |
| 768 | [GO:0000280](file:///E:\2018-7-3%E7%81%AB%E9%BE%99%E6%9E%9C%E8%BD%AC%E5%BD%95%E7%BB%84%E6%B5%8B%E5%BA%8F\%E5%AE%8C%E6%95%B4%E7%89%88%E6%95%B0%E6%8D%AE\GDR3855-Hylocereus_undulatus_Britt-12-RNAseq_result\4_Function\2_Group_Diff_Function\UP_DOWN\GO\NL-VS-L1.P.html#gene768) | nuclear division | 2 (0.16%) | 8 (0.24%) | 0.855463 | 0.999992 |
| 769 | [GO:0009112](file:///E:\2018-7-3%E7%81%AB%E9%BE%99%E6%9E%9C%E8%BD%AC%E5%BD%95%E7%BB%84%E6%B5%8B%E5%BA%8F\%E5%AE%8C%E6%95%B4%E7%89%88%E6%95%B0%E6%8D%AE\GDR3855-Hylocereus_undulatus_Britt-12-RNAseq_result\4_Function\2_Group_Diff_Function\UP_DOWN\GO\NL-VS-L1.P.html#gene769) | nucleobase metabolic process | 2 (0.16%) | 8 (0.24%) | 0.855463 | 0.999992 |
| 770 | [GO:0010053](file:///E:\2018-7-3%E7%81%AB%E9%BE%99%E6%9E%9C%E8%BD%AC%E5%BD%95%E7%BB%84%E6%B5%8B%E5%BA%8F\%E5%AE%8C%E6%95%B4%E7%89%88%E6%95%B0%E6%8D%AE\GDR3855-Hylocereus_undulatus_Britt-12-RNAseq_result\4_Function\2_Group_Diff_Function\UP_DOWN\GO\NL-VS-L1.P.html#gene770) | root epidermal cell differentiation | 2 (0.16%) | 8 (0.24%) | 0.855463 | 0.999992 |
| 771 | [GO:0042440](file:///E:\2018-7-3%E7%81%AB%E9%BE%99%E6%9E%9C%E8%BD%AC%E5%BD%95%E7%BB%84%E6%B5%8B%E5%BA%8F\%E5%AE%8C%E6%95%B4%E7%89%88%E6%95%B0%E6%8D%AE\GDR3855-Hylocereus_undulatus_Britt-12-RNAseq_result\4_Function\2_Group_Diff_Function\UP_DOWN\GO\NL-VS-L1.P.html#gene771) | pigment metabolic process | 2 (0.16%) | 8 (0.24%) | 0.855463 | 0.999992 |
| 772 | [GO:0046148](file:///E:\2018-7-3%E7%81%AB%E9%BE%99%E6%9E%9C%E8%BD%AC%E5%BD%95%E7%BB%84%E6%B5%8B%E5%BA%8F\%E5%AE%8C%E6%95%B4%E7%89%88%E6%95%B0%E6%8D%AE\GDR3855-Hylocereus_undulatus_Britt-12-RNAseq_result\4_Function\2_Group_Diff_Function\UP_DOWN\GO\NL-VS-L1.P.html#gene772) | pigment biosynthetic process | 2 (0.16%) | 8 (0.24%) | 0.855463 | 0.999992 |
| 773 | [GO:0090627](file:///E:\2018-7-3%E7%81%AB%E9%BE%99%E6%9E%9C%E8%BD%AC%E5%BD%95%E7%BB%84%E6%B5%8B%E5%BA%8F\%E5%AE%8C%E6%95%B4%E7%89%88%E6%95%B0%E6%8D%AE\GDR3855-Hylocereus_undulatus_Britt-12-RNAseq_result\4_Function\2_Group_Diff_Function\UP_DOWN\GO\NL-VS-L1.P.html#gene773) | plant epidermal cell differentiation | 2 (0.16%) | 8 (0.24%) | 0.855463 | 0.999992 |
| 774 | [GO:0009888](file:///E:\2018-7-3%E7%81%AB%E9%BE%99%E6%9E%9C%E8%BD%AC%E5%BD%95%E7%BB%84%E6%B5%8B%E5%BA%8F\%E5%AE%8C%E6%95%B4%E7%89%88%E6%95%B0%E6%8D%AE\GDR3855-Hylocereus_undulatus_Britt-12-RNAseq_result\4_Function\2_Group_Diff_Function\UP_DOWN\GO\NL-VS-L1.P.html#gene774) | tissue development | 11 (0.89%) | 37 (1.1%) | 0.856096 | 0.999992 |
| 775 | [GO:0045184](file:///E:\2018-7-3%E7%81%AB%E9%BE%99%E6%9E%9C%E8%BD%AC%E5%BD%95%E7%BB%84%E6%B5%8B%E5%BA%8F\%E5%AE%8C%E6%95%B4%E7%89%88%E6%95%B0%E6%8D%AE\GDR3855-Hylocereus_undulatus_Britt-12-RNAseq_result\4_Function\2_Group_Diff_Function\UP_DOWN\GO\NL-VS-L1.P.html#gene775) | establishment of protein localization | 38 (3.08%) | 117 (3.48%) | 0.858039 | 0.999992 |
| 776 | [GO:0043604](file:///E:\2018-7-3%E7%81%AB%E9%BE%99%E6%9E%9C%E8%BD%AC%E5%BD%95%E7%BB%84%E6%B5%8B%E5%BA%8F\%E5%AE%8C%E6%95%B4%E7%89%88%E6%95%B0%E6%8D%AE\GDR3855-Hylocereus_undulatus_Britt-12-RNAseq_result\4_Function\2_Group_Diff_Function\UP_DOWN\GO\NL-VS-L1.P.html#gene776) | amide biosynthetic process | 26 (2.11%) | 82 (2.44%) | 0.858649 | 0.999992 |
| 777 | [GO:0016042](file:///E:\2018-7-3%E7%81%AB%E9%BE%99%E6%9E%9C%E8%BD%AC%E5%BD%95%E7%BB%84%E6%B5%8B%E5%BA%8F\%E5%AE%8C%E6%95%B4%E7%89%88%E6%95%B0%E6%8D%AE\GDR3855-Hylocereus_undulatus_Britt-12-RNAseq_result\4_Function\2_Group_Diff_Function\UP_DOWN\GO\NL-VS-L1.P.html#gene777) | lipid catabolic process | 4 (0.32%) | 15 (0.45%) | 0.860910 | 0.999992 |
| 778 | [GO:0044242](file:///E:\2018-7-3%E7%81%AB%E9%BE%99%E6%9E%9C%E8%BD%AC%E5%BD%95%E7%BB%84%E6%B5%8B%E5%BA%8F\%E5%AE%8C%E6%95%B4%E7%89%88%E6%95%B0%E6%8D%AE\GDR3855-Hylocereus_undulatus_Britt-12-RNAseq_result\4_Function\2_Group_Diff_Function\UP_DOWN\GO\NL-VS-L1.P.html#gene778) | cellular lipid catabolic process | 4 (0.32%) | 15 (0.45%) | 0.860910 | 0.999992 |
| 779 | [GO:0048523](file:///E:\2018-7-3%E7%81%AB%E9%BE%99%E6%9E%9C%E8%BD%AC%E5%BD%95%E7%BB%84%E6%B5%8B%E5%BA%8F\%E5%AE%8C%E6%95%B4%E7%89%88%E6%95%B0%E6%8D%AE\GDR3855-Hylocereus_undulatus_Britt-12-RNAseq_result\4_Function\2_Group_Diff_Function\UP_DOWN\GO\NL-VS-L1.P.html#gene779) | negative regulation of cellular process | 4 (0.32%) | 15 (0.45%) | 0.860910 | 0.999992 |
| 780 | [GO:0098771](file:///E:\2018-7-3%E7%81%AB%E9%BE%99%E6%9E%9C%E8%BD%AC%E5%BD%95%E7%BB%84%E6%B5%8B%E5%BA%8F\%E5%AE%8C%E6%95%B4%E7%89%88%E6%95%B0%E6%8D%AE\GDR3855-Hylocereus_undulatus_Britt-12-RNAseq_result\4_Function\2_Group_Diff_Function\UP_DOWN\GO\NL-VS-L1.P.html#gene780) | inorganic ion homeostasis | 4 (0.32%) | 15 (0.45%) | 0.860910 | 0.999992 |
| 781 | [GO:0007049](file:///E:\2018-7-3%E7%81%AB%E9%BE%99%E6%9E%9C%E8%BD%AC%E5%BD%95%E7%BB%84%E6%B5%8B%E5%BA%8F\%E5%AE%8C%E6%95%B4%E7%89%88%E6%95%B0%E6%8D%AE\GDR3855-Hylocereus_undulatus_Britt-12-RNAseq_result\4_Function\2_Group_Diff_Function\UP_DOWN\GO\NL-VS-L1.P.html#gene781) | cell cycle | 8 (0.65%) | 28 (0.83%) | 0.864649 | 0.999992 |
| 782 | [GO:0033013](file:///E:\2018-7-3%E7%81%AB%E9%BE%99%E6%9E%9C%E8%BD%AC%E5%BD%95%E7%BB%84%E6%B5%8B%E5%BA%8F\%E5%AE%8C%E6%95%B4%E7%89%88%E6%95%B0%E6%8D%AE\GDR3855-Hylocereus_undulatus_Britt-12-RNAseq_result\4_Function\2_Group_Diff_Function\UP_DOWN\GO\NL-VS-L1.P.html#gene782) | tetrapyrrole metabolic process | 6 (0.49%) | 22 (0.65%) | 0.875725 | 0.999992 |
| 783 | [GO:0065007](file:///E:\2018-7-3%E7%81%AB%E9%BE%99%E6%9E%9C%E8%BD%AC%E5%BD%95%E7%BB%84%E6%B5%8B%E5%BA%8F\%E5%AE%8C%E6%95%B4%E7%89%88%E6%95%B0%E6%8D%AE\GDR3855-Hylocereus_undulatus_Britt-12-RNAseq_result\4_Function\2_Group_Diff_Function\UP_DOWN\GO\NL-VS-L1.P.html#gene783) | biological regulation | 137 (11.1%) | 400 (11.91%) | 0.876146 | 0.999992 |
| 784 | [GO:0006220](file:///E:\2018-7-3%E7%81%AB%E9%BE%99%E6%9E%9C%E8%BD%AC%E5%BD%95%E7%BB%84%E6%B5%8B%E5%BA%8F\%E5%AE%8C%E6%95%B4%E7%89%88%E6%95%B0%E6%8D%AE\GDR3855-Hylocereus_undulatus_Britt-12-RNAseq_result\4_Function\2_Group_Diff_Function\UP_DOWN\GO\NL-VS-L1.P.html#gene784) | pyrimidine nucleotide metabolic process | 3 (0.24%) | 12 (0.36%) | 0.876231 | 0.999992 |
| 785 | [GO:0009606](file:///E:\2018-7-3%E7%81%AB%E9%BE%99%E6%9E%9C%E8%BD%AC%E5%BD%95%E7%BB%84%E6%B5%8B%E5%BA%8F\%E5%AE%8C%E6%95%B4%E7%89%88%E6%95%B0%E6%8D%AE\GDR3855-Hylocereus_undulatus_Britt-12-RNAseq_result\4_Function\2_Group_Diff_Function\UP_DOWN\GO\NL-VS-L1.P.html#gene785) | tropism | 3 (0.24%) | 12 (0.36%) | 0.876231 | 0.999992 |
| 786 | [GO:0042278](file:///E:\2018-7-3%E7%81%AB%E9%BE%99%E6%9E%9C%E8%BD%AC%E5%BD%95%E7%BB%84%E6%B5%8B%E5%BA%8F\%E5%AE%8C%E6%95%B4%E7%89%88%E6%95%B0%E6%8D%AE\GDR3855-Hylocereus_undulatus_Britt-12-RNAseq_result\4_Function\2_Group_Diff_Function\UP_DOWN\GO\NL-VS-L1.P.html#gene786) | purine nucleoside metabolic process | 5 (0.41%) | 19 (0.57%) | 0.883981 | 0.999992 |
| 787 | [GO:0042451](file:///E:\2018-7-3%E7%81%AB%E9%BE%99%E6%9E%9C%E8%BD%AC%E5%BD%95%E7%BB%84%E6%B5%8B%E5%BA%8F\%E5%AE%8C%E6%95%B4%E7%89%88%E6%95%B0%E6%8D%AE\GDR3855-Hylocereus_undulatus_Britt-12-RNAseq_result\4_Function\2_Group_Diff_Function\UP_DOWN\GO\NL-VS-L1.P.html#gene787) | purine nucleoside biosynthetic process | 5 (0.41%) | 19 (0.57%) | 0.883981 | 0.999992 |
| 788 | [GO:0046128](file:///E:\2018-7-3%E7%81%AB%E9%BE%99%E6%9E%9C%E8%BD%AC%E5%BD%95%E7%BB%84%E6%B5%8B%E5%BA%8F\%E5%AE%8C%E6%95%B4%E7%89%88%E6%95%B0%E6%8D%AE\GDR3855-Hylocereus_undulatus_Britt-12-RNAseq_result\4_Function\2_Group_Diff_Function\UP_DOWN\GO\NL-VS-L1.P.html#gene788) | purine ribonucleoside metabolic process | 5 (0.41%) | 19 (0.57%) | 0.883981 | 0.999992 |
| 789 | [GO:0046129](file:///E:\2018-7-3%E7%81%AB%E9%BE%99%E6%9E%9C%E8%BD%AC%E5%BD%95%E7%BB%84%E6%B5%8B%E5%BA%8F\%E5%AE%8C%E6%95%B4%E7%89%88%E6%95%B0%E6%8D%AE\GDR3855-Hylocereus_undulatus_Britt-12-RNAseq_result\4_Function\2_Group_Diff_Function\UP_DOWN\GO\NL-VS-L1.P.html#gene789) | purine ribonucleoside biosynthetic process | 5 (0.41%) | 19 (0.57%) | 0.883981 | 0.999992 |
| 790 | [GO:0006793](file:///E:\2018-7-3%E7%81%AB%E9%BE%99%E6%9E%9C%E8%BD%AC%E5%BD%95%E7%BB%84%E6%B5%8B%E5%BA%8F\%E5%AE%8C%E6%95%B4%E7%89%88%E6%95%B0%E6%8D%AE\GDR3855-Hylocereus_undulatus_Britt-12-RNAseq_result\4_Function\2_Group_Diff_Function\UP_DOWN\GO\NL-VS-L1.P.html#gene790) | phosphorus metabolic process | 236 (19.12%) | 678 (20.18%) | 0.887220 | 0.999992 |
| 791 | [GO:0003006](file:///E:\2018-7-3%E7%81%AB%E9%BE%99%E6%9E%9C%E8%BD%AC%E5%BD%95%E7%BB%84%E6%B5%8B%E5%BA%8F\%E5%AE%8C%E6%95%B4%E7%89%88%E6%95%B0%E6%8D%AE\GDR3855-Hylocereus_undulatus_Britt-12-RNAseq_result\4_Function\2_Group_Diff_Function\UP_DOWN\GO\NL-VS-L1.P.html#gene791) | developmental process involved in reproduction | 24 (1.94%) | 78 (2.32%) | 0.890850 | 0.999992 |
| 792 | [GO:0006970](file:///E:\2018-7-3%E7%81%AB%E9%BE%99%E6%9E%9C%E8%BD%AC%E5%BD%95%E7%BB%84%E6%B5%8B%E5%BA%8F\%E5%AE%8C%E6%95%B4%E7%89%88%E6%95%B0%E6%8D%AE\GDR3855-Hylocereus_undulatus_Britt-12-RNAseq_result\4_Function\2_Group_Diff_Function\UP_DOWN\GO\NL-VS-L1.P.html#gene792) | response to osmotic stress | 19 (1.54%) | 63 (1.88%) | 0.891059 | 0.999992 |
| 793 | [GO:0080090](file:///E:\2018-7-3%E7%81%AB%E9%BE%99%E6%9E%9C%E8%BD%AC%E5%BD%95%E7%BB%84%E6%B5%8B%E5%BA%8F\%E5%AE%8C%E6%95%B4%E7%89%88%E6%95%B0%E6%8D%AE\GDR3855-Hylocereus_undulatus_Britt-12-RNAseq_result\4_Function\2_Group_Diff_Function\UP_DOWN\GO\NL-VS-L1.P.html#gene793) | regulation of primary metabolic process | 18 (1.46%) | 60 (1.79%) | 0.891503 | 0.999992 |
| 794 | [GO:0006796](file:///E:\2018-7-3%E7%81%AB%E9%BE%99%E6%9E%9C%E8%BD%AC%E5%BD%95%E7%BB%84%E6%B5%8B%E5%BA%8F\%E5%AE%8C%E6%95%B4%E7%89%88%E6%95%B0%E6%8D%AE\GDR3855-Hylocereus_undulatus_Britt-12-RNAseq_result\4_Function\2_Group_Diff_Function\UP_DOWN\GO\NL-VS-L1.P.html#gene794) | phosphate-containing compound metabolic process | 231 (18.72%) | 665 (19.8%) | 0.892704 | 0.999992 |
| 795 | [GO:0009791](file:///E:\2018-7-3%E7%81%AB%E9%BE%99%E6%9E%9C%E8%BD%AC%E5%BD%95%E7%BB%84%E6%B5%8B%E5%BA%8F\%E5%AE%8C%E6%95%B4%E7%89%88%E6%95%B0%E6%8D%AE\GDR3855-Hylocereus_undulatus_Britt-12-RNAseq_result\4_Function\2_Group_Diff_Function\UP_DOWN\GO\NL-VS-L1.P.html#gene795) | post-embryonic development | 16 (1.3%) | 54 (1.61%) | 0.892958 | 0.999992 |
| 796 | [GO:0072593](file:///E:\2018-7-3%E7%81%AB%E9%BE%99%E6%9E%9C%E8%BD%AC%E5%BD%95%E7%BB%84%E6%B5%8B%E5%BA%8F\%E5%AE%8C%E6%95%B4%E7%89%88%E6%95%B0%E6%8D%AE\GDR3855-Hylocereus_undulatus_Britt-12-RNAseq_result\4_Function\2_Group_Diff_Function\UP_DOWN\GO\NL-VS-L1.P.html#gene796) | reactive oxygen species metabolic process | 4 (0.32%) | 16 (0.48%) | 0.894961 | 0.999992 |
| 797 | [GO:0006544](file:///E:\2018-7-3%E7%81%AB%E9%BE%99%E6%9E%9C%E8%BD%AC%E5%BD%95%E7%BB%84%E6%B5%8B%E5%BA%8F\%E5%AE%8C%E6%95%B4%E7%89%88%E6%95%B0%E6%8D%AE\GDR3855-Hylocereus_undulatus_Britt-12-RNAseq_result\4_Function\2_Group_Diff_Function\UP_DOWN\GO\NL-VS-L1.P.html#gene797) | glycine metabolic process | 1 (0.08%) | 5 (0.15%) | 0.898844 | 0.999992 |
| 798 | [GO:0006839](file:///E:\2018-7-3%E7%81%AB%E9%BE%99%E6%9E%9C%E8%BD%AC%E5%BD%95%E7%BB%84%E6%B5%8B%E5%BA%8F\%E5%AE%8C%E6%95%B4%E7%89%88%E6%95%B0%E6%8D%AE\GDR3855-Hylocereus_undulatus_Britt-12-RNAseq_result\4_Function\2_Group_Diff_Function\UP_DOWN\GO\NL-VS-L1.P.html#gene798) | mitochondrial transport | 1 (0.08%) | 5 (0.15%) | 0.898844 | 0.999992 |
| 799 | [GO:0007126](file:///E:\2018-7-3%E7%81%AB%E9%BE%99%E6%9E%9C%E8%BD%AC%E5%BD%95%E7%BB%84%E6%B5%8B%E5%BA%8F\%E5%AE%8C%E6%95%B4%E7%89%88%E6%95%B0%E6%8D%AE\GDR3855-Hylocereus_undulatus_Britt-12-RNAseq_result\4_Function\2_Group_Diff_Function\UP_DOWN\GO\NL-VS-L1.P.html#gene799) | meiotic nuclear division | 1 (0.08%) | 5 (0.15%) | 0.898844 | 0.999992 |
| 800 | [GO:0007127](file:///E:\2018-7-3%E7%81%AB%E9%BE%99%E6%9E%9C%E8%BD%AC%E5%BD%95%E7%BB%84%E6%B5%8B%E5%BA%8F\%E5%AE%8C%E6%95%B4%E7%89%88%E6%95%B0%E6%8D%AE\GDR3855-Hylocereus_undulatus_Britt-12-RNAseq_result\4_Function\2_Group_Diff_Function\UP_DOWN\GO\NL-VS-L1.P.html#gene800) | meiosis I | 1 (0.08%) | 5 (0.15%) | 0.898844 | 0.999992 |
| 801 | [GO:0007186](file:///E:\2018-7-3%E7%81%AB%E9%BE%99%E6%9E%9C%E8%BD%AC%E5%BD%95%E7%BB%84%E6%B5%8B%E5%BA%8F\%E5%AE%8C%E6%95%B4%E7%89%88%E6%95%B0%E6%8D%AE\GDR3855-Hylocereus_undulatus_Britt-12-RNAseq_result\4_Function\2_Group_Diff_Function\UP_DOWN\GO\NL-VS-L1.P.html#gene801) | G-protein coupled receptor signaling pathway | 1 (0.08%) | 5 (0.15%) | 0.898844 | 0.999992 |
| 802 | [GO:0009826](file:///E:\2018-7-3%E7%81%AB%E9%BE%99%E6%9E%9C%E8%BD%AC%E5%BD%95%E7%BB%84%E6%B5%8B%E5%BA%8F\%E5%AE%8C%E6%95%B4%E7%89%88%E6%95%B0%E6%8D%AE\GDR3855-Hylocereus_undulatus_Britt-12-RNAseq_result\4_Function\2_Group_Diff_Function\UP_DOWN\GO\NL-VS-L1.P.html#gene802) | unidimensional cell growth | 1 (0.08%) | 5 (0.15%) | 0.898844 | 0.999992 |
| 803 | [GO:0016143](file:///E:\2018-7-3%E7%81%AB%E9%BE%99%E6%9E%9C%E8%BD%AC%E5%BD%95%E7%BB%84%E6%B5%8B%E5%BA%8F\%E5%AE%8C%E6%95%B4%E7%89%88%E6%95%B0%E6%8D%AE\GDR3855-Hylocereus_undulatus_Britt-12-RNAseq_result\4_Function\2_Group_Diff_Function\UP_DOWN\GO\NL-VS-L1.P.html#gene803) | S-glycoside metabolic process | 1 (0.08%) | 5 (0.15%) | 0.898844 | 0.999992 |
| 804 | [GO:0016144](file:///E:\2018-7-3%E7%81%AB%E9%BE%99%E6%9E%9C%E8%BD%AC%E5%BD%95%E7%BB%84%E6%B5%8B%E5%BA%8F\%E5%AE%8C%E6%95%B4%E7%89%88%E6%95%B0%E6%8D%AE\GDR3855-Hylocereus_undulatus_Britt-12-RNAseq_result\4_Function\2_Group_Diff_Function\UP_DOWN\GO\NL-VS-L1.P.html#gene804) | S-glycoside biosynthetic process | 1 (0.08%) | 5 (0.15%) | 0.898844 | 0.999992 |
| 805 | [GO:0019757](file:///E:\2018-7-3%E7%81%AB%E9%BE%99%E6%9E%9C%E8%BD%AC%E5%BD%95%E7%BB%84%E6%B5%8B%E5%BA%8F\%E5%AE%8C%E6%95%B4%E7%89%88%E6%95%B0%E6%8D%AE\GDR3855-Hylocereus_undulatus_Britt-12-RNAseq_result\4_Function\2_Group_Diff_Function\UP_DOWN\GO\NL-VS-L1.P.html#gene805) | glycosinolate metabolic process | 1 (0.08%) | 5 (0.15%) | 0.898844 | 0.999992 |
| 806 | [GO:0019758](file:///E:\2018-7-3%E7%81%AB%E9%BE%99%E6%9E%9C%E8%BD%AC%E5%BD%95%E7%BB%84%E6%B5%8B%E5%BA%8F\%E5%AE%8C%E6%95%B4%E7%89%88%E6%95%B0%E6%8D%AE\GDR3855-Hylocereus_undulatus_Britt-12-RNAseq_result\4_Function\2_Group_Diff_Function\UP_DOWN\GO\NL-VS-L1.P.html#gene806) | glycosinolate biosynthetic process | 1 (0.08%) | 5 (0.15%) | 0.898844 | 0.999992 |
| 807 | [GO:0032392](file:///E:\2018-7-3%E7%81%AB%E9%BE%99%E6%9E%9C%E8%BD%AC%E5%BD%95%E7%BB%84%E6%B5%8B%E5%BA%8F\%E5%AE%8C%E6%95%B4%E7%89%88%E6%95%B0%E6%8D%AE\GDR3855-Hylocereus_undulatus_Britt-12-RNAseq_result\4_Function\2_Group_Diff_Function\UP_DOWN\GO\NL-VS-L1.P.html#gene807) | DNA geometric change | 1 (0.08%) | 5 (0.15%) | 0.898844 | 0.999992 |
| 808 | [GO:0046496](file:///E:\2018-7-3%E7%81%AB%E9%BE%99%E6%9E%9C%E8%BD%AC%E5%BD%95%E7%BB%84%E6%B5%8B%E5%BA%8F\%E5%AE%8C%E6%95%B4%E7%89%88%E6%95%B0%E6%8D%AE\GDR3855-Hylocereus_undulatus_Britt-12-RNAseq_result\4_Function\2_Group_Diff_Function\UP_DOWN\GO\NL-VS-L1.P.html#gene808) | nicotinamide nucleotide metabolic process | 1 (0.08%) | 5 (0.15%) | 0.898844 | 0.999992 |
| 809 | [GO:0048193](file:///E:\2018-7-3%E7%81%AB%E9%BE%99%E6%9E%9C%E8%BD%AC%E5%BD%95%E7%BB%84%E6%B5%8B%E5%BA%8F\%E5%AE%8C%E6%95%B4%E7%89%88%E6%95%B0%E6%8D%AE\GDR3855-Hylocereus_undulatus_Britt-12-RNAseq_result\4_Function\2_Group_Diff_Function\UP_DOWN\GO\NL-VS-L1.P.html#gene809) | Golgi vesicle transport | 1 (0.08%) | 5 (0.15%) | 0.898844 | 0.999992 |
| 810 | [GO:0060560](file:///E:\2018-7-3%E7%81%AB%E9%BE%99%E6%9E%9C%E8%BD%AC%E5%BD%95%E7%BB%84%E6%B5%8B%E5%BA%8F\%E5%AE%8C%E6%95%B4%E7%89%88%E6%95%B0%E6%8D%AE\GDR3855-Hylocereus_undulatus_Britt-12-RNAseq_result\4_Function\2_Group_Diff_Function\UP_DOWN\GO\NL-VS-L1.P.html#gene810) | developmental growth involved in morphogenesis | 1 (0.08%) | 5 (0.15%) | 0.898844 | 0.999992 |
| 811 | [GO:0000375](file:///E:\2018-7-3%E7%81%AB%E9%BE%99%E6%9E%9C%E8%BD%AC%E5%BD%95%E7%BB%84%E6%B5%8B%E5%BA%8F\%E5%AE%8C%E6%95%B4%E7%89%88%E6%95%B0%E6%8D%AE\GDR3855-Hylocereus_undulatus_Britt-12-RNAseq_result\4_Function\2_Group_Diff_Function\UP_DOWN\GO\NL-VS-L1.P.html#gene811) | RNA splicing, via transesterification reactions | 2 (0.16%) | 9 (0.27%) | 0.899251 | 0.999992 |
| 812 | [GO:0000377](file:///E:\2018-7-3%E7%81%AB%E9%BE%99%E6%9E%9C%E8%BD%AC%E5%BD%95%E7%BB%84%E6%B5%8B%E5%BA%8F\%E5%AE%8C%E6%95%B4%E7%89%88%E6%95%B0%E6%8D%AE\GDR3855-Hylocereus_undulatus_Britt-12-RNAseq_result\4_Function\2_Group_Diff_Function\UP_DOWN\GO\NL-VS-L1.P.html#gene812) | RNA splicing, via transesterification reactions with bulged adenosine as nucleophile | 2 (0.16%) | 9 (0.27%) | 0.899251 | 0.999992 |
| 813 | [GO:0009404](file:///E:\2018-7-3%E7%81%AB%E9%BE%99%E6%9E%9C%E8%BD%AC%E5%BD%95%E7%BB%84%E6%B5%8B%E5%BA%8F\%E5%AE%8C%E6%95%B4%E7%89%88%E6%95%B0%E6%8D%AE\GDR3855-Hylocereus_undulatus_Britt-12-RNAseq_result\4_Function\2_Group_Diff_Function\UP_DOWN\GO\NL-VS-L1.P.html#gene813) | toxin metabolic process | 2 (0.16%) | 9 (0.27%) | 0.899251 | 0.999992 |
| 814 | [GO:0010015](file:///E:\2018-7-3%E7%81%AB%E9%BE%99%E6%9E%9C%E8%BD%AC%E5%BD%95%E7%BB%84%E6%B5%8B%E5%BA%8F\%E5%AE%8C%E6%95%B4%E7%89%88%E6%95%B0%E6%8D%AE\GDR3855-Hylocereus_undulatus_Britt-12-RNAseq_result\4_Function\2_Group_Diff_Function\UP_DOWN\GO\NL-VS-L1.P.html#gene814) | root morphogenesis | 2 (0.16%) | 9 (0.27%) | 0.899251 | 0.999992 |
| 815 | [GO:0016109](file:///E:\2018-7-3%E7%81%AB%E9%BE%99%E6%9E%9C%E8%BD%AC%E5%BD%95%E7%BB%84%E6%B5%8B%E5%BA%8F\%E5%AE%8C%E6%95%B4%E7%89%88%E6%95%B0%E6%8D%AE\GDR3855-Hylocereus_undulatus_Britt-12-RNAseq_result\4_Function\2_Group_Diff_Function\UP_DOWN\GO\NL-VS-L1.P.html#gene815) | tetraterpenoid biosynthetic process | 2 (0.16%) | 9 (0.27%) | 0.899251 | 0.999992 |
| 816 | [GO:0016114](file:///E:\2018-7-3%E7%81%AB%E9%BE%99%E6%9E%9C%E8%BD%AC%E5%BD%95%E7%BB%84%E6%B5%8B%E5%BA%8F\%E5%AE%8C%E6%95%B4%E7%89%88%E6%95%B0%E6%8D%AE\GDR3855-Hylocereus_undulatus_Britt-12-RNAseq_result\4_Function\2_Group_Diff_Function\UP_DOWN\GO\NL-VS-L1.P.html#gene816) | terpenoid biosynthetic process | 2 (0.16%) | 9 (0.27%) | 0.899251 | 0.999992 |
| 817 | [GO:0044036](file:///E:\2018-7-3%E7%81%AB%E9%BE%99%E6%9E%9C%E8%BD%AC%E5%BD%95%E7%BB%84%E6%B5%8B%E5%BA%8F\%E5%AE%8C%E6%95%B4%E7%89%88%E6%95%B0%E6%8D%AE\GDR3855-Hylocereus_undulatus_Britt-12-RNAseq_result\4_Function\2_Group_Diff_Function\UP_DOWN\GO\NL-VS-L1.P.html#gene817) | cell wall macromolecule metabolic process | 2 (0.16%) | 9 (0.27%) | 0.899251 | 0.999992 |
| 818 | [GO:0044710](file:///E:\2018-7-3%E7%81%AB%E9%BE%99%E6%9E%9C%E8%BD%AC%E5%BD%95%E7%BB%84%E6%B5%8B%E5%BA%8F\%E5%AE%8C%E6%95%B4%E7%89%88%E6%95%B0%E6%8D%AE\GDR3855-Hylocereus_undulatus_Britt-12-RNAseq_result\4_Function\2_Group_Diff_Function\UP_DOWN\GO\NL-VS-L1.P.html#gene818) | single-organism metabolic process | 393 (31.85%) | 1115 (33.19%) | 0.903527 | 0.999992 |
| 819 | [GO:0009719](file:///E:\2018-7-3%E7%81%AB%E9%BE%99%E6%9E%9C%E8%BD%AC%E5%BD%95%E7%BB%84%E6%B5%8B%E5%BA%8F\%E5%AE%8C%E6%95%B4%E7%89%88%E6%95%B0%E6%8D%AE\GDR3855-Hylocereus_undulatus_Britt-12-RNAseq_result\4_Function\2_Group_Diff_Function\UP_DOWN\GO\NL-VS-L1.P.html#gene819) | response to endogenous stimulus | 24 (1.94%) | 79 (2.35%) | 0.905343 | 0.999992 |
| 820 | [GO:0031323](file:///E:\2018-7-3%E7%81%AB%E9%BE%99%E6%9E%9C%E8%BD%AC%E5%BD%95%E7%BB%84%E6%B5%8B%E5%BA%8F\%E5%AE%8C%E6%95%B4%E7%89%88%E6%95%B0%E6%8D%AE\GDR3855-Hylocereus_undulatus_Britt-12-RNAseq_result\4_Function\2_Group_Diff_Function\UP_DOWN\GO\NL-VS-L1.P.html#gene820) | regulation of cellular metabolic process | 19 (1.54%) | 64 (1.91%) | 0.906902 | 0.999992 |
| 821 | [GO:0009057](file:///E:\2018-7-3%E7%81%AB%E9%BE%99%E6%9E%9C%E8%BD%AC%E5%BD%95%E7%BB%84%E6%B5%8B%E5%BA%8F\%E5%AE%8C%E6%95%B4%E7%89%88%E6%95%B0%E6%8D%AE\GDR3855-Hylocereus_undulatus_Britt-12-RNAseq_result\4_Function\2_Group_Diff_Function\UP_DOWN\GO\NL-VS-L1.P.html#gene821) | macromolecule catabolic process | 16 (1.3%) | 55 (1.64%) | 0.909630 | 0.999992 |
| 822 | [GO:0007031](file:///E:\2018-7-3%E7%81%AB%E9%BE%99%E6%9E%9C%E8%BD%AC%E5%BD%95%E7%BB%84%E6%B5%8B%E5%BA%8F\%E5%AE%8C%E6%95%B4%E7%89%88%E6%95%B0%E6%8D%AE\GDR3855-Hylocereus_undulatus_Britt-12-RNAseq_result\4_Function\2_Group_Diff_Function\UP_DOWN\GO\NL-VS-L1.P.html#gene822) | peroxisome organization | 3 (0.24%) | 13 (0.39%) | 0.909818 | 0.999992 |
| 823 | [GO:0009062](file:///E:\2018-7-3%E7%81%AB%E9%BE%99%E6%9E%9C%E8%BD%AC%E5%BD%95%E7%BB%84%E6%B5%8B%E5%BA%8F\%E5%AE%8C%E6%95%B4%E7%89%88%E6%95%B0%E6%8D%AE\GDR3855-Hylocereus_undulatus_Britt-12-RNAseq_result\4_Function\2_Group_Diff_Function\UP_DOWN\GO\NL-VS-L1.P.html#gene823) | fatty acid catabolic process | 3 (0.24%) | 13 (0.39%) | 0.909818 | 0.999992 |
| 824 | [GO:0010243](file:///E:\2018-7-3%E7%81%AB%E9%BE%99%E6%9E%9C%E8%BD%AC%E5%BD%95%E7%BB%84%E6%B5%8B%E5%BA%8F\%E5%AE%8C%E6%95%B4%E7%89%88%E6%95%B0%E6%8D%AE\GDR3855-Hylocereus_undulatus_Britt-12-RNAseq_result\4_Function\2_Group_Diff_Function\UP_DOWN\GO\NL-VS-L1.P.html#gene824) | response to organonitrogen compound | 3 (0.24%) | 13 (0.39%) | 0.909818 | 0.999992 |
| 825 | [GO:0031326](file:///E:\2018-7-3%E7%81%AB%E9%BE%99%E6%9E%9C%E8%BD%AC%E5%BD%95%E7%BB%84%E6%B5%8B%E5%BA%8F\%E5%AE%8C%E6%95%B4%E7%89%88%E6%95%B0%E6%8D%AE\GDR3855-Hylocereus_undulatus_Britt-12-RNAseq_result\4_Function\2_Group_Diff_Function\UP_DOWN\GO\NL-VS-L1.P.html#gene825) | regulation of cellular biosynthetic process | 3 (0.24%) | 13 (0.39%) | 0.909818 | 0.999992 |
| 826 | [GO:0072329](file:///E:\2018-7-3%E7%81%AB%E9%BE%99%E6%9E%9C%E8%BD%AC%E5%BD%95%E7%BB%84%E6%B5%8B%E5%BA%8F\%E5%AE%8C%E6%95%B4%E7%89%88%E6%95%B0%E6%8D%AE\GDR3855-Hylocereus_undulatus_Britt-12-RNAseq_result\4_Function\2_Group_Diff_Function\UP_DOWN\GO\NL-VS-L1.P.html#gene826) | monocarboxylic acid catabolic process | 3 (0.24%) | 13 (0.39%) | 0.909818 | 0.999992 |
| 827 | [GO:0006778](file:///E:\2018-7-3%E7%81%AB%E9%BE%99%E6%9E%9C%E8%BD%AC%E5%BD%95%E7%BB%84%E6%B5%8B%E5%BA%8F\%E5%AE%8C%E6%95%B4%E7%89%88%E6%95%B0%E6%8D%AE\GDR3855-Hylocereus_undulatus_Britt-12-RNAseq_result\4_Function\2_Group_Diff_Function\UP_DOWN\GO\NL-VS-L1.P.html#gene827) | porphyrin-containing compound metabolic process | 5 (0.41%) | 20 (0.6%) | 0.910941 | 0.999992 |
| 828 | [GO:0008380](file:///E:\2018-7-3%E7%81%AB%E9%BE%99%E6%9E%9C%E8%BD%AC%E5%BD%95%E7%BB%84%E6%B5%8B%E5%BA%8F\%E5%AE%8C%E6%95%B4%E7%89%88%E6%95%B0%E6%8D%AE\GDR3855-Hylocereus_undulatus_Britt-12-RNAseq_result\4_Function\2_Group_Diff_Function\UP_DOWN\GO\NL-VS-L1.P.html#gene828) | RNA splicing | 5 (0.41%) | 20 (0.6%) | 0.910941 | 0.999992 |
| 829 | [GO:0072527](file:///E:\2018-7-3%E7%81%AB%E9%BE%99%E6%9E%9C%E8%BD%AC%E5%BD%95%E7%BB%84%E6%B5%8B%E5%BA%8F\%E5%AE%8C%E6%95%B4%E7%89%88%E6%95%B0%E6%8D%AE\GDR3855-Hylocereus_undulatus_Britt-12-RNAseq_result\4_Function\2_Group_Diff_Function\UP_DOWN\GO\NL-VS-L1.P.html#gene829) | pyrimidine-containing compound metabolic process | 5 (0.41%) | 20 (0.6%) | 0.910941 | 0.999992 |
| 830 | [GO:0019538](file:///E:\2018-7-3%E7%81%AB%E9%BE%99%E6%9E%9C%E8%BD%AC%E5%BD%95%E7%BB%84%E6%B5%8B%E5%BA%8F\%E5%AE%8C%E6%95%B4%E7%89%88%E6%95%B0%E6%8D%AE\GDR3855-Hylocereus_undulatus_Britt-12-RNAseq_result\4_Function\2_Group_Diff_Function\UP_DOWN\GO\NL-VS-L1.P.html#gene830) | protein metabolic process | 217 (17.59%) | 630 (18.76%) | 0.915050 | 0.999992 |
| 831 | [GO:0044281](file:///E:\2018-7-3%E7%81%AB%E9%BE%99%E6%9E%9C%E8%BD%AC%E5%BD%95%E7%BB%84%E6%B5%8B%E5%BA%8F\%E5%AE%8C%E6%95%B4%E7%89%88%E6%95%B0%E6%8D%AE\GDR3855-Hylocereus_undulatus_Britt-12-RNAseq_result\4_Function\2_Group_Diff_Function\UP_DOWN\GO\NL-VS-L1.P.html#gene831) | small molecule metabolic process | 167 (13.53%) | 490 (14.59%) | 0.915129 | 0.999992 |
| 832 | [GO:0006399](file:///E:\2018-7-3%E7%81%AB%E9%BE%99%E6%9E%9C%E8%BD%AC%E5%BD%95%E7%BB%84%E6%B5%8B%E5%BA%8F\%E5%AE%8C%E6%95%B4%E7%89%88%E6%95%B0%E6%8D%AE\GDR3855-Hylocereus_undulatus_Britt-12-RNAseq_result\4_Function\2_Group_Diff_Function\UP_DOWN\GO\NL-VS-L1.P.html#gene832) | tRNA metabolic process | 7 (0.57%) | 27 (0.8%) | 0.917962 | 0.999992 |
| 833 | [GO:0043043](file:///E:\2018-7-3%E7%81%AB%E9%BE%99%E6%9E%9C%E8%BD%AC%E5%BD%95%E7%BB%84%E6%B5%8B%E5%BA%8F\%E5%AE%8C%E6%95%B4%E7%89%88%E6%95%B0%E6%8D%AE\GDR3855-Hylocereus_undulatus_Britt-12-RNAseq_result\4_Function\2_Group_Diff_Function\UP_DOWN\GO\NL-VS-L1.P.html#gene833) | peptide biosynthetic process | 24 (1.94%) | 80 (2.38%) | 0.918246 | 0.999992 |
| 834 | [GO:0006721](file:///E:\2018-7-3%E7%81%AB%E9%BE%99%E6%9E%9C%E8%BD%AC%E5%BD%95%E7%BB%84%E6%B5%8B%E5%BA%8F\%E5%AE%8C%E6%95%B4%E7%89%88%E6%95%B0%E6%8D%AE\GDR3855-Hylocereus_undulatus_Britt-12-RNAseq_result\4_Function\2_Group_Diff_Function\UP_DOWN\GO\NL-VS-L1.P.html#gene834) | terpenoid metabolic process | 4 (0.32%) | 17 (0.51%) | 0.921450 | 0.999992 |
| 835 | [GO:0006164](file:///E:\2018-7-3%E7%81%AB%E9%BE%99%E6%9E%9C%E8%BD%AC%E5%BD%95%E7%BB%84%E6%B5%8B%E5%BA%8F\%E5%AE%8C%E6%95%B4%E7%89%88%E6%95%B0%E6%8D%AE\GDR3855-Hylocereus_undulatus_Britt-12-RNAseq_result\4_Function\2_Group_Diff_Function\UP_DOWN\GO\NL-VS-L1.P.html#gene835) | purine nucleotide biosynthetic process | 10 (0.81%) | 37 (1.1%) | 0.922558 | 0.999992 |
| 836 | [GO:0009152](file:///E:\2018-7-3%E7%81%AB%E9%BE%99%E6%9E%9C%E8%BD%AC%E5%BD%95%E7%BB%84%E6%B5%8B%E5%BA%8F\%E5%AE%8C%E6%95%B4%E7%89%88%E6%95%B0%E6%8D%AE\GDR3855-Hylocereus_undulatus_Britt-12-RNAseq_result\4_Function\2_Group_Diff_Function\UP_DOWN\GO\NL-VS-L1.P.html#gene836) | purine ribonucleotide biosynthetic process | 10 (0.81%) | 37 (1.1%) | 0.922558 | 0.999992 |
| 837 | [GO:0044712](file:///E:\2018-7-3%E7%81%AB%E9%BE%99%E6%9E%9C%E8%BD%AC%E5%BD%95%E7%BB%84%E6%B5%8B%E5%BA%8F\%E5%AE%8C%E6%95%B4%E7%89%88%E6%95%B0%E6%8D%AE\GDR3855-Hylocereus_undulatus_Britt-12-RNAseq_result\4_Function\2_Group_Diff_Function\UP_DOWN\GO\NL-VS-L1.P.html#gene837) | single-organism catabolic process | 56 (4.54%) | 175 (5.21%) | 0.922626 | 0.999992 |
| 838 | [GO:0019222](file:///E:\2018-7-3%E7%81%AB%E9%BE%99%E6%9E%9C%E8%BD%AC%E5%BD%95%E7%BB%84%E6%B5%8B%E5%BA%8F\%E5%AE%8C%E6%95%B4%E7%89%88%E6%95%B0%E6%8D%AE\GDR3855-Hylocereus_undulatus_Britt-12-RNAseq_result\4_Function\2_Group_Diff_Function\UP_DOWN\GO\NL-VS-L1.P.html#gene838) | regulation of metabolic process | 32 (2.59%) | 105 (3.13%) | 0.928708 | 0.999992 |
| 839 | [GO:0048856](file:///E:\2018-7-3%E7%81%AB%E9%BE%99%E6%9E%9C%E8%BD%AC%E5%BD%95%E7%BB%84%E6%B5%8B%E5%BA%8F\%E5%AE%8C%E6%95%B4%E7%89%88%E6%95%B0%E6%8D%AE\GDR3855-Hylocereus_undulatus_Britt-12-RNAseq_result\4_Function\2_Group_Diff_Function\UP_DOWN\GO\NL-VS-L1.P.html#gene839) | anatomical structure development | 39 (3.16%) | 126 (3.75%) | 0.930229 | 0.999992 |
| 840 | [GO:0006188](file:///E:\2018-7-3%E7%81%AB%E9%BE%99%E6%9E%9C%E8%BD%AC%E5%BD%95%E7%BB%84%E6%B5%8B%E5%BA%8F\%E5%AE%8C%E6%95%B4%E7%89%88%E6%95%B0%E6%8D%AE\GDR3855-Hylocereus_undulatus_Britt-12-RNAseq_result\4_Function\2_Group_Diff_Function\UP_DOWN\GO\NL-VS-L1.P.html#gene840) | IMP biosynthetic process | 2 (0.16%) | 10 (0.3%) | 0.930396 | 0.999992 |
| 841 | [GO:0008299](file:///E:\2018-7-3%E7%81%AB%E9%BE%99%E6%9E%9C%E8%BD%AC%E5%BD%95%E7%BB%84%E6%B5%8B%E5%BA%8F\%E5%AE%8C%E6%95%B4%E7%89%88%E6%95%B0%E6%8D%AE\GDR3855-Hylocereus_undulatus_Britt-12-RNAseq_result\4_Function\2_Group_Diff_Function\UP_DOWN\GO\NL-VS-L1.P.html#gene841) | isoprenoid biosynthetic process | 2 (0.16%) | 10 (0.3%) | 0.930396 | 0.999992 |
| 842 | [GO:0046040](file:///E:\2018-7-3%E7%81%AB%E9%BE%99%E6%9E%9C%E8%BD%AC%E5%BD%95%E7%BB%84%E6%B5%8B%E5%BA%8F\%E5%AE%8C%E6%95%B4%E7%89%88%E6%95%B0%E6%8D%AE\GDR3855-Hylocereus_undulatus_Britt-12-RNAseq_result\4_Function\2_Group_Diff_Function\UP_DOWN\GO\NL-VS-L1.P.html#gene842) | IMP metabolic process | 2 (0.16%) | 10 (0.3%) | 0.930396 | 0.999992 |
| 843 | [GO:2000112](file:///E:\2018-7-3%E7%81%AB%E9%BE%99%E6%9E%9C%E8%BD%AC%E5%BD%95%E7%BB%84%E6%B5%8B%E5%BA%8F\%E5%AE%8C%E6%95%B4%E7%89%88%E6%95%B0%E6%8D%AE\GDR3855-Hylocereus_undulatus_Britt-12-RNAseq_result\4_Function\2_Group_Diff_Function\UP_DOWN\GO\NL-VS-L1.P.html#gene843) | regulation of cellular macromolecule biosynthetic process | 2 (0.16%) | 10 (0.3%) | 0.930396 | 0.999992 |
| 844 | [GO:0006508](file:///E:\2018-7-3%E7%81%AB%E9%BE%99%E6%9E%9C%E8%BD%AC%E5%BD%95%E7%BB%84%E6%B5%8B%E5%BA%8F\%E5%AE%8C%E6%95%B4%E7%89%88%E6%95%B0%E6%8D%AE\GDR3855-Hylocereus_undulatus_Britt-12-RNAseq_result\4_Function\2_Group_Diff_Function\UP_DOWN\GO\NL-VS-L1.P.html#gene844) | proteolysis | 41 (3.32%) | 132 (3.93%) | 0.930903 | 0.999992 |
| 845 | [GO:0016192](file:///E:\2018-7-3%E7%81%AB%E9%BE%99%E6%9E%9C%E8%BD%AC%E5%BD%95%E7%BB%84%E6%B5%8B%E5%BA%8F\%E5%AE%8C%E6%95%B4%E7%89%88%E6%95%B0%E6%8D%AE\GDR3855-Hylocereus_undulatus_Britt-12-RNAseq_result\4_Function\2_Group_Diff_Function\UP_DOWN\GO\NL-VS-L1.P.html#gene845) | vesicle-mediated transport | 5 (0.41%) | 21 (0.63%) | 0.932237 | 0.999992 |
| 846 | [GO:0006972](file:///E:\2018-7-3%E7%81%AB%E9%BE%99%E6%9E%9C%E8%BD%AC%E5%BD%95%E7%BB%84%E6%B5%8B%E5%BA%8F\%E5%AE%8C%E6%95%B4%E7%89%88%E6%95%B0%E6%8D%AE\GDR3855-Hylocereus_undulatus_Britt-12-RNAseq_result\4_Function\2_Group_Diff_Function\UP_DOWN\GO\NL-VS-L1.P.html#gene846) | hyperosmotic response | 3 (0.24%) | 14 (0.42%) | 0.934909 | 0.999992 |
| 847 | [GO:0009889](file:///E:\2018-7-3%E7%81%AB%E9%BE%99%E6%9E%9C%E8%BD%AC%E5%BD%95%E7%BB%84%E6%B5%8B%E5%BA%8F\%E5%AE%8C%E6%95%B4%E7%89%88%E6%95%B0%E6%8D%AE\GDR3855-Hylocereus_undulatus_Britt-12-RNAseq_result\4_Function\2_Group_Diff_Function\UP_DOWN\GO\NL-VS-L1.P.html#gene847) | regulation of biosynthetic process | 3 (0.24%) | 14 (0.42%) | 0.934909 | 0.999992 |
| 848 | [GO:0016054](file:///E:\2018-7-3%E7%81%AB%E9%BE%99%E6%9E%9C%E8%BD%AC%E5%BD%95%E7%BB%84%E6%B5%8B%E5%BA%8F\%E5%AE%8C%E6%95%B4%E7%89%88%E6%95%B0%E6%8D%AE\GDR3855-Hylocereus_undulatus_Britt-12-RNAseq_result\4_Function\2_Group_Diff_Function\UP_DOWN\GO\NL-VS-L1.P.html#gene848) | organic acid catabolic process | 3 (0.24%) | 14 (0.42%) | 0.934909 | 0.999992 |
| 849 | [GO:0046395](file:///E:\2018-7-3%E7%81%AB%E9%BE%99%E6%9E%9C%E8%BD%AC%E5%BD%95%E7%BB%84%E6%B5%8B%E5%BA%8F\%E5%AE%8C%E6%95%B4%E7%89%88%E6%95%B0%E6%8D%AE\GDR3855-Hylocereus_undulatus_Britt-12-RNAseq_result\4_Function\2_Group_Diff_Function\UP_DOWN\GO\NL-VS-L1.P.html#gene849) | carboxylic acid catabolic process | 3 (0.24%) | 14 (0.42%) | 0.934909 | 0.999992 |
| 850 | [GO:0001510](file:///E:\2018-7-3%E7%81%AB%E9%BE%99%E6%9E%9C%E8%BD%AC%E5%BD%95%E7%BB%84%E6%B5%8B%E5%BA%8F\%E5%AE%8C%E6%95%B4%E7%89%88%E6%95%B0%E6%8D%AE\GDR3855-Hylocereus_undulatus_Britt-12-RNAseq_result\4_Function\2_Group_Diff_Function\UP_DOWN\GO\NL-VS-L1.P.html#gene850) | RNA methylation | 1 (0.08%) | 6 (0.18%) | 0.936061 | 0.999992 |
| 851 | [GO:0006310](file:///E:\2018-7-3%E7%81%AB%E9%BE%99%E6%9E%9C%E8%BD%AC%E5%BD%95%E7%BB%84%E6%B5%8B%E5%BA%8F\%E5%AE%8C%E6%95%B4%E7%89%88%E6%95%B0%E6%8D%AE\GDR3855-Hylocereus_undulatus_Britt-12-RNAseq_result\4_Function\2_Group_Diff_Function\UP_DOWN\GO\NL-VS-L1.P.html#gene851) | DNA recombination | 1 (0.08%) | 6 (0.18%) | 0.936061 | 0.999992 |
| 852 | [GO:0006486](file:///E:\2018-7-3%E7%81%AB%E9%BE%99%E6%9E%9C%E8%BD%AC%E5%BD%95%E7%BB%84%E6%B5%8B%E5%BA%8F\%E5%AE%8C%E6%95%B4%E7%89%88%E6%95%B0%E6%8D%AE\GDR3855-Hylocereus_undulatus_Britt-12-RNAseq_result\4_Function\2_Group_Diff_Function\UP_DOWN\GO\NL-VS-L1.P.html#gene852) | protein glycosylation | 1 (0.08%) | 6 (0.18%) | 0.936061 | 0.999992 |
| 853 | [GO:0008283](file:///E:\2018-7-3%E7%81%AB%E9%BE%99%E6%9E%9C%E8%BD%AC%E5%BD%95%E7%BB%84%E6%B5%8B%E5%BA%8F\%E5%AE%8C%E6%95%B4%E7%89%88%E6%95%B0%E6%8D%AE\GDR3855-Hylocereus_undulatus_Britt-12-RNAseq_result\4_Function\2_Group_Diff_Function\UP_DOWN\GO\NL-VS-L1.P.html#gene853) | cell proliferation | 1 (0.08%) | 6 (0.18%) | 0.936061 | 0.999992 |
| 854 | [GO:0008643](file:///E:\2018-7-3%E7%81%AB%E9%BE%99%E6%9E%9C%E8%BD%AC%E5%BD%95%E7%BB%84%E6%B5%8B%E5%BA%8F\%E5%AE%8C%E6%95%B4%E7%89%88%E6%95%B0%E6%8D%AE\GDR3855-Hylocereus_undulatus_Britt-12-RNAseq_result\4_Function\2_Group_Diff_Function\UP_DOWN\GO\NL-VS-L1.P.html#gene854) | carbohydrate transport | 1 (0.08%) | 6 (0.18%) | 0.936061 | 0.999992 |
| 855 | [GO:0009100](file:///E:\2018-7-3%E7%81%AB%E9%BE%99%E6%9E%9C%E8%BD%AC%E5%BD%95%E7%BB%84%E6%B5%8B%E5%BA%8F\%E5%AE%8C%E6%95%B4%E7%89%88%E6%95%B0%E6%8D%AE\GDR3855-Hylocereus_undulatus_Britt-12-RNAseq_result\4_Function\2_Group_Diff_Function\UP_DOWN\GO\NL-VS-L1.P.html#gene855) | glycoprotein metabolic process | 1 (0.08%) | 6 (0.18%) | 0.936061 | 0.999992 |
| 856 | [GO:0009101](file:///E:\2018-7-3%E7%81%AB%E9%BE%99%E6%9E%9C%E8%BD%AC%E5%BD%95%E7%BB%84%E6%B5%8B%E5%BA%8F\%E5%AE%8C%E6%95%B4%E7%89%88%E6%95%B0%E6%8D%AE\GDR3855-Hylocereus_undulatus_Britt-12-RNAseq_result\4_Function\2_Group_Diff_Function\UP_DOWN\GO\NL-VS-L1.P.html#gene856) | glycoprotein biosynthetic process | 1 (0.08%) | 6 (0.18%) | 0.936061 | 0.999992 |
| 857 | [GO:0009890](file:///E:\2018-7-3%E7%81%AB%E9%BE%99%E6%9E%9C%E8%BD%AC%E5%BD%95%E7%BB%84%E6%B5%8B%E5%BA%8F\%E5%AE%8C%E6%95%B4%E7%89%88%E6%95%B0%E6%8D%AE\GDR3855-Hylocereus_undulatus_Britt-12-RNAseq_result\4_Function\2_Group_Diff_Function\UP_DOWN\GO\NL-VS-L1.P.html#gene857) | negative regulation of biosynthetic process | 1 (0.08%) | 6 (0.18%) | 0.936061 | 0.999992 |
| 858 | [GO:0009966](file:///E:\2018-7-3%E7%81%AB%E9%BE%99%E6%9E%9C%E8%BD%AC%E5%BD%95%E7%BB%84%E6%B5%8B%E5%BA%8F\%E5%AE%8C%E6%95%B4%E7%89%88%E6%95%B0%E6%8D%AE\GDR3855-Hylocereus_undulatus_Britt-12-RNAseq_result\4_Function\2_Group_Diff_Function\UP_DOWN\GO\NL-VS-L1.P.html#gene858) | regulation of signal transduction | 1 (0.08%) | 6 (0.18%) | 0.936061 | 0.999992 |
| 859 | [GO:0010383](file:///E:\2018-7-3%E7%81%AB%E9%BE%99%E6%9E%9C%E8%BD%AC%E5%BD%95%E7%BB%84%E6%B5%8B%E5%BA%8F\%E5%AE%8C%E6%95%B4%E7%89%88%E6%95%B0%E6%8D%AE\GDR3855-Hylocereus_undulatus_Britt-12-RNAseq_result\4_Function\2_Group_Diff_Function\UP_DOWN\GO\NL-VS-L1.P.html#gene859) | cell wall polysaccharide metabolic process | 1 (0.08%) | 6 (0.18%) | 0.936061 | 0.999992 |
| 860 | [GO:0010410](file:///E:\2018-7-3%E7%81%AB%E9%BE%99%E6%9E%9C%E8%BD%AC%E5%BD%95%E7%BB%84%E6%B5%8B%E5%BA%8F\%E5%AE%8C%E6%95%B4%E7%89%88%E6%95%B0%E6%8D%AE\GDR3855-Hylocereus_undulatus_Britt-12-RNAseq_result\4_Function\2_Group_Diff_Function\UP_DOWN\GO\NL-VS-L1.P.html#gene860) | hemicellulose metabolic process | 1 (0.08%) | 6 (0.18%) | 0.936061 | 0.999992 |
| 861 | [GO:0010558](file:///E:\2018-7-3%E7%81%AB%E9%BE%99%E6%9E%9C%E8%BD%AC%E5%BD%95%E7%BB%84%E6%B5%8B%E5%BA%8F\%E5%AE%8C%E6%95%B4%E7%89%88%E6%95%B0%E6%8D%AE\GDR3855-Hylocereus_undulatus_Britt-12-RNAseq_result\4_Function\2_Group_Diff_Function\UP_DOWN\GO\NL-VS-L1.P.html#gene861) | negative regulation of macromolecule biosynthetic process | 1 (0.08%) | 6 (0.18%) | 0.936061 | 0.999992 |
| 862 | [GO:0010646](file:///E:\2018-7-3%E7%81%AB%E9%BE%99%E6%9E%9C%E8%BD%AC%E5%BD%95%E7%BB%84%E6%B5%8B%E5%BA%8F\%E5%AE%8C%E6%95%B4%E7%89%88%E6%95%B0%E6%8D%AE\GDR3855-Hylocereus_undulatus_Britt-12-RNAseq_result\4_Function\2_Group_Diff_Function\UP_DOWN\GO\NL-VS-L1.P.html#gene862) | regulation of cell communication | 1 (0.08%) | 6 (0.18%) | 0.936061 | 0.999992 |
| 863 | [GO:0023051](file:///E:\2018-7-3%E7%81%AB%E9%BE%99%E6%9E%9C%E8%BD%AC%E5%BD%95%E7%BB%84%E6%B5%8B%E5%BA%8F\%E5%AE%8C%E6%95%B4%E7%89%88%E6%95%B0%E6%8D%AE\GDR3855-Hylocereus_undulatus_Britt-12-RNAseq_result\4_Function\2_Group_Diff_Function\UP_DOWN\GO\NL-VS-L1.P.html#gene863) | regulation of signaling | 1 (0.08%) | 6 (0.18%) | 0.936061 | 0.999992 |
| 864 | [GO:0031327](file:///E:\2018-7-3%E7%81%AB%E9%BE%99%E6%9E%9C%E8%BD%AC%E5%BD%95%E7%BB%84%E6%B5%8B%E5%BA%8F\%E5%AE%8C%E6%95%B4%E7%89%88%E6%95%B0%E6%8D%AE\GDR3855-Hylocereus_undulatus_Britt-12-RNAseq_result\4_Function\2_Group_Diff_Function\UP_DOWN\GO\NL-VS-L1.P.html#gene864) | negative regulation of cellular biosynthetic process | 1 (0.08%) | 6 (0.18%) | 0.936061 | 0.999992 |
| 865 | [GO:0043413](file:///E:\2018-7-3%E7%81%AB%E9%BE%99%E6%9E%9C%E8%BD%AC%E5%BD%95%E7%BB%84%E6%B5%8B%E5%BA%8F\%E5%AE%8C%E6%95%B4%E7%89%88%E6%95%B0%E6%8D%AE\GDR3855-Hylocereus_undulatus_Britt-12-RNAseq_result\4_Function\2_Group_Diff_Function\UP_DOWN\GO\NL-VS-L1.P.html#gene865) | macromolecule glycosylation | 1 (0.08%) | 6 (0.18%) | 0.936061 | 0.999992 |
| 866 | [GO:0045491](file:///E:\2018-7-3%E7%81%AB%E9%BE%99%E6%9E%9C%E8%BD%AC%E5%BD%95%E7%BB%84%E6%B5%8B%E5%BA%8F\%E5%AE%8C%E6%95%B4%E7%89%88%E6%95%B0%E6%8D%AE\GDR3855-Hylocereus_undulatus_Britt-12-RNAseq_result\4_Function\2_Group_Diff_Function\UP_DOWN\GO\NL-VS-L1.P.html#gene866) | xylan metabolic process | 1 (0.08%) | 6 (0.18%) | 0.936061 | 0.999992 |
| 867 | [GO:0051172](file:///E:\2018-7-3%E7%81%AB%E9%BE%99%E6%9E%9C%E8%BD%AC%E5%BD%95%E7%BB%84%E6%B5%8B%E5%BA%8F\%E5%AE%8C%E6%95%B4%E7%89%88%E6%95%B0%E6%8D%AE\GDR3855-Hylocereus_undulatus_Britt-12-RNAseq_result\4_Function\2_Group_Diff_Function\UP_DOWN\GO\NL-VS-L1.P.html#gene867) | negative regulation of nitrogen compound metabolic process | 1 (0.08%) | 6 (0.18%) | 0.936061 | 0.999992 |
| 868 | [GO:0051321](file:///E:\2018-7-3%E7%81%AB%E9%BE%99%E6%9E%9C%E8%BD%AC%E5%BD%95%E7%BB%84%E6%B5%8B%E5%BA%8F\%E5%AE%8C%E6%95%B4%E7%89%88%E6%95%B0%E6%8D%AE\GDR3855-Hylocereus_undulatus_Britt-12-RNAseq_result\4_Function\2_Group_Diff_Function\UP_DOWN\GO\NL-VS-L1.P.html#gene868) | meiotic cell cycle | 1 (0.08%) | 6 (0.18%) | 0.936061 | 0.999992 |
| 869 | [GO:0070646](file:///E:\2018-7-3%E7%81%AB%E9%BE%99%E6%9E%9C%E8%BD%AC%E5%BD%95%E7%BB%84%E6%B5%8B%E5%BA%8F\%E5%AE%8C%E6%95%B4%E7%89%88%E6%95%B0%E6%8D%AE\GDR3855-Hylocereus_undulatus_Britt-12-RNAseq_result\4_Function\2_Group_Diff_Function\UP_DOWN\GO\NL-VS-L1.P.html#gene869) | protein modification by small protein removal | 1 (0.08%) | 6 (0.18%) | 0.936061 | 0.999992 |
| 870 | [GO:1903046](file:///E:\2018-7-3%E7%81%AB%E9%BE%99%E6%9E%9C%E8%BD%AC%E5%BD%95%E7%BB%84%E6%B5%8B%E5%BA%8F\%E5%AE%8C%E6%95%B4%E7%89%88%E6%95%B0%E6%8D%AE\GDR3855-Hylocereus_undulatus_Britt-12-RNAseq_result\4_Function\2_Group_Diff_Function\UP_DOWN\GO\NL-VS-L1.P.html#gene870) | meiotic cell cycle process | 1 (0.08%) | 6 (0.18%) | 0.936061 | 0.999992 |
| 871 | [GO:2000113](file:///E:\2018-7-3%E7%81%AB%E9%BE%99%E6%9E%9C%E8%BD%AC%E5%BD%95%E7%BB%84%E6%B5%8B%E5%BA%8F\%E5%AE%8C%E6%95%B4%E7%89%88%E6%95%B0%E6%8D%AE\GDR3855-Hylocereus_undulatus_Britt-12-RNAseq_result\4_Function\2_Group_Diff_Function\UP_DOWN\GO\NL-VS-L1.P.html#gene871) | negative regulation of cellular macromolecule biosynthetic process | 1 (0.08%) | 6 (0.18%) | 0.936061 | 0.999992 |
| 872 | [GO:0045333](file:///E:\2018-7-3%E7%81%AB%E9%BE%99%E6%9E%9C%E8%BD%AC%E5%BD%95%E7%BB%84%E6%B5%8B%E5%BA%8F\%E5%AE%8C%E6%95%B4%E7%89%88%E6%95%B0%E6%8D%AE\GDR3855-Hylocereus_undulatus_Britt-12-RNAseq_result\4_Function\2_Group_Diff_Function\UP_DOWN\GO\NL-VS-L1.P.html#gene872) | cellular respiration | 10 (0.81%) | 38 (1.13%) | 0.937548 | 0.999992 |
| 873 | [GO:0072522](file:///E:\2018-7-3%E7%81%AB%E9%BE%99%E6%9E%9C%E8%BD%AC%E5%BD%95%E7%BB%84%E6%B5%8B%E5%BA%8F\%E5%AE%8C%E6%95%B4%E7%89%88%E6%95%B0%E6%8D%AE\GDR3855-Hylocereus_undulatus_Britt-12-RNAseq_result\4_Function\2_Group_Diff_Function\UP_DOWN\GO\NL-VS-L1.P.html#gene873) | purine-containing compound biosynthetic process | 10 (0.81%) | 38 (1.13%) | 0.937548 | 0.999992 |
| 874 | [GO:0007275](file:///E:\2018-7-3%E7%81%AB%E9%BE%99%E6%9E%9C%E8%BD%AC%E5%BD%95%E7%BB%84%E6%B5%8B%E5%BA%8F\%E5%AE%8C%E6%95%B4%E7%89%88%E6%95%B0%E6%8D%AE\GDR3855-Hylocereus_undulatus_Britt-12-RNAseq_result\4_Function\2_Group_Diff_Function\UP_DOWN\GO\NL-VS-L1.P.html#gene874) | multicellular organism development | 33 (2.67%) | 109 (3.25%) | 0.937833 | 0.999992 |
| 875 | [GO:0006518](file:///E:\2018-7-3%E7%81%AB%E9%BE%99%E6%9E%9C%E8%BD%AC%E5%BD%95%E7%BB%84%E6%B5%8B%E5%BA%8F\%E5%AE%8C%E6%95%B4%E7%89%88%E6%95%B0%E6%8D%AE\GDR3855-Hylocereus_undulatus_Britt-12-RNAseq_result\4_Function\2_Group_Diff_Function\UP_DOWN\GO\NL-VS-L1.P.html#gene875) | peptide metabolic process | 24 (1.94%) | 82 (2.44%) | 0.939740 | 0.999992 |
| 876 | [GO:0009165](file:///E:\2018-7-3%E7%81%AB%E9%BE%99%E6%9E%9C%E8%BD%AC%E5%BD%95%E7%BB%84%E6%B5%8B%E5%BA%8F\%E5%AE%8C%E6%95%B4%E7%89%88%E6%95%B0%E6%8D%AE\GDR3855-Hylocereus_undulatus_Britt-12-RNAseq_result\4_Function\2_Group_Diff_Function\UP_DOWN\GO\NL-VS-L1.P.html#gene876) | nucleotide biosynthetic process | 14 (1.13%) | 51 (1.52%) | 0.939961 | 0.999992 |
| 877 | [GO:0006412](file:///E:\2018-7-3%E7%81%AB%E9%BE%99%E6%9E%9C%E8%BD%AC%E5%BD%95%E7%BB%84%E6%B5%8B%E5%BA%8F\%E5%AE%8C%E6%95%B4%E7%89%88%E6%95%B0%E6%8D%AE\GDR3855-Hylocereus_undulatus_Britt-12-RNAseq_result\4_Function\2_Group_Diff_Function\UP_DOWN\GO\NL-VS-L1.P.html#gene877) | translation | 23 (1.86%) | 79 (2.35%) | 0.940291 | 0.999992 |
| 878 | [GO:0006754](file:///E:\2018-7-3%E7%81%AB%E9%BE%99%E6%9E%9C%E8%BD%AC%E5%BD%95%E7%BB%84%E6%B5%8B%E5%BA%8F\%E5%AE%8C%E6%95%B4%E7%89%88%E6%95%B0%E6%8D%AE\GDR3855-Hylocereus_undulatus_Britt-12-RNAseq_result\4_Function\2_Group_Diff_Function\UP_DOWN\GO\NL-VS-L1.P.html#gene878) | ATP biosynthetic process | 4 (0.32%) | 18 (0.54%) | 0.941777 | 0.999992 |
| 879 | [GO:0009144](file:///E:\2018-7-3%E7%81%AB%E9%BE%99%E6%9E%9C%E8%BD%AC%E5%BD%95%E7%BB%84%E6%B5%8B%E5%BA%8F\%E5%AE%8C%E6%95%B4%E7%89%88%E6%95%B0%E6%8D%AE\GDR3855-Hylocereus_undulatus_Britt-12-RNAseq_result\4_Function\2_Group_Diff_Function\UP_DOWN\GO\NL-VS-L1.P.html#gene879) | purine nucleoside triphosphate metabolic process | 4 (0.32%) | 18 (0.54%) | 0.941777 | 0.999992 |
| 880 | [GO:0009145](file:///E:\2018-7-3%E7%81%AB%E9%BE%99%E6%9E%9C%E8%BD%AC%E5%BD%95%E7%BB%84%E6%B5%8B%E5%BA%8F\%E5%AE%8C%E6%95%B4%E7%89%88%E6%95%B0%E6%8D%AE\GDR3855-Hylocereus_undulatus_Britt-12-RNAseq_result\4_Function\2_Group_Diff_Function\UP_DOWN\GO\NL-VS-L1.P.html#gene880) | purine nucleoside triphosphate biosynthetic process | 4 (0.32%) | 18 (0.54%) | 0.941777 | 0.999992 |
| 881 | [GO:0009205](file:///E:\2018-7-3%E7%81%AB%E9%BE%99%E6%9E%9C%E8%BD%AC%E5%BD%95%E7%BB%84%E6%B5%8B%E5%BA%8F\%E5%AE%8C%E6%95%B4%E7%89%88%E6%95%B0%E6%8D%AE\GDR3855-Hylocereus_undulatus_Britt-12-RNAseq_result\4_Function\2_Group_Diff_Function\UP_DOWN\GO\NL-VS-L1.P.html#gene881) | purine ribonucleoside triphosphate metabolic process | 4 (0.32%) | 18 (0.54%) | 0.941777 | 0.999992 |
| 882 | [GO:0009206](file:///E:\2018-7-3%E7%81%AB%E9%BE%99%E6%9E%9C%E8%BD%AC%E5%BD%95%E7%BB%84%E6%B5%8B%E5%BA%8F\%E5%AE%8C%E6%95%B4%E7%89%88%E6%95%B0%E6%8D%AE\GDR3855-Hylocereus_undulatus_Britt-12-RNAseq_result\4_Function\2_Group_Diff_Function\UP_DOWN\GO\NL-VS-L1.P.html#gene882) | purine ribonucleoside triphosphate biosynthetic process | 4 (0.32%) | 18 (0.54%) | 0.941777 | 0.999992 |
| 883 | [GO:0046034](file:///E:\2018-7-3%E7%81%AB%E9%BE%99%E6%9E%9C%E8%BD%AC%E5%BD%95%E7%BB%84%E6%B5%8B%E5%BA%8F\%E5%AE%8C%E6%95%B4%E7%89%88%E6%95%B0%E6%8D%AE\GDR3855-Hylocereus_undulatus_Britt-12-RNAseq_result\4_Function\2_Group_Diff_Function\UP_DOWN\GO\NL-VS-L1.P.html#gene883) | ATP metabolic process | 4 (0.32%) | 18 (0.54%) | 0.941777 | 0.999992 |
| 884 | [GO:0009116](file:///E:\2018-7-3%E7%81%AB%E9%BE%99%E6%9E%9C%E8%BD%AC%E5%BD%95%E7%BB%84%E6%B5%8B%E5%BA%8F\%E5%AE%8C%E6%95%B4%E7%89%88%E6%95%B0%E6%8D%AE\GDR3855-Hylocereus_undulatus_Britt-12-RNAseq_result\4_Function\2_Group_Diff_Function\UP_DOWN\GO\NL-VS-L1.P.html#gene884) | nucleoside metabolic process | 5 (0.41%) | 22 (0.65%) | 0.948858 | 0.999992 |
| 885 | [GO:0009119](file:///E:\2018-7-3%E7%81%AB%E9%BE%99%E6%9E%9C%E8%BD%AC%E5%BD%95%E7%BB%84%E6%B5%8B%E5%BA%8F\%E5%AE%8C%E6%95%B4%E7%89%88%E6%95%B0%E6%8D%AE\GDR3855-Hylocereus_undulatus_Britt-12-RNAseq_result\4_Function\2_Group_Diff_Function\UP_DOWN\GO\NL-VS-L1.P.html#gene885) | ribonucleoside metabolic process | 5 (0.41%) | 22 (0.65%) | 0.948858 | 0.999992 |
| 886 | [GO:0009163](file:///E:\2018-7-3%E7%81%AB%E9%BE%99%E6%9E%9C%E8%BD%AC%E5%BD%95%E7%BB%84%E6%B5%8B%E5%BA%8F\%E5%AE%8C%E6%95%B4%E7%89%88%E6%95%B0%E6%8D%AE\GDR3855-Hylocereus_undulatus_Britt-12-RNAseq_result\4_Function\2_Group_Diff_Function\UP_DOWN\GO\NL-VS-L1.P.html#gene886) | nucleoside biosynthetic process | 5 (0.41%) | 22 (0.65%) | 0.948858 | 0.999992 |
| 887 | [GO:0040007](file:///E:\2018-7-3%E7%81%AB%E9%BE%99%E6%9E%9C%E8%BD%AC%E5%BD%95%E7%BB%84%E6%B5%8B%E5%BA%8F\%E5%AE%8C%E6%95%B4%E7%89%88%E6%95%B0%E6%8D%AE\GDR3855-Hylocereus_undulatus_Britt-12-RNAseq_result\4_Function\2_Group_Diff_Function\UP_DOWN\GO\NL-VS-L1.P.html#gene887) | growth | 5 (0.41%) | 22 (0.65%) | 0.948858 | 0.999992 |
| 888 | [GO:0042455](file:///E:\2018-7-3%E7%81%AB%E9%BE%99%E6%9E%9C%E8%BD%AC%E5%BD%95%E7%BB%84%E6%B5%8B%E5%BA%8F\%E5%AE%8C%E6%95%B4%E7%89%88%E6%95%B0%E6%8D%AE\GDR3855-Hylocereus_undulatus_Britt-12-RNAseq_result\4_Function\2_Group_Diff_Function\UP_DOWN\GO\NL-VS-L1.P.html#gene888) | ribonucleoside biosynthetic process | 5 (0.41%) | 22 (0.65%) | 0.948858 | 0.999992 |
| 889 | [GO:0010556](file:///E:\2018-7-3%E7%81%AB%E9%BE%99%E6%9E%9C%E8%BD%AC%E5%BD%95%E7%BB%84%E6%B5%8B%E5%BA%8F\%E5%AE%8C%E6%95%B4%E7%89%88%E6%95%B0%E6%8D%AE\GDR3855-Hylocereus_undulatus_Britt-12-RNAseq_result\4_Function\2_Group_Diff_Function\UP_DOWN\GO\NL-VS-L1.P.html#gene889) | regulation of macromolecule biosynthetic process | 2 (0.16%) | 11 (0.33%) | 0.952271 | 0.999992 |
| 890 | [GO:0010876](file:///E:\2018-7-3%E7%81%AB%E9%BE%99%E6%9E%9C%E8%BD%AC%E5%BD%95%E7%BB%84%E6%B5%8B%E5%BA%8F\%E5%AE%8C%E6%95%B4%E7%89%88%E6%95%B0%E6%8D%AE\GDR3855-Hylocereus_undulatus_Britt-12-RNAseq_result\4_Function\2_Group_Diff_Function\UP_DOWN\GO\NL-VS-L1.P.html#gene890) | lipid localization | 2 (0.16%) | 11 (0.33%) | 0.952271 | 0.999992 |
| 891 | [GO:1901136](file:///E:\2018-7-3%E7%81%AB%E9%BE%99%E6%9E%9C%E8%BD%AC%E5%BD%95%E7%BB%84%E6%B5%8B%E5%BA%8F\%E5%AE%8C%E6%95%B4%E7%89%88%E6%95%B0%E6%8D%AE\GDR3855-Hylocereus_undulatus_Britt-12-RNAseq_result\4_Function\2_Group_Diff_Function\UP_DOWN\GO\NL-VS-L1.P.html#gene891) | carbohydrate derivative catabolic process | 45 (3.65%) | 147 (4.38%) | 0.953264 | 0.999992 |
| 892 | [GO:0050793](file:///E:\2018-7-3%E7%81%AB%E9%BE%99%E6%9E%9C%E8%BD%AC%E5%BD%95%E7%BB%84%E6%B5%8B%E5%BA%8F\%E5%AE%8C%E6%95%B4%E7%89%88%E6%95%B0%E6%8D%AE\GDR3855-Hylocereus_undulatus_Britt-12-RNAseq_result\4_Function\2_Group_Diff_Function\UP_DOWN\GO\NL-VS-L1.P.html#gene892) | regulation of developmental process | 3 (0.24%) | 15 (0.45%) | 0.953409 | 0.999992 |
| 893 | [GO:0055080](file:///E:\2018-7-3%E7%81%AB%E9%BE%99%E6%9E%9C%E8%BD%AC%E5%BD%95%E7%BB%84%E6%B5%8B%E5%BA%8F\%E5%AE%8C%E6%95%B4%E7%89%88%E6%95%B0%E6%8D%AE\GDR3855-Hylocereus_undulatus_Britt-12-RNAseq_result\4_Function\2_Group_Diff_Function\UP_DOWN\GO\NL-VS-L1.P.html#gene893) | cation homeostasis | 3 (0.24%) | 15 (0.45%) | 0.953409 | 0.999992 |
| 894 | [GO:1901698](file:///E:\2018-7-3%E7%81%AB%E9%BE%99%E6%9E%9C%E8%BD%AC%E5%BD%95%E7%BB%84%E6%B5%8B%E5%BA%8F\%E5%AE%8C%E6%95%B4%E7%89%88%E6%95%B0%E6%8D%AE\GDR3855-Hylocereus_undulatus_Britt-12-RNAseq_result\4_Function\2_Group_Diff_Function\UP_DOWN\GO\NL-VS-L1.P.html#gene894) | response to nitrogen compound | 3 (0.24%) | 15 (0.45%) | 0.953409 | 0.999992 |
| 895 | [GO:0044267](file:///E:\2018-7-3%E7%81%AB%E9%BE%99%E6%9E%9C%E8%BD%AC%E5%BD%95%E7%BB%84%E6%B5%8B%E5%BA%8F\%E5%AE%8C%E6%95%B4%E7%89%88%E6%95%B0%E6%8D%AE\GDR3855-Hylocereus_undulatus_Britt-12-RNAseq_result\4_Function\2_Group_Diff_Function\UP_DOWN\GO\NL-VS-L1.P.html#gene895) | cellular protein metabolic process | 182 (14.75%) | 541 (16.11%) | 0.954018 | 0.999992 |
| 896 | [GO:0070838](file:///E:\2018-7-3%E7%81%AB%E9%BE%99%E6%9E%9C%E8%BD%AC%E5%BD%95%E7%BB%84%E6%B5%8B%E5%BA%8F\%E5%AE%8C%E6%95%B4%E7%89%88%E6%95%B0%E6%8D%AE\GDR3855-Hylocereus_undulatus_Britt-12-RNAseq_result\4_Function\2_Group_Diff_Function\UP_DOWN\GO\NL-VS-L1.P.html#gene896) | divalent metal ion transport | 6 (0.49%) | 26 (0.77%) | 0.955466 | 0.999992 |
| 897 | [GO:0072511](file:///E:\2018-7-3%E7%81%AB%E9%BE%99%E6%9E%9C%E8%BD%AC%E5%BD%95%E7%BB%84%E6%B5%8B%E5%BA%8F\%E5%AE%8C%E6%95%B4%E7%89%88%E6%95%B0%E6%8D%AE\GDR3855-Hylocereus_undulatus_Britt-12-RNAseq_result\4_Function\2_Group_Diff_Function\UP_DOWN\GO\NL-VS-L1.P.html#gene897) | divalent inorganic cation transport | 6 (0.49%) | 26 (0.77%) | 0.955466 | 0.999992 |
| 898 | [GO:0019637](file:///E:\2018-7-3%E7%81%AB%E9%BE%99%E6%9E%9C%E8%BD%AC%E5%BD%95%E7%BB%84%E6%B5%8B%E5%BA%8F\%E5%AE%8C%E6%95%B4%E7%89%88%E6%95%B0%E6%8D%AE\GDR3855-Hylocereus_undulatus_Britt-12-RNAseq_result\4_Function\2_Group_Diff_Function\UP_DOWN\GO\NL-VS-L1.P.html#gene898) | organophosphate metabolic process | 82 (6.65%) | 256 (7.62%) | 0.955723 | 0.999992 |
| 899 | [GO:0050789](file:///E:\2018-7-3%E7%81%AB%E9%BE%99%E6%9E%9C%E8%BD%AC%E5%BD%95%E7%BB%84%E6%B5%8B%E5%BA%8F\%E5%AE%8C%E6%95%B4%E7%89%88%E6%95%B0%E6%8D%AE\GDR3855-Hylocereus_undulatus_Britt-12-RNAseq_result\4_Function\2_Group_Diff_Function\UP_DOWN\GO\NL-VS-L1.P.html#gene899) | regulation of biological process | 98 (7.94%) | 303 (9.02%) | 0.958702 | 0.999992 |
| 900 | [GO:0044707](file:///E:\2018-7-3%E7%81%AB%E9%BE%99%E6%9E%9C%E8%BD%AC%E5%BD%95%E7%BB%84%E6%B5%8B%E5%BA%8F\%E5%AE%8C%E6%95%B4%E7%89%88%E6%95%B0%E6%8D%AE\GDR3855-Hylocereus_undulatus_Britt-12-RNAseq_result\4_Function\2_Group_Diff_Function\UP_DOWN\GO\NL-VS-L1.P.html#gene900) | single-multicellular organism process | 38 (3.08%) | 127 (3.78%) | 0.958721 | 0.999992 |
| 901 | [GO:0006195](file:///E:\2018-7-3%E7%81%AB%E9%BE%99%E6%9E%9C%E8%BD%AC%E5%BD%95%E7%BB%84%E6%B5%8B%E5%BA%8F\%E5%AE%8C%E6%95%B4%E7%89%88%E6%95%B0%E6%8D%AE\GDR3855-Hylocereus_undulatus_Britt-12-RNAseq_result\4_Function\2_Group_Diff_Function\UP_DOWN\GO\NL-VS-L1.P.html#gene901) | purine nucleotide catabolic process | 44 (3.57%) | 145 (4.32%) | 0.958791 | 0.999992 |
| 902 | [GO:0009154](file:///E:\2018-7-3%E7%81%AB%E9%BE%99%E6%9E%9C%E8%BD%AC%E5%BD%95%E7%BB%84%E6%B5%8B%E5%BA%8F\%E5%AE%8C%E6%95%B4%E7%89%88%E6%95%B0%E6%8D%AE\GDR3855-Hylocereus_undulatus_Britt-12-RNAseq_result\4_Function\2_Group_Diff_Function\UP_DOWN\GO\NL-VS-L1.P.html#gene902) | purine ribonucleotide catabolic process | 44 (3.57%) | 145 (4.32%) | 0.958791 | 0.999992 |
| 903 | [GO:0009166](file:///E:\2018-7-3%E7%81%AB%E9%BE%99%E6%9E%9C%E8%BD%AC%E5%BD%95%E7%BB%84%E6%B5%8B%E5%BA%8F\%E5%AE%8C%E6%95%B4%E7%89%88%E6%95%B0%E6%8D%AE\GDR3855-Hylocereus_undulatus_Britt-12-RNAseq_result\4_Function\2_Group_Diff_Function\UP_DOWN\GO\NL-VS-L1.P.html#gene903) | nucleotide catabolic process | 44 (3.57%) | 145 (4.32%) | 0.958791 | 0.999992 |
| 904 | [GO:0009261](file:///E:\2018-7-3%E7%81%AB%E9%BE%99%E6%9E%9C%E8%BD%AC%E5%BD%95%E7%BB%84%E6%B5%8B%E5%BA%8F\%E5%AE%8C%E6%95%B4%E7%89%88%E6%95%B0%E6%8D%AE\GDR3855-Hylocereus_undulatus_Britt-12-RNAseq_result\4_Function\2_Group_Diff_Function\UP_DOWN\GO\NL-VS-L1.P.html#gene904) | ribonucleotide catabolic process | 44 (3.57%) | 145 (4.32%) | 0.958791 | 0.999992 |
| 905 | [GO:0046434](file:///E:\2018-7-3%E7%81%AB%E9%BE%99%E6%9E%9C%E8%BD%AC%E5%BD%95%E7%BB%84%E6%B5%8B%E5%BA%8F\%E5%AE%8C%E6%95%B4%E7%89%88%E6%95%B0%E6%8D%AE\GDR3855-Hylocereus_undulatus_Britt-12-RNAseq_result\4_Function\2_Group_Diff_Function\UP_DOWN\GO\NL-VS-L1.P.html#gene905) | organophosphate catabolic process | 44 (3.57%) | 145 (4.32%) | 0.958791 | 0.999992 |
| 906 | [GO:0072523](file:///E:\2018-7-3%E7%81%AB%E9%BE%99%E6%9E%9C%E8%BD%AC%E5%BD%95%E7%BB%84%E6%B5%8B%E5%BA%8F\%E5%AE%8C%E6%95%B4%E7%89%88%E6%95%B0%E6%8D%AE\GDR3855-Hylocereus_undulatus_Britt-12-RNAseq_result\4_Function\2_Group_Diff_Function\UP_DOWN\GO\NL-VS-L1.P.html#gene906) | purine-containing compound catabolic process | 44 (3.57%) | 145 (4.32%) | 0.958791 | 0.999992 |
| 907 | [GO:1901292](file:///E:\2018-7-3%E7%81%AB%E9%BE%99%E6%9E%9C%E8%BD%AC%E5%BD%95%E7%BB%84%E6%B5%8B%E5%BA%8F\%E5%AE%8C%E6%95%B4%E7%89%88%E6%95%B0%E6%8D%AE\GDR3855-Hylocereus_undulatus_Britt-12-RNAseq_result\4_Function\2_Group_Diff_Function\UP_DOWN\GO\NL-VS-L1.P.html#gene907) | nucleoside phosphate catabolic process | 44 (3.57%) | 145 (4.32%) | 0.958791 | 0.999992 |
| 908 | [GO:0006081](file:///E:\2018-7-3%E7%81%AB%E9%BE%99%E6%9E%9C%E8%BD%AC%E5%BD%95%E7%BB%84%E6%B5%8B%E5%BA%8F\%E5%AE%8C%E6%95%B4%E7%89%88%E6%95%B0%E6%8D%AE\GDR3855-Hylocereus_undulatus_Britt-12-RNAseq_result\4_Function\2_Group_Diff_Function\UP_DOWN\GO\NL-VS-L1.P.html#gene908) | cellular aldehyde metabolic process | 1 (0.08%) | 7 (0.21%) | 0.959592 | 0.999992 |
| 909 | [GO:0006816](file:///E:\2018-7-3%E7%81%AB%E9%BE%99%E6%9E%9C%E8%BD%AC%E5%BD%95%E7%BB%84%E6%B5%8B%E5%BA%8F\%E5%AE%8C%E6%95%B4%E7%89%88%E6%95%B0%E6%8D%AE\GDR3855-Hylocereus_undulatus_Britt-12-RNAseq_result\4_Function\2_Group_Diff_Function\UP_DOWN\GO\NL-VS-L1.P.html#gene909) | calcium ion transport | 1 (0.08%) | 7 (0.21%) | 0.959592 | 0.999992 |
| 910 | [GO:0009147](file:///E:\2018-7-3%E7%81%AB%E9%BE%99%E6%9E%9C%E8%BD%AC%E5%BD%95%E7%BB%84%E6%B5%8B%E5%BA%8F\%E5%AE%8C%E6%95%B4%E7%89%88%E6%95%B0%E6%8D%AE\GDR3855-Hylocereus_undulatus_Britt-12-RNAseq_result\4_Function\2_Group_Diff_Function\UP_DOWN\GO\NL-VS-L1.P.html#gene910) | pyrimidine nucleoside triphosphate metabolic process | 1 (0.08%) | 7 (0.21%) | 0.959592 | 0.999992 |
| 911 | [GO:0009148](file:///E:\2018-7-3%E7%81%AB%E9%BE%99%E6%9E%9C%E8%BD%AC%E5%BD%95%E7%BB%84%E6%B5%8B%E5%BA%8F\%E5%AE%8C%E6%95%B4%E7%89%88%E6%95%B0%E6%8D%AE\GDR3855-Hylocereus_undulatus_Britt-12-RNAseq_result\4_Function\2_Group_Diff_Function\UP_DOWN\GO\NL-VS-L1.P.html#gene911) | pyrimidine nucleoside triphosphate biosynthetic process | 1 (0.08%) | 7 (0.21%) | 0.959592 | 0.999992 |
| 912 | [GO:0009208](file:///E:\2018-7-3%E7%81%AB%E9%BE%99%E6%9E%9C%E8%BD%AC%E5%BD%95%E7%BB%84%E6%B5%8B%E5%BA%8F\%E5%AE%8C%E6%95%B4%E7%89%88%E6%95%B0%E6%8D%AE\GDR3855-Hylocereus_undulatus_Britt-12-RNAseq_result\4_Function\2_Group_Diff_Function\UP_DOWN\GO\NL-VS-L1.P.html#gene912) | pyrimidine ribonucleoside triphosphate metabolic process | 1 (0.08%) | 7 (0.21%) | 0.959592 | 0.999992 |
| 913 | [GO:0009209](file:///E:\2018-7-3%E7%81%AB%E9%BE%99%E6%9E%9C%E8%BD%AC%E5%BD%95%E7%BB%84%E6%B5%8B%E5%BA%8F\%E5%AE%8C%E6%95%B4%E7%89%88%E6%95%B0%E6%8D%AE\GDR3855-Hylocereus_undulatus_Britt-12-RNAseq_result\4_Function\2_Group_Diff_Function\UP_DOWN\GO\NL-VS-L1.P.html#gene913) | pyrimidine ribonucleoside triphosphate biosynthetic process | 1 (0.08%) | 7 (0.21%) | 0.959592 | 0.999992 |
| 914 | [GO:0019362](file:///E:\2018-7-3%E7%81%AB%E9%BE%99%E6%9E%9C%E8%BD%AC%E5%BD%95%E7%BB%84%E6%B5%8B%E5%BA%8F\%E5%AE%8C%E6%95%B4%E7%89%88%E6%95%B0%E6%8D%AE\GDR3855-Hylocereus_undulatus_Britt-12-RNAseq_result\4_Function\2_Group_Diff_Function\UP_DOWN\GO\NL-VS-L1.P.html#gene914) | pyridine nucleotide metabolic process | 1 (0.08%) | 7 (0.21%) | 0.959592 | 0.999992 |
| 915 | [GO:0034470](file:///E:\2018-7-3%E7%81%AB%E9%BE%99%E6%9E%9C%E8%BD%AC%E5%BD%95%E7%BB%84%E6%B5%8B%E5%BA%8F\%E5%AE%8C%E6%95%B4%E7%89%88%E6%95%B0%E6%8D%AE\GDR3855-Hylocereus_undulatus_Britt-12-RNAseq_result\4_Function\2_Group_Diff_Function\UP_DOWN\GO\NL-VS-L1.P.html#gene915) | ncRNA processing | 1 (0.08%) | 7 (0.21%) | 0.959592 | 0.999992 |
| 916 | [GO:1901293](file:///E:\2018-7-3%E7%81%AB%E9%BE%99%E6%9E%9C%E8%BD%AC%E5%BD%95%E7%BB%84%E6%B5%8B%E5%BA%8F\%E5%AE%8C%E6%95%B4%E7%89%88%E6%95%B0%E6%8D%AE\GDR3855-Hylocereus_undulatus_Britt-12-RNAseq_result\4_Function\2_Group_Diff_Function\UP_DOWN\GO\NL-VS-L1.P.html#gene916) | nucleoside phosphate biosynthetic process | 14 (1.13%) | 53 (1.58%) | 0.959630 | 0.999992 |
| 917 | [GO:0044699](file:///E:\2018-7-3%E7%81%AB%E9%BE%99%E6%9E%9C%E8%BD%AC%E5%BD%95%E7%BB%84%E6%B5%8B%E5%BA%8F\%E5%AE%8C%E6%95%B4%E7%89%88%E6%95%B0%E6%8D%AE\GDR3855-Hylocereus_undulatus_Britt-12-RNAseq_result\4_Function\2_Group_Diff_Function\UP_DOWN\GO\NL-VS-L1.P.html#gene917) | single-organism process | 605 (49.03%) | 1712 (50.97%) | 0.959917 | 0.999992 |
| 918 | [GO:0009260](file:///E:\2018-7-3%E7%81%AB%E9%BE%99%E6%9E%9C%E8%BD%AC%E5%BD%95%E7%BB%84%E6%B5%8B%E5%BA%8F\%E5%AE%8C%E6%95%B4%E7%89%88%E6%95%B0%E6%8D%AE\GDR3855-Hylocereus_undulatus_Britt-12-RNAseq_result\4_Function\2_Group_Diff_Function\UP_DOWN\GO\NL-VS-L1.P.html#gene918) | ribonucleotide biosynthetic process | 10 (0.81%) | 40 (1.19%) | 0.960131 | 0.999992 |
| 919 | [GO:0046390](file:///E:\2018-7-3%E7%81%AB%E9%BE%99%E6%9E%9C%E8%BD%AC%E5%BD%95%E7%BB%84%E6%B5%8B%E5%BA%8F\%E5%AE%8C%E6%95%B4%E7%89%88%E6%95%B0%E6%8D%AE\GDR3855-Hylocereus_undulatus_Britt-12-RNAseq_result\4_Function\2_Group_Diff_Function\UP_DOWN\GO\NL-VS-L1.P.html#gene919) | ribose phosphate biosynthetic process | 10 (0.81%) | 40 (1.19%) | 0.960131 | 0.999992 |
| 920 | [GO:0000003](file:///E:\2018-7-3%E7%81%AB%E9%BE%99%E6%9E%9C%E8%BD%AC%E5%BD%95%E7%BB%84%E6%B5%8B%E5%BA%8F\%E5%AE%8C%E6%95%B4%E7%89%88%E6%95%B0%E6%8D%AE\GDR3855-Hylocereus_undulatus_Britt-12-RNAseq_result\4_Function\2_Group_Diff_Function\UP_DOWN\GO\NL-VS-L1.P.html#gene920) | reproduction | 27 (2.19%) | 94 (2.8%) | 0.961305 | 0.999992 |
| 921 | [GO:0015031](file:///E:\2018-7-3%E7%81%AB%E9%BE%99%E6%9E%9C%E8%BD%AC%E5%BD%95%E7%BB%84%E6%B5%8B%E5%BA%8F\%E5%AE%8C%E6%95%B4%E7%89%88%E6%95%B0%E6%8D%AE\GDR3855-Hylocereus_undulatus_Britt-12-RNAseq_result\4_Function\2_Group_Diff_Function\UP_DOWN\GO\NL-VS-L1.P.html#gene921) | protein transport | 24 (1.94%) | 85 (2.53%) | 0.962951 | 0.999992 |
| 922 | [GO:1901657](file:///E:\2018-7-3%E7%81%AB%E9%BE%99%E6%9E%9C%E8%BD%AC%E5%BD%95%E7%BB%84%E6%B5%8B%E5%BA%8F\%E5%AE%8C%E6%95%B4%E7%89%88%E6%95%B0%E6%8D%AE\GDR3855-Hylocereus_undulatus_Britt-12-RNAseq_result\4_Function\2_Group_Diff_Function\UP_DOWN\GO\NL-VS-L1.P.html#gene922) | glycosyl compound metabolic process | 6 (0.49%) | 27 (0.8%) | 0.966182 | 0.999992 |
| 923 | [GO:1901659](file:///E:\2018-7-3%E7%81%AB%E9%BE%99%E6%9E%9C%E8%BD%AC%E5%BD%95%E7%BB%84%E6%B5%8B%E5%BA%8F\%E5%AE%8C%E6%95%B4%E7%89%88%E6%95%B0%E6%8D%AE\GDR3855-Hylocereus_undulatus_Britt-12-RNAseq_result\4_Function\2_Group_Diff_Function\UP_DOWN\GO\NL-VS-L1.P.html#gene923) | glycosyl compound biosynthetic process | 6 (0.49%) | 27 (0.8%) | 0.966182 | 0.999992 |
| 924 | [GO:0009069](file:///E:\2018-7-3%E7%81%AB%E9%BE%99%E6%9E%9C%E8%BD%AC%E5%BD%95%E7%BB%84%E6%B5%8B%E5%BA%8F\%E5%AE%8C%E6%95%B4%E7%89%88%E6%95%B0%E6%8D%AE\GDR3855-Hylocereus_undulatus_Britt-12-RNAseq_result\4_Function\2_Group_Diff_Function\UP_DOWN\GO\NL-VS-L1.P.html#gene924) | serine family amino acid metabolic process | 3 (0.24%) | 16 (0.48%) | 0.966897 | 0.999992 |
| 925 | [GO:0090501](file:///E:\2018-7-3%E7%81%AB%E9%BE%99%E6%9E%9C%E8%BD%AC%E5%BD%95%E7%BB%84%E6%B5%8B%E5%BA%8F\%E5%AE%8C%E6%95%B4%E7%89%88%E6%95%B0%E6%8D%AE\GDR3855-Hylocereus_undulatus_Britt-12-RNAseq_result\4_Function\2_Group_Diff_Function\UP_DOWN\GO\NL-VS-L1.P.html#gene925) | RNA phosphodiester bond hydrolysis | 3 (0.24%) | 16 (0.48%) | 0.966897 | 0.999992 |
| 926 | [GO:0006418](file:///E:\2018-7-3%E7%81%AB%E9%BE%99%E6%9E%9C%E8%BD%AC%E5%BD%95%E7%BB%84%E6%B5%8B%E5%BA%8F\%E5%AE%8C%E6%95%B4%E7%89%88%E6%95%B0%E6%8D%AE\GDR3855-Hylocereus_undulatus_Britt-12-RNAseq_result\4_Function\2_Group_Diff_Function\UP_DOWN\GO\NL-VS-L1.P.html#gene926) | tRNA aminoacylation for protein translation | 2 (0.16%) | 12 (0.36%) | 0.967479 | 0.999992 |
| 927 | [GO:0009266](file:///E:\2018-7-3%E7%81%AB%E9%BE%99%E6%9E%9C%E8%BD%AC%E5%BD%95%E7%BB%84%E6%B5%8B%E5%BA%8F\%E5%AE%8C%E6%95%B4%E7%89%88%E6%95%B0%E6%8D%AE\GDR3855-Hylocereus_undulatus_Britt-12-RNAseq_result\4_Function\2_Group_Diff_Function\UP_DOWN\GO\NL-VS-L1.P.html#gene927) | response to temperature stimulus | 2 (0.16%) | 12 (0.36%) | 0.967479 | 0.999992 |
| 928 | [GO:0033365](file:///E:\2018-7-3%E7%81%AB%E9%BE%99%E6%9E%9C%E8%BD%AC%E5%BD%95%E7%BB%84%E6%B5%8B%E5%BA%8F\%E5%AE%8C%E6%95%B4%E7%89%88%E6%95%B0%E6%8D%AE\GDR3855-Hylocereus_undulatus_Britt-12-RNAseq_result\4_Function\2_Group_Diff_Function\UP_DOWN\GO\NL-VS-L1.P.html#gene928) | protein localization to organelle | 2 (0.16%) | 12 (0.36%) | 0.967479 | 0.999992 |
| 929 | [GO:0043038](file:///E:\2018-7-3%E7%81%AB%E9%BE%99%E6%9E%9C%E8%BD%AC%E5%BD%95%E7%BB%84%E6%B5%8B%E5%BA%8F\%E5%AE%8C%E6%95%B4%E7%89%88%E6%95%B0%E6%8D%AE\GDR3855-Hylocereus_undulatus_Britt-12-RNAseq_result\4_Function\2_Group_Diff_Function\UP_DOWN\GO\NL-VS-L1.P.html#gene929) | amino acid activation | 2 (0.16%) | 12 (0.36%) | 0.967479 | 0.999992 |
| 930 | [GO:0043039](file:///E:\2018-7-3%E7%81%AB%E9%BE%99%E6%9E%9C%E8%BD%AC%E5%BD%95%E7%BB%84%E6%B5%8B%E5%BA%8F\%E5%AE%8C%E6%95%B4%E7%89%88%E6%95%B0%E6%8D%AE\GDR3855-Hylocereus_undulatus_Britt-12-RNAseq_result\4_Function\2_Group_Diff_Function\UP_DOWN\GO\NL-VS-L1.P.html#gene930) | tRNA aminoacylation | 2 (0.16%) | 12 (0.36%) | 0.967479 | 0.999992 |
| 931 | [GO:0072594](file:///E:\2018-7-3%E7%81%AB%E9%BE%99%E6%9E%9C%E8%BD%AC%E5%BD%95%E7%BB%84%E6%B5%8B%E5%BA%8F\%E5%AE%8C%E6%95%B4%E7%89%88%E6%95%B0%E6%8D%AE\GDR3855-Hylocereus_undulatus_Britt-12-RNAseq_result\4_Function\2_Group_Diff_Function\UP_DOWN\GO\NL-VS-L1.P.html#gene931) | establishment of protein localization to organelle | 2 (0.16%) | 12 (0.36%) | 0.967479 | 0.999992 |
| 932 | [GO:0048731](file:///E:\2018-7-3%E7%81%AB%E9%BE%99%E6%9E%9C%E8%BD%AC%E5%BD%95%E7%BB%84%E6%B5%8B%E5%BA%8F\%E5%AE%8C%E6%95%B4%E7%89%88%E6%95%B0%E6%8D%AE\GDR3855-Hylocereus_undulatus_Britt-12-RNAseq_result\4_Function\2_Group_Diff_Function\UP_DOWN\GO\NL-VS-L1.P.html#gene932) | system development | 18 (1.46%) | 67 (1.99%) | 0.968115 | 0.999992 |
| 933 | [GO:0006163](file:///E:\2018-7-3%E7%81%AB%E9%BE%99%E6%9E%9C%E8%BD%AC%E5%BD%95%E7%BB%84%E6%B5%8B%E5%BA%8F\%E5%AE%8C%E6%95%B4%E7%89%88%E6%95%B0%E6%8D%AE\GDR3855-Hylocereus_undulatus_Britt-12-RNAseq_result\4_Function\2_Group_Diff_Function\UP_DOWN\GO\NL-VS-L1.P.html#gene933) | purine nucleotide metabolic process | 57 (4.62%) | 186 (5.54%) | 0.969183 | 0.999992 |
| 934 | [GO:0032502](file:///E:\2018-7-3%E7%81%AB%E9%BE%99%E6%9E%9C%E8%BD%AC%E5%BD%95%E7%BB%84%E6%B5%8B%E5%BA%8F\%E5%AE%8C%E6%95%B4%E7%89%88%E6%95%B0%E6%8D%AE\GDR3855-Hylocereus_undulatus_Britt-12-RNAseq_result\4_Function\2_Group_Diff_Function\UP_DOWN\GO\NL-VS-L1.P.html#gene934) | developmental process | 57 (4.62%) | 186 (5.54%) | 0.969183 | 0.999992 |
| 935 | [GO:0090407](file:///E:\2018-7-3%E7%81%AB%E9%BE%99%E6%9E%9C%E8%BD%AC%E5%BD%95%E7%BB%84%E6%B5%8B%E5%BA%8F\%E5%AE%8C%E6%95%B4%E7%89%88%E6%95%B0%E6%8D%AE\GDR3855-Hylocereus_undulatus_Britt-12-RNAseq_result\4_Function\2_Group_Diff_Function\UP_DOWN\GO\NL-VS-L1.P.html#gene935) | organophosphate biosynthetic process | 17 (1.38%) | 64 (1.91%) | 0.969277 | 0.999992 |
| 936 | [GO:0050794](file:///E:\2018-7-3%E7%81%AB%E9%BE%99%E6%9E%9C%E8%BD%AC%E5%BD%95%E7%BB%84%E6%B5%8B%E5%BA%8F\%E5%AE%8C%E6%95%B4%E7%89%88%E6%95%B0%E6%8D%AE\GDR3855-Hylocereus_undulatus_Britt-12-RNAseq_result\4_Function\2_Group_Diff_Function\UP_DOWN\GO\NL-VS-L1.P.html#gene936) | regulation of cellular process | 79 (6.4%) | 251 (7.47%) | 0.969993 | 0.999992 |
| 937 | [GO:0032446](file:///E:\2018-7-3%E7%81%AB%E9%BE%99%E6%9E%9C%E8%BD%AC%E5%BD%95%E7%BB%84%E6%B5%8B%E5%BA%8F\%E5%AE%8C%E6%95%B4%E7%89%88%E6%95%B0%E6%8D%AE\GDR3855-Hylocereus_undulatus_Britt-12-RNAseq_result\4_Function\2_Group_Diff_Function\UP_DOWN\GO\NL-VS-L1.P.html#gene937) | protein modification by small protein conjugation | 9 (0.73%) | 38 (1.13%) | 0.971174 | 0.999992 |
| 938 | [GO:0034655](file:///E:\2018-7-3%E7%81%AB%E9%BE%99%E6%9E%9C%E8%BD%AC%E5%BD%95%E7%BB%84%E6%B5%8B%E5%BA%8F\%E5%AE%8C%E6%95%B4%E7%89%88%E6%95%B0%E6%8D%AE\GDR3855-Hylocereus_undulatus_Britt-12-RNAseq_result\4_Function\2_Group_Diff_Function\UP_DOWN\GO\NL-VS-L1.P.html#gene938) | nucleobase-containing compound catabolic process | 45 (3.65%) | 151 (4.5%) | 0.972378 | 0.999992 |
| 939 | [GO:1901565](file:///E:\2018-7-3%E7%81%AB%E9%BE%99%E6%9E%9C%E8%BD%AC%E5%BD%95%E7%BB%84%E6%B5%8B%E5%BA%8F\%E5%AE%8C%E6%95%B4%E7%89%88%E6%95%B0%E6%8D%AE\GDR3855-Hylocereus_undulatus_Britt-12-RNAseq_result\4_Function\2_Group_Diff_Function\UP_DOWN\GO\NL-VS-L1.P.html#gene939) | organonitrogen compound catabolic process | 45 (3.65%) | 151 (4.5%) | 0.972378 | 0.999992 |
| 940 | [GO:0009150](file:///E:\2018-7-3%E7%81%AB%E9%BE%99%E6%9E%9C%E8%BD%AC%E5%BD%95%E7%BB%84%E6%B5%8B%E5%BA%8F\%E5%AE%8C%E6%95%B4%E7%89%88%E6%95%B0%E6%8D%AE\GDR3855-Hylocereus_undulatus_Britt-12-RNAseq_result\4_Function\2_Group_Diff_Function\UP_DOWN\GO\NL-VS-L1.P.html#gene940) | purine ribonucleotide metabolic process | 56 (4.54%) | 184 (5.48%) | 0.972678 | 0.999992 |
| 941 | [GO:0072521](file:///E:\2018-7-3%E7%81%AB%E9%BE%99%E6%9E%9C%E8%BD%AC%E5%BD%95%E7%BB%84%E6%B5%8B%E5%BA%8F\%E5%AE%8C%E6%95%B4%E7%89%88%E6%95%B0%E6%8D%AE\GDR3855-Hylocereus_undulatus_Britt-12-RNAseq_result\4_Function\2_Group_Diff_Function\UP_DOWN\GO\NL-VS-L1.P.html#gene941) | purine-containing compound metabolic process | 57 (4.62%) | 187 (5.57%) | 0.972762 | 0.999992 |
| 942 | [GO:0032501](file:///E:\2018-7-3%E7%81%AB%E9%BE%99%E6%9E%9C%E8%BD%AC%E5%BD%95%E7%BB%84%E6%B5%8B%E5%BA%8F\%E5%AE%8C%E6%95%B4%E7%89%88%E6%95%B0%E6%8D%AE\GDR3855-Hylocereus_undulatus_Britt-12-RNAseq_result\4_Function\2_Group_Diff_Function\UP_DOWN\GO\NL-VS-L1.P.html#gene942) | multicellular organismal process | 39 (3.16%) | 133 (3.96%) | 0.972897 | 0.999992 |
| 943 | [GO:0010467](file:///E:\2018-7-3%E7%81%AB%E9%BE%99%E6%9E%9C%E8%BD%AC%E5%BD%95%E7%BB%84%E6%B5%8B%E5%BA%8F\%E5%AE%8C%E6%95%B4%E7%89%88%E6%95%B0%E6%8D%AE\GDR3855-Hylocereus_undulatus_Britt-12-RNAseq_result\4_Function\2_Group_Diff_Function\UP_DOWN\GO\NL-VS-L1.P.html#gene943) | gene expression | 171 (13.86%) | 517 (15.39%) | 0.973579 | 0.999992 |
| 944 | [GO:1901135](file:///E:\2018-7-3%E7%81%AB%E9%BE%99%E6%9E%9C%E8%BD%AC%E5%BD%95%E7%BB%84%E6%B5%8B%E5%BA%8F\%E5%AE%8C%E6%95%B4%E7%89%88%E6%95%B0%E6%8D%AE\GDR3855-Hylocereus_undulatus_Britt-12-RNAseq_result\4_Function\2_Group_Diff_Function\UP_DOWN\GO\NL-VS-L1.P.html#gene944) | carbohydrate derivative metabolic process | 65 (5.27%) | 211 (6.28%) | 0.973671 | 0.999992 |
| 945 | [GO:0009117](file:///E:\2018-7-3%E7%81%AB%E9%BE%99%E6%9E%9C%E8%BD%AC%E5%BD%95%E7%BB%84%E6%B5%8B%E5%BA%8F\%E5%AE%8C%E6%95%B4%E7%89%88%E6%95%B0%E6%8D%AE\GDR3855-Hylocereus_undulatus_Britt-12-RNAseq_result\4_Function\2_Group_Diff_Function\UP_DOWN\GO\NL-VS-L1.P.html#gene945) | nucleotide metabolic process | 66 (5.35%) | 214 (6.37%) | 0.973809 | 0.999992 |
| 946 | [GO:0019941](file:///E:\2018-7-3%E7%81%AB%E9%BE%99%E6%9E%9C%E8%BD%AC%E5%BD%95%E7%BB%84%E6%B5%8B%E5%BA%8F\%E5%AE%8C%E6%95%B4%E7%89%88%E6%95%B0%E6%8D%AE\GDR3855-Hylocereus_undulatus_Britt-12-RNAseq_result\4_Function\2_Group_Diff_Function\UP_DOWN\GO\NL-VS-L1.P.html#gene946) | modification-dependent protein catabolic process | 8 (0.65%) | 35 (1.04%) | 0.974167 | 0.999992 |
| 947 | [GO:0043632](file:///E:\2018-7-3%E7%81%AB%E9%BE%99%E6%9E%9C%E8%BD%AC%E5%BD%95%E7%BB%84%E6%B5%8B%E5%BA%8F\%E5%AE%8C%E6%95%B4%E7%89%88%E6%95%B0%E6%8D%AE\GDR3855-Hylocereus_undulatus_Britt-12-RNAseq_result\4_Function\2_Group_Diff_Function\UP_DOWN\GO\NL-VS-L1.P.html#gene947) | modification-dependent macromolecule catabolic process | 8 (0.65%) | 35 (1.04%) | 0.974167 | 0.999992 |
| 948 | [GO:0006595](file:///E:\2018-7-3%E7%81%AB%E9%BE%99%E6%9E%9C%E8%BD%AC%E5%BD%95%E7%BB%84%E6%B5%8B%E5%BA%8F\%E5%AE%8C%E6%95%B4%E7%89%88%E6%95%B0%E6%8D%AE\GDR3855-Hylocereus_undulatus_Britt-12-RNAseq_result\4_Function\2_Group_Diff_Function\UP_DOWN\GO\NL-VS-L1.P.html#gene948) | polyamine metabolic process | 1 (0.08%) | 8 (0.24%) | 0.974468 | 0.999992 |
| 949 | [GO:0007059](file:///E:\2018-7-3%E7%81%AB%E9%BE%99%E6%9E%9C%E8%BD%AC%E5%BD%95%E7%BB%84%E6%B5%8B%E5%BA%8F\%E5%AE%8C%E6%95%B4%E7%89%88%E6%95%B0%E6%8D%AE\GDR3855-Hylocereus_undulatus_Britt-12-RNAseq_result\4_Function\2_Group_Diff_Function\UP_DOWN\GO\NL-VS-L1.P.html#gene949) | chromosome segregation | 1 (0.08%) | 8 (0.24%) | 0.974468 | 0.999992 |
| 950 | [GO:0010212](file:///E:\2018-7-3%E7%81%AB%E9%BE%99%E6%9E%9C%E8%BD%AC%E5%BD%95%E7%BB%84%E6%B5%8B%E5%BA%8F\%E5%AE%8C%E6%95%B4%E7%89%88%E6%95%B0%E6%8D%AE\GDR3855-Hylocereus_undulatus_Britt-12-RNAseq_result\4_Function\2_Group_Diff_Function\UP_DOWN\GO\NL-VS-L1.P.html#gene950) | response to ionizing radiation | 1 (0.08%) | 8 (0.24%) | 0.974468 | 0.999992 |
| 951 | [GO:0017004](file:///E:\2018-7-3%E7%81%AB%E9%BE%99%E6%9E%9C%E8%BD%AC%E5%BD%95%E7%BB%84%E6%B5%8B%E5%BA%8F\%E5%AE%8C%E6%95%B4%E7%89%88%E6%95%B0%E6%8D%AE\GDR3855-Hylocereus_undulatus_Britt-12-RNAseq_result\4_Function\2_Group_Diff_Function\UP_DOWN\GO\NL-VS-L1.P.html#gene951) | cytochrome complex assembly | 1 (0.08%) | 8 (0.24%) | 0.974468 | 0.999992 |
| 952 | [GO:0009126](file:///E:\2018-7-3%E7%81%AB%E9%BE%99%E6%9E%9C%E8%BD%AC%E5%BD%95%E7%BB%84%E6%B5%8B%E5%BA%8F\%E5%AE%8C%E6%95%B4%E7%89%88%E6%95%B0%E6%8D%AE\GDR3855-Hylocereus_undulatus_Britt-12-RNAseq_result\4_Function\2_Group_Diff_Function\UP_DOWN\GO\NL-VS-L1.P.html#gene952) | purine nucleoside monophosphate metabolic process | 6 (0.49%) | 28 (0.83%) | 0.974486 | 0.999992 |
| 953 | [GO:0009127](file:///E:\2018-7-3%E7%81%AB%E9%BE%99%E6%9E%9C%E8%BD%AC%E5%BD%95%E7%BB%84%E6%B5%8B%E5%BA%8F\%E5%AE%8C%E6%95%B4%E7%89%88%E6%95%B0%E6%8D%AE\GDR3855-Hylocereus_undulatus_Britt-12-RNAseq_result\4_Function\2_Group_Diff_Function\UP_DOWN\GO\NL-VS-L1.P.html#gene953) | purine nucleoside monophosphate biosynthetic process | 6 (0.49%) | 28 (0.83%) | 0.974486 | 0.999992 |
| 954 | [GO:0009167](file:///E:\2018-7-3%E7%81%AB%E9%BE%99%E6%9E%9C%E8%BD%AC%E5%BD%95%E7%BB%84%E6%B5%8B%E5%BA%8F\%E5%AE%8C%E6%95%B4%E7%89%88%E6%95%B0%E6%8D%AE\GDR3855-Hylocereus_undulatus_Britt-12-RNAseq_result\4_Function\2_Group_Diff_Function\UP_DOWN\GO\NL-VS-L1.P.html#gene954) | purine ribonucleoside monophosphate metabolic process | 6 (0.49%) | 28 (0.83%) | 0.974486 | 0.999992 |
| 955 | [GO:0009168](file:///E:\2018-7-3%E7%81%AB%E9%BE%99%E6%9E%9C%E8%BD%AC%E5%BD%95%E7%BB%84%E6%B5%8B%E5%BA%8F\%E5%AE%8C%E6%95%B4%E7%89%88%E6%95%B0%E6%8D%AE\GDR3855-Hylocereus_undulatus_Britt-12-RNAseq_result\4_Function\2_Group_Diff_Function\UP_DOWN\GO\NL-VS-L1.P.html#gene955) | purine ribonucleoside monophosphate biosynthetic process | 6 (0.49%) | 28 (0.83%) | 0.974486 | 0.999992 |
| 956 | [GO:0016482](file:///E:\2018-7-3%E7%81%AB%E9%BE%99%E6%9E%9C%E8%BD%AC%E5%BD%95%E7%BB%84%E6%B5%8B%E5%BA%8F\%E5%AE%8C%E6%95%B4%E7%89%88%E6%95%B0%E6%8D%AE\GDR3855-Hylocereus_undulatus_Britt-12-RNAseq_result\4_Function\2_Group_Diff_Function\UP_DOWN\GO\NL-VS-L1.P.html#gene956) | cytoplasmic transport | 6 (0.49%) | 28 (0.83%) | 0.974486 | 0.999992 |
| 957 | [GO:0051188](file:///E:\2018-7-3%E7%81%AB%E9%BE%99%E6%9E%9C%E8%BD%AC%E5%BD%95%E7%BB%84%E6%B5%8B%E5%BA%8F\%E5%AE%8C%E6%95%B4%E7%89%88%E6%95%B0%E6%8D%AE\GDR3855-Hylocereus_undulatus_Britt-12-RNAseq_result\4_Function\2_Group_Diff_Function\UP_DOWN\GO\NL-VS-L1.P.html#gene957) | cofactor biosynthetic process | 6 (0.49%) | 28 (0.83%) | 0.974486 | 0.999992 |
| 958 | [GO:0019439](file:///E:\2018-7-3%E7%81%AB%E9%BE%99%E6%9E%9C%E8%BD%AC%E5%BD%95%E7%BB%84%E6%B5%8B%E5%BA%8F\%E5%AE%8C%E6%95%B4%E7%89%88%E6%95%B0%E6%8D%AE\GDR3855-Hylocereus_undulatus_Britt-12-RNAseq_result\4_Function\2_Group_Diff_Function\UP_DOWN\GO\NL-VS-L1.P.html#gene958) | aromatic compound catabolic process | 45 (3.65%) | 152 (4.53%) | 0.975929 | 0.999992 |
| 959 | [GO:0006753](file:///E:\2018-7-3%E7%81%AB%E9%BE%99%E6%9E%9C%E8%BD%AC%E5%BD%95%E7%BB%84%E6%B5%8B%E5%BA%8F\%E5%AE%8C%E6%95%B4%E7%89%88%E6%95%B0%E6%8D%AE\GDR3855-Hylocereus_undulatus_Britt-12-RNAseq_result\4_Function\2_Group_Diff_Function\UP_DOWN\GO\NL-VS-L1.P.html#gene959) | nucleoside phosphate metabolic process | 67 (5.43%) | 218 (6.49%) | 0.976860 | 0.999992 |
| 960 | [GO:0030163](file:///E:\2018-7-3%E7%81%AB%E9%BE%99%E6%9E%9C%E8%BD%AC%E5%BD%95%E7%BB%84%E6%B5%8B%E5%BA%8F\%E5%AE%8C%E6%95%B4%E7%89%88%E6%95%B0%E6%8D%AE\GDR3855-Hylocereus_undulatus_Britt-12-RNAseq_result\4_Function\2_Group_Diff_Function\UP_DOWN\GO\NL-VS-L1.P.html#gene960) | protein catabolic process | 9 (0.73%) | 39 (1.16%) | 0.977541 | 0.999992 |
| 961 | [GO:0044257](file:///E:\2018-7-3%E7%81%AB%E9%BE%99%E6%9E%9C%E8%BD%AC%E5%BD%95%E7%BB%84%E6%B5%8B%E5%BA%8F\%E5%AE%8C%E6%95%B4%E7%89%88%E6%95%B0%E6%8D%AE\GDR3855-Hylocereus_undulatus_Britt-12-RNAseq_result\4_Function\2_Group_Diff_Function\UP_DOWN\GO\NL-VS-L1.P.html#gene961) | cellular protein catabolic process | 9 (0.73%) | 39 (1.16%) | 0.977541 | 0.999992 |
| 962 | [GO:0009141](file:///E:\2018-7-3%E7%81%AB%E9%BE%99%E6%9E%9C%E8%BD%AC%E5%BD%95%E7%BB%84%E6%B5%8B%E5%BA%8F\%E5%AE%8C%E6%95%B4%E7%89%88%E6%95%B0%E6%8D%AE\GDR3855-Hylocereus_undulatus_Britt-12-RNAseq_result\4_Function\2_Group_Diff_Function\UP_DOWN\GO\NL-VS-L1.P.html#gene962) | nucleoside triphosphate metabolic process | 5 (0.41%) | 25 (0.74%) | 0.978938 | 0.999992 |
| 963 | [GO:0009142](file:///E:\2018-7-3%E7%81%AB%E9%BE%99%E6%9E%9C%E8%BD%AC%E5%BD%95%E7%BB%84%E6%B5%8B%E5%BA%8F\%E5%AE%8C%E6%95%B4%E7%89%88%E6%95%B0%E6%8D%AE\GDR3855-Hylocereus_undulatus_Britt-12-RNAseq_result\4_Function\2_Group_Diff_Function\UP_DOWN\GO\NL-VS-L1.P.html#gene963) | nucleoside triphosphate biosynthetic process | 5 (0.41%) | 25 (0.74%) | 0.978938 | 0.999992 |
| 964 | [GO:0009199](file:///E:\2018-7-3%E7%81%AB%E9%BE%99%E6%9E%9C%E8%BD%AC%E5%BD%95%E7%BB%84%E6%B5%8B%E5%BA%8F\%E5%AE%8C%E6%95%B4%E7%89%88%E6%95%B0%E6%8D%AE\GDR3855-Hylocereus_undulatus_Britt-12-RNAseq_result\4_Function\2_Group_Diff_Function\UP_DOWN\GO\NL-VS-L1.P.html#gene964) | ribonucleoside triphosphate metabolic process | 5 (0.41%) | 25 (0.74%) | 0.978938 | 0.999992 |
| 965 | [GO:0009201](file:///E:\2018-7-3%E7%81%AB%E9%BE%99%E6%9E%9C%E8%BD%AC%E5%BD%95%E7%BB%84%E6%B5%8B%E5%BA%8F\%E5%AE%8C%E6%95%B4%E7%89%88%E6%95%B0%E6%8D%AE\GDR3855-Hylocereus_undulatus_Britt-12-RNAseq_result\4_Function\2_Group_Diff_Function\UP_DOWN\GO\NL-VS-L1.P.html#gene965) | ribonucleoside triphosphate biosynthetic process | 5 (0.41%) | 25 (0.74%) | 0.978938 | 0.999992 |
| 966 | [GO:0006351](file:///E:\2018-7-3%E7%81%AB%E9%BE%99%E6%9E%9C%E8%BD%AC%E5%BD%95%E7%BB%84%E6%B5%8B%E5%BA%8F\%E5%AE%8C%E6%95%B4%E7%89%88%E6%95%B0%E6%8D%AE\GDR3855-Hylocereus_undulatus_Britt-12-RNAseq_result\4_Function\2_Group_Diff_Function\UP_DOWN\GO\NL-VS-L1.P.html#gene966) | transcription, DNA-templated | 46 (3.73%) | 156 (4.64%) | 0.978998 | 0.999992 |
| 967 | [GO:0097659](file:///E:\2018-7-3%E7%81%AB%E9%BE%99%E6%9E%9C%E8%BD%AC%E5%BD%95%E7%BB%84%E6%B5%8B%E5%BA%8F\%E5%AE%8C%E6%95%B4%E7%89%88%E6%95%B0%E6%8D%AE\GDR3855-Hylocereus_undulatus_Britt-12-RNAseq_result\4_Function\2_Group_Diff_Function\UP_DOWN\GO\NL-VS-L1.P.html#gene967) | nucleic acid-templated transcription | 46 (3.73%) | 156 (4.64%) | 0.978998 | 0.999992 |
| 968 | [GO:1901137](file:///E:\2018-7-3%E7%81%AB%E9%BE%99%E6%9E%9C%E8%BD%AC%E5%BD%95%E7%BB%84%E6%B5%8B%E5%BA%8F\%E5%AE%8C%E6%95%B4%E7%89%88%E6%95%B0%E6%8D%AE\GDR3855-Hylocereus_undulatus_Britt-12-RNAseq_result\4_Function\2_Group_Diff_Function\UP_DOWN\GO\NL-VS-L1.P.html#gene968) | carbohydrate derivative biosynthetic process | 13 (1.05%) | 53 (1.58%) | 0.979976 | 0.999992 |
| 969 | [GO:0070647](file:///E:\2018-7-3%E7%81%AB%E9%BE%99%E6%9E%9C%E8%BD%AC%E5%BD%95%E7%BB%84%E6%B5%8B%E5%BA%8F\%E5%AE%8C%E6%95%B4%E7%89%88%E6%95%B0%E6%8D%AE\GDR3855-Hylocereus_undulatus_Britt-12-RNAseq_result\4_Function\2_Group_Diff_Function\UP_DOWN\GO\NL-VS-L1.P.html#gene969) | protein modification by small protein conjugation or removal | 10 (0.81%) | 43 (1.28%) | 0.980510 | 0.999992 |
| 970 | [GO:0022414](file:///E:\2018-7-3%E7%81%AB%E9%BE%99%E6%9E%9C%E8%BD%AC%E5%BD%95%E7%BB%84%E6%B5%8B%E5%BA%8F\%E5%AE%8C%E6%95%B4%E7%89%88%E6%95%B0%E6%8D%AE\GDR3855-Hylocereus_undulatus_Britt-12-RNAseq_result\4_Function\2_Group_Diff_Function\UP_DOWN\GO\NL-VS-L1.P.html#gene970) | reproductive process | 25 (2.03%) | 92 (2.74%) | 0.981055 | 0.999992 |
| 971 | [GO:0009259](file:///E:\2018-7-3%E7%81%AB%E9%BE%99%E6%9E%9C%E8%BD%AC%E5%BD%95%E7%BB%84%E6%B5%8B%E5%BA%8F\%E5%AE%8C%E6%95%B4%E7%89%88%E6%95%B0%E6%8D%AE\GDR3855-Hylocereus_undulatus_Britt-12-RNAseq_result\4_Function\2_Group_Diff_Function\UP_DOWN\GO\NL-VS-L1.P.html#gene971) | ribonucleotide metabolic process | 56 (4.54%) | 187 (5.57%) | 0.981414 | 0.999992 |
| 972 | [GO:0019693](file:///E:\2018-7-3%E7%81%AB%E9%BE%99%E6%9E%9C%E8%BD%AC%E5%BD%95%E7%BB%84%E6%B5%8B%E5%BA%8F\%E5%AE%8C%E6%95%B4%E7%89%88%E6%95%B0%E6%8D%AE\GDR3855-Hylocereus_undulatus_Britt-12-RNAseq_result\4_Function\2_Group_Diff_Function\UP_DOWN\GO\NL-VS-L1.P.html#gene972) | ribose phosphate metabolic process | 57 (4.62%) | 190 (5.66%) | 0.981424 | 0.999992 |
| 973 | [GO:0032774](file:///E:\2018-7-3%E7%81%AB%E9%BE%99%E6%9E%9C%E8%BD%AC%E5%BD%95%E7%BB%84%E6%B5%8B%E5%BA%8F\%E5%AE%8C%E6%95%B4%E7%89%88%E6%95%B0%E6%8D%AE\GDR3855-Hylocereus_undulatus_Britt-12-RNAseq_result\4_Function\2_Group_Diff_Function\UP_DOWN\GO\NL-VS-L1.P.html#gene973) | RNA biosynthetic process | 46 (3.73%) | 157 (4.67%) | 0.981762 | 0.999992 |
| 974 | [GO:0044270](file:///E:\2018-7-3%E7%81%AB%E9%BE%99%E6%9E%9C%E8%BD%AC%E5%BD%95%E7%BB%84%E6%B5%8B%E5%BA%8F\%E5%AE%8C%E6%95%B4%E7%89%88%E6%95%B0%E6%8D%AE\GDR3855-Hylocereus_undulatus_Britt-12-RNAseq_result\4_Function\2_Group_Diff_Function\UP_DOWN\GO\NL-VS-L1.P.html#gene974) | cellular nitrogen compound catabolic process | 45 (3.65%) | 154 (4.58%) | 0.981850 | 0.999992 |
| 975 | [GO:0046700](file:///E:\2018-7-3%E7%81%AB%E9%BE%99%E6%9E%9C%E8%BD%AC%E5%BD%95%E7%BB%84%E6%B5%8B%E5%BA%8F\%E5%AE%8C%E6%95%B4%E7%89%88%E6%95%B0%E6%8D%AE\GDR3855-Hylocereus_undulatus_Britt-12-RNAseq_result\4_Function\2_Group_Diff_Function\UP_DOWN\GO\NL-VS-L1.P.html#gene975) | heterocycle catabolic process | 45 (3.65%) | 154 (4.58%) | 0.981850 | 0.999992 |
| 976 | [GO:1901361](file:///E:\2018-7-3%E7%81%AB%E9%BE%99%E6%9E%9C%E8%BD%AC%E5%BD%95%E7%BB%84%E6%B5%8B%E5%BA%8F\%E5%AE%8C%E6%95%B4%E7%89%88%E6%95%B0%E6%8D%AE\GDR3855-Hylocereus_undulatus_Britt-12-RNAseq_result\4_Function\2_Group_Diff_Function\UP_DOWN\GO\NL-VS-L1.P.html#gene976) | organic cyclic compound catabolic process | 45 (3.65%) | 154 (4.58%) | 0.981850 | 0.999992 |
| 977 | [GO:0009058](file:///E:\2018-7-3%E7%81%AB%E9%BE%99%E6%9E%9C%E8%BD%AC%E5%BD%95%E7%BB%84%E6%B5%8B%E5%BA%8F\%E5%AE%8C%E6%95%B4%E7%89%88%E6%95%B0%E6%8D%AE\GDR3855-Hylocereus_undulatus_Britt-12-RNAseq_result\4_Function\2_Group_Diff_Function\UP_DOWN\GO\NL-VS-L1.P.html#gene977) | biosynthetic process | 154 (12.48%) | 473 (14.08%) | 0.982027 | 0.999992 |
| 978 | [GO:0009987](file:///E:\2018-7-3%E7%81%AB%E9%BE%99%E6%9E%9C%E8%BD%AC%E5%BD%95%E7%BB%84%E6%B5%8B%E5%BA%8F\%E5%AE%8C%E6%95%B4%E7%89%88%E6%95%B0%E6%8D%AE\GDR3855-Hylocereus_undulatus_Britt-12-RNAseq_result\4_Function\2_Group_Diff_Function\UP_DOWN\GO\NL-VS-L1.P.html#gene978) | cellular process | 767 (62.16%) | 2163 (64.39%) | 0.982100 | 0.999992 |
| 979 | [GO:0034645](file:///E:\2018-7-3%E7%81%AB%E9%BE%99%E6%9E%9C%E8%BD%AC%E5%BD%95%E7%BB%84%E6%B5%8B%E5%BA%8F\%E5%AE%8C%E6%95%B4%E7%89%88%E6%95%B0%E6%8D%AE\GDR3855-Hylocereus_undulatus_Britt-12-RNAseq_result\4_Function\2_Group_Diff_Function\UP_DOWN\GO\NL-VS-L1.P.html#gene979) | cellular macromolecule biosynthetic process | 80 (6.48%) | 259 (7.71%) | 0.982974 | 0.999992 |
| 980 | [GO:0090305](file:///E:\2018-7-3%E7%81%AB%E9%BE%99%E6%9E%9C%E8%BD%AC%E5%BD%95%E7%BB%84%E6%B5%8B%E5%BA%8F\%E5%AE%8C%E6%95%B4%E7%89%88%E6%95%B0%E6%8D%AE\GDR3855-Hylocereus_undulatus_Britt-12-RNAseq_result\4_Function\2_Group_Diff_Function\UP_DOWN\GO\NL-VS-L1.P.html#gene980) | nucleic acid phosphodiester bond hydrolysis | 3 (0.24%) | 18 (0.54%) | 0.983608 | 0.999992 |
| 981 | [GO:0006366](file:///E:\2018-7-3%E7%81%AB%E9%BE%99%E6%9E%9C%E8%BD%AC%E5%BD%95%E7%BB%84%E6%B5%8B%E5%BA%8F\%E5%AE%8C%E6%95%B4%E7%89%88%E6%95%B0%E6%8D%AE\GDR3855-Hylocereus_undulatus_Britt-12-RNAseq_result\4_Function\2_Group_Diff_Function\UP_DOWN\GO\NL-VS-L1.P.html#gene981) | transcription from RNA polymerase II promoter | 1 (0.08%) | 9 (0.27%) | 0.983870 | 0.999992 |
| 982 | [GO:0072524](file:///E:\2018-7-3%E7%81%AB%E9%BE%99%E6%9E%9C%E8%BD%AC%E5%BD%95%E7%BB%84%E6%B5%8B%E5%BA%8F\%E5%AE%8C%E6%95%B4%E7%89%88%E6%95%B0%E6%8D%AE\GDR3855-Hylocereus_undulatus_Britt-12-RNAseq_result\4_Function\2_Group_Diff_Function\UP_DOWN\GO\NL-VS-L1.P.html#gene982) | pyridine-containing compound metabolic process | 1 (0.08%) | 9 (0.27%) | 0.983870 | 0.999992 |
| 983 | [GO:0009059](file:///E:\2018-7-3%E7%81%AB%E9%BE%99%E6%9E%9C%E8%BD%AC%E5%BD%95%E7%BB%84%E6%B5%8B%E5%BA%8F\%E5%AE%8C%E6%95%B4%E7%89%88%E6%95%B0%E6%8D%AE\GDR3855-Hylocereus_undulatus_Britt-12-RNAseq_result\4_Function\2_Group_Diff_Function\UP_DOWN\GO\NL-VS-L1.P.html#gene983) | macromolecule biosynthetic process | 82 (6.65%) | 266 (7.92%) | 0.985021 | 0.999992 |
| 984 | [GO:0034613](file:///E:\2018-7-3%E7%81%AB%E9%BE%99%E6%9E%9C%E8%BD%AC%E5%BD%95%E7%BB%84%E6%B5%8B%E5%BA%8F\%E5%AE%8C%E6%95%B4%E7%89%88%E6%95%B0%E6%8D%AE\GDR3855-Hylocereus_undulatus_Britt-12-RNAseq_result\4_Function\2_Group_Diff_Function\UP_DOWN\GO\NL-VS-L1.P.html#gene984) | cellular protein localization | 12 (0.97%) | 51 (1.52%) | 0.985374 | 0.999992 |
| 985 | [GO:0070727](file:///E:\2018-7-3%E7%81%AB%E9%BE%99%E6%9E%9C%E8%BD%AC%E5%BD%95%E7%BB%84%E6%B5%8B%E5%BA%8F\%E5%AE%8C%E6%95%B4%E7%89%88%E6%95%B0%E6%8D%AE\GDR3855-Hylocereus_undulatus_Britt-12-RNAseq_result\4_Function\2_Group_Diff_Function\UP_DOWN\GO\NL-VS-L1.P.html#gene985) | cellular macromolecule localization | 12 (0.97%) | 51 (1.52%) | 0.985374 | 0.999992 |
| 986 | [GO:0043170](file:///E:\2018-7-3%E7%81%AB%E9%BE%99%E6%9E%9C%E8%BD%AC%E5%BD%95%E7%BB%84%E6%B5%8B%E5%BA%8F\%E5%AE%8C%E6%95%B4%E7%89%88%E6%95%B0%E6%8D%AE\GDR3855-Hylocereus_undulatus_Britt-12-RNAseq_result\4_Function\2_Group_Diff_Function\UP_DOWN\GO\NL-VS-L1.P.html#gene986) | macromolecule metabolic process | 397 (32.17%) | 1160 (34.53%) | 0.987337 | 0.999992 |
| 987 | [GO:0008152](file:///E:\2018-7-3%E7%81%AB%E9%BE%99%E6%9E%9C%E8%BD%AC%E5%BD%95%E7%BB%84%E6%B5%8B%E5%BA%8F\%E5%AE%8C%E6%95%B4%E7%89%88%E6%95%B0%E6%8D%AE\GDR3855-Hylocereus_undulatus_Britt-12-RNAseq_result\4_Function\2_Group_Diff_Function\UP_DOWN\GO\NL-VS-L1.P.html#gene987) | metabolic process | 929 (75.28%) | 2599 (77.37%) | 0.987515 | 0.999992 |
| 988 | [GO:1901566](file:///E:\2018-7-3%E7%81%AB%E9%BE%99%E6%9E%9C%E8%BD%AC%E5%BD%95%E7%BB%84%E6%B5%8B%E5%BA%8F\%E5%AE%8C%E6%95%B4%E7%89%88%E6%95%B0%E6%8D%AE\GDR3855-Hylocereus_undulatus_Britt-12-RNAseq_result\4_Function\2_Group_Diff_Function\UP_DOWN\GO\NL-VS-L1.P.html#gene988) | organonitrogen compound biosynthetic process | 58 (4.7%) | 196 (5.84%) | 0.987538 | 0.999992 |
| 989 | [GO:0055086](file:///E:\2018-7-3%E7%81%AB%E9%BE%99%E6%9E%9C%E8%BD%AC%E5%BD%95%E7%BB%84%E6%B5%8B%E5%BA%8F\%E5%AE%8C%E6%95%B4%E7%89%88%E6%95%B0%E6%8D%AE\GDR3855-Hylocereus_undulatus_Britt-12-RNAseq_result\4_Function\2_Group_Diff_Function\UP_DOWN\GO\NL-VS-L1.P.html#gene989) | nucleobase-containing small molecule metabolic process | 68 (5.51%) | 226 (6.73%) | 0.987569 | 0.999992 |
| 990 | [GO:0051641](file:///E:\2018-7-3%E7%81%AB%E9%BE%99%E6%9E%9C%E8%BD%AC%E5%BD%95%E7%BB%84%E6%B5%8B%E5%BA%8F\%E5%AE%8C%E6%95%B4%E7%89%88%E6%95%B0%E6%8D%AE\GDR3855-Hylocereus_undulatus_Britt-12-RNAseq_result\4_Function\2_Group_Diff_Function\UP_DOWN\GO\NL-VS-L1.P.html#gene990) | cellular localization | 19 (1.54%) | 75 (2.23%) | 0.987629 | 0.999992 |
| 991 | [GO:0006732](file:///E:\2018-7-3%E7%81%AB%E9%BE%99%E6%9E%9C%E8%BD%AC%E5%BD%95%E7%BB%84%E6%B5%8B%E5%BA%8F\%E5%AE%8C%E6%95%B4%E7%89%88%E6%95%B0%E6%8D%AE\GDR3855-Hylocereus_undulatus_Britt-12-RNAseq_result\4_Function\2_Group_Diff_Function\UP_DOWN\GO\NL-VS-L1.P.html#gene991) | coenzyme metabolic process | 10 (0.81%) | 45 (1.34%) | 0.988212 | 0.999992 |
| 992 | [GO:0044265](file:///E:\2018-7-3%E7%81%AB%E9%BE%99%E6%9E%9C%E8%BD%AC%E5%BD%95%E7%BB%84%E6%B5%8B%E5%BA%8F\%E5%AE%8C%E6%95%B4%E7%89%88%E6%95%B0%E6%8D%AE\GDR3855-Hylocereus_undulatus_Britt-12-RNAseq_result\4_Function\2_Group_Diff_Function\UP_DOWN\GO\NL-VS-L1.P.html#gene992) | cellular macromolecule catabolic process | 10 (0.81%) | 45 (1.34%) | 0.988212 | 0.999992 |
| 993 | [GO:0006281](file:///E:\2018-7-3%E7%81%AB%E9%BE%99%E6%9E%9C%E8%BD%AC%E5%BD%95%E7%BB%84%E6%B5%8B%E5%BA%8F\%E5%AE%8C%E6%95%B4%E7%89%88%E6%95%B0%E6%8D%AE\GDR3855-Hylocereus_undulatus_Britt-12-RNAseq_result\4_Function\2_Group_Diff_Function\UP_DOWN\GO\NL-VS-L1.P.html#gene993) | DNA repair | 4 (0.32%) | 23 (0.68%) | 0.988302 | 0.999992 |
| 994 | [GO:0051603](file:///E:\2018-7-3%E7%81%AB%E9%BE%99%E6%9E%9C%E8%BD%AC%E5%BD%95%E7%BB%84%E6%B5%8B%E5%BA%8F\%E5%AE%8C%E6%95%B4%E7%89%88%E6%95%B0%E6%8D%AE\GDR3855-Hylocereus_undulatus_Britt-12-RNAseq_result\4_Function\2_Group_Diff_Function\UP_DOWN\GO\NL-VS-L1.P.html#gene994) | proteolysis involved in cellular protein catabolic process | 8 (0.65%) | 38 (1.13%) | 0.988420 | 0.999992 |
| 995 | [GO:0006396](file:///E:\2018-7-3%E7%81%AB%E9%BE%99%E6%9E%9C%E8%BD%AC%E5%BD%95%E7%BB%84%E6%B5%8B%E5%BA%8F\%E5%AE%8C%E6%95%B4%E7%89%88%E6%95%B0%E6%8D%AE\GDR3855-Hylocereus_undulatus_Britt-12-RNAseq_result\4_Function\2_Group_Diff_Function\UP_DOWN\GO\NL-VS-L1.P.html#gene995) | RNA processing | 12 (0.97%) | 52 (1.55%) | 0.988490 | 0.999992 |
| 996 | [GO:0044249](file:///E:\2018-7-3%E7%81%AB%E9%BE%99%E6%9E%9C%E8%BD%AC%E5%BD%95%E7%BB%84%E6%B5%8B%E5%BA%8F\%E5%AE%8C%E6%95%B4%E7%89%88%E6%95%B0%E6%8D%AE\GDR3855-Hylocereus_undulatus_Britt-12-RNAseq_result\4_Function\2_Group_Diff_Function\UP_DOWN\GO\NL-VS-L1.P.html#gene996) | cellular biosynthetic process | 137 (11.1%) | 429 (12.77%) | 0.988673 | 0.999992 |
| 997 | [GO:1901576](file:///E:\2018-7-3%E7%81%AB%E9%BE%99%E6%9E%9C%E8%BD%AC%E5%BD%95%E7%BB%84%E6%B5%8B%E5%BA%8F\%E5%AE%8C%E6%95%B4%E7%89%88%E6%95%B0%E6%8D%AE\GDR3855-Hylocereus_undulatus_Britt-12-RNAseq_result\4_Function\2_Group_Diff_Function\UP_DOWN\GO\NL-VS-L1.P.html#gene997) | organic substance biosynthetic process | 139 (11.26%) | 435 (12.95%) | 0.988920 | 0.999992 |
| 998 | [GO:0006625](file:///E:\2018-7-3%E7%81%AB%E9%BE%99%E6%9E%9C%E8%BD%AC%E5%BD%95%E7%BB%84%E6%B5%8B%E5%BA%8F\%E5%AE%8C%E6%95%B4%E7%89%88%E6%95%B0%E6%8D%AE\GDR3855-Hylocereus_undulatus_Britt-12-RNAseq_result\4_Function\2_Group_Diff_Function\UP_DOWN\GO\NL-VS-L1.P.html#gene998) | protein targeting to peroxisome | 1 (0.08%) | 10 (0.3%) | 0.989812 | 0.999992 |
| 999 | [GO:0022904](file:///E:\2018-7-3%E7%81%AB%E9%BE%99%E6%9E%9C%E8%BD%AC%E5%BD%95%E7%BB%84%E6%B5%8B%E5%BA%8F\%E5%AE%8C%E6%95%B4%E7%89%88%E6%95%B0%E6%8D%AE\GDR3855-Hylocereus_undulatus_Britt-12-RNAseq_result\4_Function\2_Group_Diff_Function\UP_DOWN\GO\NL-VS-L1.P.html#gene999) | respiratory electron transport chain | 1 (0.08%) | 10 (0.3%) | 0.989812 | 0.999992 |
| 1000 | [GO:0043574](file:///E:\2018-7-3%E7%81%AB%E9%BE%99%E6%9E%9C%E8%BD%AC%E5%BD%95%E7%BB%84%E6%B5%8B%E5%BA%8F\%E5%AE%8C%E6%95%B4%E7%89%88%E6%95%B0%E6%8D%AE\GDR3855-Hylocereus_undulatus_Britt-12-RNAseq_result\4_Function\2_Group_Diff_Function\UP_DOWN\GO\NL-VS-L1.P.html#gene1000) | peroxisomal transport | 1 (0.08%) | 10 (0.3%) | 0.989812 | 0.999992 |
| 1001 | [GO:0044703](file:///E:\2018-7-3%E7%81%AB%E9%BE%99%E6%9E%9C%E8%BD%AC%E5%BD%95%E7%BB%84%E6%B5%8B%E5%BA%8F\%E5%AE%8C%E6%95%B4%E7%89%88%E6%95%B0%E6%8D%AE\GDR3855-Hylocereus_undulatus_Britt-12-RNAseq_result\4_Function\2_Group_Diff_Function\UP_DOWN\GO\NL-VS-L1.P.html#gene1001) | multi-organism reproductive process | 1 (0.08%) | 10 (0.3%) | 0.989812 | 0.999992 |
| 1002 | [GO:0072662](file:///E:\2018-7-3%E7%81%AB%E9%BE%99%E6%9E%9C%E8%BD%AC%E5%BD%95%E7%BB%84%E6%B5%8B%E5%BA%8F\%E5%AE%8C%E6%95%B4%E7%89%88%E6%95%B0%E6%8D%AE\GDR3855-Hylocereus_undulatus_Britt-12-RNAseq_result\4_Function\2_Group_Diff_Function\UP_DOWN\GO\NL-VS-L1.P.html#gene1002) | protein localization to peroxisome | 1 (0.08%) | 10 (0.3%) | 0.989812 | 0.999992 |
| 1003 | [GO:0072663](file:///E:\2018-7-3%E7%81%AB%E9%BE%99%E6%9E%9C%E8%BD%AC%E5%BD%95%E7%BB%84%E6%B5%8B%E5%BA%8F\%E5%AE%8C%E6%95%B4%E7%89%88%E6%95%B0%E6%8D%AE\GDR3855-Hylocereus_undulatus_Britt-12-RNAseq_result\4_Function\2_Group_Diff_Function\UP_DOWN\GO\NL-VS-L1.P.html#gene1003) | establishment of protein localization to peroxisome | 1 (0.08%) | 10 (0.3%) | 0.989812 | 0.999992 |
| 1004 | [GO:0009108](file:///E:\2018-7-3%E7%81%AB%E9%BE%99%E6%9E%9C%E8%BD%AC%E5%BD%95%E7%BB%84%E6%B5%8B%E5%BA%8F\%E5%AE%8C%E6%95%B4%E7%89%88%E6%95%B0%E6%8D%AE\GDR3855-Hylocereus_undulatus_Britt-12-RNAseq_result\4_Function\2_Group_Diff_Function\UP_DOWN\GO\NL-VS-L1.P.html#gene1004) | coenzyme biosynthetic process | 2 (0.16%) | 15 (0.45%) | 0.990020 | 0.999992 |
| 1005 | [GO:0009056](file:///E:\2018-7-3%E7%81%AB%E9%BE%99%E6%9E%9C%E8%BD%AC%E5%BD%95%E7%BB%84%E6%B5%8B%E5%BA%8F\%E5%AE%8C%E6%95%B4%E7%89%88%E6%95%B0%E6%8D%AE\GDR3855-Hylocereus_undulatus_Britt-12-RNAseq_result\4_Function\2_Group_Diff_Function\UP_DOWN\GO\NL-VS-L1.P.html#gene1005) | catabolic process | 69 (5.59%) | 231 (6.88%) | 0.990412 | 0.999992 |
| 1006 | [GO:1902582](file:///E:\2018-7-3%E7%81%AB%E9%BE%99%E6%9E%9C%E8%BD%AC%E5%BD%95%E7%BB%84%E6%B5%8B%E5%BA%8F\%E5%AE%8C%E6%95%B4%E7%89%88%E6%95%B0%E6%8D%AE\GDR3855-Hylocereus_undulatus_Britt-12-RNAseq_result\4_Function\2_Group_Diff_Function\UP_DOWN\GO\NL-VS-L1.P.html#gene1006) | single-organism intracellular transport | 12 (0.97%) | 53 (1.58%) | 0.990983 | 0.999992 |
| 1007 | [GO:1901575](file:///E:\2018-7-3%E7%81%AB%E9%BE%99%E6%9E%9C%E8%BD%AC%E5%BD%95%E7%BB%84%E6%B5%8B%E5%BA%8F\%E5%AE%8C%E6%95%B4%E7%89%88%E6%95%B0%E6%8D%AE\GDR3855-Hylocereus_undulatus_Britt-12-RNAseq_result\4_Function\2_Group_Diff_Function\UP_DOWN\GO\NL-VS-L1.P.html#gene1007) | organic substance catabolic process | 66 (5.35%) | 223 (6.64%) | 0.991605 | 0.999992 |
| 1008 | [GO:0006974](file:///E:\2018-7-3%E7%81%AB%E9%BE%99%E6%9E%9C%E8%BD%AC%E5%BD%95%E7%BB%84%E6%B5%8B%E5%BA%8F\%E5%AE%8C%E6%95%B4%E7%89%88%E6%95%B0%E6%8D%AE\GDR3855-Hylocereus_undulatus_Britt-12-RNAseq_result\4_Function\2_Group_Diff_Function\UP_DOWN\GO\NL-VS-L1.P.html#gene1008) | cellular response to DNA damage stimulus | 4 (0.32%) | 24 (0.71%) | 0.991662 | 0.999992 |
| 1009 | [GO:0009123](file:///E:\2018-7-3%E7%81%AB%E9%BE%99%E6%9E%9C%E8%BD%AC%E5%BD%95%E7%BB%84%E6%B5%8B%E5%BA%8F\%E5%AE%8C%E6%95%B4%E7%89%88%E6%95%B0%E6%8D%AE\GDR3855-Hylocereus_undulatus_Britt-12-RNAseq_result\4_Function\2_Group_Diff_Function\UP_DOWN\GO\NL-VS-L1.P.html#gene1009) | nucleoside monophosphate metabolic process | 6 (0.49%) | 32 (0.95%) | 0.992197 | 0.999992 |
| 1010 | [GO:0009124](file:///E:\2018-7-3%E7%81%AB%E9%BE%99%E6%9E%9C%E8%BD%AC%E5%BD%95%E7%BB%84%E6%B5%8B%E5%BA%8F\%E5%AE%8C%E6%95%B4%E7%89%88%E6%95%B0%E6%8D%AE\GDR3855-Hylocereus_undulatus_Britt-12-RNAseq_result\4_Function\2_Group_Diff_Function\UP_DOWN\GO\NL-VS-L1.P.html#gene1010) | nucleoside monophosphate biosynthetic process | 6 (0.49%) | 32 (0.95%) | 0.992197 | 0.999992 |
| 1011 | [GO:0009156](file:///E:\2018-7-3%E7%81%AB%E9%BE%99%E6%9E%9C%E8%BD%AC%E5%BD%95%E7%BB%84%E6%B5%8B%E5%BA%8F\%E5%AE%8C%E6%95%B4%E7%89%88%E6%95%B0%E6%8D%AE\GDR3855-Hylocereus_undulatus_Britt-12-RNAseq_result\4_Function\2_Group_Diff_Function\UP_DOWN\GO\NL-VS-L1.P.html#gene1011) | ribonucleoside monophosphate biosynthetic process | 6 (0.49%) | 32 (0.95%) | 0.992197 | 0.999992 |
| 1012 | [GO:0009161](file:///E:\2018-7-3%E7%81%AB%E9%BE%99%E6%9E%9C%E8%BD%AC%E5%BD%95%E7%BB%84%E6%B5%8B%E5%BA%8F\%E5%AE%8C%E6%95%B4%E7%89%88%E6%95%B0%E6%8D%AE\GDR3855-Hylocereus_undulatus_Britt-12-RNAseq_result\4_Function\2_Group_Diff_Function\UP_DOWN\GO\NL-VS-L1.P.html#gene1012) | ribonucleoside monophosphate metabolic process | 6 (0.49%) | 32 (0.95%) | 0.992197 | 0.999992 |
| 1013 | [GO:0044238](file:///E:\2018-7-3%E7%81%AB%E9%BE%99%E6%9E%9C%E8%BD%AC%E5%BD%95%E7%BB%84%E6%B5%8B%E5%BA%8F\%E5%AE%8C%E6%95%B4%E7%89%88%E6%95%B0%E6%8D%AE\GDR3855-Hylocereus_undulatus_Britt-12-RNAseq_result\4_Function\2_Group_Diff_Function\UP_DOWN\GO\NL-VS-L1.P.html#gene1013) | primary metabolic process | 526 (42.63%) | 1522 (45.31%) | 0.992249 | 0.999992 |
| 1014 | [GO:0006733](file:///E:\2018-7-3%E7%81%AB%E9%BE%99%E6%9E%9C%E8%BD%AC%E5%BD%95%E7%BB%84%E6%B5%8B%E5%BA%8F\%E5%AE%8C%E6%95%B4%E7%89%88%E6%95%B0%E6%8D%AE\GDR3855-Hylocereus_undulatus_Britt-12-RNAseq_result\4_Function\2_Group_Diff_Function\UP_DOWN\GO\NL-VS-L1.P.html#gene1014) | oxidoreduction coenzyme metabolic process | 1 (0.08%) | 11 (0.33%) | 0.993566 | 0.999992 |
| 1015 | [GO:0051649](file:///E:\2018-7-3%E7%81%AB%E9%BE%99%E6%9E%9C%E8%BD%AC%E5%BD%95%E7%BB%84%E6%B5%8B%E5%BA%8F\%E5%AE%8C%E6%95%B4%E7%89%88%E6%95%B0%E6%8D%AE\GDR3855-Hylocereus_undulatus_Britt-12-RNAseq_result\4_Function\2_Group_Diff_Function\UP_DOWN\GO\NL-VS-L1.P.html#gene1015) | establishment of localization in cell | 16 (1.3%) | 69 (2.05%) | 0.994822 | 0.999992 |
| 1016 | [GO:0051186](file:///E:\2018-7-3%E7%81%AB%E9%BE%99%E6%9E%9C%E8%BD%AC%E5%BD%95%E7%BB%84%E6%B5%8B%E5%BA%8F\%E5%AE%8C%E6%95%B4%E7%89%88%E6%95%B0%E6%8D%AE\GDR3855-Hylocereus_undulatus_Britt-12-RNAseq_result\4_Function\2_Group_Diff_Function\UP_DOWN\GO\NL-VS-L1.P.html#gene1016) | cofactor metabolic process | 15 (1.22%) | 66 (1.96%) | 0.995301 | 0.999992 |
| 1017 | [GO:0006605](file:///E:\2018-7-3%E7%81%AB%E9%BE%99%E6%9E%9C%E8%BD%AC%E5%BD%95%E7%BB%84%E6%B5%8B%E5%BA%8F\%E5%AE%8C%E6%95%B4%E7%89%88%E6%95%B0%E6%8D%AE\GDR3855-Hylocereus_undulatus_Britt-12-RNAseq_result\4_Function\2_Group_Diff_Function\UP_DOWN\GO\NL-VS-L1.P.html#gene1017) | protein targeting | 9 (0.73%) | 45 (1.34%) | 0.995477 | 0.999992 |
| 1018 | [GO:1902580](file:///E:\2018-7-3%E7%81%AB%E9%BE%99%E6%9E%9C%E8%BD%AC%E5%BD%95%E7%BB%84%E6%B5%8B%E5%BA%8F\%E5%AE%8C%E6%95%B4%E7%89%88%E6%95%B0%E6%8D%AE\GDR3855-Hylocereus_undulatus_Britt-12-RNAseq_result\4_Function\2_Group_Diff_Function\UP_DOWN\GO\NL-VS-L1.P.html#gene1018) | single-organism cellular localization | 2 (0.16%) | 17 (0.51%) | 0.995548 | 0.999992 |
| 1019 | [GO:1901362](file:///E:\2018-7-3%E7%81%AB%E9%BE%99%E6%9E%9C%E8%BD%AC%E5%BD%95%E7%BB%84%E6%B5%8B%E5%BA%8F\%E5%AE%8C%E6%95%B4%E7%89%88%E6%95%B0%E6%8D%AE\GDR3855-Hylocereus_undulatus_Britt-12-RNAseq_result\4_Function\2_Group_Diff_Function\UP_DOWN\GO\NL-VS-L1.P.html#gene1019) | organic cyclic compound biosynthetic process | 77 (6.24%) | 261 (7.77%) | 0.995677 | 0.999992 |
| 1020 | [GO:0051171](file:///E:\2018-7-3%E7%81%AB%E9%BE%99%E6%9E%9C%E8%BD%AC%E5%BD%95%E7%BB%84%E6%B5%8B%E5%BA%8F\%E5%AE%8C%E6%95%B4%E7%89%88%E6%95%B0%E6%8D%AE\GDR3855-Hylocereus_undulatus_Britt-12-RNAseq_result\4_Function\2_Group_Diff_Function\UP_DOWN\GO\NL-VS-L1.P.html#gene1020) | regulation of nitrogen compound metabolic process | 1 (0.08%) | 12 (0.36%) | 0.995937 | 0.999992 |
| 1021 | [GO:0034654](file:///E:\2018-7-3%E7%81%AB%E9%BE%99%E6%9E%9C%E8%BD%AC%E5%BD%95%E7%BB%84%E6%B5%8B%E5%BA%8F\%E5%AE%8C%E6%95%B4%E7%89%88%E6%95%B0%E6%8D%AE\GDR3855-Hylocereus_undulatus_Britt-12-RNAseq_result\4_Function\2_Group_Diff_Function\UP_DOWN\GO\NL-VS-L1.P.html#gene1021) | nucleobase-containing compound biosynthetic process | 61 (4.94%) | 213 (6.34%) | 0.995940 | 0.999992 |
| 1022 | [GO:0044260](file:///E:\2018-7-3%E7%81%AB%E9%BE%99%E6%9E%9C%E8%BD%AC%E5%BD%95%E7%BB%84%E6%B5%8B%E5%BA%8F\%E5%AE%8C%E6%95%B4%E7%89%88%E6%95%B0%E6%8D%AE\GDR3855-Hylocereus_undulatus_Britt-12-RNAseq_result\4_Function\2_Group_Diff_Function\UP_DOWN\GO\NL-VS-L1.P.html#gene1022) | cellular macromolecule metabolic process | 294 (23.82%) | 888 (26.44%) | 0.996177 | 0.999992 |
| 1023 | [GO:0071704](file:///E:\2018-7-3%E7%81%AB%E9%BE%99%E6%9E%9C%E8%BD%AC%E5%BD%95%E7%BB%84%E6%B5%8B%E5%BA%8F\%E5%AE%8C%E6%95%B4%E7%89%88%E6%95%B0%E6%8D%AE\GDR3855-Hylocereus_undulatus_Britt-12-RNAseq_result\4_Function\2_Group_Diff_Function\UP_DOWN\GO\NL-VS-L1.P.html#gene1023) | organic substance metabolic process | 587 (47.57%) | 1698 (50.55%) | 0.996210 | 0.999992 |
| 1024 | [GO:0046907](file:///E:\2018-7-3%E7%81%AB%E9%BE%99%E6%9E%9C%E8%BD%AC%E5%BD%95%E7%BB%84%E6%B5%8B%E5%BA%8F\%E5%AE%8C%E6%95%B4%E7%89%88%E6%95%B0%E6%8D%AE\GDR3855-Hylocereus_undulatus_Britt-12-RNAseq_result\4_Function\2_Group_Diff_Function\UP_DOWN\GO\NL-VS-L1.P.html#gene1024) | intracellular transport | 15 (1.22%) | 67 (1.99%) | 0.996307 | 0.999992 |
| 1025 | [GO:0006886](file:///E:\2018-7-3%E7%81%AB%E9%BE%99%E6%9E%9C%E8%BD%AC%E5%BD%95%E7%BB%84%E6%B5%8B%E5%BA%8F\%E5%AE%8C%E6%95%B4%E7%89%88%E6%95%B0%E6%8D%AE\GDR3855-Hylocereus_undulatus_Britt-12-RNAseq_result\4_Function\2_Group_Diff_Function\UP_DOWN\GO\NL-VS-L1.P.html#gene1025) | intracellular protein transport | 9 (0.73%) | 46 (1.37%) | 0.996591 | 0.999992 |
| 1026 | [GO:0019438](file:///E:\2018-7-3%E7%81%AB%E9%BE%99%E6%9E%9C%E8%BD%AC%E5%BD%95%E7%BB%84%E6%B5%8B%E5%BA%8F\%E5%AE%8C%E6%95%B4%E7%89%88%E6%95%B0%E6%8D%AE\GDR3855-Hylocereus_undulatus_Britt-12-RNAseq_result\4_Function\2_Group_Diff_Function\UP_DOWN\GO\NL-VS-L1.P.html#gene1026) | aromatic compound biosynthetic process | 72 (5.83%) | 248 (7.38%) | 0.996760 | 0.999992 |
| 1027 | [GO:0016070](file:///E:\2018-7-3%E7%81%AB%E9%BE%99%E6%9E%9C%E8%BD%AC%E5%BD%95%E7%BB%84%E6%B5%8B%E5%BA%8F\%E5%AE%8C%E6%95%B4%E7%89%88%E6%95%B0%E6%8D%AE\GDR3855-Hylocereus_undulatus_Britt-12-RNAseq_result\4_Function\2_Group_Diff_Function\UP_DOWN\GO\NL-VS-L1.P.html#gene1027) | RNA metabolic process | 76 (6.16%) | 262 (7.8%) | 0.997521 | 0.999992 |
| 1028 | [GO:0018130](file:///E:\2018-7-3%E7%81%AB%E9%BE%99%E6%9E%9C%E8%BD%AC%E5%BD%95%E7%BB%84%E6%B5%8B%E5%BA%8F\%E5%AE%8C%E6%95%B4%E7%89%88%E6%95%B0%E6%8D%AE\GDR3855-Hylocereus_undulatus_Britt-12-RNAseq_result\4_Function\2_Group_Diff_Function\UP_DOWN\GO\NL-VS-L1.P.html#gene1028) | heterocycle biosynthetic process | 68 (5.51%) | 239 (7.12%) | 0.997959 | 0.999992 |
| 1029 | [GO:0006576](file:///E:\2018-7-3%E7%81%AB%E9%BE%99%E6%9E%9C%E8%BD%AC%E5%BD%95%E7%BB%84%E6%B5%8B%E5%BA%8F\%E5%AE%8C%E6%95%B4%E7%89%88%E6%95%B0%E6%8D%AE\GDR3855-Hylocereus_undulatus_Britt-12-RNAseq_result\4_Function\2_Group_Diff_Function\UP_DOWN\GO\NL-VS-L1.P.html#gene1029) | cellular biogenic amine metabolic process | 1 (0.08%) | 14 (0.42%) | 0.998381 | 0.999992 |
| 1030 | [GO:0044106](file:///E:\2018-7-3%E7%81%AB%E9%BE%99%E6%9E%9C%E8%BD%AC%E5%BD%95%E7%BB%84%E6%B5%8B%E5%BA%8F\%E5%AE%8C%E6%95%B4%E7%89%88%E6%95%B0%E6%8D%AE\GDR3855-Hylocereus_undulatus_Britt-12-RNAseq_result\4_Function\2_Group_Diff_Function\UP_DOWN\GO\NL-VS-L1.P.html#gene1030) | cellular amine metabolic process | 1 (0.08%) | 14 (0.42%) | 0.998381 | 0.999992 |
| 1031 | [GO:1901564](file:///E:\2018-7-3%E7%81%AB%E9%BE%99%E6%9E%9C%E8%BD%AC%E5%BD%95%E7%BB%84%E6%B5%8B%E5%BA%8F\%E5%AE%8C%E6%95%B4%E7%89%88%E6%95%B0%E6%8D%AE\GDR3855-Hylocereus_undulatus_Britt-12-RNAseq_result\4_Function\2_Group_Diff_Function\UP_DOWN\GO\NL-VS-L1.P.html#gene1031) | organonitrogen compound metabolic process | 126 (10.21%) | 417 (12.41%) | 0.998817 | 0.999992 |
| 1032 | [GO:0044248](file:///E:\2018-7-3%E7%81%AB%E9%BE%99%E6%9E%9C%E8%BD%AC%E5%BD%95%E7%BB%84%E6%B5%8B%E5%BA%8F\%E5%AE%8C%E6%95%B4%E7%89%88%E6%95%B0%E6%8D%AE\GDR3855-Hylocereus_undulatus_Britt-12-RNAseq_result\4_Function\2_Group_Diff_Function\UP_DOWN\GO\NL-VS-L1.P.html#gene1032) | cellular catabolic process | 58 (4.7%) | 212 (6.31%) | 0.998884 | 0.999992 |
| 1033 | [GO:0009308](file:///E:\2018-7-3%E7%81%AB%E9%BE%99%E6%9E%9C%E8%BD%AC%E5%BD%95%E7%BB%84%E6%B5%8B%E5%BA%8F\%E5%AE%8C%E6%95%B4%E7%89%88%E6%95%B0%E6%8D%AE\GDR3855-Hylocereus_undulatus_Britt-12-RNAseq_result\4_Function\2_Group_Diff_Function\UP_DOWN\GO\NL-VS-L1.P.html#gene1033) | amine metabolic process | 1 (0.08%) | 15 (0.45%) | 0.998978 | 0.999992 |
| 1034 | [GO:0044271](file:///E:\2018-7-3%E7%81%AB%E9%BE%99%E6%9E%9C%E8%BD%AC%E5%BD%95%E7%BB%84%E6%B5%8B%E5%BA%8F\%E5%AE%8C%E6%95%B4%E7%89%88%E6%95%B0%E6%8D%AE\GDR3855-Hylocereus_undulatus_Britt-12-RNAseq_result\4_Function\2_Group_Diff_Function\UP_DOWN\GO\NL-VS-L1.P.html#gene1034) | cellular nitrogen compound biosynthetic process | 91 (7.37%) | 316 (9.41%) | 0.999275 | 0.999992 |
| 1035 | [GO:0044237](file:///E:\2018-7-3%E7%81%AB%E9%BE%99%E6%9E%9C%E8%BD%AC%E5%BD%95%E7%BB%84%E6%B5%8B%E5%BA%8F\%E5%AE%8C%E6%95%B4%E7%89%88%E6%95%B0%E6%8D%AE\GDR3855-Hylocereus_undulatus_Britt-12-RNAseq_result\4_Function\2_Group_Diff_Function\UP_DOWN\GO\NL-VS-L1.P.html#gene1035) | cellular metabolic process | 574 (46.52%) | 1688 (50.25%) | 0.999578 | 0.999992 |
| 1036 | [GO:0090304](file:///E:\2018-7-3%E7%81%AB%E9%BE%99%E6%9E%9C%E8%BD%AC%E5%BD%95%E7%BB%84%E6%B5%8B%E5%BA%8F\%E5%AE%8C%E6%95%B4%E7%89%88%E6%95%B0%E6%8D%AE\GDR3855-Hylocereus_undulatus_Britt-12-RNAseq_result\4_Function\2_Group_Diff_Function\UP_DOWN\GO\NL-VS-L1.P.html#gene1036) | nucleic acid metabolic process | 101 (8.18%) | 350 (10.42%) | 0.999579 | 0.999992 |
| 1037 | [GO:1901360](file:///E:\2018-7-3%E7%81%AB%E9%BE%99%E6%9E%9C%E8%BD%AC%E5%BD%95%E7%BB%84%E6%B5%8B%E5%BA%8F\%E5%AE%8C%E6%95%B4%E7%89%88%E6%95%B0%E6%8D%AE\GDR3855-Hylocereus_undulatus_Britt-12-RNAseq_result\4_Function\2_Group_Diff_Function\UP_DOWN\GO\NL-VS-L1.P.html#gene1037) | organic cyclic compound metabolic process | 196 (15.88%) | 645 (19.2%) | 0.999929 | 0.999992 |
| 1038 | [GO:0006725](file:///E:\2018-7-3%E7%81%AB%E9%BE%99%E6%9E%9C%E8%BD%AC%E5%BD%95%E7%BB%84%E6%B5%8B%E5%BA%8F\%E5%AE%8C%E6%95%B4%E7%89%88%E6%95%B0%E6%8D%AE\GDR3855-Hylocereus_undulatus_Britt-12-RNAseq_result\4_Function\2_Group_Diff_Function\UP_DOWN\GO\NL-VS-L1.P.html#gene1038) | cellular aromatic compound metabolic process | 190 (15.4%) | 634 (18.87%) | 0.999970 | 0.999992 |
| 1039 | [GO:0006807](file:///E:\2018-7-3%E7%81%AB%E9%BE%99%E6%9E%9C%E8%BD%AC%E5%BD%95%E7%BB%84%E6%B5%8B%E5%BA%8F\%E5%AE%8C%E6%95%B4%E7%89%88%E6%95%B0%E6%8D%AE\GDR3855-Hylocereus_undulatus_Britt-12-RNAseq_result\4_Function\2_Group_Diff_Function\UP_DOWN\GO\NL-VS-L1.P.html#gene1039) | nitrogen compound metabolic process | 229 (18.56%) | 748 (22.27%) | 0.999971 | 0.999992 |
| 1040 | [GO:0006139](file:///E:\2018-7-3%E7%81%AB%E9%BE%99%E6%9E%9C%E8%BD%AC%E5%BD%95%E7%BB%84%E6%B5%8B%E5%BA%8F\%E5%AE%8C%E6%95%B4%E7%89%88%E6%95%B0%E6%8D%AE\GDR3855-Hylocereus_undulatus_Britt-12-RNAseq_result\4_Function\2_Group_Diff_Function\UP_DOWN\GO\NL-VS-L1.P.html#gene1040) | nucleobase-containing compound metabolic process | 167 (13.53%) | 568 (16.91%) | 0.999977 | 0.999992 |
| 1041 | [GO:0034641](file:///E:\2018-7-3%E7%81%AB%E9%BE%99%E6%9E%9C%E8%BD%AC%E5%BD%95%E7%BB%84%E6%B5%8B%E5%BA%8F\%E5%AE%8C%E6%95%B4%E7%89%88%E6%95%B0%E6%8D%AE\GDR3855-Hylocereus_undulatus_Britt-12-RNAseq_result\4_Function\2_Group_Diff_Function\UP_DOWN\GO\NL-VS-L1.P.html#gene1041) | cellular nitrogen compound metabolic process | 199 (16.13%) | 667 (19.86%) | 0.999988 | 0.999992 |
| 1042 | [GO:0046483](file:///E:\2018-7-3%E7%81%AB%E9%BE%99%E6%9E%9C%E8%BD%AC%E5%BD%95%E7%BB%84%E6%B5%8B%E5%BA%8F\%E5%AE%8C%E6%95%B4%E7%89%88%E6%95%B0%E6%8D%AE\GDR3855-Hylocereus_undulatus_Britt-12-RNAseq_result\4_Function\2_Group_Diff_Function\UP_DOWN\GO\NL-VS-L1.P.html#gene1042) | heterocycle metabolic process | 181 (14.67%) | 617 (18.37%) | 0.999992 | 0.999992 |
