# Supplementary material for: Transcriptomic analysis of flower induction for long-day pitaya by supplementary lighting in short-day winter season
Source: BMC Genomics. 2020 Apr 29;21:329. doi: 10.1186/s12864-020-6726-6 (PMC7191803; doi:10.1186/s12864-020-6726-6)
Supplement: Supplementary file 9 — Additional file 9: Supplemental S9. The DEGs related to plant hormone. [file 12864_2020_6726_MOESM9_ESM.docx]

Supplemental S9 The DEGs related to plant hormone

| **Gene ID** | **NL rpkm** | **L0 rpkm** | **up or down** | **function** |
| --- | --- | --- | --- | --- |
| auxin-related genes | | | | |
| Unigene0029163 | 26.615 | 10.638 | down | auxin-responsive protein SAUR32 |
| Brassinolide-related genes | | | | |
| Unigene0026249 | 8.509 | 20.728 | up | BRI1 kinase inhibitor 1 |
| Jasmonic acid-related genes | | | | |
| Unigene0021973 | 65.278 | 16.230 | down | jasmonate-induced protein homolog |
| **Gene ID** | **NL rpkm** | **L1 rpkm** | **up or down** | **function** |
| auxin-related genes | | | | |
| Unigene0000921 | 0.001 | 1.652 | up | auxin-responsive protein SAUR21 |
| Unigene0002811 | 15.619 | 31.367 | up | auxin response factor 7 isoform X1 |
| Unigene0002914 | 0.495 | 6.515 | up | probable indole-3-acetic acid-amido synthetase GH3.1 |
| Unigene0004150 | 0.001 | 2.923 | up | auxin-responsive protein SAUR50 |
| Unigene0006432 | 5.988 | 14.552 | up | IAA-ARF-dimerization |
| Unigene0012516 | 0.354 | 3.874 | up | auxin-responsive protein SAUR50-like |
| Unigene0012821 | 0.689 | 4.756 | up | auxin-responsive protein SAUR72-like |
| Unigene0015734 | 0.001 | 1.905 | up | indole-3-acetic acid-amido synthetase GH3.6-like |
| Unigene0030711 | 0.531 | 10.302 | up | indole-3-acetic acid-amido synthetase GH3.6 |
| Unigene0031013 | 2.658 | 56.500 | up | auxin-responsive protein IAA27-like |
| Unigene0032942 | 1.144 | 12.193 | up | auxin-responsive protein IAA1 |
| Unigene0033388 | 24.108 | 60.359 | up | auxin-responsive protein IAA2 |
| Unigene0036400 | 0.753 | 20.033 | up | probable indole-3-acetic acid-amido synthetase GH3.1 |
| Unigene0036729 | 2.762 | 13.854 | up | auxin transporter-like protein 4 |
| Unigene0037298 | 0.529 | 10.709 | up | auxin-responsive protein IAA14 |
| Unigene0037786 | 0.069 | 10.571 | up | auxin transporter-like protein 2 |
| Unigene0021847 | 0.881 | 3.857 | up | auxin-responsive protein SAUR71 |
| Unigene0024170 | 4.127 | 21.369 | up | protein AUXIN RESPONSE 4 |
| Unigene0027945 | 4.721 | 24.266 | up | auxin-induced protein 22D |
| Unigene0028194 | 0.588 | 2.480 | up | probable indole-3-acetic acid-amido synthetase GH3.6 |
| Unigene0028447 | 7.887 | 51.809 | up | auxin response factor 5 |
| Unigene0027380 | 24.606 | 4.157 | domn | auxin-responsive protein IAA4 |
| Unigene0030569 | 46.678 | 8.147 | domn | auxin-induced protein IAA6 |
| Gibberellic acid-related genes | | | | |
| Unigene0005738 | 0.274 | 5.017 | up | DELLA protein GAI1 |
| Unigene0031663 | 0.262 | 7.077 | up | DELLA protein GAI1 |
| Unigene0046182 | 24.142 | 63.940 | up | DELLA protein GAI |
| Brassinolide-related genes | | | | |
| Unigene0004470 | 4.015 | 15.049 | up | probable serine/threonine-protein kinase At4g35230 |
| Unigene0025032 | 19.775 | 88.820 | up | probable xyloglucan endotransglucosylase/hydrolase protein 23 |
| Unigene0030389 | 12.369 | 27.881 | up | shaggy-related protein kinase eta-like |
| Unigene0038596 | 1.517 | 17.534 | up | cyclin-D3-1 |
| Unigene0035058 | 1.089 | 30.895 | up | cyclin-D3-1 |
| Unigene0042473 | 9.549 | 21.461 | up | systemin receptor SR160 |
| Jasmonic acid-related genes | | | | |
| Unigene0030164 | 12.298 | 36.553 | up | transcription factor MYC2 |
| Unigene0032865 | 44.134 | 107.242 | up | JAZ-like protein |
| Unigene0034754 | 9.656 | 24.657 | up | coronatine-insensitive protein 1 |
| Unigene0049406 | 3.553 | 36.488 | up | protein TIFY 9 |
| **Gene ID** | **L0 rpkm** | **L1 rpkm** | **up or down** | **function** |
| auxin-related genes | | | | |
| Unigene0037786 | 0.001 | 10.571 | up | auxin transporter-like protein 2 |
| Unigene0036729 | 2.085 | 13.854 | up | auxin transporter-like protein 4 |
| Unigene0038741 | 1.098 | 9.933 | up | protein TRANSPORT INHIBITOR RESPONSE 1 |
| Unigene0027945 | 7.419 | 24.266 | up | auxin-induced protein 22D |
| Unigene0030711 | 0.036 | 10.302 | up | indole-3-acetic acid-amido synthetase GH3.6 |
| Unigene0036400 | 1.449 | 20.033 | up | probable indole-3-acetic acid-amido synthetase GH3.1 |
| Unigene0002914 | 0.152 | 6.515 | up | probable indole-3-acetic acid-amido synthetase GH3.1 |
| Unigene0012516 | 0.001 | 3.874 | up | auxin-responsive protein SAUR50-like |
| Unigene0028447 | 5.827 | 51.809 | up | auxin response factor 5 |
| Unigene0015734 | 0.001 | 1.905 | up | indole-3-acetic acid-amido synthetase GH3.6-like |
| Unigene0000921 | 0.001 | 1.652 | up | auxin-responsive protein SAUR21 |
| Unigene0054849 | 0.001 | 2.343 | up | auxin-responsive protein SAUR32-like |
| Unigene0018363 | 0.001 | 1.273 | up | auxin-responsive protein SAUR21 |
| Unigene0027380 | 25.967 | 4.157 | domn | auxin-responsive protein IAA4 |
| Unigene0030569 | 23.066 | 8.147 | domn | auxin-induced protein IAA6 |
| Unigene0003051 | 372.577 | 70.228 | down | SAUR-like auxin-responsive protein family |
| Gibberellic acid-related genes | | | | |
| Unigene0005738 | 0.388 | 5.017 | up | DELLA protein GAI1 |
| Unigene0031663 | 0.856 | 7.077 | up | DELLA protein GAI1 |
| Unigene0046182 | 24.744 | 63.940 | up | DELLA protein GAI |
| Brassinolide-related genes | | | | |
| Unigene0040040 | 8.430 | 23.112 | up | probable serine/threonine-protein kinase At5g41260 |
| Unigene0004470 | 3.026 | 15.049 | up | probable serine/threonine-protein kinase At4g35230 |
| Unigene0000915 | 0.485 | 25.824 | up | probable serine/threonine-protein kinase At5g41260 |
| Unigene0034810 | 1.857 | 5.333 | up | probable serine/threonine-protein kinase At5g41260 |
| Unigene0030389 | 11.222 | 27.881 | up | shaggy-related protein kinase eta-like |
| Unigene0025032 | 27.239 | 88.820 | up | probable xyloglucan endotransglucosylase/hydrolase protein 23 |
| Unigene0035058 | 0.050 | 30.895 | up | cyclin-D3-1 |
| Unigene0038596 | 0.128 | 17.534 | up | cyclin-D3-1 |
| Jasmonic acid-related genes | | | | |
| Unigene0049406 | 3.133 | 36.488 | up | protein TIFY 9 |
| Unigene0033693 | 0.902 | 6.907 | up | transcription factor MYC2 |
