# Supplementary material for: Transcriptomic analysis of flower induction for long-day pitaya by supplementary lighting in short-day winter season
Source: BMC Genomics. 2020 Apr 29;21:329. doi: 10.1186/s12864-020-6726-6 (PMC7191803; doi:10.1186/s12864-020-6726-6)
Supplement: Supplementary file 10 — Additional file 10: Supplemental S10. The DEGs of CO and FT. [file 12864_2020_6726_MOESM10_ESM.docx]

Supplemental S10 The DEGs of *CO* and *FT*

| **Gene ID** | **NL rpkm** | **L0 rpkm** | **up or down** | **function** |
| --- | --- | --- | --- | --- |
| CO gene | | | | |
| Unigene0025406 | 149.9579 | 66.03707 | down | zinc finger protein CONSTANS-LIKE 2 |
| **Gene ID** | **NL rpkm** | **L1 rpkm** | **up or down** | **function** |
| CO gene | | | | |
| Unigene0023462 | 0.652333 | 3.625 | up | zinc finger protein CONSTANS-LIKE 6 |
| Unigene0027129 | 1.906733 | 9.5895 | up | zinc finger protein CONSTANS-LIKE 15 |
| Unigene0035118 | 3.8895 | 25.40467 | up | zinc finger protein CONSTANS-LIKE 15 |
| Unigene0025406 | 149.9579 | 28.9243 | down | zinc finger protein CONSTANS-LIKE 2 |
| Unigene0027332 | 152.9907 | 65.17177 | down | zinc finger protein CONSTANS-LIKE 9-like |
| Unigene0028118 | 248.362 | 76.02927 | down | zinc finger protein CONSTANS-LIKE 4 |
| Unigene0029386 | 134.6283 | 18.51783 | down | zinc finger protein CONSTANS-LIKE 2 |
| Unigene0030366 | 19.68613 | 5.715667 | down | zinc finger protein CONSTANS-LIKE 14 isoform X2 |
| Unigene0034031 | 13.20053 | 3.434533 | down | zinc finger protein CONSTANS-LIKE 16 |
| Unigene0034048 | 119.6957 | 45.3622 | down | zinc finger protein CONSTANS-LIKE 13 isoform X1 |
| Unigene0035131 | 32.49693 | 2.677033 | down | zinc finger protein CONSTANS-LIKE 6-like |
| Unigene0043102 | 60.54423 | 20.04363 | down | zinc finger protein CONSTANS-LIKE 5 |
| Unigene0050191 | 16.53933 | 5.908433 | down | zinc finger protein CONSTANS-LIKE 5 |
| FT gene | | | | |
| Unigene0001751 | 2.422633 | 5.448933 | up | flowering locus T |
| Unigene0005649 | 0.150267 | 6.409367 | up | flowering locus T |
| **Gene ID** | **L0 rpkm** | **L1 rpkm** | **up or down** | **function** |
| CO gene | | | | |
| Unigene0023462 | 0.491167 | 3.625 | up | zinc finger protein CONSTANS-LIKE 6 |
| Unigene0027129 | 1.44 | 9.5895 | up | zinc finger protein CONSTANS-LIKE 15 |
| Unigene0035118 | 7.1763 | 25.40467 | up | zinc finger protein CONSTANS-LIKE 15 |
| Unigene0025406 | 66.03707 | 28.9243 | down | zinc finger protein CONSTANS-LIKE 2 |
| Unigene0029386 | 71.0612 | 18.51783 | down | zinc finger protein CONSTANS-LIKE 2 |
| Unigene0030366 | 19.58523 | 5.715667 | down | zinc finger protein CONSTANS-LIKE 14 isoform X2 |
| Unigene0035131 | 14.9491 | 2.677033 | down | zinc finger protein CONSTANS-LIKE 6-like |
| FT gene | | | | |
| Unigene0002836 | 0.001 | 2.429767 | up | FT-interacting protein 1 |
| Unigene0006372 | 0.001 | 2.0228 | up | FT-interacting protein 1 |
| Unigene0025249 | 0.001 | 1.940967 | up | FT-interacting protein 1 |
| Unigene0038103 | 1.371767 | 10.13167 | up | FT-interacting protein 1 |
| Unigene0042376 | 0.258433 | 8.6344 | up | FT-interacting protein 1 |
| Unigene0045680 | 10.5155 | 46.86363 | up | FT-interacting protein 1 |
| Unigene0025148 | 3.187833 | 0.839333 | down | FT-interacting protein 1 |
