# Supplementary material for: Transcriptomic analysis of flower induction for long-day pitaya by supplementary lighting in short-day winter season
Source: BMC Genomics. 2020 Apr 29;21:329. doi: 10.1186/s12864-020-6726-6 (PMC7191803; doi:10.1186/s12864-020-6726-6)
Supplement: Supplementary file 11 — Additional file 11: Supplemental S11. Three main TFs involved in pitaya flowering stage. [file 12864_2020_6726_MOESM11_ESM.docx]

Supplemental S11 Three main TFs involved in pitaya flowering stage

| **Gene ID** | **NL rpkm** | **L0 rpkm** | **up or down** | **function** |
| --- | --- | --- | --- | --- |
| CDF | | | | |
| Unigene0033003 | 27.3217 | 8.099567 | down | cyclic dof factor 2 |
| Unigene0031426 | 85.15217 | 35.44767 | down | cyclic dof factor 1 |
| **Gene ID** | **NL rpkm** | **L1 rpkm** | **up or down** | **function** |
| CDF | | | | |
| Unigene0006016 | 0.399733 | 5.343767 | up | dof zinc finger protein DOF1.5-like |
| Unigene0029432 | 0.2407 | 4.818767 | up | cyclic dof factor 2 |
| Unigene0026114 | 26.6774 | 11.26 | down | cyclic dof factor 3-like |
| Unigene0031426 | 85.15217 | 14.87807 | down | cyclic dof factor 1 |
| Unigene0033003 | 27.3217 | 6.380667 | down | cyclic dof factor 2 |
| MADS-box | | | | |
| Unigene0018425 | 0.2315 | 1.668767 | up | agamous-like MADS-box protein AGL16 |
| Unigene0021927 | 0.7743 | 4.316467 | up | agamous-like MADS-box protein AGL9 homolog |
| Unigene0025394 | 0.870533 | 5.236733 | up | agamous-like MADS-box protein AGL12 |
| Unigene0037726 | 0.775733 | 12.987 | up | MADS-box transcription factor 23-like |
| Unigene0047376 | 0.560033 | 2.319367 | up | Agamous-like MADS-box protein AGL8 |
| Unigene0022108 | 0.200367 | 1.516733 | up | floral homeotic protein AGAMOUS-like |
| Unigene0027094 | 19.5641 | 9.1122 | down | agamous-like MADS-box protein AGL19 |
| TCP | | | | |
| Unigene0005880 | 3.9476 | 18.5369 | up | transcription factor TCP15 |
| Unigene0009351 | 0.9384 | 9.448333 | up | transcription factor TCP4 |
| Unigene0025734 | 0.4759 | 7.736333 | up | transcription factor TCP18 |
| Unigene0028812 | 2.652833 | 13.84017 | up | transcription factor TCP14 |
| Unigene0030793 | 6.8182 | 15.31663 | up | transcription factor TCP9 |
| Unigene0033768 | 0.001 | 5.124867 | up | transcription factor TCP4 |
| **Gene ID** | **L0 rpkm** | **L1 rpkm** | **up or down** | **function** |
| CDF | | | | |
| Unigene0006016 | 0.226167 | 5.343767 | up | dof zinc finger protein DOF1.5-like |
| Unigene0029432 | 0.608433 | 4.818767 | up | cyclic dof factor 2 |
| Unigene0031426 | 35.44767 | 14.87807 | down | cyclic dof factor 1 |
| MADS-box | | | | |
| Unigene0027094 | 20.49717 | 9.1122 | down | agamous-like MADS-box protein AGL19 |
| Unigene0037726 | 3.471367 | 12.987 | up | MADS-box transcription factor 23-like |
