# Supplementary material for: Transcriptomic analysis of flower induction for long-day pitaya by supplementary lighting in short-day winter season
Source: BMC Genomics. 2020 Apr 29;21:329. doi: 10.1186/s12864-020-6726-6 (PMC7191803; doi:10.1186/s12864-020-6726-6)
Supplement: Supplementary file 12 — Additional file 12: Supplemental S12. A flowchart of the RNAseq process. [file 12864_2020_6726_MOESM12_ESM.doc]

Supplemental S12 A flowchart of the RNAseq process
